# Fibroblasts derived from oesophageal adenocarcinoma differ in DNA methylation profile from normal oesophageal fibroblasts

**DOI:** 10.1038/s41598-017-03501-6

**Published:** 2017-06-13

**Authors:** Eric Smith, Helen M. Palethorpe, Annette L. Hayden, Joanne P. Young, Timothy J. Underwood, Paul A. Drew

**Affiliations:** 1 Discipline of Surgical Specialities, Adelaide Medical School, Faculty of Health Sciences, The University of Adelaide, South Australia, 5000 Australia; 2 Department of Haematology and Oncology, The Queen Elizabeth Hospital, Woodville, South Australia 5011 Australia; 3 Cancer Sciences Unit, Somers Cancer Research Building, University of Southampton, Southampton General Hospital, Tremona Road, Southampton, SO16 6YD UK; 4 School of Nursing and Midwifery, Flinders University, PO Box 2100, Adelaide, South Australia 5001 Australia

## Abstract

Oesophageal adenocarcinoma (OAC) is increasing in incidence and has a poor prognosis. Tumour derived fibroblasts (TDFs) differ functionally from normal fibroblasts (NDFs), and play a pivotal role in cancer. Many of the differences persist through subculture. We measured the DNA methylation profiles of 10 TDFs from OAC with 12 NDF from normal oesophageal mucosa using Infinium HumanMethylation450 Beadchips and found they differed in multidimensional scaling analysis. We identified 4,856 differentially methylated CpGs (DMCs, adjusted p < 0.01 and absolute difference in average β-value > 0.15), of which 3,243 (66.8%) were hypomethylated in TDFs compared to NDFs. Hypermethylated DMCs were enriched at transcription start sites (TSSs) and in CpG islands, and depleted in transcriptional enhancers. Gene ontology analysis of genes with DMCs at TSSs revealed an enrichment of genes involved in development, morphogenesis, migration, adhesion, regulation of processes and response to stimuli. Alpha-smooth muscle actin (*α*-SMA) is a marker of activated fibroblasts and a poor prognostic indicator in OAC. Hypomethylated DMCs were observed at the TSS of transcript variant 2 of α-SMA, which correlated with an increase in *α-*SMA protein expression. These data suggest that DNA methylation may contribute to the maintenance of the TDF phenotype.

## Introduction

Oesophageal adenocarcinoma (OAC), which has increased rapidly in incidence in the Western world over recent decades^1^, has a five year survival rate of about 15%^2^. Most patients are unsuitable for treatment with curative intent. The major risk factors include gastro-oesophageal reflux disease and obesity, which lead to the premalignant condition, Barrett’s oesophagus, the only described precursor lesion for OAC. A deeper understanding of the mechanisms that regulate the development and progression of OAC may lead to improvements in early diagnosis and treatment.

An emerging body of evidence demonstrates that fibroblasts play a significant role in the development and progression of solid tumours (reviewed in ref. 3). Within a cancer they are a phenotypically heterogeneous population of cells, distinct from the fibroblasts found in normal tissue, and are referred to as activated, cancer associated, or tumour derived fibroblasts (reviewed in ref. 4). These have been shown to promote tumour growth, facilitate tumour cell invasion, migration and metastasis, promote therapeutic drug resistance and act to prevent immune cell infiltration. Expression signatures that characterise these fibroblasts are associated with poor survival outcomes in many solid tumour types including OAC^5–10^.

A number of studies have reported that many of the phenotypic characteristics of tumour derived fibroblasts (TDFs) are maintained in culture^11, 12^. This is consistent with at least some of the phenotypic alterations being maintained by epigenetic mechanisms such as DNA methylation^13–15^, which involves the covalent addition of a methyl group to, most commonly, the cytosine residue of a cytosine-phosphate-guanine (CpG) dinucleotide. Regions of the genome with a relatively high density of CpGs, CpG islands, and their flanking shores and shelves are associated with 60–70% of all human genes^16^. Methylation at the transcription start site (TSS) or within the body of genes is frequently associated with the silencing of transcription, and methylation of transcriptional enhancers may also affect gene transcription^17^. Aberrant methylation in intergenic regions has been associated with genomic instability or global silencing of large chromatin domains. Whilst genome-wide DNA methylation profiles of many tumour types, including OAC^18–22^, have been ascertained, these studies have been conducted using whole tissue samples or cancer cell lines. There are reports of the genome-wide DNA methylation profiles of TDFs in breast^13^, gastric^23^, colorectal^14^, and non-small cell lung carcinoma^15^, but none in OAC.

The aim of this study was to compare the genome-wide DNA methylation profiles of low-passage primary TDFs from patients with OAC to fibroblasts derived from macroscopically normal oesophageal squamous mucosa. We show that the TDFs have a DNA methylation profile which distinguishes them from most NDFs. Differentially methylated CpGs were observed at TSSs of genes which have a known role in cancer development and progression, suggesting that the TDF phenotype may be regulated, at least in part, by epigenetic mechanisms.

## Results

### Tumour derived fibroblasts were aberrantly methylated

Twenty-two primary fibroblast lines were established from resected specimens of 16 patients with oesophageal cancer (Supplementary Table S1). There were 10 TDFs and 12 NDFs, which included six patient matched fibroblast pairs. The median age of the patients was 65 years (range 57 to 82). There was not a significant difference in the age of the patients from whom the TDFs and NDFs were established. There were 13 males and three females. Five patients were treated with surgery alone, and 11 received a combination of neoadjuvant chemotherapy and surgery.

**Figure 1:**
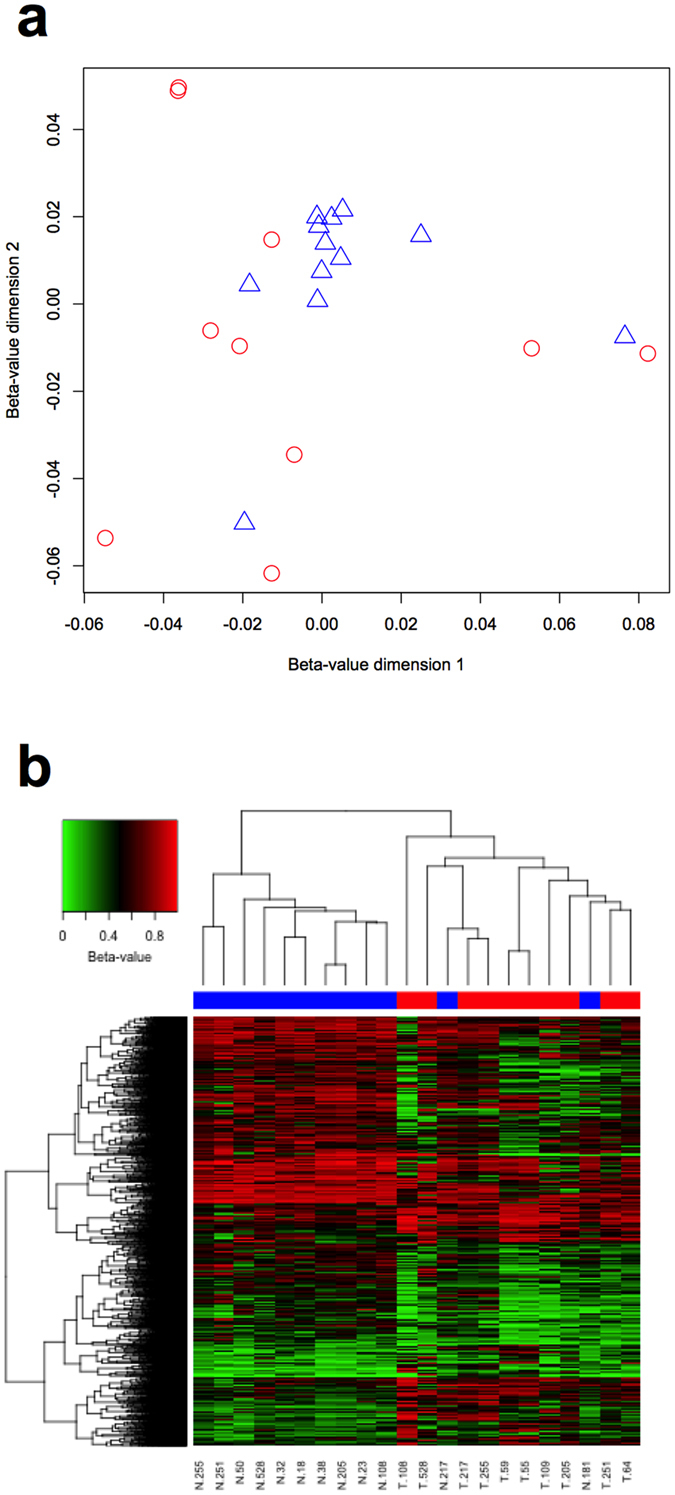
Genome-wide DNA methylation profiles of TDFs and NDFs. (**a**) Multidimensional scaling performed using the β-values for all 408,329 probes for NDFs (blue triangles) and TDFs (red circles). (**b**) Hierarchical clustering using the 4,856 DMC for NDFs (blue) and TDFs (red).

The genome-wide DNA methylation profile of the fibroblasts was measured using the Infinium HumanMethylation450 Beadchip. Unsupervised pairwise multidimensional scaling was performed using the β-values for all 408,329 probes included in the analysis (Fig. 1a). The distribution of the TDFs differed from NDFs. The NDFs formed a tight cluster, with two outliers. In contrast, the TDFs were more widely dispersed. The coefficient of variation (CV) for the median β-values of each fibroblast was 7.6% for the NDFs and 10.2% for the TDFs, but the variance of median β-values of each fibroblast was not significantly different (p = 0.1836). Comparing methylation in the TDFs and NDFs, there were 4,856 DMCs, of which 3,243 (66.8%) were hypomethylated and 1,613 (33.2%) hypermethylated. Hierarchical clustering of these 4,856 DMCs revealed that the fibroblasts formed two major clusters, with 10 of the 12 NDF clustering together, the remaining two NDF (N.181 and N.217) within the TDF cluster (Fig. 1b).

### Differentially methylated CpGs and functional genomic regions

We analysed the distribution of the DMCs between the functional genomic regions. The probes were allocated as TSS1500, TSS200, 5′UTR, 1st exon, gene body or 3′UTR according to the Illumina probe annotation^24^. Many probes are annotated to more than one genomic region since a locus may be within more than one gene, or more than one variant of a gene, so that the sum of the loci in genomic locations is greater than the number of probes analysed. Probes which were not annotated to a gene region were categorised as intergenic. The results in Table 1 show the proportion of all CpGs analysed and DMCs in each of these regions. There was a significant difference in the distribution of the DMCs across the functional genomic locations compared to that of all the cytosines analysed (Chi square test for proportions: p < 0.0001). The most significant differences were a depletion around the TSS, particularly the TSS200 (3.6% of DMCs compared to 11.6% of all analysed) and the first exon (2.2% v 7.2%), and an enrichment in the intergenic region (32.0% v 19.5%). Overall there were significantly fewer differentially methylated cytosines associated with the promoter region (defined as TSS1500, TSS200, 5′UTR and 1st Exon; 27.9% versus 46.1%). There were no significant differences in the distribution of DMCs within the annotated microRNAs or lncRNAs. The proportion of hypomethylated and hypermethylated DMCs differed between the gene regions (Table 2). Hypermethylated DMCs were more frequent in the TSS200 and 3′UTR, and less in the gene body and intergenic regions.

**Table 1:** The proportion of all CpGs analysed and differentially methylated cytosines (DMC) in each annotated region.

	DMC (%)	All CpGs Analysed (%)	OR (95% CI)	p-value
**Gene Regions**				
Total^a^	5,302	479,691		
TSS1500	682 (12.9%)	73,530 (15.3%)	0.8137 (0.7505–0.8822)	<0.0001
TSS200	192 (3.6%)	55,640 (11.6%)	0.2839 (0.2457–0.3280)	<0.0001
5′UTR	488 (9.2%)	57,408 (12.0%)	0.7435 (0.6771–0.8164)	<0.0001
1^st^ Exon	117 (2.2%)	34,391 (7.2%)	0.2898 (0.2415–0.3482)	<0.0001
Gene body	1,954 (36.9%)	148,809 (31.0%)	1.302 (1.231–1.377)	<0.0001
3′UTR	173 (3.3%)	16,571 (3.5%)	0.9421 (0.8089–1.097)	0.4652
Intragenic	1,686 (32.0%)	93,342 (19.5%)	1.947 (1.837–2.064)	<0.0001
microRNA	33 (0.6%)	2,331 (0.5%)	0.9995 (0.7114–1.404)	>0.999
lncRNA	4 (0.08%)	429 (0.01%)	0.658 (0.2562–1.675)	0.5835
**CpG Island Regions**				
Total	4,856	408,329		
CGI	453 (9.3%)	133,415 (32.7%)	0.2093 (0.1900–0.2306)	<0.0001
Shores	1,208 (24.9%)	97,243 (23.8%)	1.060 (0.9929–1.132)	0.0836
Shelves	488 (10.0%)	37,691 (9.2%)	1.100 (1.001–1.209)	0.0502
Open sea	2,707 (55.7%)	139,980 (34.3%)	2.443 (2.307–2.586)	<0.0001
**Enhancer Regions**				
Non-enhancer	2,608 (53.7%)	317,333 (77.7%)		
Enhancer	2,248 (46.3%)	90,996 (22.3%)	3.057 (2.888–3.246)	<0.0001

^a^Probes may annotate to more than one gene region.

**Table 2:** The percentage of hypermethylated or hypomethylated differentially methylated cytosines (DMC) in each or the annotated region.

	Hypermethylated (%)	Hypomethylated (%)	Total	OR (95% CI)	p-value
**Total**	1,613 (33.2%)	3,243 (66.8%)	4,856		
**Gene Regions**					
TSS1500	237 (34.8%)	445 (65.2%)	682	1.083 (0.9133–1.284)	0.3823
TSS200	86 (44.8%)	106 (55.2%)	192	16.78 (13.59–20.72)	<0.0001
5′UTR	166 (34.0%)	322 (66.0%)	488	1.041 (0.8540–1.268)	0.7302
1^st^ Exon	39 (33.3%)	78 (66.7%)	117	1.005 (0.6813–1.484)	0.9424
Gene body	705 (36.1%)	1,249 (63.9%)	1,954	0.7294 (0.6510–0.8173)	<0.0001
3′UTR	77 (44.5%)	96 (55.5%)	173	1.643 (1.210–2.232)	0.0018
Intragenic	468 (27.6%)	1,228 (72.4%)	1,696	0.6707 (0.5896–0.7629)	<0.0001
**CpG Island Regions**					
CGI	270 (59.6%)	183 (40.3%)	453	1.954 (1.790–2.134)	<0.0001
Shores	489 (40.5%)	719 (59.5%)	1,208	1.314 (1.208–1.429)	<0.0001
Shelves	138 (28.3%)	350 (71.7%)	488	0.8374 (0.7227–0.9703)	<0.0001
Open sea	716 (26.4%)	1,991 (73.6%)	2,707	0.6337 (0.5848–0.6866)	<0.0001
**Enhancer Regions**					
Non-enhancer	976 (37.4%)	1,632 (62.6%)	2,608		
Enhancer	637 (28.3%)	1,611 (71.7%)	2,248	0.6612 (0.5856–0.7464)	<0.0001

### Differentially methylated CpGs and CpG islands

CpG islands are important genomic regulatory elements that are defined by a high density of CpGs relative to entire genome. The regions 2 kilobases either side of an island are defined as shores, the 2 kilobase regions flanking the shores are defined as shelves^24^, and here we define the remainder of the genome as open seas. The distribution of DMCs in the context of CpG islands is shown in Table 1. Of all the CpGs for analysis, 65.7% were in islands, shores or shelves, compared to 44.3% of the DMCs. Within the CpG islands DMCs were significantly depleted (9.3% v 32.7% of all analysed cytosines), but there was no significant difference in the distribution of DMCs in the shores or shelves. There was a significantly greater proportion of DMCs in the open seas (55.7% v 34.3%). There was significant enrichment of hypermethylated DMCs in CpG islands and adjacent shores, and depletion in shelves and open seas (Table 2).

We then determined if there were a difference in the distribution of DMCs between CpG islands that overlap annotated genes and those located in the intergenic regions. An island was classified as intragenic if any of its CpGs were in an annotated gene region (that is, within the TSS1500 to 3′UTR regions). Of the DMCs within CpG islands, a significantly greater proportion were in islands in the intergenic regions (34.2% v 13.8% of all CpGs, odds ratio (OR) 3.276, 95% confidence interval (CI) 2.696–3.981, p < 0.0001), and lesser in islands which overlapped genes (31.8% v 70.0%, p < 0.0001). The proportion of hypermethylated DMCs within CpG islands did not significantly vary between intergenic and intragenic CpG islands (62.1% and 54.8% respectively, OR 1.389, 95% CI 0.9096–1.998, p = 0.1647).

### Differentially methylated CpGs and enhancer regions

Next, we investigated the distribution of DMCs between enhancer and non-enhancer regions. Of the total of 408,329 CpGs for analysis, 90,996 (22.3%) were in enhancer regions. The DMCs were significantly enriched in enhancer (46.3% of DMCs compared to 22.3% of all analysed, p < 0.0001) compared to non-enhancer regions (53.7% v 77.7%) (Table 1). The proportion of hypermethylated DMCs was significantly lower in enhancer compared to non-enhancer regions (Table 2; p < 0.0001). Further analysis of the DMCs in enhancers revealed that they were enriched in the intergenic compared to intragenic regions (57.9% versus 40.1% respectively, OR 2.058, 95% CI 1.825–2.320, p < 0.0001). The proportion of hypermethylated DMCs in enhancers was greater in those in intragenic compared to intergenic regions (31.9% and 23.7% respectively, OR 1.507, 95% CI 1.248–1.919, p < 0.0001). The proportion of hypermethylated DMCs in non-enhancer regions was greater in the intragenic compared to intergenic regions (39.1% and 32.9% respectively, OR 1.310, 95% CI 1.093–1.570, p = 0.0040).

### Methylation of ACTA2 correlated with decreased *α*-SMA protein expression

To ascertain the potential functional significance of the observed DMC, we conducted gene ontology enrichment analyses using genes that had one or more DMCs located within 1,500 bases of their TSS. Of the 4,856 DMCs, 1,354 (27.9%) were located within 1,500 bases of a TSS, representing 1,145 unique Entrez Gene IDs. Of these, 743 (64.9%) were hypomethylated in TDFs, and 402 (35.1%) were hypermethylated. Hypermethylated DMCs were observed about the TSS of genes predominantly involved in development, morphogenesis and migration, whilst genes with hypomethylated DMCs were involved in regulation of processes, response to stimuli, development and adhesion (Supplementary Table S2).

**Figure 2:**
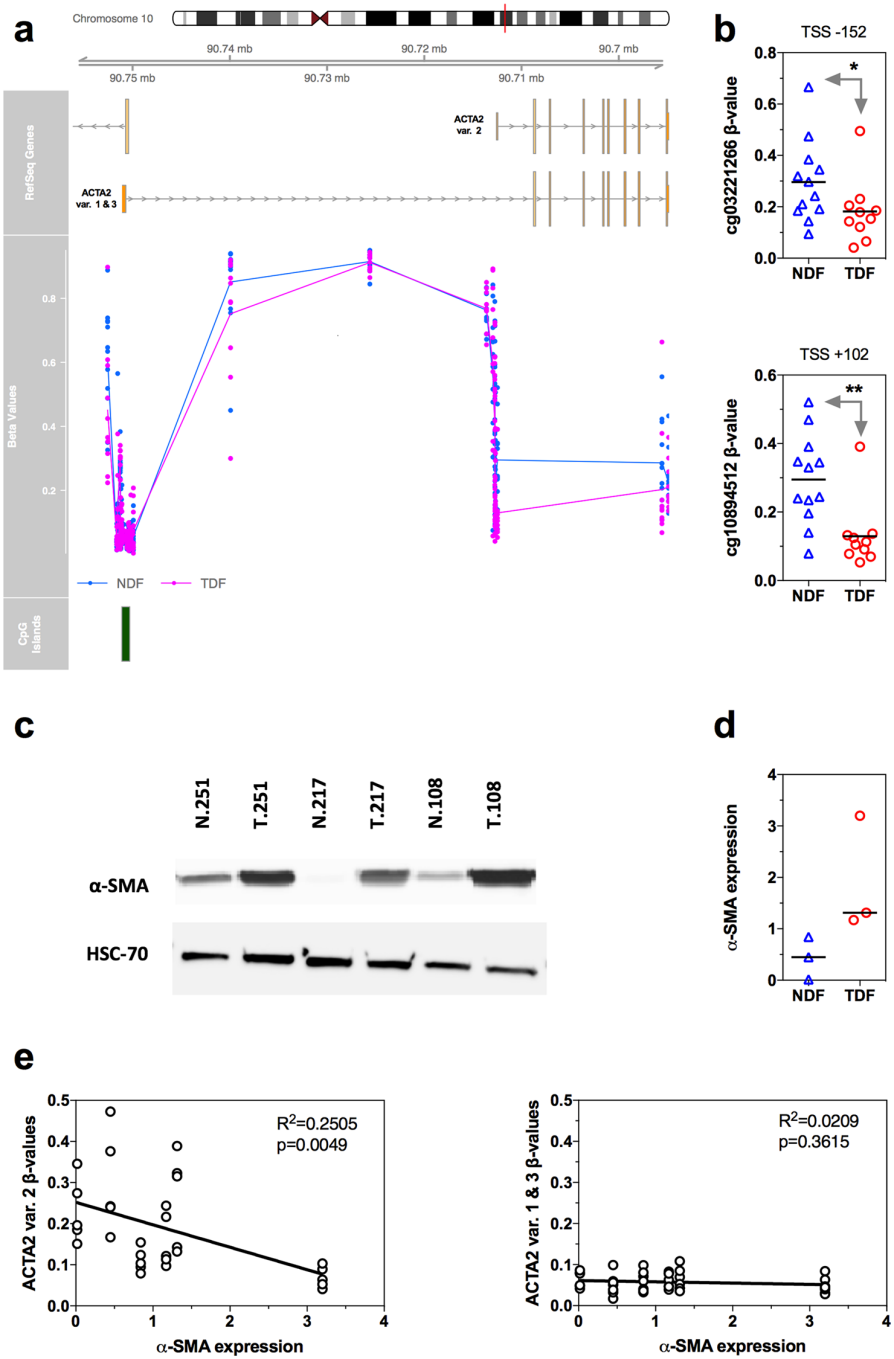
DNA methylation and expression of α-SMA (ACTA2). (**a**) The relative location of ACTA2 splice variants, individual β-values for all NDFs (blue circles) and TDFs (pink circles), and CpG islands. The lines for the β-values represent the average β-value for the NDF and TDF groups. (**b**) The β-values for all individual NDF and TDF samples for the probes cg03221266 and cg10894512 located at positions −152 bp or +102 bp respectively of the TSS of ACTA2 variant 2. *Benjamini-Hochberg adjusted p = 8.55 × 10^−9^, **Benjamini-Hochberg adjusted p = 2.46 × 10^−19^. (**c**) Western immunoblot for α-SMA and the loading control HSC-70 for the three available patient matched pairs of NDFs (N.251, N.217 and N.108) and TDFs (T.251, T.217 and T.108). (**d**) Quantification of α-SMA protein expression for the three patient matched pairs. (**e**) Correlation between α-SMA protein expression and β-values for the probes about ACTA2 TSS of the splice variants for the three patient matched pairs.

A gene which featured in several enriched biological processes was ACTA2. Multiple alternatively spliced variants of ACTA2 have been reported, and they each encode the same protein, alpha-smooth muscle actin (*α*-SMA). Variant 2 varies from the other variants by an alternate TSS (Fig. 2a). We observed that the region about the TSS for transcript variant 2 was hypomethylated in TDFs compared to NDFs (Fig. 2a and b). In contrast, the β-values for the probes about the TSS of variant 1 and 3 varied little between TDFs and NDFs, and were relatively low (β-value < 0.15). Sufficient material was available from three patient matched fibroblast pairs to analyse the expression of *α*-SMA by western immunoblot. The results confirmed that *α*-SMA was elevated in these TDFs compared to the NDFs (Fig. 2c and d). Methylation about the TSS of variant 2, but not variant 1 and 3, inversely correlated with *α*-SMA protein expression (Fig. 2e), suggesting that the low *α*-SMA expression observed in cultured oesophageal NDFs was associated with DNA methylation about the TSS of variant 2.

## Discussion

This is the first study to compare the genome-wide DNA methylation profiles of oesophageal NDFs to OAC TDFs using the high resolution Infinium HumanMethylation450 BeadChip. Multidimensional scaling analysis of all probes analysed showed that, with respect to DNA methylation, the NDFs clustered tightly apart from two outliers, whereas the TDFs were markedly heterogeneous. Hierarchical clustering using the 4,856 DMCs demonstrated that the TDFs grouped differently to the NDFs. Detailed examination of the genomic locations of the DMCs revealed significant regional variation in DNA methylation between the two fibroblast groups. In TDFs, the DMCs were depleted about the transcription start sites and in CpG islands and enriched in gene bodies, open seas and in enhancers. The DMCs were observed in the TSSs of genes which have a known role in cancer development and progression. Methylation was significantly decreased at the TSS of variant 2 of *α*-SMA, which correlated with an increase in *α*-SMA protein expression.

Previous studies have investigated DNA methylation profiles of TDFs in breast^13^, gastric^23^, colorectal^14^, and non-small cell lung carcinoma^15^. Consistent with our findings, these studies demonstrated differences in DNA methylation between TDF and NDFs, with general DNA hypomethylation and concomitant focal hypermethylation observed in TDFs compared to NDFs. Only one used the Infinium HumanMethylation450 BeadChip^15^, and reported a strikingly similar distribution of DMCs across the functional genomic regions, including the depletion about TSSs and CpG islands, and the enrichment in gene bodies and open seas. In addition, we report the novel observation of differential methylation in transcriptional enhancers. Multiple enhancers may cooperate to finely tune the expression of a single transcript, and integrate extracellular signals with intracellular cell fate information to generate cell type-specific transcriptional responses^25^. Together, these results suggest that differences in DNA methylation, through their role in regulation of gene expression, contribute to the alterations in fibroblast phenotypes observed in cancer.

The results from the multidimensional scaling of all CpGs analysed and the hierarchical clustering of DMCs showed that the DNA methylation profiles of the TDFs were markedly more heterogeneous than the NDFs. The primary function of fibroblasts is to establish, maintain, and modify connective tissue^26^. They are a heterogeneous population of cells, particularly in disease. The origin of TDFs can be from resident fibroblasts, as well as infiltrating cells, including epithelial, endothelial, and bone marrow-derived mesenchymal stem cells^27^ and fibrocytes^15, 28^. They can exist in differing states of activation and functional potential^29–31^. It is therefore highly likely that primary cultures of TDFs contain differing proportions of fibroblast subpopulations. The heterogeneity of their DNA methylation profiles most likely reflects the heterogeneity of their origins and functions in cancer.

Expression of α-SMA is commonly used as a marker for TDFs, and is associated with poor prognosis in a range of cancers, including OAC^10, 31^, oesophageal squamous cell carcinoma^32^, colorectal^6^, breast^33^, and head and neck cancers^34^. In humans, the *α*-SMA protein is encoded by the ACTA2 gene, and transcript variant 2 varies from 1 and 3 by an alternate TSS, with the entire first exon of each variant being a 5′UTR. We observed the novel finding that DNA methylation about the TSS of variant 2 inversely correlated with *α*-SMA protein expression. This raises the possibility that methylation of this region may be of functional significance in repressing *α*-SMA expression in oesophageal fibroblasts. In rat lung fibroblasts, myofibroblasts, and alveolar epithelial type cells, methylation of the ACTA2 promoter inversely correlated with expression^35^. In addition, inhibition of DNMT activity led to significant induction of *α*-SMA expression, while ectopic expression of DNMTs suppressed its expression, suggesting that DNA methylation plays a key role in the regulation of *α*-SMA gene expression during myofibroblast differentiation^35^. Further experiments confirming the functional significance of the observed methylation are warranted, considering the prognostic significance of *α*-SMA expression.

It is possible that neoadjuvant chemotherapy might have altered the DNA methylation profiles in either of the normal or cancer associated fibroblasts. To the best of our knowledge, there are no studies that demonstrate this in fibroblasts, although several reports suggest that this may occur in cancer cells^36, 37^. Future studies to compare the DNA methylation of fibroblasts before and after chemotherapy would require the harvesting of sufficient fibroblasts from the small amount of tissue obtainable by biopsy.

In conclusion, we compared the genome-wide DNA methylation profiles of 10 TDFs from oesophageal adenocarcinoma tumour tissues with 12 NDFs from macroscopically normal oesophageal mucosa using Infinium HumanMethylation450 Beadchips. The genome-wide DNA methylation profile of TDFs differed significantly from that of NDFs. The focal distribution of the DMCs about the transcription start sites and within CpG islands and transcriptional enhancers may, by the regulation of gene expression, contribute to the establishment and maintenance of the TDF phenotype *in vitro* and *in vivo*.

## Methods

### Research Ethics

All methods were carried out in accordance with relevant guidelines and regulations.

All experimental protocols were approved by the Southampton and South West Hampshire Research Ethics Committee (09/H0504/66). Informed consent was obtained from all subjects.

### Primary human oesophageal fibroblasts

Primary human oesophageal fibroblast lines were established as described previously^38^. Briefly, macroscopically normal squamous mucosa and tumour tissues were sampled from resection specimens and transported in Hank’s balanced salt solution (Invitrogen, Carlsbad, CA, USA). Tissues were washed twice in Dulbecco’s phosphate buffered saline (DPBS; Invitrogen), placed in fresh DPBS supplemented with 250 ng/ml amphotericin B (Invitrogen), and diced into 2 mm^3^ pieces. Single fragments of tissue were then placed into individual wells of six-well plates, and cultured at 37 °C in a humidified atmosphere with 10% CO_2_. The fibroblast culture medium was composed of Dulbecco’s modified Eagle’s medium (Invitrogen) supplemented with 10% (v/v) fetal bovine serum (Autogen Bioclear UK Ltd, Wiltshire, UK or Sigma-Aldrich, St Louis, MO, USA), 100 units/ml penicillin, 100 μg/ml streptomycin, 250 ng/ml amphotericin B and 292 μg/ml L-glutamine (Invitrogen). The primary fibroblasts were expanded by subculturing in fibroblast medium, on tissue culture treated plastic, at 37 °C in a humidified atmosphere with 5% CO_2_. The phenotype of *ex-vivo* fibroblasts was confirmed as vimentin-positive, cytokeratin-negative, CD31-negative and desmin-negative, as described previously^38^.

### Genome-wide DNA methylation profiling

Genomic DNA was isolated from the primary fibroblasts at the earliest subculture that sufficient cells were available. The DNA was isolated using either the DNeasy Blood and Tissue Kit (Qiagen, Hilden, Germany) or Trizol (Invitrogen), and concentrated, if required, using the phenol chloroform ethanol precipitation method. The DNA (500–2000 ng) was bisulphite modified with the EZ DNA Methylation-Gold Kit (Zymo Research, Irvine, CA, USA), as described previously^39, 40^. The bisulphite-modified DNA was hybridized onto Infinium HumanMethylation450 BeadChips following the Illumina Infinium HD Methylation protocol, and scanned using an Illumina HiScan SQ scanner (Illumina, San Diego, CA, USA), as described previously^41^.

Raw fluorescence intensity values were normalised using the GenomeStudio Methylation Module (v1.8.5; Illumina), with background subtraction and normalisation to internal controls. Normalised intensities were used to calculate β-values. The β-value represents the percentage of the cytosines at that locus which were methylated, and ranges from 0 (no methylation) to 1 (complete methylation). The average β-value at each locus was calculated for the NDF and TDF groups.

Probes were excluded from the analysis if they did not target a cytosine within a CpG, or if they were known to align to a single nucleotide polymorphism (SNP) or to multiple locations^42^, or if its target cytosine was two or fewer nucleotides from a known SNP for which the SNP had a minor allele frequency above 0.05^43^, or if the detection p value, which defines the chance that the target signal was not distinguishable from background, was greater than 0.01 in any sample, or if the bead count was less than three. Probes on the X and Y chromosomes were also excluded.

Differentially methylated CpGs (DMCs) between the TDF and NDF groups were determined using the Illumina Custom Model in the GenomeStudio Methylation Module with false discovery rate (FDR) adjustment. The software calculates a p value for the significance of the difference in β-values between the groups for each locus, corrected for multiple testing using the Benjamini-Hochberg FDR adjustment. A CpG was considered to be differentially methylated if p < 0.01 and the absolute difference in the average β-values of each group was >0.15. A DMC was defined to be hypermethylated if the average β-value for the TDFs was greater than the NDFs, and hypomethylated if the average β-value for the TDFs was less than the NDFs. The allocation of DMCs into gene regions, CpG islands, and enhancer regions was determined from the Illumina GenomeStudio probe annotation^24^.

### Gene ontology enrichment analysis of differentially methylated CpGs

The DMCs were aligned to the TSS of the nearest transcript using the FDb. Infinium Methylation. hg 19 annotation package (v2.2.0) in R (v3.3.0). Transcripts with one or more DMCs located within 1,500 bases up- or down-stream of its TSS were selected. The transcripts were converted to Entrez Gene IDs, and gene ontology enrichment analysis on all, hypomethylated, and hypermethylated DMC was performed using the clusterProfiler R package (v2.4.3)^44^.

### Western immunoblot for alpha-smooth muscle actin (*α*-SMA)

Measurement of specific protein expression by western immunoblots was performed as previously described^10^. Briefly, adherent fibroblasts were washed with DPBS, detached by trypsin digestion and pelleted by centrifugation. Pelleted cells were lysed with 50 μl RIPA buffer (0.75 M NaCl, 5% NP40, 2.5% deoxycholic acid, 0.5% SDS, 0.25 M Tris, pH 8.0) for 15 minutes at 4 °C, and clarified by centrifugation at 8000 × *g* for 5 min. Protein was quantified by Bradford protein assay, and 20 μg was resolved using sodium dodecyl sulfate-polyacrylamide gel electrophoresis, transferred to Hybond-ECL membranes (GE Healthcare Life Sciences, Buckinghamshire, UK). Membranes were immunostained using mouse monoclonal anti-*α*-SMA (M085129-2, Dako) and mouse monoclonal anti-HSC-70 (sc-7298, Santa Cruz, USA). Immunoreactivity was detected using horseradish peroxidase-labelled secondary antibody, and visualised with SuperSignal West Pico Chemiluminescent Substrate (Thermo Scientific Pierce, Waltham, MA, USA) using a ChemiDoc-It Imager (UVP, Upland, CA, USA). The intensity of the *α*-SMA and the HSC-70 bands were determined using ImageJ (v1.47). The *α*-SMA expression was calculated as the ratio of the intensity of *α*-SMA divided by the intensity of HSC-70.

### Statistical analysis

Pairwise multidimensional scaling was conducted using the LIMMA R package (v3.18.5). The equality of the fibroblast group variances was compared using the median centred Levene test in the car R package (v2.1-2). The proportion of DMCs in gene regions, CpG islands, or enhancer regions and the proportion of hypomethylated and hypermethylated DMC in each of these regions was analysed with the Chi-squared test with Yates correction, using Prism 6.0 h for Macintosh (GraphPad Software, San Diego, CA, USA). A two-tailed p < 0.05 was considered statistically significant.

## Electronic supplementary material

Supplementary Table S1 and S2
