# Supplementary material for: Fibroblasts derived from oesophageal adenocarcinoma differ in DNA methylation profile from normal oesophageal fibroblasts
Source: Sci Rep. 2017 Jun 13;7:3368. doi: 10.1038/s41598-017-03501-6 (PMC5469830; doi:10.1038/s41598-017-03501-6)
Supplement: Supplementary file 1 — Supplementary Table S1 and S2 [file 41598_2017_3501_MOESM1_ESM.pdf]

Fibroblasts derived from oesophageal adenocarcinoma differ in DNA methylation profile from normal oesophageal fibroblasts

Authors: Eric Smith, Helen M. Palethorpe, Annette L. Hayden, Joanne P. Young, Timothy J. Underwood, Paul A. Drew

Supplementary Table S1

Patient Number	TDF	NDF	Gender	Age	Tumour Type	Tumour Differentiation	Pretreatment T Staging	Pretreatment N Staging	Pretreatment M Staging	Curative Treatment Modality	Radiology Response	Pathological T Staging	Pathological N Staging	Pathological M Staging	Positive Nodes	Total Nodes Counted	Proximal Resection Margin	Distal Resection Margin	Circumferential Resection Margin (CRM)	CRM (mm)	Vascular Invasion	Lymphatic Invasion	Perineural Invasion	Tumour Location
18		N.18	Male	63	AC	G2	T3	N1	M0	Neoadjuvant Chemotherapy + Surgery	Partial Response	T2	N0	M0	0	35	No	No	No	Clear	No	No	No	OGJ-S2
23		N.23	Male	65	AC	G3	T3	N1	M0	Neoadjuvant Chemotherapy + Surgery	Complete Response	T2	N0	M0	0	16	No	No	No	20	No	No	No	OGJ-S2
32		N.32	Female	68	SCC	G2	T3	N1	M0	Neoadjuvant Chemotherapy + Surgery	Partial Response	T2	N0	M0	0	17	No	No	No	1	No	No	No	Lower 1/3
38		N.38	Male	66	AC	G1	T3	N1	M0	Neoadjuvant Chemotherapy + Surgery	Partial Response	T2	N0	M0	0	21	No	No	No	13	Yes	Yes	No	OGJ-S1
50		N.50	Female	57	AC	G2	T3	N0	M0	Neoadjuvant Chemotherapy + Surgery	Partial Response	T2	N0	M0	0	17	No	No	No	8	No	No	No	Lower 1/3
55	T.55		Male	61	AC	G2	T3	N1	M0	Neoadjuvant Chemotherapy + Surgery	Partial Response	T3	N3	M0	13	38	No	No	Yes	<1	Yes	No	Yes	Lower 1/3
59	T.59		Male	71	AC	G3	T3	N1	M0	Neoadjuvant Chemotherapy + Surgery	Partial Response	T3	N3	M0	7	18	No	No	No	Clear	Yes	No	No	OGJ-S1
64	T.64		Male	70	AC	G3	T3	N0	M0	Neoadjuvant Chemotherapy + Surgery	Partial Response	T1	N0	M0	0	11	No	No	No	9.5	No	No	No	OGJ-S1
108	T.108	N.108	Male	63	AC	G3	T3	N1	M0	Neoadjuvant Chemotherapy + Surgery	Partial Response	T3	N0	M0	0	22	No	No	No	7	Yes	No	Yes	OGJ-S2
109	T.109		Female	79	AC	G3	T3	N1	M0	Surgery only		T3	N1	M0	2	34	No	No	No	1	No	No	No	Lower 1/3
181		N.181	Male	63	AC	G3	T2	N0	M0	Surgery only		T1	N0	M0	0	13	No	No	No	11	No	No	No	OGJ-S2
205	T.205	N.205	Male	64	AC	G1	T1	N0	M0	Surgery only		T IS/HGD	N0	M0	0	28	No	No	No		No	No	No	Lower 1/3
217	T.217	N.217	Male	58	AC	G2	T2	N0	M0	Surgery only		T3	N3	M0	15	27	No	No	Yes	1	No	No	No	Lower 1/3
251	T.251	N.251	Male	82	AC	G2	T3	N1	M0	Surgery only		T3	N0	M0	0	26	No	No	Yes	<1	No	No	Yes	OGJ-S2
255	T.255	N.255	Male	64	AC	G3	T3	N1	M0	Neoadjuvant Chemotherapy + Surgery	Partial Response	T3	N0	M0	0	45	No	No	Yes	0	No	No	Yes	OGJ-S2
528	T.528	N.528	Male	76	AC	G2	T2	N1	M0	Neoadjuvant Chemotherapy + Surgery		T2	N1	M0	2	36	No	No	No		NA	NA	NA	OGJ

Supplementary Table S2

**All DMC, Biological Processes**								
ID	Description	GeneRatio	BgRatio	pvalue	p.adjust	qvalue	geneID	Count
GO:0008150	biological_process	901/901	15230/17046	4.38E-46	2.61E-42	2.05E-42	AKT3/ABI1/CDH3/TANK/CD300LD/GNE/ZNF783/CDH9/TSPAN5/CDH12/CDH13/SUGP2/MBNL2/FARP1/KLRG1/RCAN2/KCNMB2/CDKN1C/SPEG/BCKDK/TCIRG1/MRVI1/TRDN/ABCA9/SPON2/C1D/COG5/ZBTB18/PITRM1/TACC2/MTHFS/PDPN/DMRT2/CELF1/CELF2/TBR1/SEPT9/GJB6/HCST/NPFFR2/ADCY3/PNRC1/TMED10/SLC27A2/LECT1/RER1/ESM1/ADAM29/HNRNPUL1/RPP14/HIBADH/CHGA/CHI3L1/ERLIN2/PSIP1/CHI3L2/PKP3/EGLN2/TP53TG1/PXMP4/ATXN2L/B4GALT7/PTH2/KIF12/ACOT7/PDAP1/EXOC3/CHRNA1/CHRNA2/ADPRHL1/CHRNA5/CARD16/GPRIN1/SORCS1/ZBED9/CIDEA/C1QTNF7/GBP4/ALPK2/PANX3/RBP7/GALNT15/AP3S1/CLCA1/FAT3/CLN5/ANKRD9/MRPL52/FRMD6/CCR1/SLC51B/C15orf27/ZG16B/SLC38A10/SEZ6/TNFAIP8L1/CNP/AADACL3/APOA1BP/NEU4/COL9A3/COL11A1/GALM/COMP/SCLT1/MAP3K8/ZFP42/ADM/IL31RA/EGFLAM/UBLCP1/HUS1B/OR2A14/CPD/CPM/CPS1/NDUFAF6/PXDNL/CRABP1/ZNF358/TRPM6/CRYBB3/MIB2/PARP4/MPP7/LDLRAD3/FAM101A/B3GLCT/MGAT5B/APCDD1/CSTA/KLC3/ZNF738/CTGF/ABCC13/SMYD1/SGOL1/PPM1L/LRRC34/SH3D19/CYB561/CYLD/MBOAT1/ADRB3/ESCO2/CYP11A1/ZNF782/FITM1/ADAL/TRPV3/ZNF709/ZNF781/CITED4/DDB1/WBP2NL/LONRF2/DDOST/RNF168/ZNF366/BHLHA15/COCH/NLRP6/DIO3/DLG2/DMBT1/DNAH6/DNAH8/DNMT3A/ABAT/DPH1/DRD4/DSG3/DTNA/ECE1/AGXT/EEF2/EFNA2/EGFR/EGR3/PATL2/EIF4G1/A2M/ELK4/ANKRD23/TMEM17/LIPH/EML1/UNC13D/DNAH12/SLC10A4/ENO2/ADCK5/EPHA1/EPHA3/EPHB4/ESR1/ALAS1/F11/FAH/FAT2/SPATA13/PRSS54/FCGR2A/RNF182/PHACTR1/SP8/FGA/FGF10/FHIT/XRN2/RASA3/PPM1E/VASH1/BTBD3/SBNO2/TRAK1/MSRB2/ACIN1/LIMCH1/FOXL1/FOXC2/TBC1D9B/FOXO1/EXPH5/AKR1B1/SPG20/NFASC/EPB41L3/GGA3/FLNB/DIP2A/FLOT2/MLC1/TBC1D1/RHOBTB2/NUP210/ATP11A/NEDD4L/SYNE1/PSD3/LARP1/PPP1R13B/PUM2/ARHGEF18/RYBP/MORC3/MAPK8IP2/TSSK2/VGLL2/MTOR/FUCA1/SLC37A4/GABBR1/RASGEF1C/RNF144B/ZNF549/ST6GALNAC3/DSCR9/GAK/SAMM50/DFNB31/ALS2CL/PNKD/SEC31B/TENM4/ACOT11/LTN1/RGS22/STEAP2/GAS2/FBXL21/FBXO2/LCE2B/SACS/GATM/GBGT1/GAPDHS/PLEK2/SLC17A5/ADGRF1/RPS6KC1/PABPC1/AKAP8L/GJA3/DNAJC2/FGF22/NPTN/GJB2/CLUL1/AMPD2/SDCBP2/PDE7B/DKK3/CYTH4/GLS2/VPS4A/AMPD3/GPR162/DHDH/BMP10/ZNF638/GNAS/ZNF311/CRACR2B/TMPRSS12/PIGW/IZUMO1/ZNF844/THEM5/GPR26/GPER1/EOGT/DOK7/FFAR2/GRB10/MRPS18B/FLVCR1/GRIK4/ZBTB44/DNAJC15/SCG3/GSTP1/GTF2B/BRF1/TMOD4/GUCY1A3/NME7/GPR132/PADI1/GZMA/ANXA2/HAS1/SERPIND1/SOX8/KCNIP2/NRG1/ANXA6/HK1/HLA-B/HLA-DOA/HLA-DPA1/ANXA13/HLA-E/HLA-F/HLX/HMGA1/NR4A1/ACACB/HPCA/APBA2/HOXB3/HOXC4/HOXC5/HOXC6/HOXD3/AGFG2/HRH1/HSD11B1/HSD17B2/ACADL/HSPA1L/HSP90AA1/HSP90AB1/HTR3A/HTR5A/DUPD1/ADAMTSL5/ANKRD45/TFAP2E/ID3/ZC3H12D/COL28A1/RSPO2/FMN1/CD300E/BARHL2/NME9/IGF1/IGF2/CYR61/GPR142/LCE1C/LCE1D/LCE2D/IL1R1/IL1RN/IL6/IL10RA/AQP2/IL11RA/IL12RB2/IL15RA/PRSS41/IL16/FOXK2/AQP5/INHBA/INPP5A/IRF1/AQP9/ISL1/ITGA7/ITGB2/ITGB7/ITIH3/ITIH4/IVL/JUP/CD82/USP50/HILS1/ATP9B/KCNH2/KCNJ8/KCNJ9/KCNMB1/KDR/ACAT1/KIF25/IPO5/KRT7/KRT15/AMIGO3/INSC/TOMM20L/HES5/SLC6A17/RESP18/CDHR4/AFF3/LAMA3/STMN1/OR2A5/LCK/LCP1/MUC21/LDLR/ARHGDIA/LGALS9/LHCGR/LLGL1/LMNA/LMO2/RAB19/LOX/SRRD/LPP/LTB/LTBP1/SMAD3/MC2R/MCC/ME1/ME2/MEF2D/MAP3K1/MEOX1/MEOX2/MFI2/MFNG/MGAT1/SCGB2A1/MITF/LHX8/ASGR1/MOCS1/MOV10/MPZ/PLEKHG7/MT1A/NUDT1/MYH4/MYL2/NUBP1/NDUFB4/DRG1/NEDD9/NEU1/ATP1A2/NFATC3/NFYB/NHLH2/NMBR/NOV/NPPC/NRAS/NTF3/OAS2/OPRL1/OR2C1/OR3A2/SLC22A18/P2RY6/PAFAH2/ATP5B/IL21R/ANO7/PALM/ARHGEF3/PARK2/SPOCK3/UTP11L/LEF1/DDX47/CEND1/PRR16/CHST15/ANGPT4/PDE4C/PCYOX1/PDE7A/C11orf73/SIRT6/PDE6B/ATP8A2/GALNT7/PGAM2/PI3/PIGC/PIK3CG/PITX2/PKHD1/PKM/PLA2G2A/PLAGL1/SPA17/LRP1B/PLEC/PRKAG3/PML/RIPPLY3/FXYD6/GPR84/IL20RB/SLCO1C1/PNLIP/RIPK4/TLR9/TREM1/CYTL1/POMC/SSH1/PON1/RIN2/MOV10L1/POU2AF1/ZDHHC13/APBB1IP/ROBO4/MXRA8/FBLIM1/BNC2/MED18/PALMD/CYP2W1/RPP25/LPCAT2/BANP/PPP1CB/HERC6/PPP1CC/PIWIL2/ELP3/ARHGEF10L/PRMT6/DNAJC17/GOLPH3L/ZNF532/PPP2R2B/FANCI/MOB1A/SLC47A1/SLC29A3/MIS18BP1/WDR33/TRPV6/SMPD3/SLC30A10/CNOT11/CHRNA9/SYBU/PEX26/LIMS2/FRMD4A/VAC14/CARKD/PARVA/PRKAR1B/TTC17/IFT122/ERMARD/MCTP2/LMBRD1/CSGALNACT1/PAG1/CISD1/PRKD1/WSB2/MYNN/BIN3/APOBR/MAPK3/MAP2K2/PCDHGC4/PCDHGB7/PCDHGB3/PCDHGA11/PRKRIR/PROC/MRAP/TRPV5/PRMT8/MASP1/HTRA1/SLAMF8/CDC42SE1/PSMB4/PAK6/ARNTL2/RGMA/PRDM11/TRPC7/LPAR5/PSMD7/SLURP1/ACTR3B/PTGFR/PLEKHG5/TENM2/GATAD2B/ERMN/KLHL8/RDH14/METTL14/MARK4/CCAR2/PTPRCAP/PTPRE/PXN/CREBZF/ABHD17C/	901

GO:0009987	cellular process	827/901	13765/17046	2.52E-21	7.52E-18	5.91E-18	AKT3/ABI1/CDH3/TANK/GNE/ZNF783/CDH9/TSPAN5/CDH12/CDH13/SUGP2/MBNL2/FARP1/KLRG1/RCAN2/KCNMB2/CDKN1C/SPEG/BCKDK/TCIRG1/MRVI1/TRDN/ABCA9/SPON2/C1D/ZBTB18/TACC2/MTHFS/PDPN/DMRT2/CELF1/CELF2/TBR1/SEPT9/GJB6/HCST/NPFFR2/ADCY3/PNRC1/TMED10/SLC27A2/LECT1/RER1/ESM1/HNRNPUL1/RPP14/HIBADH/CHGA/CHI3L1/ERLIN2/PSIP1/PKP3/EGLN2/TP53TG1/ATXN2L/B4GALT7/PTH2/KIF12/ACOT7/PDAP1/EXOC3/CHRNA1/CHRNA2/ADPRHL1/CHRNA5/CARD16/GPRIN1/SORCS1/ZBED9/CIDEA/C1QTNF7/ALPK2/PANX3/GALNT15/AP3S1/CLCA1/CLN5/ANKRD9/MRPL52/FRMD6/CCR1/SLC51B/C15orf27/SLC38A10/SEZ6/TNFAIP8L1/CNP/APOA1BP/NEU4/COL9A3/COL11A1/COMP/SCLT1/MAP3K8/ZFP42/ADM/IL31RA/EGFLAM/UBLCP1/HUS1B/OR2A14/CPS1/NDUFAF6/PXDNL/CRABP1/ZNF358/TRPM6/MIB2/PARP4/MPP7/FAM101A/B3GLCT/MGAT5B/APCDD1/CSTA/KLC3/ZNF738/CTGF/ABCC13/SMYD1/SGOL1/PPM1L/SH3D19/CYB561/CYLD/MBOAT1/ADRB3/ESCO2/CYP11A1/ZNF782/FITM1/ADAL/TRPV3/ZNF709/ZNF781/CITED4/DDB1/WBP2NL/DDOST/RNF168/ZNF366/BHLHA15/COCH/NLRP6/DIO3/DLG2/DMBT1/DNAH6/DNAH8/DNMT3A/ABAT/DPH1/DRD4/DSG3/DTNA/ECE1/AGXT/EEF2/EFNA2/EGFR/EGR3/PATL2/EIF4G1/A2M/ELK4/TMEM17/EML1/UNC13D/DNAH12/SLC10A4/ENO2/ADCK5/EPHA1/EPHA3/EPHB4/ESR1/ALAS1/FAH/FAT2/SPATA13/FCGR2A/RNF182/PHACTR1/SP8/FGA/FGF10/FHIT/XRN2/RASA3/PPM1E/VASH1/BTBD3/SBNO2/TRAK1/MSRB2/ACIN1/LIMCH1/FOXL1/FOXC2/FOXO1/EXPH5/AKR1B1/SPG20/NFASC/EPB41L3/FLNB/DIP2A/FLOT2/MLC1/TBC1D1/RHOBTB2/NUP210/ATP11A/NEDD4L/SYNE1/PSD3/LARP1/PPP1R13B/PUM2/ARHGEF18/RYBP/MORC3/MAPK8IP2/TSSK2/VGLL2/MTOR/SLC37A4/GABBR1/RASGEF1C/RNF144B/ZNF549/ST6GALNAC3/GAK/SAMM50/DFNB31/ALS2CL/PNKD/TENM4/ACOT11/LTN1/RGS22/STEAP2/GAS2/FBXL21/FBXO2/LCE2B/SACS/GATM/GBGT1/GAPDHS/PLEK2/SLC17A5/ADGRF1/RPS6KC1/PABPC1/GJA3/DNAJC2/FGF22/NPTN/GJB2/CLUL1/AMPD2/SDCBP2/PDE7B/DKK3/CYTH4/GLS2/VPS4A/AMPD3/GPR162/BMP10/ZNF638/GNAS/ZNF311/PIGW/IZUMO1/ZNF844/THEM5/GPR26/GPER1/EOGT/DOK7/FFAR2/GRB10/MRPS18B/FLVCR1/GRIK4/ZBTB44/DNAJC15/SCG3/GSTP1/GTF2B/BRF1/TMOD4/GUCY1A3/NME7/GPR132/PADI1/GZMA/ANXA2/HAS1/SERPIND1/SOX8/KCNIP2/NRG1/ANXA6/HK1/HLA-B/HLA-DOA/HLA-DPA1/ANXA13/HLA-E/HLA-F/HLX/HMGA1/NR4A1/ACACB/HPCA/APBA2/HOXB3/HOXC4/HOXC5/HOXC6/HOXD3/HRH1/ACADL/HSPA1L/HSP90AA1/HSP90AB1/HTR3A/HTR5A/DUPD1/TFAP2E/ID3/ZC3H12D/COL28A1/RSPO2/FMN1/BARHL2/NME9/IGF1/IGF2/CYR61/GPR142/LCE1C/LCE1D/LCE2D/IL1R1/IL1RN/IL6/IL10RA/AQP2/IL11RA/IL12RB2/IL15RA/IL16/FOXK2/AQP5/INHBA/INPP5A/IRF1/AQP9/ISL1/ITGA7/ITGB2/ITGB7/ITIH3/ITIH4/IVL/JUP/CD82/USP50/HILS1/ATP9B/KCNH2/KCNJ8/KCNJ9/KCNMB1/KDR/ACAT1/KIF25/IPO5/KRT7/KRT15/AMIGO3/INSC/HES5/SLC6A17/AFF3/LAMA3/STMN1/OR2A5/LCK/LCP1/MUC21/LDLR/ARHGDIA/LGALS9/LHCGR/LLGL1/LMNA/LMO2/RAB19/LOX/LTB/LTBP1/SMAD3/MC2R/MCC/ME1/ME2/MEF2D/MAP3K1/MEOX1/MEOX2/MFI2/MFNG/MGAT1/SCGB2A1/MITF/LHX8/ASGR1/MOCS1/MOV10/MPZ/PLEKHG7/MT1A/NUDT1/MYH4/MYL2/NUBP1/NDUFB4/DRG1/NEDD9/NEU1/ATP1A2/NFATC3/NFYB/NHLH2/NMBR/NOV/NPPC/NRAS/NTF3/OAS2/OPRL1/OR2C1/OR3A2/SLC22A18/P2RY6/PAFAH2/ATP5B/IL21R/ANO7/PALM/ARHGEF3/PARK2/SPOCK3/UTP11L/LEF1/DDX47/CEND1/PRR16/CHST15/ANGPT4/PDE4C/PCYOX1/PDE7A/C11orf73/SIRT6/PDE6B/ATP8A2/GALNT7/PGAM2/PI3/PIGC/PIK3CG/PITX2/PKHD1/PKM/PLA2G2A/PLAGL1/SPA17/PLEC/PRKAG3/PML/RIPPLY3/FXYD6/GPR84/IL20RB/SLCO1C1/PNLIP/RIPK4/TLR9/TREM1/CYTL1/POMC/SSH1/PON1/RIN2/MOV10L1/POU2AF1/ZDHHC13/APBB1IP/ROBO4/MXRA8/FBLIM1/BNC2/MED18/PALMD/CYP2W1/RPP25/LPCAT2/BANP/PPP1CB/HERC6/PPP1CC/PIWIL2/ELP3/ARHGEF10L/PRMT6/DNAJC17/GOLPH3L/ZNF532/PPP2R2B/FANCI/MOB1A/SLC47A1/SLC29A3/MIS18BP1/WDR33/TRPV6/SMPD3/SLC30A10/CNOT11/CHRNA9/SYBU/PEX26/LIMS2/FRMD4A/VAC14/PARVA/PRKAR1B/TTC17/IFT122/MCTP2/LMBRD1/CSGALNACT1/PAG1/CISD1/PRKD1/WSB2/MYNN/BIN3/APOBR/MAPK3/MAP2K2/PRKRIR/PROC/MRAP/TRPV5/PRMT8/MASP1/HTRA1/SLAMF8/CDC42SE1/PSMB4/PAK6/ARNTL2/RGMA/PRDM11/TRPC7/LPAR5/PSMD7/SLURP1/ACTR3B/PTGFR/PLEKHG5/TENM2/GATAD2B/ERMN/KLHL8/RDH14/METTL14/MARK4/CCAR2/PTPRE/PXN/CREBZF/FAM60A/ACTA2/RASGRF2/RFC2/TRIM27/RGR/RGS12/RIT2/EXOC4/RPA3/RPL8/RPL29/S100A4/S100A6/BGLAP/SCT/CCL11/CCL17/MRPS14/NPAS3/PARVG/NOD2/STRA6/SFRP2/CXCR5/MAP1LC3B2/ARHGAP9/TRA2B/GZF1/DNAI2/SGK1/MICAL1/CERK/TMEM237/VPS33A/BMP4/SLC4A1/ZNF649/SLC6A12/SLC8A1/SLC9A3/SLC20A2/BMPR1B/SLIT1/BRD9/ZSCAN18/BOK/SOX9/BPI/SRP68/STAT2/STK3/STK10/SUPT6H/BST2/VAMP2/TAF4B/TBP/TCEA1/TCEB2/ZEB1/ACTC1/TEAD3/TERF1/TGM2/TCHH/TIMP3/TLE3/TLR5/TRAPPC10/TNFAIP3/TNFRSF1A/TNX	827

GO:0044699	single-organism process	770/901	12449/17046	3.78E-20	7.51E-17	5.91E-17	AKT3/ABI1/CDH3/TANK/GNE/CDH9/TSPAN5/CDH12/CDH13/FARP1/KLRG1/RCAN2/KCNMB2/CDKN1C/SPEG/BCKDK/TCIRG1/MRVI1/TRDN/ABCA9/SPON2/C1D/COG5/ZBTB18/TACC2/MTHFS/PDPN/DMRT2/CELF1/CELF2/TBR1/SEPT9/GJB6/HCST/NPFFR2/ADCY3/TMED10/SLC27A2/LECT1/RER1/ESM1/ADAM29/HIBADH/CHGA/CHI3L1/ERLIN2/PKP3/EGLN2/B4GALT7/PTH2/KIF12/ACOT7/PDAP1/EXOC3/CHRNA1/CHRNA2/CHRNA5/CARD16/GPRIN1/SORCS1/CIDEA/PANX3/GALNT15/AP3S1/CLCA1/FAT3/CLN5/ANKRD9/MRPL52/FRMD6/CCR1/SLC51B/C15orf27/ZG16B/SLC38A10/SEZ6/TNFAIP8L1/CNP/APOA1BP/NEU4/COL9A3/COL11A1/GALM/COMP/SCLT1/MAP3K8/ZFP42/ADM/IL31RA/EGFLAM/HUS1B/OR2A14/CPS1/NDUFAF6/PXDNL/CRABP1/ZNF358/TRPM6/CRYBB3/MIB2/PARP4/MPP7/FAM101A/B3GLCT/MGAT5B/APCDD1/CSTA/KLC3/CTGF/ABCC13/SMYD1/SGOL1/PPM1L/SH3D19/CYB561/CYLD/MBOAT1/ADRB3/ESCO2/CYP11A1/FITM1/ADAL/TRPV3/DDB1/WBP2NL/DDOST/RNF168/ZNF366/BHLHA15/COCH/NLRP6/DIO3/DLG2/DMBT1/DNAH6/DNAH8/DNMT3A/ABAT/DPH1/DRD4/DSG3/DTNA/ECE1/AGXT/EEF2/EFNA2/EGFR/EGR3/EIF4G1/A2M/ELK4/TMEM17/LIPH/EML1/UNC13D/DNAH12/SLC10A4/ENO2/EPHA1/EPHA3/EPHB4/ESR1/ALAS1/F11/FAH/FAT2/SPATA13/FCGR2A/PHACTR1/SP8/FGA/FGF10/FHIT/XRN2/RASA3/PPM1E/VASH1/BTBD3/SBNO2/TRAK1/MSRB2/ACIN1/LIMCH1/FOXL1/FOXC2/FOXO1/EXPH5/AKR1B1/SPG20/NFASC/EPB41L3/FLNB/DIP2A/FLOT2/MLC1/RHOBTB2/NUP210/ATP11A/NEDD4L/SYNE1/PSD3/LARP1/PPP1R13B/PUM2/ARHGEF18/RYBP/MORC3/MAPK8IP2/TSSK2/VGLL2/MTOR/FUCA1/SLC37A4/GABBR1/RASGEF1C/RNF144B/ST6GALNAC3/GAK/SAMM50/DFNB31/ALS2CL/PNKD/TENM4/ACOT11/RGS22/STEAP2/GAS2/FBXO2/LCE2B/GATM/GBGT1/GAPDHS/PLEK2/SLC17A5/ADGRF1/RPS6KC1/PABPC1/GJA3/FGF22/NPTN/GJB2/CLUL1/AMPD2/SDCBP2/PDE7B/DKK3/CYTH4/GLS2/VPS4A/AMPD3/GPR162/DHDH/BMP10/GNAS/CRACR2B/PIGW/IZUMO1/THEM5/GPR26/GPER1/EOGT/FFAR2/GRB10/MRPS18B/FLVCR1/GRIK4/DNAJC15/SCG3/GSTP1/TMOD4/GUCY1A3/NME7/GPR132/PADI1/GZMA/ANXA2/HAS1/SERPIND1/SOX8/KCNIP2/NRG1/ANXA6/HK1/HLA-B/HLA-DOA/HLA-DPA1/ANXA13/HLA-E/HLA-F/HLX/HMGA1/NR4A1/ACACB/HPCA/APBA2/HOXB3/HOXC4/HOXC5/HOXC6/HOXD3/HRH1/HSD11B1/HSD17B2/ACADL/HSPA1L/HSP90AA1/HSP90AB1/HTR3A/HTR5A/TFAP2E/ID3/ZC3H12D/COL28A1/RSPO2/FMN1/BARHL2/NME9/IGF1/IGF2/CYR61/GPR142/LCE1C/LCE1D/LCE2D/IL1R1/IL1RN/IL6/IL10RA/AQP2/IL11RA/IL12RB2/IL15RA/IL16/FOXK2/AQP5/INHBA/INPP5A/IRF1/AQP9/ISL1/ITGA7/ITGB2/ITGB7/IVL/JUP/CD82/USP50/HILS1/ATP9B/KCNH2/KCNJ8/KCNJ9/KCNMB1/KDR/ACAT1/KIF25/IPO5/KRT15/AMIGO3/INSC/TOMM20L/HES5/SLC6A17/RESP18/AFF3/LAMA3/STMN1/OR2A5/LCK/LCP1/MUC21/LDLR/ARHGDIA/LGALS9/LHCGR/LLGL1/LMNA/LMO2/RAB19/LOX/LTB/LTBP1/SMAD3/MC2R/MCC/ME1/ME2/MEF2D/MAP3K1/MEOX1/MEOX2/MFI2/MFNG/MGAT1/SCGB2A1/MITF/LHX8/ASGR1/MOCS1/MOV10/MPZ/PLEKHG7/NUDT1/MYH4/MYL2/NUBP1/NDUFB4/DRG1/NEDD9/NEU1/ATP1A2/NFATC3/NFYB/NHLH2/NMBR/NOV/NPPC/NRAS/NTF3/OAS2/OPRL1/OR2C1/OR3A2/SLC22A18/P2RY6/PAFAH2/ATP5B/IL21R/ANO7/PALM/ARHGEF3/PARK2/SPOCK3/UTP11L/LEF1/DDX47/CEND1/PRR16/CHST15/ANGPT4/PDE4C/PCYOX1/PDE7A/C11orf73/SIRT6/PDE6B/ATP8A2/GALNT7/PGAM2/PIGC/PIK3CG/PITX2/PKHD1/PKM/PLA2G2A/PLAGL1/SPA17/PLEC/PRKAG3/PML/RIPPLY3/FXYD6/GPR84/IL20RB/SLCO1C1/PNLIP/RIPK4/TLR9/TREM1/CYTL1/POMC/SSH1/PON1/RIN2/MOV10L1/ZDHHC13/APBB1IP/ROBO4/MXRA8/FBLIM1/BNC2/PALMD/CYP2W1/LPCAT2/BANP/PPP1CB/HERC6/PPP1CC/PIWIL2/ELP3/ARHGEF10L/PRMT6/GOLPH3L/PPP2R2B/FANCI/MOB1A/SLC47A1/SLC29A3/MIS18BP1/WDR33/TRPV6/SMPD3/SLC30A10/CNOT11/CHRNA9/SYBU/PEX26/LIMS2/FRMD4A/VAC14/PARVA/PRKAR1B/TTC17/IFT122/ERMARD/MCTP2/LMBRD1/CSGALNACT1/PAG1/CISD1/PRKD1/WSB2/BIN3/APOBR/MAPK3/MAP2K2/PRKRIR/PROC/MRAP/TRPV5/PRMT8/MASP1/HTRA1/SLAMF8/CDC42SE1/PSMB4/PAK6/RGMA/TRPC7/LPAR5/PSMD7/SLURP1/ACTR3B/PTGFR/PLEKHG5/TENM2/ERMN/RDH14/METTL14/MARK4/CCAR2/PTPRE/PXN/FAM60A/ACTA2/RASGRF2/RFC2/TRIM27/RGR/RGS12/RIT2/EXOC4/RPA3/RPL8/RPL29/S100A4/S100A6/BGLAP/SCT/CCL11/CCL17/ABHD4/MRPS14/NPAS3/PARVG/NOD2/TINAGL1/STRA6/SFRP2/CXCR5/ARHGAP9/TRA2B/GZF1/DNAI2/SGK1/MICAL1/CERK/TMEM237/VPS33A/BMP4/SLC4A1/SLC6A12/SLC8A1/SLC9A3/SLC20A2/BMPR1B/SLIT1/BOK/SOX9/BPI/SRP68/STAT2/STK3/STK10/SUPT6H/BST2/VAMP2/TAF4B/TBP/TCEA1/ZEB1/ACTC1/TEAD3/TERF1/TGM2/TCHH/TIMP3/TLE3/TLR5/TRAPPC10/TNFAIP3/TNFRSF1A/TNXB/TRAF1/TRAF5/TRPC4/TRPC6/TRPM2/PHLDA2/TWIST1/CCR2/TNFRSF4/UCP1/UPP1/VARS/WNT10B/YWHAG/ZAP70/ZNF7/CA7/CACNA1E/PTP4A1/CACNB2/MOGS/PAX8/CXCR4/FZD5/RAB7A/CARD14/PPDPF/GDPD3/BCL2L14/LST1/CERS4/TMEM204/NLRX1/CSPP1/EPHX3/CALD1/FAM188A/G	770

GO:0044763	single-organism cellular process	715/901	11314/17046	8.64E-19	1.29E-15	1.01E-15	AKT3/ABI1/CDH3/TANK/GNE/CDH9/TSPAN5/CDH12/CDH13/FARP1/KLRG1/RCAN2/KCNMB2/CDKN1C/SPEG/BCKDK/TCIRG1/MRVI1/TRDN/ABCA9/SPON2/C1D/ZBTB18/TACC2/MTHFS/PDPN/CELF1/TBR1/SEPT9/GJB6/HCST/NPFFR2/ADCY3/TMED10/SLC27A2/LECT1/RER1/ESM1/HIBADH/CHGA/CHI3L1/ERLIN2/PKP3/EGLN2/B4GALT7/PTH2/KIF12/ACOT7/PDAP1/EXOC3/CHRNA1/CHRNA2/CHRNA5/CARD16/GPRIN1/SORCS1/CIDEA/PANX3/GALNT15/AP3S1/CLCA1/CLN5/ANKRD9/MRPL52/FRMD6/CCR1/SLC51B/C15orf27/SLC38A10/SEZ6/TNFAIP8L1/CNP/APOA1BP/NEU4/COL9A3/COL11A1/COMP/SCLT1/MAP3K8/ZFP42/ADM/IL31RA/EGFLAM/HUS1B/OR2A14/CPS1/NDUFAF6/PXDNL/CRABP1/TRPM6/MIB2/PARP4/MPP7/FAM101A/B3GLCT/MGAT5B/APCDD1/CSTA/KLC3/CTGF/ABCC13/SMYD1/SGOL1/PPM1L/SH3D19/CYB561/CYLD/MBOAT1/ADRB3/ESCO2/CYP11A1/FITM1/ADAL/TRPV3/DDB1/WBP2NL/DDOST/RNF168/ZNF366/BHLHA15/COCH/NLRP6/DLG2/DMBT1/DNAH6/DNAH8/DNMT3A/ABAT/DPH1/DRD4/DSG3/DTNA/AGXT/EEF2/EFNA2/EGFR/EGR3/EIF4G1/A2M/ELK4/TMEM17/EML1/UNC13D/DNAH12/SLC10A4/ENO2/EPHA1/EPHA3/EPHB4/ESR1/ALAS1/FAH/FAT2/SPATA13/FCGR2A/PHACTR1/FGA/FGF10/FHIT/XRN2/RASA3/PPM1E/VASH1/BTBD3/SBNO2/TRAK1/MSRB2/ACIN1/LIMCH1/FOXL1/FOXC2/FOXO1/EXPH5/AKR1B1/SPG20/NFASC/EPB41L3/FLNB/DIP2A/FLOT2/RHOBTB2/NUP210/ATP11A/NEDD4L/SYNE1/PSD3/LARP1/PPP1R13B/PUM2/ARHGEF18/RYBP/MORC3/MAPK8IP2/TSSK2/MTOR/SLC37A4/GABBR1/RASGEF1C/RNF144B/ST6GALNAC3/GAK/SAMM50/DFNB31/ALS2CL/PNKD/TENM4/ACOT11/RGS22/STEAP2/GAS2/FBXO2/LCE2B/GATM/GBGT1/GAPDHS/PLEK2/SLC17A5/ADGRF1/RPS6KC1/PABPC1/GJA3/FGF22/NPTN/GJB2/CLUL1/AMPD2/SDCBP2/PDE7B/DKK3/CYTH4/GLS2/VPS4A/AMPD3/GPR162/BMP10/GNAS/PIGW/IZUMO1/THEM5/GPR26/GPER1/EOGT/FFAR2/GRB10/MRPS18B/FLVCR1/GRIK4/DNAJC15/SCG3/GSTP1/TMOD4/GUCY1A3/NME7/GPR132/PADI1/GZMA/ANXA2/HAS1/SOX8/KCNIP2/NRG1/ANXA6/HK1/HLA-B/HLA-DOA/HLA-DPA1/ANXA13/HLA-E/HLA-F/HLX/HMGA1/NR4A1/ACACB/HPCA/APBA2/HOXB3/HOXD3/HRH1/ACADL/HSPA1L/HSP90AA1/HSP90AB1/HTR3A/HTR5A/ID3/ZC3H12D/COL28A1/RSPO2/FMN1/BARHL2/NME9/IGF1/IGF2/CYR61/GPR142/LCE1C/LCE1D/LCE2D/IL1R1/IL1RN/IL6/IL10RA/AQP2/IL11RA/IL12RB2/IL15RA/IL16/FOXK2/AQP5/INHBA/INPP5A/IRF1/AQP9/ISL1/ITGA7/ITGB2/ITGB7/IVL/JUP/CD82/USP50/HILS1/ATP9B/KCNH2/KCNJ8/KCNJ9/KCNMB1/KDR/ACAT1/KIF25/KRT15/AMIGO3/INSC/HES5/SLC6A17/LAMA3/STMN1/OR2A5/LCK/LCP1/MUC21/LDLR/ARHGDIA/LGALS9/LHCGR/LLGL1/LMNA/RAB19/LOX/LTB/LTBP1/SMAD3/MC2R/MCC/ME1/ME2/MEF2D/MAP3K1/MEOX1/MEOX2/MFI2/MFNG/MGAT1/SCGB2A1/MITF/LHX8/ASGR1/MOV10/MPZ/PLEKHG7/NUDT1/MYH4/MYL2/NUBP1/NDUFB4/NEDD9/NEU1/ATP1A2/NFATC3/NHLH2/NMBR/NOV/NPPC/NRAS/NTF3/OAS2/OPRL1/OR2C1/OR3A2/SLC22A18/P2RY6/PAFAH2/ATP5B/IL21R/ANO7/PALM/ARHGEF3/PARK2/SPOCK3/UTP11L/LEF1/DDX47/CEND1/PRR16/CHST15/ANGPT4/PDE4C/PCYOX1/PDE7A/SIRT6/PDE6B/ATP8A2/GALNT7/PGAM2/PIGC/PIK3CG/PITX2/PKHD1/PKM/PLA2G2A/PLAGL1/SPA17/PLEC/PRKAG3/PML/FXYD6/GPR84/IL20RB/SLCO1C1/PNLIP/TLR9/TREM1/CYTL1/POMC/SSH1/PON1/RIN2/MOV10L1/ZDHHC13/APBB1IP/ROBO4/MXRA8/FBLIM1/PALMD/CYP2W1/LPCAT2/BANP/PPP1CB/HERC6/PPP1CC/PIWIL2/ELP3/ARHGEF10L/PRMT6/GOLPH3L/PPP2R2B/FANCI/MOB1A/SLC47A1/SLC29A3/MIS18BP1/WDR33/TRPV6/SMPD3/SLC30A10/CNOT11/CHRNA9/SYBU/PEX26/LIMS2/FRMD4A/VAC14/PARVA/PRKAR1B/TTC17/IFT122/MCTP2/LMBRD1/CSGALNACT1/PAG1/CISD1/PRKD1/WSB2/BIN3/APOBR/MAPK3/MAP2K2/PRKRIR/PROC/MRAP/TRPV5/PRMT8/HTRA1/SLAMF8/CDC42SE1/PSMB4/PAK6/RGMA/TRPC7/LPAR5/PSMD7/SLURP1/ACTR3B/PTGFR/PLEKHG5/TENM2/ERMN/RDH14/MARK4/CCAR2/PTPRE/PXN/FAM60A/ACTA2/RASGRF2/RFC2/TRIM27/RGR/RGS12/RIT2/EXOC4/RPA3/RPL8/RPL29/S100A4/S100A6/BGLAP/SCT/CCL11/CCL17/MRPS14/PARVG/NOD2/STRA6/SFRP2/CXCR5/ARHGAP9/DNAI2/SGK1/MICAL1/CERK/TMEM237/VPS33A/BMP4/SLC4A1/SLC6A12/SLC8A1/SLC9A3/SLC20A2/BMPR1B/SLIT1/BOK/SOX9/BPI/SRP68/STAT2/STK3/STK10/SUPT6H/BST2/VAMP2/TAF4B/TBP/TCEA1/ZEB1/ACTC1/TEAD3/TERF1/TGM2/TCHH/TLE3/TLR5/TRAPPC10/TNFAIP3/TNFRSF1A/TNXB/TRAF1/TRAF5/TRPC4/TRPC6/TRPM2/PHLDA2/TWIST1/CCR2/TNFRSF4/UCP1/UPP1/VARS/WNT10B/YWHAG/ZAP70/CA7/CACNA1E/PTP4A1/CACNB2/MOGS/PAX8/CXCR4/FZD5/RAB7A/CARD14/PPDPF/BCL2L14/LST1/CERS4/TMEM204/NLRX1/CSPP1/EPHX3/CALD1/FAM188A/GPR157/ZC3H12A/RAB11FIP1/FAAP100/CPEB4/C6orf25/COL18A1/EEPD1/CLPTM1L/CALR/COL21A1/UNC93B1/QTRT1/SLIRP/CAPS/COLQ/CAST/CAPZB/SH3BGRL3/SCRT1/HIST1H3A/DYNLRB2/SLC25A18/SPATA16/ANTXR1/SLA2/MFSD7/CMAHP/BFSP2/ATP13A4/NR0B2/MON1A/CASQ1/HOPX/PARD6G/PARD6B/TTBK1/IL1F10/TRIM63/KDM2B/LOXL3/MGARP/CBX2/RAE1/SLC43A1/IFITM1/GAS7/SCIN/CDK10/KMO/R	715

GO:0065007	biological regulation	666/901	10343/17046	6.62E-18	7.89E-15	6.21E-15	AKT3/ABI1/CDH3/TANK/ZNF783/TSPAN5/CDH13/MBNL2/FARP1/KLRG1/RCAN2/KCNMB2/CDKN1C/SPEG/TCIRG1/MRVI1/TRDN/SPON2/C1D/ZBTB18/PITRM1/TACC2/PDPN/DMRT2/CELF1/CELF2/TBR1/GJB6/HCST/NPFFR2/ADCY3/PNRC1/TMED10/LECT1/RER1/ESM1/HNRNPUL1/CHGA/CHI3L1/ERLIN2/PSIP1/EGLN2/ATXN2L/B4GALT7/PTH2/PDAP1/CHRNA1/CHRNA2/CHRNA5/CARD16/SORCS1/ZBED9/CIDEA/AP3S1/CLN5/ANKRD9/FRMD6/CCR1/SLC51B/C15orf27/ZG16B/SEZ6/TNFAIP8L1/CNP/COMP/MAP3K8/ZFP42/ADM/IL31RA/EGFLAM/HUS1B/OR2A14/CPS1/CRABP1/ZNF358/MIB2/MPP7/LDLRAD3/FAM101A/APCDD1/CSTA/ZNF738/CTGF/SMYD1/SGOL1/PPM1L/SH3D19/CYLD/ADRB3/ESCO2/CYP11A1/ZNF782/FITM1/TRPV3/ZNF709/ZNF781/CITED4/DDB1/RNF168/ZNF366/BHLHA15/COCH/NLRP6/DIO3/DLG2/DMBT1/DNMT3A/ABAT/DRD4/DTNA/ECE1/AGXT/EEF2/EFNA2/EGFR/EGR3/PATL2/EIF4G1/A2M/ELK4/TMEM17/UNC13D/EPHA1/EPHA3/EPHB4/ESR1/F11/SPATA13/FCGR2A/PHACTR1/SP8/FGA/FGF10/FHIT/XRN2/RASA3/PPM1E/VASH1/SBNO2/TRAK1/MSRB2/ACIN1/FOXL1/FOXC2/TBC1D9B/FOXO1/EXPH5/AKR1B1/SPG20/EPB41L3/GGA3/FLNB/DIP2A/FLOT2/MLC1/TBC1D1/RHOBTB2/NUP210/ATP11A/NEDD4L/SYNE1/PSD3/LARP1/PPP1R13B/PUM2/ARHGEF18/RYBP/MORC3/MAPK8IP2/TSSK2/VGLL2/MTOR/SLC37A4/GABBR1/RASGEF1C/RNF144B/ZNF549/DFNB31/ALS2CL/PNKD/TENM4/ACOT11/RGS22/STEAP2/GAS2/FBXL21/FBXO2/SACS/GAPDHS/PLEK2/ADGRF1/RPS6KC1/PABPC1/DNAJC2/FGF22/NPTN/AMPD2/SDCBP2/PDE7B/DKK3/CYTH4/GLS2/VPS4A/AMPD3/GPR162/BMP10/ZNF638/GNAS/ZNF311/CRACR2B/ZNF844/GPR26/GPER1/DOK7/FFAR2/GRB10/FLVCR1/GRIK4/ZBTB44/DNAJC15/SCG3/GSTP1/GTF2B/BRF1/TMOD4/GUCY1A3/NME7/GPR132/GZMA/ANXA2/HAS1/SERPIND1/SOX8/KCNIP2/NRG1/ANXA6/HK1/HLA-B/HLA-DOA/HLA-DPA1/ANXA13/HLA-E/HLA-F/HLX/HMGA1/NR4A1/ACACB/HPCA/APBA2/HOXB3/HOXC4/HOXC5/HOXC6/HOXD3/AGFG2/HRH1/ACADL/HSPA1L/HSP90AA1/HSP90AB1/HTR3A/HTR5A/TFAP2E/ID3/ZC3H12D/COL28A1/RSPO2/FMN1/BARHL2/NME9/IGF1/IGF2/CYR61/GPR142/IL1R1/IL1RN/IL6/IL10RA/AQP2/IL11RA/IL12RB2/IL15RA/IL16/FOXK2/AQP5/INHBA/IRF1/AQP9/ISL1/ITGA7/ITGB2/ITGB7/ITIH3/ITIH4/JUP/CD82/USP50/HILS1/ATP9B/KCNH2/KCNJ8/KCNJ9/KCNMB1/KDR/KIF25/IPO5/AMIGO3/HES5/AFF3/LAMA3/STMN1/OR2A5/LCK/LCP1/LDLR/ARHGDIA/LGALS9/LHCGR/LLGL1/LMNA/LMO2/RAB19/LTB/LTBP1/SMAD3/MC2R/MCC/ME1/ME2/MEF2D/MAP3K1/MEOX1/MEOX2/MFI2/MFNG/SCGB2A1/MITF/LHX8/MOV10/MPZ/PLEKHG7/MT1A/MYL2/NUBP1/NEDD9/NEU1/ATP1A2/NFATC3/NFYB/NHLH2/NMBR/NOV/NPPC/NRAS/NTF3/OAS2/OPRL1/OR2C1/OR3A2/P2RY6/PAFAH2/ATP5B/IL21R/ANO7/PALM/ARHGEF3/PARK2/SPOCK3/UTP11L/LEF1/DDX47/CEND1/PRR16/ANGPT4/PDE4C/PDE7A/C11orf73/SIRT6/PDE6B/ATP8A2/PGAM2/PI3/PIK3CG/PITX2/PKHD1/PLA2G2A/PLAGL1/PRKAG3/PML/RIPPLY3/FXYD6/GPR84/IL20RB/PNLIP/RIPK4/TLR9/TREM1/CYTL1/POMC/SSH1/PON1/RIN2/POU2AF1/ZDHHC13/APBB1IP/ROBO4/FBLIM1/BNC2/MED18/PALMD/BANP/PPP1CB/PPP1CC/PIWIL2/ELP3/ARHGEF10L/PRMT6/DNAJC17/GOLPH3L/ZNF532/PPP2R2B/FANCI/MOB1A/TRPV6/SMPD3/SLC30A10/CNOT11/CHRNA9/SYBU/LIMS2/VAC14/PARVA/PRKAR1B/IFT122/MCTP2/LMBRD1/PAG1/CISD1/PRKD1/WSB2/MYNN/BIN3/MAPK3/MAP2K2/PRKRIR/PROC/MRAP/PRMT8/MASP1/HTRA1/SLAMF8/CDC42SE1/PSMB4/PAK6/ARNTL2/RGMA/PRDM11/TRPC7/LPAR5/PSMD7/SLURP1/ACTR3B/PTGFR/PLEKHG5/TENM2/GATAD2B/ERMN/RDH14/METTL14/MARK4/CCAR2/PTPRE/PXN/CREBZF/FAM60A/ACTA2/RASGRF2/RFC2/TRIM27/RGR/RGS12/RIT2/RPA3/S100A4/S100A6/BGLAP/SCT/CCL11/CCL17/ABHD4/NPAS3/NOD2/STRA6/SFRP2/CXCR5/ARHGAP9/TRA2B/GZF1/SGK1/MICAL1/TMEM237/VPS33A/BMP4/SLC4A1/ZNF649/SLC6A12/SLC8A1/SLC9A3/BMPR1B/SLIT1/BRD9/ZSCAN18/BOK/SOX9/BPI/STAT2/STK3/STK10/SUPT6H/BST2/VAMP2/TAF4B/TBP/TCEA1/TCEB2/ZEB1/ACTC1/TEAD3/TERF1/TGM2/TIMP3/TLE3/TLR5/TNFAIP3/TNFRSF1A/TNXB/TRAF1/TRAF5/TRPC4/TRPC6/PHLDA2/TWIST1/CCR2/TNFRSF4/UCP1/VARS/WNT10B/YWHAG/ZAP70/ZNF7/CA7/ZNF124/ZNF177/CACNA1E/PTP4A1/CACNB2/PAX8/CXCR4/FZD5/RAB7A/CARD14/BCL2L14/LST1/TMEM204/NLRX1/ZNF665/CSPP1/ZC3H14/GPR157/ZNF606/ZC3H12A/RAB11FIP1/CPEB4/C6orf25/COL18A1/ZNF436/CALR/UNC93B1/SLIRP/CAPS/COLQ/CAST/CAPZB/SH3BGRL3/SCRT1/HIST1H3A/ANTXR1/SLA2/CMAHP/ATP13A4/ZNF397/NR0B2/CASQ1/HOPX/PARD6B/TTBK1/IL1F10/SPINK7/TRIM63/KDM2B/LOXL3/MGARP/CBX2/RAE1/IFITM1/GAS7/SCIN/CDK10/RUNX1/TP63/RUNX3/SERPINA6/IRS2/ACTN1/CRADD/FADD/TNFRSF11A/ALDH1A2/SPHK1/BUD31/CCNA1/SKAP2/LIMD1/TSPAN18/CCRL2/ERI1/PRC1/STARD13/PIAS2/ZFAND2A/MAP3K6/SYT7/LDB2/ESAM/SLC16A3/CBFA2T2/RSAD2/SMDT1/AURKB/DAPL1/CD8A/REEP6/TRIP10/ADIPOQ/ARHGAP29/LY86/RAB3D/H2AFY/SMAD5-	666

GO:0050789	regulation of biological process	636/901	9837/17046	1.71E-16	1.70E-13	1.33E-13	AKT3/ABI1/CDH3/TANK/ZNF783/TSPAN5/CDH13/MBNL2/FARP1/KLRG1/RCAN2/KCNMB2/CDKN1C/SPEG/TCIRG1/MRVI1/TRDN/SPON2/C1D/ZBTB18/PITRM1/TACC2/PDPN/DMRT2/CELF1/CELF2/TBR1/GJB6/HCST/NPFFR2/ADCY3/PNRC1/TMED10/LECT1/RER1/ESM1/HNRNPUL1/CHGA/CHI3L1/ERLIN2/PSIP1/EGLN2/ATXN2L/B4GALT7/PTH2/PDAP1/CHRNA1/CHRNA2/CHRNA5/CARD16/SORCS1/ZBED9/CIDEA/AP3S1/ANKRD9/FRMD6/CCR1/SLC51B/SEZ6/TNFAIP8L1/COMP/MAP3K8/ZFP42/ADM/IL31RA/EGFLAM/HUS1B/OR2A14/CPS1/CRABP1/ZNF358/MIB2/MPP7/LDLRAD3/FAM101A/APCDD1/CSTA/ZNF738/CTGF/SMYD1/SGOL1/PPM1L/SH3D19/CYLD/ADRB3/ESCO2/ZNF782/FITM1/TRPV3/ZNF709/ZNF781/CITED4/DDB1/RNF168/ZNF366/BHLHA15/COCH/NLRP6/DIO3/DLG2/DMBT1/DNMT3A/ABAT/DRD4/DTNA/ECE1/AGXT/EEF2/EFNA2/EGFR/EGR3/PATL2/EIF4G1/A2M/ELK4/TMEM17/UNC13D/EPHA1/EPHA3/EPHB4/ESR1/F11/SPATA13/FCGR2A/PHACTR1/SP8/FGA/FGF10/FHIT/XRN2/RASA3/PPM1E/VASH1/SBNO2/TRAK1/MSRB2/ACIN1/FOXL1/FOXC2/TBC1D9B/FOXO1/EXPH5/AKR1B1/SPG20/EPB41L3/GGA3/FLNB/DIP2A/FLOT2/MLC1/TBC1D1/RHOBTB2/NUP210/NEDD4L/PSD3/LARP1/PPP1R13B/PUM2/ARHGEF18/RYBP/MORC3/MAPK8IP2/TSSK2/VGLL2/MTOR/SLC37A4/GABBR1/RASGEF1C/RNF144B/ZNF549/ALS2CL/PNKD/TENM4/ACOT11/RGS22/GAS2/FBXL21/FBXO2/SACS/GAPDHS/PLEK2/ADGRF1/RPS6KC1/PABPC1/DNAJC2/FGF22/NPTN/SDCBP2/PDE7B/DKK3/CYTH4/GLS2/VPS4A/GPR162/BMP10/ZNF638/GNAS/ZNF311/CRACR2B/ZNF844/GPR26/GPER1/DOK7/FFAR2/GRB10/FLVCR1/GRIK4/ZBTB44/DNAJC15/GSTP1/GTF2B/BRF1/TMOD4/GUCY1A3/NME7/GPR132/GZMA/ANXA2/HAS1/SERPIND1/SOX8/KCNIP2/NRG1/ANXA6/HK1/HLA-B/HLA-DOA/HLA-DPA1/ANXA13/HLA-E/HLA-F/HLX/HMGA1/NR4A1/ACACB/HPCA/APBA2/HOXB3/HOXC4/HOXC5/HOXC6/HOXD3/AGFG2/HRH1/ACADL/HSPA1L/HSP90AA1/HSP90AB1/HTR3A/HTR5A/TFAP2E/ID3/ZC3H12D/COL28A1/RSPO2/FMN1/BARHL2/NME9/IGF1/IGF2/CYR61/GPR142/IL1R1/IL1RN/IL6/IL10RA/IL11RA/IL12RB2/IL15RA/IL16/FOXK2/INHBA/IRF1/ISL1/ITGA7/ITGB2/ITGB7/ITIH3/ITIH4/JUP/CD82/USP50/HILS1/KCNH2/KCNJ8/KCNJ9/KDR/KIF25/IPO5/AMIGO3/HES5/AFF3/LAMA3/STMN1/OR2A5/LCK/LCP1/LDLR/ARHGDIA/LGALS9/LHCGR/LLGL1/LMNA/LMO2/RAB19/LTB/LTBP1/SMAD3/MC2R/MCC/ME1/ME2/MEF2D/MAP3K1/MEOX1/MEOX2/MFI2/MFNG/SCGB2A1/MITF/LHX8/MOV10/MPZ/PLEKHG7/MT1A/MYL2/NUBP1/NEDD9/NEU1/ATP1A2/NFATC3/NFYB/NHLH2/NMBR/NOV/NPPC/NRAS/NTF3/OAS2/OPRL1/OR2C1/OR3A2/P2RY6/PAFAH2/ATP5B/IL21R/PALM/ARHGEF3/PARK2/SPOCK3/UTP11L/LEF1/DDX47/CEND1/PRR16/ANGPT4/PDE4C/PDE7A/C11orf73/SIRT6/PDE6B/ATP8A2/PGAM2/PI3/PIK3CG/PITX2/PKHD1/PLA2G2A/PLAGL1/PRKAG3/PML/RIPPLY3/FXYD6/GPR84/IL20RB/PNLIP/RIPK4/TLR9/TREM1/CYTL1/POMC/SSH1/PON1/RIN2/POU2AF1/ZDHHC13/APBB1IP/ROBO4/FBLIM1/BNC2/MED18/PALMD/BANP/PPP1CB/PPP1CC/PIWIL2/ELP3/ARHGEF10L/PRMT6/DNAJC17/GOLPH3L/ZNF532/PPP2R2B/FANCI/MOB1A/TRPV6/SMPD3/SLC30A10/CNOT11/CHRNA9/SYBU/LIMS2/VAC14/PARVA/PRKAR1B/IFT122/MCTP2/LMBRD1/PAG1/CISD1/PRKD1/WSB2/MYNN/BIN3/MAPK3/MAP2K2/PRKRIR/PROC/MRAP/PRMT8/MASP1/HTRA1/SLAMF8/CDC42SE1/PSMB4/PAK6/ARNTL2/RGMA/PRDM11/LPAR5/PSMD7/SLURP1/ACTR3B/PTGFR/PLEKHG5/TENM2/GATAD2B/ERMN/METTL14/MARK4/CCAR2/PTPRE/PXN/CREBZF/FAM60A/ACTA2/RASGRF2/TRIM27/RGR/RGS12/RIT2/RPA3/S100A4/S100A6/BGLAP/SCT/CCL11/CCL17/NPAS3/NOD2/STRA6/SFRP2/CXCR5/ARHGAP9/TRA2B/GZF1/SGK1/MICAL1/TMEM237/VPS33A/BMP4/ZNF649/SLC8A1/SLC9A3/BMPR1B/SLIT1/BRD9/ZSCAN18/BOK/SOX9/BPI/STAT2/STK3/STK10/SUPT6H/BST2/VAMP2/TAF4B/TBP/TCEA1/TCEB2/ZEB1/ACTC1/TEAD3/TERF1/TGM2/TIMP3/TLE3/TLR5/TNFAIP3/TNFRSF1A/TNXB/TRAF1/TRAF5/TRPC6/PHLDA2/TWIST1/CCR2/TNFRSF4/UCP1/VARS/WNT10B/YWHAG/ZAP70/ZNF7/CA7/ZNF124/ZNF177/CACNA1E/PTP4A1/CACNB2/PAX8/CXCR4/FZD5/RAB7A/CARD14/BCL2L14/LST1/TMEM204/NLRX1/ZNF665/CSPP1/ZC3H14/GPR157/ZNF606/ZC3H12A/RAB11FIP1/CPEB4/C6orf25/COL18A1/ZNF436/CALR/UNC93B1/SLIRP/CAPS/COLQ/CAST/CAPZB/SH3BGRL3/SCRT1/HIST1H3A/ANTXR1/SLA2/CMAHP/ZNF397/NR0B2/CASQ1/HOPX/PARD6B/TTBK1/IL1F10/SPINK7/TRIM63/KDM2B/LOXL3/MGARP/CBX2/RAE1/IFITM1/GAS7/SCIN/CDK10/RUNX1/TP63/RUNX3/SERPINA6/IRS2/ACTN1/CRADD/FADD/TNFRSF11A/ALDH1A2/SPHK1/BUD31/CCNA1/SKAP2/LIMD1/TSPAN18/CCRL2/ERI1/PRC1/STARD13/PIAS2/ZFAND2A/MAP3K6/SYT7/LDB2/CBFA2T2/RSAD2/AURKB/DAPL1/CD8A/REEP6/TRIP10/ADIPOQ/ARHGAP29/LY86/RAB3D/H2AFY/SMAD5-AS1/RAB36/ARHGEF10/MICAL2/N4BP1/VGLL4/NUP93/RAPGEF2/ULK2/USP6NL/CD79A/ZBTB39/TELO2/RABGAP1L/IQSEC1/FGF19/NR1H4	636

GO:0050794	regulation of cellular process	610/901	9347/17046	4.20E-16	3.58E-13	2.81E-13	AKT3/ABI1/CDH3/TANK/ZNF783/TSPAN5/CDH13/MBNL2/FARP1/KLRG1/RCAN2/CDKN1C/SPEG/TCIRG1/MRVI1/TRDN/C1D/ZBTB18/TACC2/PDPN/DMRT2/CELF1/TBR1/GJB6/HCST/NPFFR2/ADCY3/PNRC1/TMED10/LECT1/RER1/ESM1/HNRNPUL1/CHGA/CHI3L1/ERLIN2/PSIP1/EGLN2/ATXN2L/B4GALT7/PTH2/PDAP1/CHRNA1/CHRNA2/CHRNA5/CARD16/SORCS1/ZBED9/CIDEA/AP3S1/ANKRD9/FRMD6/CCR1/SLC51B/SEZ6/TNFAIP8L1/COMP/MAP3K8/ZFP42/ADM/IL31RA/EGFLAM/HUS1B/OR2A14/CRABP1/ZNF358/MIB2/MPP7/FAM101A/APCDD1/CSTA/ZNF738/CTGF/SMYD1/SGOL1/PPM1L/SH3D19/CYLD/ADRB3/ESCO2/ZNF782/TRPV3/ZNF709/ZNF781/CITED4/DDB1/RNF168/ZNF366/BHLHA15/COCH/NLRP6/DLG2/DMBT1/DNMT3A/ABAT/DRD4/DTNA/ECE1/AGXT/EEF2/EFNA2/EGFR/EGR3/PATL2/EIF4G1/A2M/ELK4/TMEM17/UNC13D/EPHA1/EPHA3/EPHB4/ESR1/SPATA13/FCGR2A/SP8/FGA/FGF10/FHIT/XRN2/RASA3/PPM1E/VASH1/SBNO2/TRAK1/MSRB2/ACIN1/FOXL1/FOXC2/FOXO1/EXPH5/AKR1B1/SPG20/EPB41L3/FLNB/DIP2A/FLOT2/MLC1/RHOBTB2/NUP210/NEDD4L/PSD3/LARP1/PPP1R13B/PUM2/ARHGEF18/RYBP/MORC3/MAPK8IP2/TSSK2/VGLL2/MTOR/GABBR1/RASGEF1C/RNF144B/ZNF549/ALS2CL/PNKD/TENM4/ACOT11/RGS22/GAS2/FBXO2/SACS/GAPDHS/PLEK2/ADGRF1/RPS6KC1/PABPC1/DNAJC2/FGF22/NPTN/SDCBP2/PDE7B/DKK3/CYTH4/GLS2/VPS4A/GPR162/BMP10/ZNF638/GNAS/ZNF311/ZNF844/GPR26/GPER1/DOK7/FFAR2/GRB10/GRIK4/ZBTB44/DNAJC15/GSTP1/GTF2B/BRF1/TMOD4/GUCY1A3/NME7/GPR132/GZMA/ANXA2/HAS1/SERPIND1/SOX8/KCNIP2/NRG1/ANXA6/HK1/HLA-B/HLA-DOA/HLA-DPA1/ANXA13/HLA-E/HLA-F/HLX/HMGA1/NR4A1/ACACB/HPCA/HOXB3/HOXC4/HOXC5/HOXC6/HOXD3/HRH1/ACADL/HSPA1L/HSP90AA1/HSP90AB1/HTR3A/HTR5A/TFAP2E/ID3/ZC3H12D/COL28A1/RSPO2/FMN1/BARHL2/NME9/IGF1/IGF2/CYR61/GPR142/IL1R1/IL1RN/IL6/IL10RA/IL11RA/IL12RB2/IL15RA/IL16/FOXK2/INHBA/IRF1/ISL1/ITGA7/ITGB2/ITGB7/ITIH3/ITIH4/JUP/CD82/USP50/HILS1/KCNH2/KCNJ8/KCNJ9/KDR/KIF25/IPO5/AMIGO3/HES5/AFF3/LAMA3/STMN1/OR2A5/LCK/LCP1/LDLR/ARHGDIA/LGALS9/LHCGR/LLGL1/LMNA/LMO2/RAB19/LTB/LTBP1/SMAD3/MC2R/MCC/ME1/ME2/MEF2D/MAP3K1/MEOX1/MEOX2/MFI2/MFNG/SCGB2A1/MITF/LHX8/MOV10/MPZ/PLEKHG7/MYL2/NUBP1/NEDD9/NEU1/ATP1A2/NFATC3/NFYB/NHLH2/NMBR/NOV/NPPC/NRAS/NTF3/OAS2/OPRL1/OR2C1/OR3A2/P2RY6/PAFAH2/ATP5B/IL21R/PALM/ARHGEF3/PARK2/SPOCK3/UTP11L/LEF1/DDX47/CEND1/PRR16/ANGPT4/PDE4C/PDE7A/C11orf73/SIRT6/PDE6B/ATP8A2/PGAM2/PI3/PIK3CG/PITX2/PKHD1/PLA2G2A/PLAGL1/PRKAG3/PML/RIPPLY3/FXYD6/GPR84/IL20RB/PNLIP/RIPK4/TLR9/TREM1/CYTL1/POMC/SSH1/RIN2/POU2AF1/ZDHHC13/APBB1IP/ROBO4/FBLIM1/BNC2/MED18/PALMD/BANP/PPP1CB/PPP1CC/PIWIL2/ELP3/ARHGEF10L/PRMT6/DNAJC17/GOLPH3L/ZNF532/PPP2R2B/FANCI/MOB1A/TRPV6/SMPD3/SLC30A10/CNOT11/CHRNA9/SYBU/LIMS2/VAC14/PARVA/PRKAR1B/IFT122/MCTP2/LMBRD1/PAG1/CISD1/PRKD1/WSB2/MYNN/BIN3/MAPK3/MAP2K2/PRKRIR/PROC/MRAP/PRMT8/MASP1/HTRA1/SLAMF8/CDC42SE1/PSMB4/PAK6/ARNTL2/RGMA/PRDM11/LPAR5/PSMD7/SLURP1/ACTR3B/PTGFR/PLEKHG5/TENM2/GATAD2B/ERMN/METTL14/MARK4/CCAR2/PTPRE/PXN/CREBZF/FAM60A/RASGRF2/TRIM27/RGR/RGS12/RIT2/RPA3/S100A4/S100A6/BGLAP/SCT/CCL11/CCL17/NPAS3/NOD2/STRA6/SFRP2/CXCR5/ARHGAP9/TRA2B/GZF1/SGK1/MICAL1/TMEM237/BMP4/ZNF649/SLC8A1/BMPR1B/SLIT1/BRD9/ZSCAN18/BOK/SOX9/BPI/STAT2/STK3/STK10/SUPT6H/BST2/VAMP2/TAF4B/TBP/TCEA1/TCEB2/ZEB1/ACTC1/TEAD3/TERF1/TGM2/TIMP3/TLE3/TLR5/TNFAIP3/TNFRSF1A/TNXB/TRAF1/TRAF5/TRPC6/PHLDA2/TWIST1/CCR2/TNFRSF4/UCP1/VARS/WNT10B/YWHAG/ZAP70/ZNF7/CA7/ZNF124/ZNF177/CACNA1E/PTP4A1/CACNB2/PAX8/CXCR4/FZD5/RAB7A/CARD14/BCL2L14/LST1/TMEM204/NLRX1/ZNF665/CSPP1/ZC3H14/GPR157/ZNF606/ZC3H12A/RAB11FIP1/CPEB4/C6orf25/COL18A1/ZNF436/CALR/UNC93B1/SLIRP/CAPS/COLQ/CAST/CAPZB/SH3BGRL3/SCRT1/HIST1H3A/ANTXR1/SLA2/CMAHP/ZNF397/NR0B2/CASQ1/HOPX/PARD6B/TTBK1/IL1F10/SPINK7/TRIM63/KDM2B/LOXL3/MGARP/CBX2/RAE1/IFITM1/GAS7/SCIN/CDK10/RUNX1/TP63/RUNX3/SERPINA6/IRS2/ACTN1/CRADD/FADD/TNFRSF11A/ALDH1A2/SPHK1/BUD31/CCNA1/SKAP2/LIMD1/TSPAN18/CCRL2/PRC1/STARD13/PIAS2/ZFAND2A/MAP3K6/SYT7/LDB2/CBFA2T2/RSAD2/AURKB/DAPL1/CD8A/REEP6/TRIP10/ADIPOQ/ARHGAP29/LY86/RAB3D/H2AFY/SMAD5-AS1/RAB36/ARHGEF10/MICAL2/N4BP1/VGLL4/NUP93/RAPGEF2/ULK2/USP6NL/CD79A/ZBTB39/TELO2/IQSEC1/FGF19/NR1H4	610
GO:0044767	single-organism developmental process	372/901	5251/17046	5.16E-12	3.84E-09	3.02E-09	ABI1/CDH3/CDH13/FARP1/CDKN1C/SPEG/SPON2/ZBTB18/TACC2/PDPN/DMRT2/CELF1/TBR1/GJB6/TMED10/LECT1/ESM1/CHI3L1/CHRNA1/GPRIN1/FAT3/CLN5/FRMD6/CCR1/SLC38A10/SEZ6/CNP/COL9A3/COL11A1/COMP/SCLT1/ZFP42/ADM/IL31RA/CPS1/CRABP1/ZNF358/FAM101A/APCDD1/CSTA/CTGF/SMYD1/SH3D19/CYLD/ADRB3/ESCO2/CYP11A1/RNF168/BHLHA15/COCH/DIO3/DLG2/DMBT1/DNMT3A/DRD4/ECE1/EEF2/EFNA2/EGFR/EGR3/EIF4G1/A2M/ELK4/TMEM17/EML1/UNC13D/EPHA1/EPHA3/EPHB4/ESR1/SP8/FGA/FGF10/RASA3/VASH1/BTBD3/SBNO2/ACIN1/FOXL1/FOXC2/FOXO1/EXPH5/AKR1B1/SPG20/NFASC/EPB41L3/FLNB/DIP2A/FLOT2/NEDD4L/SYNE1/PSD3/ARHGEF18/RYBP/MORC3/MAPK8IP2/TSSK2/VGLL2/MTOR/DFNB31/TENM4/GAS2/LCE2B/GATM/GAPDHS/FGF22/NPTN/GJB2/SDCBP2/DKK3/VPS4A/BMP10/GNAS/GPER1/FFAR2/FLVCR1/GSTP1/TMOD4/NME7/ANXA2/SOX8/KCNIP2/NRG1/HLA-B/HLA-DOA/ANXA13/HLX/HMGA1/NR4A1/ACACB/HPCA/APBA2/HOXB3/HOXC4/HOXC5/HOXC6/HOXD3/HSD11B1/HSD17B2/HSP90AA1/HSP90AB1/HTR5A/ID3/RSPO2/FMN1/BARHL2/IGF1/IGF2/CYR61/LCE1C/LCE1D/LCE2D/IL1RN/IL6/AQP2/IL11RA/FOXK2/AQP5/INHBA/IRF1/ISL1/ITGA7/ITGB2/ITGB7/IVL/JUP/HILS1/KCNJ8/KDR/ACAT1/AMIGO3/INSC/HES5/SLC6A17/RESP18/AFF3/LAMA3/STMN1/LCK/ARHGDIA/LGALS9/LHCGR/LLGL1/LMNA/LMO2/LOX/LTB/SMAD3/MC2R/MEF2D/MAP3K1/MEOX1/MEOX2/MFI2/MFNG/MGAT1/MITF/LHX8/MYL2/DRG1/NEU1/NFATC3/NHLH2/NOV/NPPC/NRAS/NTF3/ATP5B/PALM/PARK2/UTP11L/LEF1/CEND1/ANGPT4/C11orf73/SIRT6/PDE6B/ATP8A2/PIK3CG/PITX2/PKHD1/PKM/PLA2G2A/PLAGL1/PML/RIPPLY3/RIPK4/CYTL1/SSH1/MOV10L1/ROBO4/MXRA8/FBLIM1/BNC2/PALMD/BANP/HERC6/PPP1CC/PIWIL2/ELP3/PRMT6/SMPD3/CHRNA9/LIMS2/PARVA/IFT122/ERMARD/MCTP2/CSGALNACT1/PRKD1/BIN3/MAPK3/MAP2K2/MRAP/HTRA1/CDC42SE1/PSMB4/RGMA/TRPC7/PSMD7/TENM2/ERMN/RDH14/METTL14/MARK4/PXN/ACTA2/RASGRF2/RPL29/S100A4/S100A6/BGLAP/SCT/CCL11/CCL17/STRA6/SFRP2/CXCR5/TRA2B/GZF1/DNAI2/SGK1/TMEM237/VPS33A/BMP4/SLC8A1/BMPR1B/SLIT1/BOK/SOX9/STK3/SUPT6H/BST2/TAF4B/TCEA1/ZEB1/ACTC1/TEAD3/TGM2/TCHH/TIMP3/TLE3/TLR5/TNFAIP3/TNFRSF1A/TRPC4/TRPC6/PHLDA2/TWIST1/CCR2/UCP1/WNT10B/YWHAG/ZAP70/ZNF7/PTP4A1/CACNB2/PAX8/CXCR4/FZD5/PPDPF/LST1/TMEM204/ZC3H12A/C6orf25/COL18A1/CALR/SLIRP/COLQ/CAST/CAPZB/SCRT1/SPATA16/ANTXR1/BFSP2/NR0B2/CASQ1/HOPX/PARD6B/TTBK1/KDM2B/LOXL3/CBX2/IFITM1/GAS7/SCIN/RUNX1/TP63/RUNX3/IRS2/ACTN1/FADD/TNFRSF11A/ALDH1A2/SPHK1/LIMD1/PIAS2/LDB2/CBFA2T2/RSAD2/AURKB/DAPL1/CD8A/ADIPOQ/H2AFY/MTL5/ARHGEF10/MICAL2/RAPGEF2/ULK2/CD79A/FGF19	372

GO:0032502	developmental process	376/901	5327/17046	6.04E-12	4.00E-09	3.14E-09	ABI1/CDH3/CDH13/FARP1/CDKN1C/SPEG/SPON2/ZBTB18/TACC2/PDPN/DMRT2/CELF1/TBR1/GJB6/TMED10/LECT1/ESM1/CHI3L1/CHRNA1/GPRIN1/FAT3/CLN5/FRMD6/CCR1/SLC38A10/SEZ6/CNP/COL9A3/COL11A1/COMP/SCLT1/ZFP42/ADM/IL31RA/CPM/CPS1/CRABP1/ZNF358/FAM101A/APCDD1/CSTA/CTGF/SMYD1/SH3D19/CYLD/ADRB3/ESCO2/CYP11A1/RNF168/BHLHA15/COCH/DIO3/DLG2/DMBT1/DNMT3A/DRD4/ECE1/EEF2/EFNA2/EGFR/EGR3/EIF4G1/A2M/ELK4/TMEM17/EML1/UNC13D/EPHA1/EPHA3/EPHB4/ESR1/SP8/FGA/FGF10/RASA3/VASH1/BTBD3/SBNO2/ACIN1/FOXL1/FOXC2/FOXO1/EXPH5/AKR1B1/SPG20/NFASC/EPB41L3/FLNB/DIP2A/FLOT2/NEDD4L/SYNE1/PSD3/ARHGEF18/RYBP/MORC3/MAPK8IP2/TSSK2/VGLL2/MTOR/DFNB31/TENM4/GAS2/LCE2B/GATM/GAPDHS/FGF22/NPTN/GJB2/SDCBP2/DKK3/VPS4A/BMP10/GNAS/GPER1/FFAR2/FLVCR1/GSTP1/TMOD4/NME7/ANXA2/SOX8/KCNIP2/NRG1/HLA-B/HLA-DOA/ANXA13/HLX/HMGA1/NR4A1/ACACB/HPCA/APBA2/HOXB3/HOXC4/HOXC5/HOXC6/HOXD3/HSD11B1/HSD17B2/HSP90AA1/HSP90AB1/HTR5A/TFAP2E/ID3/RSPO2/FMN1/BARHL2/IGF1/IGF2/CYR61/LCE1C/LCE1D/LCE2D/IL1RN/IL6/AQP2/IL11RA/FOXK2/AQP5/INHBA/IRF1/ISL1/ITGA7/ITGB2/ITGB7/IVL/JUP/HILS1/KCNJ8/KDR/ACAT1/KRT15/AMIGO3/INSC/HES5/SLC6A17/RESP18/AFF3/LAMA3/STMN1/LCK/ARHGDIA/LGALS9/LHCGR/LLGL1/LMNA/LMO2/LOX/LTB/SMAD3/MC2R/MEF2D/MAP3K1/MEOX1/MEOX2/MFI2/MFNG/MGAT1/MITF/LHX8/MYL2/DRG1/NEU1/NFATC3/NHLH2/NOV/NPPC/NRAS/NTF3/ATP5B/PALM/PARK2/UTP11L/LEF1/CEND1/ANGPT4/C11orf73/SIRT6/PDE6B/ATP8A2/PIK3CG/PITX2/PKHD1/PKM/PLA2G2A/PLAGL1/PML/RIPPLY3/RIPK4/CYTL1/SSH1/MOV10L1/ROBO4/MXRA8/FBLIM1/BNC2/PALMD/BANP/HERC6/PPP1CC/PIWIL2/ELP3/PRMT6/SMPD3/CHRNA9/LIMS2/PARVA/IFT122/ERMARD/MCTP2/CSGALNACT1/PRKD1/BIN3/MAPK3/MAP2K2/MRAP/HTRA1/CDC42SE1/PSMB4/RGMA/TRPC7/PSMD7/TENM2/ERMN/RDH14/METTL14/MARK4/PXN/ACTA2/RASGRF2/RPL29/S100A4/S100A6/BGLAP/SCT/CCL11/CCL17/NPAS3/STRA6/SFRP2/CXCR5/TRA2B/GZF1/DNAI2/SGK1/TMEM237/VPS33A/BMP4/SLC8A1/BMPR1B/SLIT1/BOK/SOX9/STK3/SUPT6H/BST2/TAF4B/TCEA1/ZEB1/ACTC1/TEAD3/TGM2/TCHH/TIMP3/TLE3/TLR5/TNFAIP3/TNFRSF1A/TRPC4/TRPC6/PHLDA2/TWIST1/CCR2/UCP1/WNT10B/YWHAG/ZAP70/ZNF7/PTP4A1/CACNB2/PAX8/CXCR4/FZD5/PPDPF/LST1/TMEM204/ZC3H12A/C6orf25/COL18A1/CALR/SLIRP/COLQ/CAST/CAPZB/SCRT1/SPATA16/ANTXR1/BFSP2/NR0B2/CASQ1/HOPX/PARD6B/TTBK1/KDM2B/LOXL3/CBX2/IFITM1/GAS7/SCIN/RUNX1/TP63/RUNX3/IRS2/ACTN1/FADD/TNFRSF11A/ALDH1A2/SPHK1/LIMD1/PIAS2/LDB2/CBFA2T2/RSAD2/AURKB/DAPL1/CD8A/ADIPOQ/H2AFY/MTL5/ARHGEF10/MICAL2/RAPGEF2/ULK2/CD79A/FGF19	376
GO:0050896	response to stimulus	502/901	7634/17046	8.90E-12	5.30E-09	4.17E-09	AKT3/ABI1/CDH3/TANK/TSPAN5/CDH13/FARP1/KLRG1/RCAN2/KCNMB2/CDKN1C/TCIRG1/MRVI1/SPON2/PDPN/TBR1/GJB6/HCST/NPFFR2/ADCY3/TMED10/LECT1/ESM1/HNRNPUL1/CHGA/CHI3L1/ERLIN2/PSIP1/EGLN2/TP53TG1/PTH2/PDAP1/CHRNA1/CHRNA2/CHRNA5/SORCS1/CIDEA/AP3S1/CCR1/SEZ6/TNFAIP8L1/CNP/COL9A3/COL11A1/MAP3K8/ADM/IL31RA/HUS1B/OR2A14/CPS1/PXDNL/CRABP1/TRPM6/MIB2/PARP4/MPP7/APCDD1/CTGF/SGOL1/PPM1L/CYLD/ADRB3/ESCO2/CYP11A1/TRPV3/CITED4/DDB1/DDOST/RNF168/ZNF366/BHLHA15/COCH/NLRP6/DMBT1/DNMT3A/ABAT/DRD4/DTNA/AGXT/EFNA2/EGFR/EGR3/EIF4G1/A2M/ANKRD23/TMEM17/UNC13D/EPHA1/EPHA3/EPHB4/ESR1/F11/SPATA13/FCGR2A/FGA/FGF10/FHIT/RASA3/PPM1E/VASH1/SBNO2/MSRB2/ACIN1/FOXL1/FOXC2/FOXO1/AKR1B1/SPG20/NFASC/FLNB/MLC1/RHOBTB2/NUP210/NEDD4L/PSD3/LARP1/PPP1R13B/PUM2/ARHGEF18/MAPK8IP2/TSSK2/MTOR/SLC37A4/GABBR1/RASGEF1C/ALS2CL/TENM4/ACOT11/RGS22/STEAP2/FBXL21/FBXO2/GATM/PLEK2/ADGRF1/RPS6KC1/GJA3/DNAJC2/FGF22/NPTN/GJB2/SDCBP2/PDE7B/DKK3/CYTH4/GPR162/BMP10/GNAS/GPR26/GPER1/FFAR2/GRB10/GRIK4/DNAJC15/SCG3/GSTP1/GUCY1A3/NME7/GPR132/GZMA/ANXA2/HAS1/SERPIND1/SOX8/KCNIP2/NRG1/ANXA6/HK1/HLA-B/HLA-DOA/HLA-DPA1/HLA-E/HLA-F/HLX/HMGA1/NR4A1/HPCA/HOXD3/HRH1/HSD17B2/HSPA1L/HSP90AA1/HSP90AB1/HTR3A/HTR5A/ID3/RSPO2/CD300E/IGF1/IGF2/CYR61/GPR142/LCE1D/IL1R1/IL1RN/IL6/IL10RA/AQP2/IL11RA/IL12RB2/IL15RA/IL16/INHBA/IRF1/AQP9/ISL1/ITGA7/ITGB2/ITGB7/ITIH4/IVL/JUP/CD82/KCNH2/KCNJ8/KCNMB1/KDR/ACAT1/IPO5/KRT15/HES5/AFF3/STMN1/OR2A5/LCK/LCP1/LDLR/ARHGDIA/LGALS9/LHCGR/LLGL1/LMNA/LMO2/RAB19/LOX/LTB/LTBP1/SMAD3/MC2R/MCC/ME1/MAP3K1/MFNG/SCGB2A1/MITF/ASGR1/MOV10/PLEKHG7/MT1A/NUDT1/MYH4/NDUFB4/NEDD9/ATP1A2/NFATC3/NMBR/NOV/NPPC/NRAS/NTF3/OAS2/OPRL1/OR2C1/OR3A2/SLC22A18/P2RY6/IL21R/PALM/ARHGEF3/PARK2/SPOCK3/LEF1/DDX47/ANGPT4/PDE4C/PDE7A/C11orf73/SIRT6/PDE6B/ATP8A2/PGAM2/PIK3CG/PITX2/PKHD1/PKM/PLA2G2A/PRKAG3/PML/GPR84/IL20RB/PNLIP/TLR9/TREM1/CYTL1/POMC/SSH1/PON1/RIN2/POU2AF1/ZDHHC13/APBB1IP/CYP2W1/PPP1CB/PPP1CC/ARHGEF10L/PRMT6/FANCI/MOB1A/SLC47A1/WDR33/SLC30A10/CHRNA9/SYBU/LIMS2/VAC14/PARVA/PRKAR1B/IFT122/MCTP2/LMBRD1/PAG1/PRKD1/WSB2/BIN3/MAPK3/MAP2K2/PRKRIR/PROC/MASP1/HTRA1/SLAMF8/CDC42SE1/PSMB4/PAK6/ARNTL2/RGMA/TRPC7/LPAR5/PSMD7/PTGFR/PLEKHG5/TENM2/CCAR2/PTPRCAP/PTPRE/PXN/CREBZF/ACTA2/RASGRF2/RFC2/TRIM27/RGR/RGS12/RIT2/RPA3/DEFB134/S100A4/S100A6/BGLAP/SCT/CCL11/CCL17/NPAS3/NOD2/TINAGL1/STRA6/SFRP2/CXCR5/ARHGAP9/SGK1/SYNDIG1L/MICAL1/TMEM237/VPS33A/BMP4/SLC8A1/SLC9A3/BMPR1B/SLIT1/BOK/SOX9/BPI/SRP68/STAT2/STK3/STK10/SUPT6H/BST2/VAMP2/TCEA1/TCEB2/ZEB1/ACTC1/TEAD3/TERF1/TGM2/TIMP3/TLE3/TLR5/TNFAIP3/TNFRSF1A/TNXB/TRAF1/TRAF5/TRPC4/TRPC6/TRPM2/TWIST1/CCR2/TNFRSF4/UPP1/WNT10B/YWHAG/ZAP70/CA7/CACNA1E/CACNB2/PAX8/CXCR4/FZD5/RAB7A/CARD14/BCL2L14/LST1/TMEM204/NLRX1/GPR157/ZC3H12A/FAAP100/CPEB4/C6orf25/COL18A1/EEPD1/CALR/UNC93B1/CAPS/CAPZB/HIST1H3A/ANTXR1/SLA2/CMAHP/BFSP2/NR0B2/CASQ1/HOPX/IL1F10/TRIM63/MGARP/RAE1/IFITM1/CDK10/KMO/RUNX1/TP63/RUNX3/IRS2/ACTN1/CRADD/FADD/TNFRSF11A/ALDH1A2/SPHK1/ENDOU/SKAP2/STBD1/HSPB3/LIMD1/TSPAN18/CCRL2/MAP7/PRC1/STARD13/PIAS2/ZFAND2A/MAP3K6/SYT7/ESAM/SLC16A3/RSAD2/AURKB/DAPL1/RCSD1/IL32/CD8A/TRIP10/ADIPOQ/ARHGAP29/LY86/RAB3D/SMAD5-AS1/RAB36/MTL5/ARHGEF10/N4BP1/NUP93/RAPGEF2/ULK2/CD79A/TELO2/IQSEC1/FGF19/NR1H4	502

GO:0007275	multicellular organismal development	329/901	4527/17046	9.99E-12	5.41E-09	4.25E-09	ABI1/CDH3/CDH13/FARP1/CDKN1C/SPEG/SPON2/ZBTB18/TACC2/PDPN/DMRT2/CELF1/TBR1/GJB6/TMED10/LECT1/ESM1/CHI3L1/CHRNA1/GPRIN1/FAT3/CLN5/CCR1/SLC38A10/SEZ6/CNP/COL9A3/COL11A1/COMP/SCLT1/ZFP42/ADM/IL31RA/CPS1/CRABP1/ZNF358/FAM101A/APCDD1/CSTA/CTGF/SMYD1/CYLD/ESCO2/CYP11A1/RNF168/DLG2/DMBT1/DNMT3A/DRD4/ECE1/EEF2/EFNA2/EGFR/EGR3/EIF4G1/EML1/EPHA1/EPHA3/EPHB4/ESR1/SP8/FGA/FGF10/RASA3/VASH1/BTBD3/SBNO2/ACIN1/FOXL1/FOXC2/FOXO1/EXPH5/AKR1B1/SPG20/NFASC/EPB41L3/FLNB/DIP2A/NEDD4L/PSD3/RYBP/MORC3/MAPK8IP2/TSSK2/VGLL2/MTOR/DFNB31/TENM4/LCE2B/GATM/FGF22/NPTN/GJB2/SDCBP2/DKK3/VPS4A/BMP10/GNAS/GPER1/FLVCR1/GSTP1/NME7/ANXA2/SOX8/KCNIP2/NRG1/HLA-B/HLA-DOA/HLX/NR4A1/ACACB/HPCA/APBA2/HOXB3/HOXC4/HOXC5/HOXC6/HOXD3/HSD11B1/HSD17B2/HSP90AA1/HSP90AB1/HTR5A/ID3/RSPO2/FMN1/BARHL2/IGF1/IGF2/CYR61/LCE1C/LCE1D/LCE2D/IL1RN/IL6/AQP2/AQP5/INHBA/IRF1/ISL1/ITGA7/ITGB2/IVL/JUP/HILS1/KCNJ8/KDR/ACAT1/AMIGO3/INSC/HES5/SLC6A17/RESP18/AFF3/LAMA3/STMN1/LCK/ARHGDIA/LGALS9/LHCGR/LLGL1/LMNA/LMO2/LOX/LTB/SMAD3/MC2R/MEF2D/MAP3K1/MEOX1/MEOX2/MFNG/MGAT1/MITF/LHX8/MYL2/DRG1/NEU1/NFATC3/NHLH2/NOV/NPPC/NRAS/NTF3/ATP5B/PALM/PARK2/UTP11L/LEF1/CEND1/ANGPT4/C11orf73/SIRT6/PDE6B/ATP8A2/PIK3CG/PITX2/PKHD1/PKM/PLAGL1/PML/RIPPLY3/CYTL1/SSH1/MOV10L1/ROBO4/MXRA8/BNC2/BANP/HERC6/PPP1CC/PIWIL2/ELP3/SMPD3/CHRNA9/LIMS2/PARVA/IFT122/ERMARD/MCTP2/CSGALNACT1/PRKD1/BIN3/MAPK3/MAP2K2/HTRA1/PSMB4/RGMA/TRPC7/PSMD7/TENM2/MARK4/PXN/ACTA2/RASGRF2/RPL29/S100A4/S100A6/BGLAP/SCT/CCL11/CCL17/STRA6/SFRP2/CXCR5/TRA2B/GZF1/DNAI2/SGK1/VPS33A/BMP4/SLC8A1/BMPR1B/SLIT1/BOK/SOX9/STK3/SUPT6H/BST2/TCEA1/ZEB1/ACTC1/TGM2/TCHH/TIMP3/TLE3/TLR5/TNFAIP3/TNFRSF1A/TRPC4/TRPC6/PHLDA2/TWIST1/CCR2/WNT10B/YWHAG/ZAP70/ZNF7/PTP4A1/CACNB2/PAX8/CXCR4/FZD5/PPDPF/LST1/TMEM204/ZC3H12A/C6orf25/COL18A1/CALR/COLQ/CAST/SCRT1/SPATA16/BFSP2/NR0B2/CASQ1/HOPX/PARD6B/TTBK1/KDM2B/LOXL3/CBX2/GAS7/SCIN/RUNX1/TP63/RUNX3/IRS2/ACTN1/FADD/TNFRSF11A/ALDH1A2/SPHK1/LIMD1/LDB2/CBFA2T2/RSAD2/AURKB/CD8A/ADIPOQ/H2AFY/MTL5/ARHGEF10/MICAL2/RAPGEF2/ULK2/CD79A/FGF19	329
GO:0048856	anatomical structure development	340/901	4751/17046	2.65E-11	1.32E-08	1.04E-08	ABI1/CDH3/CDH13/FARP1/CDKN1C/SPEG/SPON2/ZBTB18/TACC2/PDPN/DMRT2/CELF1/TBR1/GJB6/TMED10/LECT1/ESM1/CHI3L1/CHRNA1/GPRIN1/CLN5/FRMD6/CCR1/SLC38A10/SEZ6/CNP/COL9A3/COL11A1/COMP/SCLT1/ZFP42/ADM/IL31RA/CPM/CPS1/ZNF358/FAM101A/APCDD1/CSTA/CTGF/SMYD1/SH3D19/CYLD/ESCO2/CYP11A1/RNF168/BHLHA15/COCH/DLG2/DMBT1/DNMT3A/DRD4/ECE1/EEF2/EFNA2/EGFR/EGR3/EIF4G1/TMEM17/EML1/UNC13D/EPHA1/EPHA3/EPHB4/ESR1/SP8/FGA/FGF10/RASA3/VASH1/BTBD3/SBNO2/ACIN1/FOXL1/FOXC2/FOXO1/EXPH5/AKR1B1/SPG20/NFASC/EPB41L3/FLNB/FLOT2/NEDD4L/SYNE1/PSD3/ARHGEF18/MAPK8IP2/TSSK2/VGLL2/MTOR/DFNB31/TENM4/GAS2/LCE2B/GATM/GAPDHS/FGF22/NPTN/GJB2/SDCBP2/DKK3/BMP10/GNAS/GPER1/FLVCR1/GSTP1/TMOD4/NME7/ANXA2/SOX8/KCNIP2/NRG1/HLA-B/HLA-DOA/HLX/NR4A1/ACACB/HPCA/APBA2/HOXB3/HOXC4/HOXC5/HOXC6/HOXD3/HSD11B1/HSD17B2/HSP90AA1/HSP90AB1/HTR5A/TFAP2E/ID3/RSPO2/FMN1/BARHL2/IGF1/IGF2/CYR61/LCE1C/LCE1D/LCE2D/IL1RN/IL6/AQP2/IL11RA/FOXK2/AQP5/INHBA/IRF1/ISL1/ITGA7/ITGB2/ITGB7/IVL/JUP/HILS1/KCNJ8/KDR/ACAT1/KRT15/AMIGO3/INSC/HES5/SLC6A17/RESP18/AFF3/LAMA3/STMN1/LCK/ARHGDIA/LGALS9/LHCGR/LLGL1/LMNA/LMO2/LOX/LTB/SMAD3/MC2R/MEF2D/MAP3K1/MEOX1/MEOX2/MFI2/MGAT1/MITF/LHX8/MYL2/NEU1/NFATC3/NHLH2/NOV/NPPC/NRAS/NTF3/ATP5B/PALM/PARK2/UTP11L/LEF1/CEND1/ANGPT4/C11orf73/SIRT6/PDE6B/ATP8A2/PIK3CG/PITX2/PKHD1/PKM/PLAGL1/PML/RIPPLY3/RIPK4/CYTL1/SSH1/MOV10L1/ROBO4/MXRA8/FBLIM1/BNC2/PALMD/HERC6/PPP1CC/PIWIL2/ELP3/SMPD3/CHRNA9/LIMS2/PARVA/IFT122/CSGALNACT1/PRKD1/BIN3/MAPK3/MAP2K2/HTRA1/CDC42SE1/PSMB4/RGMA/TRPC7/PSMD7/TENM2/ERMN/MARK4/PXN/ACTA2/RASGRF2/S100A4/S100A6/BGLAP/SCT/CCL11/CCL17/STRA6/SFRP2/CXCR5/TRA2B/GZF1/DNAI2/SGK1/TMEM237/VPS33A/BMP4/SLC8A1/BMPR1B/SLIT1/BOK/SOX9/STK3/SUPT6H/TAF4B/TCEA1/ZEB1/ACTC1/TEAD3/TGM2/TCHH/TIMP3/TLE3/TLR5/TNFAIP3/TNFRSF1A/TRPC4/TRPC6/PHLDA2/TWIST1/CCR2/WNT10B/YWHAG/ZAP70/CACNB2/PAX8/CXCR4/FZD5/PPDPF/LST1/TMEM204/ZC3H12A/C6orf25/COL18A1/CALR/SLIRP/COLQ/CAST/CAPZB/SCRT1/ANTXR1/BFSP2/NR0B2/CASQ1/HOPX/PARD6B/TTBK1/KDM2B/LOXL3/GAS7/SCIN/RUNX1/TP63/RUNX3/IRS2/ACTN1/FADD/TNFRSF11A/ALDH1A2/SPHK1/LIMD1/LDB2/CBFA2T2/RSAD2/CD8A/ADIPOQ/H2AFY/ARHGEF10/MICAL2/RAPGEF2/ULK2/CD79A/FGF19	340
GO:0032501	multicellular organismal process	433/901	6425/17046	4.64E-11	2.12E-08	1.67E-08	ABI1/CDH3/CDH13/FARP1/RCAN2/KCNMB2/CDKN1C/SPEG/MRVI1/TRDN/SPON2/ZBTB18/TACC2/PDPN/DMRT2/CELF1/CELF2/TBR1/GJB6/ADCY3/TMED10/LECT1/ESM1/ADAM29/CHGA/CHI3L1/CHRNA1/CHRNA2/CHRNA5/GPRIN1/CIDEA/FAT3/CLN5/CCR1/ZG16B/SLC38A10/SEZ6/CNP/COL9A3/COL11A1/COMP/SCLT1/ZFP42/ADM/IL31RA/OR2A14/CPS1/CRABP1/ZNF358/CRYBB3/FAM101A/APCDD1/CSTA/CTGF/SMYD1/CYLD/ADRB3/ESCO2/CYP11A1/TRPV3/RNF168/COCH/NLRP6/DIO3/DLG2/DMBT1/DNMT3A/ABAT/DRD4/DTNA/ECE1/EEF2/EFNA2/EGFR/EGR3/EIF4G1/A2M/EML1/EPHA1/EPHA3/EPHB4/ESR1/F11/FAT2/SP8/FGA/FGF10/XRN2/RASA3/VASH1/BTBD3/SBNO2/ACIN1/FOXL1/FOXC2/FOXO1/EXPH5/AKR1B1/SPG20/NFASC/EPB41L3/FLNB/DIP2A/NEDD4L/PSD3/RYBP/MORC3/MAPK8IP2/TSSK2/VGLL2/MTOR/DFNB31/PNKD/TENM4/LCE2B/GATM/GAPDHS/GJA3/FGF22/NPTN/GJB2/AMPD2/SDCBP2/DKK3/VPS4A/AMPD3/BMP10/GNAS/IZUMO1/GPER1/FFAR2/FLVCR1/SCG3/GSTP1/TMOD4/GUCY1A3/NME7/ANXA2/HAS1/SERPIND1/SOX8/KCNIP2/NRG1/ANXA6/HLA-B/HLA-DOA/HLA-DPA1/HLA-E/HLX/NR4A1/ACACB/HPCA/APBA2/HOXB3/HOXC4/HOXC5/HOXC6/HOXD3/HRH1/HSD11B1/HSD17B2/ACADL/HSP90AA1/HSP90AB1/HTR3A/HTR5A/ID3/RSPO2/FMN1/BARHL2/IGF1/IGF2/CYR61/LCE1C/LCE1D/LCE2D/IL1R1/IL1RN/IL6/AQP2/IL12RB2/AQP5/INHBA/IRF1/AQP9/ISL1/ITGA7/ITGB2/IVL/JUP/HILS1/KCNH2/KCNJ8/KCNMB1/KDR/ACAT1/AMIGO3/INSC/HES5/SLC6A17/RESP18/AFF3/LAMA3/STMN1/OR2A5/LCK/LDLR/ARHGDIA/LGALS9/LHCGR/LLGL1/LMNA/LMO2/LOX/LTB/SMAD3/MC2R/MCC/MEF2D/MAP3K1/MEOX1/MEOX2/MFNG/MGAT1/MITF/LHX8/MYH4/MYL2/DRG1/NEU1/ATP1A2/NFATC3/NHLH2/NOV/NPPC/NRAS/NTF3/OPRL1/OR2C1/OR3A2/SLC22A18/ATP5B/PALM/PARK2/UTP11L/LEF1/CEND1/ANGPT4/C11orf73/SIRT6/PDE6B/ATP8A2/PGAM2/PIK3CG/PITX2/PKHD1/PKM/PLA2G2A/PLAGL1/SPA17/PML/RIPPLY3/IL20RB/PNLIP/TLR9/TREM1/CYTL1/POMC/SSH1/MOV10L1/APBB1IP/ROBO4/MXRA8/BNC2/BANP/PPP1CB/HERC6/PPP1CC/PIWIL2/ELP3/WDR33/SMPD3/CHRNA9/LIMS2/PARVA/PRKAR1B/IFT122/ERMARD/MCTP2/CSGALNACT1/PRKD1/BIN3/MAPK3/MAP2K2/PROC/HTRA1/PSMB4/RGMA/TRPC7/PSMD7/SLURP1/PTGFR/PLEKHG5/TENM2/ERMN/RDH14/METTL14/MARK4/PXN/ACTA2/RASGRF2/TRIM27/RGR/RPL29/S100A4/S100A6/BGLAP/SCT/CCL11/CCL17/NPAS3/NOD2/STRA6/SFRP2/CXCR5/TRA2B/GZF1/DNAI2/SGK1/VPS33A/BMP4/SLC8A1/BMPR1B/SLIT1/BOK/SOX9/BPI/STK3/SUPT6H/BST2/TAF4B/TBP/TCEA1/ZEB1/ACTC1/TEAD3/TGM2/TCHH/TIMP3/TLE3/TLR5/TNFAIP3/TNFRSF1A/TNXB/TRPC4/TRPC6/PHLDA2/TWIST1/CCR2/TNFRSF4/WNT10B/YWHAG/ZAP70/ZNF7/CACNA1E/PTP4A1/CACNB2/PAX8/CXCR4/FZD5/RAB7A/PPDPF/LST1/TMEM204/NLRX1/CALD1/ZC3H12A/RAB11FIP1/C6orf25/COL18A1/CALR/SLIRP/COLQ/CAST/CAPZB/SH3BGRL3/SCRT1/HIST1H3A/SPATA16/BFSP2/NR0B2/CASQ1/HOPX/PARD6B/TTBK1/TRIM63/KDM2B/LOXL3/CBX2/IFITM1/GAS7/SCIN/RUNX1/TP63/RUNX3/IRS2/ACTN1/FADD/TNFRSF11A/ALDH1A2/SPHK1/CCNA1/ENDOU/STBD1/LIMD1/PIAS2/LDB2/ESAM/SLC16A3/CBFA2T2/RSAD2/AURKB/RCSD1/CD8A/ADIPOQ/H2AFY/MTL5/ARHGEF10/MICAL2/RAPGEF2/ULK2/CD79A/FGF19	433

GO:0044707	single-multicellular organism process	419/901	6214/17046	1.36E-10	5.78E-08	4.55E-08	ABI1/CDH3/CDH13/FARP1/RCAN2/KCNMB2/CDKN1C/SPEG/MRVI1/TRDN/SPON2/ZBTB18/TACC2/PDPN/DMRT2/CELF1/CELF2/TBR1/GJB6/ADCY3/TMED10/LECT1/ESM1/CHGA/CHI3L1/CHRNA1/CHRNA2/CHRNA5/GPRIN1/CIDEA/FAT3/CLN5/CCR1/ZG16B/SLC38A10/SEZ6/CNP/COL9A3/COL11A1/COMP/SCLT1/ZFP42/ADM/IL31RA/OR2A14/CPS1/CRABP1/ZNF358/CRYBB3/FAM101A/APCDD1/CSTA/CTGF/SMYD1/CYLD/ADRB3/ESCO2/CYP11A1/TRPV3/RNF168/COCH/NLRP6/DIO3/DLG2/DMBT1/DNMT3A/ABAT/DRD4/DTNA/ECE1/EEF2/EFNA2/EGFR/EGR3/EIF4G1/A2M/EML1/EPHA1/EPHA3/EPHB4/ESR1/F11/FAT2/SP8/FGA/FGF10/RASA3/VASH1/BTBD3/SBNO2/ACIN1/FOXL1/FOXC2/FOXO1/EXPH5/AKR1B1/SPG20/NFASC/EPB41L3/FLNB/DIP2A/NEDD4L/PSD3/RYBP/MORC3/MAPK8IP2/TSSK2/VGLL2/MTOR/DFNB31/PNKD/TENM4/LCE2B/GATM/GJA3/FGF22/NPTN/GJB2/AMPD2/SDCBP2/DKK3/VPS4A/AMPD3/BMP10/GNAS/GPER1/FFAR2/FLVCR1/SCG3/GSTP1/TMOD4/GUCY1A3/NME7/ANXA2/SERPIND1/SOX8/KCNIP2/NRG1/ANXA6/HLA-B/HLA-DOA/HLA-DPA1/HLA-E/HLX/NR4A1/ACACB/HPCA/APBA2/HOXB3/HOXC4/HOXC5/HOXC6/HOXD3/HRH1/HSD11B1/HSD17B2/ACADL/HSP90AA1/HSP90AB1/HTR3A/HTR5A/ID3/RSPO2/FMN1/BARHL2/IGF1/IGF2/CYR61/LCE1C/LCE1D/LCE2D/IL1RN/IL6/AQP2/IL12RB2/AQP5/INHBA/IRF1/AQP9/ISL1/ITGA7/ITGB2/IVL/JUP/HILS1/KCNH2/KCNJ8/KCNMB1/KDR/ACAT1/AMIGO3/INSC/HES5/SLC6A17/RESP18/AFF3/LAMA3/STMN1/OR2A5/LCK/LDLR/ARHGDIA/LGALS9/LHCGR/LLGL1/LMNA/LMO2/LOX/LTB/SMAD3/MC2R/MCC/MEF2D/MAP3K1/MEOX1/MEOX2/MFNG/MGAT1/MITF/LHX8/MYH4/MYL2/DRG1/NEU1/ATP1A2/NFATC3/NHLH2/NOV/NPPC/NRAS/NTF3/OPRL1/OR2C1/OR3A2/SLC22A18/ATP5B/PALM/PARK2/UTP11L/LEF1/CEND1/ANGPT4/C11orf73/SIRT6/PDE6B/ATP8A2/PGAM2/PIK3CG/PITX2/PKHD1/PKM/PLA2G2A/PLAGL1/PML/RIPPLY3/IL20RB/PNLIP/TLR9/TREM1/CYTL1/POMC/SSH1/MOV10L1/APBB1IP/ROBO4/MXRA8/BNC2/BANP/PPP1CB/HERC6/PPP1CC/PIWIL2/ELP3/SMPD3/CHRNA9/LIMS2/PARVA/PRKAR1B/IFT122/ERMARD/MCTP2/CSGALNACT1/PRKD1/BIN3/MAPK3/MAP2K2/PROC/HTRA1/PSMB4/RGMA/TRPC7/PSMD7/SLURP1/PLEKHG5/TENM2/ERMN/RDH14/METTL14/MARK4/PXN/ACTA2/RASGRF2/TRIM27/RGR/RPL29/S100A4/S100A6/BGLAP/SCT/CCL11/CCL17/NPAS3/NOD2/STRA6/SFRP2/CXCR5/TRA2B/GZF1/DNAI2/SGK1/VPS33A/BMP4/SLC8A1/BMPR1B/SLIT1/BOK/SOX9/BPI/STK3/SUPT6H/BST2/TCEA1/ZEB1/ACTC1/TEAD3/TGM2/TCHH/TIMP3/TLE3/TLR5/TNFAIP3/TNFRSF1A/TNXB/TRPC4/TRPC6/PHLDA2/TWIST1/CCR2/TNFRSF4/WNT10B/YWHAG/ZAP70/ZNF7/CACNA1E/PTP4A1/CACNB2/PAX8/CXCR4/FZD5/RAB7A/PPDPF/LST1/TMEM204/NLRX1/CALD1/ZC3H12A/RAB11FIP1/C6orf25/COL18A1/CALR/COLQ/CAST/CAPZB/SH3BGRL3/SCRT1/HIST1H3A/SPATA16/BFSP2/NR0B2/CASQ1/HOPX/PARD6B/TTBK1/TRIM63/KDM2B/LOXL3/CBX2/IFITM1/GAS7/SCIN/RUNX1/TP63/RUNX3/IRS2/ACTN1/FADD/TNFRSF11A/ALDH1A2/SPHK1/STBD1/LIMD1/PIAS2/LDB2/ESAM/SLC16A3/CBFA2T2/RSAD2/AURKB/RCSD1/CD8A/ADIPOQ/H2AFY/MTL5/ARHGEF10/MICAL2/RAPGEF2/ULK2/CD79A/FGF19	419
GO:0048518	positive regulation of biological process	347/901	4960/17046	2.79E-10	1.11E-07	8.73E-08	ABI1/CDH3/TANK/TSPAN5/CDH13/FARP1/CDKN1C/TCIRG1/TRDN/SPON2/PITRM1/PDPN/DMRT2/CELF1/TBR1/HCST/ADCY3/RER1/ESM1/CHGA/CHI3L1/ERLIN2/PSIP1/EGLN2/CIDEA/CCR1/SLC51B/SEZ6/MAP3K8/ADM/IL31RA/EGFLAM/CPS1/MIB2/MPP7/CTGF/SMYD1/SH3D19/CYLD/ADRB3/FITM1/TRPV3/CITED4/DDB1/RNF168/BHLHA15/COCH/NLRP6/DIO3/DMBT1/DNMT3A/ABAT/DRD4/ECE1/EEF2/EGFR/EGR3/EIF4G1/A2M/UNC13D/EPHA1/EPHA3/ESR1/F11/SPATA13/FCGR2A/FGA/FGF10/RASA3/PPM1E/SBNO2/ACIN1/FOXC2/TBC1D9B/FOXO1/EXPH5/AKR1B1/GGA3/FLOT2/MLC1/TBC1D1/NEDD4L/PSD3/LARP1/PUM2/ARHGEF18/MAPK8IP2/VGLL2/MTOR/RASGEF1C/RNF144B/ALS2CL/TENM4/RGS22/GAPDHS/PABPC1/DNAJC2/FGF22/NPTN/CYTH4/GLS2/VPS4A/BMP10/GNAS/GPR26/GPER1/DOK7/FFAR2/GRB10/DNAJC15/GSTP1/BRF1/GUCY1A3/GZMA/ANXA2/SOX8/NRG1/HK1/HLA-B/HLA-DPA1/ANXA13/HLA-E/HLX/HMGA1/NR4A1/ACACB/HPCA/HOXD3/AGFG2/HRH1/HSPA1L/HSP90AA1/HSP90AB1/TFAP2E/ID3/RSPO2/FMN1/BARHL2/IGF1/IGF2/CYR61/IL1RN/IL6/IL12RB2/IL16/FOXK2/INHBA/IRF1/ISL1/ITGB2/JUP/KCNH2/KDR/IPO5/AMIGO3/HES5/STMN1/LCK/LCP1/LDLR/ARHGDIA/LGALS9/LHCGR/LLGL1/LMNA/LMO2/LTB/SMAD3/MC2R/MEF2D/MAP3K1/MEOX1/MEOX2/MFI2/MFNG/MITF/PLEKHG7/NEU1/NFATC3/NFYB/NHLH2/NOV/NPPC/NRAS/NTF3/OPRL1/P2RY6/PALM/ARHGEF3/PARK2/UTP11L/LEF1/PRR16/ANGPT4/SIRT6/ATP8A2/PIK3CG/PITX2/PKHD1/PLA2G2A/PLAGL1/PML/IL20RB/PNLIP/RIPK4/TLR9/CYTL1/POMC/PON1/RIN2/ZDHHC13/APBB1IP/BANP/PIWIL2/ELP3/ARHGEF10L/GOLPH3L/FANCI/SMPD3/LIMS2/PRKAR1B/PAG1/PRKD1/MAPK3/MAP2K2/MRAP/MASP1/HTRA1/PSMB4/PAK6/ARNTL2/RGMA/PSMD7/ACTR3B/PTGFR/PLEKHG5/TENM2/MARK4/CCAR2/PXN/ACTA2/RASGRF2/TRIM27/RGS12/S100A4/S100A6/SCT/CCL11/CCL17/NPAS3/NOD2/STRA6/SFRP2/CXCR5/ARHGAP9/TRA2B/SGK1/BMP4/ZNF649/SLC8A1/BMPR1B/BOK/SOX9/STK3/STK10/SUPT6H/BST2/VAMP2/TBP/TCEA1/TCEB2/ZEB1/ACTC1/TEAD3/TERF1/TGM2/TLR5/TNFAIP3/TNFRSF1A/TRAF1/TRAF5/TRPC6/TWIST1/CCR2/TNFRSF4/WNT10B/YWHAG/ZAP70/CA7/PTP4A1/CACNB2/PAX8/CXCR4/FZD5/RAB7A/CARD14/BCL2L14/NLRX1/CSPP1/ZC3H12A/COL18A1/CALR/UNC93B1/SH3BGRL3/SLA2/NR0B2/HOPX/MGARP/IFITM1/SCIN/CDK10/RUNX1/TP63/RUNX3/IRS2/CRADD/FADD/TNFRSF11A/ALDH1A2/SPHK1/SKAP2/LIMD1/PRC1/STARD13/PIAS2/ZFAND2A/MAP3K6/SYT7/LDB2/CBFA2T2/RSAD2/AURKB/CD8A/ADIPOQ/ARHGAP29/LY86/RAB3D/H2AFY/ARHGEF10/MICAL2/RAPGEF2/USP6NL/CD79A/RABGAP1L/IQSEC1/FGF19/NR1H4	347
GO:0048731	system development	292/901	4014/17046	3.05E-10	1.14E-07	8.94E-08	ABI1/CDH3/CDH13/FARP1/CDKN1C/SPEG/SPON2/ZBTB18/TACC2/PDPN/DMRT2/TBR1/GJB6/TMED10/LECT1/ESM1/CHI3L1/CHRNA1/GPRIN1/CLN5/CCR1/SLC38A10/SEZ6/CNP/COL9A3/COL11A1/COMP/SCLT1/ZFP42/ADM/IL31RA/CPS1/ZNF358/FAM101A/APCDD1/CSTA/CTGF/SMYD1/CYLD/ESCO2/CYP11A1/RNF168/DLG2/DNMT3A/DRD4/ECE1/EEF2/EFNA2/EGFR/EGR3/EIF4G1/EML1/EPHA1/EPHA3/EPHB4/ESR1/FGA/FGF10/RASA3/VASH1/BTBD3/SBNO2/ACIN1/FOXL1/FOXC2/FOXO1/EXPH5/AKR1B1/SPG20/NFASC/EPB41L3/FLNB/NEDD4L/PSD3/MAPK8IP2/VGLL2/MTOR/DFNB31/TENM4/LCE2B/GATM/FGF22/NPTN/GJB2/SDCBP2/DKK3/BMP10/GNAS/GPER1/FLVCR1/GSTP1/NME7/ANXA2/SOX8/KCNIP2/NRG1/HLA-B/HLA-DOA/HLX/NR4A1/ACACB/HPCA/APBA2/HOXB3/HOXC4/HOXC5/HOXC6/HOXD3/HSD11B1/HSD17B2/HSP90AA1/HSP90AB1/HTR5A/ID3/RSPO2/FMN1/BARHL2/IGF1/IGF2/CYR61/LCE1C/LCE1D/LCE2D/IL6/AQP2/AQP5/INHBA/IRF1/ISL1/ITGA7/IVL/JUP/KCNJ8/KDR/ACAT1/AMIGO3/INSC/HES5/SLC6A17/STMN1/LCK/ARHGDIA/LGALS9/LHCGR/LLGL1/LMNA/LMO2/LOX/LTB/SMAD3/MC2R/MEF2D/MAP3K1/MEOX1/MEOX2/MITF/LHX8/MYL2/NEU1/NFATC3/NHLH2/NOV/NPPC/NRAS/NTF3/ATP5B/PALM/PARK2/UTP11L/LEF1/CEND1/ANGPT4/C11orf73/SIRT6/PDE6B/ATP8A2/PIK3CG/PITX2/PKHD1/PKM/PLAGL1/PML/RIPPLY3/CYTL1/SSH1/ROBO4/MXRA8/BNC2/HERC6/PPP1CC/ELP3/SMPD3/CHRNA9/LIMS2/PARVA/IFT122/CSGALNACT1/PRKD1/BIN3/MAPK3/MAP2K2/HTRA1/PSMB4/RGMA/TRPC7/PSMD7/TENM2/MARK4/ACTA2/RASGRF2/S100A4/S100A6/BGLAP/SCT/CCL11/STRA6/SFRP2/CXCR5/TRA2B/GZF1/SGK1/VPS33A/BMP4/SLC8A1/BMPR1B/SLIT1/BOK/SOX9/STK3/SUPT6H/TCEA1/ZEB1/ACTC1/TGM2/TCHH/TIMP3/TLE3/TLR5/TNFAIP3/TRPC4/TRPC6/PHLDA2/TWIST1/CCR2/WNT10B/YWHAG/ZAP70/CACNB2/PAX8/CXCR4/FZD5/PPDPF/LST1/TMEM204/ZC3H12A/C6orf25/COL18A1/CALR/COLQ/CAST/SCRT1/BFSP2/NR0B2/CASQ1/HOPX/PARD6B/TTBK1/KDM2B/LOXL3/GAS7/SCIN/RUNX1/TP63/RUNX3/IRS2/ACTN1/FADD/TNFRSF11A/ALDH1A2/SPHK1/LDB2/CBFA2T2/RSAD2/CD8A/ADIPOQ/H2AFY/ARHGEF10/MICAL2/RAPGEF2/ULK2/CD79A/FGF19	292

GO:0009653	anatomical structure morphogenesis	205/901	2579/17046	3.27E-10	1.15E-07	9.01E-08	ABI1/CDH13/FARP1/CDKN1C/SPON2/PDPN/DMRT2/TBR1/GJB6/LECT1/ESM1/CHI3L1/FRMD6/CNP/COL9A3/COL11A1/COMP/SCLT1/ADM/CPM/ZNF358/FAM101A/CTGF/SH3D19/CYLD/COCH/ECE1/EFNA2/EGFR/EGR3/TMEM17/UNC13D/EPHA1/EPHA3/EPHB4/ESR1/SP8/FGA/FGF10/RASA3/VASH1/BTBD3/SBNO2/FOXL1/FOXC2/FOXO1/SPG20/NFASC/EPB41L3/FLNB/NEDD4L/ARHGEF18/MAPK8IP2/TENM4/GAS2/FGF22/DKK3/BMP10/GNAS/FLVCR1/TMOD4/ANXA2/SOX8/NRG1/HLX/NR4A1/HOXB3/HOXC4/HOXD3/HSP90AA1/HSP90AB1/ID3/RSPO2/FMN1/BARHL2/IGF1/IGF2/CYR61/IL1RN/IL6/FOXK2/AQP5/INHBA/ISL1/ITGA7/ITGB2/ITGB7/KDR/HES5/AFF3/LAMA3/STMN1/ARHGDIA/LLGL1/SMAD3/MEF2D/MAP3K1/MEOX1/MEOX2/MFI2/LHX8/MYL2/NFATC3/NOV/NPPC/NRAS/NTF3/ATP5B/PALM/PARK2/LEF1/CEND1/ANGPT4/SIRT6/ATP8A2/PIK3CG/PITX2/PKHD1/PML/RIPK4/SSH1/ROBO4/FBLIM1/BNC2/PALMD/CHRNA9/LIMS2/PARVA/IFT122/CSGALNACT1/PRKD1/BIN3/MAPK3/MAP2K2/HTRA1/CDC42SE1/PSMB4/RGMA/TRPC7/PSMD7/TENM2/ERMN/PXN/ACTA2/RASGRF2/S100A4/S100A6/BGLAP/CCL11/STRA6/SFRP2/GZF1/DNAI2/SGK1/TMEM237/VPS33A/BMP4/SLC8A1/BMPR1B/SLIT1/SOX9/STK3/ZEB1/ACTC1/TGM2/TLE3/TNFAIP3/TRPC4/TRPC6/PHLDA2/TWIST1/CCR2/WNT10B/CACNB2/PAX8/FZD5/LST1/ZC3H12A/C6orf25/COL18A1/CALR/SLIRP/CAST/CAPZB/ANTXR1/CASQ1/HOPX/PARD6B/TTBK1/KDM2B/LOXL3/GAS7/RUNX1/TP63/RUNX3/IRS2/ACTN1/ALDH1A2/SPHK1/LIMD1/ADIPOQ/MICAL2/RAPGEF2/ULK2/FGF19	205
GO:0006928	movement of cell or subcellular component	152/901	1789/17046	1.37E-09	4.55E-07	3.58E-07	ABI1/CDH13/SPON2/PDPN/TBR1/ADCY3/CHGA/KIF12/AP3S1/FRMD6/CCR1/COL9A3/APCDD1/KLC3/CTGF/DNAH6/DNAH8/EFNA2/EGFR/EGR3/DNAH12/EPHA1/EPHA3/EPHB4/FAT2/SPATA13/PHACTR1/FGF10/RASA3/VASH1/FOXC2/NFASC/GAPDHS/FGF22/BMP10/GPER1/FFAR2/NME7/HAS1/SOX8/NRG1/NR4A1/HRH1/HSP90AA1/HSP90AB1/BARHL2/IGF1/CYR61/IL1RN/IL6/IL16/ISL1/ITGA7/ITGB2/ITGB7/JUP/KDR/KIF25/LAMA3/STMN1/LCK/LCP1/ARHGDIA/LGALS9/LMNA/SMAD3/MCC/MAP3K1/MYH4/MYL2/ATP1A2/NOV/NRAS/NTF3/P2RY6/ATP5B/PALM/LEF1/CEND1/ANGPT4/PIK3CG/PITX2/PKHD1/SPA17/PML/TREM1/ROBO4/ELP3/PARVA/IFT122/PRKD1/BIN3/MAPK3/MAP2K2/PROC/PSMB4/PAK6/RGMA/TRPC7/PSMD7/PLEKHG5/TENM2/FAM60A/RASGRF2/CCL11/CCL17/NOD2/SFRP2/CXCR5/DNAI2/SGK1/BMP4/SLC8A1/BMPR1B/SLIT1/SOX9/STK10/BST2/ACTC1/TRPC4/TRPC6/PHLDA2/TWIST1/CCR2/ZAP70/CACNA1E/PTP4A1/CACNB2/CXCR4/CALD1/COL18A1/CALR/SLIRP/CAPZB/SH3BGRL3/SCRT1/DYNLRB2/PARD6B/MGARP/IFITM1/RUNX3/IRS2/ACTN1/FADD/TNFRSF11A/SPHK1/LIMD1/ESAM/SLC16A3/ADIPOQ/RAPGEF2/FGF19	152
GO:0048869	cellular developmental process	271/901	3716/17046	1.54E-09	4.84E-07	3.81E-07	ABI1/CDH3/FARP1/CDKN1C/SPEG/SPON2/ZBTB18/TACC2/PDPN/CELF1/TBR1/LECT1/GPRIN1/CLN5/FRMD6/CCR1/SEZ6/CNP/COL9A3/COL11A1/SCLT1/ZFP42/ADM/IL31RA/CPS1/FAM101A/APCDD1/CSTA/CTGF/SMYD1/SH3D19/CYLD/ADRB3/ESCO2/CYP11A1/BHLHA15/COCH/DMBT1/DNMT3A/EEF2/EFNA2/EGFR/EGR3/EIF4G1/A2M/ELK4/TMEM17/EML1/UNC13D/EPHA1/EPHA3/EPHB4/ESR1/FGA/FGF10/RASA3/BTBD3/SBNO2/ACIN1/FOXL1/FOXC2/FOXO1/EXPH5/SPG20/NFASC/EPB41L3/FLNB/FLOT2/NEDD4L/SYNE1/PSD3/ARHGEF18/MAPK8IP2/TSSK2/MTOR/DFNB31/TENM4/GAS2/LCE2B/GAPDHS/FGF22/NPTN/BMP10/GNAS/GPER1/FFAR2/FLVCR1/TMOD4/ANXA2/SOX8/KCNIP2/NRG1/HLA-B/HLA-DOA/ANXA13/HLX/NR4A1/HOXB3/HOXD3/HSP90AA1/HSP90AB1/ID3/RSPO2/FMN1/BARHL2/IGF1/IGF2/CYR61/LCE1C/LCE1D/LCE2D/IL6/FOXK2/INHBA/IRF1/ISL1/ITGA7/ITGB2/ITGB7/IVL/JUP/HILS1/KDR/INSC/HES5/LAMA3/STMN1/LCK/ARHGDIA/LGALS9/LLGL1/LMNA/SMAD3/MEF2D/MAP3K1/MEOX1/MFI2/MITF/LHX8/MYL2/NEU1/NFATC3/NHLH2/NOV/NPPC/NRAS/NTF3/ATP5B/PALM/PARK2/LEF1/CEND1/SIRT6/ATP8A2/PITX2/PKHD1/PLA2G2A/PLAGL1/PML/CYTL1/SSH1/MOV10L1/ROBO4/MXRA8/FBLIM1/PALMD/HERC6/PPP1CC/PIWIL2/ELP3/SMPD3/PARVA/IFT122/PRKD1/BIN3/MAPK3/MAP2K2/MRAP/HTRA1/CDC42SE1/PSMB4/RGMA/TRPC7/PSMD7/TENM2/ERMN/RDH14/PXN/ACTA2/RASGRF2/S100A4/S100A6/BGLAP/CCL11/CCL17/SFRP2/DNAI2/SGK1/TMEM237/VPS33A/BMP4/SLC8A1/BMPR1B/SLIT1/BOK/SOX9/STK3/SUPT6H/TAF4B/TCEA1/ZEB1/ACTC1/TEAD3/TCHH/TNFRSF1A/TRPC4/TRPC6/TWIST1/UCP1/WNT10B/YWHAG/ZAP70/CACNB2/PAX8/CXCR4/FZD5/PPDPF/LST1/TMEM204/ZC3H12A/C6orf25/COL18A1/CALR/SLIRP/CAST/CAPZB/SCRT1/SPATA16/ANTXR1/BFSP2/CASQ1/HOPX/PARD6B/TTBK1/LOXL3/CBX2/IFITM1/GAS7/SCIN/RUNX1/TP63/RUNX3/IRS2/ACTN1/FADD/TNFRSF11A/ALDH1A2/LIMD1/PIAS2/CBFA2T2/RSAD2/DAPL1/CD8A/ADIPOQ/H2AFY/MTL5/ARHGEF10/RAPGEF2/ULK2/CD79A/FGF19	271
GO:0048513	organ development	218/901	2848/17046	2.23E-09	6.63E-07	5.21E-07	ABI1/CDH3/CDKN1C/SPEG/ZBTB18/TACC2/PDPN/TBR1/GJB6/TMED10/LECT1/CHI3L1/CHRNA1/CLN5/CCR1/SLC38A10/SEZ6/CNP/COL9A3/COL11A1/COMP/ZFP42/ADM/IL31RA/CPS1/FAM101A/APCDD1/CSTA/CTGF/SMYD1/CYLD/ESCO2/CYP11A1/DRD4/ECE1/EEF2/EFNA2/EGFR/EGR3/EML1/EPHA3/EPHB4/ESR1/FGA/FGF10/BTBD3/SBNO2/ACIN1/FOXL1/FOXC2/FOXO1/EXPH5/AKR1B1/SPG20/FLNB/VGLL2/MTOR/DFNB31/TENM4/LCE2B/GATM/GJB2/DKK3/BMP10/GNAS/FLVCR1/NME7/ANXA2/SOX8/NRG1/HLA-B/HLA-DOA/HLX/ACACB/HPCA/HOXB3/HOXC4/HOXD3/HSD11B1/HSD17B2/HSP90AB1/HTR5A/ID3/RSPO2/FMN1/BARHL2/IGF1/IGF2/CYR61/LCE1C/LCE1D/LCE2D/IL6/AQP2/AQP5/INHBA/IRF1/ISL1/ITGA7/IVL/JUP/KCNJ8/KDR/ACAT1/AMIGO3/INSC/HES5/SLC6A17/STMN1/LCK/LGALS9/LHCGR/LMNA/LMO2/LOX/LTB/SMAD3/MC2R/MEF2D/MAP3K1/MEOX1/MEOX2/MITF/LHX8/MYL2/NFATC3/NOV/NPPC/NRAS/NTF3/LEF1/CEND1/C11orf73/SIRT6/PDE6B/ATP8A2/PITX2/PKHD1/PKM/PLAGL1/PML/RIPPLY3/CYTL1/BNC2/HERC6/SMPD3/CHRNA9/LIMS2/PARVA/IFT122/CSGALNACT1/BIN3/MAPK3/MAP2K2/HTRA1/ACTA2/S100A4/BGLAP/SCT/CCL11/STRA6/SFRP2/CXCR5/TRA2B/GZF1/VPS33A/BMP4/SLC8A1/BMPR1B/SLIT1/BOK/SOX9/STK3/TCEA1/ZEB1/ACTC1/TGM2/TCHH/TLE3/TLR5/TNFAIP3/PHLDA2/TWIST1/WNT10B/ZAP70/PAX8/FZD5/PPDPF/C6orf25/COL18A1/CALR/CAST/BFSP2/NR0B2/CASQ1/HOPX/TTBK1/KDM2B/LOXL3/SCIN/RUNX1/TP63/RUNX3/IRS2/ACTN1/FADD/TNFRSF11A/ALDH1A2/SPHK1/LDB2/RSAD2/CD8A/ADIPOQ/H2AFY/MICAL2/RAPGEF2/CD79A/FGF19	218
GO:0010033	response to organic substance	207/901	2687/17046	3.90E-09	1.04E-06	8.19E-07	CDH13/CDKN1C/TCIRG1/SPON2/GJB6/NPFFR2/ADCY3/TMED10/LECT1/CHI3L1/ERLIN2/CHRNA1/CHRNA2/CHRNA5/CIDEA/AP3S1/CCR1/CNP/ADM/IL31RA/CPS1/CTGF/CYP11A1/CITED4/DDOST/ZNF366/BHLHA15/DNMT3A/ABAT/DRD4/AGXT/EGFR/EGR3/EIF4G1/EPHA3/ESR1/FGA/FGF10/RASA3/SBNO2/FOXC2/FOXO1/AKR1B1/SPG20/FLNB/MLC1/NUP210/NEDD4L/ARHGEF18/MTOR/STEAP2/FBXO2/GATM/FGF22/NPTN/GJB2/BMP10/GNAS/GPER1/FFAR2/GRB10/GSTP1/GUCY1A3/HAS1/NRG1/HLA-B/HLA-DPA1/HLA-E/HLA-F/NR4A1/HPCA/HRH1/HSD17B2/HSPA1L/HSP90AA1/HSP90AB1/HTR3A/HTR5A/IGF2/CYR61/IL1R1/IL1RN/IL6/IL10RA/IL11RA/IL12RB2/IL15RA/INHBA/IRF1/AQP9/ISL1/ITIH4/JUP/KCNJ8/KDR/ACAT1/IPO5/HES5/AFF3/LCK/ARHGDIA/LGALS9/LHCGR/LMNA/LMO2/LOX/LTB/LTBP1/SMAD3/ME1/MAP3K1/MOV10/ATP1A2/NPPC/NRAS/OAS2/OPRL1/P2RY6/IL21R/ARHGEF3/PARK2/LEF1/PIK3CG/PITX2/PKM/PRKAG3/PML/IL20RB/TLR9/SSH1/PON1/PPP1CB/PPP1CC/CHRNA9/SYBU/PRKAR1B/LMBRD1/PRKD1/MAPK3/MAP2K2/HTRA1/PSMB4/RGMA/PSMD7/PTGFR/PLEKHG5/PTPRE/PXN/RASGRF2/RIT2/BGLAP/CCL11/CCL17/NOD2/SFRP2/CXCR5/BMP4/SLC8A1/SLC9A3/BMPR1B/SOX9/STAT2/BST2/VAMP2/ZEB1/ACTC1/TIMP3/TLR5/TNFAIP3/TNFRSF1A/TWIST1/CCR2/TNFRSF4/WNT10B/CACNA1E/PAX8/CXCR4/FZD5/RAB7A/CARD14/TMEM204/ZC3H12A/CPEB4/CALR/NR0B2/CASQ1/IL1F10/TRIM63/MGARP/RAE1/IFITM1/RUNX1/RUNX3/IRS2/FADD/TNFRSF11A/ALDH1A2/SPHK1/HSPB3/CCRL2/RSAD2/ADIPOQ/LY86/NUP93/RAPGEF2/FGF19/NR1H4	207

GO:0007154	cell communication	390/901	5832/17046	4.00E-09	1.04E-06	8.19E-07	AKT3/ABI1/CDH3/TANK/TSPAN5/CDH13/FARP1/KLRG1/RCAN2/KCNMB2/CDKN1C/TCIRG1/MRVI1/TRDN/PDPN/GJB6/HCST/NPFFR2/ADCY3/LECT1/ESM1/CHI3L1/ERLIN2/EGLN2/PTH2/PDAP1/CHRNA1/CHRNA2/CHRNA5/SORCS1/CIDEA/PANX3/AP3S1/CCR1/SEZ6/TNFAIP8L1/CNP/MAP3K8/ADM/IL31RA/OR2A14/CRABP1/MIB2/MPP7/APCDD1/CTGF/SGOL1/PPM1L/CYLD/ADRB3/DDB1/ZNF366/BHLHA15/NLRP6/DLG2/DMBT1/ABAT/DRD4/DTNA/AGXT/EFNA2/EGFR/EGR3/EIF4G1/A2M/TMEM17/EPHA1/EPHA3/EPHB4/ESR1/SPATA13/FCGR2A/FGA/FGF10/FHIT/RASA3/ACIN1/FOXL1/FOXC2/FOXO1/AKR1B1/SPG20/NFASC/FLNB/RHOBTB2/NUP210/NEDD4L/PSD3/LARP1/PPP1R13B/PUM2/ARHGEF18/MAPK8IP2/TSSK2/MTOR/SLC37A4/GABBR1/RASGEF1C/ALS2CL/PNKD/TENM4/ACOT11/RGS22/PLEK2/ADGRF1/RPS6KC1/GJA3/FGF22/NPTN/GJB2/SDCBP2/PDE7B/DKK3/CYTH4/GLS2/GPR162/BMP10/GNAS/GPR26/GPER1/FFAR2/GRB10/GRIK4/DNAJC15/GSTP1/GUCY1A3/NME7/GPR132/ANXA2/SOX8/KCNIP2/NRG1/ANXA6/HK1/HLA-B/HLA-DOA/HLA-DPA1/HLA-E/HLA-F/NR4A1/HPCA/APBA2/HOXD3/HRH1/HSP90AA1/HSP90AB1/HTR3A/HTR5A/RSPO2/IGF1/IGF2/CYR61/GPR142/IL1R1/IL1RN/IL6/IL10RA/AQP2/IL11RA/IL12RB2/IL15RA/INHBA/IRF1/ISL1/ITGA7/ITGB2/ITGB7/JUP/CD82/KCNH2/KCNJ8/KCNJ9/KCNMB1/KDR/KRT15/HES5/STMN1/OR2A5/LCK/LCP1/LDLR/ARHGDIA/LGALS9/LHCGR/LLGL1/LMNA/RAB19/LTB/LTBP1/SMAD3/MC2R/MCC/MAP3K1/MFNG/SCGB2A1/MITF/ASGR1/MOV10/MPZ/PLEKHG7/NUDT1/NEDD9/ATP1A2/NFATC3/NMBR/NOV/NPPC/NRAS/NTF3/OAS2/OPRL1/OR2C1/OR3A2/P2RY6/IL21R/PALM/ARHGEF3/PARK2/SPOCK3/LEF1/DDX47/ANGPT4/PDE4C/PDE7A/PDE6B/PGAM2/PIK3CG/PITX2/PKHD1/PLA2G2A/PRKAG3/PML/GPR84/IL20RB/PNLIP/TLR9/TREM1/CYTL1/POMC/RIN2/ZDHHC13/APBB1IP/PPP1CB/PPP1CC/ARHGEF10L/MOB1A/SMPD3/CHRNA9/SYBU/LIMS2/VAC14/PRKAR1B/IFT122/MCTP2/LMBRD1/PAG1/PRKD1/WSB2/MAPK3/MAP2K2/PRKRIR/HTRA1/CDC42SE1/PSMB4/PAK6/RGMA/LPAR5/PSMD7/PTGFR/PLEKHG5/TENM2/CCAR2/PTPRE/PXN/RASGRF2/RGR/RGS12/RIT2/S100A4/S100A6/BGLAP/SCT/CCL11/CCL17/NOD2/STRA6/SFRP2/CXCR5/ARHGAP9/SGK1/MICAL1/TMEM237/VPS33A/BMP4/SLC6A12/SLC8A1/BMPR1B/BOK/SOX9/STAT2/STK3/STK10/BST2/VAMP2/ZEB1/TEAD3/TGM2/TLE3/TLR5/TNFAIP3/TNFRSF1A/TNXB/TRAF1/TRAF5/TWIST1/CCR2/TNFRSF4/UPP1/WNT10B/YWHAG/ZAP70/CA7/CACNA1E/CACNB2/PAX8/CXCR4/FZD5/RAB7A/CARD14/BCL2L14/TMEM204/NLRX1/GPR157/RAB11FIP1/CPEB4/C6orf25/CALR/UNC93B1/CAPS/COLQ/HIST1H3A/ANTXR1/SLA2/CMAHP/NR0B2/CASQ1/IL1F10/TRIM63/RAE1/IFITM1/CDK10/RUNX1/TP63/RUNX3/IRS2/CRADD/FADD/TNFRSF11A/ALDH1A2/SPHK1/SKAP2/STBD1/LIMD1/TSPAN18/CCRL2/PRC1/STARD13/PIAS2/MAP3K6/SYT7/RSAD2/AURKB/DAPL1/CD8A/TRIP10/ADIPOQ/ARHGAP29/LY86/RAB3D/SMAD5-AS1/RAB36/ARHGEF10/NUP93/RAPGEF2/ULK2/CD79A/TELO2/IQSEC1/FGF19/NR1H4	390
GO:0009605	response to external stimulus	187/901	2366/17046	4.02E-09	1.04E-06	8.19E-07	CDH13/SPON2/TBR1/GJB6/HNRNPUL1/CHGA/CHI3L1/CCR1/CNP/COL9A3/COL11A1/ADM/CPS1/ADRB3/CYP11A1/BHLHA15/COCH/NLRP6/DMBT1/DNMT3A/EFNA2/EGFR/EGR3/A2M/ANKRD23/UNC13D/EPHA1/EPHA3/EPHB4/F11/FGA/FGF10/RASA3/SBNO2/ACIN1/FOXO1/NFASC/LARP1/PUM2/MTOR/SLC37A4/FBXL21/GATM/FGF22/GPER1/FFAR2/DNAJC15/GSTP1/GUCY1A3/ANXA2/SERPIND1/NRG1/HK1/HLA-B/HLA-E/HMGA1/NR4A1/HRH1/HSP90AA1/HSP90AB1/CYR61/IL1R1/IL1RN/IL6/IL10RA/AQP2/IL12RB2/IL16/IRF1/ISL1/ITGB2/JUP/KCNJ8/KDR/ACAT1/KRT15/STMN1/LCK/LDLR/LGALS9/SMAD3/MAP3K1/ASGR1/NUDT1/ATP1A2/NOV/NRAS/NTF3/OAS2/PARK2/LEF1/SIRT6/PDE6B/ATP8A2/PIK3CG/PITX2/PKM/PLA2G2A/PRKAG3/PML/IL20RB/PNLIP/TLR9/TREM1/POMC/PON1/PPP1CB/PPP1CC/CHRNA9/PARVA/PRKD1/MAPK3/MAP2K2/PROC/MASP1/HTRA1/SLAMF8/PSMB4/ARNTL2/RGMA/TRPC7/PSMD7/PTGFR/PLEKHG5/TENM2/CREBZF/ACTA2/RASGRF2/RGR/DEFB134/BGLAP/CCL11/CCL17/NPAS3/NOD2/STRA6/SFRP2/CXCR5/VPS33A/BMP4/SLC8A1/BMPR1B/SLIT1/SOX9/BPI/STAT2/BST2/TIMP3/TLR5/TNFAIP3/TNFRSF1A/TRPC4/TRPC6/CCR2/UPP1/CA7/CACNB2/CXCR4/FZD5/RAB7A/NLRX1/ZC3H12A/CPEB4/CALR/UNC93B1/CAPS/HIST1H3A/HOPX/IFITM1/RUNX3/IRS2/CRADD/FADD/TNFRSF11A/ALDH1A2/STBD1/CCRL2/RSAD2/DAPL1/CD8A/ADIPOQ/LY86/NUP93/RAPGEF2/ULK2/FGF19/NR1H4	187
GO:0030154	cell differentiation	254/901	3469/17046	4.61E-09	1.13E-06	8.88E-07	ABI1/CDH3/FARP1/CDKN1C/SPEG/SPON2/ZBTB18/TACC2/CELF1/TBR1/LECT1/GPRIN1/CLN5/FRMD6/CCR1/SEZ6/CNP/COL9A3/COL11A1/SCLT1/ZFP42/ADM/IL31RA/CPS1/FAM101A/APCDD1/CSTA/CTGF/SMYD1/CYLD/ADRB3/ESCO2/CYP11A1/BHLHA15/DMBT1/DNMT3A/EEF2/EFNA2/EGFR/EGR3/EIF4G1/A2M/ELK4/EML1/UNC13D/EPHA1/EPHA3/EPHB4/ESR1/FGA/FGF10/RASA3/BTBD3/SBNO2/ACIN1/FOXL1/FOXC2/FOXO1/EXPH5/SPG20/NFASC/EPB41L3/FLNB/FLOT2/NEDD4L/SYNE1/PSD3/MAPK8IP2/TSSK2/MTOR/DFNB31/TENM4/LCE2B/GAPDHS/FGF22/NPTN/BMP10/GNAS/GPER1/FFAR2/FLVCR1/TMOD4/ANXA2/SOX8/KCNIP2/NRG1/HLA-B/HLA-DOA/ANXA13/HLX/NR4A1/HOXB3/HOXD3/HSP90AA1/HSP90AB1/ID3/RSPO2/FMN1/BARHL2/IGF1/IGF2/CYR61/LCE1C/LCE1D/LCE2D/IL6/FOXK2/INHBA/IRF1/ISL1/ITGA7/ITGB2/ITGB7/IVL/JUP/HILS1/KDR/INSC/HES5/LAMA3/STMN1/LCK/ARHGDIA/LGALS9/LLGL1/LMNA/SMAD3/MEF2D/MAP3K1/MEOX1/MFI2/MITF/LHX8/MYL2/NEU1/NFATC3/NHLH2/NOV/NPPC/NRAS/NTF3/ATP5B/PALM/PARK2/LEF1/CEND1/SIRT6/ATP8A2/PITX2/PLA2G2A/PLAGL1/PML/CYTL1/SSH1/MOV10L1/ROBO4/MXRA8/HERC6/PPP1CC/PIWIL2/ELP3/SMPD3/PARVA/PRKD1/BIN3/MAPK3/MAP2K2/MRAP/HTRA1/PSMB4/RGMA/TRPC7/PSMD7/TENM2/RDH14/PXN/ACTA2/RASGRF2/S100A4/S100A6/BGLAP/CCL17/SFRP2/SGK1/VPS33A/BMP4/SLC8A1/BMPR1B/SLIT1/BOK/SOX9/STK3/SUPT6H/TAF4B/TCEA1/ZEB1/ACTC1/TEAD3/TCHH/TNFRSF1A/TRPC4/TRPC6/TWIST1/UCP1/WNT10B/YWHAG/ZAP70/CACNB2/PAX8/CXCR4/FZD5/PPDPF/LST1/TMEM204/ZC3H12A/C6orf25/COL18A1/CALR/SLIRP/CAST/SCRT1/SPATA16/ANTXR1/BFSP2/CASQ1/HOPX/PARD6B/LOXL3/CBX2/IFITM1/GAS7/SCIN/RUNX1/TP63/RUNX3/IRS2/ACTN1/FADD/TNFRSF11A/ALDH1A2/LIMD1/PIAS2/CBFA2T2/RSAD2/DAPL1/CD8A/ADIPOQ/H2AFY/MTL5/ARHGEF10/RAPGEF2/ULK2/CD79A/FGF19	254

GO:0044700	single organism signaling	378/901	5624/17046	4.74E-09	1.13E-06	8.88E-07	AKT3/ABI1/CDH3/TANK/TSPAN5/CDH13/FARP1/KLRG1/RCAN2/KCNMB2/CDKN1C/TCIRG1/MRVI1/TRDN/PDPN/HCST/NPFFR2/ADCY3/LECT1/ESM1/CHI3L1/ERLIN2/EGLN2/PTH2/PDAP1/CHRNA1/CHRNA2/CHRNA5/SORCS1/CIDEA/PANX3/AP3S1/CCR1/SEZ6/TNFAIP8L1/CNP/MAP3K8/ADM/IL31RA/OR2A14/CRABP1/MIB2/MPP7/APCDD1/CTGF/SGOL1/PPM1L/CYLD/ADRB3/DDB1/ZNF366/BHLHA15/NLRP6/DLG2/DMBT1/ABAT/DRD4/DTNA/ECE1/AGXT/EFNA2/EGFR/EGR3/EIF4G1/A2M/TMEM17/EPHA1/EPHA3/EPHB4/ESR1/SPATA13/FCGR2A/FGA/FGF10/FHIT/RASA3/FOXL1/FOXC2/FOXO1/AKR1B1/SPG20/NFASC/FLNB/RHOBTB2/NUP210/NEDD4L/PSD3/LARP1/PPP1R13B/PUM2/ARHGEF18/MAPK8IP2/TSSK2/MTOR/GABBR1/RASGEF1C/ALS2CL/PNKD/TENM4/ACOT11/RGS22/PLEK2/ADGRF1/RPS6KC1/GJA3/FGF22/NPTN/GJB2/SDCBP2/PDE7B/DKK3/CYTH4/GLS2/GPR162/BMP10/GNAS/GPR26/GPER1/FFAR2/GRB10/GRIK4/GSTP1/GUCY1A3/NME7/GPR132/ANXA2/SOX8/KCNIP2/NRG1/ANXA6/HLA-B/HLA-DOA/HLA-DPA1/HLA-E/HLA-F/NR4A1/HPCA/APBA2/HOXD3/HRH1/HSP90AA1/HSP90AB1/HTR3A/HTR5A/RSPO2/IGF1/IGF2/CYR61/GPR142/IL1R1/IL1RN/IL6/IL10RA/IL11RA/IL12RB2/IL15RA/INHBA/IRF1/ISL1/ITGA7/ITGB2/ITGB7/JUP/CD82/KCNH2/KCNJ8/KCNJ9/KCNMB1/KDR/HES5/STMN1/OR2A5/LCK/LCP1/LDLR/ARHGDIA/LGALS9/LHCGR/LLGL1/LMNA/RAB19/LTB/LTBP1/SMAD3/MC2R/MCC/MAP3K1/MFNG/SCGB2A1/MITF/MOV10/MPZ/PLEKHG7/NEDD9/ATP1A2/NFATC3/NMBR/NOV/NPPC/NRAS/NTF3/OAS2/OPRL1/OR2C1/OR3A2/P2RY6/IL21R/PALM/ARHGEF3/PARK2/SPOCK3/LEF1/DDX47/ANGPT4/PDE4C/PDE7A/PDE6B/PGAM2/PIK3CG/PITX2/PKHD1/PLA2G2A/PRKAG3/PML/GPR84/IL20RB/PNLIP/TLR9/TREM1/CYTL1/POMC/RIN2/ZDHHC13/APBB1IP/PPP1CB/PPP1CC/ARHGEF10L/MOB1A/SMPD3/CHRNA9/SYBU/LIMS2/VAC14/PRKAR1B/IFT122/MCTP2/LMBRD1/PAG1/PRKD1/WSB2/MAPK3/MAP2K2/PRKRIR/HTRA1/CDC42SE1/PSMB4/PAK6/RGMA/LPAR5/PSMD7/PTGFR/PLEKHG5/TENM2/CCAR2/PTPRE/PXN/RASGRF2/RGR/RGS12/RIT2/S100A4/S100A6/SCT/CCL11/CCL17/NOD2/STRA6/SFRP2/CXCR5/ARHGAP9/SGK1/MICAL1/TMEM237/BMP4/SLC6A12/SLC8A1/BMPR1B/BOK/SOX9/STAT2/STK3/STK10/BST2/VAMP2/ZEB1/TEAD3/TGM2/TLE3/TLR5/TNFAIP3/TNFRSF1A/TNXB/TRAF1/TRAF5/TWIST1/CCR2/TNFRSF4/WNT10B/YWHAG/ZAP70/CA7/CACNA1E/CACNB2/PAX8/CXCR4/FZD5/RAB7A/CARD14/BCL2L14/TMEM204/NLRX1/GPR157/RAB11FIP1/CPEB4/C6orf25/CALR/UNC93B1/CAPS/COLQ/HIST1H3A/ANTXR1/SLA2/CMAHP/NR0B2/CASQ1/IL1F10/TRIM63/RAE1/IFITM1/CDK10/RUNX1/TP63/RUNX3/IRS2/CRADD/FADD/TNFRSF11A/ALDH1A2/SPHK1/SKAP2/LIMD1/TSPAN18/CCRL2/PRC1/STARD13/PIAS2/MAP3K6/SYT7/RSAD2/AURKB/DAPL1/CD8A/TRIP10/ADIPOQ/ARHGAP29/LY86/RAB3D/SMAD5-AS1/RAB36/ARHGEF10/NUP93/RAPGEF2/ULK2/CD79A/TELO2/IQSEC1/FGF19/NR1H4	378
GO:0023052	signaling	378/901	5629/17046	5.32E-09	1.22E-06	9.59E-07	AKT3/ABI1/CDH3/TANK/TSPAN5/CDH13/FARP1/KLRG1/RCAN2/KCNMB2/CDKN1C/TCIRG1/MRVI1/TRDN/PDPN/HCST/NPFFR2/ADCY3/LECT1/ESM1/CHI3L1/ERLIN2/EGLN2/PTH2/PDAP1/CHRNA1/CHRNA2/CHRNA5/SORCS1/CIDEA/PANX3/AP3S1/CCR1/SEZ6/TNFAIP8L1/CNP/MAP3K8/ADM/IL31RA/OR2A14/CRABP1/MIB2/MPP7/APCDD1/CTGF/SGOL1/PPM1L/CYLD/ADRB3/DDB1/ZNF366/BHLHA15/NLRP6/DLG2/DMBT1/ABAT/DRD4/DTNA/ECE1/AGXT/EFNA2/EGFR/EGR3/EIF4G1/A2M/TMEM17/EPHA1/EPHA3/EPHB4/ESR1/SPATA13/FCGR2A/FGA/FGF10/FHIT/RASA3/FOXL1/FOXC2/FOXO1/AKR1B1/SPG20/NFASC/FLNB/RHOBTB2/NUP210/NEDD4L/PSD3/LARP1/PPP1R13B/PUM2/ARHGEF18/MAPK8IP2/TSSK2/MTOR/GABBR1/RASGEF1C/ALS2CL/PNKD/TENM4/ACOT11/RGS22/PLEK2/ADGRF1/RPS6KC1/GJA3/FGF22/NPTN/GJB2/SDCBP2/PDE7B/DKK3/CYTH4/GLS2/GPR162/BMP10/GNAS/GPR26/GPER1/FFAR2/GRB10/GRIK4/GSTP1/GUCY1A3/NME7/GPR132/ANXA2/SOX8/KCNIP2/NRG1/ANXA6/HLA-B/HLA-DOA/HLA-DPA1/HLA-E/HLA-F/NR4A1/HPCA/APBA2/HOXD3/HRH1/HSP90AA1/HSP90AB1/HTR3A/HTR5A/RSPO2/IGF1/IGF2/CYR61/GPR142/IL1R1/IL1RN/IL6/IL10RA/IL11RA/IL12RB2/IL15RA/INHBA/IRF1/ISL1/ITGA7/ITGB2/ITGB7/JUP/CD82/KCNH2/KCNJ8/KCNJ9/KCNMB1/KDR/HES5/STMN1/OR2A5/LCK/LCP1/LDLR/ARHGDIA/LGALS9/LHCGR/LLGL1/LMNA/RAB19/LTB/LTBP1/SMAD3/MC2R/MCC/MAP3K1/MFNG/SCGB2A1/MITF/MOV10/MPZ/PLEKHG7/NEDD9/ATP1A2/NFATC3/NMBR/NOV/NPPC/NRAS/NTF3/OAS2/OPRL1/OR2C1/OR3A2/P2RY6/IL21R/PALM/ARHGEF3/PARK2/SPOCK3/LEF1/DDX47/ANGPT4/PDE4C/PDE7A/PDE6B/PGAM2/PIK3CG/PITX2/PKHD1/PLA2G2A/PRKAG3/PML/GPR84/IL20RB/PNLIP/TLR9/TREM1/CYTL1/POMC/RIN2/ZDHHC13/APBB1IP/PPP1CB/PPP1CC/ARHGEF10L/MOB1A/SMPD3/CHRNA9/SYBU/LIMS2/VAC14/PRKAR1B/IFT122/MCTP2/LMBRD1/PAG1/PRKD1/WSB2/MAPK3/MAP2K2/PRKRIR/HTRA1/CDC42SE1/PSMB4/PAK6/RGMA/LPAR5/PSMD7/PTGFR/PLEKHG5/TENM2/CCAR2/PTPRE/PXN/RASGRF2/RGR/RGS12/RIT2/S100A4/S100A6/SCT/CCL11/CCL17/NOD2/STRA6/SFRP2/CXCR5/ARHGAP9/SGK1/MICAL1/TMEM237/BMP4/SLC6A12/SLC8A1/BMPR1B/BOK/SOX9/STAT2/STK3/STK10/BST2/VAMP2/ZEB1/TEAD3/TGM2/TLE3/TLR5/TNFAIP3/TNFRSF1A/TNXB/TRAF1/TRAF5/TWIST1/CCR2/TNFRSF4/WNT10B/YWHAG/ZAP70/CA7/CACNA1E/CACNB2/PAX8/CXCR4/FZD5/RAB7A/CARD14/BCL2L14/TMEM204/NLRX1/GPR157/RAB11FIP1/CPEB4/C6orf25/CALR/UNC93B1/CAPS/COLQ/HIST1H3A/ANTXR1/SLA2/CMAHP/NR0B2/CASQ1/IL1F10/TRIM63/RAE1/IFITM1/CDK10/RUNX1/TP63/RUNX3/IRS2/CRADD/FADD/TNFRSF11A/ALDH1A2/SPHK1/SKAP2/LIMD1/TSPAN18/CCRL2/PRC1/STARD13/PIAS2/MAP3K6/SYT7/RSAD2/AURKB/DAPL1/CD8A/TRIP10/ADIPOQ/ARHGAP29/LY86/RAB3D/SMAD5-AS1/RAB36/ARHGEF10/NUP93/RAPGEF2/ULK2/CD79A/TELO2/IQSEC1/FGF19/NR1H4	378
GO:0061448	connective tissue development	35/901	224/17046	7.76E-09	1.71E-06	1.35E-06	LECT1/CHI3L1/COL11A1/COMP/FAM101A/CTGF/FOXC2/SPG20/BMP10/GNAS/SOX8/HOXB3/HOXC4/HOXD3/RSPO2/IGF1/CYR61/ACAT1/HES5/SMAD3/MEF2D/NOV/NPPC/CYTL1/CSGALNACT1/MAPK3/ACTA2/SFRP2/BMP4/BMPR1B/SOX9/ZEB1/WNT10B/SCIN/RUNX3	35
GO:0065008	regulation of biological quality	236/901	3225/17046	2.26E-08	4.80E-06	3.77E-06	CDH3/KCNMB2/TCIRG1/MRVI1/TRDN/ZBTB18/PDPN/GJB6/ADCY3/CHGA/EGLN2/CHRNA1/CHRNA2/CHRNA5/CIDEA/CLN5/CCR1/SLC51B/C15orf27/ZG16B/SEZ6/CNP/ADM/IL31RA/CPS1/CRABP1/CTGF/ADRB3/CYP11A1/FITM1/DDB1/BHLHA15/COCH/NLRP6/DIO3/ABAT/DRD4/ECE1/A2M/ESR1/F11/FGA/FGF10/ACIN1/FOXC2/FOXO1/AKR1B1/SPG20/EPB41L3/FLNB/FLOT2/ATP11A/NEDD4L/SYNE1/ARHGEF18/MORC3/MAPK8IP2/MTOR/SLC37A4/DFNB31/STEAP2/GAS2/PABPC1/NPTN/AMPD2/DKK3/GLS2/AMPD3/GNAS/GPR26/GPER1/FFAR2/FLVCR1/SCG3/GSTP1/BRF1/TMOD4/GUCY1A3/ANXA2/SERPIND1/SOX8/KCNIP2/ANXA6/HK1/ACACB/HRH1/ACADL/HSP90AB1/HTR3A/FMN1/BARHL2/NME9/IGF1/IGF2/IL1R1/IL1RN/IL6/AQP2/AQP5/INHBA/IRF1/AQP9/ISL1/ITGA7/ITGB2/JUP/ATP9B/KCNH2/KCNJ8/KCNMB1/KDR/AMIGO3/LCK/LDLR/LGALS9/LTBP1/SMAD3/MAP3K1/MFI2/MYL2/NUBP1/ATP1A2/NOV/NPPC/NRAS/OPRL1/ATP5B/ANO7/PALM/PARK2/LEF1/PRR16/ANGPT4/PDE4C/SIRT6/PDE6B/ATP8A2/PIK3CG/PKHD1/PML/IL20RB/TLR9/TREM1/CYTL1/POMC/SSH1/APBB1IP/FBLIM1/PALMD/SMPD3/SLC30A10/CHRNA9/SYBU/PARVA/PRKAR1B/PRKD1/MAPK3/PROC/SLAMF8/CDC42SE1/TRPC7/ACTR3B/ERMN/RDH14/METTL14/CCAR2/PXN/ACTA2/RASGRF2/RFC2/TRIM27/RPA3/BGLAP/SCT/CCL11/ABHD4/NOD2/STRA6/SGK1/BMP4/SLC4A1/SLC6A12/SLC8A1/SLC9A3/SLIT1/SOX9/STK3/VAMP2/TCEA1/TERF1/TGM2/TNFAIP3/TRPC4/TRPC6/CCR2/VARS/WNT10B/YWHAG/CA7/CACNA1E/PAX8/CXCR4/RAB7A/LST1/ZC3H14/ZC3H12A/RAB11FIP1/C6orf25/CALR/COLQ/CAPZB/SH3BGRL3/HIST1H3A/ATP13A4/NR0B2/CASQ1/TTBK1/GAS7/SCIN/TP63/IRS2/ACTN1/FADD/TNFRSF11A/ALDH1A2/LIMD1/SYT7/LDB2/ESAM/SLC16A3/SMDT1/ADIPOQ/RAB3D/MTL5/RAPGEF2/ULK2	236

GO:0007165	signal transduction	349/901	5179/17046	2.47E-08	5.07E-06	3.99E-06	AKT3/ABI1/CDH3/TANK/TSPAN5/CDH13/FARP1/KLRG1/RCAN2/CDKN1C/TCIRG1/MRVI1/PDPN/HCST/NPFFR2/ADCY3/LECT1/ESM1/CHI3L1/ERLIN2/EGLN2/PTH2/PDAP1/CHRNA1/CHRNA2/CHRNA5/SORCS1/CIDEA/AP3S1/CCR1/SEZ6/TNFAIP8L1/MAP3K8/ADM/IL31RA/OR2A14/CRABP1/MIB2/MPP7/APCDD1/CTGF/SGOL1/PPM1L/CYLD/ADRB3/DDB1/ZNF366/BHLHA15/NLRP6/DMBT1/DRD4/DTNA/AGXT/EFNA2/EGFR/EIF4G1/A2M/TMEM17/EPHA1/EPHA3/EPHB4/ESR1/SPATA13/FCGR2A/FGA/FGF10/FHIT/RASA3/FOXL1/FOXC2/FOXO1/AKR1B1/SPG20/FLNB/RHOBTB2/NUP210/NEDD4L/PSD3/LARP1/PPP1R13B/PUM2/ARHGEF18/MAPK8IP2/TSSK2/MTOR/GABBR1/RASGEF1C/ALS2CL/TENM4/ACOT11/RGS22/PLEK2/ADGRF1/RPS6KC1/FGF22/NPTN/SDCBP2/PDE7B/DKK3/CYTH4/GPR162/BMP10/GNAS/GPR26/GPER1/FFAR2/GRB10/GRIK4/GSTP1/GUCY1A3/NME7/GPR132/ANXA2/SOX8/KCNIP2/NRG1/ANXA6/HLA-B/HLA-DOA/HLA-DPA1/HLA-E/HLA-F/NR4A1/HPCA/HOXD3/HRH1/HSP90AA1/HSP90AB1/HTR3A/HTR5A/RSPO2/IGF1/IGF2/CYR61/GPR142/IL1R1/IL1RN/IL6/IL10RA/IL11RA/IL12RB2/IL15RA/INHBA/IRF1/ISL1/ITGA7/ITGB2/ITGB7/JUP/CD82/KCNH2/KDR/HES5/STMN1/OR2A5/LCK/LCP1/LDLR/ARHGDIA/LGALS9/LHCGR/LLGL1/LMNA/RAB19/LTB/LTBP1/SMAD3/MC2R/MCC/MAP3K1/MFNG/SCGB2A1/MITF/MOV10/PLEKHG7/NEDD9/ATP1A2/NFATC3/NMBR/NOV/NPPC/NRAS/NTF3/OAS2/OPRL1/OR2C1/OR3A2/P2RY6/IL21R/PALM/ARHGEF3/PARK2/SPOCK3/LEF1/DDX47/ANGPT4/PDE4C/PDE7A/PDE6B/PGAM2/PIK3CG/PITX2/PKHD1/PLA2G2A/PRKAG3/PML/GPR84/IL20RB/PNLIP/TLR9/TREM1/CYTL1/POMC/RIN2/ZDHHC13/APBB1IP/PPP1CB/PPP1CC/ARHGEF10L/MOB1A/CHRNA9/LIMS2/VAC14/PRKAR1B/IFT122/MCTP2/LMBRD1/PAG1/PRKD1/WSB2/MAPK3/MAP2K2/PRKRIR/HTRA1/CDC42SE1/PSMB4/PAK6/RGMA/LPAR5/PSMD7/PTGFR/PLEKHG5/TENM2/CCAR2/PTPRE/PXN/RASGRF2/RGR/RGS12/RIT2/S100A4/S100A6/CCL11/CCL17/NOD2/STRA6/SFRP2/CXCR5/ARHGAP9/SGK1/MICAL1/TMEM237/BMP4/SLC8A1/BMPR1B/BOK/SOX9/STAT2/STK3/STK10/BST2/ZEB1/TEAD3/TGM2/TLE3/TLR5/TNFAIP3/TNFRSF1A/TNXB/TRAF1/TRAF5/TWIST1/CCR2/TNFRSF4/WNT10B/YWHAG/ZAP70/PAX8/CXCR4/FZD5/RAB7A/CARD14/BCL2L14/TMEM204/NLRX1/GPR157/CPEB4/C6orf25/CALR/UNC93B1/CAPS/HIST1H3A/ANTXR1/SLA2/CMAHP/NR0B2/CASQ1/IL1F10/TRIM63/RAE1/IFITM1/CDK10/RUNX1/TP63/RUNX3/IRS2/CRADD/FADD/TNFRSF11A/ALDH1A2/SPHK1/SKAP2/LIMD1/TSPAN18/CCRL2/PRC1/STARD13/PIAS2/MAP3K6/RSAD2/AURKB/DAPL1/CD8A/TRIP10/ADIPOQ/ARHGAP29/LY86/RAB3D/SMAD5-AS1/RAB36/ARHGEF10/NUP93/RAPGEF2/ULK2/CD79A/TELO2/IQSEC1/FGF19/NR1H4	349
GO:0022610	biological adhesion	118/901	1348/17046	2.55E-08	5.07E-06	3.99E-06	CDH3/GNE/CDH9/CDH12/CDH13/SPON2/PDPN/PKP3/FAT3/CCR1/COMP/MAP3K8/EGFLAM/CSTA/CTGF/CYLD/DDOST/DSG3/EGFR/EGR3/UNC13D/EPHA1/EPHA3/EPHB4/FAT2/FGA/FOXC2/NFASC/FLOT2/MTOR/NPTN/CYTH4/BMP10/GNAS/IZUMO1/HAS1/NRG1/HLA-DOA/HLA-DPA1/HLA-E/HLX/HOXD3/HSP90AB1/ZC3H12D/COL28A1/FMN1/IGF1/IGF2/CYR61/IL1RN/IL6/IRF1/ITGA7/ITGB2/ITGB7/JUP/KDR/AMIGO3/HES5/CDHR4/LAMA3/LCK/LCP1/ARHGDIA/LGALS9/LPP/SMAD3/MFI2/NEDD9/NFATC3/NOV/ATP5B/LEF1/PIK3CG/PKHD1/PML/IL20RB/APBB1IP/FBLIM1/PPP1CB/LIMS2/PARVA/PAG1/PCDHGC4/PCDHGB7/PCDHGB3/PCDHGA11/SLURP1/TENM2/PXN/BGLAP/CCL11/PARVG/NOD2/TINAGL1/SFRP2/PCDH20/BMP4/SOX9/STK10/ZEB1/TGM2/TNXB/CCR2/TNFRSF4/ZAP70/FZD5/COL18A1/CALR/ANTXR1/SLA2/ACTN1/FADD/ESAM/RSAD2/IL32/CD8A/ADIPOQ	118
GO:0009888	tissue development	140/901	1684/17046	2.78E-08	5.35E-06	4.21E-06	ABI1/CDH3/SPEG/ZBTB18/DMRT2/LECT1/CHI3L1/CHRNA1/FRMD6/CCR1/COL11A1/COMP/ADM/CPS1/ZNF358/FAM101A/APCDD1/CSTA/CTGF/SMYD1/CYP11A1/DMBT1/DRD4/EGFR/EPHA3/ESR1/FGF10/SBNO2/FOXL1/FOXC2/EXPH5/SPG20/FLNB/FLOT2/VGLL2/TENM4/LCE2B/GATM/GJB2/BMP10/GNAS/SOX8/NRG1/HLX/HPCA/HOXB3/HOXC4/HOXD3/ID3/RSPO2/FMN1/BARHL2/IGF1/CYR61/LCE1C/LCE1D/LCE2D/IL6/INHBA/ISL1/ITGA7/ITGB2/IVL/KDR/ACAT1/KRT15/INSC/HES5/LAMA3/LMNA/SMAD3/MEF2D/MAP3K1/MEOX1/MEOX2/MITF/MYL2/NFATC3/NOV/NPPC/NRAS/NTF3/LEF1/SIRT6/PDE6B/ATP8A2/PITX2/PKM/PLAGL1/PML/RIPK4/CYTL1/BNC2/IFT122/CSGALNACT1/BIN3/MAPK3/MAP2K2/PXN/ACTA2/S100A4/BGLAP/CCL11/STRA6/SFRP2/GZF1/BMP4/SLC8A1/BMPR1B/SOX9/STK3/ZEB1/ACTC1/TGM2/TCHH/TIMP3/TNFRSF1A/TWIST1/WNT10B/PAX8/FZD5/COL18A1/CALR/BFSP2/CASQ1/HOPX/KDM2B/LOXL3/SCIN/RUNX1/TP63/RUNX3/ALDH1A2/LDB2/CBFA2T2/ADIPOQ/H2AFY/MICAL2/RAPGEF2/FGF19	140
GO:0007166	cell surface receptor signaling pathway	192/901	2511/17046	3.11E-08	5.79E-06	4.56E-06	ABI1/CDH3/TSPAN5/CDH13/KLRG1/RCAN2/CDKN1C/TCIRG1/ADCY3/LECT1/ESM1/CIDEA/AP3S1/CCR1/IL31RA/MIB2/APCDD1/CTGF/PPM1L/CYLD/DDB1/AGXT/EFNA2/EGFR/EIF4G1/TMEM17/EPHA1/EPHA3/EPHB4/FCGR2A/FGA/FGF10/RASA3/FOXL1/FOXC2/FOXO1/SPG20/FLNB/NUP210/NEDD4L/ARHGEF18/MAPK8IP2/MTOR/ADGRF1/FGF22/NPTN/DKK3/BMP10/GPER1/FFAR2/GRB10/GRIK4/GSTP1/NME7/NRG1/HLA-B/HLA-DPA1/HLA-E/HLA-F/NR4A1/HOXD3/HSP90AA1/HSP90AB1/RSPO2/IGF1/IGF2/CYR61/IL1R1/IL1RN/IL6/IL10RA/IL11RA/IL12RB2/IL15RA/INHBA/IRF1/ISL1/ITGA7/ITGB2/ITGB7/JUP/CD82/KDR/HES5/LCK/ARHGDIA/LLGL1/LMNA/LTB/LTBP1/SMAD3/MCC/MAP3K1/MFNG/MITF/MOV10/NEDD9/NFATC3/NOV/NPPC/NRAS/NTF3/OAS2/P2RY6/IL21R/ARHGEF3/PARK2/LEF1/DDX47/ANGPT4/PGAM2/PITX2/PRKAG3/PML/IL20RB/TLR9/PPP1CB/PPP1CC/LIMS2/PRKAR1B/IFT122/LMBRD1/PAG1/PRKD1/MAPK3/MAP2K2/HTRA1/PSMB4/RGMA/PSMD7/PLEKHG5/CCAR2/PTPRE/PXN/RASGRF2/RIT2/CCL11/CCL17/NOD2/SFRP2/CXCR5/TMEM237/BMP4/BMPR1B/BOK/SOX9/STAT2/STK3/BST2/ZEB1/TLE3/TNFAIP3/TNFRSF1A/TRAF1/CCR2/TNFRSF4/WNT10B/ZAP70/CXCR4/FZD5/RAB7A/CARD14/BCL2L14/TMEM204/GPR157/CPEB4/C6orf25/SLA2/CMAHP/NR0B2/IL1F10/RAE1/IFITM1/TP63/RUNX3/IRS2/CRADD/FADD/TNFRSF11A/SPHK1/LIMD1/TSPAN18/CCRL2/RSAD2/CD8A/ADIPOQ/LY86/NUP93/RAPGEF2/CD79A/FGF19/NR1H4	192
GO:0007155	cell adhesion	117/901	1343/17046	3.84E-08	6.94E-06	5.46E-06	CDH3/GNE/CDH9/CDH12/CDH13/SPON2/PDPN/PKP3/FAT3/CCR1/COMP/MAP3K8/EGFLAM/CSTA/CTGF/CYLD/DDOST/DSG3/EGFR/EGR3/UNC13D/EPHA1/EPHA3/EPHB4/FAT2/FGA/FOXC2/NFASC/FLOT2/MTOR/NPTN/CYTH4/BMP10/GNAS/IZUMO1/HAS1/NRG1/HLA-DOA/HLA-DPA1/HLA-E/HLX/HOXD3/ZC3H12D/COL28A1/FMN1/IGF1/IGF2/CYR61/IL1RN/IL6/IRF1/ITGA7/ITGB2/ITGB7/JUP/KDR/AMIGO3/HES5/CDHR4/LAMA3/LCK/LCP1/ARHGDIA/LGALS9/LPP/SMAD3/MFI2/NEDD9/NFATC3/NOV/ATP5B/LEF1/PIK3CG/PKHD1/PML/IL20RB/APBB1IP/FBLIM1/PPP1CB/LIMS2/PARVA/PAG1/PCDHGC4/PCDHGB7/PCDHGB3/PCDHGA11/SLURP1/TENM2/PXN/BGLAP/CCL11/PARVG/NOD2/TINAGL1/SFRP2/PCDH20/BMP4/SOX9/STK10/ZEB1/TGM2/TNXB/CCR2/TNFRSF4/ZAP70/FZD5/COL18A1/CALR/ANTXR1/SLA2/ACTN1/FADD/ESAM/RSAD2/IL32/CD8A/ADIPOQ	117

GO:0051179	localization	347/901	5173/17046	4.79E-08	8.39E-06	6.60E-06	ABI1/CDH3/TSPAN5/CDH13/KCNMB2/TCIRG1/TRDN/ABCA9/SPON2/COG5/TACC2/PDPN/TBR1/ADCY3/TMED10/SLC27A2/RER1/CHGA/CHI3L1/PKP3/EXOC3/CHRNA1/CHRNA2/CHRNA5/CIDEA/PANX3/RBP7/AP3S1/CLCA1/FRMD6/CCR1/SLC51B/C15orf27/SLC38A10/CNP/ADM/CRABP1/TRPM6/PARP4/MPP7/LDLRAD3/APCDD1/KLC3/CTGF/ABCC13/SH3D19/CYB561/CYLD/ESCO2/FITM1/TRPV3/DDOST/BHLHA15/NLRP6/DLG2/DMBT1/DNAH6/DNAH8/ABAT/DRD4/AGXT/EGFR/EGR3/A2M/UNC13D/SLC10A4/EPHA1/EPHA3/EPHB4/FAT2/SPATA13/FCGR2A/PHACTR1/FGA/FGF10/RASA3/VASH1/TRAK1/FOXC2/EXPH5/NFASC/EPB41L3/GGA3/FLNB/FLOT2/MLC1/TBC1D1/NUP210/ATP11A/NEDD4L/SYNE1/MORC3/MAPK8IP2/MTOR/SLC37A4/SAMM50/SEC31B/STEAP2/GAPDHS/SLC17A5/GJA3/GJB2/SDCBP2/GLS2/VPS4A/BMP10/GNAS/CRACR2B/PIGW/GPR26/GPER1/FFAR2/GRB10/FLVCR1/GRIK4/DNAJC15/SCG3/GSTP1/NME7/ANXA2/HAS1/SOX8/KCNIP2/NRG1/ANXA6/HK1/ANXA13/HLA-E/NR4A1/ACACB/HPCA/APBA2/HRH1/HSPA1L/HSP90AA1/HSP90AB1/HTR3A/BARHL2/IGF1/IGF2/CYR61/IL1RN/IL6/AQP2/IL16/AQP5/INHBA/AQP9/ISL1/ITGA7/ITGB2/ITGB7/JUP/ATP9B/KCNH2/KCNJ8/KCNJ9/KCNMB1/KDR/IPO5/INSC/TOMM20L/SLC6A17/LAMA3/STMN1/LCK/LCP1/LDLR/LGALS9/LLGL1/LMNA/RAB19/LTBP1/SMAD3/MCC/MAP3K1/MFI2/ASGR1/NUBP1/ATP1A2/NFATC3/NOV/NRAS/NTF3/OPRL1/SLC22A18/P2RY6/ATP5B/ANO7/PALM/PARK2/LEF1/CEND1/ANGPT4/PDE4C/PCYOX1/C11orf73/SIRT6/ATP8A2/PIK3CG/PITX2/PKHD1/LRP1B/PRKAG3/PML/FXYD6/SLCO1C1/PNLIP/TLR9/TREM1/POMC/PON1/RIN2/ZDHHC13/ROBO4/BANP/ELP3/DNAJC17/GOLPH3L/SLC47A1/SLC29A3/TRPV6/SMPD3/SLC30A10/CHRNA9/SYBU/PEX26/PARVA/PRKAR1B/IFT122/LMBRD1/PRKD1/BIN3/APOBR/MAPK3/MAP2K2/PROC/MRAP/TRPV5/MASP1/CDC42SE1/PAK6/TRPC7/PLEKHG5/FAM60A/RASGRF2/TRIM27/EXOC4/RPL8/RPL29/S100A6/SCT/CCL11/CCL17/ABHD4/NOD2/TINAGL1/STRA6/SFRP2/SGK1/VPS33A/BMP4/SLC4A1/ZG16/SLC6A12/SLC8A1/SLC9A3/SLC20A2/SLIT1/SOX9/SRP68/STK10/SUPT6H/BST2/VAMP2/TGM2/TRAPPC10/TNFAIP3/TNFRSF1A/TRPC4/TRPC6/TRPM2/PHLDA2/TWIST1/CCR2/TNFRSF4/UCP1/YWHAG/ZAP70/CA7/CACNA1E/PTP4A1/CACNB2/PAX8/CXCR4/FZD5/RAB7A/ZC3H12A/RAB11FIP1/COL18A1/CALR/UNC93B1/SLIRP/COLQ/SH3BGRL3/SCRT1/DYNLRB2/SLC25A18/MFSD7/ATP13A4/NR0B2/MON1A/CASQ1/PARD6B/LOXL3/MGARP/RAE1/SLC43A1/IFITM1/SCIN/SERPINA6/IRS2/ACTN1/FADD/TNFRSF11A/SPHK1/ENDOU/LIMD1/MAP7/SYT7/ESAM/SLC16A3/RSAD2/SMDT1/AURKB/REEP6/TRIP10/ADIPOQ/RAB3D/H2AFY/RAB36/NUP93/RAPGEF2/USP6NL/RABGAP1L/FGF19/NR1H4	347
GO:0071310	cellular response to organic substance	165/901	2092/17046	4.94E-08	8.41E-06	6.62E-06	CDH13/CDKN1C/TCIRG1/SPON2/GJB6/NPFFR2/ADCY3/LECT1/CHI3L1/CIDEA/AP3S1/CCR1/IL31RA/CPS1/CTGF/CYP11A1/BHLHA15/DNMT3A/EGFR/EGR3/EIF4G1/EPHA3/ESR1/FGA/FGF10/RASA3/SBNO2/FOXC2/FOXO1/AKR1B1/SPG20/FLNB/MLC1/NUP210/NEDD4L/ARHGEF18/MTOR/FGF22/NPTN/BMP10/GNAS/GPER1/FFAR2/GRB10/GSTP1/HAS1/NRG1/HLA-B/HLA-DPA1/HLA-E/HLA-F/NR4A1/HRH1/HSP90AB1/HTR3A/IGF2/CYR61/IL1R1/IL1RN/IL6/IL10RA/IL11RA/IL12RB2/IL15RA/INHBA/IRF1/AQP9/ISL1/JUP/KDR/IPO5/HES5/LCK/ARHGDIA/LGALS9/LHCGR/LMNA/LMO2/LTB/LTBP1/SMAD3/MAP3K1/MOV10/ATP1A2/NRAS/OAS2/P2RY6/IL21R/ARHGEF3/PARK2/LEF1/PIK3CG/PRKAG3/PML/IL20RB/TLR9/SSH1/PPP1CB/PPP1CC/SYBU/PRKAR1B/LMBRD1/PRKD1/MAPK3/MAP2K2/HTRA1/PSMB4/RGMA/PSMD7/PTGFR/PLEKHG5/PTPRE/PXN/RASGRF2/RIT2/BGLAP/CCL11/CCL17/NOD2/SFRP2/CXCR5/BMP4/SLC8A1/BMPR1B/SOX9/STAT2/BST2/VAMP2/ZEB1/TIMP3/TLR5/TNFAIP3/TNFRSF1A/TWIST1/CCR2/TNFRSF4/WNT10B/CACNA1E/PAX8/CXCR4/CARD14/TMEM204/ZC3H12A/CPEB4/CALR/NR0B2/IL1F10/TRIM63/MGARP/RAE1/IFITM1/RUNX1/IRS2/FADD/TNFRSF11A/ALDH1A2/SPHK1/CCRL2/RSAD2/ADIPOQ/LY86/NUP93/RAPGEF2/FGF19/NR1H4	165
GO:0048468	cell development	159/901	1998/17046	5.12E-08	8.48E-06	6.67E-06	ABI1/FARP1/CDKN1C/SPEG/SPON2/ZBTB18/CELF1/TBR1/LECT1/GPRIN1/CLN5/FRMD6/SEZ6/CNP/COL9A3/COL11A1/SCLT1/ZFP42/ADM/FAM101A/BHLHA15/EFNA2/EGFR/EIF4G1/UNC13D/EPHA1/EPHA3/EPHB4/ESR1/FGA/FGF10/RASA3/BTBD3/FOXC2/EXPH5/SPG20/NFASC/EPB41L3/FLNB/NEDD4L/MAPK8IP2/TSSK2/MTOR/DFNB31/TENM4/GAPDHS/FGF22/NPTN/BMP10/GPER1/FLVCR1/TMOD4/ANXA2/SOX8/KCNIP2/NRG1/HOXB3/HOXD3/HSP90AA1/HSP90AB1/FMN1/BARHL2/IGF1/CYR61/IL6/INHBA/ISL1/ITGB7/HILS1/KDR/HES5/STMN1/ARHGDIA/LLGL1/LMNA/SMAD3/MAP3K1/MFI2/LHX8/MYL2/NEU1/NFATC3/NPPC/NRAS/NTF3/PALM/PARK2/LEF1/SIRT6/ATP8A2/PITX2/PML/SSH1/MOV10L1/MXRA8/PIWIL2/PARVA/PRKD1/BIN3/MAPK3/MAP2K2/PSMB4/RGMA/TRPC7/PSMD7/TENM2/PXN/ACTA2/RASGRF2/S100A4/S100A6/BGLAP/SFRP2/SGK1/VPS33A/BMP4/SLC8A1/BMPR1B/SLIT1/SOX9/TAF4B/ZEB1/ACTC1/TEAD3/TNFRSF1A/TRPC4/TRPC6/TWIST1/WNT10B/YWHAG/CACNB2/PAX8/CXCR4/FZD5/LST1/C6orf25/COL18A1/CALR/SLIRP/SCRT1/ANTXR1/BFSP2/CASQ1/PARD6B/LOXL3/GAS7/RUNX1/TP63/RUNX3/IRS2/ACTN1/ALDH1A2/LIMD1/CBFA2T2/ADIPOQ/ARHGEF10/RAPGEF2/ULK2/FGF19	159
GO:0009628	response to abiotic stimulus	98/901	1071/17046	5.47E-08	8.61E-06	6.77E-06	GJB6/NPFFR2/CHI3L1/PSIP1/EGLN2/COL11A1/ADM/CTGF/ADRB3/CYP11A1/TRPV3/DDB1/RNF168/DNMT3A/ABAT/DRD4/EGFR/ANKRD23/FOXO1/AKR1B1/MLC1/NUP210/MTOR/ACOT11/FBXL21/GJA3/DNAJC2/HPCA/HRH1/HSPA1L/HSP90AA1/HSP90AB1/IGF1/IL1R1/IL6/AQP2/IRF1/AQP9/IVL/JUP/KCNJ8/LCK/LDLR/LMNA/SMAD3/MAP3K1/ATP1A2/NFATC3/NPPC/PALM/ANGPT4/C11orf73/PDE6B/ATP8A2/PKM/PML/PNLIP/PPP1CB/PPP1CC/CHRNA9/MAPK3/CCAR2/RGR/RPA3/BGLAP/SCT/CCL11/STRA6/SFRP2/BMP4/SLC8A1/SOX9/TCEB2/TIMP3/TLR5/TNFRSF1A/TRPC6/TWIST1/CACNA1E/CXCR4/CPEB4/COL18A1/CASQ1/TRIM63/MGARP/RAE1/KMO/TP63/CRADD/FADD/TNFRSF11A/LIMD1/MAP7/AURKB/RCSD1/ADIPOQ/N4BP1/NUP93	98

GO:0051716	cellular response to stimulus	415/901	6405/17046	5.49E-08	8.61E-06	6.77E-06	AKT3/ABI1/CDH3/TANK/TSPAN5/CDH13/FARP1/KLRG1/RCAN2/CDKN1C/TCIRG1/MRVI1/SPON2/PDPN/GJB6/HCST/NPFFR2/ADCY3/LECT1/ESM1/CHGA/CHI3L1/ERLIN2/EGLN2/TP53TG1/PTH2/PDAP1/CHRNA1/CHRNA2/CHRNA5/SORCS1/CIDEA/AP3S1/CCR1/SEZ6/TNFAIP8L1/MAP3K8/ADM/IL31RA/HUS1B/OR2A14/CPS1/CRABP1/MIB2/PARP4/MPP7/APCDD1/CTGF/SGOL1/PPM1L/CYLD/ADRB3/ESCO2/CYP11A1/DDB1/RNF168/ZNF366/BHLHA15/NLRP6/DMBT1/DNMT3A/DRD4/DTNA/AGXT/EFNA2/EGFR/EGR3/EIF4G1/A2M/TMEM17/EPHA1/EPHA3/EPHB4/ESR1/SPATA13/FCGR2A/FGA/FGF10/FHIT/RASA3/PPM1E/VASH1/SBNO2/ACIN1/FOXL1/FOXC2/FOXO1/AKR1B1/SPG20/FLNB/MLC1/RHOBTB2/NUP210/NEDD4L/PSD3/LARP1/PPP1R13B/PUM2/ARHGEF18/MAPK8IP2/TSSK2/MTOR/SLC37A4/GABBR1/RASGEF1C/ALS2CL/TENM4/ACOT11/RGS22/FBXO2/PLEK2/ADGRF1/RPS6KC1/DNAJC2/FGF22/NPTN/GJB2/SDCBP2/PDE7B/DKK3/CYTH4/GPR162/BMP10/GNAS/GPR26/GPER1/FFAR2/GRB10/GRIK4/DNAJC15/GSTP1/GUCY1A3/NME7/GPR132/ANXA2/HAS1/SOX8/KCNIP2/NRG1/ANXA6/HK1/HLA-B/HLA-DOA/HLA-DPA1/HLA-E/HLA-F/HMGA1/NR4A1/HPCA/HOXD3/HRH1/HSPA1L/HSP90AA1/HSP90AB1/HTR3A/HTR5A/RSPO2/IGF1/IGF2/CYR61/GPR142/LCE1D/IL1R1/IL1RN/IL6/IL10RA/AQP2/IL11RA/IL12RB2/IL15RA/IL16/INHBA/IRF1/AQP9/ISL1/ITGA7/ITGB2/ITGB7/JUP/CD82/KCNH2/KDR/IPO5/KRT15/HES5/STMN1/OR2A5/LCK/LCP1/LDLR/ARHGDIA/LGALS9/LHCGR/LLGL1/LMNA/LMO2/RAB19/LTB/LTBP1/SMAD3/MC2R/MCC/MAP3K1/MFNG/SCGB2A1/MITF/ASGR1/MOV10/PLEKHG7/MT1A/NUDT1/NEDD9/ATP1A2/NFATC3/NMBR/NOV/NPPC/NRAS/NTF3/OAS2/OPRL1/OR2C1/OR3A2/P2RY6/IL21R/PALM/ARHGEF3/PARK2/SPOCK3/LEF1/DDX47/ANGPT4/PDE4C/PDE7A/C11orf73/SIRT6/PDE6B/PGAM2/PIK3CG/PITX2/PKHD1/PLA2G2A/PRKAG3/PML/GPR84/IL20RB/PNLIP/TLR9/TREM1/CYTL1/POMC/SSH1/RIN2/ZDHHC13/APBB1IP/CYP2W1/PPP1CB/PPP1CC/ARHGEF10L/PRMT6/FANCI/MOB1A/WDR33/CHRNA9/SYBU/LIMS2/VAC14/PARVA/PRKAR1B/IFT122/MCTP2/LMBRD1/PAG1/PRKD1/WSB2/MAPK3/MAP2K2/PRKRIR/HTRA1/SLAMF8/CDC42SE1/PSMB4/PAK6/RGMA/LPAR5/PSMD7/PTGFR/PLEKHG5/TENM2/CCAR2/PTPRE/PXN/RASGRF2/RFC2/RGR/RGS12/RIT2/RPA3/S100A4/S100A6/BGLAP/CCL11/CCL17/NOD2/STRA6/SFRP2/CXCR5/ARHGAP9/SGK1/MICAL1/TMEM237/VPS33A/BMP4/SLC8A1/BMPR1B/BOK/SOX9/STAT2/STK3/STK10/BST2/VAMP2/TCEA1/TCEB2/ZEB1/TEAD3/TGM2/TIMP3/TLE3/TLR5/TNFAIP3/TNFRSF1A/TNXB/TRAF1/TRAF5/TRPC6/TWIST1/CCR2/TNFRSF4/UPP1/WNT10B/YWHAG/ZAP70/CA7/CACNA1E/PAX8/CXCR4/FZD5/RAB7A/CARD14/BCL2L14/TMEM204/NLRX1/GPR157/ZC3H12A/FAAP100/CPEB4/C6orf25/EEPD1/CALR/UNC93B1/CAPS/HIST1H3A/ANTXR1/SLA2/CMAHP/NR0B2/CASQ1/IL1F10/TRIM63/MGARP/RAE1/IFITM1/CDK10/RUNX1/TP63/RUNX3/IRS2/CRADD/FADD/TNFRSF11A/ALDH1A2/SPHK1/SKAP2/STBD1/LIMD1/TSPAN18/CCRL2/PRC1/STARD13/PIAS2/ZFAND2A/MAP3K6/RSAD2/AURKB/DAPL1/RCSD1/CD8A/TRIP10/ADIPOQ/ARHGAP29/LY86/RAB3D/SMAD5-AS1/RAB36/ARHGEF10/N4BP1/NUP93/RAPGEF2/ULK2/CD79A/TELO2/IQSEC1/FGF19/NR1H4	415
GO:0019932	second-messenger-mediated signaling	32/901	210/17046	6.21E-08	9.49E-06	7.47E-06	CDH13/RCAN2/MRVI1/ADCY3/ADM/BHLHA15/DRD4/EGFR/GNAS/GUCY1A3/NRG1/HPCA/HRH1/HTR5A/IGF1/KDR/LHCGR/ATP1A2/NFATC3/PDE7A/MCTP2/PTGFR/TENM2/SLC8A1/SOX9/ZAP70/CXCR4/SLA2/CASQ1/SPHK1/CD8A/RAPGEF2	32
GO:0051216	cartilage development	29/901	179/17046	6.61E-08	9.85E-06	7.75E-06	LECT1/CHI3L1/COL11A1/COMP/FAM101A/CTGF/BMP10/GNAS/HOXB3/HOXC4/HOXD3/RSPO2/CYR61/HES5/SMAD3/MEF2D/NOV/NPPC/CYTL1/CSGALNACT1/MAPK3/SFRP2/BMP4/BMPR1B/SOX9/ZEB1/WNT10B/SCIN/RUNX3	29
GO:0048870	cell motility	105/901	1187/17046	9.82E-08	1.39E-05	1.09E-05	CDH13/PDPN/ADCY3/CHGA/CCR1/APCDD1/CTGF/DNAH6/DNAH8/EGFR/EGR3/EPHA1/EPHA3/EPHB4/FAT2/SPATA13/PHACTR1/FGF10/VASH1/FOXC2/GAPDHS/BMP10/GPER1/FFAR2/HAS1/SOX8/NRG1/NR4A1/HRH1/BARHL2/IGF1/CYR61/IL1RN/IL6/IL16/ISL1/ITGA7/ITGB2/ITGB7/JUP/KDR/LAMA3/LCK/LCP1/LGALS9/LMNA/SMAD3/MCC/MAP3K1/NOV/NRAS/NTF3/P2RY6/ATP5B/LEF1/CEND1/ANGPT4/PIK3CG/PITX2/PML/TREM1/ROBO4/ELP3/PARVA/PRKD1/BIN3/MAP2K2/PROC/PAK6/PLEKHG5/FAM60A/CCL11/CCL17/NOD2/SFRP2/SGK1/BMP4/SLC8A1/SOX9/STK10/BST2/PHLDA2/TWIST1/CCR2/ZAP70/CACNA1E/PTP4A1/CXCR4/COL18A1/CALR/SLIRP/SH3BGRL3/SCRT1/PARD6B/IFITM1/IRS2/FADD/TNFRSF11A/SPHK1/LIMD1/ESAM/SLC16A3/ADIPOQ/RAPGEF2/FGF19	105
GO:0051674	localization of cell	105/901	1187/17046	9.82E-08	1.39E-05	1.09E-05	CDH13/PDPN/ADCY3/CHGA/CCR1/APCDD1/CTGF/DNAH6/DNAH8/EGFR/EGR3/EPHA1/EPHA3/EPHB4/FAT2/SPATA13/PHACTR1/FGF10/VASH1/FOXC2/GAPDHS/BMP10/GPER1/FFAR2/HAS1/SOX8/NRG1/NR4A1/HRH1/BARHL2/IGF1/CYR61/IL1RN/IL6/IL16/ISL1/ITGA7/ITGB2/ITGB7/JUP/KDR/LAMA3/LCK/LCP1/LGALS9/LMNA/SMAD3/MCC/MAP3K1/NOV/NRAS/NTF3/P2RY6/ATP5B/LEF1/CEND1/ANGPT4/PIK3CG/PITX2/PML/TREM1/ROBO4/ELP3/PARVA/PRKD1/BIN3/MAP2K2/PROC/PAK6/PLEKHG5/FAM60A/CCL11/CCL17/NOD2/SFRP2/SGK1/BMP4/SLC8A1/SOX9/STK10/BST2/PHLDA2/TWIST1/CCR2/ZAP70/CACNA1E/PTP4A1/CXCR4/COL18A1/CALR/SLIRP/SH3BGRL3/SCRT1/PARD6B/IFITM1/IRS2/FADD/TNFRSF11A/SPHK1/LIMD1/ESAM/SLC16A3/ADIPOQ/RAPGEF2/FGF19	105
GO:0048583	regulation of response to stimulus	236/901	3283/17046	1.05E-07	1.46E-05	1.15E-05	ABI1/CDH3/TANK/TSPAN5/CDH13/FARP1/KLRG1/RCAN2/CDKN1C/SPON2/TBR1/HCST/NPFFR2/LECT1/ESM1/CHI3L1/CIDEA/CCR1/SEZ6/TNFAIP8L1/MAP3K8/ADM/IL31RA/MIB2/MPP7/APCDD1/CTGF/CYLD/ADRB3/RNF168/ZNF366/COCH/NLRP6/DMBT1/DRD4/EGFR/A2M/UNC13D/ESR1/F11/SPATA13/FCGR2A/FGA/FGF10/RASA3/VASH1/SBNO2/ACIN1/FOXL1/FOXC2/FOXO1/AKR1B1/SPG20/MLC1/RHOBTB2/NUP210/NEDD4L/PSD3/LARP1/PUM2/ARHGEF18/MAPK8IP2/MTOR/SLC37A4/ALS2CL/RGS22/DNAJC2/FGF22/NPTN/DKK3/CYTH4/BMP10/GNAS/GPER1/FFAR2/GRB10/GSTP1/ANXA2/NRG1/HK1/HLA-B/HLA-DPA1/HLA-E/HLA-F/HLX/HMGA1/NR4A1/HSPA1L/HSP90AA1/HSP90AB1/RSPO2/IGF1/IGF2/CYR61/IL1R1/IL1RN/IL6/IL16/INHBA/IRF1/ISL1/ITGB2/ITGB7/JUP/KDR/HES5/LCK/ARHGDIA/LGALS9/LHCGR/LLGL1/LMNA/LTBP1/SMAD3/MCC/MAP3K1/MFNG/MOV10/PLEKHG7/NFATC3/NOV/NRAS/NTF3/PALM/ARHGEF3/PARK2/LEF1/C11orf73/SIRT6/PDE6B/PIK3CG/PKHD1/PLA2G2A/PML/IL20RB/TLR9/TREM1/ZDHHC13/PPP1CB/PPP1CC/ARHGEF10L/SYBU/LIMS2/IFT122/LMBRD1/PAG1/PRKD1/MAPK3/MAP2K2/PROC/MASP1/HTRA1/PSMB4/PAK6/PSMD7/PLEKHG5/CCAR2/PTPRE/PXN/RASGRF2/TRIM27/RGS12/RPA3/S100A4/CCL11/CCL17/NOD2/STRA6/SFRP2/ARHGAP9/TMEM237/BMP4/BMPR1B/SOX9/STAT2/STK3/SUPT6H/BST2/ZEB1/TLR5/TNFAIP3/TNFRSF1A/TNXB/TRAF1/TRAF5/TWIST1/CCR2/WNT10B/YWHAG/ZAP70/CA7/CACNA1E/CXCR4/FZD5/RAB7A/CARD14/BCL2L14/TMEM204/NLRX1/CALR/UNC93B1/HIST1H3A/SLA2/CMAHP/CASQ1/HOPX/TRIM63/RAE1/IFITM1/CDK10/RUNX1/TP63/RUNX3/IRS2/CRADD/FADD/TNFRSF11A/SPHK1/SKAP2/LIMD1/STARD13/PIAS2/MAP3K6/RSAD2/CD8A/TRIP10/ADIPOQ/ARHGAP29/LY86/ARHGEF10/NUP93/RAPGEF2/CD79A/TELO2/IQSEC1/FGF19	236

GO:0048519	negative regulation of biological process	285/901	4153/17046	2.25E-07	3.05E-05	2.40E-05	ABI1/CDH3/CDH13/FARP1/CDKN1C/SPEG/MRVI1/TRDN/C1D/ZBTB18/CELF1/GJB6/LECT1/CHGA/ERLIN2/B4GALT7/CARD16/CIDEA/ANKRD9/CCR1/SEZ6/TNFAIP8L1/COMP/ADM/IL31RA/HUS1B/FAM101A/APCDD1/CSTA/CTGF/SMYD1/CYLD/ADRB3/TRPV3/DDB1/RNF168/ZNF366/BHLHA15/NLRP6/DLG2/DNMT3A/DRD4/EGFR/EGR3/PATL2/A2M/ELK4/EPHA1/ESR1/F11/PHACTR1/FGA/FGF10/FHIT/RASA3/PPM1E/VASH1/SBNO2/ACIN1/FOXC2/FOXO1/SPG20/DIP2A/FLOT2/NEDD4L/PPP1R13B/PUM2/RYBP/MORC3/MTOR/GABBR1/RGS22/GAS2/FBXO2/SACS/PABPC1/DNAJC2/DKK3/VPS4A/BMP10/GNAS/GPER1/GRB10/DNAJC15/GSTP1/TMOD4/GUCY1A3/GPR132/GZMA/ANXA2/HAS1/SERPIND1/SOX8/NRG1/HLA-B/HLA-DOA/ANXA13/HLA-E/HLX/HMGA1/NR4A1/ACACB/HPCA/HOXB3/HOXC6/ACADL/HSP90AB1/ID3/ZC3H12D/COL28A1/RSPO2/IGF1/CYR61/IL1RN/IL6/INHBA/IRF1/ISL1/ITIH3/ITIH4/HILS1/KCNH2/KDR/KIF25/IPO5/HES5/STMN1/LCK/ARHGDIA/LGALS9/LMNA/LTBP1/SMAD3/MCC/MFI2/MITF/MOV10/MPZ/MT1A/MYL2/NUBP1/ATP1A2/NOV/NPPC/NRAS/NTF3/OPRL1/PAFAH2/ATP5B/PALM/PARK2/SPOCK3/LEF1/CEND1/ANGPT4/PDE4C/SIRT6/ATP8A2/PI3/PIK3CG/PITX2/PKHD1/PLA2G2A/PLAGL1/PRKAG3/PML/RIPPLY3/IL20RB/TLR9/POMC/BANP/PPP1CB/PPP1CC/PIWIL2/PRMT6/DNAJC17/FANCI/CNOT11/LIMS2/PRKAR1B/IFT122/LMBRD1/PAG1/PRKD1/MAP2K2/PRKRIR/PROC/MASP1/HTRA1/CDC42SE1/PSMB4/PSMD7/SLURP1/PTGFR/TENM2/GATAD2B/METTL14/CCAR2/PTPRE/CREBZF/FAM60A/TRIM27/RGS12/SCT/CCL17/NOD2/SFRP2/GZF1/BMP4/ZNF649/SLC8A1/BMPR1B/SLIT1/SOX9/BPI/STK3/SUPT6H/BST2/TBP/ZEB1/ACTC1/TERF1/TIMP3/TNFAIP3/TNFRSF1A/TWIST1/CCR2/TNFRSF4/WNT10B/YWHAG/ZNF177/PAX8/FZD5/RAB7A/CARD14/LST1/NLRX1/ZC3H14/ZC3H12A/RAB11FIP1/CPEB4/COL18A1/CALR/SLIRP/CAST/CAPZB/SCRT1/HIST1H3A/SLA2/NR0B2/CASQ1/HOPX/SPINK7/KDM2B/LOXL3/CBX2/IFITM1/GAS7/SCIN/CDK10/RUNX1/TP63/RUNX3/SERPINA6/IRS2/ACTN1/CRADD/FADD/ALDH1A2/SPHK1/SKAP2/LIMD1/ERI1/PIAS2/CBFA2T2/RSAD2/AURKB/DAPL1/ADIPOQ/H2AFY/N4BP1/RAPGEF2/ULK2/FGF19/NR1H4	285
GO:0048522	positive regulation of cellular process	292/901	4283/17046	2.72E-07	3.60E-05	2.83E-05	ABI1/CDH3/TSPAN5/CDH13/CDKN1C/TCIRG1/TRDN/PDPN/DMRT2/TBR1/HCST/ADCY3/RER1/ESM1/CHI3L1/ERLIN2/PSIP1/CCR1/SLC51B/SEZ6/MAP3K8/ADM/IL31RA/EGFLAM/MIB2/MPP7/CTGF/SMYD1/SH3D19/CYLD/ADRB3/TRPV3/CITED4/DDB1/RNF168/BHLHA15/DMBT1/DNMT3A/ABAT/DRD4/ECE1/EEF2/EGFR/EGR3/EIF4G1/UNC13D/EPHA1/EPHA3/ESR1/FGA/FGF10/RASA3/PPM1E/SBNO2/ACIN1/FOXC2/FOXO1/EXPH5/AKR1B1/FLOT2/NEDD4L/LARP1/PUM2/ARHGEF18/MAPK8IP2/VGLL2/MTOR/RNF144B/TENM4/GAPDHS/PABPC1/DNAJC2/FGF22/NPTN/GLS2/VPS4A/BMP10/GNAS/GPR26/GPER1/DOK7/FFAR2/GRB10/GSTP1/BRF1/GUCY1A3/GZMA/ANXA2/SOX8/NRG1/HK1/HLA-DPA1/ANXA13/HLA-E/HLX/HMGA1/NR4A1/ACACB/HPCA/HOXD3/HRH1/HSPA1L/HSP90AA1/HSP90AB1/TFAP2E/ID3/RSPO2/FMN1/BARHL2/IGF1/IGF2/CYR61/IL1RN/IL6/IL12RB2/FOXK2/INHBA/IRF1/ISL1/JUP/KCNH2/KDR/IPO5/AMIGO3/HES5/STMN1/LCK/LCP1/LDLR/ARHGDIA/LGALS9/LHCGR/LMNA/LMO2/LTB/SMAD3/MC2R/MEF2D/MAP3K1/MEOX1/MEOX2/MFI2/MFNG/MITF/NEU1/NFATC3/NFYB/NHLH2/NOV/NPPC/NRAS/NTF3/OPRL1/P2RY6/PALM/ARHGEF3/PARK2/UTP11L/LEF1/PRR16/ANGPT4/SIRT6/ATP8A2/PIK3CG/PITX2/PKHD1/PLA2G2A/PLAGL1/PML/RIPK4/TLR9/CYTL1/POMC/ZDHHC13/APBB1IP/BANP/PIWIL2/ELP3/GOLPH3L/FANCI/SMPD3/LIMS2/PRKAR1B/PAG1/PRKD1/MAPK3/MAP2K2/MRAP/HTRA1/PSMB4/PAK6/ARNTL2/RGMA/PSMD7/ACTR3B/PTGFR/PLEKHG5/TENM2/MARK4/CCAR2/PXN/RASGRF2/TRIM27/S100A4/S100A6/SCT/CCL11/CCL17/NPAS3/NOD2/SFRP2/CXCR5/TRA2B/BMP4/ZNF649/SLC8A1/BMPR1B/BOK/SOX9/STK3/STK10/SUPT6H/BST2/VAMP2/TBP/TCEA1/TCEB2/ZEB1/TEAD3/TERF1/TGM2/TLR5/TNFAIP3/TNFRSF1A/TRAF1/TRAF5/TRPC6/TWIST1/CCR2/TNFRSF4/WNT10B/YWHAG/ZAP70/CA7/PTP4A1/PAX8/CXCR4/FZD5/RAB7A/CARD14/BCL2L14/CSPP1/ZC3H12A/COL18A1/CALR/SLA2/NR0B2/HOPX/MGARP/IFITM1/SCIN/CDK10/RUNX1/TP63/RUNX3/IRS2/CRADD/FADD/TNFRSF11A/ALDH1A2/SPHK1/SKAP2/LIMD1/PRC1/PIAS2/ZFAND2A/MAP3K6/SYT7/LDB2/CBFA2T2/RSAD2/AURKB/CD8A/ADIPOQ/LY86/RAB3D/H2AFY/ARHGEF10/MICAL2/RAPGEF2/FGF19/NR1H4	292
GO:0072358	cardiovascular system development	80/901	861/17046	5.41E-07	6.83E-05	5.37E-05	CDH13/SPEG/PDPN/LECT1/ESM1/CHI3L1/COL11A1/ADM/CTGF/SMYD1/ECE1/EGR3/EPHA1/EPHB4/FGF10/VASH1/FOXL1/FOXC2/FOXO1/MTOR/TENM4/BMP10/FLVCR1/ANXA2/NRG1/NR4A1/ACACB/HOXB3/ID3/IGF1/CYR61/IL6/ISL1/ITGA7/KCNJ8/KDR/LMNA/LOX/SMAD3/MEF2D/MEOX2/MYL2/NFATC3/NOV/ATP5B/LEF1/ANGPT4/SIRT6/PIK3CG/PITX2/PML/RIPPLY3/ROBO4/PARVA/IFT122/PRKD1/ACTA2/CCL11/STRA6/SFRP2/BMP4/SLC8A1/SOX9/STK3/ACTC1/TNFAIP3/TWIST1/CCR2/FZD5/TMEM204/ZC3H12A/COL18A1/CALR/HOPX/RUNX1/ALDH1A2/SPHK1/MICAL2/RAPGEF2/FGF19	80
GO:0072359	circulatory system development	80/901	861/17046	5.41E-07	6.83E-05	5.37E-05	CDH13/SPEG/PDPN/LECT1/ESM1/CHI3L1/COL11A1/ADM/CTGF/SMYD1/ECE1/EGR3/EPHA1/EPHB4/FGF10/VASH1/FOXL1/FOXC2/FOXO1/MTOR/TENM4/BMP10/FLVCR1/ANXA2/NRG1/NR4A1/ACACB/HOXB3/ID3/IGF1/CYR61/IL6/ISL1/ITGA7/KCNJ8/KDR/LMNA/LOX/SMAD3/MEF2D/MEOX2/MYL2/NFATC3/NOV/ATP5B/LEF1/ANGPT4/SIRT6/PIK3CG/PITX2/PML/RIPPLY3/ROBO4/PARVA/IFT122/PRKD1/ACTA2/CCL11/STRA6/SFRP2/BMP4/SLC8A1/SOX9/STK3/ACTC1/TNFAIP3/TWIST1/CCR2/FZD5/TMEM204/ZC3H12A/COL18A1/CALR/HOPX/RUNX1/ALDH1A2/SPHK1/MICAL2/RAPGEF2/FGF19	80
GO:0016477	cell migration	96/901	1095/17046	5.50E-07	6.83E-05	5.37E-05	CDH13/CHGA/CCR1/APCDD1/CTGF/EGFR/EGR3/EPHA1/EPHA3/EPHB4/FAT2/SPATA13/FGF10/VASH1/FOXC2/BMP10/GPER1/FFAR2/HAS1/SOX8/NRG1/NR4A1/HRH1/BARHL2/IGF1/CYR61/IL1RN/IL6/IL16/ISL1/ITGA7/ITGB2/ITGB7/JUP/KDR/LAMA3/LCK/LCP1/LGALS9/LMNA/SMAD3/MCC/MAP3K1/NOV/NRAS/NTF3/P2RY6/ATP5B/LEF1/CEND1/ANGPT4/PIK3CG/PITX2/PML/TREM1/ROBO4/ELP3/PARVA/PRKD1/BIN3/PROC/PAK6/PLEKHG5/FAM60A/CCL11/CCL17/NOD2/SFRP2/SGK1/BMP4/SLC8A1/SOX9/STK10/BST2/PHLDA2/TWIST1/CCR2/ZAP70/PTP4A1/CXCR4/COL18A1/CALR/SH3BGRL3/SCRT1/PARD6B/IFITM1/IRS2/FADD/TNFRSF11A/SPHK1/LIMD1/ESAM/SLC16A3/ADIPOQ/RAPGEF2/FGF19	96
GO:0040011	locomotion	133/901	1666/17046	6.63E-07	8.06E-05	6.34E-05	CDH13/RCAN2/SPON2/PDPN/TBR1/ADCY3/CHGA/CCR1/COL9A3/APCDD1/CTGF/DNAH6/DNAH8/EFNA2/EGFR/EGR3/EPHA1/EPHA3/EPHB4/FAT2/SPATA13/PHACTR1/FGF10/RASA3/VASH1/FOXC2/NFASC/GAPDHS/FGF22/BMP10/GPER1/FFAR2/HAS1/SERPIND1/SOX8/NRG1/NR4A1/HRH1/HSP90AA1/HSP90AB1/BARHL2/IGF1/CYR61/IL1RN/IL6/IL16/ISL1/ITGA7/ITGB2/ITGB7/JUP/KDR/LAMA3/LCK/LCP1/LGALS9/LMNA/SMAD3/MCC/MAP3K1/ATP1A2/NOV/NRAS/NTF3/P2RY6/ATP5B/LEF1/CEND1/ANGPT4/PIK3CG/PITX2/PML/TREM1/ROBO4/ELP3/PARVA/PRKD1/BIN3/MAPK3/MAP2K2/PROC/PSMB4/PAK6/RGMA/TRPC7/PSMD7/PLEKHG5/TENM2/FAM60A/RASGRF2/CCL11/CCL17/NOD2/SFRP2/CXCR5/SGK1/BMP4/SLC8A1/SLC20A2/BMPR1B/SLIT1/SOX9/STK10/BST2/TRPC4/TRPC6/PHLDA2/TWIST1/CCR2/ZAP70/CACNA1E/PTP4A1/CACNB2/CXCR4/COL18A1/CALR/SLIRP/SH3BGRL3/SCRT1/PARD6B/IFITM1/RUNX3/IRS2/FADD/TNFRSF11A/SPHK1/LIMD1/CCRL2/ESAM/SLC16A3/ADIPOQ/RAPGEF2/FGF19	133

GO:0070887	cellular response to chemical stimulus	186/901	2524/17046	7.46E-07	8.89E-05	6.99E-05	CDH13/CDKN1C/TCIRG1/SPON2/GJB6/NPFFR2/ADCY3/LECT1/CHGA/CHI3L1/EGLN2/CIDEA/AP3S1/CCR1/IL31RA/CPS1/CTGF/CYP11A1/BHLHA15/DNMT3A/EGFR/EGR3/EIF4G1/EPHA3/ESR1/FGA/FGF10/RASA3/PPM1E/SBNO2/FOXC2/FOXO1/AKR1B1/SPG20/FLNB/MLC1/NUP210/NEDD4L/ARHGEF18/MTOR/FGF22/NPTN/GJB2/BMP10/GNAS/GPER1/FFAR2/GRB10/GSTP1/HAS1/NRG1/HLA-B/HLA-DPA1/HLA-E/HLA-F/NR4A1/HPCA/HRH1/HSP90AB1/HTR3A/IGF2/CYR61/LCE1D/IL1R1/IL1RN/IL6/IL10RA/AQP2/IL11RA/IL12RB2/IL15RA/IL16/INHBA/IRF1/AQP9/ISL1/ITGB2/JUP/KCNH2/KDR/IPO5/HES5/LCK/ARHGDIA/LGALS9/LHCGR/LMNA/LMO2/LTB/LTBP1/SMAD3/MAP3K1/MOV10/MT1A/ATP1A2/NFATC3/NOV/NRAS/OAS2/P2RY6/IL21R/ARHGEF3/PARK2/LEF1/ANGPT4/PIK3CG/PRKAG3/PML/IL20RB/TLR9/TREM1/SSH1/CYP2W1/PPP1CB/PPP1CC/SYBU/PARVA/PRKAR1B/LMBRD1/PRKD1/MAPK3/MAP2K2/HTRA1/SLAMF8/PSMB4/RGMA/PSMD7/PTGFR/PLEKHG5/PTPRE/PXN/RASGRF2/RIT2/BGLAP/CCL11/CCL17/NOD2/SFRP2/CXCR5/BMP4/SLC8A1/BMPR1B/SOX9/STAT2/BST2/VAMP2/TCEB2/ZEB1/TIMP3/TLR5/TNFAIP3/TNFRSF1A/TRPC6/TWIST1/CCR2/TNFRSF4/WNT10B/CACNA1E/PAX8/CXCR4/CARD14/TMEM204/ZC3H12A/CPEB4/CALR/NR0B2/IL1F10/TRIM63/MGARP/RAE1/IFITM1/RUNX1/IRS2/FADD/TNFRSF11A/ALDH1A2/SPHK1/CCRL2/ZFAND2A/RSAD2/ADIPOQ/LY86/NUP93/RAPGEF2/FGF19/NR1H4	186
GO:0012501	programmed cell death	136/901	1718/17046	7.76E-07	9.07E-05	7.13E-05	C1D/GJB6/CHI3L1/EGLN2/CARD16/CIDEA/ANKRD9/COMP/MAP3K8/ADM/IL31RA/CTGF/CYLD/DDB1/DNMT3A/DSG3/EGFR/EGR3/ESR1/FGA/FGF10/FHIT/ACIN1/FOXC2/FOXO1/EPB41L3/DIP2A/PPP1R13B/ARHGEF18/RYBP/RNF144B/GAS2/GLS2/BMP10/GPER1/GSTP1/GZMA/SOX8/NRG1/ANXA6/HSP90AB1/ID3/IGF1/CYR61/IL1RN/IL6/INHBA/IRF1/ISL1/ITGB2/KDR/LCK/ARHGDIA/LGALS9/LMNA/SMAD3/MEF2D/MAP3K1/MPZ/NTF3/PAFAH2/ARHGEF3/PARK2/UTP11L/LEF1/DDX47/ANGPT4/PIK3CG/PKHD1/PKM/PLAGL1/PLEC/PML/PPP2R2B/LIMS2/PRKD1/MAPK3/PROC/PSMB4/PAK6/PSMD7/PTGFR/PLEKHG5/MARK4/CCAR2/RASGRF2/SCT/NOD2/SFRP2/SGK1/BMP4/BMPR1B/BOK/SOX9/STK3/STK10/ACTC1/TERF1/TGM2/TNFAIP3/TNFRSF1A/TRAF1/TRAF5/PHLDA2/TWIST1/TNFRSF4/WNT10B/YWHAG/PAX8/CXCR4/FZD5/CARD14/BCL2L14/FAM188A/ZC3H12A/CPEB4/COL18A1/CLPTM1L/CALR/CAST/KDM2B/SCIN/TP63/RUNX3/IRS2/ACTN1/CRADD/FADD/ALDH1A2/SPHK1/MAP3K6/AURKB/DAPL1/ADIPOQ/LY86/RAPGEF2	136
GO:0009719	response to endogenous stimulus	127/901	1578/17046	8.07E-07	9.17E-05	7.21E-05	CDH13/CDKN1C/TCIRG1/NPFFR2/ADCY3/TMED10/CHRNA1/CHRNA2/CHRNA5/CIDEA/AP3S1/ADM/CPS1/CTGF/CYP11A1/CITED4/ZNF366/DNMT3A/ABAT/DRD4/AGXT/EGFR/EGR3/EIF4G1/ESR1/FGA/FGF10/RASA3/FOXC2/FOXO1/AKR1B1/SPG20/NEDD4L/ARHGEF18/MTOR/STEAP2/GATM/FGF22/NPTN/GJB2/BMP10/GNAS/GPER1/GRB10/HAS1/NRG1/NR4A1/HPCA/HRH1/HTR3A/HTR5A/IGF2/CYR61/IL1R1/IL1RN/IL6/INHBA/AQP9/ISL1/JUP/ACAT1/IPO5/HES5/LCK/LHCGR/LMO2/LOX/LTBP1/SMAD3/ME1/MAP3K1/MOV10/ATP1A2/NRAS/OPRL1/P2RY6/PARK2/LEF1/PIK3CG/PITX2/PKM/PRKAG3/PML/TLR9/SSH1/PPP1CB/PPP1CC/CHRNA9/PRKAR1B/LMBRD1/MAPK3/MAP2K2/HTRA1/PSMB4/RGMA/PSMD7/PTGFR/PTPRE/PXN/RASGRF2/BGLAP/NOD2/SFRP2/BMP4/SLC8A1/SLC9A3/BMPR1B/SOX9/VAMP2/ZEB1/TIMP3/TNFAIP3/WNT10B/PAX8/CPEB4/CALR/NR0B2/TRIM63/MGARP/RUNX1/RUNX3/IRS2/ALDH1A2/ADIPOQ/RAPGEF2/FGF19/NR1H4	127
GO:0051239	regulation of multicellular organismal process	172/901	2298/17046	8.25E-07	9.17E-05	7.21E-05	CDH3/KCNMB2/MRVI1/TRDN/SPON2/DMRT2/CELF1/CELF2/TBR1/LECT1/CHI3L1/CIDEA/CCR1/SEZ6/ADM/CPS1/FAM101A/CTGF/CYLD/ADRB3/TRPV3/NLRP6/DIO3/DMBT1/ABAT/DRD4/ECE1/EGR3/EIF4G1/EPHA1/EPHA3/ESR1/F11/FGA/FGF10/VASH1/ACIN1/FOXC2/FOXO1/SPG20/NEDD4L/PUM2/MTOR/TENM4/NPTN/BMP10/GNAS/GPER1/FFAR2/FLVCR1/GSTP1/GUCY1A3/ANXA2/SOX8/KCNIP2/NRG1/ANXA6/HLA-B/HLA-DOA/HLA-DPA1/HLA-E/HLX/ACACB/HOXB3/HOXD3/HRH1/ID3/RSPO2/BARHL2/IGF1/CYR61/IL1RN/IL6/IL12RB2/INHBA/IRF1/ISL1/JUP/KCNH2/KDR/AMIGO3/HES5/LAMA3/LCK/ARHGDIA/LGALS9/LTB/SMAD3/MCC/MITF/MYL2/NEU1/ATP1A2/NOV/NPPC/NRAS/NTF3/OPRL1/PALM/PARK2/LEF1/CEND1/ANGPT4/ATP8A2/PIK3CG/PML/IL20RB/TLR9/POMC/SSH1/LIMS2/IFT122/PRKD1/MAP2K2/PROC/TRIM27/BGLAP/SCT/CCL11/NOD2/SFRP2/SGK1/BMP4/SLC8A1/BMPR1B/SLIT1/SOX9/BPI/STK3/SUPT6H/BST2/ZEB1/TEAD3/TLR5/TNFAIP3/TNFRSF1A/PHLDA2/TWIST1/CCR2/TNFRSF4/WNT10B/YWHAG/ZAP70/CACNA1E/PAX8/CXCR4/FZD5/NLRX1/ZC3H12A/RAB11FIP1/CALR/COLQ/SH3BGRL3/SCRT1/CASQ1/HOPX/TRIM63/IFITM1/SCIN/RUNX1/TP63/FADD/TNFRSF11A/SPHK1/LIMD1/PIAS2/CBFA2T2/RSAD2/ADIPOQ/H2AFY/RAPGEF2/ULK2	172
GO:0051270	regulation of cellular component movement	66/901	672/17046	8.31E-07	9.17E-05	7.21E-05	CDH13/PDPN/TBR1/CCR1/EGFR/EPHA1/SPATA13/FGF10/VASH1/FOXC2/NEDD4L/BMP10/GPER1/HAS1/IGF1/CYR61/IL1RN/IL6/JUP/KCNH2/KDR/LAMA3/STMN1/LGALS9/LMNA/SMAD3/MCC/MAP3K1/ATP1A2/NOV/NTF3/P2RY6/LEF1/ANGPT4/PITX2/PKHD1/ROBO4/ELP3/PRKD1/MAP2K2/FAM60A/CCL11/NOD2/SFRP2/SGK1/BMP4/SLC8A1/SOX9/STK10/BST2/PHLDA2/TWIST1/CCR2/PTP4A1/COL18A1/CALR/SH3BGRL3/SCRT1/PARD6B/IFITM1/IRS2/ACTN1/FADD/SPHK1/ADIPOQ/RAPGEF2	66
GO:0009966	regulation of signal transduction	176/901	2366/17046	8.86E-07	9.60E-05	7.55E-05	CDH3/TSPAN5/CDH13/FARP1/RCAN2/CDKN1C/HCST/NPFFR2/LECT1/ESM1/CHI3L1/CIDEA/CCR1/SEZ6/TNFAIP8L1/MAP3K8/ADM/IL31RA/MIB2/MPP7/APCDD1/CTGF/CYLD/ADRB3/ZNF366/NLRP6/DRD4/EGFR/A2M/ESR1/SPATA13/FGA/FGF10/RASA3/FOXL1/FOXO1/AKR1B1/SPG20/RHOBTB2/NEDD4L/PSD3/PUM2/ARHGEF18/MAPK8IP2/MTOR/ALS2CL/RGS22/FGF22/NPTN/DKK3/CYTH4/BMP10/GNAS/GPER1/GRB10/GSTP1/ANXA2/NRG1/HSP90AB1/RSPO2/IGF1/IGF2/CYR61/IL1RN/IL6/INHBA/IRF1/ISL1/JUP/KDR/HES5/LCK/ARHGDIA/LGALS9/LHCGR/LLGL1/LMNA/LTBP1/SMAD3/MCC/MAP3K1/MFNG/PLEKHG7/NOV/NRAS/NTF3/PALM/ARHGEF3/PARK2/LEF1/PDE6B/PIK3CG/PKHD1/PLA2G2A/PML/TLR9/ZDHHC13/PPP1CB/PPP1CC/ARHGEF10L/LIMS2/IFT122/LMBRD1/PAG1/PRKD1/MAPK3/MAP2K2/HTRA1/PSMB4/PAK6/PSMD7/PLEKHG5/CCAR2/PTPRE/PXN/RASGRF2/RGS12/S100A4/CCL11/CCL17/NOD2/SFRP2/ARHGAP9/TMEM237/BMP4/BMPR1B/SOX9/STAT2/STK3/BST2/ZEB1/TLR5/TNFAIP3/TNFRSF1A/TNXB/TRAF1/TRAF5/TWIST1/WNT10B/YWHAG/ZAP70/CXCR4/FZD5/RAB7A/CARD14/BCL2L14/TMEM204/NLRX1/CALR/SLA2/CMAHP/CDK10/RUNX1/TP63/RUNX3/IRS2/CRADD/FADD/TNFRSF11A/SPHK1/SKAP2/LIMD1/STARD13/PIAS2/MAP3K6/RSAD2/CD8A/TRIP10/ADIPOQ/ARHGAP29/LY86/ARHGEF10/RAPGEF2/TELO2/IQSEC1/FGF19	176
GO:0023051	regulation of signaling	195/901	2685/17046	1.02E-06	0.00011	8.44E-05	CDH3/TSPAN5/CDH13/FARP1/RCAN2/CDKN1C/TRDN/HCST/NPFFR2/LECT1/ESM1/CHI3L1/CIDEA/CCR1/SEZ6/TNFAIP8L1/MAP3K8/ADM/IL31RA/MIB2/MPP7/APCDD1/CTGF/CYLD/ADRB3/ZNF366/NLRP6/ABAT/DRD4/ECE1/EGFR/A2M/ESR1/SPATA13/FGA/FGF10/RASA3/FOXL1/FOXO1/AKR1B1/SPG20/RHOBTB2/NEDD4L/PSD3/PUM2/ARHGEF18/MAPK8IP2/MTOR/ALS2CL/PNKD/RGS22/FGF22/NPTN/DKK3/CYTH4/BMP10/GNAS/GPER1/FFAR2/GRB10/GSTP1/ANXA2/NRG1/HRH1/HSP90AB1/RSPO2/IGF1/IGF2/CYR61/IL1RN/IL6/INHBA/IRF1/ISL1/JUP/KDR/HES5/LCK/ARHGDIA/LGALS9/LHCGR/LLGL1/LMNA/LTBP1/SMAD3/MCC/MAP3K1/MFNG/PLEKHG7/ATP1A2/NOV/NRAS/NTF3/PALM/ARHGEF3/PARK2/LEF1/PDE4C/PDE6B/PIK3CG/PKHD1/PLA2G2A/PML/TLR9/POMC/ZDHHC13/PPP1CB/PPP1CC/ARHGEF10L/SYBU/LIMS2/PRKAR1B/IFT122/LMBRD1/PAG1/PRKD1/MAPK3/MAP2K2/HTRA1/PSMB4/PAK6/PSMD7/PLEKHG5/CCAR2/PTPRE/PXN/RASGRF2/RGS12/S100A4/SCT/CCL11/CCL17/NOD2/SFRP2/ARHGAP9/TMEM237/BMP4/BMPR1B/SOX9/STAT2/STK3/BST2/VAMP2/ZEB1/TLR5/TNFAIP3/TNFRSF1A/TNXB/TRAF1/TRAF5/TWIST1/WNT10B/YWHAG/ZAP70/CA7/CACNA1E/PAX8/CXCR4/FZD5/RAB7A/CARD14/BCL2L14/TMEM204/NLRX1/RAB11FIP1/CALR/SLA2/CMAHP/NR0B2/CDK10/RUNX1/TP63/RUNX3/IRS2/CRADD/FADD/TNFRSF11A/SPHK1/SKAP2/LIMD1/STARD13/PIAS2/MAP3K6/SYT7/RSAD2/CD8A/TRIP10/ADIPOQ/ARHGAP29/LY86/ARHGEF10/RAPGEF2/TELO2/IQSEC1/FGF19	195

GO:0098602	single organism cell adhesion	68/901	704/17046	1.03E-06	0.00011	8.44E-05	CDH3/CDH9/PDPN/PKP3/MAP3K8/CSTA/CYLD/DDOST/EGFR/EGR3/UNC13D/EPHA1/FGA/NFASC/FLOT2/MTOR/GNAS/HLA-DOA/HLA-DPA1/HLA-E/HLX/ZC3H12D/IGF1/IGF2/CYR61/IL1RN/IL6/IRF1/ITGA7/ITGB2/ITGB7/JUP/LCK/LCP1/LGALS9/SMAD3/MFI2/NFATC3/NOV/LEF1/PIK3CG/PKHD1/IL20RB/APBB1IP/FBLIM1/LIMS2/PARVA/PAG1/TENM2/PXN/NOD2/BMP4/SOX9/STK10/ZEB1/TNXB/CCR2/TNFRSF4/ZAP70/FZD5/CALR/ANTXR1/SLA2/FADD/ESAM/RSAD2/CD8A/ADIPOQ	68
GO:0042127	regulation of cell proliferation	114/901	1385/17046	1.08E-06	0.00011	8.74E-05	ABI1/CDH3/CDH13/CDKN1C/SPEG/TCIRG1/GJB6/LECT1/ESM1/B4GALT7/ADM/IL31RA/CTGF/EGFR/EGR3/EPHA1/ESR1/FGF10/VASH1/FOXO1/AKR1B1/MORC3/MTOR/FBXO2/BMP10/GPER1/GSTP1/ANXA2/SOX8/NRG1/HLA-DPA1/HLA-E/HLX/HMGA1/NR4A1/TFAP2E/ZC3H12D/IGF1/IGF2/CYR61/IL6/IL12RB2/INHBA/IRF1/ISL1/JUP/KDR/HES5/LCK/LGALS9/SMAD3/MCC/MITF/NEU1/NOV/NPPC/NRAS/NTF3/LEF1/CEND1/SIRT6/ATP8A2/PITX2/PKHD1/PLA2G2A/PML/RIPPLY3/IL20RB/LIMS2/IFT122/PRKD1/PRKRIR/HTRA1/SLURP1/PTGFR/RPA3/S100A6/CCL11/NOD2/SFRP2/SGK1/BMP4/BMPR1B/SOX9/STK3/ZEB1/TNFAIP3/TRAF5/TWIST1/CCR2/TNFRSF4/WNT10B/ZAP70/FZD5/LST1/COL18A1/CALR/KDM2B/IFITM1/SCIN/CDK10/RUNX1/TP63/RUNX3/IRS2/FADD/TNFRSF11A/ALDH1A2/SPHK1/SKAP2/PRC1/ADIPOQ/RAPGEF2/FGF19	114
GO:0006915	apoptotic process	134/901	1700/17046	1.20E-06	0.00012	9.52E-05	C1D/GJB6/CHI3L1/EGLN2/CARD16/CIDEA/ANKRD9/COMP/MAP3K8/ADM/IL31RA/CTGF/CYLD/DDB1/DNMT3A/DSG3/EGFR/EGR3/ESR1/FGA/FGF10/FHIT/ACIN1/FOXC2/FOXO1/EPB41L3/DIP2A/PPP1R13B/ARHGEF18/RYBP/RNF144B/GAS2/GLS2/BMP10/GPER1/GSTP1/GZMA/SOX8/NRG1/ANXA6/HSP90AB1/ID3/IGF1/CYR61/IL1RN/IL6/INHBA/IRF1/ISL1/ITGB2/KDR/LCK/ARHGDIA/LGALS9/LMNA/SMAD3/MEF2D/MAP3K1/MPZ/NTF3/PAFAH2/ARHGEF3/PARK2/UTP11L/LEF1/DDX47/ANGPT4/PIK3CG/PKHD1/PLAGL1/PLEC/PML/PPP2R2B/LIMS2/PRKD1/MAPK3/PROC/PSMB4/PAK6/PSMD7/PTGFR/PLEKHG5/CCAR2/RASGRF2/SCT/NOD2/SFRP2/SGK1/BMP4/BMPR1B/BOK/SOX9/STK3/STK10/ACTC1/TERF1/TGM2/TNFAIP3/TNFRSF1A/TRAF1/TRAF5/PHLDA2/TWIST1/TNFRSF4/WNT10B/YWHAG/PAX8/CXCR4/FZD5/CARD14/BCL2L14/FAM188A/ZC3H12A/CPEB4/COL18A1/CLPTM1L/CALR/CAST/KDM2B/SCIN/TP63/RUNX3/IRS2/ACTN1/CRADD/FADD/ALDH1A2/SPHK1/MAP3K6/AURKB/DAPL1/ADIPOQ/LY86/RAPGEF2	134
GO:0010646	regulation of cell communication	197/901	2731/17046	1.42E-06	0.00014	0.00011	CDH3/TSPAN5/CDH13/FARP1/RCAN2/CDKN1C/TRDN/HCST/NPFFR2/LECT1/ESM1/CHI3L1/CIDEA/CCR1/SEZ6/TNFAIP8L1/MAP3K8/ADM/IL31RA/MIB2/MPP7/APCDD1/CTGF/CYLD/ADRB3/ZNF366/NLRP6/ABAT/DRD4/EGFR/A2M/ESR1/SPATA13/FGA/FGF10/RASA3/FOXL1/FOXO1/AKR1B1/SPG20/RHOBTB2/NEDD4L/PSD3/LARP1/PUM2/ARHGEF18/MAPK8IP2/MTOR/ALS2CL/PNKD/RGS22/FGF22/NPTN/DKK3/CYTH4/BMP10/GNAS/GPER1/FFAR2/GRB10/GSTP1/ANXA2/NRG1/HK1/HRH1/HSP90AB1/RSPO2/IGF1/IGF2/CYR61/IL1RN/IL6/INHBA/IRF1/ISL1/JUP/KDR/HES5/LCK/ARHGDIA/LGALS9/LHCGR/LLGL1/LMNA/LTBP1/SMAD3/MCC/MAP3K1/MFNG/PLEKHG7/ATP1A2/NOV/NRAS/NTF3/PALM/ARHGEF3/PARK2/LEF1/PDE4C/PDE6B/PIK3CG/PKHD1/PLA2G2A/PML/TLR9/POMC/ZDHHC13/PPP1CB/PPP1CC/ARHGEF10L/SYBU/LIMS2/PRKAR1B/IFT122/LMBRD1/PAG1/PRKD1/MAPK3/MAP2K2/HTRA1/PSMB4/PAK6/PSMD7/PLEKHG5/CCAR2/PTPRE/PXN/RASGRF2/RGS12/S100A4/SCT/CCL11/CCL17/NOD2/SFRP2/ARHGAP9/TMEM237/BMP4/SLC8A1/BMPR1B/SOX9/STAT2/STK3/BST2/VAMP2/ZEB1/TLR5/TNFAIP3/TNFRSF1A/TNXB/TRAF1/TRAF5/TWIST1/WNT10B/YWHAG/ZAP70/CA7/CACNA1E/PAX8/CXCR4/FZD5/RAB7A/CARD14/BCL2L14/TMEM204/NLRX1/RAB11FIP1/CALR/SLA2/CMAHP/NR0B2/CDK10/RUNX1/TP63/RUNX3/IRS2/CRADD/FADD/TNFRSF11A/SPHK1/SKAP2/LIMD1/STARD13/PIAS2/MAP3K6/SYT7/RSAD2/CD8A/TRIP10/ADIPOQ/ARHGAP29/LY86/ARHGEF10/RAPGEF2/TELO2/IQSEC1/FGF19	197
GO:0050793	regulation of developmental process	149/901	1953/17046	1.82E-06	0.00018	0.00014	CDH3/PDPN/DMRT2/CELF1/TBR1/LECT1/CHI3L1/CCR1/SEZ6/ADM/FAM101A/CTGF/SMYD1/SH3D19/CYLD/ADRB3/BHLHA15/COCH/DIO3/DMBT1/EGR3/EIF4G1/UNC13D/EPHA1/EPHA3/ESR1/FGA/FGF10/VASH1/ACIN1/FOXC2/FOXO1/SPG20/EPB41L3/FLOT2/NEDD4L/ARHGEF18/MTOR/TENM4/GAS2/NPTN/BMP10/GNAS/GPER1/FLVCR1/SOX8/NRG1/HLA-B/HLA-DOA/HLX/HMGA1/ACACB/HOXB3/HOXD3/ID3/RSPO2/BARHL2/IGF1/CYR61/IL1RN/IL6/INHBA/IRF1/ISL1/ITGA7/ITGB2/JUP/KDR/AMIGO3/HES5/LAMA3/LCK/ARHGDIA/LGALS9/LMNA/SMAD3/MFI2/MITF/NEU1/NOV/NPPC/NRAS/NTF3/PALM/PARK2/LEF1/CEND1/ANGPT4/ATP8A2/PLA2G2A/PML/SSH1/FBLIM1/PALMD/LIMS2/PARVA/IFT122/PRKD1/MAP2K2/CDC42SE1/ERMN/PXN/BGLAP/CCL11/CCL17/SFRP2/VPS33A/BMP4/SLC8A1/BMPR1B/SLIT1/SOX9/STK3/SUPT6H/ZEB1/TEAD3/TNFAIP3/TNFRSF1A/PHLDA2/TWIST1/CCR2/WNT10B/YWHAG/ZAP70/PAX8/CXCR4/LST1/ZC3H12A/CALR/COLQ/CAPZB/SCRT1/HOPX/TTBK1/IFITM1/GAS7/SCIN/RUNX1/TP63/RUNX3/FADD/SPHK1/LIMD1/PIAS2/CBFA2T2/ADIPOQ/H2AFY/RAPGEF2/ULK2	149
GO:0098609	cell-cell adhesion	75/901	816/17046	1.87E-06	0.00018	0.00014	CDH3/CDH9/CDH12/CDH13/PDPN/PKP3/FAT3/MAP3K8/CSTA/CYLD/DDOST/DSG3/EGFR/EGR3/FAT2/FGA/NFASC/FLOT2/MTOR/NPTN/GNAS/HLA-DOA/HLA-DPA1/HLA-E/HLX/ZC3H12D/IGF1/IGF2/CYR61/IL1RN/IL6/IRF1/ITGA7/ITGB2/ITGB7/JUP/AMIGO3/CDHR4/LCK/LCP1/LGALS9/SMAD3/NFATC3/NOV/LEF1/PIK3CG/PKHD1/IL20RB/APBB1IP/FBLIM1/LIMS2/PARVA/PAG1/PCDHGC4/PCDHGB7/PCDHGB3/PCDHGA11/TENM2/NOD2/PCDH20/BMP4/SOX9/STK10/ZEB1/TNXB/CCR2/TNFRSF4/ZAP70/FZD5/SLA2/FADD/ESAM/RSAD2/CD8A/ADIPOQ	75
GO:0008219	cell death	140/901	1816/17046	2.27E-06	0.00021	0.00017	C1D/GJB6/CHI3L1/EGLN2/CARD16/CIDEA/ANKRD9/COMP/MAP3K8/ADM/IL31RA/PARP4/CTGF/CYLD/DDB1/DNMT3A/DSG3/EGFR/EGR3/ESR1/FGA/FGF10/FHIT/ACIN1/FOXC2/FOXO1/EPB41L3/DIP2A/PPP1R13B/ARHGEF18/RYBP/RNF144B/GAS2/CLUL1/GLS2/BMP10/GPER1/GSTP1/GZMA/SOX8/NRG1/ANXA6/HSP90AB1/ID3/IGF1/CYR61/IL1RN/IL6/INHBA/IRF1/ISL1/ITGB2/KDR/LCK/ARHGDIA/LGALS9/LMNA/SMAD3/MEF2D/MAP3K1/MEOX2/MPZ/NOV/NTF3/PAFAH2/ARHGEF3/PARK2/UTP11L/LEF1/DDX47/ANGPT4/PIK3CG/PKHD1/PKM/PLAGL1/PLEC/PML/PPP2R2B/LIMS2/PRKD1/MAPK3/PROC/PSMB4/PAK6/PSMD7/PTGFR/PLEKHG5/MARK4/CCAR2/RASGRF2/SCT/NOD2/SFRP2/SGK1/BMP4/BMPR1B/BOK/SOX9/STK3/STK10/ACTC1/TERF1/TGM2/TNFAIP3/TNFRSF1A/TRAF1/TRAF5/PHLDA2/TWIST1/TNFRSF4/WNT10B/YWHAG/PAX8/CXCR4/FZD5/CARD14/BCL2L14/FAM188A/ZC3H12A/CPEB4/COL18A1/CLPTM1L/CALR/CAST/KDM2B/SCIN/TP63/RUNX3/IRS2/ACTN1/CRADD/FADD/ALDH1A2/SPHK1/MAP3K6/AURKB/DAPL1/ADIPOQ/LY86/RAPGEF2	140
GO:0016265	death	140/901	1816/17046	2.27E-06	0.00021	0.00017	C1D/GJB6/CHI3L1/EGLN2/CARD16/CIDEA/ANKRD9/COMP/MAP3K8/ADM/IL31RA/PARP4/CTGF/CYLD/DDB1/DNMT3A/DSG3/EGFR/EGR3/ESR1/FGA/FGF10/FHIT/ACIN1/FOXC2/FOXO1/EPB41L3/DIP2A/PPP1R13B/ARHGEF18/RYBP/RNF144B/GAS2/CLUL1/GLS2/BMP10/GPER1/GSTP1/GZMA/SOX8/NRG1/ANXA6/HSP90AB1/ID3/IGF1/CYR61/IL1RN/IL6/INHBA/IRF1/ISL1/ITGB2/KDR/LCK/ARHGDIA/LGALS9/LMNA/SMAD3/MEF2D/MAP3K1/MEOX2/MPZ/NOV/NTF3/PAFAH2/ARHGEF3/PARK2/UTP11L/LEF1/DDX47/ANGPT4/PIK3CG/PKHD1/PKM/PLAGL1/PLEC/PML/PPP2R2B/LIMS2/PRKD1/MAPK3/PROC/PSMB4/PAK6/PSMD7/PTGFR/PLEKHG5/MARK4/CCAR2/RASGRF2/SCT/NOD2/SFRP2/SGK1/BMP4/BMPR1B/BOK/SOX9/STK3/STK10/ACTC1/TERF1/TGM2/TNFAIP3/TNFRSF1A/TRAF1/TRAF5/PHLDA2/TWIST1/TNFRSF4/WNT10B/YWHAG/PAX8/CXCR4/FZD5/CARD14/BCL2L14/FAM188A/ZC3H12A/CPEB4/COL18A1/CLPTM1L/CALR/CAST/KDM2B/SCIN/TP63/RUNX3/IRS2/ACTN1/CRADD/FADD/ALDH1A2/SPHK1/MAP3K6/AURKB/DAPL1/ADIPOQ/LY86/RAPGEF2	140

GO:0019722	calcium-mediated signaling	21/901	125/17046	2.32E-06	0.00021	0.00017	CDH13/RCAN2/BHLHA15/DRD4/EGFR/NRG1/HPCA/IGF1/KDR/ATP1A2/NFATC3/MCTP2/PTGFR/TENM2/SLC8A1/ZAP70/CXCR4/SLA2/CASQ1/SPHK1/CD8A	21
GO:0032720	negative regulation of tumor necrosis factor production	11/901	38/17046	2.73E-06	0.00025	0.00019	CIDEA/GSTP1/LGALS9/POMC/TRIM27/NOD2/BPI/TNFAIP3/TWIST1/ZC3H12A/ADIPOQ	11
GO:0002376	immune system process	175/901	2392/17046	2.76E-06	0.00025	0.00019	ABI1/TANK/CD300LD/KLRG1/CDKN1C/SPON2/HCST/ADCY3/CHGA/CCR1/MAP3K8/ADM/IL31RA/CYLD/ESCO2/DDOST/RNF168/COCH/NLRP6/DMBT1/EEF2/EFNA2/EGFR/EGR3/A2M/EML1/UNC13D/FCGR2A/FGA/FGF10/RASA3/SBNO2/ACIN1/FOXL1/FOXO1/FLOT2/PUM2/MTOR/SLC37A4/FGF22/AMPD3/GNAS/GPER1/FFAR2/FLVCR1/GZMA/ANXA2/NRG1/HLA-B/HLA-DOA/HLA-DPA1/HLA-E/HLA-F/HLX/NR4A1/HOXB3/HRH1/HSP90AA1/HSP90AB1/ZC3H12D/CD300E/IGF1/IGF2/IL1R1/IL1RN/IL6/IL16/INHBA/IRF1/AQP9/ITGB2/ITGB7/KCNJ8/KDR/HES5/LCK/LCP1/LGALS9/LMO2/LTB/SMAD3/MAP3K1/MEOX1/MITF/MOV10/NFATC3/NOV/NRAS/OAS2/IL21R/LEF1/ANGPT4/PIK3CG/PITX2/PML/IL20RB/TLR9/TREM1/POU2AF1/APBB1IP/HERC6/SMPD3/PRKAR1B/PAG1/PRKD1/MAPK3/MAP2K2/PROC/MASP1/HTRA1/PSMB4/PSMD7/PTPRE/RASGRF2/TRIM27/DEFB134/BGLAP/CCL11/CCL17/NOD2/TINAGL1/SFRP2/CXCR5/VPS33A/BMP4/SOX9/BPI/STAT2/STK3/STK10/SUPT6H/BST2/VAMP2/TCEA1/ZEB1/TLR5/TNFAIP3/CCR2/TNFRSF4/WNT10B/ZAP70/CA7/CXCR4/FZD5/RAB7A/LST1/NLRX1/ZC3H12A/C6orf25/CALR/UNC93B1/HIST1H3A/SLA2/IL1F10/IFITM1/SCIN/RUNX1/RUNX3/IRS2/ACTN1/FADD/TNFRSF11A/ENDOU/SKAP2/ESAM/SLC16A3/RSAD2/IL32/CD8A/ADIPOQ/LY86/NUP93/RAPGEF2/CD79A/FGF19	175
GO:0019222	regulation of metabolic process	386/901	6084/17046	3.16E-06	0.00028	0.00022	ABI1/CDH3/ZNF783/CDH13/MBNL2/FARP1/RCAN2/CDKN1C/C1D/ZBTB18/PITRM1/DMRT2/CELF1/TBR1/NPFFR2/ADCY3/PNRC1/TMED10/HNRNPUL1/CHI3L1/ERLIN2/PSIP1/EGLN2/CARD16/ZBED9/CIDEA/CCR1/SLC51B/MAP3K8/ZFP42/ADM/IL31RA/ZNF358/LDLRAD3/CSTA/ZNF738/CTGF/SMYD1/SH3D19/CYLD/ADRB3/ESCO2/ZNF782/ZNF709/ZNF781/CITED4/RNF168/ZNF366/BHLHA15/NLRP6/DLG2/DNMT3A/DRD4/ECE1/EEF2/EGFR/EGR3/PATL2/EIF4G1/A2M/ELK4/EPHA1/EPHA3/ESR1/SPATA13/PHACTR1/SP8/FGA/FGF10/FHIT/XRN2/RASA3/PPM1E/SBNO2/TRAK1/MSRB2/ACIN1/FOXL1/FOXC2/TBC1D9B/FOXO1/SPG20/GGA3/DIP2A/FLOT2/TBC1D1/NEDD4L/PSD3/LARP1/PUM2/ARHGEF18/RYBP/MAPK8IP2/VGLL2/MTOR/GABBR1/RASGEF1C/RNF144B/ZNF549/ALS2CL/PNKD/RGS22/FBXO2/GAPDHS/PABPC1/DNAJC2/FGF22/NPTN/DKK3/CYTH4/BMP10/ZNF638/GNAS/ZNF311/ZNF844/GPER1/DOK7/GRB10/ZBTB44/DNAJC15/GSTP1/GTF2B/BRF1/GUCY1A3/GZMA/ANXA2/SERPIND1/SOX8/NRG1/HK1/HLX/HMGA1/NR4A1/ACACB/HPCA/APBA2/HOXB3/HOXC4/HOXC5/HOXC6/HOXD3/AGFG2/HRH1/ACADL/HSP90AA1/HSP90AB1/TFAP2E/ID3/COL28A1/BARHL2/IGF1/IGF2/CYR61/IL1RN/IL6/IL16/FOXK2/INHBA/IRF1/ISL1/ITGB2/ITIH3/ITIH4/JUP/USP50/HILS1/KDR/KIF25/IPO5/HES5/AFF3/LCK/LDLR/ARHGDIA/LGALS9/LHCGR/LLGL1/LMNA/LMO2/LTB/SMAD3/MC2R/ME1/ME2/MEF2D/MAP3K1/MEOX1/MEOX2/MFI2/MITF/LHX8/MOV10/PLEKHG7/NFATC3/NFYB/NHLH2/NOV/NPPC/NRAS/NTF3/OPRL1/PALM/ARHGEF3/PARK2/SPOCK3/LEF1/PRR16/ANGPT4/SIRT6/PGAM2/PI3/PIK3CG/PITX2/PKHD1/PLA2G2A/PLAGL1/PML/RIPPLY3/PNLIP/RIPK4/TLR9/CYTL1/POMC/SSH1/RIN2/POU2AF1/BNC2/MED18/BANP/PPP1CB/PPP1CC/PIWIL2/ELP3/ARHGEF10L/PRMT6/DNAJC17/ZNF532/PPP2R2B/FANCI/CNOT11/VAC14/PRKAR1B/CISD1/PRKD1/MYNN/MAPK3/MAP2K2/MRAP/PRMT8/MASP1/HTRA1/SLAMF8/CDC42SE1/PSMB4/PAK6/ARNTL2/RGMA/PRDM11/PSMD7/PTGFR/PLEKHG5/TENM2/GATAD2B/METTL14/CCAR2/PXN/CREBZF/ACTA2/RASGRF2/TRIM27/RGS12/SCT/CCL11/CCL17/NPAS3/NOD2/SFRP2/ARHGAP9/TRA2B/GZF1/SGK1/BMP4/ZNF649/BMPR1B/BRD9/ZSCAN18/BOK/SOX9/STAT2/STK3/STK10/SUPT6H/BST2/TAF4B/TBP/TCEA1/TCEB2/ZEB1/ACTC1/TEAD3/TERF1/TIMP3/TLE3/TLR5/TNFAIP3/TNFRSF1A/TNXB/TRAF1/TRAF5/PHLDA2/TWIST1/CCR2/TNFRSF4/UCP1/VARS/WNT10B/YWHAG/ZNF7/ZNF124/ZNF177/PAX8/CXCR4/FZD5/RAB7A/CARD14/ZNF665/ZC3H14/ZNF606/ZC3H12A/CPEB4/ZNF436/CALR/SLIRP/CAST/SH3BGRL3/SCRT1/HIST1H3A/SLA2/ZNF397/NR0B2/HOPX/SPINK7/TRIM63/KDM2B/LOXL3/CBX2/GAS7/CDK10/RUNX1/TP63/RUNX3/SERPINA6/IRS2/ACTN1/CRADD/FADD/TNFRSF11A/ALDH1A2/SPHK1/BUD31/CCNA1/LIMD1/ERI1/STARD13/PIAS2/ZFAND2A/MAP3K6/LDB2/CBFA2T2/AURKB/DAPL1/ADIPOQ/ARHGAP29/H2AFY/ARHGEF10/MICAL2/N4BP1/VGLL4/RAPGEF2/ULK2/USP6NL/ZBTB39/RABGAP1L/IQSEC1/FGF19/NR1H4	386
GO:0032989	cellular component morphogenesis	115/901	1432/17046	3.19E-06	0.00028	0.00022	ABI1/FARP1/SPON2/PDPN/TBR1/LECT1/FRMD6/CNP/COL9A3/SCLT1/SH3D19/CYLD/COCH/EFNA2/EGFR/TMEM17/UNC13D/EPHA1/EPHA3/EPHB4/FGA/FGF10/RASA3/BTBD3/SPG20/NFASC/EPB41L3/FLNB/NEDD4L/ARHGEF18/MAPK8IP2/TENM4/GAS2/FGF22/BMP10/TMOD4/NRG1/HSP90AA1/HSP90AB1/FMN1/BARHL2/IL6/ISL1/ITGA7/ITGB2/ITGB7/KDR/STMN1/ARHGDIA/LLGL1/SMAD3/MAP3K1/MFI2/MYL2/NRAS/NTF3/PALM/PARK2/LEF1/ATP8A2/PKHD1/SSH1/FBLIM1/PALMD/PARVA/IFT122/BIN3/MAPK3/MAP2K2/CDC42SE1/PSMB4/RGMA/TRPC7/PSMD7/TENM2/ERMN/PXN/RASGRF2/S100A4/S100A6/CCL11/SFRP2/DNAI2/SGK1/TMEM237/VPS33A/BMP4/BMPR1B/SLIT1/SOX9/ACTC1/TRPC4/TRPC6/TWIST1/CACNB2/PAX8/LST1/C6orf25/COL18A1/CALR/SLIRP/CAPZB/ANTXR1/CASQ1/PARD6B/TTBK1/LOXL3/GAS7/RUNX3/IRS2/ACTN1/LIMD1/RAPGEF2/ULK2/FGF19	115
GO:0030036	actin cytoskeleton organization	52/901	511/17046	4.53E-06	0.00038	0.0003	ABI1/FAM101A/CTGF/EPHA1/EPHA3/PHACTR1/FGF10/PPM1E/MSRB2/LIMCH1/EPB41L3/FLNB/ARHGEF18/MTOR/PLEK2/BMP10/TMOD4/FMN1/LCP1/LLGL1/SMAD3/MAP3K1/MYL2/NEDD9/NTF3/PARK2/SSH1/PARVA/TTC17/BIN3/PAK6/ACTR3B/ERMN/TRIM27/CCL11/PARVG/MICAL1/BST2/ACTC1/TNXB/CALR/CAPZB/SH3BGRL3/ANTXR1/CASQ1/GAS7/SCIN/ACTN1/TRIP10/ARHGEF10/MICAL2/IQSEC1	52
GO:0035556	intracellular signal transduction	179/901	2478/17046	4.58E-06	0.00038	0.0003	ABI1/TANK/CDH13/FARP1/RCAN2/MRVI1/HCST/NPFFR2/ADCY3/CHI3L1/CCR1/SEZ6/TNFAIP8L1/MAP3K8/ADM/IL31RA/MIB2/CTGF/SGOL1/PPM1L/CYLD/ADRB3/BHLHA15/NLRP6/DRD4/EGFR/A2M/ESR1/SPATA13/FGA/FGF10/FHIT/RASA3/FOXO1/AKR1B1/RHOBTB2/PSD3/LARP1/PPP1R13B/PUM2/ARHGEF18/MAPK8IP2/TSSK2/MTOR/RASGEF1C/ALS2CL/ACOT11/PLEK2/FGF22/SDCBP2/CYTH4/BMP10/GNAS/GPER1/GSTP1/GUCY1A3/NRG1/NR4A1/HPCA/HRH1/HTR5A/IGF1/IGF2/CYR61/IL1RN/IL6/INHBA/ISL1/KCNH2/KDR/HES5/STMN1/LCK/LCP1/ARHGDIA/LGALS9/LHCGR/RAB19/SMAD3/MAP3K1/MOV10/PLEKHG7/ATP1A2/NFATC3/NOV/NRAS/NTF3/ARHGEF3/PARK2/PDE7A/PIK3CG/PKHD1/PLA2G2A/PRKAG3/PML/TLR9/TREM1/RIN2/ZDHHC13/PPP1CB/PPP1CC/ARHGEF10L/MOB1A/IFT122/MCTP2/LMBRD1/PAG1/PRKD1/WSB2/MAPK3/MAP2K2/PSMB4/PAK6/PSMD7/PTGFR/PLEKHG5/TENM2/CCAR2/PXN/RASGRF2/RGS12/RIT2/S100A4/CCL11/CCL17/NOD2/SFRP2/ARHGAP9/SGK1/BMP4/SLC8A1/BOK/SOX9/STAT2/STK3/STK10/BST2/TEAD3/TNFAIP3/TNFRSF1A/TNXB/TRAF5/TWIST1/CCR2/YWHAG/ZAP70/CXCR4/FZD5/RAB7A/CARD14/NLRX1/CAPS/HIST1H3A/SLA2/CASQ1/IFITM1/CDK10/TP63/IRS2/CRADD/FADD/TNFRSF11A/SPHK1/LIMD1/PRC1/STARD13/MAP3K6/AURKB/CD8A/TRIP10/ADIPOQ/ARHGAP29/RAB3D/RAB36/ARHGEF10/RAPGEF2/TELO2/IQSEC1/FGF19	179

GO:1903556	negative regulation of tumor necrosis factor superfamily cytokine production	11/901	40/17046	4.76E-06	0.00039	0.0003	CIDEA/GSTP1/LGALS9/POMC/TRIM27/NOD2/BPI/TNFAIP3/TWIST1/ZC3H12A/ADIPOQ	11
GO:0048646	anatomical structure formation involved in morphogenesis	92/901	1090/17046	4.76E-06	0.00039	0.0003	ABI1/CDH13/PDPN/DMRT2/TBR1/LECT1/ESM1/CHI3L1/COL11A1/SCLT1/ADM/CTGF/CYLD/EGR3/TMEM17/UNC13D/EPHA1/EPHB4/FGF10/VASH1/SBNO2/FOXC2/NFASC/EPB41L3/TENM4/BMP10/GNAS/TMOD4/ANXA2/SOX8/NR4A1/HOXB3/FMN1/CYR61/IL6/INHBA/ISL1/ITGA7/ITGB2/KDR/HES5/LAMA3/SMAD3/MEOX1/MEOX2/MYL2/NFATC3/NOV/ATP5B/LEF1/CEND1/ANGPT4/ATP8A2/PIK3CG/PITX2/PKHD1/PML/ROBO4/PARVA/IFT122/PRKD1/MAPK3/MAP2K2/HTRA1/CCL11/STRA6/SFRP2/DNAI2/TMEM237/VPS33A/BMP4/SOX9/STK3/ACTC1/TGM2/TNFAIP3/TWIST1/CCR2/WNT10B/PAX8/FZD5/ZC3H12A/C6orf25/COL18A1/CAST/CASQ1/HOPX/KDM2B/RUNX1/TP63/ACTN1/SPHK1	92
GO:0032680	regulation of tumor necrosis factor production	17/901	91/17046	4.82E-06	0.00039	0.0003	SPON2/CIDEA/GSTP1/HLA-E/ISL1/LGALS9/TLR9/POMC/TRIM27/NOD2/BPI/TNFAIP3/TWIST1/CCR2/ZC3H12A/FADD/ADIPOQ	17
GO:0032879	regulation of localization	159/901	2151/17046	4.94E-06	0.00039	0.00031	CDH3/CDH13/TRDN/PDPN/TBR1/RER1/CHGA/CIDEA/CCR1/SLC51B/CYLD/FITM1/TRPV3/NLRP6/ABAT/DRD4/EGFR/UNC13D/EPHA1/SPATA13/FGA/FGF10/VASH1/FOXC2/EXPH5/MLC1/TBC1D1/NUP210/NEDD4L/MAPK8IP2/MTOR/GLS2/VPS4A/BMP10/GNAS/CRACR2B/GPR26/GPER1/FFAR2/GRB10/ANXA2/HAS1/KCNIP2/NRG1/ANXA13/HLA-E/ACACB/HPCA/HSPA1L/HSP90AB1/IGF1/CYR61/IL1RN/IL6/INHBA/ISL1/JUP/KCNH2/KCNJ8/KCNJ9/KDR/IPO5/LAMA3/STMN1/LCK/LCP1/LGALS9/LLGL1/LMNA/SMAD3/MCC/MAP3K1/ATP1A2/NFATC3/NOV/NTF3/OPRL1/P2RY6/PARK2/LEF1/ANGPT4/PDE4C/SIRT6/ATP8A2/PIK3CG/PITX2/PKHD1/PML/FXYD6/TLR9/POMC/PON1/ROBO4/ELP3/GOLPH3L/TRPV6/SMPD3/SLC30A10/SYBU/PRKAR1B/LMBRD1/PRKD1/MAPK3/MAP2K2/FAM60A/RASGRF2/TRIM27/SCT/CCL11/NOD2/SFRP2/SGK1/BMP4/SLC8A1/SLC9A3/SOX9/STK10/SUPT6H/BST2/VAMP2/TRPC6/PHLDA2/TWIST1/CCR2/TNFRSF4/YWHAG/CA7/CACNA1E/PTP4A1/CACNB2/PAX8/FZD5/RAB7A/ZC3H12A/RAB11FIP1/COL18A1/CALR/SH3BGRL3/SCRT1/NR0B2/CASQ1/PARD6B/RAE1/IFITM1/SCIN/IRS2/ACTN1/FADD/TNFRSF11A/SPHK1/SYT7/RSAD2/REEP6/ADIPOQ/RAB3D/NUP93/RAPGEF2/RABGAP1L/FGF19	159
GO:0061061	muscle structure development	55/901	554/17046	5.05E-06	0.0004	0.00031	SPEG/ZBTB18/CHRNA1/COL11A1/ADM/SMYD1/BHLHA15/EGR3/FGF10/FOXC2/FLNB/FLOT2/SYNE1/VGLL2/MTOR/BMP10/TMOD4/SOX8/NRG1/HLX/ID3/IGF1/IGF2/IL6/ISL1/ITGA7/LMNA/SMAD3/MEF2D/MEOX2/MYL2/NFATC3/NOV/NRAS/NTF3/LEF1/SIRT6/PITX2/PLAGL1/BIN3/CCL17/STRA6/BMP4/SLC8A1/SOX9/SUPT6H/ZEB1/ACTC1/TWIST1/WNT10B/TMEM204/CALR/CAST/CASQ1/HOPX	55
GO:0009887	organ morphogenesis	78/901	884/17046	5.38E-06	0.00042	0.00033	TBR1/GJB6/COL11A1/COMP/ADM/FAM101A/CTGF/EGFR/EPHB4/ESR1/FGF10/FOXL1/FOXC2/BMP10/GNAS/FLVCR1/SOX8/NRG1/HLX/HOXB3/HOXC4/HOXD3/ID3/RSPO2/FMN1/IGF1/IGF2/CYR61/IL6/AQP5/INHBA/ISL1/HES5/SMAD3/MEF2D/LHX8/MYL2/NPPC/LEF1/SIRT6/ATP8A2/PITX2/PML/BNC2/CHRNA9/LIMS2/PARVA/IFT122/CSGALNACT1/MAPK3/MAP2K2/HTRA1/ACTA2/BGLAP/CCL11/STRA6/SFRP2/GZF1/BMP4/SLC8A1/BMPR1B/SLIT1/SOX9/ZEB1/ACTC1/TGM2/TLE3/TNFAIP3/PHLDA2/TWIST1/WNT10B/PAX8/FZD5/COL18A1/KDM2B/TP63/ALDH1A2/MICAL2	78
GO:0008360	regulation of cell shape	21/901	132/17046	5.66E-06	0.00043	0.00034	PDPN/COCH/EPB41L3/ARHGEF18/GAS2/IL6/ITGA7/ITGB2/KDR/PALM/FBLIM1/PALMD/PARVA/CDC42SE1/ERMN/PXN/CCL11/LST1/TTBK1/GAS7/LIMD1	21
GO:0032640	tumor necrosis factor production	17/901	93/17046	6.54E-06	0.00049	0.00038	SPON2/CIDEA/GSTP1/HLA-E/ISL1/LGALS9/TLR9/POMC/TRIM27/NOD2/BPI/TNFAIP3/TWIST1/CCR2/ZC3H12A/FADD/ADIPOQ	17
GO:0016337	single organismal cell-cell adhesion	62/901	657/17046	6.57E-06	0.00049	0.00038	CDH3/CDH9/PDPN/PKP3/MAP3K8/CSTA/CYLD/DDOST/EGFR/EGR3/FGA/NFASC/FLOT2/MTOR/GNAS/HLA-DOA/HLA-DPA1/HLA-E/HLX/ZC3H12D/IGF1/IGF2/CYR61/IL1RN/IL6/IRF1/ITGA7/ITGB2/ITGB7/JUP/LCK/LCP1/LGALS9/SMAD3/NFATC3/NOV/LEF1/PIK3CG/PKHD1/IL20RB/APBB1IP/FBLIM1/LIMS2/PARVA/PAG1/TENM2/NOD2/BMP4/SOX9/STK10/ZEB1/TNXB/CCR2/TNFRSF4/ZAP70/FZD5/SLA2/FADD/ESAM/RSAD2/CD8A/ADIPOQ	62
GO:0043067	regulation of programmed cell death	106/901	1314/17046	6.64E-06	0.00049	0.00038	EGLN2/CARD16/CIDEA/ANKRD9/COMP/MAP3K8/ADM/IL31RA/CTGF/CYLD/DDB1/EGFR/EGR3/ESR1/FGA/FGF10/ACIN1/FOXC2/FOXO1/DIP2A/ARHGEF18/GLS2/BMP10/GPER1/GSTP1/GZMA/SOX8/NRG1/HSP90AB1/ID3/IGF1/CYR61/IL1RN/IL6/INHBA/ISL1/KDR/LCK/ARHGDIA/LGALS9/LMNA/SMAD3/MAP3K1/MITF/MPZ/NTF3/PAFAH2/ARHGEF3/PARK2/UTP11L/LEF1/ANGPT4/PIK3CG/PKHD1/PML/LIMS2/PROC/PSMB4/PAK6/PSMD7/PTGFR/PLEKHG5/MARK4/CCAR2/RASGRF2/SCT/NOD2/SFRP2/SGK1/BMP4/BMPR1B/BOK/SOX9/STK3/STK10/ACTC1/TERF1/TGM2/TNFAIP3/TRAF1/TRAF5/TWIST1/TNFRSF4/WNT10B/YWHAG/PAX8/CARD14/BCL2L14/CPEB4/COL18A1/CALR/CAST/KDM2B/SCIN/TP63/RUNX3/IRS2/ACTN1/CRADD/FADD/ALDH1A2/SPHK1/MAP3K6/AURKB/ADIPOQ/RAPGEF2	106
GO:0008283	cell proliferation	135/901	1775/17046	7.16E-06	0.00052	0.00041	ABI1/CDH3/CDH13/CDKN1C/SPEG/TCIRG1/TACC2/PDPN/GJB6/LECT1/ESM1/B4GALT7/PDAP1/ADM/IL31RA/CTGF/DMBT1/DPH1/EGFR/EGR3/EML1/EPHA1/ESR1/FGF10/VASH1/FOXC2/FOXO1/AKR1B1/LARP1/MORC3/MTOR/TENM4/FBXO2/BMP10/GPER1/GSTP1/ANXA2/SOX8/NRG1/HLA-DPA1/HLA-E/HLX/HMGA1/NR4A1/TFAP2E/ZC3H12D/IGF1/IGF2/CYR61/IL6/IL12RB2/IL15RA/INHBA/IRF1/ISL1/ITGB2/JUP/KDR/HES5/LCK/LGALS9/SMAD3/MCC/MITF/NEU1/NOV/NPPC/NRAS/NTF3/LEF1/CEND1/SIRT6/ATP8A2/PIK3CG/PITX2/PKHD1/PLA2G2A/PML/RIPPLY3/IL20RB/CNOT11/LIMS2/IFT122/CSGALNACT1/PRKD1/MAP2K2/PRKRIR/HTRA1/SLURP1/PTGFR/TRIM27/RPA3/S100A6/CCL11/NOD2/SFRP2/SGK1/BMP4/BMPR1B/BOK/SOX9/STK3/BST2/ZEB1/TNFAIP3/TRAF5/TWIST1/CCR2/TNFRSF4/WNT10B/ZAP70/FZD5/LST1/COL18A1/CALR/RETNLB/KDM2B/IFITM1/SCIN/CDK10/RUNX1/TP63/RUNX3/IRS2/FADD/TNFRSF11A/ALDH1A2/SPHK1/SKAP2/PRC1/ADIPOQ/LY86/RAPGEF2/CD79A/FGF19	135

GO:0030029	actin filament-based process	55/901	561/17046	7.31E-06	0.00052	0.00041	ABI1/FRMD6/FAM101A/CTGF/EPHA1/EPHA3/PHACTR1/FGF10/PPM1E/MSRB2/LIMCH1/EPB41L3/FLNB/ARHGEF18/MTOR/PLEK2/BMP10/TMOD4/FMN1/LCP1/LLGL1/SMAD3/MAP3K1/MYH4/MYL2/NEDD9/ATP1A2/NTF3/PARK2/SSH1/PARVA/TTC17/BIN3/PAK6/ACTR3B/ERMN/TRIM27/CCL11/PARVG/MICAL1/BST2/ACTC1/TNXB/CALR/CAPZB/SH3BGRL3/ANTXR1/CASQ1/GAS7/SCIN/ACTN1/TRIP10/ARHGEF10/MICAL2/IQSEC1	55
GO:1903555	regulation of tumor necrosis factor superfamily cytokine production	17/901	94/17046	7.60E-06	0.00054	0.00042	SPON2/CIDEA/GSTP1/HLA-E/ISL1/LGALS9/TLR9/POMC/TRIM27/NOD2/BPI/TNFAIP3/TWIST1/CCR2/ZC3H12A/FADD/ADIPOQ	17
GO:0042221	response to chemical	260/901	3882/17046	7.65E-06	0.00054	0.00042	CDH3/CDH13/KCNMB2/CDKN1C/TCIRG1/SPON2/TBR1/GJB6/NPFFR2/ADCY3/TMED10/LECT1/CHGA/CHI3L1/ERLIN2/EGLN2/CHRNA1/CHRNA2/CHRNA5/CIDEA/AP3S1/CCR1/CNP/COL9A3/ADM/IL31RA/OR2A14/CPS1/TRPM6/PARP4/CTGF/ADRB3/CYP11A1/CITED4/DDOST/ZNF366/BHLHA15/DNMT3A/ABAT/DRD4/AGXT/EFNA2/EGFR/EGR3/EIF4G1/EPHA1/EPHA3/EPHB4/ESR1/FGA/FGF10/RASA3/PPM1E/SBNO2/FOXC2/FOXO1/AKR1B1/SPG20/NFASC/FLNB/MLC1/NUP210/NEDD4L/ARHGEF18/MTOR/STEAP2/FBXO2/GATM/GJA3/FGF22/NPTN/GJB2/BMP10/GNAS/GPER1/FFAR2/GRB10/GSTP1/GUCY1A3/HAS1/SERPIND1/KCNIP2/NRG1/HLA-B/HLA-DPA1/HLA-E/HLA-F/NR4A1/HPCA/HRH1/HSD17B2/HSPA1L/HSP90AA1/HSP90AB1/HTR3A/HTR5A/IGF2/CYR61/LCE1D/IL1R1/IL1RN/IL6/IL10RA/AQP2/IL11RA/IL12RB2/IL15RA/IL16/INHBA/IRF1/AQP9/ISL1/ITGB2/ITIH4/JUP/KCNH2/KCNJ8/KCNMB1/KDR/ACAT1/IPO5/HES5/AFF3/OR2A5/LCK/ARHGDIA/LGALS9/LHCGR/LMNA/LMO2/LOX/LTB/LTBP1/SMAD3/ME1/MAP3K1/MOV10/MT1A/ATP1A2/NFATC3/NOV/NPPC/NRAS/NTF3/OAS2/OPRL1/OR2C1/OR3A2/SLC22A18/P2RY6/IL21R/ARHGEF3/PARK2/LEF1/ANGPT4/PGAM2/PIK3CG/PITX2/PKM/PRKAG3/PML/IL20RB/TLR9/TREM1/SSH1/PON1/CYP2W1/PPP1CB/PPP1CC/SLC47A1/SLC30A10/CHRNA9/SYBU/PARVA/PRKAR1B/LMBRD1/PRKD1/MAPK3/MAP2K2/HTRA1/SLAMF8/PSMB4/RGMA/TRPC7/PSMD7/PTGFR/PLEKHG5/TENM2/PTPRE/PXN/RASGRF2/RIT2/BGLAP/CCL11/CCL17/NOD2/SFRP2/CXCR5/BMP4/SLC8A1/SLC9A3/BMPR1B/SLIT1/SOX9/SRP68/STAT2/BST2/VAMP2/TCEB2/ZEB1/ACTC1/TERF1/TIMP3/TLR5/TNFAIP3/TNFRSF1A/TRPC4/TRPC6/TRPM2/TWIST1/CCR2/TNFRSF4/WNT10B/CACNA1E/CACNB2/PAX8/CXCR4/FZD5/RAB7A/CARD14/TMEM204/ZC3H12A/CPEB4/COL18A1/CALR/NR0B2/CASQ1/IL1F10/TRIM63/MGARP/RAE1/IFITM1/RUNX1/RUNX3/IRS2/FADD/TNFRSF11A/ALDH1A2/SPHK1/HSPB3/CCRL2/ZFAND2A/RSAD2/ADIPOQ/LY86/MTL5/NUP93/RAPGEF2/FGF19/NR1H4	260
GO:0048523	negative regulation of cellular process	257/901	3831/17046	7.85E-06	0.00054	0.00043	ABI1/CDH13/FARP1/CDKN1C/SPEG/TRDN/C1D/ZBTB18/CELF1/GJB6/LECT1/CHGA/ERLIN2/B4GALT7/CARD16/CIDEA/ANKRD9/SEZ6/TNFAIP8L1/COMP/ADM/IL31RA/HUS1B/FAM101A/APCDD1/CSTA/CTGF/SMYD1/CYLD/DDB1/RNF168/ZNF366/BHLHA15/NLRP6/DLG2/DNMT3A/DRD4/EGFR/EGR3/PATL2/A2M/ELK4/EPHA1/ESR1/FGA/FGF10/FHIT/RASA3/PPM1E/VASH1/SBNO2/ACIN1/FOXC2/FOXO1/SPG20/NEDD4L/PPP1R13B/RYBP/MORC3/MTOR/GABBR1/RGS22/GAS2/FBXO2/SACS/PABPC1/DNAJC2/DKK3/VPS4A/BMP10/GPER1/GRB10/DNAJC15/GSTP1/TMOD4/GPR132/GZMA/ANXA2/HAS1/SERPIND1/SOX8/NRG1/ANXA13/HLX/HMGA1/NR4A1/ACACB/HPCA/HOXB3/HOXC6/ACADL/HSP90AB1/ID3/ZC3H12D/COL28A1/IGF1/CYR61/IL1RN/IL6/INHBA/IRF1/ISL1/ITIH3/ITIH4/HILS1/KCNH2/KDR/KIF25/HES5/STMN1/LCK/ARHGDIA/LGALS9/LMNA/LTBP1/SMAD3/MCC/MFI2/MITF/MPZ/MYL2/NUBP1/ATP1A2/NOV/NPPC/NTF3/OPRL1/PAFAH2/ATP5B/PALM/PARK2/SPOCK3/LEF1/CEND1/ANGPT4/PDE4C/SIRT6/ATP8A2/PI3/PIK3CG/PITX2/PKHD1/PLA2G2A/PLAGL1/PRKAG3/PML/RIPPLY3/IL20RB/TLR9/PPP1CB/PPP1CC/PRMT6/DNAJC17/FANCI/LIMS2/PRKAR1B/IFT122/LMBRD1/PAG1/PRKD1/PRKRIR/PROC/MASP1/HTRA1/PSMB4/PSMD7/SLURP1/PTGFR/TENM2/GATAD2B/METTL14/CCAR2/PTPRE/CREBZF/FAM60A/TRIM27/RGS12/SCT/CCL17/NOD2/SFRP2/GZF1/BMP4/ZNF649/SLC8A1/BMPR1B/SLIT1/SOX9/BPI/STK3/SUPT6H/BST2/TBP/ZEB1/ACTC1/TERF1/TIMP3/TNFAIP3/TWIST1/CCR2/TNFRSF4/WNT10B/YWHAG/ZNF177/PAX8/FZD5/RAB7A/CARD14/LST1/NLRX1/ZC3H14/ZC3H12A/RAB11FIP1/CPEB4/COL18A1/CALR/SLIRP/CAST/CAPZB/SCRT1/HIST1H3A/SLA2/NR0B2/CASQ1/HOPX/SPINK7/KDM2B/LOXL3/CBX2/IFITM1/GAS7/SCIN/CDK10/RUNX1/TP63/RUNX3/SERPINA6/IRS2/ACTN1/CRADD/FADD/ALDH1A2/SPHK1/SKAP2/LIMD1/PIAS2/CBFA2T2/RSAD2/AURKB/DAPL1/ADIPOQ/H2AFY/N4BP1/RAPGEF2/ULK2/FGF19/NR1H4	257
GO:0042981	regulation of apoptotic process	105/901	1305/17046	8.25E-06	0.00056	0.00044	EGLN2/CARD16/CIDEA/ANKRD9/COMP/MAP3K8/ADM/IL31RA/CTGF/CYLD/DDB1/EGFR/EGR3/ESR1/FGA/FGF10/ACIN1/FOXC2/FOXO1/DIP2A/ARHGEF18/GLS2/BMP10/GPER1/GSTP1/GZMA/SOX8/NRG1/HSP90AB1/ID3/IGF1/CYR61/IL1RN/IL6/INHBA/ISL1/KDR/LCK/ARHGDIA/LGALS9/LMNA/SMAD3/MAP3K1/MITF/MPZ/NTF3/PAFAH2/ARHGEF3/PARK2/UTP11L/LEF1/ANGPT4/PIK3CG/PKHD1/PML/LIMS2/PROC/PSMB4/PAK6/PSMD7/PTGFR/PLEKHG5/CCAR2/RASGRF2/SCT/NOD2/SFRP2/SGK1/BMP4/BMPR1B/BOK/SOX9/STK3/STK10/ACTC1/TERF1/TGM2/TNFAIP3/TRAF1/TRAF5/TWIST1/TNFRSF4/WNT10B/YWHAG/PAX8/CARD14/BCL2L14/CPEB4/COL18A1/CALR/CAST/KDM2B/SCIN/TP63/RUNX3/IRS2/ACTN1/CRADD/FADD/ALDH1A2/SPHK1/MAP3K6/AURKB/ADIPOQ/RAPGEF2	105
GO:0000902	cell morphogenesis	108/901	1352/17046	8.33E-06	0.00056	0.00044	ABI1/FARP1/SPON2/PDPN/TBR1/LECT1/FRMD6/CNP/COL9A3/SCLT1/SH3D19/CYLD/COCH/EFNA2/EGFR/TMEM17/UNC13D/EPHA1/EPHA3/EPHB4/FGA/FGF10/RASA3/BTBD3/SPG20/NFASC/EPB41L3/FLNB/NEDD4L/ARHGEF18/MAPK8IP2/GAS2/FGF22/NRG1/HSP90AA1/HSP90AB1/FMN1/BARHL2/IL6/ISL1/ITGA7/ITGB2/ITGB7/KDR/STMN1/ARHGDIA/LLGL1/SMAD3/MAP3K1/MFI2/NRAS/NTF3/PALM/PARK2/LEF1/ATP8A2/PKHD1/SSH1/FBLIM1/PALMD/PARVA/IFT122/BIN3/MAPK3/MAP2K2/CDC42SE1/PSMB4/RGMA/TRPC7/PSMD7/TENM2/ERMN/PXN/RASGRF2/S100A4/S100A6/CCL11/SFRP2/DNAI2/SGK1/TMEM237/VPS33A/BMP4/BMPR1B/SLIT1/SOX9/TRPC4/TRPC6/TWIST1/CACNB2/PAX8/LST1/C6orf25/COL18A1/CALR/CAPZB/ANTXR1/PARD6B/TTBK1/LOXL3/GAS7/RUNX3/IRS2/ACTN1/LIMD1/RAPGEF2/ULK2/FGF19	108
GO:0030334	regulation of cell migration	55/901	568/17046	1.05E-05	0.0007	0.00055	CDH13/CCR1/EGFR/EPHA1/SPATA13/FGF10/VASH1/FOXC2/BMP10/GPER1/HAS1/IGF1/CYR61/IL1RN/IL6/KDR/LAMA3/LGALS9/LMNA/SMAD3/MCC/MAP3K1/NOV/NTF3/P2RY6/LEF1/ANGPT4/PITX2/ROBO4/ELP3/PRKD1/FAM60A/CCL11/NOD2/SFRP2/SGK1/BMP4/SLC8A1/SOX9/STK10/BST2/PHLDA2/CCR2/PTP4A1/COL18A1/CALR/SH3BGRL3/SCRT1/PARD6B/IFITM1/IRS2/FADD/SPHK1/ADIPOQ/RAPGEF2	55

GO:0002682	regulation of immune system process	110/901	1391/17046	1.07E-05	0.00071	0.00056	ABI1/TANK/KLRG1/SPON2/HCST/CCR1/MAP3K8/IL31RA/CYLD/COCH/NLRP6/DMBT1/EGFR/EGR3/A2M/UNC13D/FCGR2A/FGF10/RASA3/ACIN1/FOXO1/FLOT2/PUM2/MTOR/SLC37A4/FGF22/GNAS/GPER1/FFAR2/NRG1/HLA-B/HLA-DOA/HLA-DPA1/HLA-E/HLA-F/HLX/NR4A1/HSP90AA1/HSP90AB1/ZC3H12D/IGF1/IGF2/IL6/INHBA/IRF1/ITGB2/ITGB7/KDR/HES5/LCK/LGALS9/SMAD3/MAP3K1/MITF/MOV10/NFATC3/NOV/NRAS/LEF1/PML/IL20RB/TLR9/TREM1/PAG1/MAPK3/MAP2K2/MASP1/HTRA1/PSMB4/PSMD7/PTPRE/RASGRF2/TRIM27/BGLAP/NOD2/BMP4/SOX9/BPI/STAT2/STK10/SUPT6H/BST2/ZEB1/TLR5/TNFAIP3/CCR2/TNFRSF4/ZAP70/CA7/FZD5/LST1/NLRX1/ZC3H12A/CALR/UNC93B1/HIST1H3A/SLA2/IFITM1/SCIN/RUNX1/IRS2/FADD/SKAP2/RSAD2/CD8A/ADIPOQ/NUP93/RAPGEF2/CD79A/FGF19	110
GO:0050678	regulation of epithelial cell proliferation	32/901	265/17046	1.12E-05	0.00073	0.00058	CDH3/CDH13/CDKN1C/LECT1/EGFR/EGR3/FGF10/VASH1/MTOR/NR4A1/IGF1/IL6/KDR/SMAD3/MCC/PLA2G2A/LIMS2/IFT122/PRKD1/HTRA1/SLURP1/CCL11/NOD2/SFRP2/BMP4/SOX9/TNFAIP3/TWIST1/WNT10B/TP63/RUNX3/ALDH1A2	32
GO:2000145	regulation of cell motility	57/901	599/17046	1.22E-05	0.00079	0.00062	CDH13/CCR1/EGFR/EPHA1/SPATA13/FGF10/VASH1/FOXC2/BMP10/GPER1/HAS1/IGF1/CYR61/IL1RN/IL6/KDR/LAMA3/LGALS9/LMNA/SMAD3/MCC/MAP3K1/NOV/NTF3/P2RY6/LEF1/ANGPT4/PITX2/ROBO4/ELP3/PRKD1/MAP2K2/FAM60A/CCL11/NOD2/SFRP2/SGK1/BMP4/SLC8A1/SOX9/STK10/BST2/PHLDA2/TWIST1/CCR2/PTP4A1/COL18A1/CALR/SH3BGRL3/SCRT1/PARD6B/IFITM1/IRS2/FADD/SPHK1/ADIPOQ/RAPGEF2	57
GO:0006796	phosphate-containing compound metabolic process	196/901	2808/17046	1.33E-05	0.00085	0.00067	AKT3/ABI1/GNE/FARP1/CDKN1C/SPEG/BCKDK/TCIRG1/HCST/NPFFR2/ADCY3/CHI3L1/ACOT7/ALPK2/CCR1/CNP/APOA1BP/MAP3K8/ADM/IL31RA/UBLCP1/CPS1/TRPM6/CTGF/PPM1L/MBOAT1/ADRB3/FITM1/ADAL/NLRP6/DLG2/DRD4/EGFR/ENO2/ADCK5/EPHA1/EPHA3/EPHB4/FGA/FGF10/FHIT/RASA3/PPM1E/FOXO1/MORC3/MAPK8IP2/TSSK2/MTOR/GABBR1/GAK/GAPDHS/RPS6KC1/FGF22/NPTN/AMPD2/PDE7B/AMPD3/BMP10/GNAS/PIGW/THEM5/GPER1/DOK7/GRB10/DNAJC15/GSTP1/GUCY1A3/NME7/ANXA2/NRG1/HK1/ACACB/HPCA/HRH1/HSP90AB1/DUPD1/NME9/IGF1/IGF2/CYR61/IL1RN/IL6/IL12RB2/INHBA/INPP5A/ISL1/ITGB2/KCNH2/KDR/HES5/LCK/LDLR/LGALS9/LHCGR/SMAD3/MC2R/ME1/ME2/MAP3K1/MGAT1/MOCS1/NUDT1/MYH4/NDUFB4/ATP1A2/NPPC/NRAS/NTF3/OAS2/OPRL1/ATP5B/PALM/PARK2/ANGPT4/PDE4C/PDE7A/SIRT6/PDE6B/PGAM2/PIGC/PIK3CG/PKHD1/PKM/PLA2G2A/PRKAG3/PML/RIPK4/TLR9/SSH1/PON1/LPCAT2/PPP1CB/PPP1CC/ELP3/PPP2R2B/SMPD3/VAC14/PRKAR1B/CSGALNACT1/PRKD1/MAPK3/MAP2K2/MRAP/SLAMF8/PSMB4/PAK6/PSMD7/MARK4/PTPRE/PXN/RASGRF2/TRIM27/SCT/CCL11/CCL17/NOD2/SFRP2/SGK1/CERK/BMP4/BMPR1B/SOX9/STK3/STK10/TNFAIP3/TNFRSF1A/TNXB/TWIST1/CCR2/TNFRSF4/UPP1/YWHAG/ZAP70/PTP4A1/CXCR4/FZD5/CARD14/TTBK1/CDK10/KMO/RUNX3/IRS2/TNFRSF11A/STK19/SYNJ2/SPHK1/LIMD1/MAP3K6/AURKB/ADIPOQ/H2AFY/ENTPD3/RAPGEF2/ULK2/LPGAT1/FGF19	196
GO:0071706	tumor necrosis factor superfamily cytokine production	17/901	98/17046	1.35E-05	0.00086	0.00067	SPON2/CIDEA/GSTP1/HLA-E/ISL1/LGALS9/TLR9/POMC/TRIM27/NOD2/BPI/TNFAIP3/TWIST1/CCR2/ZC3H12A/FADD/ADIPOQ	17
GO:1902531	regulation of intracellular signal transduction	115/901	1479/17046	1.42E-05	0.00088	0.00069	CDH13/FARP1/RCAN2/HCST/NPFFR2/CHI3L1/CCR1/SEZ6/TNFAIP8L1/MAP3K8/IL31RA/MIB2/CTGF/CYLD/ADRB3/NLRP6/DRD4/EGFR/A2M/ESR1/SPATA13/FGA/FGF10/RASA3/FOXO1/AKR1B1/RHOBTB2/PSD3/PUM2/ARHGEF18/MAPK8IP2/MTOR/ALS2CL/FGF22/CYTH4/BMP10/GNAS/GPER1/GSTP1/NRG1/IGF1/IGF2/CYR61/IL1RN/IL6/INHBA/ISL1/KDR/HES5/LCK/ARHGDIA/LGALS9/LHCGR/MAP3K1/PLEKHG7/NOV/NRAS/NTF3/ARHGEF3/PARK2/PIK3CG/PKHD1/PLA2G2A/TLR9/ZDHHC13/ARHGEF10L/LMBRD1/PRKD1/MAPK3/MAP2K2/PSMB4/PAK6/PSMD7/PLEKHG5/CCAR2/PXN/RASGRF2/S100A4/CCL11/CCL17/NOD2/SFRP2/ARHGAP9/BMP4/SOX9/STK3/BST2/TNFAIP3/TNFRSF1A/TNXB/TRAF5/TWIST1/ZAP70/CXCR4/FZD5/CARD14/NLRX1/SLA2/CDK10/IRS2/FADD/TNFRSF11A/SPHK1/LIMD1/STARD13/MAP3K6/CD8A/TRIP10/ADIPOQ/ARHGAP29/ARHGEF10/RAPGEF2/TELO2/IQSEC1/FGF19	115
GO:0048584	positive regulation of response to stimulus	138/901	1848/17046	1.42E-05	0.00088	0.00069	ABI1/CDH3/TANK/TSPAN5/CDH13/CDKN1C/SPON2/HCST/ESM1/CHI3L1/CCR1/MAP3K8/IL31RA/MIB2/MPP7/CTGF/CYLD/ADRB3/RNF168/COCH/NLRP6/DMBT1/DRD4/EGFR/A2M/FCGR2A/FGA/FGF10/RASA3/FOXC2/AKR1B1/LARP1/PUM2/MAPK8IP2/MTOR/FGF22/NPTN/BMP10/GNAS/GPER1/FFAR2/GRB10/NRG1/HK1/HLA-B/HLA-DPA1/HLA-E/HLX/HMGA1/HSP90AA1/HSP90AB1/RSPO2/IGF1/IGF2/CYR61/IL1RN/IL6/IL16/INHBA/IRF1/ISL1/ITGB2/JUP/KDR/HES5/LCK/LGALS9/LHCGR/SMAD3/MAP3K1/MFNG/NFATC3/NOV/NRAS/NTF3/PARK2/PIK3CG/PLA2G2A/PML/TLR9/ZDHHC13/LIMS2/PAG1/PRKD1/MAPK3/MAP2K2/MASP1/PSMB4/PSMD7/PLEKHG5/CCAR2/PXN/RASGRF2/S100A4/CCL11/CCL17/NOD2/STRA6/SFRP2/BMP4/BMPR1B/SOX9/STK3/BST2/TLR5/TNFAIP3/TNFRSF1A/TRAF5/CCR2/WNT10B/YWHAG/ZAP70/CXCR4/FZD5/CARD14/BCL2L14/NLRX1/CALR/UNC93B1/SLA2/HOPX/CDK10/TP63/RUNX3/IRS2/CRADD/FADD/TNFRSF11A/SPHK1/SKAP2/MAP3K6/RSAD2/CD8A/ADIPOQ/LY86/RAPGEF2/CD79A/FGF19	138
GO:0001501	skeletal system development	48/901	478/17046	1.49E-05	0.00092	0.00072	ABI1/CDKN1C/DMRT2/LECT1/CHI3L1/SLC38A10/COL11A1/COMP/FAM101A/CTGF/FOXC2/BMP10/GNAS/FLVCR1/ANXA2/HOXB3/HOXC4/HOXC5/HOXC6/HOXD3/RSPO2/FMN1/IGF1/IGF2/CYR61/HES5/SMAD3/MEF2D/NOV/NPPC/PITX2/CYTL1/BNC2/CSGALNACT1/MAPK3/BGLAP/SFRP2/BMP4/BMPR1B/SOX9/ZEB1/TWIST1/WNT10B/C6orf25/SCIN/RUNX1/TP63/RUNX3	48
GO:0042592	homeostatic process	112/901	1434/17046	1.53E-05	0.00093	0.00073	CDH3/TCIRG1/TRDN/ZBTB18/GJB6/ADCY3/EGLN2/CHRNA1/CIDEA/CLN5/CCR1/ZG16B/ADM/IL31RA/CPS1/CTGF/ADRB3/DDB1/BHLHA15/ABAT/DRD4/ESR1/ACIN1/FOXO1/AKR1B1/NEDD4L/SLC37A4/DFNB31/STEAP2/NPTN/AMPD2/AMPD3/GNAS/GPER1/FFAR2/FLVCR1/GSTP1/ANXA6/HK1/ACADL/NME9/IGF1/IL1R1/IL1RN/IL6/AQP2/AQP5/INHBA/AQP9/KCNH2/KDR/LCK/LDLR/LGALS9/MFI2/NUBP1/ATP1A2/NOV/OPRL1/ATP5B/PARK2/SIRT6/PDE6B/PIK3CG/PKHD1/PML/IL20RB/TLR9/CYTL1/POMC/SLC30A10/CHRNA9/SYBU/PRKAR1B/PRKD1/SLAMF8/TRPC7/RFC2/RPA3/BGLAP/CCL11/ABHD4/NOD2/SGK1/BMP4/SLC4A1/SLC8A1/SLC9A3/SOX9/TCEA1/TERF1/TGM2/TNFAIP3/TRPC4/TRPC6/CCR2/CA7/CACNA1E/CXCR4/RAB7A/CALR/SH3BGRL3/ATP13A4/CASQ1/TP63/IRS2/FADD/TNFRSF11A/LDB2/SMDT1/ADIPOQ/MTL5	112
GO:0001525	angiogenesis	42/901	398/17046	1.57E-05	0.00094	0.00074	CDH13/LECT1/ESM1/CHI3L1/ADM/CTGF/EGR3/EPHA1/EPHB4/FGF10/VASH1/FOXC2/ANXA2/NR4A1/HOXB3/CYR61/IL6/ISL1/KDR/MEOX2/NFATC3/NOV/ATP5B/LEF1/ANGPT4/PIK3CG/PITX2/PML/ROBO4/PARVA/PRKD1/CCL11/SFRP2/BMP4/TNFAIP3/TWIST1/CCR2/FZD5/ZC3H12A/COL18A1/RUNX1/SPHK1	42
GO:0001503	ossification	38/901	348/17046	1.84E-05	0.0011	0.00086	CCR1/COL11A1/FAM101A/CTGF/EGFR/SBNO2/FOXC2/GNAS/SOX8/ID3/RSPO2/IGF1/IGF2/CYR61/IL6/SMAD3/MEF2D/NPPC/ATP5B/LEF1/CSGALNACT1/PRKD1/RDH14/BGLAP/SFRP2/BMP4/SLC8A1/BMPR1B/SOX9/TWIST1/WNT10B/IFITM1/TP63/RUNX3/TNFRSF11A/LIMD1/PIAS2/RSAD2	38

GO:0008285	negative regulation of cell proliferation	57/901	608/17046	1.88E-05	0.00111	0.00087	ABI1/CDH13/CDKN1C/SPEG/GJB6/LECT1/B4GALT7/ADM/FGF10/VASH1/MORC3/FBXO2/GPER1/GSTP1/HMGA1/ZC3H12D/IGF1/IL6/INHBA/IRF1/LGALS9/SMAD3/MCC/NOV/NPPC/CEND1/SIRT6/ATP8A2/PLA2G2A/PML/RIPPLY3/IL20RB/LIMS2/IFT122/PRKRIR/SLURP1/SFRP2/BMP4/BMPR1B/SOX9/STK3/ZEB1/TNFAIP3/WNT10B/FZD5/LST1/COL18A1/KDM2B/IFITM1/SCIN/CDK10/RUNX1/RUNX3/ALDH1A2/SKAP2/ADIPOQ/RAPGEF2	57
GO:0031323	regulation of cellular metabolic process	340/901	5352/17046	1.92E-05	0.00112	0.00088	ABI1/CDH3/ZNF783/CDH13/MBNL2/FARP1/CDKN1C/C1D/ZBTB18/DMRT2/CELF1/TBR1/NPFFR2/ADCY3/PNRC1/TMED10/HNRNPUL1/CHI3L1/ERLIN2/PSIP1/EGLN2/CARD16/ZBED9/CIDEA/CCR1/SLC51B/MAP3K8/ZFP42/ADM/IL31RA/ZNF358/CSTA/ZNF738/CTGF/SMYD1/SH3D19/CYLD/ADRB3/ESCO2/ZNF782/ZNF709/ZNF781/CITED4/RNF168/ZNF366/BHLHA15/NLRP6/DLG2/DNMT3A/DRD4/ECE1/EEF2/EGFR/EGR3/PATL2/EIF4G1/A2M/ELK4/EPHA1/ESR1/SP8/FGA/FGF10/FHIT/XRN2/RASA3/PPM1E/SBNO2/TRAK1/MSRB2/ACIN1/FOXL1/FOXC2/FOXO1/SPG20/NEDD4L/LARP1/PUM2/RYBP/MAPK8IP2/VGLL2/MTOR/GABBR1/RNF144B/ZNF549/PNKD/FBXO2/GAPDHS/PABPC1/DNAJC2/FGF22/NPTN/DKK3/BMP10/ZNF638/GNAS/ZNF311/ZNF844/GPER1/DOK7/GRB10/ZBTB44/DNAJC15/GSTP1/GTF2B/BRF1/GUCY1A3/GZMA/ANXA2/SERPIND1/SOX8/NRG1/HK1/HLX/HMGA1/NR4A1/ACACB/HPCA/HOXB3/HOXC4/HOXC5/HOXC6/HOXD3/HRH1/ACADL/HSP90AA1/HSP90AB1/TFAP2E/ID3/COL28A1/BARHL2/IGF1/IGF2/CYR61/IL1RN/IL6/IL16/FOXK2/INHBA/IRF1/ISL1/ITGB2/ITIH3/ITIH4/JUP/USP50/HILS1/KDR/KIF25/HES5/AFF3/LCK/LDLR/LGALS9/LHCGR/LMNA/LMO2/LTB/SMAD3/MC2R/ME1/ME2/MEF2D/MAP3K1/MEOX1/MEOX2/MFI2/MITF/LHX8/MOV10/NFATC3/NFYB/NHLH2/NPPC/NRAS/NTF3/OPRL1/PALM/PARK2/SPOCK3/LEF1/PRR16/ANGPT4/SIRT6/PGAM2/PI3/PIK3CG/PITX2/PKHD1/PLA2G2A/PLAGL1/PML/RIPPLY3/RIPK4/TLR9/CYTL1/POMC/SSH1/POU2AF1/BNC2/MED18/BANP/PPP1CB/PIWIL2/ELP3/PRMT6/DNAJC17/ZNF532/PPP2R2B/FANCI/CNOT11/VAC14/PRKAR1B/CISD1/PRKD1/MYNN/MAPK3/MAP2K2/MRAP/PRMT8/MASP1/HTRA1/SLAMF8/PSMB4/PAK6/ARNTL2/RGMA/PRDM11/PSMD7/TENM2/GATAD2B/METTL14/CCAR2/PXN/CREBZF/RASGRF2/TRIM27/SCT/CCL11/CCL17/NPAS3/NOD2/SFRP2/TRA2B/GZF1/SGK1/BMP4/ZNF649/BMPR1B/BRD9/ZSCAN18/BOK/SOX9/STAT2/STK3/STK10/SUPT6H/BST2/TAF4B/TBP/TCEA1/TCEB2/ZEB1/TEAD3/TERF1/TIMP3/TLE3/TLR5/TNFAIP3/TNFRSF1A/TNXB/TRAF1/TRAF5/PHLDA2/TWIST1/CCR2/TNFRSF4/UCP1/VARS/WNT10B/YWHAG/ZNF7/ZNF124/ZNF177/PAX8/CXCR4/FZD5/RAB7A/CARD14/ZNF665/ZC3H14/ZNF606/ZC3H12A/CPEB4/ZNF436/CALR/SLIRP/CAST/SCRT1/HIST1H3A/SLA2/ZNF397/NR0B2/HOPX/SPINK7/KDM2B/LOXL3/CBX2/GAS7/CDK10/RUNX1/TP63/RUNX3/SERPINA6/IRS2/ACTN1/CRADD/FADD/TNFRSF11A/SPHK1/BUD31/CCNA1/LIMD1/PIAS2/ZFAND2A/MAP3K6/LDB2/CBFA2T2/AURKB/DAPL1/ADIPOQ/H2AFY/MICAL2/N4BP1/VGLL4/RAPGEF2/ULK2/ZBTB39/FGF19/NR1H4	340
GO:0010941	regulation of cell death	109/901	1395/17046	1.98E-05	0.00114	0.0009	EGLN2/CARD16/CIDEA/ANKRD9/COMP/MAP3K8/ADM/IL31RA/CTGF/CYLD/DDB1/DNMT3A/EGFR/EGR3/ESR1/FGA/FGF10/ACIN1/FOXC2/FOXO1/DIP2A/ARHGEF18/GLS2/BMP10/GPER1/GSTP1/GZMA/SOX8/NRG1/HSP90AB1/ID3/IGF1/CYR61/IL1RN/IL6/INHBA/ISL1/KDR/LCK/ARHGDIA/LGALS9/LMNA/SMAD3/MAP3K1/MITF/MPZ/NOV/NTF3/PAFAH2/ARHGEF3/PARK2/UTP11L/LEF1/ANGPT4/PIK3CG/PKHD1/PML/LIMS2/PRKD1/PROC/PSMB4/PAK6/PSMD7/PTGFR/PLEKHG5/MARK4/CCAR2/RASGRF2/SCT/NOD2/SFRP2/SGK1/BMP4/BMPR1B/BOK/SOX9/STK3/STK10/ACTC1/TERF1/TGM2/TNFAIP3/TRAF1/TRAF5/TWIST1/TNFRSF4/WNT10B/YWHAG/PAX8/CARD14/BCL2L14/CPEB4/COL18A1/CALR/CAST/KDM2B/SCIN/TP63/RUNX3/IRS2/ACTN1/CRADD/FADD/ALDH1A2/SPHK1/MAP3K6/AURKB/ADIPOQ/RAPGEF2	109
GO:0009967	positive regulation of signal transduction	105/901	1334/17046	2.10E-05	0.0012	0.00095	CDH3/TSPAN5/CDH13/CDKN1C/HCST/ESM1/CHI3L1/CCR1/MAP3K8/IL31RA/MIB2/MPP7/CTGF/CYLD/ADRB3/DRD4/EGFR/FGA/FGF10/RASA3/AKR1B1/PUM2/MAPK8IP2/MTOR/FGF22/NPTN/BMP10/GNAS/GPER1/GRB10/NRG1/RSPO2/IGF1/IGF2/CYR61/IL1RN/IL6/INHBA/ISL1/JUP/KDR/HES5/LCK/LGALS9/LHCGR/SMAD3/MAP3K1/MFNG/NOV/NRAS/NTF3/PARK2/PIK3CG/PLA2G2A/PML/TLR9/ZDHHC13/LIMS2/PAG1/PRKD1/MAPK3/MAP2K2/PSMB4/PSMD7/PLEKHG5/CCAR2/PXN/RASGRF2/S100A4/CCL11/CCL17/NOD2/SFRP2/BMP4/BMPR1B/SOX9/STK3/BST2/TLR5/TNFRSF1A/TRAF5/WNT10B/YWHAG/ZAP70/CXCR4/FZD5/CARD14/BCL2L14/SLA2/CDK10/TP63/RUNX3/IRS2/CRADD/FADD/TNFRSF11A/SPHK1/SKAP2/MAP3K6/RSAD2/CD8A/ADIPOQ/LY86/RAPGEF2/FGF19	105
GO:0060537	muscle tissue development	37/901	338/17046	2.24E-05	0.00127	0.001	SPEG/ZBTB18/CHRNA1/COL11A1/SMYD1/FOXC2/FLNB/VGLL2/TENM4/BMP10/SOX8/NRG1/HLX/ISL1/LMNA/SMAD3/MEF2D/MEOX2/MYL2/NFATC3/NRAS/LEF1/SIRT6/PITX2/PLAGL1/BIN3/STRA6/BMP4/SLC8A1/SOX9/ACTC1/TWIST1/WNT10B/CALR/CASQ1/TP63/ALDH1A2	37
GO:0045669	positive regulation of osteoblast differentiation	12/901	55/17046	2.36E-05	0.00133	0.00104	GNAS/IGF1/CYR61/IL6/NPPC/PRKD1/SFRP2/BMP4/BMPR1B/WNT10B/IFITM1/TP63	12
GO:0001944	vasculature development	53/901	557/17046	2.46E-05	0.00137	0.00108	CDH13/PDPN/LECT1/ESM1/CHI3L1/ADM/CTGF/EGR3/EPHA1/EPHB4/FGF10/VASH1/FOXC2/FOXO1/FLVCR1/ANXA2/NR4A1/HOXB3/IGF1/CYR61/IL6/ISL1/ITGA7/KDR/LOX/MEOX2/NFATC3/NOV/ATP5B/LEF1/ANGPT4/PIK3CG/PITX2/PML/ROBO4/PARVA/PRKD1/ACTA2/CCL11/STRA6/SFRP2/BMP4/TNFAIP3/TWIST1/CCR2/FZD5/TMEM204/ZC3H12A/COL18A1/RUNX1/ALDH1A2/SPHK1/RAPGEF2	53
GO:0010243	response to organonitrogen compound	76/901	893/17046	2.51E-05	0.00139	0.00109	TCIRG1/NPFFR2/ADCY3/TMED10/CHRNA1/CHRNA2/CHRNA5/AP3S1/ADM/CPS1/CTGF/CYP11A1/DNMT3A/ABAT/DRD4/AGXT/EGFR/EGR3/EIF4G1/FGA/FGF10/RASA3/FOXC2/FOXO1/AKR1B1/MTOR/GATM/FGF22/GNAS/GPER1/GRB10/NRG1/NR4A1/HPCA/HRH1/HTR3A/IGF2/IL1RN/IL6/AQP9/JUP/IPO5/LCK/ATP1A2/NRAS/PARK2/PIK3CG/PKM/PRKAG3/TLR9/SSH1/CHRNA9/PRKAR1B/LMBRD1/MAPK3/MAP2K2/PSMB4/PSMD7/PTPRE/PXN/RASGRF2/NOD2/SLC8A1/SOX9/VAMP2/ZEB1/TIMP3/TNFAIP3/WNT10B/CPEB4/MGARP/IRS2/ADIPOQ/RAPGEF2/FGF19/NR1H4	76
GO:0040012	regulation of locomotion	60/901	659/17046	2.71E-05	0.00148	0.00117	CDH13/TBR1/CCR1/EGFR/EPHA1/SPATA13/FGF10/VASH1/FOXC2/BMP10/GPER1/HAS1/IGF1/CYR61/IL1RN/IL6/IL16/KDR/LAMA3/LGALS9/LMNA/SMAD3/MCC/MAP3K1/NOV/NTF3/P2RY6/LEF1/ANGPT4/PITX2/ROBO4/ELP3/PRKD1/MAP2K2/FAM60A/CCL11/NOD2/SFRP2/SGK1/BMP4/SLC8A1/SOX9/STK10/BST2/PHLDA2/TWIST1/CCR2/PTP4A1/CXCR4/COL18A1/CALR/SH3BGRL3/SCRT1/PARD6B/IFITM1/IRS2/FADD/SPHK1/ADIPOQ/RAPGEF2	60
GO:0045321	leukocyte activation	59/901	647/17046	3.03E-05	0.00164	0.00129	CHGA/MAP3K8/IL31RA/CYLD/DDOST/RNF168/EGR3/UNC13D/FGF10/SBNO2/FLOT2/MTOR/GPER1/HLA-DOA/HLA-DPA1/HLA-E/HLX/ZC3H12D/IGF1/IGF2/IL6/INHBA/IRF1/ITGB2/LCK/LCP1/LGALS9/SMAD3/NFATC3/IL21R/LEF1/PIK3CG/IL20RB/APBB1IP/PAG1/PTPRE/NOD2/CXCR5/BMP4/BPI/SUPT6H/BST2/VAMP2/ZEB1/TNFAIP3/CCR2/TNFRSF4/ZAP70/CXCR4/FZD5/LST1/ZC3H12A/SLA2/IRS2/FADD/SKAP2/RSAD2/CD8A/CD79A	59

GO:0080090	regulation of primary metabolic process	334/901	5271/17046	3.06E-05	0.00164	0.00129	ABI1/ZNF783/CDH13/MBNL2/FARP1/CDKN1C/C1D/ZBTB18/DMRT2/CELF1/TBR1/NPFFR2/ADCY3/PNRC1/TMED10/HNRNPUL1/CHI3L1/ERLIN2/PSIP1/EGLN2/CARD16/ZBED9/CIDEA/CCR1/SLC51B/MAP3K8/ZFP42/ADM/IL31RA/ZNF358/LDLRAD3/CSTA/ZNF738/CTGF/SMYD1/SH3D19/CYLD/ADRB3/ESCO2/ZNF782/ZNF709/ZNF781/CITED4/RNF168/ZNF366/BHLHA15/NLRP6/DLG2/DNMT3A/DRD4/ECE1/EEF2/EGFR/EGR3/PATL2/EIF4G1/A2M/ELK4/EPHA1/ESR1/SP8/FGA/FGF10/FHIT/XRN2/RASA3/PPM1E/SBNO2/TRAK1/MSRB2/ACIN1/FOXL1/FOXC2/FOXO1/SPG20/GGA3/FLOT2/NEDD4L/LARP1/PUM2/RYBP/MAPK8IP2/VGLL2/MTOR/GABBR1/RNF144B/ZNF549/PNKD/FBXO2/GAPDHS/PABPC1/DNAJC2/FGF22/NPTN/DKK3/BMP10/ZNF638/GNAS/ZNF311/ZNF844/GPER1/DOK7/GRB10/ZBTB44/DNAJC15/GSTP1/GTF2B/BRF1/GUCY1A3/GZMA/ANXA2/SERPIND1/SOX8/NRG1/HLX/HMGA1/NR4A1/ACACB/HPCA/HOXB3/HOXC4/HOXC5/HOXC6/HOXD3/HRH1/ACADL/HSP90AB1/TFAP2E/ID3/COL28A1/BARHL2/IGF1/IGF2/CYR61/IL1RN/IL6/IL16/FOXK2/INHBA/IRF1/ISL1/ITGB2/ITIH3/ITIH4/JUP/USP50/HILS1/KDR/HES5/AFF3/LCK/LDLR/LGALS9/LHCGR/LMNA/LMO2/LTB/SMAD3/MC2R/ME1/ME2/MEF2D/MAP3K1/MEOX1/MEOX2/MFI2/MITF/LHX8/MOV10/NFATC3/NFYB/NHLH2/NPPC/NRAS/NTF3/OPRL1/PALM/PARK2/SPOCK3/LEF1/PRR16/ANGPT4/SIRT6/PGAM2/PI3/PIK3CG/PITX2/PKHD1/PLA2G2A/PLAGL1/PML/RIPPLY3/RIPK4/TLR9/CYTL1/POMC/SSH1/POU2AF1/BNC2/MED18/BANP/PPP1CB/PIWIL2/ELP3/PRMT6/DNAJC17/ZNF532/PPP2R2B/FANCI/CNOT11/VAC14/PRKAR1B/PRKD1/MYNN/MAPK3/MAP2K2/MRAP/PRMT8/MASP1/HTRA1/PSMB4/PAK6/ARNTL2/RGMA/PRDM11/PSMD7/TENM2/GATAD2B/METTL14/CCAR2/PXN/CREBZF/RASGRF2/TRIM27/SCT/CCL11/CCL17/NPAS3/NOD2/SFRP2/TRA2B/GZF1/SGK1/BMP4/ZNF649/BMPR1B/BRD9/ZSCAN18/BOK/SOX9/STAT2/STK3/STK10/SUPT6H/BST2/TAF4B/TBP/TCEA1/TCEB2/ZEB1/TEAD3/TERF1/TIMP3/TLE3/TNFAIP3/TNFRSF1A/TNXB/TRAF1/TRAF5/PHLDA2/TWIST1/CCR2/TNFRSF4/UCP1/VARS/WNT10B/YWHAG/ZNF7/ZNF124/ZNF177/PAX8/CXCR4/FZD5/RAB7A/CARD14/ZNF665/ZC3H14/ZNF606/ZC3H12A/CPEB4/ZNF436/CALR/SLIRP/CAST/SCRT1/HIST1H3A/SLA2/ZNF397/NR0B2/HOPX/SPINK7/KDM2B/LOXL3/CBX2/GAS7/CDK10/RUNX1/TP63/RUNX3/SERPINA6/IRS2/ACTN1/CRADD/FADD/TNFRSF11A/SPHK1/BUD31/CCNA1/LIMD1/PIAS2/ZFAND2A/MAP3K6/LDB2/CBFA2T2/AURKB/ADIPOQ/H2AFY/MICAL2/N4BP1/VGLL4/RAPGEF2/ZBTB39/FGF19/NR1H4	334
GO:0071495	cellular response to endogenous stimulus	95/901	1190/17046	3.12E-05	0.00166	0.00131	CDH13/CDKN1C/TCIRG1/NPFFR2/ADCY3/CIDEA/AP3S1/CPS1/CTGF/CYP11A1/DNMT3A/EGFR/EGR3/EIF4G1/ESR1/FGF10/RASA3/FOXC2/FOXO1/AKR1B1/SPG20/NEDD4L/ARHGEF18/MTOR/FGF22/NPTN/BMP10/GNAS/GPER1/GRB10/HAS1/NRG1/NR4A1/HRH1/IGF2/CYR61/IL1RN/IL6/INHBA/AQP9/ISL1/JUP/IPO5/HES5/LCK/LHCGR/LMO2/LTBP1/SMAD3/MAP3K1/MOV10/ATP1A2/NRAS/P2RY6/PARK2/LEF1/PIK3CG/PRKAG3/PML/TLR9/SSH1/PPP1CB/PPP1CC/PRKAR1B/LMBRD1/MAPK3/MAP2K2/HTRA1/PSMB4/RGMA/PSMD7/PTGFR/PTPRE/PXN/RASGRF2/NOD2/SFRP2/BMP4/SLC8A1/BMPR1B/SOX9/VAMP2/ZEB1/WNT10B/PAX8/CPEB4/NR0B2/TRIM63/MGARP/RUNX1/IRS2/ADIPOQ/RAPGEF2/FGF19/NR1H4	95
GO:0030278	regulation of ossification	23/901	170/17046	3.19E-05	0.00168	0.00132	CCR1/FAM101A/GNAS/ID3/IGF1/CYR61/IL6/SMAD3/NPPC/PRKD1/BGLAP/SFRP2/BMP4/SLC8A1/BMPR1B/SOX9/TWIST1/WNT10B/IFITM1/TP63/LIMD1/PIAS2/RSAD2	23
GO:0006950	response to stress	244/901	3676/17046	3.22E-05	0.00168	0.00132	ABI1/CDH3/TANK/KLRG1/RCAN2/KCNMB2/TCIRG1/MRVI1/SPON2/ADCY3/CHGA/CHI3L1/ERLIN2/PSIP1/EGLN2/TP53TG1/CCR1/MAP3K8/ADM/IL31RA/HUS1B/CPS1/PXDNL/PARP4/CTGF/CYLD/ADRB3/ESCO2/CYP11A1/TRPV3/DDB1/DDOST/RNF168/BHLHA15/COCH/NLRP6/DMBT1/DNMT3A/ABAT/DRD4/EGFR/EIF4G1/A2M/UNC13D/F11/FCGR2A/FGA/FGF10/RASA3/VASH1/SBNO2/MSRB2/ACIN1/FOXC2/FOXO1/AKR1B1/MLC1/NUP210/LARP1/PUM2/MAPK8IP2/MTOR/SLC37A4/ACOT11/FBXO2/GATM/GJA3/DNAJC2/FGF22/GJB2/BMP10/GNAS/GPER1/FFAR2/DNAJC15/SCG3/GSTP1/GUCY1A3/ANXA2/SERPIND1/NRG1/HK1/HLA-B/HLA-DPA1/HLA-E/HLA-F/HMGA1/NR4A1/HRH1/HSPA1L/HSP90AA1/HSP90AB1/ID3/CD300E/IGF1/IGF2/CYR61/IL1R1/IL1RN/IL6/AQP2/INHBA/IRF1/AQP9/ISL1/ITGB2/ITIH4/KCNJ8/KCNMB1/ACAT1/KRT15/LCK/LGALS9/LMNA/LOX/SMAD3/MAP3K1/MOV10/NUDT1/NDUFB4/NFATC3/NOV/NPPC/NRAS/OAS2/PARK2/ANGPT4/C11orf73/SIRT6/PIK3CG/PKM/PLA2G2A/PRKAG3/PML/IL20RB/TLR9/TREM1/APBB1IP/PRMT6/FANCI/WDR33/PRKAR1B/PRKD1/BIN3/MAPK3/MAP2K2/PRKRIR/PROC/MASP1/HTRA1/SLAMF8/PSMB4/PAK6/TRPC7/PSMD7/CCAR2/PTPRCAP/PXN/RASGRF2/RFC2/TRIM27/RPA3/DEFB134/CCL11/CCL17/NOD2/SFRP2/SGK1/VPS33A/BMP4/SLC8A1/BMPR1B/BOK/BPI/STAT2/STK3/STK10/BST2/TCEA1/TCEB2/TIMP3/TLR5/TNFAIP3/TNFRSF1A/TNXB/TRPC6/TRPM2/TWIST1/CCR2/TNFRSF4/UPP1/WNT10B/ZAP70/CA7/CACNA1E/CXCR4/FZD5/RAB7A/NLRX1/FAAP100/CPEB4/C6orf25/COL18A1/EEPD1/CALR/UNC93B1/CAPS/CAPZB/HIST1H3A/CASQ1/HOPX/IL1F10/MGARP/RAE1/IFITM1/KMO/RUNX1/TP63/IRS2/ACTN1/CRADD/FADD/TNFRSF11A/SPHK1/STBD1/HSPB3/LIMD1/CCRL2/MAP7/MAP3K6/SYT7/ESAM/SLC16A3/RSAD2/DAPL1/RCSD1/IL32/CD8A/ADIPOQ/LY86/NUP93/RAPGEF2/ULK2/FGF19	244
GO:0060429	epithelium development	85/901	1037/17046	3.29E-05	0.0017	0.00134	ABI1/CDH3/DMRT2/LECT1/FRMD6/ADM/CPS1/ZNF358/APCDD1/CSTA/CTGF/CYP11A1/DMBT1/EGFR/ESR1/FGF10/FOXC2/EXPH5/FLNB/FLOT2/LCE2B/GNAS/SOX8/NRG1/ID3/RSPO2/FMN1/IGF1/CYR61/LCE1C/LCE1D/LCE2D/IL6/INHBA/IVL/KDR/ACAT1/KRT15/INSC/HES5/LAMA3/SMAD3/MAP3K1/MEOX1/MEOX2/MITF/NFATC3/NTF3/LEF1/PITX2/PML/RIPK4/IFT122/MAP2K2/PXN/ACTA2/CCL11/STRA6/SFRP2/GZF1/BMP4/SLC8A1/SOX9/STK3/ZEB1/TGM2/TCHH/TNFRSF1A/TWIST1/WNT10B/PAX8/FZD5/COL18A1/BFSP2/KDM2B/RUNX1/TP63/RUNX3/ALDH1A2/LDB2/CBFA2T2/ADIPOQ/H2AFY/MICAL2/RAPGEF2	85
GO:1901698	response to nitrogen compound	80/901	961/17046	3.33E-05	0.00171	0.00134	TCIRG1/NPFFR2/ADCY3/TMED10/CHRNA1/CHRNA2/CHRNA5/AP3S1/ADM/CPS1/CTGF/CYP11A1/DNMT3A/ABAT/DRD4/AGXT/EGFR/EGR3/EIF4G1/FGA/FGF10/RASA3/FOXC2/FOXO1/AKR1B1/MTOR/GATM/FGF22/GNAS/GPER1/GRB10/NRG1/NR4A1/HPCA/HRH1/HTR3A/IGF2/IL1R1/IL1RN/IL6/AQP9/JUP/KCNJ8/IPO5/LCK/SMAD3/ATP1A2/NRAS/PARK2/PIK3CG/PKM/PRKAG3/TLR9/SSH1/CHRNA9/PRKAR1B/LMBRD1/MAPK3/MAP2K2/PSMB4/PSMD7/PTPRE/PXN/RASGRF2/NOD2/BMP4/SLC8A1/SOX9/VAMP2/ZEB1/TIMP3/TNFAIP3/WNT10B/CPEB4/MGARP/IRS2/ADIPOQ/RAPGEF2/FGF19/NR1H4	80
GO:0006793	phosphorus metabolic process	196/901	2851/17046	3.41E-05	0.00173	0.00136	AKT3/ABI1/GNE/FARP1/CDKN1C/SPEG/BCKDK/TCIRG1/HCST/NPFFR2/ADCY3/CHI3L1/ACOT7/ALPK2/CCR1/CNP/APOA1BP/MAP3K8/ADM/IL31RA/UBLCP1/CPS1/TRPM6/CTGF/PPM1L/MBOAT1/ADRB3/FITM1/ADAL/NLRP6/DLG2/DRD4/EGFR/ENO2/ADCK5/EPHA1/EPHA3/EPHB4/FGA/FGF10/FHIT/RASA3/PPM1E/FOXO1/MORC3/MAPK8IP2/TSSK2/MTOR/GABBR1/GAK/GAPDHS/RPS6KC1/FGF22/NPTN/AMPD2/PDE7B/AMPD3/BMP10/GNAS/PIGW/THEM5/GPER1/DOK7/GRB10/DNAJC15/GSTP1/GUCY1A3/NME7/ANXA2/NRG1/HK1/ACACB/HPCA/HRH1/HSP90AB1/DUPD1/NME9/IGF1/IGF2/CYR61/IL1RN/IL6/IL12RB2/INHBA/INPP5A/ISL1/ITGB2/KCNH2/KDR/HES5/LCK/LDLR/LGALS9/LHCGR/SMAD3/MC2R/ME1/ME2/MAP3K1/MGAT1/MOCS1/NUDT1/MYH4/NDUFB4/ATP1A2/NPPC/NRAS/NTF3/OAS2/OPRL1/ATP5B/PALM/PARK2/ANGPT4/PDE4C/PDE7A/SIRT6/PDE6B/PGAM2/PIGC/PIK3CG/PKHD1/PKM/PLA2G2A/PRKAG3/PML/RIPK4/TLR9/SSH1/PON1/LPCAT2/PPP1CB/PPP1CC/ELP3/PPP2R2B/SMPD3/VAC14/PRKAR1B/CSGALNACT1/PRKD1/MAPK3/MAP2K2/MRAP/SLAMF8/PSMB4/PAK6/PSMD7/MARK4/PTPRE/PXN/RASGRF2/TRIM27/SCT/CCL11/CCL17/NOD2/SFRP2/SGK1/CERK/BMP4/BMPR1B/SOX9/STK3/STK10/TNFAIP3/TNFRSF1A/TNXB/TWIST1/CCR2/TNFRSF4/UPP1/YWHAG/ZAP70/PTP4A1/CXCR4/FZD5/CARD14/TTBK1/CDK10/KMO/RUNX3/IRS2/TNFRSF11A/STK19/SYNJ2/SPHK1/LIMD1/MAP3K6/AURKB/ADIPOQ/H2AFY/ENTPD3/RAPGEF2/ULK2/LPGAT1/FGF19	196

GO:0045667	regulation of osteoblast differentiation	17/901	105/17046	3.43E-05	0.00173	0.00136	GNAS/ID3/IGF1/CYR61/IL6/SMAD3/NPPC/PRKD1/SFRP2/BMP4/BMPR1B/TWIST1/WNT10B/IFITM1/TP63/LIMD1/PIAS2	17
GO:1901700	response to oxygen-containing compound	112/901	1462/17046	3.53E-05	0.00177	0.00139	TCIRG1/SPON2/GJB6/NPFFR2/ADCY3/AP3S1/CNP/ADM/CPS1/CTGF/CYP11A1/DNMT3A/ABAT/DRD4/AGXT/EGFR/EGR3/EIF4G1/EPHA3/ESR1/FGA/FGF10/RASA3/SBNO2/FOXC2/FOXO1/AKR1B1/MLC1/MTOR/GATM/GJA3/FGF22/GJB2/GNAS/GPER1/FFAR2/GRB10/GSTP1/NRG1/NR4A1/HPCA/HSD17B2/HTR3A/HTR5A/IGF2/IL1R1/IL1RN/IL6/IL10RA/AQP2/IL12RB2/INHBA/AQP9/JUP/KCNJ8/IPO5/LCK/LGALS9/ME1/ATP1A2/NPPC/NRAS/OPRL1/P2RY6/PARK2/PIK3CG/PKM/PRKAG3/TLR9/SSH1/PON1/SYBU/PRKAR1B/LMBRD1/MAPK3/MAP2K2/PSMB4/PSMD7/PTGFR/PTPRE/PXN/RASGRF2/BGLAP/NOD2/BMP4/SLC8A1/SOX9/VAMP2/ZEB1/ACTC1/TIMP3/TLR5/TNFAIP3/TRPC6/TRPM2/WNT10B/CACNA1E/ZC3H12A/CPEB4/COL18A1/CALR/NR0B2/TRIM63/MGARP/IRS2/TNFRSF11A/ALDH1A2/ADIPOQ/LY86/RAPGEF2/FGF19/NR1H4	112
GO:0014070	response to organic cyclic compound	67/901	768/17046	3.60E-05	0.00179	0.0014	ADCY3/CHRNA1/CHRNA2/CHRNA5/CIDEA/ADM/CPS1/CTGF/CYP11A1/CITED4/ZNF366/DNMT3A/ABAT/DRD4/AGXT/EGFR/EGR3/ESR1/FGA/FGF10/MLC1/GJB2/GNAS/GPER1/GUCY1A3/NR4A1/HPCA/HRH1/HSP90AB1/HTR3A/HTR5A/IL1RN/IL6/INHBA/AQP9/ISL1/JUP/KCNJ8/ACAT1/LOX/SMAD3/ATP1A2/OPRL1/P2RY6/PARK2/LEF1/PIK3CG/SSH1/CHRNA9/MAPK3/PTGFR/BGLAP/NOD2/BMP4/SLC8A1/SLC9A3/TIMP3/WNT10B/CALR/NR0B2/TRIM63/MGARP/RAE1/ALDH1A2/ADIPOQ/RAPGEF2/NR1H4	67
GO:0042692	muscle cell differentiation	37/901	346/17046	3.73E-05	0.00184	0.00144	SPEG/ADM/SMYD1/BHLHA15/FGF10/FLOT2/SYNE1/MTOR/BMP10/TMOD4/SOX8/NRG1/ID3/IGF1/IGF2/ISL1/LMNA/MYL2/NFATC3/NOV/NTF3/LEF1/PITX2/BIN3/CCL17/BMP4/SLC8A1/SOX9/SUPT6H/ZEB1/ACTC1/WNT10B/TMEM204/CALR/CAST/CASQ1/HOPX	37
GO:0023056	positive regulation of signaling	112/901	1467/17046	4.07E-05	0.00199	0.00156	CDH3/TSPAN5/CDH13/CDKN1C/TRDN/HCST/ESM1/CHI3L1/CCR1/MAP3K8/IL31RA/MIB2/MPP7/CTGF/CYLD/ADRB3/ABAT/DRD4/ECE1/EGFR/FGA/FGF10/RASA3/AKR1B1/PUM2/MAPK8IP2/MTOR/FGF22/NPTN/BMP10/GNAS/GPER1/GRB10/NRG1/RSPO2/IGF1/IGF2/CYR61/IL1RN/IL6/INHBA/ISL1/JUP/KDR/HES5/LCK/LGALS9/LHCGR/SMAD3/MAP3K1/MFNG/NOV/NRAS/NTF3/PARK2/PIK3CG/PLA2G2A/PML/TLR9/ZDHHC13/LIMS2/PAG1/PRKD1/MAPK3/MAP2K2/PSMB4/PSMD7/PLEKHG5/CCAR2/PXN/RASGRF2/S100A4/SCT/CCL11/CCL17/NOD2/SFRP2/BMP4/BMPR1B/SOX9/STK3/BST2/VAMP2/TLR5/TNFRSF1A/TRAF5/WNT10B/YWHAG/ZAP70/CA7/CXCR4/FZD5/CARD14/BCL2L14/SLA2/NR0B2/CDK10/TP63/RUNX3/IRS2/CRADD/FADD/TNFRSF11A/SPHK1/SKAP2/MAP3K6/RSAD2/CD8A/ADIPOQ/LY86/RAPGEF2/FGF19	112
GO:0010647	positive regulation of cell communication	113/901	1485/17046	4.32E-05	0.00209	0.00164	CDH3/TSPAN5/CDH13/CDKN1C/TRDN/HCST/ESM1/CHI3L1/CCR1/MAP3K8/IL31RA/MIB2/MPP7/CTGF/CYLD/ADRB3/ABAT/DRD4/EGFR/FGA/FGF10/RASA3/AKR1B1/LARP1/PUM2/MAPK8IP2/MTOR/FGF22/NPTN/BMP10/GNAS/GPER1/GRB10/NRG1/HK1/RSPO2/IGF1/IGF2/CYR61/IL1RN/IL6/INHBA/ISL1/JUP/KDR/HES5/LCK/LGALS9/LHCGR/SMAD3/MAP3K1/MFNG/NOV/NRAS/NTF3/PARK2/PIK3CG/PLA2G2A/PML/TLR9/ZDHHC13/LIMS2/PAG1/PRKD1/MAPK3/MAP2K2/PSMB4/PSMD7/PLEKHG5/CCAR2/PXN/RASGRF2/S100A4/SCT/CCL11/CCL17/NOD2/SFRP2/BMP4/BMPR1B/SOX9/STK3/BST2/VAMP2/TLR5/TNFRSF1A/TRAF5/WNT10B/YWHAG/ZAP70/CA7/CXCR4/FZD5/CARD14/BCL2L14/SLA2/NR0B2/CDK10/TP63/RUNX3/IRS2/CRADD/FADD/TNFRSF11A/SPHK1/SKAP2/MAP3K6/RSAD2/CD8A/ADIPOQ/LY86/RAPGEF2/FGF19	113
GO:0051241	negative regulation of multicellular organismal process	76/901	908/17046	4.40E-05	0.00211	0.00166	CDH3/MRVI1/LECT1/CIDEA/CCR1/SEZ6/ADM/FAM101A/CYLD/ADRB3/TRPV3/F11/FGA/VASH1/FOXC2/SPG20/BMP10/GNAS/GSTP1/GUCY1A3/ANXA2/SOX8/HLX/ID3/RSPO2/IGF1/IL6/INHBA/IRF1/ISL1/HES5/ARHGDIA/LGALS9/SMAD3/MCC/ATP1A2/NOV/NPPC/NRAS/LEF1/CEND1/ANGPT4/PIK3CG/PML/IL20RB/TLR9/POMC/LIMS2/IFT122/PROC/TRIM27/SCT/NOD2/SFRP2/BMP4/SLIT1/SOX9/BPI/STK3/BST2/TNFAIP3/TWIST1/CCR2/TNFRSF4/PAX8/NLRX1/ZC3H12A/RAB11FIP1/CALR/RUNX1/TP63/LIMD1/CBFA2T2/ADIPOQ/RAPGEF2/ULK2	76
GO:0009889	regulation of biosynthetic process	252/901	3834/17046	4.52E-05	0.00211	0.00166	CDH3/ZNF783/CDH13/CDKN1C/C1D/ZBTB18/DMRT2/CELF1/TBR1/NPFFR2/PNRC1/HNRNPUL1/ERLIN2/PSIP1/EGLN2/ZBED9/CIDEA/SLC51B/ZFP42/ADM/IL31RA/ZNF358/ZNF738/CTGF/SMYD1/CYLD/ADRB3/ESCO2/ZNF782/ZNF709/ZNF781/CITED4/RNF168/ZNF366/BHLHA15/DNMT3A/DRD4/EEF2/EGFR/EGR3/PATL2/EIF4G1/ELK4/ESR1/SP8/FGF10/FHIT/XRN2/SBNO2/TRAK1/MSRB2/FOXL1/FOXC2/FOXO1/SPG20/NEDD4L/LARP1/PUM2/RYBP/VGLL2/MTOR/GABBR1/ZNF549/PABPC1/DNAJC2/DKK3/BMP10/ZNF638/GNAS/ZNF311/ZNF844/GPER1/GRB10/ZBTB44/GSTP1/GTF2B/BRF1/GUCY1A3/SOX8/NRG1/HLX/HMGA1/NR4A1/HPCA/HOXB3/HOXC4/HOXC5/HOXC6/HOXD3/HRH1/ACADL/HSP90AA1/HSP90AB1/TFAP2E/ID3/BARHL2/IGF1/IGF2/CYR61/IL6/IL16/FOXK2/INHBA/IRF1/ISL1/JUP/USP50/HILS1/KDR/HES5/AFF3/LDLR/LGALS9/LHCGR/LMNA/LMO2/LTB/SMAD3/MC2R/MEF2D/MEOX1/MEOX2/MITF/LHX8/MOV10/NFATC3/NFYB/NHLH2/NPPC/NTF3/OPRL1/PALM/PARK2/LEF1/PRR16/SIRT6/PITX2/PKHD1/PLAGL1/PML/RIPPLY3/RIPK4/TLR9/CYTL1/POMC/POU2AF1/BNC2/MED18/BANP/PPP1CB/PIWIL2/ELP3/PRMT6/DNAJC17/ZNF532/CNOT11/PRKD1/MYNN/MAPK3/MRAP/PRMT8/HTRA1/PAK6/ARNTL2/RGMA/PRDM11/TENM2/GATAD2B/METTL14/CCAR2/CREBZF/TRIM27/SCT/NPAS3/NOD2/SFRP2/GZF1/SGK1/BMP4/ZNF649/BMPR1B/BRD9/ZSCAN18/SOX9/STAT2/STK3/SUPT6H/TAF4B/TBP/TCEA1/TCEB2/ZEB1/TEAD3/TERF1/TLE3/TLR5/TNFAIP3/TNFRSF1A/TRAF1/TRAF5/TWIST1/CCR2/TNFRSF4/UCP1/VARS/WNT10B/ZNF7/ZNF124/ZNF177/PAX8/FZD5/CARD14/ZNF665/ZNF606/ZC3H12A/CPEB4/ZNF436/CALR/SLIRP/SCRT1/HIST1H3A/SLA2/ZNF397/NR0B2/HOPX/KDM2B/LOXL3/CBX2/GAS7/RUNX1/TP63/RUNX3/IRS2/ACTN1/FADD/TNFRSF11A/SPHK1/BUD31/CCNA1/LIMD1/PIAS2/LDB2/CBFA2T2/AURKB/ADIPOQ/H2AFY/MICAL2/VGLL4/RAPGEF2/ZBTB39/FGF19/NR1H4	252
GO:0009893	positive regulation of metabolic process	220/901	3277/17046	4.54E-05	0.00211	0.00166	ABI1/CDH3/CDH13/FARP1/CDKN1C/PITRM1/DMRT2/TBR1/ADCY3/CHI3L1/ERLIN2/PSIP1/EGLN2/CCR1/SLC51B/MAP3K8/ADM/IL31RA/CTGF/SH3D19/ADRB3/CITED4/RNF168/BHLHA15/DRD4/ECE1/EEF2/EGFR/EPHA1/ESR1/SPATA13/FGA/FGF10/RASA3/SBNO2/FOXC2/TBC1D9B/FOXO1/GGA3/TBC1D1/PSD3/LARP1/ARHGEF18/MAPK8IP2/VGLL2/MTOR/RASGEF1C/RNF144B/ALS2CL/RGS22/GAPDHS/PABPC1/DNAJC2/FGF22/NPTN/CYTH4/BMP10/GNAS/GPER1/DOK7/GRB10/DNAJC15/GSTP1/BRF1/GUCY1A3/ANXA2/SOX8/NRG1/HK1/HMGA1/NR4A1/ACACB/HPCA/HOXD3/AGFG2/HRH1/HSP90AA1/HSP90AB1/TFAP2E/BARHL2/IGF1/IGF2/CYR61/IL1RN/IL6/FOXK2/INHBA/IRF1/ISL1/JUP/KDR/HES5/LCK/LDLR/ARHGDIA/LGALS9/LHCGR/LLGL1/LMNA/LMO2/LTB/SMAD3/MC2R/MEF2D/MAP3K1/MEOX1/MEOX2/MFI2/MITF/PLEKHG7/NFATC3/NFYB/NHLH2/NPPC/NRAS/NTF3/OPRL1/ARHGEF3/PARK2/LEF1/PRR16/ANGPT4/PIK3CG/PITX2/PLA2G2A/PLAGL1/PML/PNLIP/RIPK4/TLR9/CYTL1/POMC/RIN2/BANP/PIWIL2/ARHGEF10L/FANCI/PRKAR1B/PRKD1/MAPK3/MAP2K2/MRAP/PSMB4/PAK6/ARNTL2/RGMA/PSMD7/PTGFR/PLEKHG5/PXN/ACTA2/RASGRF2/TRIM27/RGS12/SCT/CCL11/CCL17/NPAS3/NOD2/SFRP2/ARHGAP9/TRA2B/BMP4/ZNF649/BMPR1B/BOK/SOX9/STK3/STK10/SUPT6H/TBP/TCEA1/TCEB2/ZEB1/ACTC1/TEAD3/TLR5/TNFAIP3/TNFRSF1A/TRAF1/TRAF5/TWIST1/CCR2/WNT10B/PAX8/CXCR4/FZD5/RAB7A/CARD14/ZC3H12A/CALR/SH3BGRL3/NR0B2/CDK10/RUNX1/TP63/RUNX3/IRS2/CRADD/FADD/TNFRSF11A/ALDH1A2/SPHK1/STARD13/PIAS2/ZFAND2A/MAP3K6/LDB2/CBFA2T2/ADIPOQ/ARHGAP29/H2AFY/ARHGEF10/MICAL2/RAPGEF2/USP6NL/RABGAP1L/IQSEC1/FGF19/NR1H4	220

GO:0009725	response to hormone	81/901	985/17046	4.54E-05	0.00211	0.00166	TCIRG1/NPFFR2/ADCY3/AP3S1/ADM/CPS1/CTGF/CYP11A1/CITED4/ZNF366/DNMT3A/DRD4/AGXT/EGFR/EGR3/EIF4G1/ESR1/FGA/FGF10/RASA3/FOXC2/FOXO1/AKR1B1/MTOR/STEAP2/GATM/FGF22/GJB2/GNAS/GPER1/GRB10/NRG1/NR4A1/HTR5A/IGF2/IL1RN/IL6/INHBA/ISL1/ACAT1/LCK/LHCGR/LMO2/LOX/ME1/ATP1A2/NRAS/OPRL1/P2RY6/LEF1/PITX2/PKM/PRKAG3/TLR9/PRKAR1B/LMBRD1/MAPK3/MAP2K2/PSMB4/PSMD7/PTGFR/PTPRE/PXN/RASGRF2/BGLAP/BMP4/SLC9A3/VAMP2/TIMP3/WNT10B/PAX8/CALR/NR0B2/TRIM63/MGARP/IRS2/ALDH1A2/ADIPOQ/RAPGEF2/FGF19/NR1H4	81
GO:0006955	immune response	113/901	1487/17046	4.57E-05	0.00211	0.00166	ABI1/TANK/KLRG1/SPON2/HCST/ADCY3/CHGA/CCR1/MAP3K8/ADM/CYLD/DDOST/RNF168/COCH/NLRP6/DMBT1/EGFR/A2M/UNC13D/FCGR2A/FGA/FGF10/RASA3/SBNO2/FOXO1/MTOR/FGF22/GPER1/FFAR2/GZMA/NRG1/HLA-B/HLA-DOA/HLA-DPA1/HLA-E/HLA-F/HLX/NR4A1/HSP90AA1/HSP90AB1/CD300E/IL1R1/IL1RN/IL6/IL16/IRF1/AQP9/ITGB2/ITGB7/LCK/LCP1/LGALS9/LTB/SMAD3/MAP3K1/MOV10/NFATC3/NRAS/OAS2/LEF1/PIK3CG/PML/IL20RB/TLR9/TREM1/POU2AF1/APBB1IP/PRKAR1B/PAG1/PRKD1/MAPK3/MAP2K2/MASP1/PSMB4/PSMD7/RASGRF2/TRIM27/DEFB134/CCL11/CCL17/NOD2/TINAGL1/CXCR5/BPI/STAT2/SUPT6H/BST2/VAMP2/ZEB1/TLR5/TNFAIP3/CCR2/TNFRSF4/ZAP70/FZD5/LST1/NLRX1/UNC93B1/SLA2/IL1F10/IFITM1/IRS2/FADD/TNFRSF11A/ENDOU/SKAP2/RSAD2/IL32/CD8A/LY86/RAPGEF2/CD79A/FGF19	113
GO:0050673	epithelial cell proliferation	35/901	323/17046	4.58E-05	0.00211	0.00166	CDH3/CDH13/CDKN1C/LECT1/EGFR/EGR3/ESR1/FGF10/VASH1/MTOR/NR4A1/IGF1/IL6/KDR/SMAD3/MCC/NOV/PLA2G2A/LIMS2/IFT122/PRKD1/MAP2K2/HTRA1/SLURP1/CCL11/NOD2/SFRP2/BMP4/SOX9/TNFAIP3/TWIST1/WNT10B/TP63/RUNX3/ALDH1A2	35
GO:0007517	muscle organ development	36/901	337/17046	4.84E-05	0.00222	0.00174	SPEG/ZBTB18/CHRNA1/COL11A1/SMYD1/EGR3/FOXC2/FLNB/VGLL2/BMP10/SOX8/NRG1/HLX/ID3/IGF1/IL6/ISL1/ITGA7/LMNA/SMAD3/MEF2D/MEOX2/MYL2/NFATC3/NRAS/LEF1/SIRT6/PITX2/PLAGL1/BIN3/STRA6/BMP4/ACTC1/TWIST1/WNT10B/CASQ1	36
GO:1901362	organic cyclic compound biosynthetic process	263/901	4032/17046	4.92E-05	0.00224	0.00176	CDH3/ZNF783/CDH13/CDKN1C/TCIRG1/C1D/ZBTB18/MTHFS/DMRT2/TBR1/NPFFR2/ADCY3/PNRC1/SLC27A2/HNRNPUL1/ERLIN2/PSIP1/EGLN2/ACOT7/ZBED9/CIDEA/ZFP42/ADM/IL31RA/CPS1/ZNF358/ZNF738/CTGF/SMYD1/CYLD/ADRB3/CYP11A1/ZNF782/ADAL/ZNF709/ZNF781/CITED4/DDB1/RNF168/ZNF366/BHLHA15/DNMT3A/DRD4/EGFR/EGR3/ELK4/ESR1/ALAS1/SP8/FGF10/FHIT/XRN2/SBNO2/TRAK1/MSRB2/FOXL1/FOXC2/FOXO1/AKR1B1/NUP210/NEDD4L/RYBP/VGLL2/MTOR/GABBR1/ZNF549/DNAJC2/AMPD2/DKK3/GLS2/AMPD3/BMP10/ZNF638/GNAS/ZNF311/ZNF844/GPER1/ZBTB44/GTF2B/BRF1/GUCY1A3/NME7/SOX8/NRG1/HLX/HMGA1/NR4A1/ACACB/HPCA/HOXB3/HOXC4/HOXC5/HOXC6/HOXD3/HSD11B1/HSD17B2/TFAP2E/ID3/BARHL2/NME9/IGF1/IGF2/CYR61/IL6/IL16/FOXK2/INHBA/IRF1/ISL1/JUP/USP50/HILS1/HES5/AFF3/LGALS9/LHCGR/LMO2/SMAD3/MC2R/ME1/MEF2D/MEOX1/MEOX2/MITF/LHX8/MOCS1/MOV10/DRG1/NFATC3/NFYB/NHLH2/NPPC/NTF3/OAS2/OPRL1/ATP5B/PALM/PARK2/LEF1/SIRT6/PITX2/PKHD1/PKM/PLAGL1/PRKAG3/PML/RIPPLY3/RIPK4/TLR9/CYTL1/POMC/POU2AF1/BNC2/MED18/BANP/ELP3/PRMT6/DNAJC17/ZNF532/CNOT11/PRKD1/MYNN/MAPK3/MRAP/PRMT8/PAK6/ARNTL2/RGMA/PRDM11/TENM2/GATAD2B/CCAR2/CREBZF/RFC2/TRIM27/RGS12/RPA3/RPL8/RPL29/SCT/NPAS3/NOD2/SFRP2/GZF1/SGK1/BMP4/ZNF649/BMPR1B/BRD9/ZSCAN18/SOX9/STAT2/STK3/SUPT6H/TAF4B/TBP/TCEA1/TCEB2/ZEB1/TEAD3/TERF1/TLE3/TNFAIP3/TNFRSF1A/TRAF1/TRAF5/TWIST1/CCR2/TNFRSF4/UCP1/UPP1/WNT10B/YWHAG/ZNF7/ZNF124/ZNF177/PAX8/FZD5/CARD14/ZNF665/ZNF606/ZC3H12A/ZNF436/CALR/QTRT1/SLIRP/SCRT1/HIST1H3A/SLA2/ZNF397/NR0B2/HOPX/KDM2B/LOXL3/CBX2/RAE1/GAS7/KMO/RUNX1/TP63/RUNX3/ACTN1/FADD/TNFRSF11A/SPHK1/BUD31/CCNA1/LIMD1/CH25H/PIAS2/LDB2/CBFA2T2/AURKB/SDR42E1/ADIPOQ/H2AFY/MICAL2/VGLL4/NUP93/RAPGEF2/ZBTB39/FGF19/NR1H4	263
GO:0044057	regulation of system process	42/901	418/17046	4.98E-05	0.00225	0.00177	KCNMB2/MRVI1/TRDN/CELF2/ADM/CPS1/CTGF/ECE1/FGA/FGF10/NEDD4L/TENM4/BMP10/GPER1/GUCY1A3/KCNIP2/NRG1/ANXA6/HRH1/IGF1/IL6/INHBA/JUP/KCNH2/LCK/MYL2/ATP1A2/NOV/NPPC/OPRL1/PIK3CG/POMC/SCT/SGK1/SLC8A1/CACNA1E/RAB11FIP1/CASQ1/HOPX/TRIM63/SPHK1/ADIPOQ	42
GO:0055086	nucleobase-containing small molecule metabolic process	60/901	675/17046	5.43E-05	0.00243	0.00191	GNE/TCIRG1/NPFFR2/ADCY3/ACOT7/CNP/APOA1BP/ADM/CPS1/ADRB3/ADAL/DLG2/DNMT3A/DRD4/ENO2/FHIT/MTOR/GABBR1/GAPDHS/AMPD2/PDE7B/AMPD3/GNAS/GPER1/DNAJC15/GUCY1A3/NME7/HK1/ACACB/HPCA/NME9/IGF1/LHCGR/MC2R/ME1/ME2/MGAT1/NUDT1/MYH4/NDUFB4/ATP1A2/NPPC/OAS2/OPRL1/ATP5B/PALM/PDE4C/PDE7A/SIRT6/PDE6B/PGAM2/PKM/CSGALNACT1/MRAP/SCT/CCR2/UPP1/QTRT1/KMO/ENTPD3	60
GO:0031326	regulation of cellular biosynthetic process	249/901	3792/17046	5.50E-05	0.00245	0.00192	CDH3/ZNF783/CDH13/CDKN1C/C1D/ZBTB18/DMRT2/CELF1/TBR1/NPFFR2/PNRC1/HNRNPUL1/ERLIN2/PSIP1/EGLN2/ZBED9/CIDEA/SLC51B/ZFP42/ADM/IL31RA/ZNF358/ZNF738/SMYD1/CYLD/ADRB3/ESCO2/ZNF782/ZNF709/ZNF781/CITED4/RNF168/ZNF366/BHLHA15/DNMT3A/DRD4/EEF2/EGFR/EGR3/PATL2/EIF4G1/ELK4/ESR1/SP8/FGF10/FHIT/XRN2/SBNO2/TRAK1/MSRB2/FOXL1/FOXC2/FOXO1/SPG20/NEDD4L/LARP1/PUM2/RYBP/VGLL2/MTOR/GABBR1/ZNF549/PABPC1/DNAJC2/DKK3/BMP10/ZNF638/GNAS/ZNF311/ZNF844/GPER1/GRB10/ZBTB44/GTF2B/BRF1/GUCY1A3/SOX8/NRG1/HLX/HMGA1/NR4A1/HPCA/HOXB3/HOXC4/HOXC5/HOXC6/HOXD3/HRH1/ACADL/HSP90AA1/HSP90AB1/TFAP2E/ID3/BARHL2/IGF1/IGF2/CYR61/IL6/IL16/FOXK2/INHBA/IRF1/ISL1/JUP/USP50/HILS1/HES5/AFF3/LDLR/LGALS9/LHCGR/LMNA/LMO2/LTB/SMAD3/MC2R/MEF2D/MEOX1/MEOX2/MITF/LHX8/MOV10/NFATC3/NFYB/NHLH2/NPPC/NTF3/OPRL1/PALM/PARK2/LEF1/PRR16/SIRT6/PITX2/PKHD1/PLAGL1/PML/RIPPLY3/RIPK4/TLR9/CYTL1/POMC/POU2AF1/BNC2/MED18/BANP/PPP1CB/PIWIL2/ELP3/PRMT6/DNAJC17/ZNF532/CNOT11/PRKD1/MYNN/MAPK3/MRAP/PRMT8/HTRA1/PAK6/ARNTL2/RGMA/PRDM11/TENM2/GATAD2B/METTL14/CCAR2/CREBZF/TRIM27/SCT/NPAS3/NOD2/SFRP2/GZF1/SGK1/BMP4/ZNF649/BMPR1B/BRD9/ZSCAN18/SOX9/STAT2/STK3/SUPT6H/TAF4B/TBP/TCEA1/TCEB2/ZEB1/TEAD3/TERF1/TLE3/TLR5/TNFAIP3/TNFRSF1A/TRAF1/TRAF5/TWIST1/CCR2/TNFRSF4/UCP1/VARS/WNT10B/ZNF7/ZNF124/ZNF177/PAX8/FZD5/CARD14/ZNF665/ZNF606/ZC3H12A/CPEB4/ZNF436/CALR/SLIRP/SCRT1/HIST1H3A/SLA2/ZNF397/NR0B2/HOPX/KDM2B/LOXL3/CBX2/GAS7/RUNX1/TP63/RUNX3/IRS2/ACTN1/FADD/TNFRSF11A/SPHK1/BUD31/CCNA1/LIMD1/PIAS2/LDB2/CBFA2T2/AURKB/ADIPOQ/H2AFY/MICAL2/VGLL4/RAPGEF2/ZBTB39/FGF19/NR1H4	249
GO:0071417	cellular response to organonitrogen compound	56/901	619/17046	5.97E-05	0.00264	0.00207	TCIRG1/ADCY3/AP3S1/CPS1/CYP11A1/DNMT3A/EGFR/EGR3/EIF4G1/FGF10/RASA3/FOXC2/FOXO1/AKR1B1/MTOR/FGF22/GNAS/GPER1/GRB10/NRG1/NR4A1/HRH1/IGF2/IL1RN/AQP9/JUP/IPO5/LCK/NRAS/PARK2/PIK3CG/PRKAG3/TLR9/SSH1/PRKAR1B/LMBRD1/MAPK3/MAP2K2/PSMB4/PSMD7/PTPRE/PXN/RASGRF2/NOD2/SLC8A1/SOX9/VAMP2/ZEB1/WNT10B/CPEB4/MGARP/IRS2/ADIPOQ/RAPGEF2/FGF19/NR1H4	56

GO:0045778	positive regulation of ossification	14/901	79/17046	6.02E-05	0.00264	0.00207	GNAS/IGF1/CYR61/IL6/SMAD3/NPPC/PRKD1/SFRP2/BMP4/SLC8A1/BMPR1B/WNT10B/IFITM1/TP63	14
GO:0045597	positive regulation of cell differentiation	66/901	768/17046	6.48E-05	0.00282	0.00222	CCR1/SEZ6/ADM/CTGF/SMYD1/DMBT1/EGR3/EIF4G1/UNC13D/EPHA3/FGA/ACIN1/NEDD4L/MTOR/TENM4/NPTN/BMP10/GNAS/GPER1/SOX8/NRG1/HLX/HOXD3/IGF1/CYR61/IL6/INHBA/KDR/LCK/ARHGDIA/LGALS9/SMAD3/NEU1/NPPC/PALM/PARK2/LEF1/ATP8A2/PLA2G2A/PML/PRKD1/MAP2K2/SFRP2/BMP4/BMPR1B/SOX9/STK3/ZEB1/TEAD3/TWIST1/WNT10B/ZAP70/PAX8/CXCR4/ZC3H12A/CALR/HOPX/IFITM1/SCIN/RUNX1/TP63/FADD/CBFA2T2/ADIPOQ/H2AFY/RAPGEF2	66
GO:0044281	small molecule metabolic process	166/901	2375/17046	6.81E-05	0.00294	0.00231	GNE/BCKDK/TCIRG1/MTHFS/PDPN/NPFFR2/ADCY3/SLC27A2/HIBADH/ERLIN2/EGLN2/B4GALT7/ACOT7/CNP/APOA1BP/NEU4/GALM/ADM/EGFLAM/CPS1/NDUFAF6/CRABP1/B3GLCT/CTGF/PPM1L/MBOAT1/ADRB3/CYP11A1/ADAL/DIO3/DLG2/DNMT3A/ABAT/DRD4/AGXT/ENO2/ALAS1/FAH/FHIT/FOXO1/AKR1B1/NUP210/MTOR/FUCA1/SLC37A4/GABBR1/PNKD/ACOT11/GATM/GAPDHS/AMPD2/PDE7B/DKK3/GLS2/AMPD3/DHDH/GNAS/THEM5/GPER1/GRB10/DNAJC15/GSTP1/GUCY1A3/NME7/PADI1/HAS1/HK1/ACACB/HPCA/HRH1/HSD11B1/ACADL/HSP90AA1/NME9/IGF1/IGF2/IL1RN/IL6/INPP5A/ACAT1/LDLR/LHCGR/MC2R/ME1/ME2/MGAT1/MOCS1/NUDT1/MYH4/NUBP1/NDUFB4/NEU1/ATP1A2/NFYB/NPPC/OAS2/OPRL1/ATP5B/PALM/PARK2/SPOCK3/CHST15/PDE4C/PCYOX1/PDE7A/SIRT6/PDE6B/PGAM2/PIK3CG/PKM/PLA2G2A/PRKAG3/SLCO1C1/PNLIP/CYTL1/POMC/PON1/CYP2W1/LPCAT2/PPP1CB/PPP1CC/SMPD3/VAC14/PRKAR1B/LMBRD1/CSGALNACT1/PRKD1/APOBR/MAPK3/MRAP/PSMB4/PSMD7/RDH14/SCT/STRA6/CERK/SLC4A1/VAMP2/TNFRSF1A/TNXB/PHLDA2/TWIST1/CCR2/UCP1/UPP1/VARS/CA7/CACNA1E/PAX8/CERS4/QTRT1/NR0B2/RAE1/KMO/IRS2/ALDH1A2/SYNJ2/SPHK1/CH25H/SLC16A3/ADIPOQ/ENTPD3/NUP93/LPGAT1/FGF19/NR1H4	166
GO:0001568	blood vessel development	50/901	536/17046	6.89E-05	0.00295	0.00232	CDH13/LECT1/ESM1/CHI3L1/ADM/CTGF/EGR3/EPHA1/EPHB4/FGF10/VASH1/FOXC2/FOXO1/FLVCR1/ANXA2/NR4A1/HOXB3/CYR61/IL6/ISL1/ITGA7/KDR/LOX/MEOX2/NFATC3/NOV/ATP5B/LEF1/ANGPT4/PIK3CG/PITX2/PML/ROBO4/PARVA/PRKD1/ACTA2/CCL11/STRA6/SFRP2/BMP4/TNFAIP3/TWIST1/CCR2/FZD5/ZC3H12A/COL18A1/RUNX1/ALDH1A2/SPHK1/RAPGEF2	50
GO:0022603	regulation of anatomical structure morphogenesis	70/901	830/17046	7.01E-05	0.00298	0.00234	PDPN/DMRT2/TBR1/LECT1/CHI3L1/ADM/SH3D19/CYLD/COCH/UNC13D/EPHA1/EPHA3/ESR1/FGA/FGF10/VASH1/FOXC2/SPG20/EPB41L3/NEDD4L/ARHGEF18/TENM4/GAS2/BMP10/SOX8/RSPO2/BARHL2/IL1RN/IL6/ISL1/ITGA7/ITGB2/KDR/ARHGDIA/SMAD3/MFI2/PALM/PARK2/LEF1/ANGPT4/PML/SSH1/FBLIM1/PALMD/LIMS2/PARVA/PRKD1/MAP2K2/CDC42SE1/ERMN/PXN/CCL11/SFRP2/BMP4/SOX9/TNFAIP3/TWIST1/CCR2/PAX8/LST1/CALR/CAPZB/TTBK1/GAS7/RUNX1/SPHK1/LIMD1/ADIPOQ/RAPGEF2/ULK2	70
GO:0019220	regulation of phosphate metabolic process	117/901	1568/17046	7.23E-05	0.00305	0.0024	ABI1/FARP1/CDKN1C/NPFFR2/ADCY3/CHI3L1/CCR1/MAP3K8/ADM/IL31RA/CTGF/ADRB3/NLRP6/DLG2/DRD4/EGFR/EPHA1/FGA/FGF10/RASA3/PPM1E/FOXO1/MAPK8IP2/MTOR/GABBR1/GAPDHS/FGF22/NPTN/BMP10/GNAS/GPER1/DOK7/GRB10/DNAJC15/GSTP1/GUCY1A3/ANXA2/NRG1/HPCA/HRH1/HSP90AB1/IGF1/IGF2/CYR61/IL1RN/IL6/INHBA/ISL1/ITGB2/KDR/HES5/LCK/LDLR/LGALS9/LHCGR/SMAD3/MC2R/ME1/ME2/MAP3K1/NPPC/NRAS/NTF3/OPRL1/PALM/PARK2/ANGPT4/SIRT6/PGAM2/PIK3CG/PKHD1/PLA2G2A/PML/TLR9/ELP3/PPP2R2B/VAC14/PRKAR1B/PRKD1/MAPK3/MAP2K2/MRAP/SLAMF8/PSMB4/PAK6/PSMD7/PXN/RASGRF2/TRIM27/SCT/CCL11/CCL17/NOD2/SFRP2/BMP4/SOX9/STK3/STK10/TNFAIP3/TNFRSF1A/TNXB/TWIST1/CCR2/TNFRSF4/YWHAG/CXCR4/FZD5/CARD14/CDK10/IRS2/TNFRSF11A/SPHK1/MAP3K6/ADIPOQ/H2AFY/RAPGEF2/FGF19	117
GO:0006753	nucleoside phosphate metabolic process	56/901	624/17046	7.43E-05	0.00311	0.00245	GNE/TCIRG1/NPFFR2/ADCY3/ACOT7/CNP/APOA1BP/ADM/ADRB3/ADAL/DLG2/DRD4/ENO2/FHIT/GABBR1/GAPDHS/AMPD2/PDE7B/AMPD3/GNAS/GPER1/DNAJC15/GUCY1A3/NME7/HK1/ACACB/HPCA/NME9/IGF1/LHCGR/MC2R/ME1/ME2/MGAT1/NUDT1/MYH4/NDUFB4/ATP1A2/NPPC/OAS2/OPRL1/ATP5B/PALM/PDE4C/PDE7A/SIRT6/PDE6B/PGAM2/PKM/CSGALNACT1/MRAP/SCT/CCR2/UPP1/KMO/ENTPD3	56
GO:0070371	ERK1 and ERK2 cascade	27/901	228/17046	7.47E-05	0.00311	0.00245	CHI3L1/CCR1/CTGF/NLRP6/EGFR/FGA/FGF10/GPER1/GSTP1/IGF1/CYR61/IL6/KDR/LGALS9/PKHD1/PLA2G2A/MAPK3/MAP2K2/CCL11/CCL17/NOD2/BMP4/SOX9/TNFRSF11A/ADIPOQ/RAPGEF2/FGF19	27
GO:0071407	cellular response to organic cyclic compound	37/901	359/17046	8.15E-05	0.00337	0.00265	ADCY3/CPS1/CYP11A1/EGFR/EGR3/ESR1/FGA/MLC1/GNAS/GPER1/NR4A1/HRH1/HSP90AB1/IL1RN/IL6/INHBA/AQP9/ISL1/JUP/SMAD3/ATP1A2/P2RY6/PARK2/LEF1/PIK3CG/SSH1/BGLAP/BMP4/SLC8A1/WNT10B/NR0B2/TRIM63/MGARP/RAE1/ADIPOQ/RAPGEF2/NR1H4	37
GO:0019221	cytokine-mediated signaling pathway	48/901	512/17046	8.49E-05	0.00349	0.00274	CCR1/IL31RA/EIF4G1/FLNB/NUP210/GSTP1/HLA-B/HLA-DPA1/HLA-E/HLA-F/HSP90AB1/IL1R1/IL1RN/IL6/IL10RA/IL11RA/IL12RB2/IL15RA/IRF1/LTB/OAS2/IL21R/PARK2/PML/IL20RB/MAPK3/PSMB4/PSMD7/CCL11/CCL17/CXCR5/STAT2/BST2/TNFRSF1A/CCR2/TNFRSF4/CXCR4/CARD14/IL1F10/RAE1/IFITM1/FADD/TNFRSF11A/SPHK1/CCRL2/RSAD2/ADIPOQ/NUP93	48
GO:0033993	response to lipid	65/901	761/17046	8.81E-05	0.00359	0.00283	SPON2/GJB6/ADCY3/CNP/ADM/CPS1/CTGF/CYP11A1/CITED4/ZNF366/DNMT3A/DRD4/AGXT/EGFR/EPHA3/ESR1/FGA/FGF10/SBNO2/MLC1/GJB2/GNAS/GPER1/FFAR2/GSTP1/NR4A1/HSD17B2/HTR5A/IL1RN/IL6/IL10RA/IL12RB2/INHBA/ISL1/KCNJ8/LGALS9/LOX/ATP1A2/OPRL1/P2RY6/LEF1/PITX2/PON1/MAPK3/PTGFR/BGLAP/NOD2/BMP4/SLC9A3/SOX9/TIMP3/TLR5/TNFAIP3/WNT10B/RAB7A/ZC3H12A/CALR/NR0B2/TRIM63/MGARP/TNFRSF11A/ALDH1A2/ADIPOQ/LY86/NR1H4	65

GO:0051171	regulation of nitrogen compound metabolic process	253/901	3889/17046	8.90E-05	0.00361	0.00284	ZNF783/CDH13/MBNL2/CDKN1C/C1D/ZBTB18/DMRT2/CELF1/TBR1/NPFFR2/PNRC1/TMED10/HNRNPUL1/ERLIN2/PSIP1/EGLN2/ZBED9/CIDEA/ZFP42/ADM/IL31RA/ZNF358/ZNF738/SMYD1/CYLD/ADRB3/ESCO2/ZNF782/ZNF709/ZNF781/CITED4/RNF168/ZNF366/BHLHA15/DNMT3A/DRD4/EEF2/EGFR/EGR3/PATL2/EIF4G1/ELK4/ESR1/SP8/FGF10/FHIT/XRN2/SBNO2/TRAK1/MSRB2/ACIN1/FOXL1/FOXC2/FOXO1/SPG20/NEDD4L/LARP1/PUM2/RYBP/VGLL2/MTOR/GABBR1/ZNF549/PNKD/GAPDHS/PABPC1/DNAJC2/DKK3/BMP10/ZNF638/GNAS/ZNF311/ZNF844/GPER1/ZBTB44/DNAJC15/GTF2B/BRF1/GUCY1A3/GZMA/SOX8/NRG1/HLX/HMGA1/NR4A1/HPCA/HOXB3/HOXC4/HOXC5/HOXC6/HOXD3/HSP90AA1/HSP90AB1/TFAP2E/ID3/BARHL2/IGF1/IGF2/CYR61/IL6/IL16/FOXK2/INHBA/IRF1/ISL1/JUP/USP50/HILS1/HES5/AFF3/LDLR/LGALS9/LHCGR/LMNA/LMO2/SMAD3/MC2R/ME1/ME2/MEF2D/MEOX1/MEOX2/MITF/LHX8/MOV10/NFATC3/NFYB/NHLH2/NPPC/NTF3/OPRL1/PALM/PARK2/LEF1/PRR16/SIRT6/PGAM2/PITX2/PKHD1/PLAGL1/PML/RIPPLY3/RIPK4/TLR9/CYTL1/POMC/POU2AF1/BNC2/MED18/BANP/PIWIL2/ELP3/PRMT6/DNAJC17/ZNF532/CNOT11/PRKD1/MYNN/MAPK3/MRAP/PRMT8/HTRA1/PSMB4/PAK6/ARNTL2/RGMA/PRDM11/PSMD7/TENM2/GATAD2B/METTL14/CCAR2/CREBZF/TRIM27/SCT/NPAS3/NOD2/SFRP2/TRA2B/GZF1/SGK1/BMP4/ZNF649/BMPR1B/BRD9/ZSCAN18/SOX9/STAT2/STK3/SUPT6H/TAF4B/TBP/TCEA1/TCEB2/ZEB1/TEAD3/TERF1/TLE3/TLR5/TNFAIP3/TNFRSF1A/TRAF1/TRAF5/TWIST1/CCR2/TNFRSF4/UCP1/VARS/WNT10B/ZNF7/ZNF124/ZNF177/PAX8/FZD5/CARD14/ZNF665/ZC3H14/ZNF606/ZC3H12A/CPEB4/ZNF436/CALR/SLIRP/SCRT1/HIST1H3A/SLA2/ZNF397/NR0B2/HOPX/KDM2B/LOXL3/CBX2/GAS7/RUNX1/TP63/RUNX3/ACTN1/FADD/TNFRSF11A/SPHK1/BUD31/CCNA1/LIMD1/PIAS2/LDB2/CBFA2T2/AURKB/ADIPOQ/H2AFY/MICAL2/VGLL4/ZBTB39/NR1H4	253
GO:0022604	regulation of cell morphogenesis	44/901	457/17046	9.06E-05	0.00365	0.00287	PDPN/TBR1/SH3D19/CYLD/COCH/UNC13D/EPHA3/FGA/SPG20/EPB41L3/NEDD4L/ARHGEF18/GAS2/BARHL2/IL6/ITGA7/ITGB2/KDR/ARHGDIA/SMAD3/MFI2/PALM/PARK2/LEF1/SSH1/FBLIM1/PALMD/PARVA/MAP2K2/CDC42SE1/ERMN/PXN/CCL11/SFRP2/TWIST1/PAX8/LST1/CALR/CAPZB/TTBK1/GAS7/LIMD1/RAPGEF2/ULK2	44
GO:1901701	cellular response to oxygen-containing compound	79/901	975/17046	9.23E-05	0.00369	0.0029	TCIRG1/SPON2/GJB6/ADCY3/AP3S1/CPS1/CYP11A1/DNMT3A/EGFR/EGR3/EIF4G1/EPHA3/ESR1/FGF10/RASA3/SBNO2/FOXC2/FOXO1/AKR1B1/MLC1/MTOR/FGF22/GNAS/GPER1/FFAR2/GRB10/GSTP1/NRG1/NR4A1/IGF2/IL1R1/IL1RN/IL6/AQP2/INHBA/AQP9/JUP/IPO5/LCK/NRAS/P2RY6/PARK2/PIK3CG/PRKAG3/TLR9/SSH1/SYBU/PRKAR1B/LMBRD1/MAPK3/MAP2K2/PSMB4/PSMD7/PTGFR/PTPRE/PXN/RASGRF2/BGLAP/NOD2/SLC8A1/SOX9/VAMP2/ZEB1/TLR5/TNFAIP3/TRPC6/WNT10B/CACNA1E/ZC3H12A/CPEB4/TRIM63/MGARP/IRS2/ALDH1A2/ADIPOQ/LY86/RAPGEF2/FGF19/NR1H4	79
GO:0001649	osteoblast differentiation	24/901	194/17046	9.31E-05	0.0037	0.00291	GNAS/SOX8/ID3/RSPO2/IGF1/CYR61/IL6/SMAD3/MEF2D/NPPC/ATP5B/LEF1/PRKD1/RDH14/BGLAP/SFRP2/BMP4/BMPR1B/TWIST1/WNT10B/IFITM1/TP63/LIMD1/PIAS2	24
GO:0002520	immune system development	65/901	763/17046	9.51E-05	0.00375	0.00295	ABI1/CDKN1C/CCR1/IL31RA/CYLD/ESCO2/RNF168/EEF2/EFNA2/EGR3/EML1/FGF10/SBNO2/ACIN1/FOXL1/MTOR/GNAS/FLVCR1/ANXA2/HLA-B/HLA-DOA/HLX/HOXB3/IL6/INHBA/IRF1/KDR/HES5/LCK/LGALS9/LMO2/LTB/SMAD3/MEOX1/MITF/NFATC3/LEF1/PITX2/PML/HERC6/SMPD3/BGLAP/SFRP2/CXCR5/VPS33A/BMP4/STK3/SUPT6H/TCEA1/ZEB1/TNFAIP3/WNT10B/ZAP70/FZD5/C6orf25/SCIN/RUNX1/RUNX3/ACTN1/FADD/TNFRSF11A/RSAD2/CD8A/ADIPOQ/CD79A	65
GO:0051174	regulation of phosphorus metabolic process	117/901	1579/17046	9.68E-05	0.00379	0.00298	ABI1/FARP1/CDKN1C/NPFFR2/ADCY3/CHI3L1/CCR1/MAP3K8/ADM/IL31RA/CTGF/ADRB3/NLRP6/DLG2/DRD4/EGFR/EPHA1/FGA/FGF10/RASA3/PPM1E/FOXO1/MAPK8IP2/MTOR/GABBR1/GAPDHS/FGF22/NPTN/BMP10/GNAS/GPER1/DOK7/GRB10/DNAJC15/GSTP1/GUCY1A3/ANXA2/NRG1/HPCA/HRH1/HSP90AB1/IGF1/IGF2/CYR61/IL1RN/IL6/INHBA/ISL1/ITGB2/KDR/HES5/LCK/LDLR/LGALS9/LHCGR/SMAD3/MC2R/ME1/ME2/MAP3K1/NPPC/NRAS/NTF3/OPRL1/PALM/PARK2/ANGPT4/SIRT6/PGAM2/PIK3CG/PKHD1/PLA2G2A/PML/TLR9/ELP3/PPP2R2B/VAC14/PRKAR1B/PRKD1/MAPK3/MAP2K2/MRAP/SLAMF8/PSMB4/PAK6/PSMD7/PXN/RASGRF2/TRIM27/SCT/CCL11/CCL17/NOD2/SFRP2/BMP4/SOX9/STK3/STK10/TNFAIP3/TNFRSF1A/TNXB/TWIST1/CCR2/TNFRSF4/YWHAG/CXCR4/FZD5/CARD14/CDK10/IRS2/TNFRSF11A/SPHK1/MAP3K6/ADIPOQ/H2AFY/RAPGEF2/FGF19	117
GO:0002683	negative regulation of immune system process	36/901	349/17046	9.95E-05	0.00386	0.00304	IL31RA/CYLD/NLRP6/A2M/GPER1/HLA-B/HLA-DOA/HLA-E/HLX/ZC3H12D/INHBA/IRF1/HES5/LGALS9/NOV/IL20RB/TLR9/PAG1/MASP1/HTRA1/PSMB4/TRIM27/NOD2/BMP4/SOX9/BPI/BST2/TNFAIP3/CCR2/LST1/NLRX1/ZC3H12A/SLA2/RUNX1/FADD/ADIPOQ	36

GO:0008152	metabolic process	624/901	10817/17046	9.98E-05	0.00386	0.00304	AKT3/ABI1/CDH3/GNE/ZNF783/TSPAN5/CDH13/SUGP2/MBNL2/FARP1/RCAN2/CDKN1C/SPEG/BCKDK/TCIRG1/ABCA9/C1D/ZBTB18/PITRM1/MTHFS/PDPN/DMRT2/CELF1/CELF2/TBR1/HCST/NPFFR2/ADCY3/PNRC1/TMED10/SLC27A2/LECT1/ADAM29/HNRNPUL1/RPP14/HIBADH/CHI3L1/ERLIN2/PSIP1/CHI3L2/EGLN2/B4GALT7/KIF12/ACOT7/EXOC3/ADPRHL1/CARD16/ZBED9/CIDEA/GBP4/ALPK2/GALNT15/CLCA1/CLN5/MRPL52/CCR1/SLC51B/CNP/AADACL3/APOA1BP/NEU4/COL9A3/COL11A1/GALM/MAP3K8/ZFP42/ADM/IL31RA/EGFLAM/UBLCP1/HUS1B/CPD/CPM/CPS1/NDUFAF6/PXDNL/CRABP1/ZNF358/TRPM6/MIB2/PARP4/LDLRAD3/B3GLCT/MGAT5B/CSTA/KLC3/ZNF738/CTGF/SMYD1/PPM1L/SH3D19/CYB561/CYLD/MBOAT1/ADRB3/ESCO2/CYP11A1/ZNF782/FITM1/ADAL/ZNF709/ZNF781/CITED4/DDB1/LONRF2/DDOST/RNF168/ZNF366/BHLHA15/NLRP6/DIO3/DLG2/DNAH6/DNAH8/DNMT3A/ABAT/DPH1/DRD4/ECE1/AGXT/EEF2/EGFR/EGR3/PATL2/EIF4G1/A2M/ELK4/LIPH/DNAH12/ENO2/ADCK5/EPHA1/EPHA3/EPHB4/ESR1/ALAS1/F11/FAH/SPATA13/PRSS54/RNF182/PHACTR1/SP8/FGA/FGF10/FHIT/XRN2/RASA3/PPM1E/SBNO2/TRAK1/MSRB2/ACIN1/FOXL1/FOXC2/TBC1D9B/FOXO1/AKR1B1/GGA3/DIP2A/FLOT2/TBC1D1/NUP210/ATP11A/NEDD4L/PSD3/LARP1/PUM2/ARHGEF18/RYBP/MORC3/MAPK8IP2/TSSK2/VGLL2/MTOR/FUCA1/SLC37A4/GABBR1/RASGEF1C/RNF144B/ZNF549/ST6GALNAC3/GAK/SAMM50/ALS2CL/PNKD/TENM4/ACOT11/LTN1/RGS22/STEAP2/FBXL21/FBXO2/LCE2B/SACS/GATM/GBGT1/GAPDHS/SLC17A5/RPS6KC1/PABPC1/DNAJC2/FGF22/NPTN/AMPD2/PDE7B/DKK3/CYTH4/GLS2/VPS4A/AMPD3/DHDH/BMP10/ZNF638/GNAS/ZNF311/TMPRSS12/PIGW/ZNF844/THEM5/GPER1/EOGT/DOK7/GRB10/MRPS18B/ZBTB44/DNAJC15/GSTP1/GTF2B/BRF1/GUCY1A3/NME7/PADI1/GZMA/ANXA2/HAS1/SERPIND1/SOX8/NRG1/HK1/HLX/HMGA1/NR4A1/ACACB/HPCA/APBA2/HOXB3/HOXC4/HOXC5/HOXC6/HOXD3/AGFG2/HRH1/HSD11B1/HSD17B2/ACADL/HSPA1L/HSP90AA1/HSP90AB1/DUPD1/ADAMTSL5/TFAP2E/ID3/ZC3H12D/COL28A1/FMN1/BARHL2/NME9/IGF1/IGF2/CYR61/LCE1C/LCE1D/LCE2D/IL1RN/IL6/IL12RB2/PRSS41/IL16/FOXK2/INHBA/INPP5A/IRF1/AQP9/ISL1/ITGB2/ITIH3/ITIH4/IVL/JUP/USP50/HILS1/ATP9B/KCNH2/KDR/ACAT1/KIF25/IPO5/KRT15/HES5/AFF3/LCK/MUC21/LDLR/ARHGDIA/LGALS9/LHCGR/LLGL1/LMNA/LMO2/LOX/LTB/SMAD3/MC2R/ME1/ME2/MEF2D/MAP3K1/MEOX1/MEOX2/MFI2/MFNG/MGAT1/MITF/LHX8/MOCS1/MOV10/PLEKHG7/NUDT1/MYH4/NUBP1/NDUFB4/DRG1/NEU1/ATP1A2/NFATC3/NFYB/NHLH2/NOV/NPPC/NRAS/NTF3/OAS2/OPRL1/PAFAH2/ATP5B/PALM/ARHGEF3/PARK2/SPOCK3/UTP11L/LEF1/DDX47/PRR16/CHST15/ANGPT4/PDE4C/PCYOX1/PDE7A/SIRT6/PDE6B/ATP8A2/GALNT7/PGAM2/PI3/PIGC/PIK3CG/PITX2/PKHD1/PKM/PLA2G2A/PLAGL1/PRKAG3/PML/RIPPLY3/SLCO1C1/PNLIP/RIPK4/TLR9/TREM1/CYTL1/POMC/SSH1/PON1/RIN2/MOV10L1/POU2AF1/ZDHHC13/BNC2/MED18/CYP2W1/RPP25/LPCAT2/BANP/PPP1CB/HERC6/PPP1CC/PIWIL2/ELP3/ARHGEF10L/PRMT6/DNAJC17/ZNF532/PPP2R2B/FANCI/WDR33/SMPD3/CNOT11/VAC14/CARKD/PRKAR1B/LMBRD1/CSGALNACT1/CISD1/PRKD1/WSB2/MYNN/APOBR/MAPK3/MAP2K2/PROC/MRAP/PRMT8/MASP1/HTRA1/SLAMF8/CDC42SE1/PSMB4/PAK6/ARNTL2/RGMA/PRDM11/PSMD7/PTGFR/PLEKHG5/TENM2/GATAD2B/KLHL8/RDH14/METTL14/MARK4/CCAR2/PTPRE/PXN/CREBZF/ABHD17C/ACTA2/RASGRF2/RFC2/TRIM27/RGR/RGS12/EXOC4/RPA3/RPL8/RPL29/SCT/CCL11/CCL17/ABHD4/MRPS14/PRSS22/NPAS3/NOD2/STRA6/SFRP2/MAP1LC3B2/ARHGAP9/TRA2B/GZF1/DNAI2/SGK1/MICAL1/CERK/VPS33A/BMP4/SLC4A1/ZNF649/BMPR1B/BRD9/ZSCAN18/BOK/SOX9/SRP68/STAT2/STK3/STK10/SUPT6H/BST2/VAMP2/TAF4B/TBP/TCEA1/TCEB2/ZEB1/ACTC1/TEAD3/TERF1/TGM2/TIMP3/TLE3/TLR5/TNFAIP3/TNFRSF1A/TNXB/TRAF1/TRAF5/TRPM2/PHLDA2/TWIST1/CCR2/TNFRSF4/UCP1/UPP1/VARS/WNT10B/YWHAG/ZAP70/ZNF7/CA7/ZNF124/ZNF177/CACNA1E/PTP4A1/MOGS/PAX8/CXCR4/FZD5/RAB7A/ERI3/CARD14/GDPD3/CERS4/ZNF665/EPHX3/ZC3H14/ERMP1/TMEM62/ZNF606/ZC3H12A/FAAP100/CPEB4/COL18A1/ZNF436/EEPD1/CALR/QTRT1/SLIRP/CAPS/COLQ/CAST/SH3BGRL3/SCRT1/HIST1H3A/DYNLRB2/SLA2/ATP13A4/ZNF397/NR0B2/HOPX/TTBK1/SPINK7/TRIM63/KDM2B/LOXL3/GTPBP3/CBX2/RAE1/GAS7/CDK10/KMO/RUNX1/TP63/RUNX3/SERPINA6/IRS2/ACTN1/CRADD/FADD/TNFRSF11A/ALDH1A2/STK19/SYNJ2/SPHK1/BUD31/CCNA1/ENDOU/STBD1/LIMD1/CH25H/ERI1/STARD13/PIAS2/ZFAND2A/MAP3K6/LDB2/SLC16A3/CBFA2T2/RSAD2/AURKB/DAPL1/CCDC102A/NEURL3/SDR42E1/ADIPOQ/ARHGAP29/RAB3D/H2AFY/ENTPD3/PREPL/ARHGEF10/MICAL2/N4BP1/VGLL4/NUP93/RAPGEF2/ULK2/USP6NL/ZBTB39/RABGAP1L/IQSEC1/LPGAT1/FGF19/NR1H4	624
GO:0002062	chondrocyte differentiation	15/901	93/17046	0.0001	0.00392	0.00308	COL11A1/FAM101A/CTGF/SMAD3/MEF2D/NOV/NPPC/CYTL1/SFRP2/BMP4/BMPR1B/SOX9/WNT10B/SCIN/RUNX3	15
GO:0009117	nucleotide metabolic process	55/901	617/17046	0.0001	0.00392	0.00308	GNE/TCIRG1/NPFFR2/ADCY3/ACOT7/CNP/APOA1BP/ADM/ADRB3/ADAL/DLG2/DRD4/ENO2/FHIT/GABBR1/GAPDHS/AMPD2/PDE7B/AMPD3/GNAS/GPER1/DNAJC15/GUCY1A3/NME7/HK1/ACACB/HPCA/NME9/IGF1/LHCGR/MC2R/ME1/ME2/MGAT1/NUDT1/MYH4/NDUFB4/ATP1A2/NPPC/OAS2/OPRL1/ATP5B/PALM/PDE4C/PDE7A/SIRT6/PDE6B/PGAM2/PKM/CSGALNACT1/MRAP/SCT/CCR2/UPP1/KMO	55
GO:0006935	chemotaxis	67/901	796/17046	0.00011	0.004	0.00315	CDH13/SPON2/TBR1/CHGA/CCR1/COL9A3/EFNA2/EGFR/EGR3/EPHA1/EPHA3/EPHB4/FGF10/RASA3/NFASC/FGF22/FFAR2/SERPIND1/NRG1/NR4A1/HRH1/HSP90AA1/HSP90AB1/CYR61/IL6/IL16/ISL1/ITGB2/KDR/LGALS9/SMAD3/NOV/NRAS/NTF3/LEF1/PIK3CG/TREM1/PARVA/PRKD1/MAPK3/MAP2K2/PSMB4/RGMA/TRPC7/PSMD7/PLEKHG5/TENM2/RASGRF2/CCL11/CCL17/NOD2/CXCR5/BMP4/BMPR1B/SLIT1/TRPC4/TRPC6/CCR2/CACNB2/CXCR4/CALR/RUNX3/IRS2/TNFRSF11A/CCRL2/RAPGEF2/FGF19	67
GO:0042330	taxis	67/901	796/17046	0.00011	0.004	0.00315	CDH13/SPON2/TBR1/CHGA/CCR1/COL9A3/EFNA2/EGFR/EGR3/EPHA1/EPHA3/EPHB4/FGF10/RASA3/NFASC/FGF22/FFAR2/SERPIND1/NRG1/NR4A1/HRH1/HSP90AA1/HSP90AB1/CYR61/IL6/IL16/ISL1/ITGB2/KDR/LGALS9/SMAD3/NOV/NRAS/NTF3/LEF1/PIK3CG/TREM1/PARVA/PRKD1/MAPK3/MAP2K2/PSMB4/RGMA/TRPC7/PSMD7/PLEKHG5/TENM2/RASGRF2/CCL11/CCL17/NOD2/CXCR5/BMP4/BMPR1B/SLIT1/TRPC4/TRPC6/CCR2/CACNB2/CXCR4/CALR/RUNX3/IRS2/TNFRSF11A/CCRL2/RAPGEF2/FGF19	67
GO:0048878	chemical homeostasis	77/901	949/17046	0.00011	0.00407	0.0032	TCIRG1/TRDN/GJB6/ADCY3/CLN5/CCR1/ADM/CPS1/DDB1/BHLHA15/DRD4/ESR1/FOXO1/NEDD4L/SLC37A4/STEAP2/NPTN/GNAS/GPER1/FFAR2/FLVCR1/GSTP1/ANXA6/HK1/ACADL/IGF1/IL1R1/IL6/AQP2/AQP5/AQP9/KCNH2/KDR/LCK/LDLR/MFI2/NUBP1/ATP1A2/OPRL1/ATP5B/PARK2/SIRT6/PDE6B/PIK3CG/PKHD1/PML/POMC/SLC30A10/CHRNA9/SYBU/PRKAR1B/PRKD1/SLAMF8/TRPC7/CCL11/ABHD4/SGK1/BMP4/SLC4A1/SLC8A1/SLC9A3/TGM2/TRPC4/TRPC6/CCR2/CA7/CACNA1E/CXCR4/RAB7A/CALR/ATP13A4/CASQ1/TP63/IRS2/SMDT1/ADIPOQ/MTL5	77
GO:0048514	blood vessel morphogenesis	45/901	475/17046	0.00011	0.00408	0.00321	CDH13/LECT1/ESM1/CHI3L1/ADM/CTGF/EGR3/EPHA1/EPHB4/FGF10/VASH1/FOXC2/ANXA2/NR4A1/HOXB3/CYR61/IL6/ISL1/ITGA7/KDR/MEOX2/NFATC3/NOV/ATP5B/LEF1/ANGPT4/PIK3CG/PITX2/PML/ROBO4/PARVA/PRKD1/CCL11/STRA6/SFRP2/BMP4/TNFAIP3/TWIST1/CCR2/FZD5/ZC3H12A/COL18A1/RUNX1/SPHK1/RAPGEF2	45
GO:0014706	striated muscle tissue development	34/901	324/17046	0.00011	0.00408	0.00321	SPEG/ZBTB18/CHRNA1/COL11A1/SMYD1/FOXC2/FLNB/VGLL2/TENM4/BMP10/SOX8/NRG1/HLX/ISL1/LMNA/SMAD3/MEF2D/MEOX2/MYL2/NFATC3/NRAS/LEF1/SIRT6/PITX2/PLAGL1/BIN3/BMP4/SLC8A1/ACTC1/TWIST1/WNT10B/CALR/CASQ1/ALDH1A2	34

GO:0006164	purine nucleotide biosynthetic process	28/901	246/17046	0.00011	0.00408	0.00321	TCIRG1/NPFFR2/ADCY3/ACOT7/ADM/ADRB3/DRD4/GABBR1/AMPD2/AMPD3/GNAS/GPER1/GUCY1A3/NME7/ACACB/HPCA/NME9/LHCGR/MC2R/NPPC/OAS2/OPRL1/ATP5B/PALM/PKM/MRAP/SCT/CCR2	28
GO:0072522	purine-containing compound biosynthetic process	29/901	259/17046	0.00011	0.00413	0.00324	TCIRG1/NPFFR2/ADCY3/ACOT7/ADM/ADRB3/ADAL/DRD4/GABBR1/AMPD2/AMPD3/GNAS/GPER1/GUCY1A3/NME7/ACACB/HPCA/NME9/LHCGR/MC2R/NPPC/OAS2/OPRL1/ATP5B/PALM/PKM/MRAP/SCT/CCR2	29
GO:0007010	cytoskeleton organization	80/901	997/17046	0.00011	0.00415	0.00326	ABI1/TRDN/TACC2/CNP/FAM101A/CTGF/SGOL1/SH3D19/CYLD/EML1/EPHA1/EPHA3/PHACTR1/FGF10/PPM1E/MSRB2/LIMCH1/EPB41L3/FLNB/SYNE1/ARHGEF18/MTOR/PLEK2/BMP10/TMOD4/FMN1/JUP/INSC/STMN1/LCP1/LLGL1/LMNA/SMAD3/MAP3K1/MYL2/NUBP1/NEDD9/NTF3/PALM/PARK2/ATP8A2/PKHD1/SSH1/PARVA/TTC17/BIN3/MAPK3/PAK6/ACTR3B/ERMN/MARK4/PXN/TRIM27/CCL11/PARVG/DNAI2/MICAL1/SOX9/BST2/ACTC1/TERF1/TCHH/TNXB/CALR/CAPZB/SH3BGRL3/ANTXR1/BFSP2/CASQ1/GAS7/SCIN/ACTN1/LIMD1/MAP7/PRC1/AURKB/TRIP10/ARHGEF10/MICAL2/IQSEC1	80
GO:0008284	positive regulation of cell proliferation	64/901	753/17046	0.00011	0.00415	0.00326	CDH3/CDH13/TCIRG1/ESM1/ADM/IL31RA/CTGF/EGFR/EGR3/EPHA1/ESR1/FGF10/AKR1B1/MTOR/BMP10/GPER1/ANXA2/SOX8/NRG1/HLA-DPA1/HLA-E/HLX/NR4A1/IGF1/IGF2/CYR61/IL6/IL12RB2/ISL1/KDR/HES5/NRAS/NTF3/LEF1/SIRT6/PITX2/PKHD1/PML/PRKD1/HTRA1/PTGFR/S100A6/CCL11/NOD2/SFRP2/BMP4/SOX9/TNFAIP3/TRAF5/TWIST1/CCR2/TNFRSF4/WNT10B/ZAP70/COL18A1/CALR/TP63/IRS2/FADD/TNFRSF11A/ALDH1A2/SPHK1/PRC1/FGF19	64
GO:0048589	developmental growth	48/901	519/17046	0.00012	0.00423	0.00333	CDKN1C/CELF1/CHRNA1/COMP/ADM/ADRB3/DIO3/DMBT1/ESR1/FGF10/FOXC2/SPG20/NEDD4L/TENM4/GATM/BMP10/GNAS/FLVCR1/NRG1/HLX/ACACB/APBA2/RSPO2/FMN1/BARHL2/IGF1/SMAD3/NOV/NPPC/PARK2/SIRT6/ATP8A2/PKM/BNC2/BIN3/CCL11/STRA6/SFRP2/BMP4/BMPR1B/SLIT1/SOX9/STK3/TIMP3/WNT10B/COLQ/HOPX/ULK2	48
GO:0046649	lymphocyte activation	50/901	548/17046	0.00012	0.00424	0.00334	MAP3K8/CYLD/DDOST/RNF168/EGR3/UNC13D/FGF10/FLOT2/MTOR/HLA-DOA/HLA-DPA1/HLA-E/HLX/ZC3H12D/IGF1/IGF2/IL6/INHBA/IRF1/ITGB2/LCK/LCP1/LGALS9/SMAD3/NFATC3/IL21R/LEF1/PIK3CG/IL20RB/APBB1IP/PAG1/NOD2/CXCR5/BMP4/SUPT6H/BST2/ZEB1/TNFAIP3/CCR2/TNFRSF4/ZAP70/FZD5/LST1/SLA2/IRS2/FADD/SKAP2/RSAD2/CD8A/CD79A	50
GO:0009891	positive regulation of biosynthetic process	123/901	1685/17046	0.00012	0.00424	0.00334	CDH3/CDH13/CDKN1C/DMRT2/TBR1/ERLIN2/PSIP1/SLC51B/ADM/IL31RA/CTGF/ADRB3/CITED4/BHLHA15/EEF2/EGFR/ESR1/FGF10/SBNO2/FOXC2/FOXO1/LARP1/VGLL2/MTOR/PABPC1/DNAJC2/BMP10/GNAS/GPER1/BRF1/GUCY1A3/SOX8/HMGA1/NR4A1/HPCA/HRH1/HSP90AA1/HSP90AB1/TFAP2E/BARHL2/IGF1/IGF2/CYR61/IL6/FOXK2/INHBA/IRF1/ISL1/JUP/KDR/HES5/LDLR/LGALS9/LHCGR/LMNA/LMO2/LTB/SMAD3/MC2R/MEF2D/MEOX1/MEOX2/MITF/NFATC3/NFYB/NHLH2/NPPC/NTF3/PARK2/LEF1/PRR16/PITX2/PLAGL1/RIPK4/TLR9/CYTL1/POMC/BANP/PIWIL2/PRKD1/MAPK3/MRAP/ARNTL2/RGMA/TRIM27/SCT/NPAS3/NOD2/SFRP2/BMP4/ZNF649/BMPR1B/SOX9/STK3/SUPT6H/TBP/TCEA1/TCEB2/ZEB1/TEAD3/TLR5/TNFRSF1A/TRAF1/TRAF5/TWIST1/CCR2/WNT10B/PAX8/FZD5/CARD14/CALR/RUNX1/TP63/RUNX3/IRS2/FADD/TNFRSF11A/SPHK1/PIAS2/LDB2/CBFA2T2/MICAL2/NR1H4	123
GO:0019725	cellular homeostasis	62/901	725/17046	0.00012	0.00435	0.00342	TCIRG1/TRDN/GJB6/EGLN2/CHRNA1/CLN5/CCR1/ADM/DRD4/ESR1/FOXO1/NEDD4L/NPTN/GPER1/FLVCR1/ANXA6/HK1/NME9/IL1R1/IL6/AQP2/AQP5/AQP9/LCK/NUBP1/ATP1A2/OPRL1/ATP5B/PARK2/PDE6B/PIK3CG/PKHD1/PML/SLC30A10/CHRNA9/SYBU/PRKD1/SLAMF8/TRPC7/RFC2/RPA3/CCL11/SGK1/BMP4/SLC4A1/SLC8A1/TERF1/TGM2/TRPC4/TRPC6/CCR2/CA7/CACNA1E/CXCR4/RAB7A/CALR/SH3BGRL3/ATP13A4/CASQ1/IRS2/SMDT1/MTL5	62
GO:0046058	cAMP metabolic process	20/901	150/17046	0.00013	0.00435	0.00342	NPFFR2/ADCY3/ADM/ADRB3/DRD4/GABBR1/PDE7B/GNAS/GPER1/HPCA/LHCGR/MC2R/NPPC/OPRL1/PALM/PDE4C/PDE7A/MRAP/SCT/CCR2	20
GO:0010562	positive regulation of phosphorus metabolic process	84/901	1062/17046	0.00013	0.00435	0.00342	ABI1/ADCY3/CHI3L1/CCR1/MAP3K8/ADM/IL31RA/CTGF/ADRB3/DRD4/EGFR/FGA/FGF10/RASA3/MAPK8IP2/MTOR/GAPDHS/FGF22/NPTN/BMP10/GNAS/GPER1/DOK7/GRB10/GUCY1A3/ANXA2/NRG1/HPCA/HRH1/HSP90AB1/IGF1/IGF2/CYR61/IL1RN/IL6/INHBA/ISL1/KDR/HES5/LCK/LGALS9/LHCGR/SMAD3/MC2R/MAP3K1/NPPC/NRAS/NTF3/OPRL1/ANGPT4/PIK3CG/PLA2G2A/TLR9/PRKAR1B/PRKD1/MAPK3/MAP2K2/MRAP/PSMB4/PAK6/PSMD7/PXN/RASGRF2/SCT/CCL11/CCL17/NOD2/SFRP2/BMP4/SOX9/STK3/STK10/TNFRSF1A/CXCR4/FZD5/CARD14/CDK10/IRS2/TNFRSF11A/SPHK1/MAP3K6/ADIPOQ/RAPGEF2/FGF19	84
GO:0045937	positive regulation of phosphate metabolic process	84/901	1062/17046	0.00013	0.00435	0.00342	ABI1/ADCY3/CHI3L1/CCR1/MAP3K8/ADM/IL31RA/CTGF/ADRB3/DRD4/EGFR/FGA/FGF10/RASA3/MAPK8IP2/MTOR/GAPDHS/FGF22/NPTN/BMP10/GNAS/GPER1/DOK7/GRB10/GUCY1A3/ANXA2/NRG1/HPCA/HRH1/HSP90AB1/IGF1/IGF2/CYR61/IL1RN/IL6/INHBA/ISL1/KDR/HES5/LCK/LGALS9/LHCGR/SMAD3/MC2R/MAP3K1/NPPC/NRAS/NTF3/OPRL1/ANGPT4/PIK3CG/PLA2G2A/TLR9/PRKAR1B/PRKD1/MAPK3/MAP2K2/MRAP/PSMB4/PAK6/PSMD7/PXN/RASGRF2/SCT/CCL11/CCL17/NOD2/SFRP2/BMP4/SOX9/STK3/STK10/TNFRSF1A/CXCR4/FZD5/CARD14/CDK10/IRS2/TNFRSF11A/SPHK1/MAP3K6/ADIPOQ/RAPGEF2/FGF19	84
GO:0031328	positive regulation of cellular biosynthetic process	121/901	1655/17046	0.00013	0.00441	0.00347	CDH3/CDH13/CDKN1C/DMRT2/TBR1/ERLIN2/PSIP1/SLC51B/ADM/IL31RA/ADRB3/CITED4/BHLHA15/EEF2/EGFR/ESR1/FGF10/SBNO2/FOXC2/FOXO1/LARP1/VGLL2/MTOR/PABPC1/DNAJC2/BMP10/GNAS/GPER1/BRF1/GUCY1A3/SOX8/HMGA1/NR4A1/HPCA/HRH1/HSP90AA1/HSP90AB1/TFAP2E/BARHL2/IGF1/IGF2/CYR61/IL6/FOXK2/INHBA/IRF1/ISL1/JUP/HES5/LDLR/LGALS9/LHCGR/LMNA/LMO2/LTB/SMAD3/MC2R/MEF2D/MEOX1/MEOX2/MITF/NFATC3/NFYB/NHLH2/NPPC/NTF3/PARK2/LEF1/PRR16/PITX2/PLAGL1/RIPK4/TLR9/CYTL1/POMC/BANP/PIWIL2/PRKD1/MAPK3/MRAP/ARNTL2/RGMA/TRIM27/SCT/NPAS3/NOD2/SFRP2/BMP4/ZNF649/BMPR1B/SOX9/STK3/SUPT6H/TBP/TCEA1/TCEB2/ZEB1/TEAD3/TLR5/TNFRSF1A/TRAF1/TRAF5/TWIST1/CCR2/WNT10B/PAX8/FZD5/CARD14/CALR/RUNX1/TP63/RUNX3/IRS2/FADD/TNFRSF11A/SPHK1/PIAS2/LDB2/CBFA2T2/MICAL2/NR1H4	121

GO:0018130	heterocycle biosynthetic process	253/901	3911/17046	0.00013	0.00447	0.00352	ZNF783/CDH13/CDKN1C/TCIRG1/C1D/ZBTB18/MTHFS/DMRT2/TBR1/NPFFR2/ADCY3/PNRC1/HNRNPUL1/ERLIN2/PSIP1/EGLN2/ACOT7/ZBED9/CIDEA/ZFP42/ADM/IL31RA/CPS1/ZNF358/ZNF738/CTGF/SMYD1/CYLD/ADRB3/ZNF782/ADAL/ZNF709/ZNF781/CITED4/DDB1/RNF168/ZNF366/BHLHA15/DNMT3A/DRD4/EGFR/EGR3/ELK4/ESR1/ALAS1/SP8/FGF10/FHIT/XRN2/SBNO2/TRAK1/MSRB2/FOXL1/FOXC2/FOXO1/NUP210/NEDD4L/RYBP/VGLL2/MTOR/GABBR1/ZNF549/DNAJC2/AMPD2/DKK3/GLS2/AMPD3/BMP10/ZNF638/GNAS/ZNF311/ZNF844/GPER1/ZBTB44/GTF2B/BRF1/GUCY1A3/NME7/SOX8/NRG1/HLX/HMGA1/NR4A1/ACACB/HPCA/HOXB3/HOXC4/HOXC5/HOXC6/HOXD3/TFAP2E/ID3/BARHL2/NME9/IGF1/IGF2/CYR61/IL6/IL16/FOXK2/INHBA/IRF1/ISL1/JUP/USP50/HILS1/HES5/AFF3/LGALS9/LHCGR/LMO2/SMAD3/MC2R/ME1/MEF2D/MEOX1/MEOX2/MITF/LHX8/MOCS1/MOV10/DRG1/NFATC3/NFYB/NHLH2/NPPC/NTF3/OAS2/OPRL1/ATP5B/PALM/PARK2/LEF1/SIRT6/PITX2/PKHD1/PKM/PLAGL1/PRKAG3/PML/RIPPLY3/RIPK4/TLR9/CYTL1/POMC/POU2AF1/BNC2/MED18/BANP/ELP3/PRMT6/DNAJC17/ZNF532/CNOT11/PRKD1/MYNN/MAPK3/MRAP/PRMT8/PAK6/ARNTL2/RGMA/PRDM11/TENM2/GATAD2B/CCAR2/CREBZF/RFC2/TRIM27/RGS12/RPA3/RPL8/RPL29/SCT/NPAS3/NOD2/SFRP2/GZF1/SGK1/BMP4/ZNF649/BMPR1B/BRD9/ZSCAN18/SOX9/STAT2/STK3/SUPT6H/TAF4B/TBP/TCEA1/TCEB2/ZEB1/TEAD3/TERF1/TLE3/TNFAIP3/TNFRSF1A/TRAF1/TRAF5/TWIST1/CCR2/TNFRSF4/UCP1/UPP1/WNT10B/YWHAG/ZNF7/ZNF124/ZNF177/PAX8/FZD5/CARD14/ZNF665/ZNF606/ZC3H12A/ZNF436/CALR/QTRT1/SLIRP/SCRT1/HIST1H3A/SLA2/ZNF397/NR0B2/HOPX/KDM2B/LOXL3/CBX2/RAE1/GAS7/KMO/RUNX1/TP63/RUNX3/ACTN1/FADD/TNFRSF11A/SPHK1/BUD31/CCNA1/LIMD1/PIAS2/LDB2/CBFA2T2/AURKB/ADIPOQ/H2AFY/MICAL2/VGLL4/NUP93/ZBTB39/NR1H4	253
GO:0043207	response to external biotic stimulus	67/901	802/17046	0.00013	0.00448	0.00352	SPON2/GJB6/HNRNPUL1/CHGA/CNP/ADM/CPS1/CYP11A1/COCH/NLRP6/DMBT1/UNC13D/FGA/FGF10/SBNO2/ACIN1/PUM2/SLC37A4/GSTP1/GUCY1A3/HLA-B/HLA-E/HMGA1/IL1RN/IL6/IL10RA/IL12RB2/IRF1/KCNJ8/STMN1/LCK/LGALS9/SMAD3/OAS2/PLA2G2A/PML/TLR9/TREM1/MAPK3/HTRA1/SLAMF8/PTGFR/CREBZF/ACTA2/DEFB134/CCL11/NOD2/BPI/STAT2/BST2/TLR5/TNFAIP3/TNFRSF1A/CA7/CXCR4/FZD5/NLRX1/ZC3H12A/UNC93B1/HIST1H3A/IFITM1/FADD/TNFRSF11A/RSAD2/CD8A/LY86/NUP93	67
GO:0051707	response to other organism	67/901	802/17046	0.00013	0.00448	0.00352	SPON2/GJB6/HNRNPUL1/CHGA/CNP/ADM/CPS1/CYP11A1/COCH/NLRP6/DMBT1/UNC13D/FGA/FGF10/SBNO2/ACIN1/PUM2/SLC37A4/GSTP1/GUCY1A3/HLA-B/HLA-E/HMGA1/IL1RN/IL6/IL10RA/IL12RB2/IRF1/KCNJ8/STMN1/LCK/LGALS9/SMAD3/OAS2/PLA2G2A/PML/TLR9/TREM1/MAPK3/HTRA1/SLAMF8/PTGFR/CREBZF/ACTA2/DEFB134/CCL11/NOD2/BPI/STAT2/BST2/TLR5/TNFAIP3/TNFRSF1A/CA7/CXCR4/FZD5/NLRX1/ZC3H12A/UNC93B1/HIST1H3A/IFITM1/FADD/TNFRSF11A/RSAD2/CD8A/LY86/NUP93	67
GO:0019438	aromatic compound biosynthetic process	253/901	3915/17046	0.00014	0.00469	0.00369	CDH3/ZNF783/CDH13/CDKN1C/TCIRG1/C1D/ZBTB18/MTHFS/DMRT2/TBR1/NPFFR2/ADCY3/PNRC1/HNRNPUL1/ERLIN2/PSIP1/EGLN2/ACOT7/ZBED9/CIDEA/ZFP42/ADM/IL31RA/ZNF358/ZNF738/CTGF/SMYD1/CYLD/ADRB3/ZNF782/ADAL/ZNF709/ZNF781/CITED4/DDB1/RNF168/ZNF366/BHLHA15/DNMT3A/DRD4/EGFR/EGR3/ELK4/ESR1/ALAS1/SP8/FGF10/FHIT/XRN2/SBNO2/TRAK1/MSRB2/FOXL1/FOXC2/FOXO1/NUP210/NEDD4L/RYBP/VGLL2/MTOR/GABBR1/ZNF549/DNAJC2/AMPD2/DKK3/GLS2/AMPD3/BMP10/ZNF638/GNAS/ZNF311/ZNF844/GPER1/ZBTB44/GTF2B/BRF1/GUCY1A3/NME7/SOX8/NRG1/HLX/HMGA1/NR4A1/ACACB/HPCA/HOXB3/HOXC4/HOXC5/HOXC6/HOXD3/TFAP2E/ID3/BARHL2/NME9/IGF1/IGF2/CYR61/IL6/IL16/FOXK2/INHBA/IRF1/ISL1/JUP/USP50/HILS1/HES5/AFF3/LGALS9/LHCGR/LMO2/SMAD3/MC2R/ME1/MEF2D/MEOX1/MEOX2/MITF/LHX8/MOV10/DRG1/NFATC3/NFYB/NHLH2/NPPC/NTF3/OAS2/OPRL1/ATP5B/PALM/PARK2/LEF1/SIRT6/PITX2/PKHD1/PKM/PLAGL1/PRKAG3/PML/RIPPLY3/RIPK4/TLR9/CYTL1/POMC/POU2AF1/BNC2/MED18/BANP/ELP3/PRMT6/DNAJC17/ZNF532/CNOT11/PRKD1/MYNN/MAPK3/MRAP/PRMT8/PAK6/ARNTL2/RGMA/PRDM11/TENM2/GATAD2B/CCAR2/CREBZF/RFC2/TRIM27/RGS12/RPA3/RPL8/RPL29/SCT/NPAS3/NOD2/SFRP2/GZF1/SGK1/BMP4/ZNF649/BMPR1B/BRD9/ZSCAN18/SOX9/STAT2/STK3/SUPT6H/TAF4B/TBP/TCEA1/TCEB2/ZEB1/TEAD3/TERF1/TLE3/TNFAIP3/TNFRSF1A/TRAF1/TRAF5/TWIST1/CCR2/TNFRSF4/UCP1/UPP1/WNT10B/YWHAG/ZNF7/ZNF124/ZNF177/PAX8/FZD5/CARD14/ZNF665/ZNF606/ZC3H12A/ZNF436/CALR/QTRT1/SLIRP/SCRT1/HIST1H3A/SLA2/ZNF397/NR0B2/HOPX/KDM2B/LOXL3/CBX2/RAE1/GAS7/KMO/RUNX1/TP63/RUNX3/ACTN1/FADD/TNFRSF11A/SPHK1/BUD31/CCNA1/LIMD1/PIAS2/LDB2/CBFA2T2/AURKB/ADIPOQ/H2AFY/MICAL2/VGLL4/NUP93/RAPGEF2/ZBTB39/NR1H4	253
GO:0006163	purine nucleotide metabolic process	45/901	480/17046	0.00014	0.00469	0.00369	TCIRG1/NPFFR2/ADCY3/ACOT7/ADM/ADRB3/DLG2/DRD4/ENO2/FHIT/GABBR1/GAPDHS/AMPD2/PDE7B/AMPD3/GNAS/GPER1/DNAJC15/GUCY1A3/NME7/HK1/ACACB/HPCA/NME9/IGF1/LHCGR/MC2R/NUDT1/MYH4/NDUFB4/ATP1A2/NPPC/OAS2/OPRL1/ATP5B/PALM/PDE4C/PDE7A/SIRT6/PDE6B/PGAM2/PKM/MRAP/SCT/CCR2	45
GO:0030155	regulation of cell adhesion	52/901	581/17046	0.00014	0.00475	0.00373	CDH13/MAP3K8/EGFLAM/CYLD/EGR3/UNC13D/EPHA1/EPHA3/FGA/FOXC2/FLOT2/MTOR/CYTH4/NRG1/HLA-DOA/HLA-DPA1/HLA-E/HLX/ZC3H12D/FMN1/IGF1/IGF2/CYR61/IL1RN/IL6/IRF1/KDR/LAMA3/LCK/ARHGDIA/LGALS9/SMAD3/MFI2/ATP5B/LEF1/PIK3CG/PML/IL20RB/APBB1IP/PPP1CB/PAG1/NOD2/SFRP2/BMP4/SOX9/ZEB1/TGM2/CCR2/ZAP70/CALR/FADD/ADIPOQ	52
GO:0034654	nucleobase-containing compound biosynthetic process	249/901	3846/17046	0.00014	0.00476	0.00374	ZNF783/CDH13/CDKN1C/TCIRG1/C1D/ZBTB18/DMRT2/TBR1/NPFFR2/ADCY3/PNRC1/HNRNPUL1/ERLIN2/PSIP1/EGLN2/ACOT7/ZBED9/CIDEA/ZFP42/ADM/IL31RA/ZNF358/ZNF738/CTGF/SMYD1/CYLD/ADRB3/ZNF782/ADAL/ZNF709/ZNF781/CITED4/DDB1/RNF168/ZNF366/BHLHA15/DNMT3A/DRD4/EGFR/EGR3/ELK4/ESR1/SP8/FGF10/FHIT/XRN2/SBNO2/TRAK1/MSRB2/FOXL1/FOXC2/FOXO1/NUP210/NEDD4L/RYBP/VGLL2/MTOR/GABBR1/ZNF549/DNAJC2/AMPD2/DKK3/GLS2/AMPD3/BMP10/ZNF638/GNAS/ZNF311/ZNF844/GPER1/ZBTB44/GTF2B/BRF1/GUCY1A3/NME7/SOX8/NRG1/HLX/HMGA1/NR4A1/ACACB/HPCA/HOXB3/HOXC4/HOXC5/HOXC6/HOXD3/TFAP2E/ID3/BARHL2/NME9/IGF1/IGF2/CYR61/IL6/IL16/FOXK2/INHBA/IRF1/ISL1/JUP/USP50/HILS1/HES5/AFF3/LGALS9/LHCGR/LMO2/SMAD3/MC2R/ME1/MEF2D/MEOX1/MEOX2/MITF/LHX8/MOV10/DRG1/NFATC3/NFYB/NHLH2/NPPC/NTF3/OAS2/OPRL1/ATP5B/PALM/PARK2/LEF1/SIRT6/PITX2/PKHD1/PKM/PLAGL1/PRKAG3/PML/RIPPLY3/RIPK4/TLR9/CYTL1/POMC/POU2AF1/BNC2/MED18/BANP/ELP3/PRMT6/DNAJC17/ZNF532/CNOT11/PRKD1/MYNN/MAPK3/MRAP/PRMT8/PAK6/ARNTL2/RGMA/PRDM11/TENM2/GATAD2B/CCAR2/CREBZF/RFC2/TRIM27/RGS12/RPA3/RPL8/RPL29/SCT/NPAS3/NOD2/SFRP2/GZF1/SGK1/BMP4/ZNF649/BMPR1B/BRD9/ZSCAN18/SOX9/STAT2/STK3/SUPT6H/TAF4B/TBP/TCEA1/TCEB2/ZEB1/TEAD3/TERF1/TLE3/TNFAIP3/TNFRSF1A/TRAF1/TRAF5/TWIST1/CCR2/TNFRSF4/UCP1/UPP1/WNT10B/YWHAG/ZNF7/ZNF124/ZNF177/PAX8/FZD5/CARD14/ZNF665/ZNF606/ZC3H12A/ZNF436/CALR/QTRT1/SLIRP/SCRT1/HIST1H3A/SLA2/ZNF397/NR0B2/HOPX/KDM2B/LOXL3/CBX2/RAE1/GAS7/KMO/RUNX1/TP63/RUNX3/ACTN1/FADD/TNFRSF11A/SPHK1/BUD31/CCNA1/LIMD1/PIAS2/LDB2/CBFA2T2/AURKB/ADIPOQ/H2AFY/MICAL2/VGLL4/NUP93/ZBTB39/NR1H4	249
GO:0009260	ribonucleotide biosynthetic process	28/901	251/17046	0.00016	0.00515	0.00405	TCIRG1/NPFFR2/ADCY3/ACOT7/ADM/ADRB3/DRD4/GABBR1/AMPD2/AMPD3/GNAS/GPER1/GUCY1A3/NME7/ACACB/HPCA/NME9/LHCGR/MC2R/NPPC/OPRL1/ATP5B/PALM/PKM/MRAP/SCT/CCR2/UPP1	28

GO:0071345	cellular response to cytokine stimulus	56/901	642/17046	0.00016	0.00515	0.00405	CHI3L1/CCR1/IL31RA/CYP11A1/EIF4G1/FGA/SBNO2/FLNB/NUP210/GPER1/GSTP1/HLA-B/HLA-DPA1/HLA-E/HLA-F/HSP90AB1/IL1R1/IL1RN/IL6/IL10RA/IL11RA/IL12RB2/IL15RA/IRF1/LGALS9/LTB/OAS2/IL21R/PARK2/LEF1/PML/IL20RB/MAPK3/PSMB4/PSMD7/CCL11/CCL17/CXCR5/SOX9/STAT2/BST2/TNFRSF1A/CCR2/TNFRSF4/CXCR4/CARD14/IL1F10/RAE1/IFITM1/FADD/TNFRSF11A/SPHK1/CCRL2/RSAD2/ADIPOQ/NUP93	56
GO:0001775	cell activation	71/901	868/17046	0.00016	0.00516	0.00406	CHGA/MAP3K8/IL31RA/CTGF/CYLD/WBP2NL/DDOST/RNF168/EGR3/A2M/UNC13D/FGA/FGF10/SBNO2/FLOT2/MTOR/GNAS/GPER1/SCG3/HLA-DOA/HLA-DPA1/HLA-E/HLX/ZC3H12D/IGF1/IGF2/IL6/INHBA/IRF1/ITGB2/LCK/LCP1/LGALS9/SMAD3/NFATC3/IL21R/LEF1/PIK3CG/IL20RB/APBB1IP/PAG1/MAPK3/TRPC7/SLURP1/PTPRE/NOD2/CXCR5/BMP4/BPI/SUPT6H/BST2/VAMP2/ZEB1/TNFAIP3/TRPC6/CCR2/TNFRSF4/ZAP70/CXCR4/FZD5/LST1/ZC3H12A/CAST/SLA2/IRS2/ACTN1/FADD/SKAP2/RSAD2/CD8A/CD79A	71
GO:0034097	response to cytokine	62/901	732/17046	0.00016	0.00522	0.00411	CHI3L1/CCR1/IL31RA/CYP11A1/DDOST/EIF4G1/FGA/SBNO2/FLNB/NUP210/GPER1/GSTP1/HLA-B/HLA-DPA1/HLA-E/HLA-F/HSP90AB1/IL1R1/IL1RN/IL6/IL10RA/IL11RA/IL12RB2/IL15RA/IRF1/ITIH4/AFF3/LGALS9/LTB/OAS2/IL21R/PARK2/LEF1/PML/IL20RB/MAPK3/PSMB4/PSMD7/CCL11/CCL17/CXCR5/SOX9/STAT2/BST2/TIMP3/TNFRSF1A/CCR2/TNFRSF4/CXCR4/CARD14/IL1F10/TRIM63/RAE1/IFITM1/FADD/TNFRSF11A/ALDH1A2/SPHK1/CCRL2/RSAD2/ADIPOQ/NUP93	62
GO:0060255	regulation of macromolecule metabolic process	327/901	5249/17046	0.00016	0.0053	0.00417	ABI1/CDH3/ZNF783/CDH13/MBNL2/FARP1/CDKN1C/C1D/ZBTB18/DMRT2/CELF1/TBR1/NPFFR2/ADCY3/PNRC1/TMED10/HNRNPUL1/CHI3L1/ERLIN2/PSIP1/EGLN2/CARD16/ZBED9/CIDEA/CCR1/SLC51B/MAP3K8/ZFP42/IL31RA/ZNF358/LDLRAD3/CSTA/ZNF738/CTGF/SMYD1/SH3D19/CYLD/ADRB3/ESCO2/ZNF782/ZNF709/ZNF781/CITED4/RNF168/ZNF366/BHLHA15/NLRP6/DLG2/DNMT3A/DRD4/ECE1/EEF2/EGFR/EGR3/PATL2/EIF4G1/A2M/ELK4/EPHA1/ESR1/SP8/FGA/FGF10/FHIT/XRN2/RASA3/PPM1E/SBNO2/TRAK1/MSRB2/ACIN1/FOXL1/FOXC2/FOXO1/SPG20/GGA3/DIP2A/FLOT2/NEDD4L/LARP1/PUM2/RYBP/MAPK8IP2/VGLL2/MTOR/RNF144B/ZNF549/FBXO2/GAPDHS/PABPC1/DNAJC2/FGF22/NPTN/DKK3/BMP10/ZNF638/GNAS/ZNF311/ZNF844/GPER1/DOK7/GRB10/ZBTB44/GSTP1/GTF2B/BRF1/GZMA/ANXA2/SERPIND1/SOX8/NRG1/HLX/HMGA1/NR4A1/APBA2/HOXB3/HOXC4/HOXC5/HOXC6/HOXD3/HSP90AB1/TFAP2E/ID3/COL28A1/BARHL2/IGF1/IGF2/CYR61/IL1RN/IL6/IL16/FOXK2/INHBA/IRF1/ISL1/ITGB2/ITIH3/ITIH4/JUP/USP50/HILS1/KDR/HES5/AFF3/LCK/LGALS9/LMNA/LMO2/LTB/SMAD3/MEF2D/MAP3K1/MEOX1/MEOX2/MFI2/MITF/LHX8/MOV10/NFATC3/NFYB/NHLH2/NOV/NPPC/NRAS/NTF3/OPRL1/PARK2/SPOCK3/LEF1/PRR16/ANGPT4/SIRT6/PGAM2/PI3/PIK3CG/PITX2/PKHD1/PLA2G2A/PLAGL1/PML/RIPPLY3/RIPK4/TLR9/CYTL1/POMC/SSH1/POU2AF1/BNC2/MED18/BANP/PPP1CB/PPP1CC/PIWIL2/ELP3/PRMT6/DNAJC17/ZNF532/PPP2R2B/FANCI/CNOT11/PRKAR1B/PRKD1/MYNN/MAPK3/MAP2K2/PRMT8/MASP1/HTRA1/PSMB4/PAK6/ARNTL2/RGMA/PRDM11/PSMD7/PTGFR/TENM2/GATAD2B/METTL14/CCAR2/PXN/CREBZF/ACTA2/RASGRF2/TRIM27/CCL11/CCL17/NPAS3/NOD2/SFRP2/TRA2B/GZF1/SGK1/BMP4/ZNF649/BMPR1B/BRD9/ZSCAN18/BOK/SOX9/STAT2/STK3/STK10/SUPT6H/BST2/TAF4B/TBP/TCEA1/TCEB2/ZEB1/ACTC1/TEAD3/TERF1/TIMP3/TLE3/TNFAIP3/TNFRSF1A/TNXB/TRAF1/TRAF5/PHLDA2/TWIST1/CCR2/TNFRSF4/UCP1/VARS/WNT10B/YWHAG/ZNF7/ZNF124/ZNF177/PAX8/CXCR4/FZD5/RAB7A/CARD14/ZNF665/ZC3H14/ZNF606/ZC3H12A/CPEB4/ZNF436/CALR/SLIRP/CAST/SCRT1/HIST1H3A/SLA2/ZNF397/NR0B2/HOPX/SPINK7/TRIM63/KDM2B/LOXL3/CBX2/GAS7/CDK10/RUNX1/TP63/RUNX3/SERPINA6/IRS2/ACTN1/CRADD/FADD/TNFRSF11A/ALDH1A2/SPHK1/BUD31/CCNA1/LIMD1/ERI1/PIAS2/ZFAND2A/MAP3K6/LDB2/CBFA2T2/AURKB/ADIPOQ/H2AFY/MICAL2/N4BP1/VGLL4/RAPGEF2/ZBTB39/FGF19/NR1H4	327
GO:0061138	morphogenesis of a branching epithelium	23/901	189/17046	0.00017	0.0053	0.00417	ADM/ESR1/FGF10/FOXC2/SOX8/RSPO2/IGF1/IL6/KDR/NFATC3/LEF1/PITX2/PML/PXN/CCL11/SFRP2/GZF1/BMP4/SOX9/TGM2/PAX8/FZD5/TP63	23
GO:0031348	negative regulation of defense response	18/901	130/17046	0.00017	0.0053	0.00417	NLRP6/A2M/GPER1/GSTP1/HLA-B/HLA-E/ISL1/LGALS9/SMAD3/NOV/IL20RB/HTRA1/PSMB4/NOD2/TNFAIP3/TNFRSF1A/NLRX1/ADIPOQ	18
GO:0035295	tube development	52/901	585/17046	0.00017	0.00536	0.00422	PDPN/CHI3L1/ADM/CPS1/ZNF358/CTGF/EGFR/ESR1/FGF10/FOXL1/FOXC2/AKR1B1/SOX8/HLX/HSD11B1/RSPO2/FMN1/IGF1/AQP2/KDR/ACAT1/INSC/HES5/LOX/SMAD3/NFATC3/LEF1/C11orf73/PITX2/PML/IFT122/MAPK3/MAP2K2/PXN/SCT/CCL11/STRA6/SFRP2/GZF1/BMP4/SLC8A1/SOX9/STK3/ZEB1/TGM2/TWIST1/PAX8/HOPX/KDM2B/TP63/ALDH1A2/MICAL2	52
GO:0009152	purine ribonucleotide biosynthetic process	27/901	240/17046	0.00018	0.00559	0.0044	TCIRG1/NPFFR2/ADCY3/ACOT7/ADM/ADRB3/DRD4/GABBR1/AMPD2/AMPD3/GNAS/GPER1/GUCY1A3/NME7/ACACB/HPCA/NME9/LHCGR/MC2R/NPPC/OPRL1/ATP5B/PALM/PKM/MRAP/SCT/CCR2	27
GO:0046390	ribose phosphate biosynthetic process	28/901	253/17046	0.00018	0.0056	0.0044	TCIRG1/NPFFR2/ADCY3/ACOT7/ADM/ADRB3/DRD4/GABBR1/AMPD2/AMPD3/GNAS/GPER1/GUCY1A3/NME7/ACACB/HPCA/NME9/LHCGR/MC2R/NPPC/OPRL1/ATP5B/PALM/PKM/MRAP/SCT/CCR2/UPP1	28
GO:0040007	growth	72/901	887/17046	0.00018	0.0056	0.0044	CDH13/CDKN1C/CELF1/ESM1/EGLN2/CHRNA1/COMP/ADM/CTGF/ADRB3/DIO3/DMBT1/ESR1/FGF10/XRN2/FOXC2/SPG20/EPB41L3/NEDD4L/MTOR/TENM4/GATM/BMP10/GNAS/FLVCR1/NRG1/HLX/ACACB/APBA2/ZC3H12D/RSPO2/FMN1/BARHL2/IGF1/CYR61/IL6/INHBA/SMAD3/MT1A/MYL2/NUBP1/NEDD9/NOV/NPPC/PARK2/LEF1/SIRT6/ATP8A2/PKM/PML/BNC2/PRMT6/BIN3/HTRA1/CCAR2/CCL11/NOD2/STRA6/SFRP2/SGK1/BMP4/BMPR1B/SLIT1/SOX9/STK3/BST2/TIMP3/WNT10B/COLQ/HOPX/SPHK1/ULK2	72
GO:0048534	hematopoietic or lymphoid organ development	61/901	720/17046	0.00018	0.00561	0.00441	ABI1/CDKN1C/CCR1/IL31RA/CYLD/ESCO2/EEF2/EFNA2/EGR3/EML1/FGF10/SBNO2/ACIN1/FOXL1/MTOR/GNAS/FLVCR1/ANXA2/HLA-B/HLA-DOA/HLX/HOXB3/IL6/INHBA/IRF1/KDR/HES5/LCK/LGALS9/LMO2/LTB/MEOX1/MITF/NFATC3/LEF1/PITX2/PML/HERC6/SMPD3/BGLAP/SFRP2/CXCR5/VPS33A/BMP4/STK3/TCEA1/ZEB1/WNT10B/ZAP70/FZD5/C6orf25/SCIN/RUNX1/RUNX3/ACTN1/FADD/TNFRSF11A/RSAD2/CD8A/ADIPOQ/CD79A	61

GO:0070372	regulation of ERK1 and ERK2 cascade	25/901	215/17046	0.00018	0.00561	0.00441	CHI3L1/CCR1/CTGF/NLRP6/EGFR/FGA/FGF10/GPER1/GSTP1/IGF1/CYR61/IL6/KDR/LGALS9/PKHD1/PLA2G2A/MAPK3/CCL11/CCL17/NOD2/BMP4/TNFRSF11A/ADIPOQ/RAPGEF2/FGF19	25
GO:0051094	positive regulation of developmental process	83/901	1059/17046	0.00019	0.00567	0.00446	DMRT2/CELF1/CHI3L1/CCR1/SEZ6/ADM/CTGF/SMYD1/DIO3/DMBT1/EGR3/EIF4G1/UNC13D/EPHA1/EPHA3/FGA/FGF10/ACIN1/FOXC2/NEDD4L/MTOR/TENM4/NPTN/BMP10/GNAS/GPER1/SOX8/NRG1/HLX/HMGA1/ACACB/HOXD3/IGF1/CYR61/IL6/INHBA/ISL1/KDR/AMIGO3/LCK/ARHGDIA/LGALS9/LMNA/SMAD3/NEU1/NPPC/PALM/PARK2/LEF1/ANGPT4/ATP8A2/PLA2G2A/PML/PRKD1/MAP2K2/CCL11/SFRP2/BMP4/SLC8A1/BMPR1B/SOX9/STK3/ZEB1/TEAD3/TNFAIP3/TWIST1/WNT10B/ZAP70/PAX8/CXCR4/ZC3H12A/CALR/HOPX/IFITM1/SCIN/RUNX1/TP63/FADD/SPHK1/CBFA2T2/ADIPOQ/H2AFY/RAPGEF2	83
GO:0007015	actin filament organization	31/901	293/17046	0.00019	0.00567	0.00446	ABI1/FAM101A/CTGF/EPHA1/PHACTR1/PPM1E/MSRB2/MTOR/TMOD4/FMN1/LCP1/SMAD3/MAP3K1/NEDD9/PARK2/SSH1/TTC17/BIN3/ACTR3B/ERMN/TRIM27/CCL11/MICAL1/ACTC1/CAPZB/SH3BGRL3/GAS7/SCIN/ACTN1/ARHGEF10/MICAL2	31
GO:0001763	morphogenesis of a branching structure	24/901	203/17046	0.00019	0.00567	0.00446	ADM/ESR1/FGF10/FOXC2/SOX8/RSPO2/IGF1/IL6/KDR/NFATC3/LEF1/PITX2/PML/ERMN/PXN/CCL11/SFRP2/GZF1/BMP4/SOX9/TGM2/PAX8/FZD5/TP63	24
GO:0006140	regulation of nucleotide metabolic process	24/901	203/17046	0.00019	0.00567	0.00446	NPFFR2/ADM/ADRB3/DRD4/GABBR1/GAPDHS/GNAS/GPER1/DNAJC15/GUCY1A3/HPCA/IGF1/LHCGR/MC2R/ME1/ME2/NPPC/OPRL1/PALM/SIRT6/PGAM2/MRAP/SCT/CCR2	24
GO:0007267	cell-cell signaling	89/901	1154/17046	0.00019	0.00567	0.00446	KCNMB2/NPFFR2/ADCY3/CHRNA1/CHRNA2/CHRNA5/PANX3/CCR1/SEZ6/CNP/ADM/CTGF/ADRB3/BHLHA15/DLG2/ABAT/DRD4/DTNA/EFNA2/EGR3/FGA/FGF10/FOXL1/MAPK8IP2/GABBR1/PNKD/GJA3/NPTN/GJB2/PDE7B/GLS2/GNAS/GPER1/FFAR2/GRIK4/SOX8/KCNIP2/APBA2/HRH1/HTR3A/CYR61/IL1RN/IL6/INHBA/ISL1/ITGB2/KCNH2/KCNJ8/KCNJ9/KCNMB1/LTB/MPZ/ATP1A2/NOV/NTF3/OPRL1/PARK2/PDE4C/POMC/SMPD3/CHRNA9/SYBU/PRKAR1B/RASGRF2/RIT2/SCT/CCL17/SFRP2/BMP4/SLC6A12/SOX9/BST2/VAMP2/YWHAG/CA7/CACNA1E/CACNB2/PAX8/FZD5/RAB11FIP1/COLQ/NR0B2/TP63/IRS2/TNFRSF11A/SYT7/ADIPOQ/RAB3D/RAPGEF2	89
GO:0009187	cyclic nucleotide metabolic process	23/901	191/17046	0.00019	0.00576	0.00453	NPFFR2/ADCY3/CNP/ADM/ADRB3/DRD4/GABBR1/AMPD2/PDE7B/GNAS/GPER1/GUCY1A3/HPCA/LHCGR/MC2R/NPPC/OPRL1/PALM/PDE4C/PDE7A/MRAP/SCT/CCR2	23
GO:0019219	regulation of nucleobase-containing compound metabolic process	235/901	3617/17046	0.00019	0.00576	0.00453	ZNF783/CDH13/MBNL2/CDKN1C/C1D/ZBTB18/DMRT2/CELF1/TBR1/NPFFR2/PNRC1/HNRNPUL1/ERLIN2/PSIP1/EGLN2/ZBED9/CIDEA/ZFP42/ADM/IL31RA/ZNF358/ZNF738/SMYD1/CYLD/ADRB3/ESCO2/ZNF782/ZNF709/ZNF781/CITED4/RNF168/ZNF366/BHLHA15/DNMT3A/DRD4/EGFR/EGR3/ELK4/ESR1/SP8/FGF10/FHIT/XRN2/SBNO2/TRAK1/MSRB2/ACIN1/FOXL1/FOXC2/FOXO1/SPG20/NEDD4L/RYBP/VGLL2/MTOR/GABBR1/ZNF549/GAPDHS/PABPC1/DNAJC2/DKK3/BMP10/ZNF638/GNAS/ZNF311/ZNF844/GPER1/ZBTB44/DNAJC15/GTF2B/BRF1/GUCY1A3/GZMA/SOX8/NRG1/HLX/HMGA1/NR4A1/HPCA/HOXB3/HOXC4/HOXC5/HOXC6/HOXD3/TFAP2E/ID3/BARHL2/IGF1/IGF2/CYR61/IL6/IL16/FOXK2/INHBA/IRF1/ISL1/JUP/USP50/HILS1/HES5/AFF3/LGALS9/LHCGR/LMNA/LMO2/SMAD3/MC2R/ME1/ME2/MEF2D/MEOX1/MEOX2/MITF/LHX8/MOV10/NFATC3/NFYB/NHLH2/NPPC/NTF3/OPRL1/PALM/PARK2/LEF1/SIRT6/PGAM2/PITX2/PKHD1/PLAGL1/PML/RIPPLY3/RIPK4/TLR9/CYTL1/POMC/POU2AF1/BNC2/MED18/BANP/ELP3/PRMT6/DNAJC17/ZNF532/CNOT11/PRKD1/MYNN/MAPK3/MRAP/PRMT8/HTRA1/PAK6/ARNTL2/RGMA/PRDM11/TENM2/GATAD2B/CCAR2/CREBZF/TRIM27/SCT/NPAS3/NOD2/SFRP2/TRA2B/GZF1/SGK1/BMP4/ZNF649/BMPR1B/BRD9/ZSCAN18/SOX9/STAT2/STK3/SUPT6H/TAF4B/TBP/TCEA1/TCEB2/ZEB1/TEAD3/TERF1/TLE3/TNFAIP3/TNFRSF1A/TRAF1/TRAF5/TWIST1/CCR2/TNFRSF4/UCP1/WNT10B/ZNF7/ZNF124/ZNF177/PAX8/FZD5/CARD14/ZNF665/ZC3H14/ZNF606/ZC3H12A/ZNF436/CALR/SLIRP/SCRT1/HIST1H3A/SLA2/ZNF397/NR0B2/HOPX/KDM2B/LOXL3/CBX2/GAS7/RUNX1/TP63/RUNX3/ACTN1/FADD/TNFRSF11A/SPHK1/BUD31/CCNA1/LIMD1/PIAS2/LDB2/CBFA2T2/AURKB/ADIPOQ/H2AFY/MICAL2/VGLL4/ZBTB39/NR1H4	235
GO:0006952	defense response	119/901	1639/17046	0.00019	0.00576	0.00453	ABI1/TANK/KLRG1/TCIRG1/SPON2/ADCY3/CHGA/CHI3L1/CCR1/MAP3K8/ADM/IL31RA/PARP4/CYLD/DDOST/COCH/NLRP6/DMBT1/DRD4/EGFR/EIF4G1/A2M/UNC13D/FCGR2A/FGA/FGF10/RASA3/SBNO2/ACIN1/FOXO1/PUM2/MAPK8IP2/MTOR/SLC37A4/FGF22/GPER1/FFAR2/GSTP1/NRG1/HLA-B/HLA-DPA1/HLA-E/HLA-F/NR4A1/HRH1/HSP90AA1/HSP90AB1/CD300E/IL1R1/IL1RN/IL6/INHBA/IRF1/ISL1/ITGB2/ITIH4/KCNJ8/LCK/LGALS9/SMAD3/MAP3K1/MOV10/NFATC3/NOV/NRAS/OAS2/PIK3CG/PLA2G2A/PML/IL20RB/TLR9/TREM1/PRKAR1B/PRKD1/MAPK3/MAP2K2/MASP1/HTRA1/SLAMF8/PSMB4/PSMD7/PTPRCAP/RASGRF2/TRIM27/DEFB134/CCL11/CCL17/NOD2/BMPR1B/BPI/STAT2/BST2/TLR5/TNFAIP3/TNFRSF1A/CCR2/TNFRSF4/ZAP70/CA7/CACNA1E/CXCR4/NLRX1/UNC93B1/HIST1H3A/IL1F10/IFITM1/IRS2/FADD/TNFRSF11A/SPHK1/CCRL2/RSAD2/IL32/CD8A/ADIPOQ/LY86/NUP93/RAPGEF2/FGF19	119
GO:0070873	regulation of glycogen metabolic process	8/901	32/17046	0.0002	0.00589	0.00463	MTOR/GRB10/IGF1/IGF2/POMC/PPP1CB/PHLDA2/IRS2	8
GO:0014032	neural crest cell development	10/901	49/17046	0.0002	0.00594	0.00467	FOXC2/SOX8/NRG1/ISL1/LEF1/PITX2/SOX9/TWIST1/ALDH1A2/FGF19	10

GO:0051704	multi-organism process	151/901	2174/17046	0.00021	0.00609	0.00479	ABI1/TCIRG1/SPON2/CELF1/GJB6/ADCY3/ADAM29/HNRNPUL1/CHGA/PSIP1/CNP/ZFP42/ADM/CPS1/CYP11A1/DDB1/WBP2NL/COCH/NLRP6/DMBT1/DNMT3A/ABAT/DRD4/EIF4G1/UNC13D/ESR1/FGA/FGF10/XRN2/SBNO2/ACIN1/AKR1B1/NUP210/NEDD4L/PUM2/MAPK8IP2/TSSK2/MTOR/SLC37A4/GAPDHS/GJB2/VPS4A/GNAS/IZUMO1/GSTP1/GTF2B/GUCY1A3/SOX8/HLA-B/HLA-E/HMGA1/HSPA1L/HSP90AB1/IGF1/IL1R1/IL1RN/IL6/IL10RA/IL12RB2/IL16/INHBA/IRF1/ITGB7/HILS1/KCNJ8/KDR/IPO5/KRT7/STMN1/LCK/LDLR/LGALS9/LHCGR/LMNA/SMAD3/NPPC/OAS2/PARK2/LEF1/PGAM2/PI3/PLA2G2A/SPA17/PML/TLR9/TREM1/MOV10L1/PIWIL2/PRMT6/WDR33/VAC14/LMBRD1/MAPK3/HTRA1/SLAMF8/PSMB4/PSMD7/PTGFR/CREBZF/ACTA2/TRIM27/RPL8/DEFB134/RPL29/CCL11/NPAS3/NOD2/BMP4/SLC20A2/BMPR1B/SOX9/BPI/STAT2/SUPT6H/BST2/TAF4B/TBP/TCEA1/TCEB2/TEAD3/TLR5/TNFAIP3/TNFRSF1A/CCR2/CA7/CXCR4/FZD5/RAB7A/CCDC86/NLRX1/ZC3H12A/CALR/UNC93B1/SLIRP/CAST/HIST1H3A/SPATA16/KDM2B/RAE1/IFITM1/TP63/FADD/TNFRSF11A/CCNA1/ENDOU/RSAD2/CD8A/LY86/MTL5/NUP93/USP6NL	151
GO:0034329	cell junction assembly	25/901	217/17046	0.00021	0.00611	0.0048	CDH3/CDH9/CDH12/CDH13/PKP3/MPP7/EPHA3/NFASC/EPB41L3/GJB2/FMN1/JUP/KDR/LAMA3/SMAD3/PLEC/FBLIM1/LIMS2/PARVA/PXN/FZD5/PARD6G/PARD6B/ACTN1/RAPGEF2	25
GO:0051272	positive regulation of cellular component movement	35/901	349/17046	0.00021	0.00619	0.00486	CDH13/PDPN/CCR1/EGFR/EPHA1/FGF10/FOXC2/GPER1/IGF1/CYR61/IL6/KDR/STMN1/LGALS9/SMAD3/NTF3/P2RY6/LEF1/ANGPT4/ELP3/PRKD1/MAP2K2/CCL11/BMP4/SLC8A1/SOX9/TWIST1/CCR2/PTP4A1/COL18A1/CALR/IRS2/FADD/SPHK1/RAPGEF2	35
GO:0044264	cellular polysaccharide metabolic process	14/901	89/17046	0.00023	0.00649	0.00511	CPS1/MTOR/GRB10/HAS1/IGF1/IGF2/PRKAG3/POMC/PPP1CB/PPP1CC/CSGALNACT1/PHLDA2/IRS2/STBD1	14
GO:0050776	regulation of immune response	72/901	894/17046	0.00023	0.00649	0.00511	ABI1/TANK/KLRG1/SPON2/HCST/MAP3K8/CYLD/COCH/NLRP6/DMBT1/EGFR/A2M/UNC13D/FCGR2A/FGF10/RASA3/FOXO1/PUM2/MTOR/FGF22/FFAR2/NRG1/HLA-B/HLA-DPA1/HLA-E/HLA-F/HLX/NR4A1/HSP90AA1/HSP90AB1/IL6/IRF1/ITGB2/ITGB7/LCK/LGALS9/SMAD3/MAP3K1/MOV10/NFATC3/NRAS/IL20RB/TLR9/PAG1/MAPK3/MAP2K2/MASP1/PSMB4/PSMD7/RASGRF2/TRIM27/NOD2/STAT2/SUPT6H/BST2/TLR5/TNFAIP3/CCR2/ZAP70/FZD5/NLRX1/UNC93B1/SLA2/IFITM1/IRS2/FADD/SKAP2/RSAD2/CD8A/RAPGEF2/CD79A/FGF19	72
GO:0055082	cellular chemical homeostasis	53/901	607/17046	0.00023	0.00652	0.00513	TCIRG1/TRDN/GJB6/CLN5/CCR1/ADM/DRD4/ESR1/FOXO1/NEDD4L/NPTN/GPER1/FLVCR1/ANXA6/HK1/IL1R1/AQP2/AQP5/AQP9/LCK/NUBP1/ATP1A2/OPRL1/ATP5B/PDE6B/PIK3CG/PKHD1/PML/SLC30A10/CHRNA9/SYBU/PRKD1/SLAMF8/TRPC7/CCL11/SGK1/BMP4/SLC4A1/SLC8A1/TGM2/TRPC4/TRPC6/CCR2/CA7/CACNA1E/CXCR4/RAB7A/CALR/ATP13A4/CASQ1/IRS2/SMDT1/MTL5	53
GO:0007167	enzyme linked receptor protein signaling pathway	89/901	1161/17046	0.00023	0.00656	0.00516	ABI1/CDH3/CDH13/CDKN1C/TCIRG1/ADCY3/LECT1/ESM1/CIDEA/AP3S1/IL31RA/CTGF/PPM1L/EFNA2/EGFR/EIF4G1/EPHA1/EPHA3/EPHB4/FGF10/RASA3/FOXC2/FOXO1/SPG20/NEDD4L/ARHGEF18/MTOR/FGF22/NPTN/BMP10/GPER1/GRB10/NRG1/NR4A1/HSP90AA1/IGF1/IGF2/CYR61/INHBA/JUP/KDR/HES5/LCK/ARHGDIA/LTBP1/SMAD3/MAP3K1/MOV10/NOV/NPPC/NRAS/NTF3/ARHGEF3/LEF1/ANGPT4/PRKAG3/PML/TLR9/PPP1CB/PPP1CC/PRKAR1B/LMBRD1/PAG1/PRKD1/MAPK3/MAP2K2/HTRA1/PSMB4/RGMA/PSMD7/PLEKHG5/PTPRE/PXN/RASGRF2/RIT2/SFRP2/BMP4/BMPR1B/SOX9/ZEB1/ZAP70/RAB7A/TMEM204/IRS2/SPHK1/CD8A/ADIPOQ/RAPGEF2/FGF19	89
GO:0048736	appendage development	21/901	169/17046	0.00023	0.00656	0.00516	COMP/ZNF358/ECE1/SP8/FGF10/GNAS/FLVCR1/RSPO2/FMN1/AFF3/MEOX2/LEF1/PITX2/IFT122/SFRP2/BMP4/BMPR1B/SOX9/TWIST1/TP63/ALDH1A2	21
GO:0060173	limb development	21/901	169/17046	0.00023	0.00656	0.00516	COMP/ZNF358/ECE1/SP8/FGF10/GNAS/FLVCR1/RSPO2/FMN1/AFF3/MEOX2/LEF1/PITX2/IFT122/SFRP2/BMP4/BMPR1B/SOX9/TWIST1/TP63/ALDH1A2	21
GO:1901699	cellular response to nitrogen compound	57/901	667/17046	0.00023	0.00656	0.00516	TCIRG1/ADCY3/AP3S1/CPS1/CYP11A1/DNMT3A/EGFR/EGR3/EIF4G1/FGF10/RASA3/FOXC2/FOXO1/AKR1B1/MTOR/FGF22/GNAS/GPER1/GRB10/NRG1/NR4A1/HRH1/IGF2/IL1RN/AQP9/JUP/IPO5/LCK/SMAD3/NRAS/PARK2/PIK3CG/PRKAG3/TLR9/SSH1/PRKAR1B/LMBRD1/MAPK3/MAP2K2/PSMB4/PSMD7/PTPRE/PXN/RASGRF2/NOD2/SLC8A1/SOX9/VAMP2/ZEB1/WNT10B/CPEB4/MGARP/IRS2/ADIPOQ/RAPGEF2/FGF19/NR1H4	57
GO:0006828	manganese ion transport	5/901	12/17046	0.00024	0.00658	0.00517	SLC30A10/TRPC7/TRPC4/TRPC6/TRPM2	5
GO:0048145	regulation of fibroblast proliferation	13/901	79/17046	0.00024	0.00658	0.00517	B4GALT7/EGFR/ESR1/FGF10/MORC3/GSTP1/ANXA2/IGF1/NRAS/SIRT6/PML/S100A6/SPHK1	13
GO:0065009	regulation of molecular function	175/901	2588/17046	0.00024	0.00661	0.00519	ABI1/CDH3/FARP1/RCAN2/CDKN1C/TRDN/PITRM1/NPFFR2/ADCY3/CHI3L1/CARD16/MAP3K8/CSTA/CTGF/CYLD/ADRB3/DLG2/DRD4/EGFR/A2M/EPHA1/EPHA3/ESR1/SPATA13/PHACTR1/FGF10/RASA3/PPM1E/TBC1D9B/TBC1D1/NEDD4L/PSD3/ARHGEF18/MAPK8IP2/MTOR/GABBR1/RASGEF1C/ALS2CL/RGS22/FGF22/CYTH4/GNAS/GPER1/DOK7/DNAJC15/GSTP1/GZMA/ANXA2/SERPIND1/KCNIP2/NRG1/HPCA/AGFG2/HSP90AA1/HSP90AB1/ID3/COL28A1/IGF1/IGF2/CYR61/IL1RN/IL6/ISL1/ITIH3/ITIH4/JUP/IPO5/LCK/ARHGDIA/LGALS9/LHCGR/LLGL1/SMAD3/MAP3K1/MFNG/PLEKHG7/ATP1A2/NHLH2/NRAS/NTF3/OPRL1/PALM/ARHGEF3/PARK2/SPOCK3/LEF1/ANGPT4/PI3/PIK3CG/PITX2/PKHD1/PML/FXYD6/PNLIP/RIPK4/TLR9/CYTL1/PON1/RIN2/ELP3/ARHGEF10L/PPP2R2B/VAC14/PRKAR1B/PRKD1/MAPK3/MAP2K2/PRMT8/SLAMF8/CDC42SE1/PSMB4/PAK6/PSMD7/PLEKHG5/CCAR2/PXN/CREBZF/RASGRF2/TRIM27/RGS12/CCL11/CCL17/NOD2/SFRP2/ARHGAP9/SGK1/BMP4/BOK/STK3/STK10/BST2/VAMP2/TCEA1/TERF1/TIMP3/TNFAIP3/TNXB/TRAF1/TRAF5/TRPC6/TWIST1/CCR2/TNFRSF4/WNT10B/YWHAG/CACNB2/CXCR4/FZD5/CARD14/ZC3H12A/CAST/SH3BGRL3/NR0B2/HOPX/SPINK7/TP63/SERPINA6/IRS2/CRADD/FADD/TNFRSF11A/SPHK1/STARD13/PIAS2/MAP3K6/AURKB/ADIPOQ/ARHGAP29/H2AFY/ARHGEF10/RAPGEF2/USP6NL/RABGAP1L/IQSEC1/FGF19	175

GO:0000904	cell morphogenesis involved in differentiation	76/901	958/17046	0.00024	0.00661	0.00519	FARP1/SPON2/TBR1/LECT1/FRMD6/CNP/COL9A3/EFNA2/EGFR/UNC13D/EPHA1/EPHA3/EPHB4/FGA/FGF10/RASA3/BTBD3/SPG20/NFASC/FLNB/NEDD4L/MAPK8IP2/FGF22/NRG1/HSP90AA1/HSP90AB1/FMN1/BARHL2/ISL1/ITGB7/STMN1/ARHGDIA/LLGL1/SMAD3/MAP3K1/MFI2/NRAS/NTF3/LEF1/ATP8A2/SSH1/PARVA/MAPK3/MAP2K2/PSMB4/RGMA/TRPC7/PSMD7/TENM2/PXN/RASGRF2/S100A4/S100A6/SFRP2/VPS33A/BMP4/BMPR1B/SLIT1/SOX9/TRPC4/TRPC6/TWIST1/CACNB2/PAX8/C6orf25/COL18A1/CALR/ANTXR1/PARD6B/LOXL3/RUNX3/IRS2/ACTN1/RAPGEF2/ULK2/FGF19	76
GO:0009790	embryo development	76/901	958/17046	0.00024	0.00661	0.00519	ABI1/CDKN1C/SPEG/ZBTB18/DMRT2/CELF1/GJB6/COL11A1/ZFP42/ADM/ZNF358/DMBT1/DNMT3A/ECE1/EGFR/SP8/FGF10/FOXC2/TENM4/GATM/GNAS/FLVCR1/SOX8/NRG1/HLX/APBA2/HOXB3/HOXC4/HOXC5/HOXC6/HOXD3/HSD17B2/ID3/RSPO2/IGF1/CYR61/IL1RN/INHBA/ISL1/ITGA7/ITGB2/KDR/HES5/RESP18/AFF3/LAMA3/LMO2/SMAD3/MEOX1/MEOX2/MGAT1/LEF1/ATP8A2/PITX2/RIPPLY3/CHRNA9/IFT122/SCT/STRA6/SFRP2/BMP4/SLC8A1/SOX9/STK3/ZEB1/PHLDA2/TWIST1/PAX8/FZD5/HOPX/KDM2B/RUNX1/TP63/ALDH1A2/ADIPOQ/MICAL2	76
GO:0071840	cellular component organization or biogenesis	351/901	5713/17046	0.00025	0.00674	0.0053	AKT3/ABI1/CDH3/CDH9/TSPAN5/CDH12/CDH13/FARP1/TCIRG1/TRDN/SPON2/C1D/TACC2/PDPN/CELF1/TBR1/SEPT9/ADCY3/TMED10/LECT1/RER1/ESM1/CHGA/PSIP1/PKP3/EGLN2/ATXN2L/B4GALT7/EXOC3/CHRNA1/CHRNA2/GPRIN1/CIDEA/C1QTNF7/PANX3/CLN5/MRPL52/FRMD6/SLC51B/SEZ6/CNP/APOA1BP/COL9A3/COL11A1/COMP/SCLT1/ZFP42/ADM/EGFLAM/NDUFAF6/MPP7/FAM101A/CTGF/SMYD1/SGOL1/SH3D19/CYLD/FITM1/DDB1/WBP2NL/DDOST/RNF168/BHLHA15/COCH/DLG2/DNMT3A/DSG3/EFNA2/EGFR/PATL2/A2M/ELK4/TMEM17/EML1/UNC13D/EPHA1/EPHA3/EPHB4/ESR1/ALAS1/SPATA13/PHACTR1/FGA/FGF10/RASA3/PPM1E/BTBD3/MSRB2/ACIN1/LIMCH1/FOXC2/SPG20/NFASC/EPB41L3/FLNB/FLOT2/MLC1/TBC1D1/NUP210/ATP11A/NEDD4L/SYNE1/PUM2/ARHGEF18/RYBP/MAPK8IP2/MTOR/SLC37A4/SAMM50/DFNB31/ALS2CL/TENM4/GAS2/SACS/PLEK2/DNAJC2/FGF22/NPTN/GJB2/GLS2/VPS4A/BMP10/PIGW/IZUMO1/GPR26/GPER1/MRPS18B/DNAJC15/TMOD4/NME7/ANXA2/HAS1/KCNIP2/NRG1/ANXA6/HK1/ANXA13/HMGA1/ACACB/HPCA/ACADL/HSPA1L/HSP90AA1/HSP90AB1/ZC3H12D/COL28A1/FMN1/BARHL2/IGF1/IGF2/CYR61/IL6/AQP2/AQP5/INHBA/AQP9/ISL1/ITGA7/ITGB2/ITGB7/JUP/USP50/HILS1/ATP9B/KDR/ACAT1/KIF25/KRT15/AMIGO3/INSC/HES5/LAMA3/STMN1/LCP1/ARHGDIA/LLGL1/LMNA/LOX/LTBP1/SMAD3/ME1/MAP3K1/MFI2/MITF/MPZ/MYH4/MYL2/NUBP1/NEDD9/NEU1/NOV/NRAS/NTF3/ATP5B/ANO7/PALM/PARK2/UTP11L/LEF1/DDX47/PRR16/ANGPT4/C11orf73/SIRT6/ATP8A2/PKHD1/PLA2G2A/PLEC/PRKAG3/PML/TREM1/SSH1/FBLIM1/PALMD/LPCAT2/BANP/PIWIL2/ELP3/PRMT6/GOLPH3L/MIS18BP1/PEX26/LIMS2/PARVA/TTC17/IFT122/CSGALNACT1/PRKD1/BIN3/MAPK3/MAP2K2/TRPV5/PRMT8/HTRA1/SLAMF8/CDC42SE1/PSMB4/PAK6/RGMA/TRPC7/PSMD7/ACTR3B/TENM2/GATAD2B/ERMN/MARK4/CCAR2/PXN/RASGRF2/RFC2/TRIM27/EXOC4/RPA3/RPL8/RPL29/S100A4/S100A6/CCL11/MRPS14/PARVG/NOD2/SFRP2/DNAI2/SGK1/MICAL1/TMEM237/VPS33A/BMP4/BMPR1B/SLIT1/BRD9/SOX9/SRP68/STAT2/SUPT6H/BST2/VAMP2/TCEB2/ACTC1/TERF1/TCHH/TNFAIP3/TNFRSF1A/TNXB/TRAF1/TRPC4/TRPC6/TWIST1/WNT10B/YWHAG/CA7/CACNB2/PAX8/CXCR4/FZD5/RAB7A/ERI3/LST1/C6orf25/COL18A1/CALR/COL21A1/SLIRP/CAPS/COLQ/CAPZB/HIST1H2BM/SH3BGRL3/HIST1H3A/DYNLRB2/ANTXR1/BFSP2/CASQ1/HOPX/PARD6G/PARD6B/TTBK1/KDM2B/LOXL3/MGARP/CBX2/RAE1/GAS7/SCIN/TP63/RUNX3/IRS2/ACTN1/FADD/SPHK1/CCNA1/SKAP2/LIMD1/ERI1/MAP7/PRC1/SYT7/LDB2/CBFA2T2/AURKB/TRIP10/ADIPOQ/H2AFY/ARHGEF10/MICAL2/NUP93/RAPGEF2/ULK2/USP6NL/IQSEC1/FGF19	351
GO:0070588	calcium ion transmembrane transport	22/901	182/17046	0.00025	0.00674	0.0053	TRDN/C15orf27/TRPM6/TRPV3/DRD4/RASA3/ATP1A2/OPRL1/PIK3CG/TLR9/TRPV6/CHRNA9/TRPV5/TRPC7/BMP4/SLC8A1/TRPC4/TRPC6/TRPM2/CACNA1E/CACNB2/SMDT1	22
GO:0072132	mesenchyme morphogenesis	8/901	33/17046	0.00025	0.00674	0.0053	FOXC2/ISL1/SMAD3/LEF1/ACTA2/SOX9/ACTC1/TWIST1	8
GO:0006816	calcium ion transport	35/901	352/17046	0.00025	0.00674	0.0053	TRDN/CLCA1/CCR1/C15orf27/TRPM6/CTGF/TRPV3/BHLHA15/DRD4/RASA3/CRACR2B/GPER1/ANXA6/LCK/ATP1A2/NFATC3/OPRL1/PIK3CG/PML/TLR9/TRPV6/CHRNA9/PRKD1/TRPV5/TRPC7/TRIM27/BMP4/SLC8A1/TRPC4/TRPC6/TRPM2/CACNA1E/CACNB2/CASQ1/SMDT1	35
GO:1901137	carbohydrate derivative biosynthetic process	61/901	729/17046	0.00025	0.00674	0.0053	GNE/TCIRG1/NPFFR2/ADCY3/B4GALT7/ACOT7/GALNT15/SLC51B/NEU4/ADM/PARP4/B3GLCT/MGAT5B/ADRB3/ADAL/DDOST/DRD4/TRAK1/FOXL1/GABBR1/ST6GALNAC3/GBGT1/SLC17A5/AMPD2/AMPD3/GNAS/PIGW/GPER1/EOGT/GUCY1A3/NME7/HAS1/ACACB/HPCA/NME9/IGF1/MUC21/LHCGR/MC2R/MGAT1/NEU1/NPPC/OAS2/OPRL1/ATP5B/PALM/CHST15/SIRT6/GALNT7/PIGC/PKM/CYTL1/CSGALNACT1/MRAP/SCT/BMPR1B/CCR2/UPP1/MOGS/CALR/QTRT1	61
GO:0009607	response to biotic stimulus	68/901	837/17046	0.00026	0.00703	0.00553	SPON2/GJB6/HNRNPUL1/CHGA/CNP/ADM/CPS1/CYP11A1/COCH/NLRP6/DMBT1/UNC13D/FGA/FGF10/SBNO2/ACIN1/PUM2/SLC37A4/GSTP1/GUCY1A3/HLA-B/HLA-E/HMGA1/IL1RN/IL6/IL10RA/IL12RB2/IRF1/KCNJ8/STMN1/LCK/LGALS9/SMAD3/OAS2/PLA2G2A/PML/TLR9/TREM1/MAPK3/HTRA1/SLAMF8/PTGFR/CREBZF/ACTA2/DEFB134/CCL11/NOD2/SYNDIG1L/BPI/STAT2/BST2/TLR5/TNFAIP3/TNFRSF1A/CA7/CXCR4/FZD5/NLRX1/ZC3H12A/UNC93B1/HIST1H3A/IFITM1/FADD/TNFRSF11A/RSAD2/CD8A/LY86/NUP93	68
GO:1901135	carbohydrate derivative metabolic process	89/901	1167/17046	0.00027	0.00727	0.00572	GNE/TCIRG1/NPFFR2/ADCY3/LECT1/B4GALT7/ACOT7/GALNT15/SLC51B/NEU4/COL11A1/ADM/EGFLAM/PARP4/B3GLCT/MGAT5B/MBOAT1/ADRB3/ADAL/DDOST/DLG2/DNMT3A/DRD4/ENO2/TRAK1/FOXL1/AKR1B1/FUCA1/GABBR1/ST6GALNAC3/FBXO2/GBGT1/GAPDHS/SLC17A5/AMPD2/PDE7B/AMPD3/GNAS/PIGW/GPER1/EOGT/DNAJC15/GUCY1A3/NME7/HAS1/HK1/ACACB/HPCA/NME9/IGF1/ITIH3/ITIH4/MUC21/LHCGR/MC2R/MGAT1/NUDT1/MYH4/NDUFB4/NEU1/ATP1A2/NPPC/OAS2/OPRL1/ATP5B/PALM/SPOCK3/CHST15/PDE4C/PDE7A/SIRT6/PDE6B/GALNT7/PGAM2/PIGC/PKM/PLA2G2A/CYTL1/SMPD3/CSGALNACT1/MRAP/SCT/CERK/BMPR1B/CCR2/UPP1/MOGS/CALR/QTRT1	89
GO:1900034	regulation of cellular response to heat	12/901	70/17046	0.00028	0.00727	0.00572	NUP210/MTOR/DNAJC2/HSPA1L/HSP90AA1/HSP90AB1/C11orf73/MAPK3/CCAR2/RPA3/RAE1/NUP93	12
GO:0072521	purine-containing compound metabolic process	47/901	524/17046	0.00028	0.00735	0.00578	TCIRG1/NPFFR2/ADCY3/ACOT7/ADM/ADRB3/ADAL/DLG2/DNMT3A/DRD4/ENO2/FHIT/GABBR1/GAPDHS/AMPD2/PDE7B/AMPD3/GNAS/GPER1/DNAJC15/GUCY1A3/NME7/HK1/ACACB/HPCA/NME9/IGF1/LHCGR/MC2R/NUDT1/MYH4/NDUFB4/ATP1A2/NPPC/OAS2/OPRL1/ATP5B/PALM/PDE4C/PDE7A/SIRT6/PDE6B/PGAM2/PKM/MRAP/SCT/CCR2	47

GO:0016043	cellular component organization	344/901	5594/17046	0.00028	0.00735	0.00578	AKT3/ABI1/CDH3/CDH9/TSPAN5/CDH12/CDH13/FARP1/TCIRG1/TRDN/SPON2/TACC2/PDPN/CELF1/TBR1/SEPT9/ADCY3/TMED10/LECT1/RER1/ESM1/PSIP1/PKP3/EGLN2/ATXN2L/B4GALT7/EXOC3/CHRNA1/CHRNA2/GPRIN1/CIDEA/C1QTNF7/PANX3/CLN5/MRPL52/FRMD6/SLC51B/SEZ6/CNP/APOA1BP/COL9A3/COL11A1/COMP/SCLT1/ZFP42/ADM/EGFLAM/NDUFAF6/MPP7/FAM101A/CTGF/SMYD1/SGOL1/SH3D19/CYLD/FITM1/DDB1/WBP2NL/DDOST/RNF168/BHLHA15/COCH/DLG2/DNMT3A/DSG3/EFNA2/EGFR/PATL2/A2M/ELK4/TMEM17/EML1/UNC13D/EPHA1/EPHA3/EPHB4/ESR1/ALAS1/SPATA13/PHACTR1/FGA/FGF10/RASA3/PPM1E/BTBD3/MSRB2/ACIN1/LIMCH1/FOXC2/SPG20/NFASC/EPB41L3/FLNB/FLOT2/MLC1/TBC1D1/NUP210/ATP11A/NEDD4L/SYNE1/PUM2/ARHGEF18/RYBP/MAPK8IP2/MTOR/SLC37A4/SAMM50/DFNB31/ALS2CL/TENM4/GAS2/SACS/PLEK2/DNAJC2/FGF22/NPTN/GJB2/GLS2/VPS4A/BMP10/PIGW/IZUMO1/GPR26/GPER1/MRPS18B/DNAJC15/TMOD4/NME7/ANXA2/HAS1/KCNIP2/NRG1/ANXA6/HK1/ANXA13/HMGA1/ACACB/HPCA/ACADL/HSPA1L/HSP90AA1/HSP90AB1/ZC3H12D/COL28A1/FMN1/BARHL2/IGF1/IGF2/CYR61/IL6/AQP2/AQP5/INHBA/AQP9/ISL1/ITGA7/ITGB2/ITGB7/JUP/USP50/HILS1/ATP9B/KDR/ACAT1/KIF25/KRT15/AMIGO3/INSC/HES5/LAMA3/STMN1/LCP1/ARHGDIA/LLGL1/LMNA/LOX/LTBP1/SMAD3/ME1/MAP3K1/MFI2/MITF/MPZ/MYH4/MYL2/NUBP1/NEDD9/NEU1/NOV/NRAS/NTF3/ATP5B/ANO7/PALM/PARK2/LEF1/PRR16/ANGPT4/C11orf73/SIRT6/ATP8A2/PKHD1/PLA2G2A/PLEC/PRKAG3/PML/TREM1/SSH1/FBLIM1/PALMD/LPCAT2/BANP/PIWIL2/ELP3/PRMT6/GOLPH3L/MIS18BP1/PEX26/LIMS2/PARVA/TTC17/IFT122/CSGALNACT1/PRKD1/BIN3/MAPK3/MAP2K2/TRPV5/PRMT8/HTRA1/SLAMF8/CDC42SE1/PSMB4/PAK6/RGMA/TRPC7/PSMD7/ACTR3B/TENM2/GATAD2B/ERMN/MARK4/CCAR2/PXN/RASGRF2/RFC2/TRIM27/EXOC4/RPA3/RPL8/RPL29/S100A4/S100A6/CCL11/MRPS14/PARVG/NOD2/SFRP2/DNAI2/SGK1/MICAL1/TMEM237/VPS33A/BMP4/BMPR1B/SLIT1/BRD9/SOX9/SRP68/STAT2/SUPT6H/BST2/VAMP2/TCEB2/ACTC1/TERF1/TCHH/TNFAIP3/TNFRSF1A/TNXB/TRAF1/TRPC4/TRPC6/TWIST1/WNT10B/YWHAG/CA7/CACNB2/PAX8/CXCR4/FZD5/RAB7A/LST1/C6orf25/COL18A1/CALR/COL21A1/SLIRP/CAPS/COLQ/CAPZB/HIST1H2BM/SH3BGRL3/HIST1H3A/DYNLRB2/ANTXR1/BFSP2/CASQ1/HOPX/PARD6G/PARD6B/TTBK1/KDM2B/LOXL3/MGARP/CBX2/RAE1/GAS7/SCIN/TP63/RUNX3/IRS2/ACTN1/FADD/SPHK1/CCNA1/SKAP2/LIMD1/MAP7/PRC1/SYT7/CBFA2T2/AURKB/TRIP10/ADIPOQ/H2AFY/ARHGEF10/MICAL2/NUP93/RAPGEF2/ULK2/USP6NL/IQSEC1/FGF19	344
GO:0010468	regulation of gene expression	245/901	3819/17046	0.0003	0.0077	0.00606	CDH3/ZNF783/CDH13/MBNL2/CDKN1C/C1D/ZBTB18/DMRT2/CELF1/TBR1/PNRC1/HNRNPUL1/ERLIN2/PSIP1/EGLN2/ZBED9/CIDEA/CCR1/ZFP42/IL31RA/ZNF358/LDLRAD3/ZNF738/CTGF/SMYD1/CYLD/ZNF782/ZNF709/ZNF781/CITED4/RNF168/ZNF366/BHLHA15/DNMT3A/EEF2/EGFR/EGR3/PATL2/EIF4G1/A2M/ELK4/ESR1/SP8/FGF10/FHIT/XRN2/SBNO2/TRAK1/MSRB2/ACIN1/FOXL1/FOXC2/FOXO1/SPG20/DIP2A/FLOT2/NEDD4L/LARP1/PUM2/RYBP/VGLL2/MTOR/ZNF549/PABPC1/DNAJC2/DKK3/BMP10/ZNF638/GNAS/ZNF311/ZNF844/GPER1/ZBTB44/GTF2B/BRF1/SOX8/NRG1/HLX/HMGA1/NR4A1/APBA2/HOXB3/HOXC4/HOXC5/HOXC6/HOXD3/TFAP2E/ID3/BARHL2/IGF1/IGF2/CYR61/IL6/IL16/FOXK2/INHBA/IRF1/ISL1/JUP/USP50/HILS1/HES5/AFF3/LCK/LGALS9/LMNA/LMO2/SMAD3/MEF2D/MEOX1/MEOX2/MFI2/MITF/LHX8/MOV10/NFATC3/NFYB/NHLH2/NOV/NRAS/NTF3/PARK2/LEF1/PRR16/SIRT6/PITX2/PKHD1/PLAGL1/PML/RIPPLY3/RIPK4/TLR9/CYTL1/POMC/POU2AF1/BNC2/MED18/BANP/PPP1CB/PPP1CC/PIWIL2/ELP3/PRMT6/DNAJC17/ZNF532/CNOT11/PRKD1/MYNN/MAPK3/MAP2K2/PRMT8/MASP1/HTRA1/PAK6/ARNTL2/RGMA/PRDM11/PTGFR/TENM2/GATAD2B/METTL14/CCAR2/CREBZF/ACTA2/TRIM27/NPAS3/NOD2/SFRP2/TRA2B/GZF1/SGK1/BMP4/ZNF649/BMPR1B/BRD9/ZSCAN18/SOX9/STAT2/STK3/SUPT6H/TAF4B/TBP/TCEA1/TCEB2/ZEB1/ACTC1/TEAD3/TLE3/TNFAIP3/TNFRSF1A/TRAF1/TRAF5/PHLDA2/TWIST1/TNFRSF4/UCP1/VARS/WNT10B/ZNF7/ZNF124/ZNF177/PAX8/FZD5/CARD14/ZNF665/ZC3H14/ZNF606/ZC3H12A/CPEB4/ZNF436/CALR/SLIRP/SCRT1/HIST1H3A/SLA2/ZNF397/NR0B2/HOPX/TRIM63/KDM2B/LOXL3/CBX2/GAS7/RUNX1/TP63/RUNX3/FADD/TNFRSF11A/ALDH1A2/SPHK1/BUD31/CCNA1/LIMD1/ERI1/PIAS2/LDB2/CBFA2T2/AURKB/ADIPOQ/H2AFY/MICAL2/VGLL4/ZBTB39/FGF19/NR1H4	245
GO:0010675	regulation of cellular carbohydrate metabolic process	19/901	148/17046	0.0003	0.0077	0.00606	SLC51B/FOXO1/MTOR/GAPDHS/GPER1/GRB10/HRH1/IGF1/IGF2/IL6/LHCGR/PARK2/SIRT6/PGAM2/POMC/PPP1CB/PHLDA2/IRS2/ADIPOQ	19
GO:0035107	appendage morphogenesis	19/901	148/17046	0.0003	0.0077	0.00606	ZNF358/ECE1/SP8/FGF10/GNAS/FLVCR1/RSPO2/FMN1/AFF3/LEF1/PITX2/IFT122/SFRP2/BMP4/BMPR1B/SOX9/TWIST1/TP63/ALDH1A2	19
GO:0035108	limb morphogenesis	19/901	148/17046	0.0003	0.0077	0.00606	ZNF358/ECE1/SP8/FGF10/GNAS/FLVCR1/RSPO2/FMN1/AFF3/LEF1/PITX2/IFT122/SFRP2/BMP4/BMPR1B/SOX9/TWIST1/TP63/ALDH1A2	19
GO:0009611	response to wounding	76/901	965/17046	0.0003	0.0077	0.00606	CDH3/KCNMB2/MRVI1/CCR1/ADM/CTGF/NLRP6/A2M/F11/FGA/FGF10/VASH1/SBNO2/FOXC2/GATM/GNAS/GPER1/FFAR2/SCG3/GSTP1/GUCY1A3/ANXA2/SERPIND1/NRG1/ID3/IGF1/IGF2/CYR61/IL1R1/IL6/IRF1/ISL1/ITGB2/KCNMB1/LCK/LOX/SMAD3/MAP3K1/NOV/NRAS/ANGPT4/PIK3CG/PKM/PLA2G2A/IL20RB/TLR9/TREM1/APBB1IP/PRKAR1B/BIN3/MAPK3/MAP2K2/PROC/MASP1/PSMB4/TRPC7/NOD2/BMP4/SLC8A1/TIMP3/TNFAIP3/TNFRSF1A/TRPC6/CCR2/WNT10B/NLRX1/C6orf25/CAPZB/HIST1H3A/HOPX/ACTN1/TNFRSF11A/SYT7/ESAM/SLC16A3/ADIPOQ	76
GO:0048144	fibroblast proliferation	13/901	81/17046	0.00031	0.0078	0.00613	B4GALT7/EGFR/ESR1/FGF10/MORC3/GSTP1/ANXA2/IGF1/NRAS/SIRT6/PML/S100A6/SPHK1	13

GO:1902578	single-organism localization	242/901	3769/17046	0.00031	0.00798	0.00627	ABI1/CDH3/TSPAN5/KCNMB2/TCIRG1/TRDN/ABCA9/SPON2/COG5/ADCY3/TMED10/SLC27A2/RER1/CHGA/CHI3L1/PKP3/EXOC3/CHRNA1/CHRNA2/CHRNA5/CIDEA/PANX3/AP3S1/CLCA1/CCR1/SLC51B/C15orf27/SLC38A10/CNP/ADM/TRPM6/KLC3/CTGF/ABCC13/SH3D19/CYB561/CYLD/TRPV3/DDOST/BHLHA15/NLRP6/DLG2/ABAT/DRD4/AGXT/EGFR/A2M/UNC13D/SLC10A4/FCGR2A/FGA/FGF10/RASA3/TRAK1/EXPH5/NFASC/EPB41L3/FLNB/FLOT2/MLC1/NUP210/ATP11A/NEDD4L/SYNE1/MORC3/MAPK8IP2/MTOR/SLC37A4/SAMM50/STEAP2/SLC17A5/GJA3/GJB2/GLS2/VPS4A/GNAS/CRACR2B/PIGW/GPR26/GPER1/FFAR2/GRB10/FLVCR1/GRIK4/DNAJC15/SCG3/NME7/ANXA2/KCNIP2/NRG1/ANXA6/HK1/ANXA13/HLA-E/ACACB/HPCA/HSPA1L/HSP90AA1/HSP90AB1/HTR3A/IGF1/IGF2/IL1RN/IL6/AQP2/AQP5/INHBA/AQP9/ISL1/ITGB2/ITGB7/JUP/ATP9B/KCNH2/KCNJ8/KCNJ9/KCNMB1/IPO5/INSC/TOMM20L/SLC6A17/LCK/LDLR/LGALS9/LLGL1/LMNA/SMAD3/MFI2/ATP1A2/NFATC3/NOV/NTF3/OPRL1/SLC22A18/P2RY6/ATP5B/ANO7/PALM/PARK2/PDE4C/PCYOX1/C11orf73/SIRT6/ATP8A2/PIK3CG/PRKAG3/PML/FXYD6/SLCO1C1/PNLIP/TLR9/TREM1/POMC/PON1/ZDHHC13/GOLPH3L/SLC47A1/SLC29A3/TRPV6/SMPD3/SLC30A10/CHRNA9/SYBU/PEX26/PRKAR1B/IFT122/LMBRD1/PRKD1/APOBR/MAPK3/MAP2K2/TRPV5/CDC42SE1/TRPC7/RASGRF2/TRIM27/EXOC4/RPL8/RPL29/S100A6/SCT/NOD2/TINAGL1/STRA6/SFRP2/SGK1/VPS33A/BMP4/SLC4A1/SLC6A12/SLC8A1/SLC9A3/SLC20A2/SRP68/SUPT6H/VAMP2/TGM2/TRAPPC10/TNFRSF1A/TRPC4/TRPC6/TRPM2/TWIST1/CCR2/TNFRSF4/UCP1/YWHAG/CA7/CACNA1E/CACNB2/PAX8/FZD5/RAB7A/RAB11FIP1/CALR/COLQ/SLC25A18/MFSD7/ATP13A4/NR0B2/MON1A/CASQ1/MGARP/RAE1/SLC43A1/SCIN/IRS2/ACTN1/TNFRSF11A/SPHK1/MAP7/SYT7/SLC16A3/RSAD2/SMDT1/ADIPOQ/RAB3D/NUP93/RAPGEF2/USP6NL/FGF19/NR1H4	242
GO:0005977	glycogen metabolic process	12/901	71/17046	0.00032	0.00798	0.00628	CPS1/MTOR/GRB10/IGF1/IGF2/PRKAG3/POMC/PPP1CB/PPP1CC/PHLDA2/IRS2/STBD1	12
GO:0060438	trachea development	6/901	19/17046	0.00032	0.00807	0.00635	RSPO2/LEF1/MAPK3/MAP2K2/BMP4/SOX9	6
GO:0002694	regulation of leukocyte activation	38/901	399/17046	0.00033	0.00831	0.00654	MAP3K8/IL31RA/CYLD/EGR3/UNC13D/FGF10/FLOT2/MTOR/GPER1/HLA-DOA/HLA-DPA1/HLA-E/HLX/ZC3H12D/IGF1/IGF2/IL6/INHBA/IRF1/LCK/LGALS9/IL20RB/PAG1/PTPRE/NOD2/BMP4/BPI/SUPT6H/ZEB1/TNFAIP3/CCR2/TNFRSF4/ZAP70/LST1/ZC3H12A/SLA2/IRS2/FADD	38
GO:0035265	organ growth	17/901	126/17046	0.00034	0.00851	0.0067	COMP/ESR1/FGF10/FOXC2/TENM4/BMP10/FLVCR1/NRG1/HLX/ACACB/RSPO2/IGF1/NPPC/SIRT6/BNC2/SOX9/STK3	17
GO:0009259	ribonucleotide metabolic process	43/901	471/17046	0.00035	0.0086	0.00676	TCIRG1/NPFFR2/ADCY3/ACOT7/ADM/ADRB3/DLG2/DRD4/ENO2/GABBR1/GAPDHS/AMPD2/PDE7B/AMPD3/GNAS/GPER1/DNAJC15/GUCY1A3/NME7/HK1/ACACB/HPCA/NME9/IGF1/LHCGR/MC2R/MYH4/NDUFB4/ATP1A2/NPPC/OPRL1/ATP5B/PALM/PDE4C/PDE7A/SIRT6/PDE6B/PGAM2/PKM/MRAP/SCT/CCR2/UPP1	43
GO:0051046	regulation of secretion	54/901	633/17046	0.00036	0.00878	0.0069	CHGA/CIDEA/NLRP6/ABAT/DRD4/UNC13D/FGA/FGF10/EXPH5/VPS4A/GNAS/GPER1/FFAR2/NRG1/HLA-E/IGF1/IL1RN/IL6/INHBA/ISL1/LGALS9/LLGL1/NOV/OPRL1/PARK2/PDE4C/PML/TLR9/POMC/GOLPH3L/TRPV6/SMPD3/SYBU/PRKAR1B/TRIM27/SCT/NOD2/SGK1/VAMP2/TWIST1/CCR2/TNFRSF4/CACNA1E/PAX8/RAB7A/RAB11FIP1/NR0B2/SCIN/IRS2/TNFRSF11A/SYT7/RSAD2/ADIPOQ/RAB3D	54
GO:0050679	positive regulation of epithelial cell proliferation	19/901	150/17046	0.00036	0.00881	0.00693	CDH3/CDH13/EGFR/EGR3/FGF10/MTOR/NR4A1/IGF1/IL6/KDR/PRKD1/HTRA1/CCL11/NOD2/BMP4/SOX9/TNFAIP3/TWIST1/TP63	19
GO:0006073	cellular glucan metabolic process	12/901	72/17046	0.00036	0.00883	0.00694	CPS1/MTOR/GRB10/IGF1/IGF2/PRKAG3/POMC/PPP1CB/PPP1CC/PHLDA2/IRS2/STBD1	12
GO:0044042	glucan metabolic process	12/901	72/17046	0.00036	0.00883	0.00694	CPS1/MTOR/GRB10/IGF1/IGF2/PRKAG3/POMC/PPP1CB/PPP1CC/PHLDA2/IRS2/STBD1	12
GO:0071593	lymphocyte aggregation	38/901	401/17046	0.00037	0.00889	0.00699	MAP3K8/CYLD/DDOST/EGR3/FLOT2/MTOR/HLA-DOA/HLA-DPA1/HLA-E/HLX/ZC3H12D/IGF1/IGF2/IL6/IRF1/ITGB2/LCK/LCP1/LGALS9/SMAD3/NFATC3/LEF1/PIK3CG/IL20RB/APBB1IP/PAG1/NOD2/BMP4/STK10/ZEB1/CCR2/TNFRSF4/ZAP70/FZD5/SLA2/FADD/RSAD2/CD8A	38
GO:0043588	skin development	26/901	238/17046	0.00037	0.00889	0.00699	CDH3/APCDD1/CSTA/EGFR/FGF10/EXPH5/FLNB/LCE2B/GNAS/LCE1C/LCE1D/LCE2D/INHBA/IVL/JUP/LTB/LEF1/ATP8A2/SOX9/TCHH/WNT10B/RUNX1/TP63/RUNX3/LDB2/H2AFY	26
GO:0009150	purine ribonucleotide metabolic process	42/901	458/17046	0.00037	0.00889	0.00699	TCIRG1/NPFFR2/ADCY3/ACOT7/ADM/ADRB3/DLG2/DRD4/ENO2/GABBR1/GAPDHS/AMPD2/PDE7B/AMPD3/GNAS/GPER1/DNAJC15/GUCY1A3/NME7/HK1/ACACB/HPCA/NME9/IGF1/LHCGR/MC2R/MYH4/NDUFB4/ATP1A2/NPPC/OPRL1/ATP5B/PALM/PDE4C/PDE7A/SIRT6/PDE6B/PGAM2/PKM/MRAP/SCT/CCR2	42
GO:0055085	transmembrane transport	94/901	1258/17046	0.00037	0.00892	0.00701	KCNMB2/TCIRG1/TRDN/ABCA9/ADCY3/CHRNA1/CHRNA2/CHRNA5/PANX3/CLCA1/C15orf27/SLC38A10/TRPM6/ABCC13/CYB561/TRPV3/DRD4/SLC10A4/RASA3/NUP210/NEDD4L/MAPK8IP2/SLC37A4/SAMM50/SLC17A5/GJA3/GJB2/GNAS/FLVCR1/GRIK4/DNAJC15/KCNIP2/ANXA6/HK1/ACACB/HSP90AA1/HTR3A/AQP2/AQP5/AQP9/KCNH2/KCNJ8/KCNJ9/KCNMB1/SLC6A17/LDLR/ATP1A2/OPRL1/SLC22A18/ATP5B/ANO7/PCYOX1/PIK3CG/FXYD6/SLCO1C1/TLR9/SLC47A1/SLC29A3/TRPV6/SLC30A10/CHRNA9/PEX26/PRKAR1B/TRPV5/TRPC7/RASGRF2/TRIM27/S100A6/STRA6/SGK1/BMP4/SLC4A1/SLC6A12/SLC8A1/SLC9A3/SLC20A2/VAMP2/TRAPPC10/TRPC4/TRPC6/TRPM2/TWIST1/CACNA1E/CACNB2/SLC25A18/MFSD7/ATP13A4/CASQ1/RAE1/SLC43A1/IRS2/SLC16A3/SMDT1/NUP93	94

GO:0034113	heterotypic cell-cell adhesion	9/901	44/17046	0.00041	0.00985	0.00775	FGA/NFASC/IL1RN/ITGA7/ITGB2/ITGB7/JUP/PARVA/ADIPOQ	9
GO:0052652	cyclic purine nucleotide metabolic process	19/901	152/17046	0.00042	0.01009	0.00794	NPFFR2/ADCY3/ADM/ADRB3/DRD4/GABBR1/AMPD2/GNAS/GPER1/GUCY1A3/HPCA/LHCGR/MC2R/NPPC/OPRL1/PALM/MRAP/SCT/CCR2	19
GO:0009612	response to mechanical stimulus	22/901	189/17046	0.00043	0.01009	0.00794	CHI3L1/COL11A1/ANKRD23/IL6/IRF1/JUP/LCK/MAP3K1/ATP1A2/ATP8A2/CHRNA9/MAPK3/BGLAP/STRA6/BMP4/SLC8A1/SOX9/TIMP3/TLR5/TNFRSF1A/CRADD/FADD	22
GO:0006936	muscle contraction	29/901	280/17046	0.00043	0.01009	0.00794	MRVI1/TRDN/CHRNA1/CTGF/DTNA/BMP10/GPER1/TMOD4/GUCY1A3/KCNIP2/ANXA6/KCNH2/LCK/MYH4/MYL2/ATP1A2/ATP8A2/PGAM2/PIK3CG/PXN/ACTA2/SLC8A1/ACTC1/CALD1/CASQ1/TRIM63/SPHK1/STBD1/RCSD1	29
GO:0031325	positive regulation of cellular metabolic process	179/901	2688/17046	0.00044	0.01025	0.00806	ABI1/CDH3/CDH13/CDKN1C/DMRT2/TBR1/ADCY3/CHI3L1/ERLIN2/PSIP1/CCR1/SLC51B/MAP3K8/ADM/IL31RA/CTGF/SH3D19/ADRB3/CITED4/RNF168/BHLHA15/DRD4/ECE1/EEF2/EGFR/ESR1/FGA/FGF10/RASA3/SBNO2/FOXC2/FOXO1/LARP1/MAPK8IP2/VGLL2/MTOR/RNF144B/GAPDHS/PABPC1/DNAJC2/FGF22/NPTN/BMP10/GNAS/GPER1/DOK7/GRB10/GSTP1/BRF1/GUCY1A3/ANXA2/SOX8/NRG1/HK1/HMGA1/NR4A1/ACACB/HPCA/HRH1/HSP90AA1/HSP90AB1/TFAP2E/BARHL2/IGF1/IGF2/CYR61/IL1RN/IL6/FOXK2/INHBA/IRF1/ISL1/JUP/KDR/HES5/LCK/LDLR/LGALS9/LHCGR/LMNA/LMO2/LTB/SMAD3/MC2R/MEF2D/MAP3K1/MEOX1/MEOX2/MFI2/MITF/NFATC3/NFYB/NHLH2/NPPC/NRAS/NTF3/OPRL1/PARK2/LEF1/PRR16/ANGPT4/PIK3CG/PITX2/PLA2G2A/PLAGL1/PML/RIPK4/TLR9/CYTL1/POMC/BANP/PIWIL2/FANCI/PRKAR1B/PRKD1/MAPK3/MAP2K2/MRAP/PSMB4/PAK6/ARNTL2/RGMA/PSMD7/PXN/RASGRF2/TRIM27/SCT/CCL11/CCL17/NPAS3/NOD2/SFRP2/TRA2B/BMP4/ZNF649/BMPR1B/BOK/SOX9/STK3/STK10/SUPT6H/TBP/TCEA1/TCEB2/ZEB1/TEAD3/TLR5/TNFAIP3/TNFRSF1A/TRAF1/TRAF5/TWIST1/CCR2/WNT10B/PAX8/CXCR4/FZD5/CARD14/ZC3H12A/CALR/CDK10/RUNX1/TP63/RUNX3/IRS2/CRADD/FADD/TNFRSF11A/SPHK1/PIAS2/ZFAND2A/MAP3K6/LDB2/CBFA2T2/ADIPOQ/MICAL2/RAPGEF2/FGF19/NR1H4	179
GO:0051240	positive regulation of multicellular organismal process	96/901	1297/17046	0.00044	0.0104	0.00818	TRDN/SPON2/DMRT2/CELF1/CHI3L1/CCR1/SEZ6/ADM/CPS1/CTGF/DIO3/ABAT/DRD4/EGR3/EIF4G1/EPHA1/EPHA3/FGA/FGF10/ACIN1/FOXC2/NEDD4L/PUM2/TENM4/NPTN/BMP10/GNAS/GPER1/FFAR2/SOX8/NRG1/HLA-DPA1/HLA-E/HLX/ACACB/HOXD3/HRH1/IGF1/CYR61/IL6/IL12RB2/INHBA/IRF1/ISL1/KDR/AMIGO3/LCK/ARHGDIA/LGALS9/LTB/SMAD3/NEU1/NPPC/OPRL1/PALM/PARK2/LEF1/ANGPT4/ATP8A2/PML/IL20RB/TLR9/PRKD1/MAP2K2/SCT/CCL11/NOD2/SFRP2/BMP4/SLC8A1/BMPR1B/SOX9/ZEB1/TEAD3/TLR5/TNFAIP3/TWIST1/CCR2/WNT10B/ZAP70/PAX8/CXCR4/FZD5/NLRX1/IFITM1/SCIN/RUNX1/TP63/FADD/TNFRSF11A/SPHK1/CBFA2T2/RSAD2/ADIPOQ/H2AFY/RAPGEF2	96
GO:0071605	monocyte chemotactic protein-1 production	4/901	8/17046	0.00046	0.0106	0.00834	GSTP1/LGALS9/TWIST1/ADIPOQ	4
GO:0071637	regulation of monocyte chemotactic protein-1 production	4/901	8/17046	0.00046	0.0106	0.00834	GSTP1/LGALS9/TWIST1/ADIPOQ	4
GO:0072282	metanephric nephron tubule morphogenesis	4/901	8/17046	0.00046	0.0106	0.00834	SOX8/HES5/SOX9/PAX8	4
GO:0009190	cyclic nucleotide biosynthetic process	19/901	153/17046	0.00046	0.01063	0.00836	NPFFR2/ADCY3/ADM/ADRB3/DRD4/GABBR1/AMPD2/GNAS/GPER1/GUCY1A3/HPCA/LHCGR/MC2R/NPPC/OPRL1/PALM/MRAP/SCT/CCR2	19
GO:0048146	positive regulation of fibroblast proliferation	10/901	54/17046	0.00046	0.01065	0.00837	EGFR/ESR1/FGF10/ANXA2/IGF1/NRAS/SIRT6/PML/S100A6/SPHK1	10

GO:0044271	cellular nitrogen compound biosynthetic process	274/901	4363/17046	0.00047	0.01065	0.00837	ZNF783/CDH13/CDKN1C/TCIRG1/C1D/ZBTB18/MTHFS/DMRT2/CELF1/TBR1/NPFFR2/ADCY3/PNRC1/HNRNPUL1/ERLIN2/PSIP1/EGLN2/ACOT7/ZBED9/CIDEA/MRPL52/ZFP42/ADM/IL31RA/CPS1/ZNF358/ZNF738/CTGF/SMYD1/CYLD/ADRB3/ZNF782/ADAL/ZNF709/ZNF781/CITED4/DDB1/DDOST/RNF168/ZNF366/BHLHA15/DNMT3A/DRD4/EEF2/EGFR/EGR3/PATL2/EIF4G1/ELK4/ESR1/ALAS1/SP8/FGF10/FHIT/XRN2/SBNO2/TRAK1/MSRB2/FOXL1/FOXC2/FOXO1/NUP210/NEDD4L/LARP1/PUM2/RYBP/VGLL2/MTOR/GABBR1/ZNF549/ST6GALNAC3/PABPC1/DNAJC2/AMPD2/DKK3/GLS2/AMPD3/BMP10/ZNF638/GNAS/ZNF311/ZNF844/GPER1/MRPS18B/ZBTB44/GTF2B/BRF1/GUCY1A3/NME7/SOX8/NRG1/HLX/HMGA1/NR4A1/ACACB/HPCA/HOXB3/HOXC4/HOXC5/HOXC6/HOXD3/HSP90AA1/HSP90AB1/TFAP2E/ID3/BARHL2/NME9/IGF1/IGF2/CYR61/IL6/IL16/FOXK2/INHBA/IRF1/ISL1/JUP/USP50/HILS1/HES5/AFF3/LGALS9/LHCGR/LMO2/SMAD3/MC2R/ME1/MEF2D/MEOX1/MEOX2/MITF/LHX8/MOV10/DRG1/NFATC3/NFYB/NHLH2/NPPC/NTF3/OAS2/OPRL1/ATP5B/PALM/PARK2/LEF1/PRR16/SIRT6/PITX2/PKHD1/PKM/PLAGL1/PRKAG3/PML/RIPPLY3/RIPK4/TLR9/CYTL1/POMC/POU2AF1/BNC2/MED18/BANP/PIWIL2/ELP3/PRMT6/DNAJC17/ZNF532/CNOT11/PRKD1/MYNN/MAPK3/MRAP/PRMT8/PAK6/ARNTL2/RGMA/PRDM11/TENM2/GATAD2B/METTL14/CCAR2/CREBZF/RFC2/TRIM27/RGS12/RPA3/RPL8/RPL29/SCT/MRPS14/NPAS3/NOD2/SFRP2/GZF1/SGK1/BMP4/ZNF649/BMPR1B/BRD9/ZSCAN18/SOX9/SRP68/STAT2/STK3/SUPT6H/TAF4B/TBP/TCEA1/TCEB2/ZEB1/TEAD3/TERF1/TLE3/TLR5/TNFAIP3/TNFRSF1A/TRAF1/TRAF5/TWIST1/CCR2/TNFRSF4/UCP1/UPP1/VARS/WNT10B/YWHAG/ZNF7/ZNF124/ZNF177/PAX8/FZD5/CARD14/CERS4/ZNF665/ZNF606/ZC3H12A/CPEB4/ZNF436/CALR/QTRT1/SLIRP/SCRT1/HIST1H3A/SLA2/ZNF397/NR0B2/HOPX/KDM2B/LOXL3/CBX2/RAE1/GAS7/KMO/RUNX1/TP63/RUNX3/ACTN1/FADD/TNFRSF11A/SPHK1/BUD31/CCNA1/LIMD1/PIAS2/LDB2/CBFA2T2/AURKB/ADIPOQ/H2AFY/MICAL2/VGLL4/NUP93/ZBTB39/NR1H4	274
GO:0008544	epidermis development	30/901	295/17046	0.00047	0.01065	0.00837	CDH3/APCDD1/CSTA/CTGF/EGFR/FGF10/EXPH5/FLNB/FLOT2/LCE2B/GNAS/LCE1C/LCE1D/LCE2D/INHBA/IVL/KRT15/HES5/LAMA3/NTF3/PITX2/BMP4/SOX9/TCHH/WNT10B/RUNX1/TP63/RUNX3/LDB2/H2AFY	30
GO:0055080	cation homeostasis	49/901	566/17046	0.00047	0.01076	0.00846	TCIRG1/TRDN/CLN5/CCR1/ADM/DRD4/ESR1/NEDD4L/STEAP2/NPTN/GPER1/FLVCR1/ANXA6/KCNH2/KDR/LCK/MFI2/NUBP1/ATP1A2/OPRL1/ATP5B/PARK2/PDE6B/PIK3CG/PKHD1/PML/SLC30A10/CHRNA9/PRKD1/SLAMF8/TRPC7/CCL11/SGK1/BMP4/SLC4A1/SLC8A1/SLC9A3/TGM2/TRPC4/TRPC6/CCR2/CA7/CXCR4/RAB7A/CALR/ATP13A4/CASQ1/SMDT1/MTL5	49
GO:0034330	cell junction organization	26/901	242/17046	0.00048	0.01079	0.00849	CDH3/CDH9/CDH12/CDH13/PKP3/MPP7/EPHA3/NFASC/EPB41L3/GJB2/FMN1/JUP/KDR/LAMA3/SMAD3/MPZ/PLEC/FBLIM1/LIMS2/PARVA/PXN/FZD5/PARD6G/PARD6B/ACTN1/RAPGEF2	26
GO:0051145	smooth muscle cell differentiation	9/901	45/17046	0.00049	0.01108	0.00871	ADM/FGF10/NFATC3/NTF3/PITX2/BMP4/SOX9/ZEB1/TMEM204	9
GO:0006468	protein phosphorylation	118/901	1662/17046	0.0005	0.01115	0.00877	AKT3/ABI1/CDKN1C/SPEG/BCKDK/HCST/NPFFR2/ADCY3/CHI3L1/ALPK2/CCR1/MAP3K8/IL31RA/TRPM6/CTGF/PPM1L/ADRB3/NLRP6/DRD4/EGFR/ADCK5/EPHA1/EPHA3/EPHB4/FGA/FGF10/RASA3/PPM1E/FOXO1/MORC3/MAPK8IP2/TSSK2/MTOR/GAK/RPS6KC1/FGF22/NPTN/BMP10/GPER1/DOK7/GSTP1/ANXA2/NRG1/HSP90AB1/IGF1/IGF2/CYR61/IL1RN/IL6/IL12RB2/INHBA/ISL1/ITGB2/KCNH2/KDR/HES5/LCK/LGALS9/SMAD3/MAP3K1/NRAS/NTF3/OPRL1/PARK2/ANGPT4/PIK3CG/PKHD1/PLA2G2A/PRKAG3/PML/RIPK4/TLR9/ELP3/PRKAR1B/PRKD1/MAPK3/MAP2K2/PSMB4/PAK6/PSMD7/MARK4/PXN/RASGRF2/TRIM27/CCL11/CCL17/NOD2/SFRP2/SGK1/BMP4/BMPR1B/SOX9/STK3/STK10/TNFAIP3/TNFRSF1A/TNXB/TWIST1/TNFRSF4/YWHAG/ZAP70/CXCR4/FZD5/CARD14/TTBK1/CDK10/RUNX3/IRS2/TNFRSF11A/STK19/SPHK1/MAP3K6/AURKB/ADIPOQ/H2AFY/RAPGEF2/ULK2/FGF19	118
GO:0070374	positive regulation of ERK1 and ERK2 cascade	19/901	154/17046	0.0005	0.01119	0.0088	CHI3L1/CCR1/CTGF/EGFR/FGA/FGF10/GPER1/IL6/KDR/LGALS9/PLA2G2A/MAPK3/CCL11/CCL17/NOD2/BMP4/TNFRSF11A/RAPGEF2/FGF19	19
GO:0060326	cell chemotaxis	24/901	217/17046	0.00051	0.01135	0.00892	CHGA/CCR1/EGR3/FFAR2/NR4A1/HRH1/IL6/IL16/ITGB2/LGALS9/NOV/LEF1/PIK3CG/TREM1/PARVA/PRKD1/PLEKHG5/CCL11/CCL17/NOD2/CCR2/CXCR4/CALR/TNFRSF11A	24
GO:0048732	gland development	38/901	408/17046	0.00051	0.01135	0.00892	CDKN1C/CPS1/EGFR/ESR1/FGA/FGF10/DKK3/NRG1/HLX/HOXB3/HOXD3/IGF1/IGF2/IL6/ISL1/ACAT1/SMAD3/NFATC3/LEF1/PITX2/PKM/PML/LIMS2/CCL11/STRA6/BMP4/SOX9/TGM2/TNFAIP3/PAX8/PPDPF/CAST/RUNX1/TP63/IRS2/FADD/TNFRSF11A/ALDH1A2	38
GO:0070486	leukocyte aggregation	38/901	408/17046	0.00051	0.01135	0.00892	MAP3K8/CYLD/DDOST/EGR3/FLOT2/MTOR/HLA-DOA/HLA-DPA1/HLA-E/HLX/ZC3H12D/IGF1/IGF2/IL6/IRF1/ITGB2/LCK/LCP1/LGALS9/SMAD3/NFATC3/LEF1/PIK3CG/IL20RB/APBB1IP/PAG1/NOD2/BMP4/STK10/ZEB1/CCR2/TNFRSF4/ZAP70/FZD5/SLA2/FADD/RSAD2/CD8A	38
GO:1902533	positive regulation of intracellular signal transduction	72/901	921/17046	0.00053	0.01178	0.00927	CDH13/HCST/CHI3L1/CCR1/MAP3K8/IL31RA/MIB2/CTGF/ADRB3/DRD4/EGFR/FGA/FGF10/RASA3/AKR1B1/PUM2/MAPK8IP2/MTOR/FGF22/GNAS/GPER1/NRG1/IGF1/IGF2/IL1RN/IL6/ISL1/KDR/HES5/LCK/LGALS9/LHCGR/MAP3K1/NRAS/NTF3/PARK2/PIK3CG/PLA2G2A/TLR9/ZDHHC13/PRKD1/MAPK3/MAP2K2/PSMB4/PSMD7/PLEKHG5/PXN/RASGRF2/S100A4/CCL11/CCL17/NOD2/BMP4/SOX9/STK3/BST2/TNFRSF1A/TRAF5/ZAP70/CXCR4/FZD5/CARD14/CDK10/IRS2/FADD/TNFRSF11A/SPHK1/MAP3K6/CD8A/ADIPOQ/RAPGEF2/FGF19	72
GO:0002040	sprouting angiogenesis	10/901	55/17046	0.00054	0.0119	0.00935	CDH13/ESM1/EGR3/EPHB4/FOXC2/NR4A1/KDR/LEF1/PARVA/BMP4	10
GO:0021781	glial cell fate commitment	5/901	14/17046	0.00055	0.01199	0.00943	SOX8/NRG1/HES5/NTF3/SOX9	5
GO:0044262	cellular carbohydrate metabolic process	28/901	271/17046	0.00055	0.01207	0.00949	GNE/SLC51B/CPS1/FOXO1/AKR1B1/MTOR/GAPDHS/GPER1/GRB10/HAS1/HK1/HRH1/IGF1/IGF2/IL6/LHCGR/PARK2/SIRT6/PGAM2/PRKAG3/POMC/PPP1CB/PPP1CC/CSGALNACT1/PHLDA2/IRS2/STBD1/ADIPOQ	28

GO:0019693	ribose phosphate metabolic process	43/901	482/17046	0.00056	0.0122	0.00959	TCIRG1/NPFFR2/ADCY3/ACOT7/ADM/ADRB3/DLG2/DRD4/ENO2/GABBR1/GAPDHS/AMPD2/PDE7B/AMPD3/GNAS/GPER1/DNAJC15/GUCY1A3/NME7/HK1/ACACB/HPCA/NME9/IGF1/LHCGR/MC2R/MYH4/NDUFB4/ATP1A2/NPPC/OPRL1/ATP5B/PALM/PDE4C/PDE7A/SIRT6/PDE6B/PGAM2/PKM/MRAP/SCT/CCR2/UPP1	43
GO:0045595	regulation of cell differentiation	99/901	1356/17046	0.00058	0.01248	0.00982	TBR1/CCR1/SEZ6/ADM/FAM101A/CTGF/SMYD1/CYLD/BHLHA15/DMBT1/EGR3/EIF4G1/UNC13D/EPHA3/FGA/FGF10/ACIN1/FOXC2/FOXO1/SPG20/FLOT2/NEDD4L/MTOR/TENM4/NPTN/BMP10/GNAS/GPER1/SOX8/NRG1/HLA-B/HLA-DOA/HLX/HOXB3/HOXD3/ID3/BARHL2/IGF1/CYR61/IL6/INHBA/IRF1/ISL1/JUP/KDR/HES5/LCK/ARHGDIA/LGALS9/SMAD3/MFI2/MITF/NEU1/NOV/NPPC/NTF3/PALM/PARK2/LEF1/ATP8A2/PLA2G2A/PML/SSH1/PRKD1/MAP2K2/BGLAP/CCL17/SFRP2/BMP4/BMPR1B/SOX9/STK3/SUPT6H/ZEB1/TEAD3/TNFRSF1A/TWIST1/WNT10B/YWHAG/ZAP70/PAX8/CXCR4/ZC3H12A/CALR/SCRT1/HOPX/IFITM1/SCIN/RUNX1/TP63/RUNX3/FADD/LIMD1/PIAS2/CBFA2T2/ADIPOQ/H2AFY/RAPGEF2/ULK2	99
GO:0031076	embryonic camera-type eye development	8/901	37/17046	0.00058	0.01251	0.00984	FGF10/PITX2/STRA6/ZEB1/TWIST1/FZD5/KDM2B/ALDH1A2	8
GO:0002407	dendritic cell chemotaxis	6/901	21/17046	0.00059	0.01259	0.0099	CCR1/LGALS9/PIK3CG/CCR2/CXCR4/CALR	6
GO:0006109	regulation of carbohydrate metabolic process	19/901	156/17046	0.00059	0.01259	0.0099	SLC51B/FOXO1/MTOR/GAPDHS/GPER1/GRB10/HRH1/IGF1/IGF2/IL6/LHCGR/PARK2/SIRT6/PGAM2/POMC/PPP1CB/PHLDA2/IRS2/ADIPOQ	19
GO:2000147	positive regulation of cell motility	33/901	341/17046	0.0006	0.0129	0.01015	CDH13/CCR1/EGFR/EPHA1/FGF10/FOXC2/GPER1/IGF1/CYR61/IL6/KDR/LGALS9/SMAD3/NTF3/P2RY6/LEF1/ANGPT4/ELP3/PRKD1/MAP2K2/CCL11/BMP4/SLC8A1/SOX9/TWIST1/CCR2/PTP4A1/COL18A1/CALR/IRS2/FADD/SPHK1/RAPGEF2	33
GO:0007274	neuromuscular synaptic transmission	7/901	29/17046	0.00063	0.01343	0.01056	CHRNA1/CHRNA2/CHRNA5/DTNA/EGR3/NTF3/CHRNA9	7
GO:0030802	regulation of cyclic nucleotide biosynthetic process	17/901	133/17046	0.00065	0.01365	0.01074	NPFFR2/ADM/ADRB3/DRD4/GABBR1/GNAS/GPER1/GUCY1A3/HPCA/LHCGR/MC2R/NPPC/OPRL1/PALM/MRAP/SCT/CCR2	17
GO:0051093	negative regulation of developmental process	58/901	710/17046	0.00065	0.01365	0.01074	CDH3/LECT1/CCR1/SEZ6/FAM101A/CYLD/ADRB3/BHLHA15/FGF10/VASH1/FOXC2/FOXO1/SPG20/GNAS/GPER1/SOX8/HLX/ID3/RSPO2/IGF1/IL6/INHBA/IRF1/ISL1/HES5/ARHGDIA/SMAD3/MFI2/NOV/NPPC/NRAS/PARK2/LEF1/CEND1/ANGPT4/PML/LIMS2/IFT122/CCL17/SFRP2/BMP4/SLIT1/SOX9/STK3/ZEB1/TWIST1/CCR2/WNT10B/PAX8/CALR/HOPX/RUNX1/TP63/LIMD1/CBFA2T2/ADIPOQ/RAPGEF2/ULK2	58
GO:0070838	divalent metal ion transport	37/901	399/17046	0.00065	0.01375	0.01081	TRDN/CLCA1/CCR1/C15orf27/TRPM6/CTGF/TRPV3/BHLHA15/DRD4/RASA3/CRACR2B/GPER1/ANXA6/LCK/ATP1A2/NFATC3/OPRL1/PIK3CG/PML/TLR9/ZDHHC13/TRPV6/SLC30A10/CHRNA9/PRKD1/TRPV5/TRPC7/TRIM27/BMP4/SLC8A1/TRPC4/TRPC6/TRPM2/CACNA1E/CACNB2/CASQ1/SMDT1	37
GO:0010628	positive regulation of gene expression	114/901	1608/17046	0.00066	0.01375	0.01081	CDH3/CDH13/CDKN1C/DMRT2/TBR1/ERLIN2/PSIP1/IL31RA/CTGF/CITED4/BHLHA15/EEF2/EGFR/ESR1/FGF10/SBNO2/FOXC2/FOXO1/LARP1/VGLL2/MTOR/PABPC1/DNAJC2/BMP10/GPER1/BRF1/SOX8/HMGA1/NR4A1/HOXD3/TFAP2E/BARHL2/IGF1/IGF2/CYR61/IL6/FOXK2/INHBA/IRF1/ISL1/JUP/HES5/LCK/LGALS9/LMNA/LMO2/SMAD3/MEF2D/MEOX1/MEOX2/MFI2/MITF/NFATC3/NFYB/NHLH2/NTF3/PARK2/LEF1/PRR16/PITX2/PLAGL1/RIPK4/TLR9/CYTL1/POMC/BANP/PIWIL2/PRKD1/MAPK3/ARNTL2/RGMA/PTGFR/ACTA2/TRIM27/NPAS3/NOD2/SFRP2/TRA2B/BMP4/ZNF649/BMPR1B/SOX9/STK3/SUPT6H/TBP/TCEA1/TCEB2/ZEB1/ACTC1/TEAD3/TNFRSF1A/TRAF1/TRAF5/TWIST1/WNT10B/PAX8/FZD5/CARD14/ZC3H12A/CALR/NR0B2/RUNX1/TP63/RUNX3/FADD/TNFRSF11A/ALDH1A2/SPHK1/PIAS2/LDB2/CBFA2T2/H2AFY/MICAL2/NR1H4	114
GO:0030097	hemopoiesis	56/901	680/17046	0.00066	0.01375	0.01081	ABI1/CDKN1C/CCR1/IL31RA/CYLD/ESCO2/EEF2/EFNA2/EGR3/EML1/SBNO2/ACIN1/MTOR/GNAS/FLVCR1/ANXA2/HLA-B/HLA-DOA/HLX/HOXB3/IL6/INHBA/IRF1/KDR/HES5/LCK/LGALS9/LMO2/MEOX1/MITF/NFATC3/LEF1/PML/HERC6/SMPD3/BGLAP/SFRP2/VPS33A/BMP4/STK3/TCEA1/ZEB1/WNT10B/ZAP70/FZD5/C6orf25/SCIN/RUNX1/RUNX3/ACTN1/FADD/TNFRSF11A/RSAD2/CD8A/ADIPOQ/CD79A	56
GO:0009165	nucleotide biosynthetic process	31/901	315/17046	0.00066	0.01375	0.01081	TCIRG1/NPFFR2/ADCY3/ACOT7/ADM/ADRB3/DRD4/GABBR1/AMPD2/AMPD3/GNAS/GPER1/GUCY1A3/NME7/ACACB/HPCA/NME9/LHCGR/MC2R/ME1/NPPC/OAS2/OPRL1/ATP5B/PALM/PKM/MRAP/SCT/CCR2/UPP1/KMO	31
GO:0030003	cellular cation homeostasis	43/901	486/17046	0.00066	0.01375	0.01081	TCIRG1/TRDN/CLN5/CCR1/ADM/DRD4/ESR1/NEDD4L/NPTN/GPER1/FLVCR1/ANXA6/LCK/NUBP1/ATP1A2/OPRL1/ATP5B/PDE6B/PIK3CG/PKHD1/PML/SLC30A10/CHRNA9/PRKD1/SLAMF8/TRPC7/CCL11/SGK1/BMP4/SLC4A1/SLC8A1/TGM2/TRPC4/TRPC6/CCR2/CA7/CXCR4/RAB7A/CALR/ATP13A4/CASQ1/SMDT1/MTL5	43

GO:0006357	regulation of transcription from RNA polymerase II promoter	118/901	1675/17046	0.00066	0.01375	0.01081	ZNF783/CDH13/CDKN1C/ZBTB18/DMRT2/TBR1/ERLIN2/PSIP1/EGLN2/RNF168/ZNF366/BHLHA15/DNMT3A/EGFR/ELK4/ESR1/FGF10/SBNO2/TRAK1/FOXL1/FOXC2/FOXO1/SPG20/NEDD4L/RYBP/VGLL2/BMP10/GPER1/SOX8/HMGA1/NR4A1/HOXB3/HOXC5/HOXC6/TFAP2E/ID3/BARHL2/IGF1/IGF2/CYR61/IL6/FOXK2/INHBA/IRF1/ISL1/HES5/LMNA/LMO2/SMAD3/MEF2D/MEOX1/MEOX2/MITF/NFATC3/NHLH2/NTF3/PARK2/LEF1/PITX2/PLAGL1/RIPPLY3/TLR9/CYTL1/POMC/MED18/ELP3/PRMT6/DNAJC17/PRKD1/MAPK3/HTRA1/ARNTL2/RGMA/TENM2/GATAD2B/TRIM27/NPAS3/NOD2/SFRP2/GZF1/BMP4/ZNF649/BMPR1B/ZSCAN18/SOX9/STAT2/SUPT6H/TCEA1/TCEB2/ZEB1/TEAD3/TNFRSF1A/TWIST1/UCP1/WNT10B/ZNF177/PAX8/FZD5/CALR/SCRT1/SLA2/NR0B2/HOPX/KDM2B/CBX2/RUNX1/TP63/RUNX3/FADD/BUD31/CCNA1/PIAS2/LDB2/CBFA2T2/AURKB/H2AFY/MICAL2/NR1H4	118
GO:0040017	positive regulation of locomotion	34/901	357/17046	0.00067	0.01379	0.01084	CDH13/CCR1/EGFR/EPHA1/FGF10/FOXC2/GPER1/IGF1/CYR61/IL6/IL16/KDR/LGALS9/SMAD3/NTF3/P2RY6/LEF1/ANGPT4/ELP3/PRKD1/MAP2K2/CCL11/BMP4/SLC8A1/SOX9/TWIST1/CCR2/PTP4A1/COL18A1/CALR/IRS2/FADD/SPHK1/RAPGEF2	34
GO:0044238	primary metabolic process	540/901	9328/17046	0.00067	0.01379	0.01084	AKT3/ABI1/GNE/ZNF783/TSPAN5/CDH13/SUGP2/MBNL2/FARP1/CDKN1C/SPEG/BCKDK/TCIRG1/C1D/ZBTB18/PITRM1/MTHFS/PDPN/DMRT2/CELF1/CELF2/TBR1/HCST/NPFFR2/ADCY3/PNRC1/TMED10/SLC27A2/LECT1/ADAM29/HNRNPUL1/RPP14/HIBADH/CHI3L1/ERLIN2/PSIP1/CHI3L2/EGLN2/B4GALT7/ACOT7/EXOC3/ADPRHL1/CARD16/ZBED9/CIDEA/ALPK2/GALNT15/CLCA1/CLN5/MRPL52/CCR1/SLC51B/CNP/APOA1BP/NEU4/COL11A1/GALM/MAP3K8/ZFP42/ADM/IL31RA/EGFLAM/UBLCP1/HUS1B/CPD/CPM/CPS1/PXDNL/CRABP1/ZNF358/TRPM6/MIB2/PARP4/LDLRAD3/B3GLCT/MGAT5B/CSTA/ZNF738/CTGF/SMYD1/PPM1L/SH3D19/CYLD/MBOAT1/ADRB3/ESCO2/CYP11A1/ZNF782/FITM1/ADAL/ZNF709/ZNF781/CITED4/DDB1/LONRF2/DDOST/RNF168/ZNF366/BHLHA15/NLRP6/DLG2/DNMT3A/ABAT/DPH1/DRD4/ECE1/AGXT/EEF2/EGFR/EGR3/PATL2/EIF4G1/A2M/ELK4/LIPH/ENO2/ADCK5/EPHA1/EPHA3/EPHB4/ESR1/ALAS1/F11/FAH/PRSS54/RNF182/SP8/FGA/FGF10/FHIT/XRN2/RASA3/PPM1E/SBNO2/TRAK1/MSRB2/ACIN1/FOXL1/FOXC2/FOXO1/AKR1B1/GGA3/FLOT2/NUP210/NEDD4L/LARP1/PUM2/RYBP/MORC3/MAPK8IP2/TSSK2/VGLL2/MTOR/FUCA1/SLC37A4/GABBR1/RNF144B/ZNF549/ST6GALNAC3/GAK/SAMM50/PNKD/TENM4/ACOT11/LTN1/FBXL21/FBXO2/LCE2B/SACS/GATM/GBGT1/GAPDHS/SLC17A5/RPS6KC1/PABPC1/DNAJC2/FGF22/NPTN/AMPD2/PDE7B/DKK3/GLS2/VPS4A/AMPD3/DHDH/BMP10/ZNF638/GNAS/ZNF311/TMPRSS12/PIGW/ZNF844/THEM5/GPER1/EOGT/DOK7/GRB10/MRPS18B/ZBTB44/DNAJC15/GSTP1/GTF2B/BRF1/GUCY1A3/NME7/PADI1/GZMA/ANXA2/HAS1/SERPIND1/SOX8/NRG1/HK1/HLX/HMGA1/NR4A1/ACACB/HPCA/HOXB3/HOXC4/HOXC5/HOXC6/HOXD3/HRH1/HSD11B1/HSD17B2/ACADL/HSPA1L/HSP90AA1/HSP90AB1/DUPD1/ADAMTSL5/TFAP2E/ID3/ZC3H12D/COL28A1/BARHL2/NME9/IGF1/IGF2/CYR61/LCE1C/LCE1D/LCE2D/IL1RN/IL6/IL12RB2/PRSS41/IL16/FOXK2/INHBA/INPP5A/IRF1/ISL1/ITGB2/ITIH3/ITIH4/IVL/JUP/USP50/HILS1/KCNH2/KDR/ACAT1/HES5/AFF3/LCK/MUC21/LDLR/LGALS9/LHCGR/LMNA/LMO2/LOX/LTB/SMAD3/MC2R/ME1/ME2/MEF2D/MAP3K1/MEOX1/MEOX2/MFI2/MGAT1/MITF/LHX8/MOCS1/MOV10/NUDT1/MYH4/NDUFB4/DRG1/NEU1/ATP1A2/NFATC3/NFYB/NHLH2/NPPC/NRAS/NTF3/OAS2/OPRL1/PAFAH2/ATP5B/PALM/PARK2/SPOCK3/UTP11L/LEF1/DDX47/PRR16/CHST15/ANGPT4/PDE4C/PCYOX1/PDE7A/SIRT6/PDE6B/GALNT7/PGAM2/PI3/PIGC/PIK3CG/PITX2/PKHD1/PKM/PLA2G2A/PLAGL1/PRKAG3/PML/RIPPLY3/SLCO1C1/PNLIP/RIPK4/TLR9/TREM1/CYTL1/POMC/SSH1/PON1/MOV10L1/POU2AF1/BNC2/MED18/RPP25/LPCAT2/BANP/PPP1CB/HERC6/PPP1CC/PIWIL2/ELP3/PRMT6/DNAJC17/ZNF532/PPP2R2B/FANCI/WDR33/SMPD3/CNOT11/VAC14/PRKAR1B/CSGALNACT1/PRKD1/WSB2/MYNN/APOBR/MAPK3/MAP2K2/PROC/MRAP/PRMT8/MASP1/HTRA1/PSMB4/PAK6/ARNTL2/RGMA/PRDM11/PSMD7/TENM2/GATAD2B/KLHL8/RDH14/METTL14/MARK4/CCAR2/PTPRE/PXN/CREBZF/RASGRF2/RFC2/TRIM27/RGR/RGS12/EXOC4/RPA3/RPL8/RPL29/SCT/CCL11/CCL17/ABHD4/MRPS14/PRSS22/NPAS3/NOD2/STRA6/SFRP2/TRA2B/GZF1/SGK1/CERK/BMP4/ZNF649/BMPR1B/BRD9/ZSCAN18/BOK/SOX9/SRP68/STAT2/STK3/STK10/SUPT6H/BST2/VAMP2/TAF4B/TBP/TCEA1/TCEB2/ZEB1/TEAD3/TERF1/TGM2/TIMP3/TLE3/TNFAIP3/TNFRSF1A/TNXB/TRAF1/TRAF5/PHLDA2/TWIST1/CCR2/TNFRSF4/UCP1/UPP1/VARS/WNT10B/YWHAG/ZAP70/ZNF7/ZNF124/ZNF177/PTP4A1/MOGS/PAX8/CXCR4/FZD5/RAB7A/ERI3/CARD14/GDPD3/CERS4/ZNF665/EPHX3/ZC3H14/ERMP1/ZNF606/ZC3H12A/FAAP100/CPEB4/ZNF436/EEPD1/CALR/QTRT1/SLIRP/CAST/SCRT1/HIST1H3A/SLA2/ZNF397/NR0B2/HOPX/TTBK1/SPINK7/TRIM63/KDM2B/LOXL3/GTPBP3/CBX2/RAE1/GAS7/CDK10/KMO/RUNX1/TP63/RUNX3/SERPINA6/IRS2/ACTN1/CRADD/FADD/TNFRSF11A/ALDH1A2/STK19/SYNJ2/SPHK1/BUD31/CCNA1/ENDOU/STBD1/LIMD1/CH25H/ERI1/PIAS2/ZFAND2A/MAP3K6/LDB2/CBFA2T2/AURKB/NEURL3/SDR42E1/ADIPOQ/RAB3D/H2AFY/ENTPD3/PREPL/MICAL2/N4BP1/VGLL4/NUP93/RAPGEF2/ULK2/ZBTB39/LPGAT1/FGF19/NR1H4	540
GO:0001934	positive regulation of protein phosphorylation	71/901	913/17046	0.00067	0.01379	0.01084	ABI1/ADCY3/CHI3L1/CCR1/MAP3K8/IL31RA/CTGF/ADRB3/DRD4/EGFR/FGA/FGF10/RASA3/MAPK8IP2/MTOR/FGF22/NPTN/BMP10/GPER1/DOK7/ANXA2/NRG1/HSP90AB1/IGF1/IGF2/CYR61/IL1RN/IL6/INHBA/ISL1/KDR/HES5/LCK/LGALS9/MAP3K1/NRAS/NTF3/OPRL1/ANGPT4/PIK3CG/PLA2G2A/TLR9/PRKAR1B/PRKD1/MAPK3/MAP2K2/PSMB4/PAK6/PSMD7/PXN/RASGRF2/CCL11/CCL17/NOD2/SFRP2/BMP4/SOX9/STK3/STK10/TNFRSF1A/CXCR4/FZD5/CARD14/CDK10/IRS2/TNFRSF11A/SPHK1/MAP3K6/ADIPOQ/RAPGEF2/FGF19	71
GO:0051049	regulation of transport	115/901	1626/17046	0.00068	0.01379	0.01084	CDH3/CDH13/TRDN/CHGA/CIDEA/CCR1/SLC51B/CYLD/TRPV3/NLRP6/ABAT/DRD4/EGFR/UNC13D/FGA/FGF10/EXPH5/MLC1/NUP210/NEDD4L/MAPK8IP2/MTOR/GLS2/VPS4A/GNAS/CRACR2B/GPR26/GPER1/FFAR2/GRB10/ANXA2/KCNIP2/NRG1/ANXA13/HLA-E/HPCA/HSPA1L/HSP90AB1/IGF1/IL1RN/IL6/INHBA/ISL1/JUP/KCNH2/KCNJ8/KCNJ9/IPO5/LCP1/LGALS9/LLGL1/SMAD3/ATP1A2/NFATC3/NOV/NTF3/OPRL1/PARK2/PDE4C/SIRT6/ATP8A2/PIK3CG/PML/FXYD6/TLR9/POMC/PON1/GOLPH3L/TRPV6/SMPD3/SYBU/PRKAR1B/LMBRD1/PRKD1/MAPK3/MAP2K2/RASGRF2/TRIM27/SCT/NOD2/SFRP2/SGK1/BMP4/SLC8A1/SLC9A3/SUPT6H/BST2/VAMP2/TRPC6/TWIST1/CCR2/TNFRSF4/YWHAG/CA7/CACNA1E/CACNB2/PAX8/FZD5/RAB7A/RAB11FIP1/CALR/NR0B2/CASQ1/RAE1/SCIN/IRS2/TNFRSF11A/SPHK1/SYT7/RSAD2/REEP6/ADIPOQ/RAB3D/NUP93/FGF19	115
GO:0046903	secretion	77/901	1008/17046	0.00068	0.01379	0.01084	ADCY3/TMED10/CHGA/CHI3L1/EXOC3/CIDEA/CCR1/ADM/CTGF/NLRP6/ABAT/DRD4/AGXT/A2M/UNC13D/FGA/FGF10/EXPH5/STEAP2/GLS2/VPS4A/GNAS/GPER1/FFAR2/SCG3/ANXA2/NRG1/HLA-E/IGF1/IGF2/IL1RN/IL6/AQP5/INHBA/AQP9/ISL1/LGALS9/LLGL1/NOV/OPRL1/PARK2/PDE4C/PIK3CG/PML/TLR9/TREM1/POMC/GOLPH3L/TRPV6/SMPD3/SYBU/PRKAR1B/TRIM27/EXOC4/SCT/NOD2/SGK1/VPS33A/SLC6A12/VAMP2/TWIST1/CCR2/TNFRSF4/CACNA1E/PAX8/RAB7A/RAB11FIP1/NR0B2/MON1A/SCIN/IRS2/ACTN1/TNFRSF11A/SYT7/RSAD2/ADIPOQ/RAB3D	77
GO:0042110	T cell activation	37/901	400/17046	0.00069	0.01382	0.01087	MAP3K8/CYLD/DDOST/EGR3/FLOT2/MTOR/HLA-DOA/HLA-DPA1/HLA-E/HLX/ZC3H12D/IGF1/IGF2/IL6/IRF1/ITGB2/LCK/LCP1/LGALS9/SMAD3/NFATC3/LEF1/PIK3CG/IL20RB/APBB1IP/PAG1/NOD2/BMP4/ZEB1/CCR2/TNFRSF4/ZAP70/FZD5/SLA2/FADD/RSAD2/CD8A	37

GO:0070489	T cell aggregation	37/901	400/17046	0.00069	0.01382	0.01087	MAP3K8/CYLD/DDOST/EGR3/FLOT2/MTOR/HLA-DOA/HLA-DPA1/HLA-E/HLX/ZC3H12D/IGF1/IGF2/IL6/IRF1/ITGB2/LCK/LCP1/LGALS9/SMAD3/NFATC3/LEF1/PIK3CG/IL20RB/APBB1IP/PAG1/NOD2/BMP4/ZEB1/CCR2/TNFRSF4/ZAP70/FZD5/SLA2/FADD/RSAD2/CD8A	37
GO:0071320	cellular response to cAMP	9/901	47/17046	0.00069	0.01382	0.01087	CPS1/CYP11A1/EGR3/AQP9/PIK3CG/SLC8A1/WNT10B/ADIPOQ/RAPGEF2	9
GO:0018108	peptidyl-tyrosine phosphorylation	31/901	316/17046	0.0007	0.01397	0.01098	ABI1/IL31RA/EGFR/EPHA1/EPHA3/EPHB4/FGF10/MTOR/NPTN/DOK7/NRG1/IGF1/IGF2/IL6/IL12RB2/ISL1/ITGB2/KDR/HES5/LCK/NTF3/ANGPT4/MAPK3/MAP2K2/PXN/TRIM27/NOD2/SFRP2/TNFRSF1A/ZAP70/ADIPOQ	31
GO:0071396	cellular response to lipid	35/901	372/17046	0.0007	0.01398	0.011	SPON2/ADCY3/CPS1/CYP11A1/EGFR/EPHA3/ESR1/SBNO2/MLC1/GNAS/GPER1/FFAR2/GSTP1/NR4A1/IL6/INHBA/ISL1/ATP1A2/P2RY6/LEF1/MAPK3/PTGFR/BGLAP/BMP4/SOX9/TLR5/TNFAIP3/WNT10B/ZC3H12A/NR0B2/TRIM63/MGARP/ALDH1A2/LY86/NR1H4	35
GO:0002695	negative regulation of leukocyte activation	17/901	134/17046	0.0007	0.01399	0.01101	IL31RA/CYLD/GPER1/HLX/ZC3H12D/INHBA/IRF1/LGALS9/IL20RB/PAG1/BMP4/BPI/TNFAIP3/CCR2/LST1/ZC3H12A/SLA2	17
GO:0042327	positive regulation of phosphorylation	73/901	946/17046	0.0007	0.01399	0.01101	ABI1/ADCY3/CHI3L1/CCR1/MAP3K8/IL31RA/CTGF/ADRB3/DRD4/EGFR/FGA/FGF10/RASA3/MAPK8IP2/MTOR/GAPDHS/FGF22/NPTN/BMP10/GPER1/DOK7/GRB10/ANXA2/NRG1/HSP90AB1/IGF1/IGF2/CYR61/IL1RN/IL6/INHBA/ISL1/KDR/HES5/LCK/LGALS9/MAP3K1/NRAS/NTF3/OPRL1/ANGPT4/PIK3CG/PLA2G2A/TLR9/PRKAR1B/PRKD1/MAPK3/MAP2K2/PSMB4/PAK6/PSMD7/PXN/RASGRF2/CCL11/CCL17/NOD2/SFRP2/BMP4/SOX9/STK3/STK10/TNFRSF1A/CXCR4/FZD5/CARD14/CDK10/IRS2/TNFRSF11A/SPHK1/MAP3K6/ADIPOQ/RAPGEF2/FGF19	73
GO:0014033	neural crest cell differentiation	10/901	57/17046	0.00073	0.01436	0.01129	FOXC2/SOX8/NRG1/ISL1/LEF1/PITX2/SOX9/TWIST1/ALDH1A2/FGF19	10
GO:0050865	regulation of cell activation	39/901	430/17046	0.00073	0.01436	0.01129	MAP3K8/IL31RA/CTGF/CYLD/EGR3/UNC13D/FGF10/FLOT2/MTOR/GPER1/HLA-DOA/HLA-DPA1/HLA-E/HLX/ZC3H12D/IGF1/IGF2/IL6/INHBA/IRF1/LCK/LGALS9/IL20RB/PAG1/PTPRE/NOD2/BMP4/BPI/SUPT6H/ZEB1/TNFAIP3/CCR2/TNFRSF4/ZAP70/LST1/ZC3H12A/SLA2/IRS2/FADD	39
GO:0050777	negative regulation of immune response	15/901	111/17046	0.00073	0.01445	0.01136	NLRP6/A2M/HLA-B/HLA-E/HLX/LGALS9/IL20RB/MASP1/PSMB4/TRIM27/NOD2/BST2/TNFAIP3/CCR2/NLRX1	15
GO:2000026	regulation of multicellular organismal development	105/901	1465/17046	0.00075	0.01464	0.01151	CDH3/DMRT2/TBR1/LECT1/CHI3L1/CCR1/SEZ6/ADM/FAM101A/CTGF/CYLD/DMBT1/EGR3/EIF4G1/EPHA1/EPHA3/ESR1/FGF10/VASH1/ACIN1/FOXC2/SPG20/NEDD4L/MTOR/TENM4/NPTN/BMP10/GNAS/GPER1/SOX8/NRG1/HLA-B/HLA-DOA/HLX/ACACB/HOXB3/HOXD3/RSPO2/BARHL2/IGF1/CYR61/IL1RN/IL6/INHBA/IRF1/ISL1/KDR/AMIGO3/HES5/LAMA3/LCK/ARHGDIA/LGALS9/SMAD3/MITF/NEU1/NRAS/NTF3/PALM/PARK2/LEF1/CEND1/ANGPT4/ATP8A2/PML/SSH1/LIMS2/IFT122/PRKD1/MAP2K2/BGLAP/CCL11/SFRP2/BMP4/SLC8A1/BMPR1B/SLIT1/SOX9/STK3/SUPT6H/ZEB1/TNFAIP3/TNFRSF1A/PHLDA2/TWIST1/CCR2/WNT10B/YWHAG/ZAP70/PAX8/CXCR4/CALR/COLQ/SCRT1/HOPX/SCIN/RUNX1/TP63/FADD/SPHK1/CBFA2T2/ADIPOQ/H2AFY/RAPGEF2/ULK2	105
GO:0072511	divalent inorganic cation transport	37/901	402/17046	0.00075	0.0147	0.01156	TRDN/CLCA1/CCR1/C15orf27/TRPM6/CTGF/TRPV3/BHLHA15/DRD4/RASA3/CRACR2B/GPER1/ANXA6/LCK/ATP1A2/NFATC3/OPRL1/PIK3CG/PML/TLR9/ZDHHC13/TRPV6/SLC30A10/CHRNA9/PRKD1/TRPV5/TRPC7/TRIM27/BMP4/SLC8A1/TRPC4/TRPC6/TRPM2/CACNA1E/CACNB2/CASQ1/SMDT1	37
GO:0032870	cellular response to hormone stimulus	56/901	684/17046	0.00076	0.01472	0.01158	TCIRG1/NPFFR2/ADCY3/AP3S1/CPS1/CYP11A1/EGFR/EGR3/EIF4G1/ESR1/FGF10/RASA3/FOXC2/FOXO1/MTOR/FGF22/GNAS/GPER1/GRB10/NRG1/NR4A1/IGF2/IL6/INHBA/ISL1/LCK/LHCGR/LMO2/ATP1A2/NRAS/P2RY6/LEF1/PRKAG3/TLR9/PRKAR1B/LMBRD1/MAPK3/MAP2K2/PSMB4/PSMD7/PTGFR/PTPRE/PXN/RASGRF2/BMP4/VAMP2/WNT10B/PAX8/NR0B2/TRIM63/MGARP/IRS2/ADIPOQ/RAPGEF2/FGF19/NR1H4	56
GO:0006171	cAMP biosynthetic process	16/901	123/17046	0.00076	0.01473	0.01158	NPFFR2/ADCY3/ADM/ADRB3/DRD4/GABBR1/GNAS/GPER1/HPCA/LHCGR/MC2R/OPRL1/PALM/MRAP/SCT/CCR2	16
GO:0044723	single-organism carbohydrate metabolic process	55/901	669/17046	0.00076	0.01475	0.0116	GNE/B4GALT7/GALNT15/CLN5/SLC51B/NEU4/GALM/CPS1/PARP4/B3GLCT/MGAT5B/DDOST/ENO2/TRAK1/FOXO1/AKR1B1/MTOR/FUCA1/SLC37A4/ST6GALNAC3/GBGT1/GAPDHS/SLC17A5/DHDH/GPER1/EOGT/GRB10/HAS1/HK1/HRH1/IGF1/IGF2/IL6/MUC21/LHCGR/MGAT1/NEU1/OAS2/PARK2/CHST15/SIRT6/GALNT7/PGAM2/PKM/PRKAG3/POMC/PPP1CB/PPP1CC/CSGALNACT1/PHLDA2/MOGS/CALR/IRS2/STBD1/ADIPOQ	55
GO:0018212	peptidyl-tyrosine modification	31/901	318/17046	0.00077	0.01491	0.01172	ABI1/IL31RA/EGFR/EPHA1/EPHA3/EPHB4/FGF10/MTOR/NPTN/DOK7/NRG1/IGF1/IGF2/IL6/IL12RB2/ISL1/ITGB2/KDR/HES5/LCK/NTF3/ANGPT4/MAPK3/MAP2K2/PXN/TRIM27/NOD2/SFRP2/TNFRSF1A/ZAP70/ADIPOQ	31
GO:0061323	cell proliferation involved in heart morphogenesis	5/901	15/17046	0.00079	0.01506	0.01184	BMP10/ISL1/PITX2/BMP4/SOX9	5
GO:0003338	metanephros morphogenesis	7/901	30/17046	0.00079	0.01506	0.01184	FGF10/SOX8/FMN1/HES5/BMP4/SOX9/PAX8	7

GO:0002862	negative regulation of inflammatory response to antigenic stimulus	4/901	9/17046	0.00079	0.01506	0.01185	NLRP6/IL20RB/PSMB4/NOD2	4
GO:1901293	nucleoside phosphate biosynthetic process	31/901	319/17046	0.00081	0.0155	0.01219	TCIRG1/NPFFR2/ADCY3/ACOT7/ADM/ADRB3/DRD4/GABBR1/AMPD2/AMPD3/GNAS/GPER1/GUCY1A3/NME7/ACACB/HPCA/NME9/LHCGR/MC2R/ME1/NPPC/OAS2/OPRL1/ATP5B/PALM/PKM/MRAP/SCT/CCR2/UPP1/KMO	31
GO:0071704	organic substance metabolic process	555/901	9633/17046	0.00083	0.01579	0.01242	AKT3/ABI1/CDH3/GNE/ZNF783/TSPAN5/CDH13/SUGP2/MBNL2/FARP1/CDKN1C/SPEG/BCKDK/TCIRG1/C1D/ZBTB18/PITRM1/MTHFS/PDPN/DMRT2/CELF1/CELF2/TBR1/HCST/NPFFR2/ADCY3/PNRC1/TMED10/SLC27A2/LECT1/ADAM29/HNRNPUL1/RPP14/HIBADH/CHI3L1/ERLIN2/PSIP1/CHI3L2/EGLN2/B4GALT7/ACOT7/EXOC3/ADPRHL1/CARD16/ZBED9/CIDEA/ALPK2/GALNT15/CLCA1/CLN5/MRPL52/CCR1/SLC51B/CNP/APOA1BP/NEU4/COL9A3/COL11A1/GALM/MAP3K8/ZFP42/ADM/IL31RA/EGFLAM/UBLCP1/HUS1B/CPD/CPM/CPS1/PXDNL/CRABP1/ZNF358/TRPM6/MIB2/PARP4/LDLRAD3/B3GLCT/MGAT5B/CSTA/ZNF738/CTGF/SMYD1/PPM1L/SH3D19/CYLD/MBOAT1/ADRB3/ESCO2/CYP11A1/ZNF782/FITM1/ADAL/ZNF709/ZNF781/CITED4/DDB1/LONRF2/DDOST/RNF168/ZNF366/BHLHA15/NLRP6/DIO3/DLG2/DNMT3A/ABAT/DPH1/DRD4/ECE1/AGXT/EEF2/EGFR/EGR3/PATL2/EIF4G1/A2M/ELK4/LIPH/ENO2/ADCK5/EPHA1/EPHA3/EPHB4/ESR1/ALAS1/F11/FAH/PRSS54/RNF182/SP8/FGA/FGF10/FHIT/XRN2/RASA3/PPM1E/SBNO2/TRAK1/MSRB2/ACIN1/FOXL1/FOXC2/FOXO1/AKR1B1/GGA3/DIP2A/FLOT2/NUP210/NEDD4L/LARP1/PUM2/RYBP/MORC3/MAPK8IP2/TSSK2/VGLL2/MTOR/FUCA1/SLC37A4/GABBR1/RNF144B/ZNF549/ST6GALNAC3/GAK/SAMM50/PNKD/TENM4/ACOT11/LTN1/FBXL21/FBXO2/LCE2B/SACS/GATM/GBGT1/GAPDHS/SLC17A5/RPS6KC1/PABPC1/DNAJC2/FGF22/NPTN/AMPD2/PDE7B/DKK3/GLS2/VPS4A/AMPD3/DHDH/BMP10/ZNF638/GNAS/ZNF311/TMPRSS12/PIGW/ZNF844/THEM5/GPER1/EOGT/DOK7/GRB10/MRPS18B/ZBTB44/DNAJC15/GSTP1/GTF2B/BRF1/GUCY1A3/NME7/PADI1/GZMA/ANXA2/HAS1/SERPIND1/SOX8/NRG1/HK1/HLX/HMGA1/NR4A1/ACACB/HPCA/APBA2/HOXB3/HOXC4/HOXC5/HOXC6/HOXD3/HRH1/HSD11B1/HSD17B2/ACADL/HSPA1L/HSP90AA1/HSP90AB1/DUPD1/ADAMTSL5/TFAP2E/ID3/ZC3H12D/COL28A1/FMN1/BARHL2/NME9/IGF1/IGF2/CYR61/LCE1C/LCE1D/LCE2D/IL1RN/IL6/IL12RB2/PRSS41/IL16/FOXK2/INHBA/INPP5A/IRF1/ISL1/ITGB2/ITIH3/ITIH4/IVL/JUP/USP50/HILS1/KCNH2/KDR/ACAT1/HES5/AFF3/LCK/MUC21/LDLR/LGALS9/LHCGR/LMNA/LMO2/LOX/LTB/SMAD3/MC2R/ME1/ME2/MEF2D/MAP3K1/MEOX1/MEOX2/MFI2/MGAT1/MITF/LHX8/MOCS1/MOV10/NUDT1/MYH4/NDUFB4/DRG1/NEU1/ATP1A2/NFATC3/NFYB/NHLH2/NOV/NPPC/NRAS/NTF3/OAS2/OPRL1/PAFAH2/ATP5B/PALM/PARK2/SPOCK3/UTP11L/LEF1/DDX47/PRR16/CHST15/ANGPT4/PDE4C/PCYOX1/PDE7A/SIRT6/PDE6B/GALNT7/PGAM2/PI3/PIGC/PIK3CG/PITX2/PKHD1/PKM/PLA2G2A/PLAGL1/PRKAG3/PML/RIPPLY3/SLCO1C1/PNLIP/RIPK4/TLR9/TREM1/CYTL1/POMC/SSH1/PON1/MOV10L1/POU2AF1/BNC2/MED18/CYP2W1/RPP25/LPCAT2/BANP/PPP1CB/HERC6/PPP1CC/PIWIL2/ELP3/PRMT6/DNAJC17/ZNF532/PPP2R2B/FANCI/WDR33/SMPD3/CNOT11/VAC14/PRKAR1B/LMBRD1/CSGALNACT1/PRKD1/WSB2/MYNN/APOBR/MAPK3/MAP2K2/PROC/MRAP/PRMT8/MASP1/HTRA1/PSMB4/PAK6/ARNTL2/RGMA/PRDM11/PSMD7/PTGFR/TENM2/GATAD2B/KLHL8/RDH14/METTL14/MARK4/CCAR2/PTPRE/PXN/CREBZF/ACTA2/RASGRF2/RFC2/TRIM27/RGR/RGS12/EXOC4/RPA3/RPL8/RPL29/SCT/CCL11/CCL17/ABHD4/MRPS14/PRSS22/NPAS3/NOD2/STRA6/SFRP2/TRA2B/GZF1/SGK1/CERK/BMP4/ZNF649/BMPR1B/BRD9/ZSCAN18/BOK/SOX9/SRP68/STAT2/STK3/STK10/SUPT6H/BST2/VAMP2/TAF4B/TBP/TCEA1/TCEB2/ZEB1/ACTC1/TEAD3/TERF1/TGM2/TIMP3/TLE3/TNFAIP3/TNFRSF1A/TNXB/TRAF1/TRAF5/PHLDA2/TWIST1/CCR2/TNFRSF4/UCP1/UPP1/VARS/WNT10B/YWHAG/ZAP70/ZNF7/ZNF124/ZNF177/PTP4A1/MOGS/PAX8/CXCR4/FZD5/RAB7A/ERI3/CARD14/GDPD3/CERS4/ZNF665/EPHX3/ZC3H14/ERMP1/ZNF606/ZC3H12A/FAAP100/CPEB4/COL18A1/ZNF436/EEPD1/CALR/QTRT1/SLIRP/COLQ/CAST/SCRT1/HIST1H3A/SLA2/ZNF397/NR0B2/HOPX/TTBK1/SPINK7/TRIM63/KDM2B/LOXL3/GTPBP3/CBX2/RAE1/GAS7/CDK10/KMO/RUNX1/TP63/RUNX3/SERPINA6/IRS2/ACTN1/CRADD/FADD/TNFRSF11A/ALDH1A2/STK19/SYNJ2/SPHK1/BUD31/CCNA1/ENDOU/STBD1/LIMD1/CH25H/ERI1/PIAS2/ZFAND2A/MAP3K6/LDB2/SLC16A3/CBFA2T2/AURKB/NEURL3/SDR42E1/ADIPOQ/RAB3D/H2AFY/ENTPD3/PREPL/MICAL2/N4BP1/VGLL4/NUP93/RAPGEF2/ULK2/ZBTB39/LPGAT1/FGF19/NR1H4	555
GO:0048545	response to steroid hormone	36/901	390/17046	0.00084	0.01582	0.01244	ADM/CPS1/CTGF/CYP11A1/CITED4/ZNF366/DNMT3A/DRD4/AGXT/EGFR/ESR1/FGA/FGF10/GJB2/GPER1/NR4A1/HTR5A/IL1RN/IL6/ISL1/LOX/ATP1A2/OPRL1/LEF1/PTGFR/BGLAP/BMP4/SLC9A3/TIMP3/CALR/NR0B2/TRIM63/MGARP/ALDH1A2/ADIPOQ/NR1H4	36
GO:0032881	regulation of polysaccharide metabolic process	8/901	39/17046	0.00084	0.01584	0.01246	MTOR/GRB10/IGF1/IGF2/POMC/PPP1CB/PHLDA2/IRS2	8
GO:0098771	inorganic ion homeostasis	49/901	581/17046	0.00084	0.01584	0.01246	TCIRG1/TRDN/CLN5/CCR1/ADM/DRD4/ESR1/NEDD4L/STEAP2/NPTN/GPER1/FLVCR1/ANXA6/KCNH2/KDR/LCK/MFI2/NUBP1/ATP1A2/OPRL1/ATP5B/PARK2/PDE6B/PIK3CG/PKHD1/PML/SLC30A10/CHRNA9/PRKD1/SLAMF8/TRPC7/CCL11/SGK1/BMP4/SLC4A1/SLC8A1/SLC9A3/TGM2/TRPC4/TRPC6/CCR2/CA7/CXCR4/RAB7A/CALR/ATP13A4/CASQ1/SMDT1/MTL5	49
GO:0005976	polysaccharide metabolic process	14/901	101/17046	0.00085	0.01584	0.01246	CPS1/MTOR/GRB10/HAS1/IGF1/IGF2/PRKAG3/POMC/PPP1CB/PPP1CC/CSGALNACT1/PHLDA2/IRS2/STBD1	14
GO:0010876	lipid localization	31/901	320/17046	0.00086	0.01599	0.01258	ABCA9/SLC27A2/CIDEA/SLC51B/FITM1/DRD4/SLC10A4/ATP11A/VPS4A/FFAR2/ACACB/IL6/INHBA/AQP9/ATP9B/LDLR/ANO7/PARK2/ATP8A2/SLCO1C1/PNLIP/POMC/PON1/APOBR/ABHD4/STRA6/ZC3H12A/IRS2/TNFRSF11A/ADIPOQ/NR1H4	31

GO:0043255	regulation of carbohydrate biosynthetic process	12/901	79/17046	0.00086	0.01599	0.01258	FOXO1/MTOR/GPER1/GRB10/HRH1/IGF1/IGF2/IL6/LHCGR/PPP1CB/IRS2/ADIPOQ	12
GO:0048568	embryonic organ development	38/901	420/17046	0.00089	0.01639	0.01289	CDKN1C/GJB6/COL11A1/ADM/EGFR/FGF10/FOXC2/GATM/GNAS/FLVCR1/HLX/HOXB3/HOXC4/HOXD3/ID3/CYR61/KDR/LMO2/SMAD3/LEF1/ATP8A2/PITX2/CHRNA9/IFT122/SCT/STRA6/BMP4/SLC8A1/SOX9/STK3/ZEB1/TWIST1/PAX8/FZD5/KDM2B/RUNX1/ALDH1A2/MICAL2	38
GO:0030817	regulation of cAMP biosynthetic process	15/901	113/17046	0.00089	0.01639	0.01289	NPFFR2/ADM/ADRB3/DRD4/GABBR1/GNAS/GPER1/HPCA/LHCGR/MC2R/OPRL1/PALM/MRAP/SCT/CCR2	15
GO:0042325	regulation of phosphorylation	97/901	1341/17046	0.00089	0.01639	0.01289	ABI1/CDKN1C/NPFFR2/ADCY3/CHI3L1/CCR1/MAP3K8/IL31RA/CTGF/ADRB3/NLRP6/DRD4/EGFR/EPHA1/FGA/FGF10/RASA3/PPM1E/FOXO1/MAPK8IP2/MTOR/GAPDHS/FGF22/NPTN/BMP10/GPER1/DOK7/GRB10/DNAJC15/GSTP1/ANXA2/NRG1/HSP90AB1/IGF1/IGF2/CYR61/IL1RN/IL6/INHBA/ISL1/ITGB2/KDR/HES5/LCK/LGALS9/SMAD3/MAP3K1/NRAS/NTF3/OPRL1/PARK2/ANGPT4/SIRT6/PIK3CG/PKHD1/PLA2G2A/PML/TLR9/ELP3/VAC14/PRKAR1B/PRKD1/MAPK3/MAP2K2/SLAMF8/PSMB4/PAK6/PSMD7/PXN/RASGRF2/TRIM27/CCL11/CCL17/NOD2/SFRP2/BMP4/SOX9/STK3/STK10/TNFAIP3/TNFRSF1A/TNXB/TWIST1/TNFRSF4/YWHAG/CXCR4/FZD5/CARD14/CDK10/IRS2/TNFRSF11A/SPHK1/MAP3K6/ADIPOQ/H2AFY/RAPGEF2/FGF19	97
GO:0051173	positive regulation of nitrogen compound metabolic process	117/901	1672/17046	0.0009	0.0165	0.01297	CDH13/CDKN1C/DMRT2/TBR1/ERLIN2/PSIP1/ADM/IL31RA/ADRB3/CITED4/RNF168/BHLHA15/EEF2/EGFR/ESR1/FGF10/SBNO2/FOXC2/FOXO1/LARP1/VGLL2/MTOR/GAPDHS/PABPC1/DNAJC2/BMP10/GNAS/GPER1/BRF1/GUCY1A3/SOX8/HMGA1/NR4A1/HPCA/HSP90AA1/HSP90AB1/TFAP2E/BARHL2/IGF1/IGF2/CYR61/IL6/FOXK2/INHBA/IRF1/ISL1/JUP/HES5/LGALS9/LHCGR/LMNA/LMO2/SMAD3/MC2R/MEF2D/MEOX1/MEOX2/MITF/NFATC3/NFYB/NHLH2/NPPC/NTF3/PARK2/LEF1/PRR16/PITX2/PLAGL1/RIPK4/TLR9/CYTL1/POMC/BANP/PIWIL2/PRKD1/MAPK3/MRAP/ARNTL2/RGMA/TRIM27/SCT/NPAS3/NOD2/SFRP2/TRA2B/BMP4/ZNF649/BMPR1B/SOX9/STK3/SUPT6H/TBP/TCEA1/TCEB2/ZEB1/TEAD3/TLR5/TNFRSF1A/TRAF1/TRAF5/TWIST1/WNT10B/PAX8/FZD5/CARD14/CALR/RUNX1/TP63/RUNX3/FADD/TNFRSF11A/SPHK1/PIAS2/LDB2/CBFA2T2/MICAL2/NR1H4	117
GO:0007159	leukocyte cell-cell adhesion	39/901	435/17046	0.00091	0.01661	0.01306	MAP3K8/CYLD/DDOST/EGR3/FLOT2/MTOR/HLA-DOA/HLA-DPA1/HLA-E/HLX/ZC3H12D/IGF1/IGF2/IL6/IRF1/ITGB2/ITGB7/LCK/LCP1/LGALS9/SMAD3/NFATC3/LEF1/PIK3CG/IL20RB/APBB1IP/PAG1/NOD2/BMP4/STK10/ZEB1/CCR2/TNFRSF4/ZAP70/FZD5/SLA2/FADD/RSAD2/CD8A	39
GO:0001667	ameboidal-type cell migration	28/901	280/17046	0.00092	0.01683	0.01323	CDH13/EGR3/EPHB4/FAT2/FGF10/VASH1/FOXC2/BMP10/HAS1/SOX8/NR4A1/ISL1/ITGB7/KDR/MCC/NOV/LEF1/ANGPT4/PITX2/PML/PRKD1/PLEKHG5/BMP4/SLC8A1/SOX9/TWIST1/SH3BGRL3/FGF19	28
GO:0007507	heart development	40/901	450/17046	0.00092	0.01683	0.01323	SPEG/COL11A1/ADM/SMYD1/ECE1/EPHB4/FOXL1/FOXC2/MTOR/TENM4/BMP10/NRG1/ACACB/ID3/CYR61/ISL1/KCNJ8/LMNA/SMAD3/MEF2D/MYL2/NFATC3/SIRT6/PITX2/RIPPLY3/PARVA/IFT122/STRA6/SFRP2/BMP4/SLC8A1/SOX9/STK3/ACTC1/TWIST1/CALR/HOPX/ALDH1A2/MICAL2/FGF19	40
GO:0034109	homotypic cell-cell adhesion	41/901	465/17046	0.00094	0.01705	0.01341	MAP3K8/CYLD/DDOST/EGR3/FGA/FLOT2/MTOR/GNAS/HLA-DOA/HLA-DPA1/HLA-E/HLX/ZC3H12D/IGF1/IGF2/IL6/IRF1/ITGB2/JUP/LCK/LCP1/LGALS9/SMAD3/NFATC3/LEF1/PIK3CG/IL20RB/APBB1IP/PAG1/NOD2/BMP4/STK10/ZEB1/CCR2/TNFRSF4/ZAP70/FZD5/SLA2/FADD/RSAD2/CD8A	41
GO:0050728	negative regulation of inflammatory response	13/901	91/17046	0.00096	0.01742	0.0137	NLRP6/GPER1/GSTP1/ISL1/SMAD3/NOV/IL20RB/PSMB4/NOD2/TNFAIP3/TNFRSF1A/NLRX1/ADIPOQ	13
GO:0001818	negative regulation of cytokine production	22/901	201/17046	0.00098	0.01776	0.01397	CDH3/CIDEA/CYLD/GSTP1/IL6/INHBA/LGALS9/LEF1/PML/IL20RB/TLR9/POMC/TRIM27/NOD2/BPI/BST2/TNFAIP3/TWIST1/TNFRSF4/NLRX1/ZC3H12A/ADIPOQ	22
GO:0050927	positive regulation of positive chemotaxis	6/901	23/17046	0.001	0.01788	0.01406	CDH13/FGF10/IL16/KDR/SMAD3/NTF3	6
GO:0072243	metanephric nephron epithelium development	6/901	23/17046	0.001	0.01788	0.01406	SOX8/ACAT1/HES5/SOX9/PAX8/ADIPOQ	6

GO:0016310	phosphorylation	134/901	1964/17046	0.00102	0.01825	0.01435	AKT3/ABI1/GNE/CDKN1C/SPEG/BCKDK/HCST/NPFFR2/ADCY3/CHI3L1/ALPK2/CCR1/MAP3K8/IL31RA/TRPM6/CTGF/PPM1L/ADRB3/NLRP6/DRD4/EGFR/ENO2/ADCK5/EPHA1/EPHA3/EPHB4/FGA/FGF10/RASA3/PPM1E/FOXO1/MORC3/MAPK8IP2/TSSK2/MTOR/GAK/GAPDHS/RPS6KC1/FGF22/NPTN/BMP10/GPER1/DOK7/GRB10/DNAJC15/GSTP1/NME7/ANXA2/NRG1/HK1/HSP90AB1/NME9/IGF1/IGF2/CYR61/IL1RN/IL6/IL12RB2/INHBA/ISL1/ITGB2/KCNH2/KDR/HES5/LCK/LGALS9/SMAD3/MAP3K1/NDUFB4/NRAS/NTF3/OPRL1/PARK2/ANGPT4/SIRT6/PGAM2/PIK3CG/PKHD1/PKM/PLA2G2A/PRKAG3/PML/RIPK4/TLR9/ELP3/VAC14/PRKAR1B/PRKD1/MAPK3/MAP2K2/SLAMF8/PSMB4/PAK6/PSMD7/MARK4/PXN/RASGRF2/TRIM27/CCL11/CCL17/NOD2/SFRP2/SGK1/CERK/BMP4/BMPR1B/SOX9/STK3/STK10/TNFAIP3/TNFRSF1A/TNXB/TWIST1/TNFRSF4/YWHAG/ZAP70/CXCR4/FZD5/CARD14/TTBK1/CDK10/RUNX3/IRS2/TNFRSF11A/STK19/SPHK1/LIMD1/MAP3K6/AURKB/ADIPOQ/H2AFY/RAPGEF2/ULK2/FGF19	134
GO:0023014	signal transduction by protein phosphorylation	64/901	817/17046	0.00102	0.01825	0.01435	NPFFR2/CHI3L1/CCR1/MAP3K8/IL31RA/CTGF/PPM1L/ADRB3/NLRP6/DRD4/EGFR/FGA/FGF10/RASA3/FOXO1/MAPK8IP2/FGF22/BMP10/GPER1/GSTP1/NRG1/IGF1/IGF2/CYR61/IL1RN/IL6/INHBA/KCNH2/KDR/LGALS9/MAP3K1/NRAS/NTF3/PARK2/PIK3CG/PKHD1/PLA2G2A/TLR9/MAPK3/MAP2K2/PSMB4/PAK6/PSMD7/PXN/RASGRF2/CCL11/CCL17/NOD2/SFRP2/BMP4/BMPR1B/SOX9/STK3/STK10/TNXB/CXCR4/FZD5/CDK10/IRS2/TNFRSF11A/MAP3K6/ADIPOQ/RAPGEF2/FGF19	64
GO:0051050	positive regulation of transport	65/901	833/17046	0.00103	0.01837	0.01444	CDH3/TRDN/CCR1/SLC51B/TRPV3/ABAT/DRD4/EGFR/UNC13D/FGA/EXPH5/MLC1/NEDD4L/GLS2/VPS4A/GPR26/GPER1/FFAR2/ANXA2/NRG1/ANXA13/HLA-E/HPCA/HSPA1L/HSP90AB1/IGF1/IL6/INHBA/ISL1/JUP/KCNH2/IPO5/LGALS9/SMAD3/NTF3/OPRL1/PARK2/ATP8A2/TLR9/PON1/GOLPH3L/SMPD3/SCT/NOD2/SFRP2/SGK1/BMP4/VAMP2/TRPC6/TWIST1/TNFRSF4/YWHAG/CACNB2/FZD5/RAB7A/CALR/NR0B2/SCIN/IRS2/TNFRSF11A/SPHK1/SYT7/ADIPOQ/RAB3D/FGF19	65
GO:0006873	cellular ion homeostasis	43/901	497/17046	0.00104	0.01837	0.01444	TCIRG1/TRDN/CLN5/CCR1/ADM/DRD4/ESR1/NEDD4L/NPTN/GPER1/FLVCR1/ANXA6/LCK/NUBP1/ATP1A2/OPRL1/ATP5B/PDE6B/PIK3CG/PKHD1/PML/SLC30A10/CHRNA9/PRKD1/SLAMF8/TRPC7/CCL11/SGK1/BMP4/SLC4A1/SLC8A1/TGM2/TRPC4/TRPC6/CCR2/CA7/CXCR4/RAB7A/CALR/ATP13A4/CASQ1/SMDT1/MTL5	43
GO:0009408	response to heat	18/901	151/17046	0.00104	0.01837	0.01444	PSIP1/TRPV3/NUP210/MTOR/DNAJC2/HSPA1L/HSP90AA1/HSP90AB1/IGF1/IL1R1/IL6/C11orf73/MAPK3/CCAR2/RPA3/CASQ1/RAE1/NUP93	18
GO:2001236	regulation of extrinsic apoptotic signaling pathway	18/901	151/17046	0.00104	0.01837	0.01444	CYLD/FGA/FGF10/GPER1/GSTP1/NRG1/IGF1/INHBA/LMNA/PML/SFRP2/BMPR1B/STK3/TNFAIP3/TRAF1/BCL2L14/RUNX3/FADD	18
GO:1904019	epithelial cell apoptotic process	11/901	70/17046	0.00105	0.01839	0.01446	DNMT3A/FGA/GPER1/IL6/KDR/PIK3CG/BMP4/STK3/TNFAIP3/COL18A1/CAST	11
GO:0030808	regulation of nucleotide biosynthetic process	17/901	139/17046	0.00107	0.01854	0.01458	NPFFR2/ADM/ADRB3/DRD4/GABBR1/GNAS/GPER1/GUCY1A3/HPCA/LHCGR/MC2R/NPPC/OPRL1/PALM/MRAP/SCT/CCR2	17
GO:1900371	regulation of purine nucleotide biosynthetic process	17/901	139/17046	0.00107	0.01854	0.01458	NPFFR2/ADM/ADRB3/DRD4/GABBR1/GNAS/GPER1/GUCY1A3/HPCA/LHCGR/MC2R/NPPC/OPRL1/PALM/MRAP/SCT/CCR2	17
GO:0030282	bone mineralization	13/901	92/17046	0.00107	0.01854	0.01458	CCR1/FAM101A/SBNO2/RSPO2/IGF1/SMAD3/BGLAP/BMP4/SLC8A1/BMPR1B/SOX9/TWIST1/WNT10B	13
GO:0032609	interferon-gamma production	13/901	92/17046	0.00107	0.01854	0.01458	HLA-DPA1/IL12RB2/INHBA/ISL1/LGALS9/IL20RB/TLR9/TRIM27/NOD2/CCR2/FZD5/RUNX3/FADD	13
GO:0030814	regulation of cAMP metabolic process	16/901	127/17046	0.00108	0.01855	0.01459	NPFFR2/ADM/ADRB3/DRD4/GABBR1/GNAS/GPER1/HPCA/LHCGR/MC2R/NPPC/OPRL1/PALM/MRAP/SCT/CCR2	16
GO:0043467	regulation of generation of precursor metabolites and energy	12/901	81/17046	0.00108	0.01855	0.01459	MTOR/GAPDHS/GRB10/DNAJC15/IGF1/IGF2/SIRT6/POMC/PPP1CB/CISD1/PHLDA2/IRS2	12
GO:0045445	myoblast differentiation	12/901	81/17046	0.00108	0.01855	0.01459	SMYD1/FLOT2/SOX8/NRG1/ID3/IGF1/ISL1/CCL17/BMP4/SOX9/WNT10B/CAST	12

GO:0006112	energy reserve metabolic process	19/901	164/17046	0.00108	0.0186	0.01463	ADCY3/CPS1/ADRB3/MTOR/GNAS/GRB10/ACACB/IGF1/IGF2/PRKAG3/POMC/PPP1CB/PPP1CC/PRKAR1B/VAMP2/PHLDA2/CACNA1E/IRS2/STBD1	19
GO:0022008	neurogenesis	109/901	1548/17046	0.0011	0.01878	0.01477	FARP1/CDKN1C/SPON2/ZBTB18/TACC2/TBR1/GPRIN1/CLN5/SEZ6/CNP/COL9A3/SCLT1/ADM/APCDD1/CYP11A1/DNMT3A/EFNA2/EGFR/EIF4G1/EML1/EPHA1/EPHA3/EPHB4/FGF10/RASA3/BTBD3/SPG20/NFASC/EPB41L3/NEDD4L/PSD3/MAPK8IP2/DFNB31/TENM4/FGF22/NPTN/GPER1/SOX8/KCNIP2/NRG1/HOXB3/HOXD3/HSP90AA1/HSP90AB1/ID3/RSPO2/FMN1/BARHL2/IGF1/IL6/INHBA/ISL1/HES5/STMN1/ARHGDIA/LLGL1/LHX8/NEU1/NRAS/NTF3/PALM/PARK2/LEF1/CEND1/ATP8A2/PITX2/SSH1/MXRA8/PPP1CC/ELP3/PRKD1/MAPK3/MAP2K2/PSMB4/RGMA/TRPC7/PSMD7/TENM2/RASGRF2/S100A6/SGK1/BMP4/BMPR1B/SLIT1/BOK/SOX9/ZEB1/TRPC4/TRPC6/TWIST1/WNT10B/YWHAG/CACNB2/CXCR4/FZD5/LST1/CALR/SCRT1/PARD6B/GAS7/RUNX1/RUNX3/IRS2/ALDH1A2/CBFA2T2/ARHGEF10/RAPGEF2/ULK2/FGF19	109
GO:0001101	response to acid chemical	28/901	284/17046	0.00115	0.01955	0.01538	CPS1/CTGF/CYP11A1/DNMT3A/EGFR/EPHA3/AKR1B1/MTOR/GNAS/FFAR2/HPCA/HSD17B2/IL6/AQP2/IPO5/P2RY6/PON1/PTGFR/BMP4/SOX9/ZEB1/TIMP3/WNT10B/CPEB4/COL18A1/ALDH1A2/ADIPOQ/NR1H4	28
GO:0010906	regulation of glucose metabolic process	13/901	93/17046	0.00118	0.02009	0.0158	FOXO1/MTOR/GRB10/IGF1/IGF2/IL6/PARK2/PGAM2/POMC/PPP1CB/PHLDA2/IRS2/ADIPOQ	13
GO:0045747	positive regulation of Notch signaling pathway	7/901	32/17046	0.00119	0.02009	0.0158	TSPAN5/FGF10/HES5/MFNG/NOV/NOD2/TP63	7
GO:0030198	extracellular matrix organization	34/901	369/17046	0.00119	0.02009	0.0158	B4GALT7/COL9A3/COL11A1/COMP/EGFLAM/CTGF/A2M/FGA/FOXC2/BMP10/ANXA2/HAS1/HSP90AB1/COL28A1/CYR61/ITGA7/ITGB2/ITGB7/KDR/LAMA3/LCP1/LOX/LTBP1/SMAD3/MFI2/PLEC/CSGALNACT1/SFRP2/BMP4/SOX9/TNXB/COL18A1/COL21A1/ACTN1	34
GO:0035239	tube morphogenesis	33/901	355/17046	0.0012	0.02012	0.01582	ADM/EGFR/ESR1/FGF10/FOXC2/SOX8/HLX/RSPO2/FMN1/IGF1/KDR/HES5/SMAD3/NFATC3/LEF1/PITX2/PML/IFT122/PXN/CCL11/STRA6/SFRP2/GZF1/BMP4/SOX9/STK3/ZEB1/TGM2/TWIST1/PAX8/KDM2B/TP63/MICAL2	33
GO:0001942	hair follicle development	12/901	82/17046	0.0012	0.02012	0.01582	CDH3/APCDD1/EGFR/FGF10/GNAS/INHBA/SOX9/WNT10B/RUNX1/TP63/RUNX3/LDB2	12
GO:0022404	molting cycle process	12/901	82/17046	0.0012	0.02012	0.01582	CDH3/APCDD1/EGFR/FGF10/GNAS/INHBA/SOX9/WNT10B/RUNX1/TP63/RUNX3/LDB2	12
GO:0022405	hair cycle process	12/901	82/17046	0.0012	0.02012	0.01582	CDH3/APCDD1/EGFR/FGF10/GNAS/INHBA/SOX9/WNT10B/RUNX1/TP63/RUNX3/LDB2	12
GO:0032940	secretion by cell	68/901	886/17046	0.00123	0.02046	0.01609	ADCY3/TMED10/CHGA/CHI3L1/EXOC3/CIDEA/CCR1/ADM/CTGF/ABAT/DRD4/A2M/UNC13D/FGA/FGF10/EXPH5/STEAP2/GLS2/VPS4A/GNAS/GPER1/FFAR2/SCG3/HLA-E/IGF1/IGF2/IL1RN/IL6/INHBA/ISL1/LGALS9/LLGL1/NOV/PARK2/PDE4C/PIK3CG/PML/TLR9/TREM1/POMC/GOLPH3L/TRPV6/SMPD3/SYBU/PRKAR1B/TRIM27/EXOC4/SCT/NOD2/VPS33A/SLC6A12/VAMP2/TWIST1/CCR2/TNFRSF4/CACNA1E/PAX8/RAB7A/RAB11FIP1/NR0B2/MON1A/SCIN/IRS2/ACTN1/SYT7/RSAD2/ADIPOQ/RAB3D	68
GO:0043062	extracellular structure organization	34/901	370/17046	0.00124	0.02069	0.01627	B4GALT7/COL9A3/COL11A1/COMP/EGFLAM/CTGF/A2M/FGA/FOXC2/BMP10/ANXA2/HAS1/HSP90AB1/COL28A1/CYR61/ITGA7/ITGB2/ITGB7/KDR/LAMA3/LCP1/LOX/LTBP1/SMAD3/MFI2/PLEC/CSGALNACT1/SFRP2/BMP4/SOX9/TNXB/COL18A1/COL21A1/ACTN1	34
GO:0035518	histone H2A monoubiquitination	4/901	10/17046	0.00126	0.02085	0.0164	DDB1/RNF168/RYBP/KDM2B	4
GO:0072173	metanephric tubule morphogenesis	4/901	10/17046	0.00126	0.02085	0.0164	SOX8/HES5/SOX9/PAX8	4
GO:0018149	peptide cross-linking	9/901	51/17046	0.00127	0.0209	0.01644	EGFLAM/CSTA/LCE2B/LCE1C/LCE1D/LCE2D/IVL/SPOCK3/TGM2	9
GO:0050926	regulation of positive chemotaxis	6/901	24/17046	0.00127	0.0209	0.01644	CDH13/FGF10/IL16/KDR/SMAD3/NTF3	6

GO:0009058	biosynthetic process	338/901	5595/17046	0.00128	0.02097	0.01649	CDH3/GNE/ZNF783/CDH13/CDKN1C/TCIRG1/C1D/ZBTB18/MTHFS/DMRT2/CELF1/TBR1/NPFFR2/ADCY3/PNRC1/SLC27A2/HNRNPUL1/ERLIN2/PSIP1/EGLN2/B4GALT7/ACOT7/ZBED9/CIDEA/GALNT15/MRPL52/SLC51B/NEU4/ZFP42/ADM/IL31RA/CPS1/NDUFAF6/ZNF358/PARP4/B3GLCT/MGAT5B/ZNF738/CTGF/SMYD1/PPM1L/CYLD/MBOAT1/ADRB3/ESCO2/CYP11A1/ZNF782/FITM1/ADAL/ZNF709/ZNF781/CITED4/DDB1/DDOST/RNF168/ZNF366/BHLHA15/DIO3/DNMT3A/ABAT/DPH1/DRD4/AGXT/EEF2/EGFR/EGR3/PATL2/EIF4G1/ELK4/ENO2/ESR1/ALAS1/SP8/FGF10/FHIT/XRN2/SBNO2/TRAK1/MSRB2/FOXL1/FOXC2/FOXO1/AKR1B1/NUP210/NEDD4L/LARP1/PUM2/RYBP/VGLL2/MTOR/GABBR1/ZNF549/ST6GALNAC3/GATM/GBGT1/GAPDHS/SLC17A5/PABPC1/DNAJC2/AMPD2/DKK3/GLS2/AMPD3/BMP10/ZNF638/GNAS/ZNF311/PIGW/ZNF844/GPER1/EOGT/GRB10/MRPS18B/ZBTB44/GSTP1/GTF2B/BRF1/GUCY1A3/NME7/PADI1/HAS1/SOX8/NRG1/HLX/HMGA1/NR4A1/ACACB/HPCA/HOXB3/HOXC4/HOXC5/HOXC6/HOXD3/HRH1/HSD11B1/HSD17B2/ACADL/HSP90AA1/HSP90AB1/TFAP2E/ID3/BARHL2/NME9/IGF1/IGF2/CYR61/IL6/IL16/FOXK2/INHBA/IRF1/ISL1/JUP/USP50/HILS1/KDR/ACAT1/HES5/AFF3/MUC21/LDLR/LGALS9/LHCGR/LMO2/LTB/SMAD3/MC2R/ME1/MEF2D/MEOX1/MEOX2/MGAT1/MITF/LHX8/MOCS1/MOV10/NUBP1/DRG1/NEU1/NFATC3/NFYB/NHLH2/NPPC/NTF3/OAS2/OPRL1/ATP5B/PALM/PARK2/LEF1/PRR16/CHST15/SIRT6/GALNT7/PGAM2/PIGC/PIK3CG/PITX2/PKHD1/PKM/PLA2G2A/PLAGL1/PRKAG3/PML/RIPPLY3/RIPK4/TLR9/CYTL1/POMC/POU2AF1/BNC2/MED18/LPCAT2/BANP/PPP1CB/PIWIL2/ELP3/PRMT6/DNAJC17/ZNF532/CNOT11/VAC14/CSGALNACT1/PRKD1/MYNN/MAPK3/MRAP/PRMT8/PAK6/ARNTL2/RGMA/PRDM11/TENM2/GATAD2B/METTL14/CCAR2/CREBZF/RFC2/TRIM27/RGS12/RPA3/RPL8/RPL29/SCT/MRPS14/NPAS3/NOD2/SFRP2/GZF1/SGK1/BMP4/ZNF649/BMPR1B/BRD9/ZSCAN18/SOX9/SRP68/STAT2/STK3/SUPT6H/TAF4B/TBP/TCEA1/TCEB2/ZEB1/TEAD3/TERF1/TLE3/TLR5/TNFAIP3/TNFRSF1A/TRAF1/TRAF5/TWIST1/CCR2/TNFRSF4/UCP1/UPP1/VARS/WNT10B/YWHAG/ZNF7/ZNF124/ZNF177/MOGS/PAX8/FZD5/CARD14/CERS4/ZNF665/ZNF606/ZC3H12A/CPEB4/ZNF436/CALR/QTRT1/SLIRP/SCRT1/HIST1H3A/SLA2/ZNF397/NR0B2/HOPX/KDM2B/LOXL3/CBX2/RAE1/GAS7/KMO/RUNX1/TP63/RUNX3/IRS2/ACTN1/FADD/TNFRSF11A/ALDH1A2/SYNJ2/SPHK1/BUD31/CCNA1/LIMD1/CH25H/PIAS2/LDB2/CBFA2T2/AURKB/SDR42E1/ADIPOQ/H2AFY/MICAL2/VGLL4/NUP93/RAPGEF2/ZBTB39/LPGAT1/FGF19/NR1H4	338
GO:0001932	regulation of protein phosphorylation	91/901	1258/17046	0.00128	0.02097	0.01649	ABI1/CDKN1C/NPFFR2/ADCY3/CHI3L1/CCR1/MAP3K8/IL31RA/CTGF/ADRB3/NLRP6/DRD4/EGFR/EPHA1/FGA/FGF10/RASA3/PPM1E/FOXO1/MAPK8IP2/MTOR/FGF22/NPTN/BMP10/GPER1/DOK7/GSTP1/ANXA2/NRG1/HSP90AB1/IGF1/IGF2/CYR61/IL1RN/IL6/INHBA/ISL1/ITGB2/KDR/HES5/LCK/LGALS9/SMAD3/MAP3K1/NRAS/NTF3/OPRL1/PARK2/ANGPT4/PIK3CG/PKHD1/PLA2G2A/PML/TLR9/ELP3/PRKAR1B/PRKD1/MAPK3/MAP2K2/PSMB4/PAK6/PSMD7/PXN/RASGRF2/TRIM27/CCL11/CCL17/NOD2/SFRP2/BMP4/SOX9/STK3/STK10/TNFAIP3/TNFRSF1A/TNXB/TWIST1/TNFRSF4/YWHAG/CXCR4/FZD5/CARD14/CDK10/IRS2/TNFRSF11A/SPHK1/MAP3K6/ADIPOQ/H2AFY/RAPGEF2/FGF19	91
GO:0043408	regulation of MAPK cascade	58/901	731/17046	0.00129	0.02112	0.0166	NPFFR2/CHI3L1/CCR1/MAP3K8/CTGF/ADRB3/NLRP6/DRD4/EGFR/FGA/FGF10/RASA3/FOXO1/MAPK8IP2/FGF22/BMP10/GPER1/GSTP1/NRG1/IGF1/IGF2/CYR61/IL1RN/IL6/INHBA/KDR/LGALS9/MAP3K1/NRAS/NTF3/PARK2/PIK3CG/PKHD1/PLA2G2A/TLR9/MAPK3/MAP2K2/PSMB4/PAK6/PSMD7/PXN/RASGRF2/CCL11/CCL17/NOD2/SFRP2/BMP4/STK3/TNXB/CXCR4/FZD5/CDK10/IRS2/TNFRSF11A/MAP3K6/ADIPOQ/RAPGEF2/FGF19	58
GO:0045785	positive regulation of cell adhesion	32/901	343/17046	0.00133	0.02168	0.01705	CDH13/MAP3K8/EGFLAM/EGR3/UNC13D/EPHA1/FGA/FOXC2/FLOT2/MTOR/NRG1/HLA-DPA1/HLA-E/HLX/FMN1/IGF1/IGF2/CYR61/IL6/KDR/LCK/LGALS9/SMAD3/LEF1/APBB1IP/NOD2/SFRP2/TGM2/CCR2/ZAP70/CALR/FADD	32
GO:0051234	establishment of localization	265/901	4274/17046	0.00134	0.02168	0.01705	ABI1/CDH3/TSPAN5/CDH13/KCNMB2/TCIRG1/TRDN/ABCA9/SPON2/COG5/ADCY3/TMED10/SLC27A2/RER1/CHGA/CHI3L1/PKP3/EXOC3/CHRNA1/CHRNA2/CHRNA5/CIDEA/PANX3/RBP7/AP3S1/CLCA1/CCR1/SLC51B/C15orf27/SLC38A10/CNP/ADM/CRABP1/TRPM6/PARP4/LDLRAD3/KLC3/CTGF/ABCC13/SH3D19/CYB561/CYLD/TRPV3/DDOST/BHLHA15/NLRP6/DMBT1/ABAT/DRD4/AGXT/EGFR/A2M/UNC13D/SLC10A4/FCGR2A/FGA/FGF10/RASA3/TRAK1/EXPH5/NFASC/GGA3/FLOT2/MLC1/NUP210/ATP11A/NEDD4L/SYNE1/MAPK8IP2/MTOR/SLC37A4/SAMM50/SEC31B/STEAP2/SLC17A5/GJA3/GJB2/SDCBP2/GLS2/VPS4A/GNAS/CRACR2B/GPR26/GPER1/FFAR2/GRB10/FLVCR1/GRIK4/DNAJC15/SCG3/NME7/ANXA2/KCNIP2/NRG1/ANXA6/HK1/ANXA13/HLA-E/ACACB/HPCA/APBA2/HSPA1L/HSP90AA1/HSP90AB1/HTR3A/IGF1/IGF2/IL1RN/IL6/AQP2/AQP5/INHBA/AQP9/ISL1/JUP/ATP9B/KCNH2/KCNJ8/KCNJ9/KCNMB1/IPO5/INSC/TOMM20L/SLC6A17/LCK/LCP1/LDLR/LGALS9/LLGL1/LMNA/RAB19/SMAD3/MCC/MFI2/ASGR1/ATP1A2/NFATC3/NOV/NRAS/NTF3/OPRL1/SLC22A18/P2RY6/ATP5B/ANO7/PALM/PARK2/PDE4C/PCYOX1/C11orf73/SIRT6/ATP8A2/PIK3CG/LRP1B/PRKAG3/PML/FXYD6/SLCO1C1/PNLIP/TLR9/TREM1/POMC/PON1/RIN2/ZDHHC13/DNAJC17/GOLPH3L/SLC47A1/SLC29A3/TRPV6/SMPD3/SLC30A10/CHRNA9/SYBU/PEX26/PRKAR1B/IFT122/LMBRD1/PRKD1/APOBR/MAPK3/MAP2K2/TRPV5/MASP1/CDC42SE1/TRPC7/RASGRF2/TRIM27/EXOC4/RPL8/RPL29/S100A6/SCT/NOD2/TINAGL1/STRA6/SFRP2/SGK1/VPS33A/BMP4/SLC4A1/ZG16/SLC6A12/SLC8A1/SLC9A3/SLC20A2/SLIT1/SRP68/SUPT6H/BST2/VAMP2/TGM2/TRAPPC10/TNFAIP3/TNFRSF1A/TRPC4/TRPC6/TRPM2/TWIST1/CCR2/TNFRSF4/UCP1/YWHAG/CA7/CACNA1E/CACNB2/PAX8/FZD5/RAB7A/RAB11FIP1/CALR/UNC93B1/COLQ/DYNLRB2/SLC25A18/MFSD7/ATP13A4/NR0B2/MON1A/CASQ1/LOXL3/MGARP/RAE1/SLC43A1/SCIN/SERPINA6/IRS2/ACTN1/TNFRSF11A/SPHK1/ENDOU/SYT7/SLC16A3/RSAD2/SMDT1/REEP6/TRIP10/ADIPOQ/RAB3D/H2AFY/RAB36/NUP93/USP6NL/FGF19/NR1H4	265
GO:0002764	immune response-regulating signaling pathway	49/901	594/17046	0.00135	0.02186	0.01719	ABI1/TANK/MAP3K8/CYLD/NLRP6/DMBT1/EGFR/FCGR2A/FGF10/RASA3/FOXO1/PUM2/MTOR/FGF22/FFAR2/NRG1/HLA-DPA1/NR4A1/HSP90AA1/HSP90AB1/IRF1/ITGB2/LCK/MAP3K1/MOV10/NFATC3/NRAS/IL20RB/TLR9/PAG1/MAPK3/MAP2K2/PSMB4/PSMD7/RASGRF2/NOD2/TLR5/TNFAIP3/ZAP70/NLRX1/UNC93B1/SLA2/IRS2/FADD/SKAP2/RSAD2/RAPGEF2/CD79A/FGF19	49
GO:0080134	regulation of response to stress	95/901	1326/17046	0.00136	0.02186	0.01719	TANK/MAP3K8/CTGF/CYLD/RNF168/COCH/NLRP6/DMBT1/EGFR/A2M/F11/FGA/FGF10/VASH1/SBNO2/ACIN1/FOXC2/FOXO1/MLC1/NUP210/LARP1/PUM2/MAPK8IP2/MTOR/SLC37A4/DNAJC2/BMP10/GPER1/FFAR2/GSTP1/ANXA2/HK1/HLA-B/HLA-E/HMGA1/HSPA1L/HSP90AA1/HSP90AB1/IL1R1/IL1RN/IL6/IRF1/ISL1/ITGB2/LCK/LGALS9/SMAD3/MAP3K1/NFATC3/NOV/NRAS/PARK2/C11orf73/SIRT6/PIK3CG/PLA2G2A/PML/IL20RB/TLR9/TREM1/MAPK3/MAP2K2/PROC/MASP1/HTRA1/PSMB4/PSMD7/CCAR2/RPA3/NOD2/SFRP2/STAT2/STK3/TLR5/TNFAIP3/TNFRSF1A/TNXB/TWIST1/CCR2/CA7/FZD5/RAB7A/NLRX1/UNC93B1/HIST1H3A/HOPX/RAE1/TP63/FADD/TNFRSF11A/MAP3K6/RSAD2/ADIPOQ/NUP93/FGF19	95

GO:0009266	response to temperature stimulus	22/901	206/17046	0.00136	0.02186	0.01719	PSIP1/ADM/ADRB3/TRPV3/FOXO1/NUP210/MTOR/ACOT11/DNAJC2/HSPA1L/HSP90AA1/HSP90AB1/IGF1/IL1R1/IL6/C11orf73/MAPK3/CCAR2/RPA3/CASQ1/RAE1/NUP93	22
GO:1901564	organonitrogen compound metabolic process	135/901	1996/17046	0.00137	0.02203	0.01732	BCKDK/TCIRG1/MTHFS/CELF1/NPFFR2/ADCY3/TMED10/HIBADH/EGLN2/B4GALT7/ACOT7/CLN5/MRPL52/APOA1BP/NEU4/ADM/EGFLAM/CPS1/PPM1L/MBOAT1/ADRB3/ADAL/DDOST/DIO3/DLG2/DNMT3A/ABAT/DRD4/ECE1/AGXT/EEF2/PATL2/EIF4G1/ENO2/ALAS1/FAH/FHIT/AKR1B1/LARP1/PUM2/MTOR/FUCA1/GABBR1/ST6GALNAC3/PNKD/GATM/GAPDHS/PABPC1/AMPD2/PDE7B/GLS2/AMPD3/GNAS/GPER1/MRPS18B/DNAJC15/GSTP1/GUCY1A3/NME7/PADI1/HAS1/HK1/ACACB/HPCA/ACADL/BARHL2/NME9/IGF1/CYR61/IL6/ITIH3/ITIH4/ACAT1/LDLR/LHCGR/MC2R/ME1/ME2/MOCS1/NUDT1/MYH4/NDUFB4/NEU1/ATP1A2/NPPC/OAS2/OPRL1/ATP5B/PALM/PARK2/SPOCK3/PRR16/CHST15/PDE4C/PCYOX1/PDE7A/SIRT6/PDE6B/PGAM2/PKM/PLA2G2A/PML/CYTL1/POMC/PON1/LPCAT2/PIWIL2/SMPD3/CNOT11/LMBRD1/CSGALNACT1/PRKD1/MAPK3/MRAP/PSMB4/PSMD7/METTL14/RPL8/RPL29/SCT/MRPS14/NOD2/CERK/SRP68/CCR2/UPP1/VARS/PAX8/CERS4/CPEB4/CALR/QTRT1/KMO/SPHK1/NR1H4	135
GO:0097191	extrinsic apoptotic signaling pathway	23/901	220/17046	0.00142	0.02276	0.0179	CYLD/FGA/FGF10/GPER1/GSTP1/NRG1/IGF1/INHBA/LMNA/SMAD3/DDX47/PML/SFRP2/BMPR1B/BOK/STK3/TNFAIP3/TNFRSF1A/TRAF1/BCL2L14/RUNX3/CRADD/FADD	23
GO:0009615	response to virus	34/901	373/17046	0.00142	0.02276	0.0179	SPON2/HNRNPUL1/DMBT1/UNC13D/ACIN1/PUM2/SLC37A4/HMGA1/IL6/IRF1/KCNJ8/STMN1/LCK/LGALS9/OAS2/PML/TREM1/HTRA1/CREBZF/ACTA2/CCL11/STAT2/BST2/TNFAIP3/CA7/CXCR4/NLRX1/UNC93B1/HIST1H3A/IFITM1/FADD/RSAD2/CD8A/NUP93	34
GO:0048729	tissue morphogenesis	48/901	581/17046	0.00146	0.02318	0.01822	COL11A1/ADM/EGFR/ESR1/FGF10/FOXL1/FOXC2/BMP10/SOX8/NRG1/RSPO2/FMN1/IGF1/CYR61/IL6/INHBA/ISL1/KDR/HES5/SMAD3/MYL2/NFATC3/LEF1/SIRT6/ATP8A2/PITX2/PML/RIPK4/IFT122/PXN/ACTA2/CCL11/SFRP2/GZF1/BMP4/SOX9/STK3/ACTC1/TGM2/TWIST1/PAX8/FZD5/KDM2B/RUNX1/TP63/RUNX3/ALDH1A2/MICAL2	48
GO:0032760	positive regulation of tumor necrosis factor production	9/901	52/17046	0.00146	0.02318	0.01822	SPON2/HLA-E/ISL1/LGALS9/TLR9/NOD2/TWIST1/CCR2/FADD	9
GO:2001238	positive regulation of extrinsic apoptotic signaling pathway	9/901	52/17046	0.00146	0.02318	0.01822	CYLD/GPER1/INHBA/PML/BMPR1B/STK3/BCL2L14/RUNX3/FADD	9
GO:0019637	organophosphate metabolic process	75/901	1004/17046	0.00147	0.02318	0.01822	GNE/TCIRG1/NPFFR2/ADCY3/ACOT7/CNP/APOA1BP/ADM/CPS1/MBOAT1/ADRB3/FITM1/ADAL/DLG2/DRD4/ENO2/FHIT/GABBR1/GAPDHS/AMPD2/PDE7B/AMPD3/GNAS/PIGW/THEM5/GPER1/DNAJC15/GUCY1A3/NME7/HK1/ACACB/HPCA/HRH1/NME9/IGF1/INPP5A/LDLR/LHCGR/MC2R/ME1/ME2/MGAT1/MOCS1/NUDT1/MYH4/NDUFB4/ATP1A2/NPPC/OAS2/OPRL1/ATP5B/PALM/PDE4C/PDE7A/SIRT6/PDE6B/PGAM2/PIGC/PIK3CG/PKM/PLA2G2A/PON1/LPCAT2/SMPD3/VAC14/CSGALNACT1/MRAP/SCT/NOD2/CCR2/UPP1/KMO/SYNJ2/ENTPD3/LPGAT1	75
GO:0098773	skin epidermis development	12/901	84/17046	0.00149	0.02337	0.01837	CDH3/APCDD1/EGFR/FGF10/GNAS/INHBA/SOX9/WNT10B/RUNX1/TP63/RUNX3/LDB2	12
GO:0060337	type I interferon signaling pathway	11/901	73/17046	0.00149	0.02337	0.01837	HLA-B/HLA-E/HLA-F/HSP90AB1/IRF1/OAS2/STAT2/BST2/IFITM1/FADD/RSAD2	11
GO:0071357	cellular response to type I interferon	11/901	73/17046	0.00149	0.02337	0.01837	HLA-B/HLA-E/HLA-F/HSP90AB1/IRF1/OAS2/STAT2/BST2/IFITM1/FADD/RSAD2	11
GO:0010556	regulation of macromolecule biosynthetic process	228/901	3621/17046	0.0015	0.0234	0.0184	ZNF783/CDH13/CDKN1C/C1D/ZBTB18/DMRT2/CELF1/TBR1/PNRC1/HNRNPUL1/ERLIN2/PSIP1/EGLN2/ZBED9/CIDEA/SLC51B/ZFP42/IL31RA/ZNF358/ZNF738/CTGF/SMYD1/CYLD/ESCO2/ZNF782/ZNF709/ZNF781/CITED4/RNF168/ZNF366/BHLHA15/DNMT3A/EEF2/EGFR/EGR3/PATL2/EIF4G1/ELK4/ESR1/SP8/FGF10/FHIT/XRN2/SBNO2/TRAK1/MSRB2/FOXL1/FOXC2/FOXO1/SPG20/NEDD4L/LARP1/PUM2/RYBP/VGLL2/MTOR/ZNF549/PABPC1/DNAJC2/DKK3/BMP10/ZNF638/ZNF311/ZNF844/GPER1/GRB10/ZBTB44/GSTP1/GTF2B/BRF1/SOX8/NRG1/HLX/HMGA1/NR4A1/HOXB3/HOXC4/HOXC5/HOXC6/HOXD3/TFAP2E/ID3/BARHL2/IGF1/IGF2/CYR61/IL6/IL16/FOXK2/INHBA/IRF1/ISL1/JUP/USP50/HILS1/KDR/HES5/AFF3/LGALS9/LMNA/LMO2/LTB/SMAD3/MEF2D/MEOX1/MEOX2/MITF/LHX8/MOV10/NFATC3/NFYB/NHLH2/NTF3/PARK2/LEF1/PRR16/SIRT6/PITX2/PKHD1/PLAGL1/PML/RIPPLY3/RIPK4/TLR9/CYTL1/POMC/POU2AF1/BNC2/MED18/BANP/PPP1CB/PIWIL2/ELP3/PRMT6/DNAJC17/ZNF532/CNOT11/PRKD1/MYNN/MAPK3/PRMT8/HTRA1/PAK6/ARNTL2/RGMA/PRDM11/TENM2/GATAD2B/METTL14/CCAR2/CREBZF/TRIM27/NPAS3/NOD2/SFRP2/GZF1/SGK1/BMP4/ZNF649/BMPR1B/BRD9/ZSCAN18/SOX9/STAT2/STK3/SUPT6H/TAF4B/TBP/TCEA1/TCEB2/ZEB1/TEAD3/TERF1/TLE3/TNFAIP3/TNFRSF1A/TRAF1/TRAF5/TWIST1/CCR2/TNFRSF4/UCP1/VARS/WNT10B/ZNF7/ZNF124/ZNF177/PAX8/FZD5/CARD14/ZNF665/ZNF606/ZC3H12A/CPEB4/ZNF436/CALR/SLIRP/SCRT1/HIST1H3A/SLA2/ZNF397/NR0B2/HOPX/KDM2B/LOXL3/CBX2/GAS7/RUNX1/TP63/RUNX3/IRS2/ACTN1/FADD/TNFRSF11A/SPHK1/BUD31/CCNA1/LIMD1/PIAS2/LDB2/CBFA2T2/AURKB/ADIPOQ/H2AFY/MICAL2/VGLL4/ZBTB39/NR1H4	228

GO:0009892	negative regulation of metabolic process	159/901	2412/17046	0.0015	0.02341	0.01841	FARP1/CDKN1C/C1D/ZBTB18/CELF1/ERLIN2/CARD16/CIDEA/CCR1/CSTA/CTGF/SMYD1/CYLD/RNF168/ZNF366/NLRP6/DLG2/DNMT3A/DRD4/EGFR/PATL2/A2M/ELK4/EPHA1/ESR1/PHACTR1/FGA/FHIT/PPM1E/SBNO2/ACIN1/FOXC2/FOXO1/DIP2A/FLOT2/NEDD4L/PUM2/RYBP/MTOR/GABBR1/PABPC1/DNAJC2/DKK3/GPER1/GRB10/DNAJC15/GSTP1/GZMA/ANXA2/SERPIND1/SOX8/NRG1/HMGA1/ACACB/HPCA/HOXB3/HOXC6/ACADL/HSP90AB1/ID3/COL28A1/IGF1/IL6/INHBA/IRF1/ISL1/ITIH3/ITIH4/HILS1/KIF25/IPO5/HES5/LGALS9/SMAD3/MITF/MOV10/NPPC/NTF3/OPRL1/PALM/PARK2/SPOCK3/LEF1/SIRT6/PI3/PIK3CG/PITX2/PKHD1/PML/RIPPLY3/TLR9/BANP/PIWIL2/PRMT6/DNAJC17/CNOT11/PRKAR1B/MAP2K2/MASP1/CDC42SE1/PSMB4/PSMD7/TENM2/GATAD2B/METTL14/CCAR2/CREBZF/TRIM27/NOD2/SFRP2/GZF1/BMP4/ZNF649/SOX9/SUPT6H/BST2/TBP/ZEB1/TERF1/TIMP3/TNFAIP3/TWIST1/CCR2/TNFRSF4/WNT10B/YWHAG/ZNF177/ZC3H14/ZC3H12A/CPEB4/CALR/SLIRP/CAST/SCRT1/HIST1H3A/SLA2/NR0B2/HOPX/SPINK7/KDM2B/LOXL3/CBX2/TP63/RUNX3/SERPINA6/IRS2/FADD/LIMD1/ERI1/PIAS2/CBFA2T2/AURKB/DAPL1/ADIPOQ/H2AFY/N4BP1/RAPGEF2/FGF19/NR1H4	159
GO:0044249	cellular biosynthetic process	328/901	5425/17046	0.00151	0.02347	0.01846	CDH3/GNE/ZNF783/CDH13/CDKN1C/TCIRG1/C1D/ZBTB18/MTHFS/DMRT2/CELF1/TBR1/NPFFR2/ADCY3/PNRC1/SLC27A2/HNRNPUL1/ERLIN2/PSIP1/EGLN2/B4GALT7/ACOT7/ZBED9/CIDEA/GALNT15/MRPL52/SLC51B/NEU4/ZFP42/ADM/IL31RA/CPS1/ZNF358/PARP4/B3GLCT/MGAT5B/ZNF738/CTGF/SMYD1/PPM1L/CYLD/MBOAT1/ADRB3/ESCO2/CYP11A1/ZNF782/FITM1/ADAL/ZNF709/ZNF781/CITED4/DDB1/DDOST/RNF168/ZNF366/BHLHA15/DNMT3A/ABAT/DPH1/DRD4/AGXT/EEF2/EGFR/EGR3/PATL2/EIF4G1/ELK4/ESR1/ALAS1/SP8/FGF10/FHIT/XRN2/SBNO2/TRAK1/MSRB2/FOXL1/FOXC2/FOXO1/AKR1B1/NUP210/NEDD4L/LARP1/PUM2/RYBP/VGLL2/MTOR/GABBR1/ZNF549/ST6GALNAC3/GATM/GBGT1/SLC17A5/PABPC1/DNAJC2/AMPD2/DKK3/GLS2/AMPD3/BMP10/ZNF638/GNAS/ZNF311/PIGW/ZNF844/GPER1/EOGT/GRB10/MRPS18B/ZBTB44/GSTP1/GTF2B/BRF1/GUCY1A3/NME7/PADI1/HAS1/SOX8/NRG1/HLX/HMGA1/NR4A1/ACACB/HPCA/HOXB3/HOXC4/HOXC5/HOXC6/HOXD3/HRH1/ACADL/HSP90AA1/HSP90AB1/TFAP2E/ID3/BARHL2/NME9/IGF1/IGF2/CYR61/IL6/IL16/FOXK2/INHBA/IRF1/ISL1/JUP/USP50/HILS1/ACAT1/HES5/AFF3/MUC21/LDLR/LGALS9/LHCGR/LMO2/LTB/SMAD3/MC2R/ME1/MEF2D/MEOX1/MEOX2/MGAT1/MITF/LHX8/MOCS1/MOV10/DRG1/NEU1/NFATC3/NFYB/NHLH2/NPPC/NTF3/OAS2/OPRL1/ATP5B/PALM/PARK2/LEF1/PRR16/CHST15/SIRT6/GALNT7/PIGC/PIK3CG/PITX2/PKHD1/PKM/PLA2G2A/PLAGL1/PRKAG3/PML/RIPPLY3/RIPK4/TLR9/CYTL1/POMC/POU2AF1/BNC2/MED18/LPCAT2/BANP/PPP1CB/PIWIL2/ELP3/PRMT6/DNAJC17/ZNF532/CNOT11/VAC14/CSGALNACT1/PRKD1/MYNN/MAPK3/MRAP/PRMT8/PAK6/ARNTL2/RGMA/PRDM11/TENM2/GATAD2B/METTL14/CCAR2/CREBZF/RFC2/TRIM27/RGS12/RPA3/RPL8/RPL29/SCT/MRPS14/NPAS3/NOD2/SFRP2/GZF1/SGK1/BMP4/ZNF649/BMPR1B/BRD9/ZSCAN18/SOX9/SRP68/STAT2/STK3/SUPT6H/TAF4B/TBP/TCEA1/TCEB2/ZEB1/TEAD3/TERF1/TLE3/TLR5/TNFAIP3/TNFRSF1A/TRAF1/TRAF5/TWIST1/CCR2/TNFRSF4/UCP1/UPP1/VARS/WNT10B/YWHAG/ZNF7/ZNF124/ZNF177/MOGS/PAX8/FZD5/CARD14/CERS4/ZNF665/ZNF606/ZC3H12A/CPEB4/ZNF436/CALR/QTRT1/SLIRP/SCRT1/HIST1H3A/SLA2/ZNF397/NR0B2/HOPX/KDM2B/LOXL3/CBX2/RAE1/GAS7/KMO/RUNX1/TP63/RUNX3/IRS2/ACTN1/FADD/TNFRSF11A/ALDH1A2/SYNJ2/SPHK1/BUD31/CCNA1/LIMD1/CH25H/PIAS2/LDB2/CBFA2T2/AURKB/ADIPOQ/H2AFY/MICAL2/VGLL4/NUP93/RAPGEF2/ZBTB39/LPGAT1/FGF19/NR1H4	328
GO:0048754	branching morphogenesis of an epithelial tube	18/901	156/17046	0.00152	0.02352	0.0185	ESR1/FGF10/FOXC2/SOX8/RSPO2/IGF1/KDR/NFATC3/LEF1/PITX2/PML/PXN/CCL11/SFRP2/GZF1/BMP4/SOX9/PAX8	18
GO:0002221	pattern recognition receptor signaling pathway	19/901	169/17046	0.00155	0.02395	0.01883	TANK/MAP3K8/CYLD/NLRP6/DMBT1/PUM2/FFAR2/IRF1/ITGB2/MAP3K1/TLR9/MAPK3/NOD2/TLR5/TNFAIP3/NLRX1/UNC93B1/FADD/RSAD2	19
GO:0072273	metanephric nephron morphogenesis	6/901	25/17046	0.0016	0.02465	0.01938	SOX8/FMN1/HES5/BMP4/SOX9/PAX8	6
GO:0051249	regulation of lymphocyte activation	32/901	347/17046	0.00161	0.02478	0.01949	MAP3K8/CYLD/EGR3/FGF10/FLOT2/MTOR/HLA-DOA/HLA-DPA1/HLA-E/HLX/ZC3H12D/IGF1/IGF2/IL6/INHBA/IRF1/LCK/LGALS9/IL20RB/PAG1/NOD2/BMP4/SUPT6H/ZEB1/TNFAIP3/CCR2/TNFRSF4/ZAP70/LST1/SLA2/IRS2/FADD	32
GO:0030500	regulation of bone mineralization	10/901	63/17046	0.00162	0.0248	0.0195	CCR1/FAM101A/SMAD3/BGLAP/BMP4/SLC8A1/BMPR1B/SOX9/TWIST1/WNT10B	10
GO:0050880	regulation of blood vessel size	16/901	132/17046	0.00162	0.02487	0.01956	KCNMB2/MRVI1/ADM/CPS1/ADRB3/ECE1/FGA/FOXC2/GPER1/GUCY1A3/HRH1/KCNJ8/ATP1A2/NPPC/ACTA2/SLC8A1	16
GO:0030335	positive regulation of cell migration	31/901	333/17046	0.00163	0.02488	0.01957	CDH13/CCR1/EGFR/EPHA1/FGF10/FOXC2/GPER1/IGF1/CYR61/IL6/KDR/LGALS9/SMAD3/NTF3/P2RY6/LEF1/ANGPT4/ELP3/PRKD1/CCL11/BMP4/SLC8A1/SOX9/CCR2/PTP4A1/COL18A1/CALR/IRS2/FADD/SPHK1/RAPGEF2	31
GO:0060348	bone development	19/901	170/17046	0.00166	0.02527	0.01987	ABI1/SLC38A10/COMP/FAM101A/GNAS/ANXA2/IGF1/MEF2D/NPPC/PITX2/BNC2/CSGALNACT1/BGLAP/SFRP2/BMP4/BMPR1B/SOX9/TWIST1/C6orf25	19
GO:0060341	regulation of cellular localization	82/901	1122/17046	0.00167	0.0253	0.01989	CDH3/TRDN/RER1/CHGA/CIDEA/SLC51B/CYLD/ABAT/DRD4/EGFR/UNC13D/FGA/EXPH5/MLC1/NEDD4L/MTOR/GLS2/VPS4A/GNAS/GPR26/GPER1/FFAR2/NRG1/ANXA13/HLA-E/HPCA/HSPA1L/HSP90AB1/IGF1/IL1RN/IL6/INHBA/ISL1/JUP/IPO5/LCP1/LGALS9/LLGL1/LMNA/SMAD3/ATP1A2/NOV/PARK2/PDE4C/PML/TLR9/POMC/GOLPH3L/TRPV6/SMPD3/SYBU/PRKAR1B/PRKD1/MAPK3/MAP2K2/TRIM27/SCT/NOD2/SFRP2/BMP4/SLC8A1/SUPT6H/BST2/VAMP2/TWIST1/CCR2/TNFRSF4/YWHAG/CACNA1E/PAX8/FZD5/RAB7A/RAB11FIP1/NR0B2/CASQ1/IRS2/SPHK1/SYT7/RSAD2/REEP6/ADIPOQ/RAB3D	82

GO:0034340	response to type I interferon	11/901	74/17046	0.00167	0.0253	0.01989	HLA-B/HLA-E/HLA-F/HSP90AB1/IRF1/OAS2/STAT2/BST2/IFITM1/FADD/RSAD2	11
GO:0048546	digestive tract morphogenesis	9/901	53/17046	0.00168	0.02544	0.02	EGFR/FGF10/HLX/SMAD3/PITX2/STRA6/SFRP2/BMP4/TP63	9
GO:1902105	regulation of leukocyte differentiation	23/901	223/17046	0.0017	0.02564	0.02016	CCR1/CYLD/EGR3/ACIN1/MTOR/GNAS/HLA-B/HLA-DOA/HLX/IL6/INHBA/IRF1/LCK/LGALS9/MITF/LEF1/BGLAP/BMP4/ZEB1/ZAP70/RUNX1/FADD/ADIPOQ	23
GO:2000112	regulation of cellular macromolecule biosynthetic process	222/901	3525/17046	0.00175	0.02628	0.02067	ZNF783/CDH13/CDKN1C/C1D/ZBTB18/DMRT2/CELF1/TBR1/PNRC1/HNRNPUL1/ERLIN2/PSIP1/EGLN2/ZBED9/CIDEA/SLC51B/ZFP42/IL31RA/ZNF358/ZNF738/SMYD1/CYLD/ESCO2/ZNF782/ZNF709/ZNF781/CITED4/RNF168/ZNF366/BHLHA15/DNMT3A/EEF2/EGFR/EGR3/PATL2/EIF4G1/ELK4/ESR1/SP8/FGF10/FHIT/XRN2/SBNO2/TRAK1/MSRB2/FOXL1/FOXC2/FOXO1/SPG20/NEDD4L/LARP1/PUM2/RYBP/VGLL2/MTOR/ZNF549/PABPC1/DNAJC2/DKK3/BMP10/ZNF638/ZNF311/ZNF844/GPER1/GRB10/ZBTB44/GTF2B/BRF1/SOX8/NRG1/HLX/HMGA1/NR4A1/HOXB3/HOXC4/HOXC5/HOXC6/HOXD3/TFAP2E/ID3/BARHL2/IGF1/IGF2/CYR61/IL6/IL16/FOXK2/INHBA/IRF1/ISL1/JUP/USP50/HILS1/HES5/AFF3/LGALS9/LMNA/LMO2/SMAD3/MEF2D/MEOX1/MEOX2/MITF/LHX8/MOV10/NFATC3/NFYB/NHLH2/NTF3/PARK2/LEF1/PRR16/SIRT6/PITX2/PKHD1/PLAGL1/PML/RIPPLY3/RIPK4/TLR9/CYTL1/POMC/POU2AF1/BNC2/MED18/BANP/PPP1CB/PIWIL2/ELP3/PRMT6/DNAJC17/ZNF532/CNOT11/PRKD1/MYNN/MAPK3/PRMT8/HTRA1/PAK6/ARNTL2/RGMA/PRDM11/TENM2/GATAD2B/METTL14/CCAR2/CREBZF/TRIM27/NPAS3/NOD2/SFRP2/GZF1/SGK1/BMP4/ZNF649/BMPR1B/BRD9/ZSCAN18/SOX9/STAT2/STK3/SUPT6H/TAF4B/TBP/TCEA1/TCEB2/ZEB1/TEAD3/TERF1/TLE3/TNFAIP3/TNFRSF1A/TRAF1/TRAF5/TWIST1/TNFRSF4/UCP1/VARS/WNT10B/ZNF7/ZNF124/ZNF177/PAX8/FZD5/CARD14/ZNF665/ZNF606/ZC3H12A/CPEB4/ZNF436/CALR/SLIRP/SCRT1/HIST1H3A/SLA2/ZNF397/NR0B2/HOPX/KDM2B/LOXL3/CBX2/GAS7/RUNX1/TP63/RUNX3/IRS2/FADD/TNFRSF11A/SPHK1/BUD31/CCNA1/LIMD1/PIAS2/LDB2/CBFA2T2/AURKB/ADIPOQ/H2AFY/MICAL2/VGLL4/ZBTB39/NR1H4	222
GO:0035150	regulation of tube size	16/901	133/17046	0.00176	0.02638	0.02074	KCNMB2/MRVI1/ADM/CPS1/ADRB3/ECE1/FGA/FOXC2/GPER1/GUCY1A3/HRH1/KCNJ8/ATP1A2/NPPC/ACTA2/SLC8A1	16
GO:0035051	cardiocyte differentiation	14/901	109/17046	0.00179	0.0268	0.02108	SPEG/MTOR/TENM4/BMP10/NRG1/ISL1/LMNA/MYL2/PITX2/BMP4/SLC8A1/ACTC1/TWIST1/CALR	14
GO:0044093	positive regulation of molecular function	115/901	1672/17046	0.00181	0.02709	0.0213	ABI1/CDH3/FARP1/TRDN/PITRM1/ADCY3/CHI3L1/MAP3K8/CTGF/ADRB3/DRD4/EGFR/EPHA1/ESR1/SPATA13/FGF10/RASA3/TBC1D9B/TBC1D1/PSD3/ARHGEF18/MAPK8IP2/RASGEF1C/ALS2CL/RGS22/FGF22/CYTH4/GNAS/GPER1/DOK7/DNAJC15/ANXA2/NRG1/HPCA/AGFG2/HSP90AB1/IGF1/IGF2/CYR61/IL1RN/IL6/ISL1/JUP/LCK/ARHGDIA/LGALS9/LHCGR/LLGL1/SMAD3/MAP3K1/MFNG/PLEKHG7/NHLH2/NRAS/NTF3/ARHGEF3/PARK2/ANGPT4/PIK3CG/PITX2/PML/PNLIP/RIPK4/TLR9/CYTL1/PON1/RIN2/ARHGEF10L/PRKAR1B/PRKD1/MAPK3/MAP2K2/PSMB4/PAK6/PSMD7/PLEKHG5/PXN/RASGRF2/TRIM27/RGS12/CCL11/CCL17/NOD2/SFRP2/ARHGAP9/SGK1/BMP4/BOK/STK3/STK10/TCEA1/TRAF1/TRAF5/TRPC6/TWIST1/WNT10B/CXCR4/FZD5/CARD14/SH3BGRL3/IRS2/CRADD/FADD/TNFRSF11A/SPHK1/STARD13/MAP3K6/ADIPOQ/ARHGAP29/ARHGEF10/RAPGEF2/USP6NL/RABGAP1L/IQSEC1/FGF19	115
GO:1903506	regulation of nucleic acid-templated transcription	205/901	3227/17046	0.00182	0.02713	0.02134	ZNF783/CDH13/CDKN1C/C1D/ZBTB18/DMRT2/TBR1/PNRC1/HNRNPUL1/ERLIN2/PSIP1/EGLN2/ZBED9/CIDEA/ZFP42/IL31RA/ZNF358/ZNF738/SMYD1/CYLD/ZNF782/ZNF709/ZNF781/CITED4/RNF168/ZNF366/BHLHA15/DNMT3A/EGFR/EGR3/ELK4/ESR1/SP8/FGF10/FHIT/XRN2/SBNO2/TRAK1/MSRB2/FOXL1/FOXC2/FOXO1/SPG20/NEDD4L/RYBP/VGLL2/MTOR/ZNF549/DNAJC2/DKK3/BMP10/ZNF638/ZNF311/ZNF844/GPER1/ZBTB44/GTF2B/BRF1/SOX8/NRG1/HLX/HMGA1/NR4A1/HOXB3/HOXC4/HOXC5/HOXC6/HOXD3/TFAP2E/ID3/BARHL2/IGF1/IGF2/CYR61/IL6/IL16/FOXK2/INHBA/IRF1/ISL1/JUP/USP50/HILS1/HES5/AFF3/LGALS9/LMNA/LMO2/SMAD3/MEF2D/MEOX1/MEOX2/MITF/LHX8/MOV10/NFATC3/NFYB/NHLH2/NTF3/PARK2/LEF1/SIRT6/PITX2/PKHD1/PLAGL1/PML/RIPPLY3/RIPK4/TLR9/CYTL1/POMC/POU2AF1/BNC2/MED18/BANP/ELP3/PRMT6/DNAJC17/ZNF532/CNOT11/PRKD1/MYNN/MAPK3/PRMT8/HTRA1/PAK6/ARNTL2/RGMA/PRDM11/TENM2/GATAD2B/CCAR2/CREBZF/TRIM27/NPAS3/NOD2/SFRP2/GZF1/SGK1/BMP4/ZNF649/BMPR1B/BRD9/ZSCAN18/SOX9/STAT2/STK3/SUPT6H/TAF4B/TBP/TCEA1/TCEB2/ZEB1/TEAD3/TLE3/TNFAIP3/TNFRSF1A/TRAF1/TRAF5/TWIST1/TNFRSF4/UCP1/WNT10B/ZNF7/ZNF124/ZNF177/PAX8/FZD5/CARD14/ZNF665/ZNF606/ZC3H12A/ZNF436/CALR/SLIRP/SCRT1/HIST1H3A/SLA2/ZNF397/NR0B2/HOPX/KDM2B/LOXL3/CBX2/GAS7/RUNX1/TP63/RUNX3/ACTN1/FADD/TNFRSF11A/SPHK1/BUD31/CCNA1/LIMD1/PIAS2/LDB2/CBFA2T2/AURKB/ADIPOQ/H2AFY/MICAL2/VGLL4/ZBTB39/NR1H4	205
GO:0001656	metanephros development	12/901	86/17046	0.00183	0.02719	0.02138	FGF10/FOXC2/SOX8/ID3/FMN1/AQP2/ACAT1/HES5/BMP4/SOX9/PAX8/ADIPOQ	12
GO:0006355	regulation of transcription, DNA-templated	204/901	3210/17046	0.00184	0.02725	0.02143	ZNF783/CDH13/CDKN1C/C1D/ZBTB18/DMRT2/TBR1/PNRC1/HNRNPUL1/ERLIN2/PSIP1/EGLN2/ZBED9/CIDEA/ZFP42/IL31RA/ZNF358/ZNF738/SMYD1/CYLD/ZNF782/ZNF709/ZNF781/CITED4/RNF168/ZNF366/BHLHA15/DNMT3A/EGFR/EGR3/ELK4/ESR1/SP8/FGF10/FHIT/XRN2/SBNO2/TRAK1/MSRB2/FOXL1/FOXC2/FOXO1/SPG20/NEDD4L/RYBP/VGLL2/MTOR/ZNF549/DNAJC2/DKK3/BMP10/ZNF638/ZNF311/ZNF844/GPER1/ZBTB44/GTF2B/BRF1/SOX8/NRG1/HLX/HMGA1/NR4A1/HOXB3/HOXC4/HOXC5/HOXC6/HOXD3/TFAP2E/ID3/BARHL2/IGF1/IGF2/CYR61/IL6/IL16/FOXK2/INHBA/IRF1/ISL1/JUP/USP50/HILS1/HES5/AFF3/LGALS9/LMNA/LMO2/SMAD3/MEF2D/MEOX1/MEOX2/MITF/LHX8/MOV10/NFATC3/NFYB/NHLH2/NTF3/PARK2/LEF1/SIRT6/PITX2/PKHD1/PLAGL1/PML/RIPPLY3/RIPK4/TLR9/CYTL1/POMC/POU2AF1/BNC2/MED18/BANP/ELP3/PRMT6/DNAJC17/ZNF532/CNOT11/PRKD1/MYNN/MAPK3/PRMT8/HTRA1/PAK6/ARNTL2/RGMA/PRDM11/TENM2/GATAD2B/CCAR2/CREBZF/TRIM27/NPAS3/NOD2/SFRP2/GZF1/SGK1/BMP4/ZNF649/BMPR1B/BRD9/ZSCAN18/SOX9/STAT2/STK3/SUPT6H/TAF4B/TBP/TCEA1/TCEB2/ZEB1/TEAD3/TLE3/TNFAIP3/TNFRSF1A/TRAF1/TRAF5/TWIST1/TNFRSF4/UCP1/WNT10B/ZNF7/ZNF124/ZNF177/PAX8/FZD5/CARD14/ZNF665/ZNF606/ZC3H12A/ZNF436/CALR/SLIRP/SCRT1/HIST1H3A/SLA2/ZNF397/NR0B2/HOPX/KDM2B/LOXL3/CBX2/GAS7/RUNX1/TP63/RUNX3/FADD/TNFRSF11A/SPHK1/BUD31/CCNA1/LIMD1/PIAS2/LDB2/CBFA2T2/AURKB/ADIPOQ/H2AFY/MICAL2/VGLL4/ZBTB39/NR1H4	204

GO:0010604	positive regulation of macromolecule metabolic process	171/901	2632/17046	0.00184	0.02725	0.02143	ABI1/CDH3/CDH13/CDKN1C/DMRT2/TBR1/ADCY3/CHI3L1/ERLIN2/PSIP1/EGLN2/CCR1/SLC51B/MAP3K8/IL31RA/CTGF/SH3D19/ADRB3/CITED4/RNF168/BHLHA15/DRD4/ECE1/EEF2/EGFR/ESR1/FGA/FGF10/RASA3/SBNO2/FOXC2/FOXO1/GGA3/LARP1/MAPK8IP2/VGLL2/MTOR/RNF144B/PABPC1/DNAJC2/FGF22/NPTN/BMP10/GPER1/DOK7/BRF1/ANXA2/SOX8/NRG1/HMGA1/NR4A1/HOXD3/HSP90AB1/TFAP2E/BARHL2/IGF1/IGF2/CYR61/IL1RN/IL6/FOXK2/INHBA/IRF1/ISL1/JUP/KDR/HES5/LCK/LGALS9/LMNA/LMO2/LTB/SMAD3/MEF2D/MAP3K1/MEOX1/MEOX2/MFI2/MITF/NFATC3/NFYB/NHLH2/NRAS/NTF3/OPRL1/PARK2/LEF1/PRR16/ANGPT4/PIK3CG/PITX2/PLA2G2A/PLAGL1/PML/RIPK4/TLR9/CYTL1/POMC/BANP/PIWIL2/FANCI/PRKAR1B/PRKD1/MAPK3/MAP2K2/PSMB4/PAK6/ARNTL2/RGMA/PSMD7/PTGFR/PXN/ACTA2/RASGRF2/TRIM27/CCL11/CCL17/NPAS3/NOD2/SFRP2/TRA2B/BMP4/ZNF649/BMPR1B/BOK/SOX9/STK3/STK10/SUPT6H/TBP/TCEA1/TCEB2/ZEB1/ACTC1/TEAD3/TNFAIP3/TNFRSF1A/TRAF1/TRAF5/TWIST1/CCR2/WNT10B/PAX8/CXCR4/FZD5/RAB7A/CARD14/ZC3H12A/CALR/NR0B2/CDK10/RUNX1/TP63/RUNX3/IRS2/CRADD/FADD/TNFRSF11A/ALDH1A2/SPHK1/PIAS2/ZFAND2A/MAP3K6/LDB2/CBFA2T2/ADIPOQ/H2AFY/MICAL2/RAPGEF2/FGF19/NR1H4	171
GO:0051146	striated muscle cell differentiation	24/901	238/17046	0.00185	0.0273	0.02147	SPEG/SMYD1/BHLHA15/MTOR/BMP10/TMOD4/NRG1/IGF1/IGF2/LMNA/MYL2/NFATC3/NOV/LEF1/PITX2/BIN3/BMP4/SLC8A1/ACTC1/WNT10B/CALR/CAST/CASQ1/HOPX	24
GO:0000165	MAPK cascade	61/901	790/17046	0.00187	0.02754	0.02166	NPFFR2/CHI3L1/CCR1/MAP3K8/IL31RA/CTGF/PPM1L/ADRB3/NLRP6/DRD4/EGFR/FGA/FGF10/RASA3/FOXO1/MAPK8IP2/FGF22/BMP10/GPER1/GSTP1/NRG1/IGF1/IGF2/CYR61/IL1RN/IL6/INHBA/KDR/LGALS9/MAP3K1/NRAS/NTF3/PARK2/PIK3CG/PKHD1/PLA2G2A/TLR9/MAPK3/MAP2K2/PSMB4/PAK6/PSMD7/PXN/RASGRF2/CCL11/CCL17/NOD2/SFRP2/BMP4/SOX9/STK3/TNXB/CXCR4/FZD5/CDK10/IRS2/TNFRSF11A/MAP3K6/ADIPOQ/RAPGEF2/FGF19	61
GO:1900542	regulation of purine nucleotide metabolic process	21/901	198/17046	0.00188	0.02759	0.02169	NPFFR2/ADM/ADRB3/DRD4/GABBR1/GAPDHS/GNAS/GPER1/DNAJC15/GUCY1A3/HPCA/IGF1/LHCGR/MC2R/NPPC/OPRL1/PALM/SIRT6/MRAP/SCT/CCR2	21
GO:0061029	eyelid development in camera-type eye	4/901	11/17046	0.0019	0.02765	0.02175	INHBA/MAP3K1/STRA6/TWIST1	4
GO:0080184	response to phenylpropanoid	4/901	11/17046	0.0019	0.02765	0.02175	CIDEA/CYP11A1/FGA/BGLAP	4
GO:0022414	reproductive process	81/901	1111/17046	0.0019	0.02765	0.02175	CDKN1C/CELF1/ADCY3/ADAM29/COL9A3/ZFP42/ADM/CYP11A1/WBP2NL/DNMT3A/ABAT/DRD4/EGFR/ESR1/FGF10/XRN2/AKR1B1/MAPK8IP2/TSSK2/MTOR/GAPDHS/GJB2/GNAS/IZUMO1/HAS1/SOX8/HSD17B2/HSPA1L/HSP90AB1/IGF1/CYR61/IL1R1/IL1RN/INHBA/HILS1/KDR/LGALS9/LHCGR/LMNA/MC2R/LHX8/NPPC/OPRL1/LEF1/PGAM2/PI3/PITX2/SPA17/MOV10L1/PIWIL2/WDR33/HTRA1/PTGFR/TRIM27/RPL29/NPAS3/STRA6/SFRP2/BMP4/SLC8A1/BMPR1B/BOK/SOX9/STK3/TAF4B/TBP/TEAD3/TLR5/PHLDA2/FZD5/CALR/SLIRP/CAST/SPATA16/ANTXR1/KDM2B/CBX2/TP63/CCNA1/ENDOU/MTL5	81
GO:0032651	regulation of interleukin-1 beta production	8/901	44/17046	0.00192	0.02778	0.02185	GSTP1/ISL1/LGALS9/SMAD3/PML/NOD2/TNFAIP3/SPHK1	8
GO:0055008	cardiac muscle tissue morphogenesis	9/901	54/17046	0.00193	0.02778	0.02185	COL11A1/FOXC2/BMP10/NRG1/ISL1/MYL2/SIRT6/PITX2/ACTC1	9
GO:0060350	endochondral bone morphogenesis	9/901	54/17046	0.00193	0.02778	0.02185	COMP/GNAS/MEF2D/NPPC/BNC2/CSGALNACT1/BMP4/BMPR1B/SOX9	9
GO:1903557	positive regulation of tumor necrosis factor superfamily cytokine production	9/901	54/17046	0.00193	0.02778	0.02185	SPON2/HLA-E/ISL1/LGALS9/TLR9/NOD2/TWIST1/CCR2/FADD	9

GO:0051252	regulation of RNA metabolic process	211/901	3337/17046	0.00193	0.02784	0.02189	ZNF783/CDH13/MBNL2/CDKN1C/C1D/ZBTB18/DMRT2/CELF1/TBR1/PNRC1/HNRNPUL1/ERLIN2/PSIP1/EGLN2/ZBED9/CIDEA/ZFP42/IL31RA/ZNF358/ZNF738/SMYD1/CYLD/ZNF782/ZNF709/ZNF781/CITED4/RNF168/ZNF366/BHLHA15/DNMT3A/EGFR/EGR3/ELK4/ESR1/SP8/FGF10/FHIT/XRN2/SBNO2/TRAK1/MSRB2/ACIN1/FOXL1/FOXC2/FOXO1/SPG20/NEDD4L/RYBP/VGLL2/MTOR/ZNF549/PABPC1/DNAJC2/DKK3/BMP10/ZNF638/ZNF311/ZNF844/GPER1/ZBTB44/GTF2B/BRF1/SOX8/NRG1/HLX/HMGA1/NR4A1/HOXB3/HOXC4/HOXC5/HOXC6/HOXD3/TFAP2E/ID3/BARHL2/IGF1/IGF2/CYR61/IL6/IL16/FOXK2/INHBA/IRF1/ISL1/JUP/USP50/HILS1/HES5/AFF3/LGALS9/LMNA/LMO2/SMAD3/MEF2D/MEOX1/MEOX2/MITF/LHX8/MOV10/NFATC3/NFYB/NHLH2/NTF3/PARK2/LEF1/SIRT6/PITX2/PKHD1/PLAGL1/PML/RIPPLY3/RIPK4/TLR9/CYTL1/POMC/POU2AF1/BNC2/MED18/BANP/ELP3/PRMT6/DNAJC17/ZNF532/CNOT11/PRKD1/MYNN/MAPK3/PRMT8/HTRA1/PAK6/ARNTL2/RGMA/PRDM11/TENM2/GATAD2B/CCAR2/CREBZF/TRIM27/NPAS3/NOD2/SFRP2/TRA2B/GZF1/SGK1/BMP4/ZNF649/BMPR1B/BRD9/ZSCAN18/SOX9/STAT2/STK3/SUPT6H/TAF4B/TBP/TCEA1/TCEB2/ZEB1/TEAD3/TLE3/TNFAIP3/TNFRSF1A/TRAF1/TRAF5/TWIST1/TNFRSF4/UCP1/WNT10B/ZNF7/ZNF124/ZNF177/PAX8/FZD5/CARD14/ZNF665/ZC3H14/ZNF606/ZC3H12A/ZNF436/CALR/SLIRP/SCRT1/HIST1H3A/SLA2/ZNF397/NR0B2/HOPX/KDM2B/LOXL3/CBX2/GAS7/RUNX1/TP63/RUNX3/ACTN1/FADD/TNFRSF11A/SPHK1/BUD31/CCNA1/LIMD1/PIAS2/LDB2/CBFA2T2/AURKB/ADIPOQ/H2AFY/MICAL2/VGLL4/ZBTB39/NR1H4	211
GO:0030878	thyroid gland development	5/901	18/17046	0.00196	0.02795	0.02198	FGF10/HOXB3/HOXD3/SMAD3/PAX8	5
GO:0071371	cellular response to gonadotropin stimulus	5/901	18/17046	0.00196	0.02795	0.02198	CYP11A1/EGR3/INHBA/LHCGR/PAX8	5
GO:0072077	renal vesicle morphogenesis	5/901	18/17046	0.00196	0.02795	0.02198	SOX8/FMN1/BMP4/SOX9/PAX8	5
GO:0070848	response to growth factor	65/901	855/17046	0.00197	0.02795	0.02198	CDKN1C/ADCY3/LECT1/CIDEA/CPS1/CTGF/CYP11A1/EGFR/EGR3/FGF10/RASA3/FOXO1/SPG20/NEDD4L/ARHGEF18/MTOR/FGF22/NPTN/BMP10/GRB10/HAS1/NRG1/NR4A1/HTR3A/CYR61/IL1R1/KDR/HES5/LCK/ARHGDIA/LTBP1/SMAD3/MAP3K1/MOV10/NRAS/ARHGEF3/LEF1/PML/PPP1CB/PPP1CC/PRKAR1B/PRKD1/MAPK3/MAP2K2/HTRA1/PSMB4/RGMA/PSMD7/PLEKHG5/PXN/RASGRF2/RIT2/BGLAP/SFRP2/BMP4/BMPR1B/SOX9/ZEB1/TWIST1/TMEM204/RUNX1/RUNX3/IRS2/RAPGEF2/FGF19	65
GO:1903035	negative regulation of response to wounding	17/901	147/17046	0.00197	0.02795	0.02198	NLRP6/F11/FGA/GPER1/GSTP1/ANXA2/ISL1/SMAD3/NOV/IL20RB/PROC/PSMB4/NOD2/TNFAIP3/TNFRSF1A/NLRX1/ADIPOQ	17
GO:0036336	dendritic cell migration	6/901	26/17046	0.00198	0.02795	0.02198	CCR1/LGALS9/PIK3CG/CCR2/CXCR4/CALR	6
GO:0060561	apoptotic process involved in morphogenesis	6/901	26/17046	0.00198	0.02795	0.02198	FOXC2/CYR61/LEF1/PML/PAX8/FZD5	6
GO:0072207	metanephric epithelium development	6/901	26/17046	0.00198	0.02795	0.02198	SOX8/ACAT1/HES5/SOX9/PAX8/ADIPOQ	6
GO:2000826	regulation of heart morphogenesis	6/901	26/17046	0.00198	0.02795	0.02198	FOXC2/BMP10/ISL1/BMP4/SOX9/TWIST1	6
GO:0019538	protein metabolic process	304/901	5009/17046	0.00199	0.02798	0.022	AKT3/ABI1/GNE/TSPAN5/FARP1/CDKN1C/SPEG/BCKDK/PITRM1/CELF1/HCST/NPFFR2/ADCY3/TMED10/LECT1/ADAM29/CHI3L1/ERLIN2/EGLN2/B4GALT7/EXOC3/ADPRHL1/CARD16/ALPK2/GALNT15/CLCA1/CLN5/MRPL52/CCR1/SLC51B/NEU4/COL11A1/MAP3K8/IL31RA/EGFLAM/UBLCP1/CPD/CPM/CPS1/TRPM6/MIB2/PARP4/LDLRAD3/B3GLCT/MGAT5B/CSTA/ZNF738/CTGF/SMYD1/PPM1L/SH3D19/CYLD/ADRB3/ESCO2/DDB1/LONRF2/DDOST/RNF168/NLRP6/DLG2/DPH1/DRD4/ECE1/EEF2/EGFR/PATL2/EIF4G1/A2M/ELK4/ADCK5/EPHA1/EPHA3/EPHB4/F11/PRSS54/RNF182/FGA/FGF10/FHIT/RASA3/PPM1E/TRAK1/MSRB2/FOXL1/FOXO1/GGA3/FLOT2/NUP210/NEDD4L/LARP1/PUM2/RYBP/MORC3/MAPK8IP2/TSSK2/MTOR/RNF144B/ST6GALNAC3/GAK/SAMM50/TENM4/LTN1/FBXL21/FBXO2/LCE2B/SACS/GBGT1/SLC17A5/RPS6KC1/PABPC1/DNAJC2/FGF22/NPTN/VPS4A/BMP10/TMPRSS12/PIGW/GPER1/EOGT/DOK7/MRPS18B/GSTP1/PADI1/GZMA/ANXA2/SERPIND1/NRG1/HSPA1L/HSP90AA1/HSP90AB1/DUPD1/ADAMTSL5/COL28A1/BARHL2/IGF1/IGF2/CYR61/LCE1C/LCE1D/LCE2D/IL1RN/IL6/IL12RB2/PRSS41/INHBA/IRF1/ISL1/ITGB2/ITIH3/ITIH4/IVL/USP50/KCNH2/KDR/HES5/LCK/MUC21/LDLR/LGALS9/LMNA/LOX/LTB/SMAD3/MAP3K1/MFI2/MGAT1/MOCS1/NEU1/NRAS/NTF3/OAS2/OPRL1/PARK2/SPOCK3/LEF1/PRR16/CHST15/ANGPT4/PCYOX1/SIRT6/GALNT7/PI3/PIGC/PIK3CG/PKHD1/PLA2G2A/PRKAG3/PML/RIPK4/TLR9/TREM1/CYTL1/POMC/SSH1/BANP/PPP1CB/HERC6/PPP1CC/PIWIL2/ELP3/PRMT6/PPP2R2B/FANCI/CNOT11/PRKAR1B/CSGALNACT1/PRKD1/WSB2/MAPK3/MAP2K2/PROC/PRMT8/MASP1/HTRA1/PSMB4/PAK6/PSMD7/TENM2/KLHL8/METTL14/MARK4/CCAR2/PTPRE/PXN/RASGRF2/TRIM27/RGR/EXOC4/RPL8/RPL29/CCL11/CCL17/MRPS14/PRSS22/NOD2/SFRP2/SGK1/BMP4/BMPR1B/BOK/SOX9/SRP68/STK3/STK10/SUPT6H/BST2/VAMP2/TCEB2/TERF1/TGM2/TIMP3/TNFAIP3/TNFRSF1A/TNXB/TRAF5/TWIST1/CCR2/TNFRSF4/VARS/WNT10B/YWHAG/ZAP70/PTP4A1/MOGS/CXCR4/FZD5/RAB7A/CARD14/ERMP1/CPEB4/CALR/CAST/HOPX/TTBK1/SPINK7/TRIM63/KDM2B/CBX2/RAE1/CDK10/TP63/RUNX3/SERPINA6/IRS2/CRADD/FADD/TNFRSF11A/STK19/SPHK1/ENDOU/PIAS2/ZFAND2A/MAP3K6/AURKB/NEURL3/ADIPOQ/RAB3D/H2AFY/PREPL/N4BP1/NUP93/RAPGEF2/ULK2/FGF19	304

GO:0010557	positive regulation of macromolecule biosynthetic process	107/901	1544/17046	0.00206	0.0289	0.02273	CDH13/CDKN1C/DMRT2/TBR1/ERLIN2/PSIP1/SLC51B/IL31RA/CTGF/CITED4/BHLHA15/EEF2/EGFR/ESR1/FGF10/SBNO2/FOXC2/FOXO1/LARP1/VGLL2/MTOR/PABPC1/DNAJC2/BMP10/GPER1/BRF1/SOX8/HMGA1/NR4A1/TFAP2E/BARHL2/IGF1/IGF2/CYR61/IL6/FOXK2/INHBA/IRF1/ISL1/JUP/KDR/HES5/LGALS9/LMNA/LMO2/LTB/SMAD3/MEF2D/MEOX1/MEOX2/MITF/NFATC3/NFYB/NHLH2/NTF3/PARK2/LEF1/PRR16/PITX2/PLAGL1/RIPK4/TLR9/CYTL1/POMC/BANP/PIWIL2/PRKD1/MAPK3/ARNTL2/RGMA/TRIM27/NPAS3/NOD2/SFRP2/BMP4/ZNF649/BMPR1B/SOX9/STK3/SUPT6H/TBP/TCEA1/TCEB2/ZEB1/TEAD3/TNFRSF1A/TRAF1/TRAF5/TWIST1/CCR2/WNT10B/PAX8/FZD5/CARD14/CALR/RUNX1/TP63/RUNX3/IRS2/FADD/TNFRSF11A/SPHK1/PIAS2/LDB2/CBFA2T2/MICAL2/NR1H4	107
GO:0050708	regulation of protein secretion	33/901	367/17046	0.00207	0.02893	0.02275	CIDEA/ABAT/DRD4/FGA/EXPH5/GNAS/GPER1/FFAR2/HLA-E/IGF1/IL6/ISL1/LGALS9/LLGL1/NOV/PARK2/PDE4C/PML/TLR9/GOLPH3L/SYBU/PRKAR1B/TRIM27/NOD2/VAMP2/TWIST1/TNFRSF4/CACNA1E/RAB11FIP1/NR0B2/IRS2/SYT7/RSAD2	33
GO:0044710	single-organism metabolic process	303/901	4994/17046	0.00207	0.02893	0.02275	CDH3/GNE/BCKDK/TCIRG1/MTHFS/PDPN/NPFFR2/ADCY3/SLC27A2/HIBADH/CHI3L1/ERLIN2/EGLN2/B4GALT7/ACOT7/CIDEA/GALNT15/CLN5/MRPL52/CCR1/SLC51B/CNP/APOA1BP/NEU4/COL9A3/COL11A1/GALM/MAP3K8/ADM/IL31RA/EGFLAM/HUS1B/CPS1/NDUFAF6/PXDNL/CRABP1/PARP4/B3GLCT/MGAT5B/CTGF/SMYD1/PPM1L/CYB561/MBOAT1/ADRB3/ESCO2/CYP11A1/FITM1/ADAL/DDB1/DDOST/RNF168/NLRP6/DIO3/DLG2/DNMT3A/ABAT/DPH1/DRD4/AGXT/EEF2/EGFR/A2M/ELK4/LIPH/ENO2/ESR1/ALAS1/F11/FAH/FGA/FGF10/FHIT/RASA3/TRAK1/MSRB2/ACIN1/FOXO1/AKR1B1/NUP210/LARP1/RYBP/MAPK8IP2/MTOR/FUCA1/SLC37A4/GABBR1/ST6GALNAC3/PNKD/ACOT11/STEAP2/FBXO2/GATM/GBGT1/GAPDHS/SLC17A5/FGF22/AMPD2/PDE7B/DKK3/GLS2/AMPD3/DHDH/BMP10/GNAS/PIGW/THEM5/GPER1/EOGT/GRB10/MRPS18B/DNAJC15/GSTP1/GUCY1A3/NME7/PADI1/ANXA2/HAS1/NRG1/HK1/HMGA1/ACACB/HPCA/HRH1/HSD11B1/HSD17B2/ACADL/HSP90AA1/NME9/IGF1/IGF2/CYR61/IL1RN/IL6/INHBA/INPP5A/ISL1/USP50/KCNH2/KDR/ACAT1/KRT15/MUC21/LDLR/LGALS9/LHCGR/LOX/SMAD3/MC2R/ME1/ME2/MAP3K1/MGAT1/MOCS1/NUDT1/MYH4/NUBP1/NDUFB4/NEU1/ATP1A2/NFATC3/NFYB/NPPC/NRAS/NTF3/OAS2/OPRL1/PAFAH2/ATP5B/PALM/PARK2/SPOCK3/LEF1/CHST15/PDE4C/PCYOX1/PDE7A/SIRT6/PDE6B/GALNT7/PGAM2/PIGC/PIK3CG/PKHD1/PKM/PLA2G2A/PRKAG3/PML/SLCO1C1/PNLIP/TLR9/TREM1/CYTL1/POMC/PON1/CYP2W1/LPCAT2/PPP1CB/PPP1CC/PIWIL2/ELP3/PRMT6/FANCI/WDR33/SMPD3/VAC14/PRKAR1B/LMBRD1/CSGALNACT1/CISD1/PRKD1/APOBR/MAPK3/MAP2K2/MRAP/PRMT8/MASP1/PSMB4/PAK6/PSMD7/RDH14/PXN/RASGRF2/RFC2/RPA3/SCT/CCL11/CCL17/ABHD4/MRPS14/NOD2/STRA6/SFRP2/MICAL1/CERK/VPS33A/BMP4/SLC4A1/BMPR1B/SOX9/STAT2/STK3/STK10/SUPT6H/VAMP2/TCEA1/TERF1/TNFRSF1A/TNXB/PHLDA2/TWIST1/CCR2/UCP1/UPP1/VARS/WNT10B/CA7/CACNA1E/MOGS/PAX8/CXCR4/FZD5/RAB7A/GDPD3/CERS4/EPHX3/FAAP100/COL18A1/EEPD1/CALR/QTRT1/CAPS/COLQ/SH3BGRL3/NR0B2/HOPX/KDM2B/LOXL3/RAE1/CDK10/KMO/SERPINA6/IRS2/TNFRSF11A/ALDH1A2/SYNJ2/SPHK1/STBD1/CH25H/MAP3K6/SLC16A3/AURKB/SDR42E1/ADIPOQ/H2AFY/ENTPD3/MICAL2/NUP93/RAPGEF2/ULK2/LPGAT1/FGF19/NR1H4	303
GO:0003012	muscle system process	31/901	339/17046	0.00215	0.02996	0.02356	MRVI1/TRDN/CHRNA1/CTGF/DTNA/BMP10/GPER1/TMOD4/GUCY1A3/KCNIP2/ANXA6/IGF1/KCNH2/LCK/MYH4/MYL2/ATP1A2/NFATC3/ATP8A2/PGAM2/PIK3CG/PXN/ACTA2/SLC8A1/ACTC1/CALD1/CASQ1/TRIM63/SPHK1/STBD1/RCSD1	31
GO:0044765	single-organism transport	226/901	3611/17046	0.00217	0.03011	0.02368	ABI1/CDH3/KCNMB2/TCIRG1/TRDN/ABCA9/SPON2/COG5/ADCY3/TMED10/SLC27A2/RER1/CHGA/CHI3L1/EXOC3/CHRNA1/CHRNA2/CHRNA5/CIDEA/PANX3/AP3S1/CLCA1/CCR1/SLC51B/C15orf27/SLC38A10/CNP/ADM/TRPM6/KLC3/CTGF/ABCC13/SH3D19/CYB561/CYLD/TRPV3/DDOST/BHLHA15/NLRP6/ABAT/DRD4/AGXT/EGFR/A2M/UNC13D/SLC10A4/FCGR2A/FGA/FGF10/RASA3/TRAK1/EXPH5/NFASC/MLC1/NUP210/ATP11A/NEDD4L/MAPK8IP2/MTOR/SLC37A4/SAMM50/STEAP2/SLC17A5/GJA3/GJB2/GLS2/VPS4A/GNAS/CRACR2B/GPR26/GPER1/FFAR2/GRB10/FLVCR1/GRIK4/DNAJC15/SCG3/NME7/ANXA2/KCNIP2/NRG1/ANXA6/HK1/ANXA13/HLA-E/ACACB/HPCA/HSPA1L/HSP90AA1/HSP90AB1/HTR3A/IGF1/IGF2/IL1RN/IL6/AQP2/AQP5/INHBA/AQP9/ISL1/JUP/ATP9B/KCNH2/KCNJ8/KCNJ9/KCNMB1/IPO5/TOMM20L/SLC6A17/LCK/LDLR/LGALS9/LLGL1/LMNA/SMAD3/MFI2/ATP1A2/NFATC3/NOV/NTF3/OPRL1/SLC22A18/P2RY6/ATP5B/ANO7/PALM/PARK2/PDE4C/PCYOX1/C11orf73/SIRT6/ATP8A2/PIK3CG/PRKAG3/PML/FXYD6/SLCO1C1/PNLIP/TLR9/TREM1/POMC/PON1/ZDHHC13/GOLPH3L/SLC47A1/SLC29A3/TRPV6/SMPD3/SLC30A10/CHRNA9/SYBU/PEX26/PRKAR1B/IFT122/LMBRD1/PRKD1/APOBR/MAPK3/MAP2K2/TRPV5/CDC42SE1/TRPC7/RASGRF2/TRIM27/EXOC4/RPL8/RPL29/S100A6/SCT/NOD2/TINAGL1/STRA6/SFRP2/SGK1/VPS33A/BMP4/SLC4A1/SLC6A12/SLC8A1/SLC9A3/SLC20A2/SRP68/SUPT6H/VAMP2/TGM2/TRAPPC10/TRPC4/TRPC6/TRPM2/TWIST1/CCR2/TNFRSF4/UCP1/YWHAG/CA7/CACNA1E/CACNB2/PAX8/FZD5/RAB7A/RAB11FIP1/CALR/SLC25A18/MFSD7/ATP13A4/NR0B2/MON1A/CASQ1/MGARP/RAE1/SLC43A1/SCIN/IRS2/ACTN1/TNFRSF11A/SPHK1/SYT7/SLC16A3/RSAD2/SMDT1/ADIPOQ/RAB3D/NUP93/USP6NL/FGF19/NR1H4	226
GO:0042246	tissue regeneration	9/901	55/17046	0.0022	0.03044	0.02394	FGF10/GATM/IGF1/NOV/PKM/BIN3/TIMP3/WNT10B/HOPX	9
GO:0016051	carbohydrate biosynthetic process	20/901	188/17046	0.0023	0.03177	0.02499	ENO2/FOXO1/AKR1B1/MTOR/GAPDHS/GPER1/GRB10/HAS1/HRH1/IGF1/IGF2/IL6/LHCGR/CHST15/PGAM2/PRKAG3/PPP1CB/CSGALNACT1/IRS2/ADIPOQ	20
GO:0045944	positive regulation of transcription from RNA polymerase II promoter	72/901	973/17046	0.00231	0.03186	0.02506	CDH13/DMRT2/TBR1/ERLIN2/PSIP1/BHLHA15/EGFR/ESR1/FGF10/SBNO2/FOXC2/FOXO1/VGLL2/GPER1/SOX8/HMGA1/NR4A1/TFAP2E/BARHL2/IGF1/IGF2/CYR61/IL6/FOXK2/INHBA/IRF1/ISL1/HES5/LMNA/LMO2/SMAD3/MEF2D/MEOX1/MEOX2/MITF/NFATC3/NHLH2/NTF3/PARK2/LEF1/PITX2/PLAGL1/TLR9/CYTL1/POMC/PRKD1/MAPK3/ARNTL2/RGMA/NOD2/SFRP2/BMP4/ZNF649/BMPR1B/SOX9/SUPT6H/TCEA1/ZEB1/TEAD3/TNFRSF1A/TWIST1/WNT10B/PAX8/FZD5/RUNX1/TP63/FADD/PIAS2/LDB2/CBFA2T2/MICAL2/NR1H4	72
GO:0042476	odontogenesis	14/901	112/17046	0.00232	0.03191	0.02509	ADM/FGF10/ID3/RSPO2/AQP5/INHBA/LHX8/LEF1/PITX2/HTRA1/BGLAP/BMP4/TWIST1/TP63	14
GO:0048705	skeletal system morphogenesis	22/901	215/17046	0.00234	0.03211	0.02525	COL11A1/COMP/FAM101A/CTGF/FOXC2/GNAS/FLVCR1/HOXB3/HOXD3/FMN1/SMAD3/MEF2D/NPPC/BNC2/CSGALNACT1/SFRP2/BMP4/BMPR1B/SOX9/ZEB1/TWIST1/WNT10B	22

GO:2001141	regulation of RNA biosynthetic process	205/901	3244/17046	0.00235	0.03218	0.02531	ZNF783/CDH13/CDKN1C/C1D/ZBTB18/DMRT2/TBR1/PNRC1/HNRNPUL1/ERLIN2/PSIP1/EGLN2/ZBED9/CIDEA/ZFP42/IL31RA/ZNF358/ZNF738/SMYD1/CYLD/ZNF782/ZNF709/ZNF781/CITED4/RNF168/ZNF366/BHLHA15/DNMT3A/EGFR/EGR3/ELK4/ESR1/SP8/FGF10/FHIT/XRN2/SBNO2/TRAK1/MSRB2/FOXL1/FOXC2/FOXO1/SPG20/NEDD4L/RYBP/VGLL2/MTOR/ZNF549/DNAJC2/DKK3/BMP10/ZNF638/ZNF311/ZNF844/GPER1/ZBTB44/GTF2B/BRF1/SOX8/NRG1/HLX/HMGA1/NR4A1/HOXB3/HOXC4/HOXC5/HOXC6/HOXD3/TFAP2E/ID3/BARHL2/IGF1/IGF2/CYR61/IL6/IL16/FOXK2/INHBA/IRF1/ISL1/JUP/USP50/HILS1/HES5/AFF3/LGALS9/LMNA/LMO2/SMAD3/MEF2D/MEOX1/MEOX2/MITF/LHX8/MOV10/NFATC3/NFYB/NHLH2/NTF3/PARK2/LEF1/SIRT6/PITX2/PKHD1/PLAGL1/PML/RIPPLY3/RIPK4/TLR9/CYTL1/POMC/POU2AF1/BNC2/MED18/BANP/ELP3/PRMT6/DNAJC17/ZNF532/CNOT11/PRKD1/MYNN/MAPK3/PRMT8/HTRA1/PAK6/ARNTL2/RGMA/PRDM11/TENM2/GATAD2B/CCAR2/CREBZF/TRIM27/NPAS3/NOD2/SFRP2/GZF1/SGK1/BMP4/ZNF649/BMPR1B/BRD9/ZSCAN18/SOX9/STAT2/STK3/SUPT6H/TAF4B/TBP/TCEA1/TCEB2/ZEB1/TEAD3/TLE3/TNFAIP3/TNFRSF1A/TRAF1/TRAF5/TWIST1/TNFRSF4/UCP1/WNT10B/ZNF7/ZNF124/ZNF177/PAX8/FZD5/CARD14/ZNF665/ZNF606/ZC3H12A/ZNF436/CALR/SLIRP/SCRT1/HIST1H3A/SLA2/ZNF397/NR0B2/HOPX/KDM2B/LOXL3/CBX2/GAS7/RUNX1/TP63/RUNX3/ACTN1/FADD/TNFRSF11A/SPHK1/BUD31/CCNA1/LIMD1/PIAS2/LDB2/CBFA2T2/AURKB/ADIPOQ/H2AFY/MICAL2/VGLL4/ZBTB39/NR1H4	205
GO:0051246	regulation of protein metabolic process	156/901	2387/17046	0.00238	0.03251	0.02557	ABI1/FARP1/CDKN1C/CELF1/NPFFR2/ADCY3/TMED10/CHI3L1/EGLN2/CARD16/CCR1/SLC51B/MAP3K8/IL31RA/LDLRAD3/CSTA/CTGF/SH3D19/ADRB3/NLRP6/DLG2/DRD4/ECE1/EEF2/EGFR/PATL2/EIF4G1/A2M/EPHA1/FGA/FGF10/FHIT/RASA3/PPM1E/FOXO1/GGA3/FLOT2/NEDD4L/LARP1/PUM2/MAPK8IP2/MTOR/RNF144B/FBXO2/PABPC1/FGF22/NPTN/BMP10/GPER1/DOK7/GSTP1/ANXA2/SERPIND1/NRG1/HSP90AB1/COL28A1/BARHL2/IGF1/IGF2/CYR61/IL1RN/IL6/INHBA/IRF1/ISL1/ITGB2/ITIH3/ITIH4/USP50/KDR/HES5/LCK/LGALS9/LTB/SMAD3/MAP3K1/MFI2/NRAS/NTF3/OPRL1/PARK2/SPOCK3/LEF1/PRR16/ANGPT4/PI3/PIK3CG/PKHD1/PLA2G2A/PML/TLR9/SSH1/BANP/PIWIL2/ELP3/PPP2R2B/FANCI/CNOT11/PRKAR1B/PRKD1/MAPK3/MAP2K2/MASP1/PSMB4/PAK6/PSMD7/METTL14/CCAR2/PXN/RASGRF2/TRIM27/CCL11/CCL17/NOD2/SFRP2/BMP4/BOK/SOX9/STK3/STK10/SUPT6H/BST2/TERF1/TIMP3/TNFAIP3/TNFRSF1A/TNXB/TWIST1/CCR2/TNFRSF4/VARS/WNT10B/YWHAG/CXCR4/FZD5/RAB7A/CARD14/CPEB4/CALR/CAST/SPINK7/CDK10/TP63/SERPINA6/IRS2/CRADD/FADD/TNFRSF11A/SPHK1/ZFAND2A/MAP3K6/ADIPOQ/H2AFY/N4BP1/RAPGEF2/FGF19	156
GO:0021700	developmental maturation	23/901	229/17046	0.0024	0.03269	0.02571	CDH3/CDKN1C/CLN5/SEZ6/SCLT1/FAM101A/BHLHA15/NFASC/FLVCR1/SOX8/KCNIP2/IGF1/KDR/HES5/NPPC/PALM/CEND1/WNT10B/FZD5/BFSP2/RUNX1/RUNX3/ALDH1A2	23
GO:0048871	multicellular organismal homeostasis	29/901	313/17046	0.00243	0.03302	0.02597	CDH3/ADCY3/CIDEA/ZG16B/CTGF/ADRB3/ABAT/FOXO1/AKR1B1/DFNB31/AMPD2/AMPD3/GNAS/ACADL/IL1RN/AQP2/KDR/IL20RB/TLR9/CYTL1/PRKAR1B/BGLAP/NOD2/SOX9/TNFAIP3/RAB7A/TP63/TNFRSF11A/LDB2	29
GO:0001659	temperature homeostasis	7/901	36/17046	0.00245	0.03319	0.0261	CIDEA/ADRB3/ABAT/FOXO1/ACADL/IL1RN/TNFRSF11A	7
GO:0050731	positive regulation of peptidyl-tyrosine phosphorylation	17/901	150/17046	0.00245	0.03319	0.0261	ABI1/IL31RA/FGF10/MTOR/DOK7/NRG1/IGF1/IGF2/IL6/ISL1/HES5/LCK/NTF3/ANGPT4/NOD2/TNFRSF1A/ADIPOQ	17
GO:1901576	organic substance biosynthetic process	331/901	5519/17046	0.00246	0.0332	0.02611	CDH3/GNE/ZNF783/CDH13/CDKN1C/TCIRG1/C1D/ZBTB18/MTHFS/DMRT2/CELF1/TBR1/NPFFR2/ADCY3/PNRC1/SLC27A2/HNRNPUL1/ERLIN2/PSIP1/EGLN2/B4GALT7/ACOT7/ZBED9/CIDEA/GALNT15/MRPL52/SLC51B/NEU4/ZFP42/ADM/IL31RA/CPS1/ZNF358/PARP4/B3GLCT/MGAT5B/ZNF738/CTGF/SMYD1/PPM1L/CYLD/MBOAT1/ADRB3/ESCO2/CYP11A1/ZNF782/FITM1/ADAL/ZNF709/ZNF781/CITED4/DDB1/DDOST/RNF168/ZNF366/BHLHA15/DNMT3A/ABAT/DRD4/AGXT/EEF2/EGFR/EGR3/PATL2/EIF4G1/ELK4/ENO2/ESR1/ALAS1/SP8/FGF10/FHIT/XRN2/SBNO2/TRAK1/MSRB2/FOXL1/FOXC2/FOXO1/AKR1B1/NUP210/NEDD4L/LARP1/PUM2/RYBP/VGLL2/MTOR/GABBR1/ZNF549/ST6GALNAC3/GATM/GBGT1/GAPDHS/SLC17A5/PABPC1/DNAJC2/AMPD2/DKK3/GLS2/AMPD3/BMP10/ZNF638/GNAS/ZNF311/PIGW/ZNF844/GPER1/EOGT/GRB10/MRPS18B/ZBTB44/GSTP1/GTF2B/BRF1/GUCY1A3/NME7/PADI1/HAS1/SOX8/NRG1/HLX/HMGA1/NR4A1/ACACB/HPCA/HOXB3/HOXC4/HOXC5/HOXC6/HOXD3/HRH1/HSD11B1/HSD17B2/ACADL/TFAP2E/ID3/BARHL2/NME9/IGF1/IGF2/CYR61/IL6/IL16/FOXK2/INHBA/IRF1/ISL1/JUP/USP50/HILS1/KDR/ACAT1/HES5/AFF3/MUC21/LDLR/LGALS9/LHCGR/LMO2/LTB/SMAD3/MC2R/ME1/MEF2D/MEOX1/MEOX2/MGAT1/MITF/LHX8/MOCS1/MOV10/DRG1/NEU1/NFATC3/NFYB/NHLH2/NPPC/NTF3/OAS2/OPRL1/ATP5B/PALM/PARK2/LEF1/PRR16/CHST15/SIRT6/GALNT7/PGAM2/PIGC/PIK3CG/PITX2/PKHD1/PKM/PLA2G2A/PLAGL1/PRKAG3/PML/RIPPLY3/RIPK4/TLR9/CYTL1/POMC/POU2AF1/BNC2/MED18/LPCAT2/BANP/PPP1CB/PIWIL2/ELP3/PRMT6/DNAJC17/ZNF532/CNOT11/VAC14/CSGALNACT1/PRKD1/MYNN/MAPK3/MRAP/PRMT8/PAK6/ARNTL2/RGMA/PRDM11/TENM2/GATAD2B/METTL14/CCAR2/CREBZF/RFC2/TRIM27/RGS12/RPA3/RPL8/RPL29/SCT/MRPS14/NPAS3/NOD2/SFRP2/GZF1/SGK1/BMP4/ZNF649/BMPR1B/BRD9/ZSCAN18/SOX9/SRP68/STAT2/STK3/SUPT6H/TAF4B/TBP/TCEA1/TCEB2/ZEB1/TEAD3/TERF1/TLE3/TNFAIP3/TNFRSF1A/TRAF1/TRAF5/TWIST1/CCR2/TNFRSF4/UCP1/UPP1/VARS/WNT10B/YWHAG/ZNF7/ZNF124/ZNF177/MOGS/PAX8/FZD5/CARD14/CERS4/ZNF665/ZNF606/ZC3H12A/CPEB4/ZNF436/CALR/QTRT1/SLIRP/SCRT1/HIST1H3A/SLA2/ZNF397/NR0B2/HOPX/KDM2B/LOXL3/CBX2/RAE1/GAS7/KMO/RUNX1/TP63/RUNX3/IRS2/ACTN1/FADD/TNFRSF11A/ALDH1A2/SYNJ2/SPHK1/BUD31/CCNA1/LIMD1/CH25H/PIAS2/LDB2/CBFA2T2/AURKB/SDR42E1/ADIPOQ/H2AFY/MICAL2/VGLL4/NUP93/RAPGEF2/ZBTB39/LPGAT1/FGF19/NR1H4	331
GO:1903530	regulation of secretion by cell	47/901	581/17046	0.00247	0.03332	0.0262	CHGA/CIDEA/ABAT/DRD4/UNC13D/FGA/EXPH5/VPS4A/GNAS/GPER1/FFAR2/HLA-E/IGF1/IL1RN/IL6/INHBA/ISL1/LGALS9/LLGL1/NOV/PARK2/PDE4C/PML/TLR9/POMC/GOLPH3L/TRPV6/SMPD3/SYBU/PRKAR1B/TRIM27/SCT/NOD2/VAMP2/TWIST1/CCR2/TNFRSF4/CACNA1E/PAX8/RAB7A/RAB11FIP1/NR0B2/IRS2/SYT7/RSAD2/ADIPOQ/RAB3D	47
GO:0060538	skeletal muscle organ development	18/901	163/17046	0.00248	0.03332	0.0262	ZBTB18/CHRNA1/SMYD1/FLNB/VGLL2/SOX8/HLX/MEF2D/MEOX2/NFATC3/NRAS/PITX2/PLAGL1/BIN3/STRA6/TWIST1/WNT10B/CASQ1	18
GO:0055006	cardiac cell development	9/901	56/17046	0.0025	0.03351	0.02635	SPEG/MTOR/BMP10/LMNA/MYL2/PITX2/BMP4/SLC8A1/ACTC1	9

GO:0045935	positive regulation of nucleobase-containing compound metabolic process	110/901	1604/17046	0.00251	0.03362	0.02644	CDH13/CDKN1C/DMRT2/TBR1/ERLIN2/PSIP1/ADM/IL31RA/ADRB3/CITED4/RNF168/BHLHA15/EGFR/ESR1/FGF10/SBNO2/FOXC2/FOXO1/VGLL2/MTOR/GAPDHS/PABPC1/DNAJC2/BMP10/GNAS/GPER1/BRF1/GUCY1A3/SOX8/HMGA1/NR4A1/HPCA/TFAP2E/BARHL2/IGF1/IGF2/CYR61/IL6/FOXK2/INHBA/IRF1/ISL1/JUP/HES5/LGALS9/LHCGR/LMNA/LMO2/SMAD3/MC2R/MEF2D/MEOX1/MEOX2/MITF/NFATC3/NFYB/NHLH2/NPPC/NTF3/PARK2/LEF1/PITX2/PLAGL1/RIPK4/TLR9/CYTL1/POMC/BANP/PRKD1/MAPK3/MRAP/ARNTL2/RGMA/TRIM27/SCT/NPAS3/NOD2/SFRP2/TRA2B/BMP4/ZNF649/BMPR1B/SOX9/STK3/SUPT6H/TBP/TCEA1/TCEB2/ZEB1/TEAD3/TNFRSF1A/TRAF1/TRAF5/TWIST1/WNT10B/PAX8/FZD5/CARD14/CALR/RUNX1/TP63/RUNX3/FADD/TNFRSF11A/SPHK1/PIAS2/LDB2/CBFA2T2/MICAL2/NR1H4	110
GO:0042303	molting cycle	13/901	101/17046	0.00252	0.03362	0.02644	CDH3/APCDD1/TRPV3/EGFR/FGF10/GNAS/INHBA/SOX9/WNT10B/RUNX1/TP63/RUNX3/LDB2	13
GO:0042633	hair cycle	13/901	101/17046	0.00252	0.03362	0.02644	CDH3/APCDD1/TRPV3/EGFR/FGF10/GNAS/INHBA/SOX9/WNT10B/RUNX1/TP63/RUNX3/LDB2	13
GO:1901342	regulation of vasculature development	21/901	203/17046	0.00255	0.03362	0.02644	LECT1/CHI3L1/ADM/EPHA1/VASH1/FOXC2/IL6/ISL1/KDR/ANGPT4/PML/PRKD1/CCL11/SFRP2/BMP4/TNFAIP3/TWIST1/CCR2/RUNX1/SPHK1/RAPGEF2	21
GO:0072087	renal vesicle development	5/901	19/17046	0.00255	0.03362	0.02644	SOX8/FMN1/BMP4/SOX9/PAX8	5
GO:0006366	transcription from RNA polymerase II promoter	124/901	1842/17046	0.00257	0.03362	0.02644	ZNF783/CDH13/CDKN1C/ZBTB18/DMRT2/TBR1/ERLIN2/PSIP1/EGLN2/CTGF/RNF168/ZNF366/BHLHA15/DNMT3A/EGFR/ELK4/ESR1/FGF10/SBNO2/TRAK1/FOXL1/FOXC2/FOXO1/NEDD4L/RYBP/VGLL2/MTOR/GLS2/BMP10/GPER1/GTF2B/SOX8/HMGA1/NR4A1/HOXB3/HOXC5/HOXC6/TFAP2E/ID3/BARHL2/IGF1/IGF2/CYR61/IL6/FOXK2/INHBA/IRF1/ISL1/HES5/LMO2/SMAD3/MEF2D/MEOX1/MEOX2/MITF/NFATC3/NHLH2/NTF3/PARK2/LEF1/PITX2/PLAGL1/PRKAG3/RIPPLY3/TLR9/CYTL1/POMC/POU2AF1/MED18/ELP3/PRMT6/DNAJC17/PRKD1/MAPK3/ARNTL2/RGMA/TENM2/GATAD2B/TRIM27/NPAS3/NOD2/SFRP2/GZF1/BMP4/ZNF649/BMPR1B/ZSCAN18/SOX9/STAT2/SUPT6H/TAF4B/TBP/TCEA1/TCEB2/ZEB1/TEAD3/TNFRSF1A/TWIST1/UCP1/WNT10B/YWHAG/ZNF177/PAX8/FZD5/CALR/SCRT1/SLA2/NR0B2/HOPX/KDM2B/CBX2/RUNX1/TP63/RUNX3/FADD/BUD31/CCNA1/PIAS2/LDB2/CBFA2T2/AURKB/H2AFY/MICAL2/NR1H4	124
GO:0072507	divalent inorganic cation homeostasis	33/901	372/17046	0.00257	0.03362	0.02644	TRDN/CCR1/ADM/DRD4/ESR1/NPTN/GPER1/ANXA6/KDR/LCK/ATP1A2/OPRL1/PARK2/PDE6B/PIK3CG/PKHD1/PML/SLC30A10/CHRNA9/PRKD1/TRPC7/CCL11/BMP4/SLC8A1/TGM2/TRPC4/TRPC6/CCR2/CXCR4/CALR/ATP13A4/CASQ1/SMDT1	33
GO:0050848	regulation of calcium-mediated signaling	8/901	46/17046	0.00257	0.03362	0.02644	CDH13/RCAN2/DRD4/NRG1/IGF1/ZAP70/SLA2/CD8A	8
GO:0048699	generation of neurons	102/901	1471/17046	0.00257	0.03362	0.02644	FARP1/CDKN1C/SPON2/ZBTB18/TBR1/GPRIN1/CLN5/SEZ6/CNP/COL9A3/SCLT1/ADM/DNMT3A/EFNA2/EGFR/EIF4G1/EML1/EPHA1/EPHA3/EPHB4/FGF10/RASA3/BTBD3/SPG20/NFASC/EPB41L3/NEDD4L/PSD3/MAPK8IP2/DFNB31/TENM4/FGF22/NPTN/GPER1/SOX8/KCNIP2/NRG1/HOXB3/HOXD3/HSP90AA1/HSP90AB1/ID3/RSPO2/FMN1/BARHL2/IL6/INHBA/ISL1/HES5/STMN1/ARHGDIA/LLGL1/LHX8/NEU1/NRAS/NTF3/PALM/PARK2/LEF1/CEND1/ATP8A2/PITX2/SSH1/PPP1CC/ELP3/PRKD1/MAPK3/MAP2K2/PSMB4/RGMA/TRPC7/PSMD7/TENM2/RASGRF2/S100A6/SGK1/BMP4/BMPR1B/SLIT1/SOX9/ZEB1/TRPC4/TRPC6/TWIST1/WNT10B/YWHAG/CACNB2/CXCR4/FZD5/LST1/CALR/SCRT1/PARD6B/GAS7/RUNX1/RUNX3/IRS2/ALDH1A2/CBFA2T2/RAPGEF2/ULK2/FGF19	102
GO:0071363	cellular response to growth factor stimulus	63/901	833/17046	0.00259	0.03362	0.02644	CDKN1C/ADCY3/LECT1/CIDEA/CPS1/CTGF/CYP11A1/EGFR/EGR3/FGF10/RASA3/FOXO1/SPG20/NEDD4L/ARHGEF18/MTOR/FGF22/NPTN/BMP10/GRB10/HAS1/NRG1/NR4A1/HTR3A/CYR61/KDR/HES5/LCK/ARHGDIA/LTBP1/SMAD3/MAP3K1/MOV10/NRAS/ARHGEF3/LEF1/PML/PPP1CB/PPP1CC/PRKAR1B/PRKD1/MAPK3/MAP2K2/HTRA1/PSMB4/RGMA/PSMD7/PLEKHG5/PXN/RASGRF2/RIT2/BGLAP/SFRP2/BMP4/BMPR1B/SOX9/ZEB1/TWIST1/TMEM204/RUNX1/IRS2/RAPGEF2/FGF19	63
GO:0000738	DNA catabolic process, exonucleolytic	3/901	6/17046	0.00261	0.03362	0.02644	XRN2/ERI3/ERI1	3
GO:0009838	abscission	3/901	6/17046	0.00261	0.03362	0.02644	SPG20/VPS4A/AURKB	3
GO:0032960	regulation of inositol trisphosphate biosynthetic process	3/901	6/17046	0.00261	0.03362	0.02644	GPER1/HRH1/LHCGR	3
GO:0043031	negative regulation of macrophage activation	3/901	6/17046	0.00261	0.03362	0.02644	IL31RA/BPI/ZC3H12A	3
GO:0060019	radial glial cell differentiation	3/901	6/17046	0.00261	0.03362	0.02644	FGF10/HES5/LEF1	3

GO:0072049	comma-shaped body morphogenesis	3/901	6/17046	0.00261	0.03362	0.02644	HES5/BMP4/PAX8	3
GO:0072174	metanephric tubule formation	3/901	6/17046	0.00261	0.03362	0.02644	SOX8/SOX9/PAX8	3
GO:1902031	regulation of NADP metabolic process	3/901	6/17046	0.00261	0.03362	0.02644	ME1/ME2/PGAM2	3
GO:0045087	innate immune response	73/901	994/17046	0.00262	0.03362	0.02644	ABI1/TANK/KLRG1/SPON2/ADCY3/CHGA/MAP3K8/CYLD/DDOST/COCH/DMBT1/EGFR/A2M/UNC13D/FCGR2A/FGA/FGF10/RASA3/FOXO1/MTOR/FGF22/GPER1/FFAR2/NRG1/HLA-B/HLA-DPA1/HLA-E/HLA-F/NR4A1/HSP90AA1/HSP90AB1/CD300E/IRF1/ITGB2/LCK/LGALS9/MAP3K1/MOV10/NFATC3/NRAS/OAS2/PIK3CG/PML/TLR9/TREM1/PRKAR1B/PRKD1/MAPK3/MAP2K2/MASP1/PSMB4/PSMD7/RASGRF2/TRIM27/DEFB134/CCL11/CCL17/NOD2/STAT2/BST2/TLR5/TNFAIP3/CCR2/ZAP70/NLRX1/UNC93B1/IFITM1/IRS2/FADD/RSAD2/LY86/RAPGEF2/FGF19	73
GO:0050790	regulation of catalytic activity	142/901	2151/17046	0.00262	0.03362	0.02644	ABI1/CDH3/FARP1/RCAN2/CDKN1C/PITRM1/NPFFR2/ADCY3/CHI3L1/CARD16/MAP3K8/CSTA/CTGF/ADRB3/DLG2/DRD4/EGFR/A2M/EPHA1/EPHA3/ESR1/SPATA13/PHACTR1/FGF10/RASA3/PPM1E/TBC1D9B/TBC1D1/PSD3/ARHGEF18/MAPK8IP2/MTOR/GABBR1/RASGEF1C/ALS2CL/RGS22/FGF22/CYTH4/GNAS/GPER1/DOK7/DNAJC15/GSTP1/GZMA/ANXA2/SERPIND1/NRG1/HPCA/AGFG2/HSP90AA1/HSP90AB1/COL28A1/IGF2/CYR61/IL1RN/IL6/ITIH3/ITIH4/IPO5/LCK/ARHGDIA/LGALS9/LHCGR/LLGL1/SMAD3/MAP3K1/PLEKHG7/NRAS/NTF3/PALM/ARHGEF3/PARK2/SPOCK3/LEF1/ANGPT4/PI3/PIK3CG/PML/PNLIP/TLR9/RIN2/ELP3/ARHGEF10L/PPP2R2B/VAC14/PRKAR1B/PRKD1/MAPK3/MAP2K2/SLAMF8/CDC42SE1/PSMB4/PAK6/PSMD7/PLEKHG5/CCAR2/PXN/RASGRF2/TRIM27/RGS12/CCL11/CCL17/NOD2/SFRP2/ARHGAP9/SGK1/BMP4/BOK/STK3/STK10/BST2/TCEA1/TERF1/TIMP3/TNFAIP3/TNXB/CCR2/TNFRSF4/YWHAG/CXCR4/FZD5/CARD14/CAST/SH3BGRL3/SPINK7/TP63/SERPINA6/IRS2/CRADD/FADD/TNFRSF11A/STARD13/MAP3K6/ADIPOQ/ARHGAP29/H2AFY/ARHGEF10/RAPGEF2/USP6NL/RABGAP1L/IQSEC1/FGF19	142
GO:0050866	negative regulation of cell activation	17/901	151/17046	0.00263	0.03362	0.02644	IL31RA/CYLD/GPER1/HLX/ZC3H12D/INHBA/IRF1/LGALS9/IL20RB/PAG1/BMP4/BPI/TNFAIP3/CCR2/LST1/ZC3H12A/SLA2	17
GO:0055123	digestive system development	17/901	151/17046	0.00263	0.03362	0.02644	CDKN1C/CPS1/EGFR/FGF10/FOXL1/HLX/IGF1/IGF2/SMAD3/PITX2/SCT/STRA6/SFRP2/BMP4/PPDPF/TP63/ALDH1A2	17
GO:0032675	regulation of interleukin-6 production	12/901	90/17046	0.0027	0.0345	0.02713	SPON2/HLA-B/IL6/ISL1/LGALS9/TLR9/NOD2/BPI/TNFAIP3/TWIST1/NLRX1/ZC3H12A	12
GO:0046689	response to mercury ion	4/901	12/17046	0.00273	0.03465	0.02725	GATM/AQP2/AQP9/PGAM2	4
GO:0060439	trachea morphogenesis	4/901	12/17046	0.00273	0.03465	0.02725	RSPO2/MAPK3/MAP2K2/BMP4	4
GO:0051051	negative regulation of transport	36/901	418/17046	0.00277	0.03518	0.02766	TRDN/CHGA/CIDEA/CYLD/DRD4/NEDD4L/MTOR/GRB10/ANXA2/NRG1/ANXA13/IL1RN/IL6/INHBA/KCNH2/LGALS9/ATP1A2/NOV/OPRL1/PARK2/PDE4C/SIRT6/PML/TLR9/LMBRD1/PRKD1/TRIM27/SCT/BST2/TWIST1/CCR2/TNFRSF4/RAB11FIP1/IRS2/RSAD2/ADIPOQ	36
GO:1901653	cellular response to peptide	40/901	478/17046	0.0028	0.03536	0.02781	TCIRG1/ADCY3/AP3S1/CPS1/CYP11A1/EGFR/EIF4G1/FGF10/RASA3/FOXC2/FOXO1/AKR1B1/MTOR/FGF22/GNAS/GPER1/GRB10/NRG1/NR4A1/IGF2/LCK/NRAS/PRKAG3/TLR9/PRKAR1B/LMBRD1/MAPK3/MAP2K2/PSMB4/PSMD7/PTPRE/PXN/RASGRF2/NOD2/VAMP2/MGARP/IRS2/ADIPOQ/RAPGEF2/FGF19	40
GO:0051048	negative regulation of secretion	19/901	178/17046	0.00281	0.03536	0.02781	CHGA/CIDEA/DRD4/NRG1/IL1RN/IL6/INHBA/LGALS9/NOV/PARK2/PDE4C/PML/TRIM27/SCT/CCR2/TNFRSF4/RAB11FIP1/RSAD2/ADIPOQ	19
GO:0042060	wound healing	53/901	678/17046	0.00281	0.03536	0.02781	CDH3/KCNMB2/MRVI1/NLRP6/A2M/F11/FGA/FGF10/FOXC2/GATM/GNAS/SCG3/GUCY1A3/ANXA2/SERPIND1/NRG1/IGF1/IGF2/CYR61/IL6/IRF1/ITGB2/KCNMB1/LCK/LOX/SMAD3/MAP3K1/NOV/NRAS/ANGPT4/PIK3CG/PKM/TREM1/APBB1IP/PRKAR1B/BIN3/MAPK3/PROC/TRPC7/BMP4/SLC8A1/TIMP3/TNFAIP3/TRPC6/WNT10B/C6orf25/CAPZB/HIST1H3A/HOPX/ACTN1/SYT7/ESAM/SLC16A3	53
GO:0055074	calcium ion homeostasis	31/901	345/17046	0.00281	0.03536	0.02781	TRDN/CCR1/ADM/DRD4/ESR1/NPTN/GPER1/ANXA6/KDR/LCK/ATP1A2/OPRL1/PDE6B/PIK3CG/PKHD1/PML/CHRNA9/PRKD1/TRPC7/CCL11/BMP4/SLC8A1/TGM2/TRPC4/TRPC6/CCR2/CXCR4/CALR/ATP13A4/CASQ1/SMDT1	31
GO:0008154	actin polymerization or depolymerization	17/901	152/17046	0.00282	0.03536	0.02781	ABI1/MSRB2/MTOR/TMOD4/FMN1/MAP3K1/SSH1/TTC17/ACTR3B/TRIM27/CCL11/MICAL1/CAPZB/SH3BGRL3/GAS7/SCIN/MICAL2	17

GO:1901652	response to peptide	47/901	585/17046	0.00283	0.03538	0.02782	TCIRG1/NPFFR2/ADCY3/AP3S1/ADM/CPS1/CTGF/CYP11A1/EGFR/EIF4G1/FGF10/RASA3/FOXC2/FOXO1/AKR1B1/MTOR/GATM/FGF22/GNAS/GPER1/GRB10/NRG1/NR4A1/IGF2/IL6/LCK/NRAS/PKM/PRKAG3/TLR9/PRKAR1B/LMBRD1/MAPK3/MAP2K2/PSMB4/PSMD7/PTPRE/PXN/RASGRF2/NOD2/VAMP2/TNFAIP3/MGARP/IRS2/ADIPOQ/RAPGEF2/FGF19	47
GO:0034250	positive regulation of cellular amide metabolic process	11/901	79/17046	0.00284	0.03548	0.0279	EEF2/LARP1/MTOR/PABPC1/BARHL2/CYR61/IL6/PRR16/PIWIL2/MAPK3/NOD2	11
GO:0030182	neuron differentiation	95/901	1359/17046	0.00285	0.0355	0.02791	FARP1/CDKN1C/SPON2/ZBTB18/TBR1/GPRIN1/CLN5/SEZ6/CNP/COL9A3/SCLT1/ADM/DNMT3A/EFNA2/EGFR/EIF4G1/EPHA1/EPHA3/EPHB4/FGF10/RASA3/BTBD3/SPG20/NFASC/EPB41L3/NEDD4L/PSD3/MAPK8IP2/DFNB31/TENM4/FGF22/NPTN/SOX8/KCNIP2/NRG1/HOXD3/HSP90AA1/HSP90AB1/ID3/RSPO2/FMN1/BARHL2/IL6/INHBA/ISL1/HES5/STMN1/ARHGDIA/LLGL1/LHX8/NEU1/NRAS/NTF3/PALM/PARK2/LEF1/CEND1/ATP8A2/PITX2/SSH1/PPP1CC/PRKD1/MAPK3/MAP2K2/PSMB4/RGMA/TRPC7/PSMD7/TENM2/RASGRF2/S100A6/SGK1/BMP4/BMPR1B/SLIT1/SOX9/ZEB1/TRPC4/TRPC6/WNT10B/YWHAG/CACNB2/FZD5/LST1/CALR/PARD6B/GAS7/RUNX1/RUNX3/IRS2/ALDH1A2/CBFA2T2/RAPGEF2/ULK2/FGF19	95
GO:0010629	negative regulation of gene expression	96/901	1376/17046	0.00287	0.03569	0.02807	CDKN1C/C1D/ZBTB18/CELF1/CCR1/CTGF/SMYD1/CYLD/RNF168/ZNF366/DNMT3A/PATL2/A2M/ELK4/ESR1/SBNO2/ACIN1/FOXC2/FOXO1/DIP2A/FLOT2/NEDD4L/PUM2/RYBP/PABPC1/DKK3/GPER1/SOX8/NRG1/HMGA1/HOXB3/ID3/IRF1/ISL1/HILS1/HES5/LGALS9/SMAD3/MITF/MOV10/PARK2/LEF1/SIRT6/PITX2/PKHD1/PML/RIPPLY3/TLR9/PIWIL2/PRMT6/DNAJC17/CNOT11/MAP2K2/MASP1/TENM2/GATAD2B/METTL14/CCAR2/CREBZF/TRIM27/NOD2/SFRP2/GZF1/BMP4/ZNF649/SOX9/TBP/ZEB1/TNFAIP3/TWIST1/TNFRSF4/WNT10B/ZNF177/ZC3H14/ZC3H12A/CPEB4/CALR/SCRT1/HIST1H3A/SLA2/NR0B2/HOPX/KDM2B/LOXL3/CBX2/TP63/RUNX3/LIMD1/ERI1/PIAS2/CBFA2T2/AURKB/ADIPOQ/H2AFY/FGF19/NR1H4	96
GO:0007399	nervous system development	143/901	2174/17046	0.0029	0.03582	0.02817	FARP1/CDKN1C/SPON2/ZBTB18/TACC2/TBR1/GPRIN1/CLN5/SEZ6/CNP/COL9A3/SCLT1/ADM/ZNF358/APCDD1/CYP11A1/DLG2/DNMT3A/EFNA2/EGFR/EGR3/EIF4G1/EML1/EPHA1/EPHA3/EPHB4/FGF10/RASA3/BTBD3/SPG20/NFASC/EPB41L3/NEDD4L/PSD3/MAPK8IP2/MTOR/DFNB31/TENM4/FGF22/NPTN/SDCBP2/GPER1/GSTP1/NME7/SOX8/KCNIP2/NRG1/HLX/HPCA/APBA2/HOXB3/HOXD3/HSP90AA1/HSP90AB1/HTR5A/ID3/RSPO2/FMN1/BARHL2/IGF1/IL6/INHBA/ISL1/ACAT1/AMIGO3/INSC/HES5/SLC6A17/STMN1/ARHGDIA/LLGL1/MEF2D/LHX8/NEU1/NHLH2/NRAS/NTF3/PALM/PARK2/UTP11L/LEF1/CEND1/ATP8A2/PITX2/SSH1/MXRA8/PPP1CC/ELP3/IFT122/CSGALNACT1/PRKD1/MAPK3/MAP2K2/PSMB4/RGMA/TRPC7/PSMD7/TENM2/MARK4/RASGRF2/S100A6/SCT/SFRP2/TRA2B/SGK1/BMP4/SLC8A1/BMPR1B/SLIT1/BOK/SOX9/STK3/ZEB1/TIMP3/TRPC4/TRPC6/TWIST1/WNT10B/YWHAG/CACNB2/PAX8/CXCR4/FZD5/LST1/CALR/COLQ/CAST/SCRT1/PARD6B/TTBK1/KDM2B/GAS7/RUNX1/TP63/RUNX3/IRS2/ALDH1A2/SPHK1/CBFA2T2/ARHGEF10/RAPGEF2/ULK2/FGF19	143
GO:0070167	regulation of biomineral tissue development	10/901	68/17046	0.0029	0.03582	0.02817	CCR1/FAM101A/SMAD3/BGLAP/BMP4/SLC8A1/BMPR1B/SOX9/TWIST1/WNT10B	10
GO:0071260	cellular response to mechanical stimulus	10/901	68/17046	0.0029	0.03582	0.02817	IRF1/MAP3K1/ATP1A2/MAPK3/BMP4/SOX9/TLR5/TNFRSF1A/CRADD/FADD	10
GO:0032101	regulation of response to external stimulus	60/901	790/17046	0.00295	0.03612	0.0284	CDH13/TBR1/CCR1/NLRP6/A2M/F11/FGA/FGF10/SBNO2/ACIN1/LARP1/PUM2/MTOR/SLC37A4/GPER1/FFAR2/GSTP1/ANXA2/HK1/IL1R1/IL6/IL16/ISL1/KDR/LCK/LGALS9/SMAD3/NOV/NTF3/PARK2/PDE6B/PIK3CG/PLA2G2A/PML/IL20RB/TLR9/TREM1/PRKD1/MAP2K2/PROC/MASP1/HTRA1/PSMB4/NOD2/STAT2/TNFAIP3/TNFRSF1A/CCR2/CA7/CXCR4/FZD5/RAB7A/NLRX1/CALR/HIST1H3A/HOPX/TNFRSF11A/ADIPOQ/LY86/NUP93	60
GO:0048598	embryonic morphogenesis	46/901	571/17046	0.00296	0.03612	0.0284	CDKN1C/GJB6/COL11A1/ADM/ZNF358/ECE1/SP8/FGF10/FOXC2/TENM4/GNAS/FLVCR1/SOX8/HLX/HOXB3/HOXC4/HOXD3/RSPO2/CYR61/IL1RN/INHBA/ITGA7/ITGB2/HES5/AFF3/LAMA3/SMAD3/LEF1/ATP8A2/PITX2/CHRNA9/IFT122/STRA6/SFRP2/BMP4/SOX9/STK3/ZEB1/TWIST1/PAX8/FZD5/KDM2B/TP63/ALDH1A2/ADIPOQ/MICAL2	46
GO:0005979	regulation of glycogen biosynthetic process	6/901	28/17046	0.00297	0.03612	0.0284	MTOR/GRB10/IGF1/IGF2/PPP1CB/IRS2	6
GO:0010962	regulation of glucan biosynthetic process	6/901	28/17046	0.00297	0.03612	0.0284	MTOR/GRB10/IGF1/IGF2/PPP1CB/IRS2	6
GO:0034698	response to gonadotropin	6/901	28/17046	0.00297	0.03612	0.0284	CYP11A1/EGR3/GJB2/INHBA/LHCGR/PAX8	6
GO:0002698	negative regulation of immune effector process	12/901	91/17046	0.00297	0.03612	0.0284	A2M/HLA-B/HLA-E/HLX/LGALS9/IL20RB/MASP1/HTRA1/NOD2/BST2/CCR2/NLRX1	12

GO:0042307	positive regulation of protein import into nucleus	12/901	91/17046	0.00297	0.03612	0.0284	EGFR/HSP90AB1/IGF1/IL6/JUP/IPO5/LGALS9/SMAD3/TLR9/SFRP2/BMP4/SPHK1	12
GO:1904018	positive regulation of vasculature development	14/901	115/17046	0.00297	0.03612	0.0284	CHI3L1/ADM/EPHA1/FOXC2/ISL1/KDR/ANGPT4/PRKD1/CCL11/SFRP2/TWIST1/RUNX1/SPHK1/RAPGEF2	14
GO:0006874	cellular calcium ion homeostasis	30/901	332/17046	0.003	0.03643	0.02865	TRDN/CCR1/ADM/DRD4/ESR1/NPTN/GPER1/ANXA6/LCK/ATP1A2/OPRL1/PDE6B/PIK3CG/PKHD1/PML/CHRNA9/PRKD1/TRPC7/CCL11/BMP4/SLC8A1/TGM2/TRPC4/TRPC6/CCR2/CXCR4/CALR/ATP13A4/CASQ1/SMDT1	30
GO:0007519	skeletal muscle tissue development	17/901	153/17046	0.00302	0.0365	0.0287	ZBTB18/CHRNA1/SMYD1/FLNB/VGLL2/SOX8/HLX/MEF2D/MEOX2/NFATC3/NRAS/PITX2/PLAGL1/BIN3/TWIST1/WNT10B/CASQ1	17
GO:0030799	regulation of cyclic nucleotide metabolic process	17/901	153/17046	0.00302	0.0365	0.0287	NPFFR2/ADM/ADRB3/DRD4/GABBR1/GNAS/GPER1/GUCY1A3/HPCA/LHCGR/MC2R/NPPC/OPRL1/PALM/MRAP/SCT/CCR2	17
GO:0060485	mesenchyme development	21/901	206/17046	0.00304	0.03663	0.02881	EPHA3/FGF10/FOXC2/SOX8/NRG1/ISL1/SMAD3/MEOX1/LEF1/PITX2/BNC2/ACTA2/S100A4/SFRP2/BMP4/SOX9/ACTC1/TWIST1/LOXL3/ALDH1A2/FGF19	21
GO:0048608	reproductive structure development	34/901	391/17046	0.00308	0.03704	0.02913	CDKN1C/COL9A3/ZFP42/ADM/CYP11A1/EGFR/ESR1/FGF10/GJB2/SOX8/HSD17B2/HSP90AB1/IGF1/CYR61/INHBA/KDR/LHCGR/MC2R/LHX8/LEF1/PITX2/HTRA1/STRA6/SFRP2/BMP4/SLC8A1/BMPR1B/BOK/SOX9/STK3/TLR5/PHLDA2/FZD5/TP63	34
GO:0043122	regulation of I-kappaB kinase/NF-kappaB signaling	22/901	220/17046	0.00311	0.03717	0.02923	MIB2/CYLD/NLRP6/ESR1/GSTP1/LGALS9/NOV/PARK2/TLR9/ZDHHC13/PRKD1/PLEKHG5/S100A4/NOD2/BST2/TNFAIP3/TNFRSF1A/TRAF5/NLRX1/FADD/SPHK1/ADIPOQ	22
GO:0045934	negative regulation of nucleobase-containing compound metabolic process	90/901	1280/17046	0.00311	0.03717	0.02923	CDKN1C/C1D/ZBTB18/SMYD1/CYLD/RNF168/ZNF366/DNMT3A/DRD4/ELK4/ESR1/SBNO2/ACIN1/FOXC2/FOXO1/NEDD4L/RYBP/GABBR1/PABPC1/DNAJC2/DKK3/GPER1/DNAJC15/GZMA/SOX8/NRG1/HMGA1/HPCA/HOXB3/HOXC6/ID3/IRF1/ISL1/HILS1/HES5/SMAD3/MITF/NPPC/OPRL1/PALM/LEF1/SIRT6/PITX2/PKHD1/PML/RIPPLY3/TLR9/PRMT6/DNAJC17/TENM2/GATAD2B/CCAR2/CREBZF/TRIM27/NOD2/SFRP2/GZF1/BMP4/ZNF649/SOX9/TBP/ZEB1/TERF1/TNFAIP3/TWIST1/CCR2/TNFRSF4/WNT10B/ZNF177/ZC3H14/ZC3H12A/CALR/SLIRP/SCRT1/HIST1H3A/SLA2/NR0B2/HOPX/KDM2B/LOXL3/CBX2/TP63/RUNX3/LIMD1/PIAS2/CBFA2T2/AURKB/ADIPOQ/H2AFY/NR1H4	90
GO:0030326	embryonic limb morphogenesis	15/901	128/17046	0.00311	0.03717	0.02923	ZNF358/ECE1/SP8/GNAS/FLVCR1/RSPO2/AFF3/LEF1/PITX2/IFT122/SFRP2/BMP4/TWIST1/TP63/ALDH1A2	15
GO:0035113	embryonic appendage morphogenesis	15/901	128/17046	0.00311	0.03717	0.02923	ZNF358/ECE1/SP8/GNAS/FLVCR1/RSPO2/AFF3/LEF1/PITX2/IFT122/SFRP2/BMP4/TWIST1/TP63/ALDH1A2	15
GO:0006954	inflammatory response	47/901	588/17046	0.00312	0.03717	0.02923	KLRG1/CHI3L1/CCR1/PARP4/NLRP6/A2M/UNC13D/FGA/SBNO2/GPER1/FFAR2/GSTP1/HRH1/IL1R1/IL1RN/IL6/ISL1/ITGB2/ITIH4/LGALS9/SMAD3/NFATC3/NOV/PIK3CG/PLA2G2A/IL20RB/TLR9/PRKD1/MASP1/PSMB4/CCL11/CCL17/NOD2/BMPR1B/TLR5/TNFAIP3/TNFRSF1A/CCR2/TNFRSF4/CXCR4/NLRX1/IL1F10/TNFRSF11A/SPHK1/CCRL2/ADIPOQ/LY86	47
GO:0030004	cellular monovalent inorganic cation homeostasis	11/901	80/17046	0.00314	0.03734	0.02937	TCIRG1/CLN5/NEDD4L/ATP1A2/ATP5B/SLAMF8/SGK1/SLC4A1/SLC8A1/CA7/RAB7A	11
GO:0051235	maintenance of location	21/901	207/17046	0.00322	0.03818	0.03003	TRDN/CIDEA/FITM1/EPB41L3/FLNB/SYNE1/MORC3/FFAR2/HK1/ACACB/IL6/JUP/LCK/LTBP1/PML/SLC30A10/ABHD4/ZC3H12A/CALR/CASQ1/SCIN	21
GO:0008015	blood circulation	35/901	407/17046	0.00323	0.03825	0.03008	KCNMB2/MRVI1/TRDN/CELF2/CHGA/ADM/CPS1/CTGF/ADRB3/ABAT/ECE1/FGA/FOXC2/BMP10/GPER1/GUCY1A3/KCNIP2/HRH1/JUP/KCNH2/KCNJ8/MEOX2/MYL2/ATP1A2/NPPC/OPRL1/PIK3CG/POMC/ACTA2/SGK1/SLC8A1/ACTC1/CACNA1E/HOPX/ADIPOQ	35

GO:0072234	metanephric nephron tubule development	5/901	20/17046	0.00325	0.03845	0.03024	SOX8/ACAT1/HES5/SOX9/PAX8	5
GO:0050801	ion homeostasis	50/901	636/17046	0.00327	0.03854	0.03031	TCIRG1/TRDN/CLN5/CCR1/ADM/CPS1/DRD4/ESR1/NEDD4L/STEAP2/NPTN/GPER1/FLVCR1/ANXA6/KCNH2/KDR/LCK/MFI2/NUBP1/ATP1A2/OPRL1/ATP5B/PARK2/PDE6B/PIK3CG/PKHD1/PML/SLC30A10/CHRNA9/PRKD1/SLAMF8/TRPC7/CCL11/SGK1/BMP4/SLC4A1/SLC8A1/SLC9A3/TGM2/TRPC4/TRPC6/CCR2/CA7/CXCR4/RAB7A/CALR/ATP13A4/CASQ1/SMDT1/MTL5	50
GO:0044711	single-organism biosynthetic process	107/901	1567/17046	0.00329	0.03875	0.03047	CDH3/TCIRG1/MTHFS/NPFFR2/ADCY3/SLC27A2/ERLIN2/B4GALT7/ACOT7/MRPL52/ADM/CPS1/CTGF/PPM1L/MBOAT1/ADRB3/CYP11A1/FITM1/ADAL/DDB1/ABAT/DPH1/DRD4/AGXT/EEF2/ENO2/ALAS1/FOXO1/AKR1B1/MTOR/GABBR1/ST6GALNAC3/GATM/GBGT1/GAPDHS/AMPD2/DKK3/GLS2/AMPD3/GNAS/PIGW/GPER1/GRB10/MRPS18B/GSTP1/GUCY1A3/NME7/PADI1/HAS1/ACACB/HPCA/HRH1/HSD11B1/HSD17B2/ACADL/NME9/IGF1/IGF2/CYR61/IL6/ACAT1/LDLR/LHCGR/MC2R/ME1/NPPC/OAS2/OPRL1/ATP5B/PALM/CHST15/PGAM2/PIGC/PIK3CG/PKM/PLA2G2A/PRKAG3/PML/CYTL1/LPCAT2/PPP1CB/VAC14/CSGALNACT1/PRKD1/MRAP/RFC2/RPA3/SCT/MRPS14/BMP4/TERF1/CCR2/UPP1/CERS4/QTRT1/KMO/IRS2/ALDH1A2/SYNJ2/SPHK1/CH25H/SDR42E1/ADIPOQ/RAPGEF2/LPGAT1/FGF19/NR1H4	107
GO:0055002	striated muscle cell development	15/901	129/17046	0.00336	0.03941	0.03099	SPEG/BMP10/TMOD4/IGF1/LMNA/MYL2/NFATC3/LEF1/PITX2/BIN3/BMP4/SLC8A1/ACTC1/WNT10B/CASQ1	15
GO:0035137	hindlimb morphogenesis	7/901	38/17046	0.00337	0.03941	0.03099	GNAS/RSPO2/FMN1/AFF3/PITX2/BMP4/TWIST1	7
GO:0072210	metanephric nephron development	7/901	38/17046	0.00337	0.03941	0.03099	SOX8/FMN1/HES5/BMP4/SOX9/PAX8/ADIPOQ	7
GO:0051223	regulation of protein transport	53/901	684/17046	0.00337	0.03941	0.03099	CIDEA/SLC51B/CYLD/ABAT/DRD4/EGFR/FGA/EXPH5/MTOR/GLS2/GNAS/GPR26/GPER1/FFAR2/NRG1/ANXA13/HLA-E/HPCA/HSPA1L/HSP90AB1/IGF1/IL6/ISL1/JUP/IPO5/LCP1/LGALS9/LLGL1/SMAD3/NOV/PARK2/PDE4C/PML/TLR9/GOLPH3L/SYBU/PRKAR1B/TRIM27/NOD2/SFRP2/BMP4/VAMP2/TWIST1/TNFRSF4/CACNA1E/FZD5/RAB11FIP1/NR0B2/IRS2/SPHK1/SYT7/RSAD2/ADIPOQ	53
GO:1904035	regulation of epithelial cell apoptotic process	8/901	48/17046	0.00339	0.03954	0.03109	FGA/GPER1/IL6/KDR/BMP4/TNFAIP3/COL18A1/CAST	8
GO:0043410	positive regulation of MAPK cascade	45/901	560/17046	0.00342	0.03978	0.03128	CHI3L1/CCR1/MAP3K8/CTGF/ADRB3/DRD4/EGFR/FGA/FGF10/RASA3/MAPK8IP2/FGF22/GPER1/NRG1/IGF1/IGF2/IL1RN/IL6/KDR/LGALS9/MAP3K1/NRAS/NTF3/PIK3CG/PLA2G2A/TLR9/MAPK3/MAP2K2/PSMB4/PSMD7/PXN/RASGRF2/CCL11/CCL17/NOD2/BMP4/STK3/CXCR4/FZD5/CDK10/IRS2/TNFRSF11A/MAP3K6/RAPGEF2/FGF19	45
GO:0031347	regulation of defense response	55/901	716/17046	0.00342	0.03978	0.03128	TANK/MAP3K8/CYLD/COCH/NLRP6/DMBT1/A2M/SBNO2/ACIN1/PUM2/SLC37A4/GPER1/FFAR2/GSTP1/HLA-B/HLA-E/HSP90AB1/IL1R1/IL6/IRF1/ISL1/ITGB2/LCK/LGALS9/SMAD3/MAP3K1/NFATC3/NOV/NRAS/PIK3CG/PLA2G2A/PML/IL20RB/TLR9/TREM1/MAPK3/MASP1/HTRA1/PSMB4/PSMD7/NOD2/STAT2/TLR5/TNFAIP3/TNFRSF1A/CCR2/CA7/NLRX1/UNC93B1/HIST1H3A/FADD/TNFRSF11A/RSAD2/ADIPOQ/NUP93	55
GO:0055001	muscle cell development	16/901	142/17046	0.00343	0.03979	0.03129	SPEG/ADM/BMP10/TMOD4/IGF1/LMNA/MYL2/NFATC3/LEF1/PITX2/BIN3/BMP4/SLC8A1/ACTC1/WNT10B/CASQ1	16
GO:0006811	ion transport	99/901	1435/17046	0.00346	0.03999	0.03145	KCNMB2/TCIRG1/TRDN/SLC27A2/CHRNA1/CHRNA2/CHRNA5/PANX3/CLCA1/CCR1/SLC51B/C15orf27/SLC38A10/TRPM6/CTGF/TRPV3/BHLHA15/DRD4/AGXT/SLC10A4/RASA3/MLC1/ATP11A/NEDD4L/MAPK8IP2/STEAP2/SLC17A5/GLS2/CRACR2B/GPER1/GRIK4/KCNIP2/ANXA6/ACACB/HTR3A/IL1RN/AQP2/AQP5/AQP9/ATP9B/KCNH2/KCNJ8/KCNJ9/KCNMB1/SLC6A17/LCK/LDLR/MFI2/ATP1A2/NFATC3/OPRL1/SLC22A18/P2RY6/ATP5B/ANO7/PARK2/PCYOX1/ATP8A2/PIK3CG/PML/FXYD6/SLCO1C1/TLR9/ZDHHC13/SLC47A1/TRPV6/SLC30A10/CHRNA9/PRKD1/TRPV5/TRPC7/RASGRF2/TRIM27/S100A6/SGK1/BMP4/SLC4A1/SLC6A12/SLC8A1/SLC9A3/SLC20A2/VAMP2/TRAPPC10/TRPC4/TRPC6/TRPM2/TWIST1/UCP1/CA7/CACNA1E/CACNB2/ATP13A4/CASQ1/SLC43A1/IRS2/TNFRSF11A/SLC16A3/SMDT1/NR1H4	99
GO:0003013	circulatory system process	35/901	409/17046	0.00349	0.04033	0.03172	KCNMB2/MRVI1/TRDN/CELF2/CHGA/ADM/CPS1/CTGF/ADRB3/ABAT/ECE1/FGA/FOXC2/BMP10/GPER1/GUCY1A3/KCNIP2/HRH1/JUP/KCNH2/KCNJ8/MEOX2/MYL2/ATP1A2/NPPC/OPRL1/PIK3CG/POMC/ACTA2/SGK1/SLC8A1/ACTC1/CACNA1E/HOPX/ADIPOQ	35
GO:0055065	metal ion homeostasis	42/901	515/17046	0.00354	0.04084	0.03212	TCIRG1/TRDN/CCR1/ADM/DRD4/ESR1/NEDD4L/STEAP2/NPTN/GPER1/FLVCR1/ANXA6/KCNH2/KDR/LCK/MFI2/NUBP1/ATP1A2/OPRL1/PARK2/PDE6B/PIK3CG/PKHD1/PML/SLC30A10/CHRNA9/PRKD1/TRPC7/CCL11/SGK1/BMP4/SLC8A1/TGM2/TRPC4/TRPC6/CCR2/CXCR4/CALR/ATP13A4/CASQ1/SMDT1/MTL5	42
GO:1904591	positive regulation of protein import	12/901	93/17046	0.00356	0.0409	0.03216	EGFR/HSP90AB1/IGF1/IL6/JUP/IPO5/LGALS9/SMAD3/TLR9/SFRP2/BMP4/SPHK1	12
GO:0035116	embryonic hindlimb morphogenesis	6/901	29/17046	0.00358	0.0409	0.03216	GNAS/RSPO2/AFF3/PITX2/BMP4/TWIST1	6

GO:0060740	prostate gland epithelium morphogenesis	6/901	29/17046	0.00358	0.0409	0.03216	ESR1/FGF10/IGF1/BMP4/SOX9/TP63	6
GO:0061036	positive regulation of cartilage development	6/901	29/17046	0.00358	0.0409	0.03216	BMP10/CYR61/SMAD3/BMP4/BMPR1B/SOX9	6
GO:0009890	negative regulation of biosynthetic process	95/901	1370/17046	0.0036	0.04105	0.03228	CDKN1C/C1D/ZBTB18/CELF1/ERLIN2/SMYD1/CYLD/RNF168/ZNF366/DNMT3A/DRD4/PATL2/ELK4/ESR1/SBNO2/FOXC2/FOXO1/NEDD4L/RYBP/GABBR1/DNAJC2/DKK3/GPER1/GRB10/GSTP1/SOX8/NRG1/HMGA1/HPCA/HOXB3/HOXC6/ACADL/ID3/IL6/INHBA/IRF1/ISL1/HILS1/HES5/SMAD3/MITF/OPRL1/PALM/LEF1/SIRT6/PITX2/PKHD1/PML/RIPPLY3/TLR9/PRMT6/DNAJC17/TENM2/GATAD2B/METTL14/CCAR2/CREBZF/TRIM27/NOD2/SFRP2/GZF1/BMP4/ZNF649/SOX9/TBP/ZEB1/TERF1/TNFAIP3/TWIST1/CCR2/TNFRSF4/WNT10B/ZNF177/ZC3H12A/CPEB4/CALR/SCRT1/HIST1H3A/SLA2/NR0B2/HOPX/KDM2B/LOXL3/CBX2/TP63/RUNX3/LIMD1/PIAS2/CBFA2T2/AURKB/ADIPOQ/H2AFY/RAPGEF2/FGF19/NR1H4	95
GO:0061458	reproductive system development	34/901	395/17046	0.00361	0.04116	0.03237	CDKN1C/COL9A3/ZFP42/ADM/CYP11A1/EGFR/ESR1/FGF10/GJB2/SOX8/HSD17B2/HSP90AB1/IGF1/CYR61/INHBA/KDR/LHCGR/MC2R/LHX8/LEF1/PITX2/HTRA1/STRA6/SFRP2/BMP4/SLC8A1/BMPR1B/BOK/SOX9/STK3/TLR5/PHLDA2/FZD5/TP63	34
GO:0030595	leukocyte chemotaxis	18/901	169/17046	0.00366	0.04167	0.03277	CHGA/CCR1/FFAR2/HRH1/IL6/IL16/ITGB2/LGALS9/NOV/PIK3CG/TREM1/CCL11/CCL17/NOD2/CCR2/CXCR4/CALR/TNFRSF11A	18
GO:0048762	mesenchymal cell differentiation	17/901	156/17046	0.00369	0.04182	0.03289	EPHA3/FGF10/FOXC2/SOX8/NRG1/ISL1/SMAD3/LEF1/PITX2/S100A4/SFRP2/BMP4/SOX9/TWIST1/LOXL3/ALDH1A2/FGF19	17
GO:0019318	hexose metabolic process	23/901	237/17046	0.00369	0.04182	0.03289	GALM/B3GLCT/ENO2/FOXO1/AKR1B1/MTOR/FUCA1/SLC37A4/GAPDHS/GRB10/HK1/IGF1/IGF2/IL6/PARK2/CHST15/PGAM2/PKM/POMC/PPP1CB/PHLDA2/IRS2/ADIPOQ	23
GO:0050727	regulation of inflammatory response	24/901	251/17046	0.0037	0.04182	0.03289	NLRP6/A2M/SBNO2/GPER1/FFAR2/GSTP1/IL1R1/IL6/ISL1/SMAD3/NOV/PIK3CG/PLA2G2A/IL20RB/TLR9/MASP1/PSMB4/NOD2/TNFAIP3/TNFRSF1A/CCR2/NLRX1/TNFRSF11A/ADIPOQ	24
GO:0031324	negative regulation of cellular metabolic process	141/901	2154/17046	0.00371	0.04182	0.03289	FARP1/CDKN1C/C1D/ZBTB18/CELF1/ERLIN2/CARD16/CSTA/SMYD1/CYLD/RNF168/ZNF366/NLRP6/DLG2/DNMT3A/DRD4/PATL2/A2M/ELK4/EPHA1/ESR1/FHIT/PPM1E/SBNO2/ACIN1/FOXC2/FOXO1/NEDD4L/RYBP/MTOR/GABBR1/PABPC1/DNAJC2/DKK3/GPER1/GRB10/DNAJC15/GSTP1/GZMA/ANXA2/SERPIND1/SOX8/NRG1/HMGA1/ACACB/HPCA/HOXB3/HOXC6/ACADL/HSP90AB1/ID3/COL28A1/IGF1/IL6/INHBA/IRF1/ISL1/ITIH3/ITIH4/HILS1/KIF25/HES5/SMAD3/MITF/NPPC/NTF3/OPRL1/PALM/PARK2/SPOCK3/LEF1/SIRT6/PI3/PIK3CG/PITX2/PKHD1/PML/RIPPLY3/TLR9/PRMT6/DNAJC17/PRKAR1B/MASP1/PSMB4/PSMD7/TENM2/GATAD2B/METTL14/CCAR2/CREBZF/TRIM27/NOD2/SFRP2/GZF1/BMP4/ZNF649/SOX9/SUPT6H/BST2/TBP/ZEB1/TERF1/TIMP3/TNFAIP3/TWIST1/CCR2/TNFRSF4/WNT10B/YWHAG/ZNF177/ZC3H14/ZC3H12A/CPEB4/CALR/SLIRP/CAST/SCRT1/HIST1H3A/SLA2/NR0B2/HOPX/SPINK7/KDM2B/LOXL3/CBX2/TP63/RUNX3/SERPINA6/IRS2/FADD/LIMD1/PIAS2/CBFA2T2/AURKB/DAPL1/ADIPOQ/H2AFY/N4BP1/RAPGEF2/FGF19/NR1H4	141
GO:0006941	striated muscle contraction	14/901	118/17046	0.00376	0.04213	0.03313	CHRNA1/CTGF/DTNA/BMP10/KCNH2/MYL2/ATP1A2/ATP8A2/PGAM2/PIK3CG/SLC8A1/ACTC1/CASQ1/RCSD1	14
GO:0007169	transmembrane receptor protein tyrosine kinase signaling pathway	65/901	879/17046	0.00377	0.04213	0.03313	ABI1/CDH3/CDH13/TCIRG1/ADCY3/LECT1/ESM1/AP3S1/IL31RA/CTGF/EFNA2/EGFR/EIF4G1/EPHA1/EPHA3/EPHB4/FGF10/RASA3/FOXC2/FOXO1/ARHGEF18/MTOR/FGF22/NPTN/GPER1/GRB10/NRG1/NR4A1/HSP90AA1/IGF1/IGF2/JUP/KDR/LCK/ARHGDIA/MOV10/NRAS/NTF3/ARHGEF3/ANGPT4/PRKAG3/TLR9/PRKAR1B/LMBRD1/PAG1/PRKD1/MAPK3/MAP2K2/PSMB4/PSMD7/PLEKHG5/PTPRE/PXN/RASGRF2/RIT2/SOX9/ZAP70/RAB7A/TMEM204/IRS2/SPHK1/CD8A/ADIPOQ/RAPGEF2/FGF19	65
GO:0009617	response to bacterium	38/901	456/17046	0.00377	0.04213	0.03313	SPON2/GJB6/CHGA/CNP/ADM/CPS1/CYP11A1/COCH/NLRP6/DMBT1/FGA/FGF10/SBNO2/GSTP1/HLA-B/HLA-E/IL1RN/IL6/IL10RA/IL12RB2/KCNJ8/LGALS9/PLA2G2A/TLR9/TREM1/MAPK3/SLAMF8/PTGFR/DEFB134/NOD2/BPI/TLR5/TNFAIP3/TNFRSF1A/FZD5/ZC3H12A/TNFRSF11A/LY86	38
GO:0072044	collecting duct development	4/901	13/17046	0.00378	0.04213	0.03313	AKR1B1/AQP2/BMP4/PAX8	4
GO:0072189	ureter development	4/901	13/17046	0.00378	0.04213	0.03313	SOX8/BMP4/SOX9/ALDH1A2	4
GO:2000479	regulation of cAMP-dependent protein kinase activity	4/901	13/17046	0.00378	0.04213	0.03313	NPFFR2/PRKAR1B/ADIPOQ/RAPGEF2	4

GO:0072503	cellular divalent inorganic cation homeostasis	31/901	352/17046	0.0038	0.04231	0.03327	TRDN/CCR1/ADM/DRD4/ESR1/NPTN/GPER1/ANXA6/LCK/ATP1A2/OPRL1/PDE6B/PIK3CG/PKHD1/PML/SLC30A10/CHRNA9/PRKD1/TRPC7/CCL11/BMP4/SLC8A1/TGM2/TRPC4/TRPC6/CCR2/CXCR4/CALR/ATP13A4/CASQ1/SMDT1	31
GO:0032649	regulation of interferon-gamma production	11/901	82/17046	0.00381	0.0424	0.03334	HLA-DPA1/IL12RB2/INHBA/ISL1/LGALS9/IL20RB/TLR9/NOD2/CCR2/FZD5/FADD	11
GO:0045766	positive regulation of angiogenesis	13/901	106/17046	0.00386	0.0428	0.03366	CHI3L1/ADM/EPHA1/FOXC2/ISL1/KDR/ANGPT4/PRKD1/CCL11/SFRP2/TWIST1/RUNX1/SPHK1	13
GO:1903522	regulation of blood circulation	22/901	224/17046	0.00387	0.0428	0.03366	KCNMB2/TRDN/CELF2/ADM/CPS1/CTGF/ECE1/FGA/NEDD4L/BMP10/GPER1/KCNIP2/HRH1/JUP/KCNH2/MYL2/ATP1A2/NPPC/PIK3CG/SLC8A1/CACNA1E/HOPX	22
GO:0032635	interleukin-6 production	12/901	94/17046	0.00389	0.04294	0.03377	SPON2/HLA-B/IL6/ISL1/LGALS9/TLR9/NOD2/BPI/TNFAIP3/TWIST1/NLRX1/ZC3H12A	12
GO:0048863	stem cell differentiation	23/901	238/17046	0.00389	0.04294	0.03377	A2M/EPHA3/FGF10/FOXC2/SOX8/NRG1/ISL1/HES5/SMAD3/MEOX1/LEF1/PITX2/S100A4/SFRP2/BMP4/SOX9/TEAD3/TWIST1/PAX8/LOXL3/TP63/ALDH1A2/FGF19	23
GO:0051090	regulation of sequence-specific DNA binding transcription factor activity	30/901	338/17046	0.0039	0.04295	0.03377	CYLD/ESR1/ID3/IL6/JUP/LGALS9/NHLH2/PKHD1/RIPK4/TLR9/CYTL1/PRKD1/MAPK3/CREBZF/TRIM27/NOD2/SGK1/STK3/TNFAIP3/TRAF1/TRAF5/TWIST1/TNFRSF4/WNT10B/CARD14/ZC3H12A/NR0B2/TNFRSF11A/SPHK1/PIAS2	30
GO:0006875	cellular metal ion homeostasis	37/901	442/17046	0.00392	0.04301	0.03382	TCIRG1/TRDN/CCR1/ADM/DRD4/ESR1/NEDD4L/NPTN/GPER1/FLVCR1/ANXA6/LCK/NUBP1/ATP1A2/OPRL1/PDE6B/PIK3CG/PKHD1/PML/SLC30A10/CHRNA9/PRKD1/TRPC7/CCL11/SGK1/BMP4/SLC8A1/TGM2/TRPC4/TRPC6/CCR2/CXCR4/CALR/ATP13A4/CASQ1/SMDT1/MTL5	37
GO:0002704	negative regulation of leukocyte mediated immunity	7/901	39/17046	0.00393	0.04301	0.03382	HLA-B/HLA-E/LGALS9/IL20RB/NOD2/BST2/CCR2	7
GO:0034121	regulation of toll-like receptor signaling pathway	7/901	39/17046	0.00393	0.04301	0.03382	NLRP6/IRF1/TLR9/NOD2/TLR5/TNFAIP3/RSAD2	7
GO:0045216	cell-cell junction organization	21/901	211/17046	0.00403	0.04403	0.03463	CDH3/CDH9/CDH12/CDH13/PKP3/MPP7/EPHA3/NFASC/EPB41L3/GJB2/FMN1/JUP/KDR/SMAD3/MPZ/LIMS2/PXN/FZD5/PARD6G/PARD6B/ACTN1	21
GO:0030324	lung development	19/901	184/17046	0.00406	0.04432	0.03486	PDPN/CHI3L1/CTGF/FGF10/HSD11B1/RSPO2/IGF1/KDR/INSC/LOX/C11orf73/PITX2/MAPK3/MAP2K2/STRA6/BMP4/SOX9/HOPX/ALDH1A2	19
GO:0035767	endothelial cell chemotaxis	5/901	21/17046	0.00409	0.04434	0.03487	EGR3/NR4A1/NOV/PRKD1/PLEKHG5	5
GO:1901020	negative regulation of calcium ion transmembrane transporter activity	5/901	21/17046	0.00409	0.04434	0.03487	TRDN/DRD4/ATP1A2/OPRL1/TLR9	5
GO:1903170	negative regulation of calcium ion transmembrane transport	5/901	21/17046	0.00409	0.04434	0.03487	TRDN/DRD4/ATP1A2/OPRL1/TLR9	5

GO:0035270	endocrine system development	15/901	132/17046	0.00418	0.04527	0.0356	CDKN1C/FGF10/FOXO1/DKK3/HOXB3/HOXD3/IL6/ISL1/SMAD3/PITX2/STRA6/BMP4/SOX9/PAX8/ALDH1A2	15
GO:0050680	negative regulation of epithelial cell proliferation	13/901	107/17046	0.00419	0.04529	0.03562	CDKN1C/LECT1/VASH1/MCC/PLA2G2A/LIMS2/IFT122/SLURP1/SFRP2/BMP4/SOX9/WNT10B/RUNX3	13
GO:0009306	protein secretion	38/901	459/17046	0.0042	0.04529	0.03562	CHI3L1/CIDEA/ABAT/DRD4/FGA/EXPH5/GNAS/GPER1/FFAR2/HLA-E/IGF1/IL1RN/IL6/ISL1/LGALS9/LLGL1/NOV/PARK2/PDE4C/PML/TLR9/TREM1/GOLPH3L/SYBU/PRKAR1B/TRIM27/NOD2/VAMP2/TWIST1/TNFRSF4/CACNA1E/RAB11FIP1/NR0B2/MON1A/IRS2/SYT7/RSAD2/RAB3D	38
GO:1903531	negative regulation of secretion by cell	17/901	158/17046	0.00421	0.04529	0.03562	CHGA/CIDEA/DRD4/IL1RN/IL6/INHBA/LGALS9/NOV/PARK2/PDE4C/PML/TRIM27/CCR2/TNFRSF4/RAB11FIP1/RSAD2/ADIPOQ	17
GO:0005975	carbohydrate metabolic process	62/901	835/17046	0.00421	0.04529	0.03562	GNE/CHI3L1/CHI3L2/B4GALT7/GALNT15/CLN5/SLC51B/NEU4/GALM/CPS1/PARP4/B3GLCT/MGAT5B/ADRB3/DDOST/ENO2/TRAK1/FOXO1/AKR1B1/NUP210/MTOR/FUCA1/SLC37A4/ST6GALNAC3/GBGT1/GAPDHS/SLC17A5/DHDH/GPER1/EOGT/GRB10/HAS1/HK1/HRH1/IGF1/IGF2/IL6/MUC21/LHCGR/ME1/MGAT1/NEU1/OAS2/PARK2/CHST15/SIRT6/GALNT7/PGAM2/PKM/PRKAG3/POMC/PPP1CB/PPP1CC/CSGALNACT1/PHLDA2/MOGS/CALR/RAE1/IRS2/STBD1/ADIPOQ/NUP93	62
GO:0045893	positive regulation of transcription, DNA-templated	94/901	1362/17046	0.00431	0.04618	0.03631	CDH13/CDKN1C/DMRT2/TBR1/ERLIN2/PSIP1/IL31RA/CITED4/BHLHA15/EGFR/ESR1/FGF10/SBNO2/FOXC2/FOXO1/VGLL2/MTOR/DNAJC2/BMP10/GPER1/BRF1/SOX8/HMGA1/NR4A1/TFAP2E/BARHL2/IGF1/IGF2/CYR61/IL6/FOXK2/INHBA/IRF1/ISL1/JUP/HES5/LGALS9/LMNA/LMO2/SMAD3/MEF2D/MEOX1/MEOX2/MITF/NFATC3/NFYB/NHLH2/NTF3/PARK2/LEF1/PITX2/PLAGL1/RIPK4/TLR9/CYTL1/POMC/BANP/PRKD1/MAPK3/ARNTL2/RGMA/TRIM27/NPAS3/NOD2/SFRP2/BMP4/ZNF649/BMPR1B/SOX9/STK3/SUPT6H/TBP/TCEA1/ZEB1/TEAD3/TNFRSF1A/TRAF1/TRAF5/TWIST1/WNT10B/PAX8/FZD5/CARD14/RUNX1/TP63/RUNX3/FADD/TNFRSF11A/SPHK1/PIAS2/LDB2/CBFA2T2/MICAL2/NR1H4	94
GO:1903508	positive regulation of nucleic acid-templated transcription	94/901	1362/17046	0.00431	0.04618	0.03631	CDH13/CDKN1C/DMRT2/TBR1/ERLIN2/PSIP1/IL31RA/CITED4/BHLHA15/EGFR/ESR1/FGF10/SBNO2/FOXC2/FOXO1/VGLL2/MTOR/DNAJC2/BMP10/GPER1/BRF1/SOX8/HMGA1/NR4A1/TFAP2E/BARHL2/IGF1/IGF2/CYR61/IL6/FOXK2/INHBA/IRF1/ISL1/JUP/HES5/LGALS9/LMNA/LMO2/SMAD3/MEF2D/MEOX1/MEOX2/MITF/NFATC3/NFYB/NHLH2/NTF3/PARK2/LEF1/PITX2/PLAGL1/RIPK4/TLR9/CYTL1/POMC/BANP/PRKD1/MAPK3/ARNTL2/RGMA/TRIM27/NPAS3/NOD2/SFRP2/BMP4/ZNF649/BMPR1B/SOX9/STK3/SUPT6H/TBP/TCEA1/ZEB1/TEAD3/TNFRSF1A/TRAF1/TRAF5/TWIST1/WNT10B/PAX8/FZD5/CARD14/RUNX1/TP63/RUNX3/FADD/TNFRSF11A/SPHK1/PIAS2/LDB2/CBFA2T2/MICAL2/NR1H4	94
GO:0044282	small molecule catabolic process	29/901	326/17046	0.00436	0.04659	0.03664	BCKDK/MTHFS/SLC27A2/HIBADH/ACOT7/CRABP1/ABAT/AGXT/ENO2/FAH/MTOR/PNKD/GAPDHS/GLS2/DHDH/HK1/ACADL/ACAT1/LDLR/NUDT1/PCYOX1/PGAM2/PKM/PON1/SMPD3/TWIST1/KMO/IRS2/ADIPOQ	29
GO:0060440	trachea formation	3/901	7/17046	0.00439	0.04683	0.03682	MAPK3/MAP2K2/BMP4	3
GO:0002252	immune effector process	53/901	693/17046	0.00439	0.04683	0.03682	ABI1/SPON2/CHGA/RNF168/DMBT1/A2M/UNC13D/FCGR2A/SBNO2/ACIN1/PUM2/SLC37A4/FFAR2/HLA-B/HLA-E/HLX/HSP90AA1/HSP90AB1/IL6/IRF1/KCNJ8/LCK/LCP1/LGALS9/OAS2/LEF1/PIK3CG/PML/IL20RB/TREM1/APBB1IP/MAPK3/MASP1/HTRA1/NOD2/STAT2/SUPT6H/BST2/VAMP2/TNFAIP3/CCR2/TNFRSF4/CA7/FZD5/NLRX1/UNC93B1/HIST1H3A/SLA2/IFITM1/FADD/RSAD2/CD8A/NUP93	53
GO:0007422	peripheral nervous system development	10/901	72/17046	0.00442	0.04697	0.03694	CYP11A1/EGR3/NFASC/SOX8/NRG1/ISL1/NTF3/RUNX1/RUNX3/ARHGEF10	10
GO:0043010	camera-type eye development	26/901	283/17046	0.00442	0.04697	0.03694	CDKN1C/DRD4/FGF10/FOXC2/SOX8/HPCA/AQP5/INHBA/HES5/SMAD3/MAP3K1/MITF/PDE6B/ATP8A2/PITX2/IFT122/STRA6/BMP4/BMPR1B/SOX9/ZEB1/TWIST1/FZD5/BFSP2/KDM2B/ALDH1A2	26
GO:0060541	respiratory system development	21/901	213/17046	0.00449	0.04763	0.03746	SPEG/PDPN/CHI3L1/CTGF/FGF10/HSD11B1/RSPO2/IGF1/KDR/INSC/LOX/LEF1/C11orf73/PITX2/MAPK3/MAP2K2/STRA6/BMP4/SOX9/HOPX/ALDH1A2	21
GO:0032637	interleukin-8 production	9/901	61/17046	0.00452	0.04784	0.03762	CHI3L1/FFAR2/LGALS9/TLR9/NOD2/BPI/TLR5/FADD/ADIPOQ	9
GO:0070663	regulation of leukocyte proliferation	19/901	186/17046	0.00457	0.04827	0.03796	FGF10/GSTP1/HLA-DPA1/HLA-E/ZC3H12D/IGF1/IGF2/IL6/IRF1/LGALS9/IL20RB/BMP4/TNFAIP3/CCR2/TNFRSF4/ZAP70/LST1/IRS2/FADD	19

GO:0048010	vascular endothelial growth factor receptor signaling pathway	26/901	284/17046	0.00463	0.04885	0.03841	ABI1/LECT1/EGFR/FGF10/RASA3/FOXC2/MTOR/FGF22/GRB10/NRG1/HSP90AA1/JUP/KDR/NRAS/PRKD1/MAPK3/MAP2K2/PSMB4/PSMD7/PXN/RASGRF2/TMEM204/IRS2/SPHK1/RAPGEF2/FGF19	26
GO:0002684	positive regulation of immune system process	62/901	839/17046	0.00468	0.04905	0.03857	ABI1/TANK/SPON2/CCR1/MAP3K8/CYLD/COCH/NLRP6/DMBT1/EGR3/A2M/UNC13D/FCGR2A/FGF10/ACIN1/FLOT2/PUM2/MTOR/GNAS/FFAR2/HLA-B/HLA-DPA1/HLA-E/HLX/HSP90AA1/HSP90AB1/IGF1/IGF2/IL6/INHBA/IRF1/ITGB2/LCK/LGALS9/MAP3K1/NFATC3/NRAS/LEF1/TLR9/PAG1/MAPK3/MASP1/PSMB4/PSMD7/NOD2/TLR5/TNFAIP3/CCR2/TNFRSF4/ZAP70/FZD5/NLRX1/CALR/UNC93B1/SLA2/SCIN/RUNX1/IRS2/FADD/SKAP2/RSAD2/CD79A	62
GO:0007219	Notch signaling pathway	20/901	200/17046	0.00468	0.04905	0.03857	TSPAN5/RCAN2/MIB2/AGXT/FGF10/FOXC2/HOXD3/HES5/LLGL1/MFNG/MOV10/NOV/ANGPT4/PGAM2/NOD2/SOX9/TLE3/NR0B2/TP63/NR1H4	20
GO:0009895	negative regulation of catabolic process	20/901	200/17046	0.00468	0.04905	0.03857	CIDEA/EGFR/FHIT/MTOR/PABPC1/ANXA2/NRG1/HSP90AB1/KIF25/SMAD3/SIRT6/PIK3CG/PML/BANP/CCAR2/TERF1/TIMP3/SLIRP/DAPL1/N4BP1	20
GO:0032355	response to estradiol	14/901	121/17046	0.00472	0.04946	0.0389	CTGF/DNMT3A/EGFR/ESR1/FGA/FGF10/GJB2/GPER1/HTR5A/OPRL1/PTGFR/BMP4/CALR/ALDH1A2	14
GO:0007249	I-kappaB kinase/NF-kappaB signaling	23/901	242/17046	0.00477	0.04987	0.03922	TANK/MIB2/CYLD/NLRP6/ESR1/GSTP1/LGALS9/NOV/PARK2/TLR9/ZDHHC13/PRKD1/PLEKHG5/S100A4/NOD2/BST2/TNFAIP3/TNFRSF1A/TRAF5/NLRX1/FADD/SPHK1/ADIPOQ	23
**All DMC, Cellular Component**								
	Description	GeneRatio	BgRatio	pvalue	p.adjust	qvalue	geneID	Count

GO:0005575	cellular_component	942/942	16277/17046	3.63E-20	2.48E-17	2.20E-17	AKT3/ABI1/CDH3/TANK/SMIM6/CD300LD/GNE/ZNF783/CCDC180/CDH9/TSPAN5/CDH12/CDH13/SUGP2/MBNL2/FARP1/KLRG1/RCAN2/KCNMB2/CDKN1C/SPEG/BCKDK/TCIRG1/MRVI1/TRDN/ABCA9/SPON2/C1D/COG5/ZBTB18/PITRM1/TACC2/MTHFS/PDPN/DMRT2/CELF1/CELF2/TBR1/SEPT9/GJB6/HCST/NPFFR2/ADCY3/PNRC1/TMED10/SLC27A2/LECT1/RER1/ESM1/ADAM29/HNRNPUL1/RPP14/HIBADH/CHGA/CHI3L1/ERLIN2/PSIP1/CHI3L2/PKP3/EGLN2/PXMP4/ATXN2L/B4GALT7/PTH2/KIF12/ACOT7/EXOC3/CHRNA1/CHRNA2/CHRNA5/GPRIN1/SORCS1/CIDEA/C1QTNF7/GBP4/ALPK2/PANX3/RBP7/GALNT15/AP3S1/CLCA1/C10orf90/FAT3/CLN5/MRPL52/FRMD6/CCR1/SLC51B/C15orf27/SPATA33/ZG16B/SLC38A10/SEZ6/KRT40/TNFAIP8L1/MOB3A/CNP/APOA1BP/NEU4/COL9A3/COL11A1/LYPD6B/GALM/COMP/SCLT1/MAP3K8/ZFP42/ADM/IL31RA/EGFLAM/UBLCP1/HUS1B/C7orf34/OR2A14/CPD/CPM/CPS1/NDUFAF6/PXDNL/CRABP1/ZNF358/TRPM6/MIB2/PARP4/MPP7/LDLRAD3/FAM101A/B3GLCT/CEP128/LYSMD4/MGAT5B/APCDD1/CSTA/KLC3/ZNF738/CTGF/ABCC13/XKR3/SMYD1/SGOL1/PPM1L/LRRC34/SH3D19/CYB561/CYLD/MBOAT1/ADRB3/ANKRD46/ESCO2/CYP11A1/ZNF782/FITM1/ADAL/TRPV3/ZNF709/ZNF781/CALML6/CITED4/DDB1/WBP2NL/DDOST/RNF168/ZNF366/BHLHA15/COCH/PPP1R18/NLRP6/DIO3/DLG2/DMBT1/DNAH6/DNAH8/DNMT3A/ABAT/DPH1/DRD4/DSG3/DTNA/ECE1/AGXT/EEF2/EFNA2/EGFR/EGR3/PATL2/EIF4G1/A2M/ELK4/ANKRD23/TMEM17/LIPH/EML1/UNC13D/DNAH12/SLC10A4/SMIM14/ENO2/ADCK5/EPHA1/EPHA3/EPHB4/ESR1/ALAS1/F11/FAH/FAT2/SPATA13/PRSS54/FCGR2A/RNF182/PHACTR1/SP8/FGA/FGF10/FHIT/XRN2/RASA3/PPM1E/VASH1/BTBD3/SBNO2/TRAK1/MSRB2/ACIN1/FOXL1/FOXC2/TBC1D9B/FOXO1/EXPH5/AKR1B1/SPG20/NFASC/EPB41L3/GGA3/FLNB/DIP2A/FLOT2/MLC1/TBC1D1/RHOBTB2/NUP210/SEL1L3/ATP11A/NEDD4L/SYNE1/PSD3/LARP1/PPP1R13B/PUM2/ARHGEF18/RYBP/MORC3/MAPK8IP2/TSSK2/VGLL2/MTOR/FUCA1/WDR27/SLC37A4/GABBR1/RASGEF1C/RNF144B/ZNF549/CCDC110/ST6GALNAC3/TMEM151A/NUTM1/DSCR9/GAK/SAMM50/DFNB31/ALS2CL/PNKD/SEC31B/TENM4/ACOT11/FAM169A/RAI14/RGS22/STEAP2/GAS2/FBXL21/FBXO2/LCE2B/SACS/GATM/GBGT1/GAPDHS/PLEK2/SLC17A5/ADGRF1/RPS6KC1/PABPC1/AKAP8L/GJA3/DNAJC2/FGF22/NPTN/GJB2/CLUL1/AMPD2/SDCBP2/PDE7B/DKK3/CYTH4/GLS2/VPS4A/AMPD3/GPR162/DHDH/BMP10/ZNF638/GNAS/ZNF311/CRACR2B/TMPRSS12/PIGW/IZUMO1/ZNF844/THEM5/GPR26/GPER1/EOGT/DOK7/DCBLD1/FFAR2/TRIM42/GRB10/MRPS18B/FLVCR1/GRIK4/ZBTB44/DNAJC15/SCG3/GSTP1/GTF2B/BRF1/TMOD4/GUCY1A3/CCDC106/NME7/GPR132/PADI1/GZMA/ANXA2/HAS1/SERPIND1/SOX8/KCNIP2/NRG1/ANXA6/HK1/HLA-B/HLA-DOA/HLA-DPA1/ANXA13/HLA-E/HLA-F/HLX/HMGA1/NR4A1/ACACB/HPCA/APBA2/HOXB3/HOXC4/HOXC5/HOXC6/HOXD3/AGFG2/HRH1/HSD11B1/HSD17B2/ACADL/HSPA1L/HSP90AA1/HSP90AB1/HTR3A/HTR5A/DUPD1/ADAMTSL5/ANKRD45/TFAP2E/ID3/ZC3H12D/COL28A1/RSPO2/MS4A10/FMN1/CD300E/BARHL2/NME9/IGF1/IGF2/CYR61/GPR142/LCE1C/LCE1D/LCE2D/IL1R1/IL1RN/IL6/IL10RA/AQP2/IL11RA/IL12RB2/IL15RA/PRSS41/IL16/FOXK2/AQP5/INHBA/INPP5A/IRF1/AQP9/ISL1/ITGA7/ITGB2/ITGB7/ITIH3/ITIH4/IVL/JUP/CD82/USP50/HILS1/ATP9B/KCP/KCNH2/KCNJ8/KCNJ9/KCNMB1/KDR/ACAT1/KIF25/IPO5/KRT7/KRT15/AMIGO3/INSC/TOMM20L/CLEC17A/HES5/SLC6A17/RESP18/CDHR4/AFF3/LAIR2/LAMA3/STMN1/OR2A5/LCK/LCP1/MUC21/LDLR/ARHGDIA/LGALS9/LHCGR/LLGL1/C11orf87/LMNA/LMO2/RAB19/LOX/LPP/LTB/LTBP1/SMAD3/MC2R/MCC/ME1/ME2/MEF2D/MAP3K1/MEOX1/MEOX2/MFI2/MFNG/MGAT1/SCGB2A1/MITF/LHX8/ASGR1/MOCS1/MOV10/MPZ/IGFL4/MT1A/NUDT1/MYH4/MYL2/NUBP1/NDUFB4/DRG1/NEDD9/NEU1/ATP1A2/NFATC3/NFYB/NHLH2/NMBR/NOV/NPPC/NRAS/NTF3/OAS2/WRAP73/OPRL1/OR2C1/OR3A2/SLC22A18/P2RY6/PAFAH2/ATP5B/IL21R/DEF6/ANO7/PALM/ARHGEF3/PARK2/SPOCK3/BOLA1/UTP11L/LEF1/DDX47/CEND1/CHST15/ANGPT4/PDE4C/PCYOX1/PDE7A/C11orf73/SIRT6/PDE6B/HIGD1B/ATP8A2/GALNT7/PGAM2/PI3/PIGC/PIK3CG/PITX2/PKHD1/PKM/PLA2G2A/PLAGL1/SPA17/LRP1B/PLEC/PRKAG3/PML/RIPPLY3/FXYD6/GPR84/IL20RB/SLCO1C1/PNLIP/RIPK4/TLR9/TREM1/CYTL1/POMC/SSH1/PON1/RIN2/MOV10L1/POU2AF1/ZDHHC13/APBB1IP/ROBO4/MXRA8/FBLIM1/BNC2/MED18/PALMD/CYP2W1/RPP25/LPCAT2/BANP/PPP1CB/FAM118A/HERC6/PPP1CC/PIWIL2/ELP3/ARHGEF10L/PRMT6/DNAJC17/GOLPH3L/ZNF532/PPP2R2B/FANCI/MOB1A/SLC47A1/SLC29A3/MIS18BP1/WDR33/TRPV6/SMPD3/SLC30A10/CNOT11/CHRNA9/SYBU/PEX26/LIMS2/FRMD4A/VAC14/CARKD/PARVA/PRKAR1B/TTC17/IFT122/CFAP44/ERMARD/MCTP2/LMBRD1/CSGALNACT1/PAG1/CISD1/PRKD1/WSB2/MYNN/BIN3/APOBR/MAPK3/MAP2K2/PCDHGC4/PCDHGB7/PCDHGB3/PCDHGA11/PRKRIR/PROC/MRAP/TRPV5/PRMT8/MASP1/HTRA1/SLAMF8/CDC42SE1/PSMB4/PAK6/ARNTL2/RGMA/CEACAM19/PRDM11/TRPC7/LPAR5/PSMD7/SLURP1/ACTR3B/PTGFR/PLEKHG5/TENM2/GATAD2B/ER	942
GO:0005886	plasma membrane	336/942	4464/17046	2.51E-11	5.73E-09	5.07E-09	AKT3/ABI1/CDH3/CD300LD/CDH9/TSPAN5/CDH12/CDH13/FARP1/KLRG1/KCNMB2/TCIRG1/TRDN/PDPN/GJB6/HCST/NPFFR2/ADCY3/TMED10/ADAM29/ERLIN2/PKP3/CHRNA1/CHRNA2/CHRNA5/GPRIN1/PANX3/CLCA1/FAT3/FRMD6/CCR1/SLC51B/C15orf27/SEZ6/CNP/LYPD6B/IL31RA/OR2A14/CPM/TRPM6/MPP7/LDLRAD3/APCDD1/CSTA/CTGF/XKR3/SH3D19/CYLD/ADRB3/TRPV3/NLRP6/DIO3/DLG2/DRD4/DSG3/DTNA/ECE1/EEF2/EFNA2/EGFR/TMEM17/LIPH/SLC10A4/ENO2/EPHA1/EPHA3/EPHB4/ESR1/F11/FAT2/SPATA13/FCGR2A/FGA/FGF10/RASA3/ACIN1/SPG20/NFASC/EPB41L3/FLNB/FLOT2/MLC1/RHOBTB2/ATP11A/NEDD4L/SYNE1/PSD3/PPP1R13B/GABBR1/TENM4/RGS22/STEAP2/LCE2B/PLEK2/SLC17A5/ADGRF1/GJA3/NPTN/GJB2/SDCBP2/CYTH4/VPS4A/GPR162/GNAS/IZUMO1/GPR26/GPER1/DOK7/FFAR2/GRB10/FLVCR1/GRIK4/GSTP1/GUCY1A3/GPR132/GZMA/ANXA2/HAS1/KCNIP2/NRG1/HLA-B/HLA-DOA/HLA-DPA1/ANXA13/HLA-E/HLA-F/HPCA/APBA2/HRH1/HSP90AA1/HSP90AB1/HTR3A/HTR5A/FMN1/CD300E/IGF1/IGF2/GPR142/LCE1C/LCE1D/LCE2D/IL1R1/IL1RN/IL6/IL10RA/AQP2/IL11RA/IL12RB2/PRSS41/IL16/AQP5/INPP5A/AQP9/ITGA7/ITGB2/ITGB7/ITIH4/IVL/JUP/CD82/ATP9B/KCNH2/KCNJ8/KCNJ9/KCNMB1/KDR/SLC6A17/CDHR4/OR2A5/LCK/LCP1/MUC21/LDLR/LHCGR/LLGL1/RAB19/LPP/LTB/SMAD3/MC2R/MCC/MFI2/ASGR1/MPZ/NUBP1/NEU1/ATP1A2/NMBR/NRAS/OPRL1/OR2C1/OR3A2/SLC22A18/P2RY6/ATP5B/DEF6/ANO7/PALM/PCYOX1/PDE6B/ATP8A2/PIK3CG/PKHD1/PKM/SPA17/PLEC/FXYD6/GPR84/IL20RB/SLCO1C1/TLR9/TREM1/SSH1/APBB1IP/BNC2/CYP2W1/FANCI/SLC47A1/TRPV6/SMPD3/SLC30A10/CHRNA9/LIMS2/PARVA/PRKAR1B/TTC17/LMBRD1/PAG1/PRKD1/APOBR/MAPK3/MAP2K2/PCDHGC4/PCDHGB7/PCDHGB3/PCDHGA11/MRAP/TRPV5/PRMT8/CDC42SE1/RGMA/TRPC7/LPAR5/PTGFR/PLEKHG5/TENM2/PTPRCAP/PTPRE/PXN/RASGRF2/TRIM27/RGR/RGS12/RIT2/EXOC4/S100A6/PARVG/NOD2/STRA6/CXCR5/DNAI2/SGK1/CLDN25/CERK/PCDH20/SLC4A1/SLC6A12/SLC8A1/SLC9A3/SLC20A2/BMPR1B/LYNX1/BPI/STAT2/STK10/BST2/VAMP2/TGM2/TLR5/TNFRSF1A/TRAF5/TRPC4/TRPC6/TRPM2/CCR2/TNFRSF4/ZAP70/CACNA1E/PTP4A1/CACNB2/CXCR4/FZD5/CARD14/TMEM204/IGFLR1/CALD1/PSCA/GPR157/ZC3H12A/C6orf25/CALR/CAPS/ANTXR1/SLA2/BFSP2/ATP13A4/YIPF4/CASQ1/PARD6G/PARD6B/SLC43A1/IFITM1/ITPRIP/IRS2/ACTN1/FADD/TNFRSF11A/SPHK1/ENDOU/SKAP2/STBD1/TSPAN18/CCRL2/MAP7/PRC1/SYT7/ESAM/SLC16A3/CD8A/TRIP10/LY86/RAB3D/ENTPD3/RAPGEF2/USP6NL/CD79A	336

GO:0071944	cell periphery	342/942	4563/17046	2.52E-11	5.73E-09	5.07E-09	AKT3/ABI1/CDH3/CD300LD/CDH9/TSPAN5/CDH12/CDH13/FARP1/KLRG1/KCNMB2/TCIRG1/TRDN/PDPN/GJB6/HCST/NPFFR2/ADCY3/TMED10/ADAM29/ERLIN2/PKP3/EXOC3/CHRNA1/CHRNA2/CHRNA5/GPRIN1/PANX3/CLCA1/FAT3/FRMD6/CCR1/SLC51B/C15orf27/SEZ6/CNP/LYPD6B/IL31RA/OR2A14/CPM/TRPM6/MPP7/LDLRAD3/APCDD1/CSTA/CTGF/XKR3/SH3D19/CYLD/ADRB3/TRPV3/NLRP6/DIO3/DLG2/DRD4/DSG3/DTNA/ECE1/EEF2/EFNA2/EGFR/TMEM17/LIPH/SLC10A4/ENO2/EPHA1/EPHA3/EPHB4/ESR1/F11/FAT2/SPATA13/FCGR2A/FGA/FGF10/RASA3/ACIN1/SPG20/NFASC/EPB41L3/FLNB/FLOT2/MLC1/RHOBTB2/ATP11A/NEDD4L/SYNE1/PSD3/PPP1R13B/GABBR1/TENM4/RAI14/RGS22/STEAP2/LCE2B/PLEK2/SLC17A5/ADGRF1/GJA3/NPTN/GJB2/SDCBP2/CYTH4/VPS4A/GPR162/GNAS/IZUMO1/GPR26/GPER1/DOK7/FFAR2/GRB10/FLVCR1/GRIK4/GSTP1/GUCY1A3/GPR132/GZMA/ANXA2/HAS1/KCNIP2/NRG1/HLA-B/HLA-DOA/HLA-DPA1/ANXA13/HLA-E/HLA-F/HPCA/APBA2/HRH1/HSP90AA1/HSP90AB1/HTR3A/HTR5A/FMN1/CD300E/IGF1/IGF2/GPR142/LCE1C/LCE1D/LCE2D/IL1R1/IL1RN/IL6/IL10RA/AQP2/IL11RA/IL12RB2/PRSS41/IL16/AQP5/INPP5A/AQP9/ITGA7/ITGB2/ITGB7/ITIH4/IVL/JUP/CD82/ATP9B/KCNH2/KCNJ8/KCNJ9/KCNMB1/KDR/SLC6A17/CDHR4/OR2A5/LCK/LCP1/MUC21/LDLR/LHCGR/LLGL1/RAB19/LPP/LTB/SMAD3/MC2R/MCC/MFI2/ASGR1/MPZ/NUBP1/NEDD9/NEU1/ATP1A2/NMBR/NRAS/OPRL1/OR2C1/OR3A2/SLC22A18/P2RY6/ATP5B/DEF6/ANO7/PALM/PCYOX1/PDE6B/ATP8A2/PIK3CG/PKHD1/PKM/SPA17/PLEC/FXYD6/GPR84/IL20RB/SLCO1C1/TLR9/TREM1/SSH1/APBB1IP/FBLIM1/BNC2/CYP2W1/FANCI/SLC47A1/TRPV6/SMPD3/SLC30A10/CHRNA9/LIMS2/PARVA/PRKAR1B/TTC17/LMBRD1/PAG1/PRKD1/APOBR/MAPK3/MAP2K2/PCDHGC4/PCDHGB7/PCDHGB3/PCDHGA11/MRAP/TRPV5/PRMT8/CDC42SE1/RGMA/TRPC7/LPAR5/PTGFR/PLEKHG5/TENM2/ERMN/PTPRCAP/PTPRE/PXN/RASGRF2/TRIM27/RGR/RGS12/RIT2/EXOC4/S100A6/PARVG/NOD2/STRA6/CXCR5/DNAI2/SGK1/CLDN25/CERK/PCDH20/SLC4A1/SLC6A12/SLC8A1/SLC9A3/SLC20A2/BMPR1B/LYNX1/BPI/STAT2/STK10/BST2/VAMP2/TGM2/TLR5/TNFRSF1A/TRAF5/TRPC4/TRPC6/TRPM2/CCR2/TNFRSF4/ZAP70/CACNA1E/PTP4A1/CACNB2/CXCR4/FZD5/CARD14/TMEM204/IGFLR1/CALD1/PSCA/GPR157/ZC3H12A/C6orf25/CALR/CAPS/ANTXR1/SLA2/BFSP2/ATP13A4/YIPF4/CASQ1/PARD6G/PARD6B/SLC43A1/IFITM1/ITPRIP/SCIN/IRS2/ACTN1/FADD/TNFRSF11A/SPHK1/ENDOU/SKAP2/STBD1/TSPAN18/CCRL2/MAP7/PRC1/SYT7/ESAM/SLC16A3/CD8A/TRIP10/LY86/RAB3D/ENTPD3/RAPGEF2/USP6NL/CD79A	342
GO:0005623	cell	859/942	14587/17046	5.95E-08	1.02E-05	9.01E-06	AKT3/ABI1/CDH3/TANK/CD300LD/GNE/ZNF783/CDH9/TSPAN5/CDH12/CDH13/SUGP2/MBNL2/FARP1/KLRG1/RCAN2/KCNMB2/CDKN1C/SPEG/BCKDK/TCIRG1/MRVI1/TRDN/ABCA9/C1D/COG5/ZBTB18/PITRM1/TACC2/MTHFS/PDPN/DMRT2/CELF1/CELF2/TBR1/SEPT9/GJB6/HCST/NPFFR2/ADCY3/PNRC1/TMED10/SLC27A2/LECT1/RER1/ADAM29/HNRNPUL1/RPP14/HIBADH/CHGA/CHI3L1/ERLIN2/PSIP1/PKP3/EGLN2/PXMP4/ATXN2L/B4GALT7/KIF12/ACOT7/EXOC3/CHRNA1/CHRNA2/CHRNA5/GPRIN1/CIDEA/GBP4/ALPK2/PANX3/RBP7/GALNT15/AP3S1/CLCA1/C10orf90/FAT3/CLN5/MRPL52/FRMD6/CCR1/SLC51B/C15orf27/SPATA33/SLC38A10/SEZ6/KRT40/TNFAIP8L1/MOB3A/CNP/APOA1BP/NEU4/COL9A3/COL11A1/LYPD6B/GALM/SCLT1/MAP3K8/ZFP42/ADM/IL31RA/UBLCP1/HUS1B/OR2A14/CPM/CPS1/NDUFAF6/PXDNL/CRABP1/ZNF358/TRPM6/MIB2/PARP4/MPP7/LDLRAD3/FAM101A/B3GLCT/CEP128/MGAT5B/APCDD1/CSTA/KLC3/ZNF738/CTGF/XKR3/SMYD1/SGOL1/PPM1L/SH3D19/CYLD/MBOAT1/ADRB3/ESCO2/CYP11A1/ZNF782/FITM1/ADAL/TRPV3/ZNF709/ZNF781/CALML6/CITED4/DDB1/WBP2NL/DDOST/RNF168/ZNF366/BHLHA15/PPP1R18/NLRP6/DIO3/DLG2/DMBT1/DNAH6/DNAH8/DNMT3A/ABAT/DPH1/DRD4/DSG3/DTNA/ECE1/AGXT/EEF2/EFNA2/EGFR/EGR3/PATL2/EIF4G1/A2M/ELK4/ANKRD23/TMEM17/LIPH/EML1/UNC13D/DNAH12/SLC10A4/SMIM14/ENO2/ADCK5/EPHA1/EPHA3/EPHB4/ESR1/ALAS1/F11/FAH/FAT2/SPATA13/PRSS54/FCGR2A/RNF182/PHACTR1/SP8/FGA/FGF10/FHIT/XRN2/RASA3/PPM1E/VASH1/BTBD3/TRAK1/MSRB2/ACIN1/FOXL1/FOXC2/FOXO1/EXPH5/AKR1B1/SPG20/NFASC/EPB41L3/GGA3/FLNB/DIP2A/FLOT2/MLC1/TBC1D1/RHOBTB2/NUP210/SEL1L3/ATP11A/NEDD4L/SYNE1/PSD3/LARP1/PPP1R13B/PUM2/ARHGEF18/RYBP/MORC3/MAPK8IP2/TSSK2/VGLL2/MTOR/FUCA1/WDR27/SLC37A4/GABBR1/RASGEF1C/RNF144B/ZNF549/CCDC110/ST6GALNAC3/NUTM1/GAK/SAMM50/DFNB31/ALS2CL/PNKD/SEC31B/TENM4/ACOT11/FAM169A/RAI14/RGS22/STEAP2/GAS2/FBXL21/FBXO2/LCE2B/SACS/GATM/GBGT1/GAPDHS/PLEK2/SLC17A5/ADGRF1/RPS6KC1/PABPC1/AKAP8L/GJA3/DNAJC2/FGF22/NPTN/GJB2/AMPD2/SDCBP2/PDE7B/CYTH4/GLS2/VPS4A/AMPD3/GPR162/DHDH/BMP10/ZNF638/GNAS/ZNF311/CRACR2B/PIGW/IZUMO1/ZNF844/THEM5/GPR26/GPER1/EOGT/DOK7/FFAR2/TRIM42/GRB10/MRPS18B/FLVCR1/GRIK4/ZBTB44/DNAJC15/SCG3/GSTP1/GTF2B/BRF1/TMOD4/GUCY1A3/CCDC106/NME7/GPR132/PADI1/GZMA/ANXA2/HAS1/SOX8/KCNIP2/NRG1/ANXA6/HK1/HLA-B/HLA-DOA/HLA-DPA1/ANXA13/HLA-E/HLA-F/HLX/HMGA1/NR4A1/ACACB/HPCA/APBA2/HOXB3/HOXC4/HOXC5/HOXC6/HOXD3/HRH1/HSD11B1/HSD17B2/ACADL/HSPA1L/HSP90AA1/HSP90AB1/HTR3A/HTR5A/DUPD1/TFAP2E/ID3/ZC3H12D/COL28A1/RSPO2/FMN1/CD300E/BARHL2/NME9/IGF1/IGF2/GPR142/LCE1C/LCE1D/LCE2D/IL1R1/IL1RN/IL6/IL10RA/AQP2/IL11RA/IL12RB2/IL15RA/PRSS41/IL16/FOXK2/AQP5/INHBA/INPP5A/IRF1/AQP9/ISL1/ITGA7/ITGB2/ITGB7/ITIH4/IVL/JUP/CD82/USP50/HILS1/ATP9B/KCNH2/KCNJ8/KCNJ9/KCNMB1/KDR/ACAT1/KIF25/IPO5/KRT7/KRT15/INSC/TOMM20L/CLEC17A/HES5/SLC6A17/RESP18/CDHR4/AFF3/STMN1/OR2A5/LCK/LCP1/MUC21/LDLR/ARHGDIA/LGALS9/LHCGR/LLGL1/LMNA/LMO2/RAB19/LOX/LPP/LTB/SMAD3/MC2R/MCC/ME1/ME2/MEF2D/MAP3K1/MEOX1/MEOX2/MFI2/MFNG/MGAT1/MITF/LHX8/ASGR1/MOCS1/MOV10/MPZ/MT1A/NUDT1/MYH4/MYL2/NUBP1/NDUFB4/DRG1/NEDD9/NEU1/ATP1A2/NFATC3/NFYB/NHLH2/NMBR/NOV/NPPC/NRAS/NTF3/OAS2/WRAP73/OPRL1/OR2C1/OR3A2/SLC22A18/P2RY6/PAFAH2/ATP5B/DEF6/ANO7/PALM/ARHGEF3/PARK2/BOLA1/UTP11L/LEF1/DDX47/CHST15/PDE4C/PCYOX1/PDE7A/C11orf73/SIRT6/PDE6B/ATP8A2/GALNT7/PGAM2/PIGC/PIK3CG/PITX2/PKHD1/PKM/PLA2G2A/PLAGL1/SPA17/PLEC/PRKAG3/PML/RIPPLY3/FXYD6/GPR84/IL20RB/SLCO1C1/RIPK4/TLR9/TREM1/POMC/SSH1/PON1/RIN2/MOV10L1/POU2AF1/ZDHHC13/APBB1IP/MXRA8/FBLIM1/BNC2/MED18/PALMD/CYP2W1/RPP25/LPCAT2/BANP/PPP1CB/HERC6/PPP1CC/PIWIL2/ELP3/ARHGEF10L/PRMT6/DNAJC17/GOLPH3L/ZNF532/PPP2R2B/FANCI/MOB1A/SLC47A1/SLC29A3/MIS18BP1/WDR33/TRPV6/SMPD3/SLC30A10/CNOT11/CHRNA9/SYBU/PEX26/LIMS2/FRMD4A/VAC14/CARKD/PARVA/PRKAR1B/TTC17/IFT122/CFAP44/ERMARD/MCTP2/LMBRD1/CSGALNACT1/PAG1/CISD1/PRKD1/WSB2/MYNN/BIN3/APOBR/MAPK3/MAP2K2/PCDHGC4/PCDHGB7/PCDHGB3/PCDHGA11/PRKRIR/PROC/MRAP/TRPV5/PRMT8/HTRA1/SLAMF8/CDC42SE1/PSMB4/PAK6/ARNTL2/RGMA/PRDM11/TRPC7/LPAR5/PSMD7/ACTR3B/PTGFR/PLEKHG5/TENM2/GATAD2B/ERMN/KLHL8/RDH14/METTL14/MARK4/CCAR2/PTPRCAP/PTPRE/PXN/CREBZF/FAM60A/ACTA2/RASGRF2/RFC2/TRIM27/RGR/RGS12/RIT2/EXOC4/RPA3/RPL8/DEFB134/RPL29/S100A4/S100A5/S100A6/BGLAP/CCL11/ABHD4/MRPS14/NPAS3/C19orf33/PARVG/NOD2/STRA6/CXCR5/MAP1LC3B2/ARHGAP9/TRA2B/GZF1/DNAI2/SGK1/CLDN25/SYNDIG1L/MICAL1/CERK/PCDH20/TMEM237/VPS33A/BMP4/SLC4A1	859

GO:0044464	cell part	856/942	14556/17046	1.50E-07	2.05E-05	1.81E-05	AKT3/ABI1/CDH3/TANK/CD300LD/GNE/ZNF783/CDH9/TSPAN5/CDH12/CDH13/SUGP2/MBNL2/FARP1/KLRG1/RCAN2/KCNMB2/CDKN1C/SPEG/BCKDK/TCIRG1/MRVI1/TRDN/ABCA9/C1D/COG5/ZBTB18/PITRM1/TACC2/MTHFS/PDPN/DMRT2/CELF1/CELF2/TBR1/SEPT9/GJB6/HCST/NPFFR2/ADCY3/PNRC1/TMED10/SLC27A2/LECT1/RER1/ADAM29/HNRNPUL1/RPP14/HIBADH/CHGA/CHI3L1/ERLIN2/PSIP1/PKP3/EGLN2/PXMP4/ATXN2L/B4GALT7/KIF12/ACOT7/EXOC3/CHRNA1/CHRNA2/CHRNA5/GPRIN1/CIDEA/GBP4/ALPK2/PANX3/RBP7/GALNT15/AP3S1/CLCA1/C10orf90/FAT3/CLN5/MRPL52/FRMD6/CCR1/SLC51B/C15orf27/SPATA33/SLC38A10/SEZ6/KRT40/TNFAIP8L1/MOB3A/CNP/APOA1BP/NEU4/COL9A3/COL11A1/LYPD6B/GALM/SCLT1/MAP3K8/ZFP42/ADM/IL31RA/UBLCP1/HUS1B/OR2A14/CPM/CPS1/NDUFAF6/PXDNL/CRABP1/ZNF358/TRPM6/MIB2/PARP4/MPP7/LDLRAD3/FAM101A/B3GLCT/CEP128/MGAT5B/APCDD1/CSTA/KLC3/ZNF738/CTGF/XKR3/SMYD1/SGOL1/PPM1L/SH3D19/CYLD/MBOAT1/ADRB3/ESCO2/CYP11A1/ZNF782/FITM1/ADAL/TRPV3/ZNF709/ZNF781/CALML6/CITED4/DDB1/WBP2NL/DDOST/RNF168/ZNF366/BHLHA15/PPP1R18/NLRP6/DIO3/DLG2/DMBT1/DNAH6/DNAH8/DNMT3A/ABAT/DPH1/DRD4/DSG3/DTNA/ECE1/AGXT/EEF2/EFNA2/EGFR/EGR3/PATL2/EIF4G1/A2M/ELK4/ANKRD23/TMEM17/LIPH/EML1/UNC13D/DNAH12/SLC10A4/SMIM14/ENO2/ADCK5/EPHA1/EPHA3/EPHB4/ESR1/ALAS1/F11/FAH/FAT2/SPATA13/PRSS54/FCGR2A/RNF182/PHACTR1/SP8/FGA/FGF10/FHIT/XRN2/RASA3/PPM1E/VASH1/BTBD3/TRAK1/MSRB2/ACIN1/FOXL1/FOXC2/FOXO1/EXPH5/AKR1B1/SPG20/NFASC/EPB41L3/GGA3/FLNB/DIP2A/FLOT2/MLC1/TBC1D1/RHOBTB2/NUP210/SEL1L3/ATP11A/NEDD4L/SYNE1/PSD3/LARP1/PPP1R13B/PUM2/ARHGEF18/RYBP/MORC3/MAPK8IP2/TSSK2/VGLL2/MTOR/FUCA1/WDR27/SLC37A4/GABBR1/RASGEF1C/RNF144B/ZNF549/CCDC110/ST6GALNAC3/NUTM1/GAK/SAMM50/DFNB31/ALS2CL/PNKD/SEC31B/TENM4/ACOT11/FAM169A/RAI14/RGS22/STEAP2/GAS2/FBXL21/FBXO2/LCE2B/SACS/GATM/GBGT1/GAPDHS/PLEK2/SLC17A5/ADGRF1/RPS6KC1/PABPC1/AKAP8L/GJA3/DNAJC2/FGF22/NPTN/GJB2/AMPD2/SDCBP2/PDE7B/CYTH4/GLS2/VPS4A/AMPD3/GPR162/DHDH/BMP10/ZNF638/GNAS/ZNF311/CRACR2B/PIGW/IZUMO1/ZNF844/THEM5/GPR26/GPER1/EOGT/DOK7/FFAR2/TRIM42/GRB10/MRPS18B/FLVCR1/GRIK4/ZBTB44/DNAJC15/SCG3/GSTP1/GTF2B/BRF1/TMOD4/GUCY1A3/CCDC106/NME7/GPR132/PADI1/GZMA/ANXA2/HAS1/SOX8/KCNIP2/NRG1/ANXA6/HK1/HLA-B/HLA-DOA/HLA-DPA1/ANXA13/HLA-E/HLA-F/HLX/HMGA1/NR4A1/ACACB/HPCA/APBA2/HOXB3/HOXC4/HOXC5/HOXC6/HOXD3/HRH1/HSD11B1/HSD17B2/ACADL/HSPA1L/HSP90AA1/HSP90AB1/HTR3A/HTR5A/DUPD1/TFAP2E/ID3/ZC3H12D/COL28A1/RSPO2/FMN1/CD300E/BARHL2/NME9/IGF1/IGF2/GPR142/LCE1C/LCE1D/LCE2D/IL1R1/IL1RN/IL6/IL10RA/AQP2/IL11RA/IL12RB2/IL15RA/PRSS41/IL16/FOXK2/AQP5/INPP5A/IRF1/AQP9/ISL1/ITGA7/ITGB2/ITGB7/ITIH4/IVL/JUP/CD82/USP50/HILS1/ATP9B/KCNH2/KCNJ8/KCNJ9/KCNMB1/KDR/ACAT1/KIF25/IPO5/KRT7/KRT15/INSC/TOMM20L/CLEC17A/HES5/SLC6A17/RESP18/CDHR4/AFF3/STMN1/OR2A5/LCK/LCP1/MUC21/LDLR/ARHGDIA/LGALS9/LHCGR/LLGL1/LMNA/LMO2/RAB19/LOX/LPP/LTB/SMAD3/MC2R/MCC/ME1/ME2/MEF2D/MAP3K1/MEOX1/MEOX2/MFI2/MFNG/MGAT1/MITF/LHX8/ASGR1/MOCS1/MOV10/MPZ/MT1A/NUDT1/MYH4/MYL2/NUBP1/NDUFB4/DRG1/NEDD9/NEU1/ATP1A2/NFATC3/NFYB/NHLH2/NMBR/NOV/NPPC/NRAS/NTF3/OAS2/WRAP73/OPRL1/OR2C1/OR3A2/SLC22A18/P2RY6/PAFAH2/ATP5B/DEF6/ANO7/PALM/ARHGEF3/PARK2/BOLA1/UTP11L/LEF1/DDX47/CHST15/PDE4C/PCYOX1/PDE7A/C11orf73/SIRT6/PDE6B/ATP8A2/GALNT7/PGAM2/PIGC/PIK3CG/PITX2/PKHD1/PKM/PLA2G2A/PLAGL1/SPA17/PLEC/PRKAG3/PML/RIPPLY3/FXYD6/GPR84/IL20RB/SLCO1C1/RIPK4/TLR9/TREM1/POMC/SSH1/PON1/RIN2/MOV10L1/POU2AF1/ZDHHC13/APBB1IP/MXRA8/FBLIM1/BNC2/MED18/PALMD/CYP2W1/RPP25/LPCAT2/BANP/PPP1CB/HERC6/PPP1CC/PIWIL2/ELP3/ARHGEF10L/PRMT6/DNAJC17/GOLPH3L/ZNF532/PPP2R2B/FANCI/MOB1A/SLC47A1/SLC29A3/MIS18BP1/WDR33/TRPV6/SMPD3/SLC30A10/CNOT11/CHRNA9/SYBU/PEX26/LIMS2/FRMD4A/VAC14/CARKD/PARVA/PRKAR1B/TTC17/IFT122/CFAP44/ERMARD/MCTP2/LMBRD1/CSGALNACT1/PAG1/CISD1/PRKD1/WSB2/MYNN/BIN3/APOBR/MAPK3/MAP2K2/PCDHGC4/PCDHGB7/PCDHGB3/PCDHGA11/PRKRIR/PROC/MRAP/TRPV5/PRMT8/HTRA1/SLAMF8/CDC42SE1/PSMB4/PAK6/ARNTL2/RGMA/PRDM11/TRPC7/LPAR5/PSMD7/ACTR3B/PTGFR/PLEKHG5/TENM2/GATAD2B/ERMN/KLHL8/RDH14/METTL14/MARK4/CCAR2/PTPRCAP/PTPRE/PXN/CREBZF/FAM60A/ACTA2/RASGRF2/RFC2/TRIM27/RGR/RGS12/RIT2/EXOC4/RPA3/RPL8/DEFB134/RPL29/S100A4/S100A5/S100A6/BGLAP/CCL11/ABHD4/MRPS14/NPAS3/C19orf33/PARVG/NOD2/STRA6/CXCR5/MAP1LC3B2/ARHGAP9/TRA2B/GZF1/DNAI2/SGK1/CLDN25/SYNDIG1L/MICAL1/CERK/PCDH20/TMEM237/VPS33A/BMP4/SLC4A1/SPATS2/	856

GO:0016020	membrane	522/942	8177/17046	1.50E-06	0.00016	0.00014	AKT3/ABI1/CDH3/SMIM6/CD300LD/CCDC180/CDH9/TSPAN5/CDH12/CDH13/FARP1/KLRG1/KCNMB2/TCIRG1/MRVI1/TRDN/ABCA9/COG5/PDPN/CELF1/GJB6/HCST/NPFFR2/ADCY3/TMED10/SLC27A2/LECT1/RER1/ADAM29/CHGA/ERLIN2/PKP3/PXMP4/ATXN2L/B4GALT7/EXOC3/CHRNA1/CHRNA2/CHRNA5/GPRIN1/SORCS1/PANX3/GALNT15/AP3S1/CLCA1/FAT3/CLN5/MRPL52/FRMD6/CCR1/SLC51B/C15orf27/SLC38A10/SEZ6/CNP/NEU4/LYPD6B/SCLT1/IL31RA/OR2A14/CPD/CPM/CPS1/NDUFAF6/TRPM6/PARP4/MPP7/LDLRAD3/B3GLCT/LYSMD4/MGAT5B/APCDD1/CSTA/CTGF/ABCC13/XKR3/PPM1L/SH3D19/CYB561/CYLD/MBOAT1/ADRB3/ANKRD46/CYP11A1/FITM1/TRPV3/DDOST/NLRP6/DIO3/DLG2/DMBT1/DRD4/DSG3/DTNA/ECE1/EEF2/EFNA2/EGFR/EIF4G1/TMEM17/LIPH/UNC13D/SLC10A4/SMIM14/ENO2/ADCK5/EPHA1/EPHA3/EPHB4/ESR1/F11/FAT2/SPATA13/FCGR2A/RNF182/FGA/FGF10/XRN2/RASA3/ACIN1/TBC1D9B/SPG20/NFASC/EPB41L3/GGA3/FLNB/FLOT2/MLC1/RHOBTB2/NUP210/SEL1L3/ATP11A/NEDD4L/SYNE1/PSD3/LARP1/PPP1R13B/PUM2/MTOR/SLC37A4/GABBR1/RNF144B/ST6GALNAC3/TMEM151A/GAK/SAMM50/PNKD/SEC31B/TENM4/FAM169A/RGS22/STEAP2/GAS2/FBXO2/LCE2B/GATM/GBGT1/PLEK2/SLC17A5/ADGRF1/RPS6KC1/PABPC1/GJA3/DNAJC2/NPTN/GJB2/SDCBP2/CYTH4/VPS4A/GPR162/GNAS/TMPRSS12/PIGW/IZUMO1/GPR26/GPER1/DOK7/DCBLD1/FFAR2/GRB10/MRPS18B/FLVCR1/GRIK4/DNAJC15/SCG3/GSTP1/GUCY1A3/GPR132/GZMA/ANXA2/HAS1/KCNIP2/NRG1/ANXA6/HK1/HLA-B/HLA-DOA/HLA-DPA1/ANXA13/HLA-E/HLA-F/NR4A1/ACACB/HPCA/APBA2/AGFG2/HRH1/HSD11B1/HSD17B2/ACADL/HSP90AA1/HSP90AB1/HTR3A/HTR5A/MS4A10/FMN1/CD300E/IGF1/IGF2/GPR142/LCE1C/LCE1D/LCE2D/IL1R1/IL1RN/IL6/IL10RA/AQP2/IL11RA/IL12RB2/IL15RA/PRSS41/IL16/AQP5/INPP5A/AQP9/ITGA7/ITGB2/ITGB7/ITIH4/IVL/JUP/CD82/ATP9B/KCNH2/KCNJ8/KCNJ9/KCNMB1/KDR/ACAT1/IPO5/AMIGO3/TOMM20L/CLEC17A/SLC6A17/CDHR4/STMN1/OR2A5/LCK/LCP1/MUC21/LDLR/LHCGR/LLGL1/C11orf87/LMNA/RAB19/LPP/LTB/SMAD3/MC2R/MCC/MFI2/MFNG/MGAT1/ASGR1/MPZ/NUBP1/NDUFB4/DRG1/NEU1/ATP1A2/NMBR/NRAS/OAS2/OPRL1/OR2C1/OR3A2/SLC22A18/P2RY6/ATP5B/IL21R/DEF6/ANO7/PALM/DDX47/CEND1/CHST15/PCYOX1/PDE6B/HIGD1B/ATP8A2/GALNT7/PIGC/PIK3CG/PKHD1/PKM/PLA2G2A/SPA17/LRP1B/PLEC/PML/FXYD6/GPR84/IL20RB/SLCO1C1/RIPK4/TLR9/TREM1/SSH1/ZDHHC13/APBB1IP/ROBO4/MXRA8/BNC2/PALMD/CYP2W1/LPCAT2/FAM118A/GOLPH3L/PPP2R2B/FANCI/SLC47A1/SLC29A3/TRPV6/SMPD3/SLC30A10/CHRNA9/SYBU/PEX26/LIMS2/VAC14/PARVA/PRKAR1B/TTC17/IFT122/ERMARD/MCTP2/LMBRD1/CSGALNACT1/PAG1/CISD1/PRKD1/APOBR/MAPK3/MAP2K2/PCDHGC4/PCDHGB7/PCDHGB3/PCDHGA11/MRAP/TRPV5/PRMT8/SLAMF8/CDC42SE1/RGMA/CEACAM19/TRPC7/LPAR5/PSMD7/PTGFR/PLEKHG5/TENM2/RNF150/RDH14/PTPRCAP/PTPRE/PXN/RASGRF2/TRIM27/RGR/RGS12/RIT2/EXOC4/RPL8/CTXN3/RPL29/S100A6/MRPS14/PARVG/NOD2/STRA6/CXCR5/MAP1LC3B2/DNAI2/SGK1/CLDN25/SYNDIG1L/CERK/PCDH20/TMEM237/VPS33A/SLC4A1/ZG16/SLC6A12/SLC8A1/SLC9A3/SLC20A2/BMPR1B/TMEM108/LYNX1/BOK/BPI/STAT2/STK10/BST2/VAMP2/ACTC1/TGM2/TLR5/TRAPPC10/TNFRSF1A/TRAF5/TRPC4/TRPC6/TRPM2/PHLDA2/CCR2/TNFRSF4/UCP1/YWHAG/ZAP70/CACNA1E/PTP4A1/CACNB2/MOGS/CXCR4/FZD5/RAB7A/REEP5/CARD14/GDPD3/BCL2L14/LST1/CERS4/TMEM204/NLRX1/IGFLR1/ERMP1/CALD1/PSCA/FAM188A/TMEM62/GPR157/ZC3H12A/RAB11FIP1/C6orf25/CLPTM1L/CALR/UNC93B1/CAPS/CAST/GSG1/HIST1H3A/SLC25A18/ANTXR1/SLA2/MFSD7/CMAHP/BFSP2/ATP13A4/YIPF4/CASQ1/PARD6G/FAXC/PARD6B/LOXL3/MGARP/RAE1/SLC43A1/IFITM1/ITPRIP/KMO/IRS2/ACTN1/FADD/TNFRSF11A/SYNJ2/SPHK1/ENDOU/SKAP2/STBD1/SLAMF9/TSPAN18/CH25H/CCRL2/MAP7/PRC1/STARD13/SYT7/ESAM/SLC16A3/RSAD2/SMDT1/IL32/CD8A/REEP6/TRIP10/SDR42E1/LY86/RAB3D/ENTPD3/RAB36/NUP93/RAPGEF2/ULK2/USP6NL/CD79A/TELO2/TMCC2/IQSEC1/LPGAT1	522
GO:0030054	cell junction	96/942	1073/17046	1.62E-06	0.00016	0.00014	ABI1/CDH3/CDH13/FARP1/GJB6/PKP3/CHRNA1/CHRNA2/CHRNA5/PANX3/FRMD6/EGFLAM/MPP7/LDLRAD3/DLG2/DSG3/DTNA/EGFR/ANKRD23/FAT2/PHACTR1/AKR1B1/NFASC/EPB41L3/FLNB/FLOT2/PSD3/ARHGEF18/GABBR1/GAK/SLC17A5/PABPC1/GJA3/GJB2/GPER1/DOK7/MRPS18B/GRIK4/ANXA6/HMGA1/HOXC5/HTR3A/FMN1/GPR142/ITGA7/JUP/KDR/SLC6A17/LCK/LCP1/LPP/NEDD9/NEU1/ATP1A2/NOV/ANO7/C11orf73/PLEC/APBB1IP/FBLIM1/PPP1CB/PPP1CC/CHRNA9/LIMS2/FRMD4A/PARVA/PRKD1/MAPK3/MAP2K2/PLEKHG5/TENM2/PXN/RPL8/PARVG/CLDN25/SLC8A1/SRP68/VAMP2/ACTC1/TGM2/TRPC4/TRPC6/YWHAG/ZAP70/CXCR4/FZD5/TMEM204/CALR/COLQ/PARD6G/PARD6B/ACTN1/LIMD1/SYT7/ESAM/RAPGEF2	96

GO:0005737	cytoplasm	604/942	9735/17046	3.88E-06	0.00033	0.00029	AKT3/ABI1/CDH3/TANK/GNE/CDH13/MBNL2/FARP1/RCAN2/CDKN1C/BCKDK/TCIRG1/MRVI1/TRDN/ABCA9/C1D/COG5/PITRM1/TACC2/MTHFS/CELF1/CELF2/SEPT9/GJB6/ADCY3/TMED10/SLC27A2/RER1/HIBADH/CHGA/CHI3L1/ERLIN2/PSIP1/EGLN2/PXMP4/ATXN2L/B4GALT7/KIF12/ACOT7/EXOC3/CIDEA/GBP4/RBP7/GALNT15/AP3S1/CLCA1/C10orf90/CLN5/MRPL52/FRMD6/CCR1/SPATA33/SLC38A10/SEZ6/TNFAIP8L1/CNP/APOA1BP/NEU4/COL9A3/COL11A1/GALM/SCLT1/MAP3K8/ZFP42/ADM/CPS1/NDUFAF6/PXDNL/CRABP1/MIB2/PARP4/MPP7/FAM101A/B3GLCT/CEP128/MGAT5B/CSTA/KLC3/CTGF/SMYD1/SGOL1/PPM1L/SH3D19/CYLD/MBOAT1/ESCO2/CYP11A1/FITM1/ADAL/CALML6/CITED4/DDB1/WBP2NL/DDOST/RNF168/PPP1R18/NLRP6/DIO3/DMBT1/DNAH6/DNAH8/DNMT3A/ABAT/DPH1/DRD4/DSG3/DTNA/ECE1/AGXT/EEF2/EGFR/EGR3/PATL2/EIF4G1/A2M/ELK4/ANKRD23/EML1/UNC13D/DNAH12/SMIM14/ENO2/ADCK5/EPHA3/EPHB4/ESR1/ALAS1/FAH/SPATA13/PRSS54/RNF182/PHACTR1/FGA/FHIT/RASA3/PPM1E/VASH1/BTBD3/TRAK1/MSRB2/ACIN1/FOXO1/EXPH5/AKR1B1/SPG20/EPB41L3/GGA3/FLNB/FLOT2/MLC1/TBC1D1/RHOBTB2/NUP210/ATP11A/NEDD4L/SYNE1/LARP1/PPP1R13B/PUM2/ARHGEF18/RYBP/MAPK8IP2/TSSK2/VGLL2/MTOR/FUCA1/SLC37A4/GABBR1/RNF144B/CCDC110/ST6GALNAC3/NUTM1/GAK/SAMM50/DFNB31/ALS2CL/PNKD/SEC31B/TENM4/ACOT11/RAI14/RGS22/STEAP2/GAS2/FBXL21/FBXO2/LCE2B/SACS/GATM/GBGT1/GAPDHS/PLEK2/SLC17A5/RPS6KC1/PABPC1/AKAP8L/DNAJC2/FGF22/NPTN/GJB2/AMPD2/SDCBP2/PDE7B/GLS2/VPS4A/AMPD3/DHDH/BMP10/ZNF638/GNAS/CRACR2B/PIGW/IZUMO1/THEM5/GPER1/EOGT/GRB10/MRPS18B/FLVCR1/DNAJC15/SCG3/GSTP1/TMOD4/GUCY1A3/CCDC106/PADI1/ANXA2/HAS1/SOX8/KCNIP2/NRG1/ANXA6/HK1/HLA-B/HLA-DOA/HLA-DPA1/ANXA13/HLA-E/HLA-F/HMGA1/NR4A1/ACACB/HPCA/APBA2/HOXC6/HRH1/HSD11B1/HSD17B2/ACADL/HSPA1L/HSP90AA1/HSP90AB1/HTR3A/HTR5A/DUPD1/ID3/ZC3H12D/COL28A1/FMN1/BARHL2/NME9/IGF1/IGF2/GPR142/LCE1C/LCE1D/LCE2D/IL1RN/IL6/AQP2/IL15RA/IL16/AQP5/IRF1/ISL1/ITGA7/ITIH4/IVL/JUP/ATP9B/KCNH2/KCNJ8/KDR/ACAT1/KIF25/IPO5/KRT7/INSC/TOMM20L/SLC6A17/RESP18/AFF3/STMN1/LCK/LCP1/MUC21/LDLR/ARHGDIA/LGALS9/LHCGR/LLGL1/LMNA/RAB19/LPP/SMAD3/MC2R/MCC/ME1/ME2/MEF2D/MAP3K1/MEOX1/MEOX2/MFNG/MGAT1/MITF/MOCS1/MOV10/MPZ/MT1A/NUDT1/MYH4/MYL2/NUBP1/NDUFB4/DRG1/NEDD9/NEU1/ATP1A2/NFATC3/NMBR/NOV/NPPC/NRAS/NTF3/OAS2/WRAP73/OPRL1/OR2C1/SLC22A18/PAFAH2/ATP5B/DEF6/ANO7/PALM/ARHGEF3/PARK2/BOLA1/UTP11L/LEF1/CHST15/PDE4C/PCYOX1/PDE7A/C11orf73/SIRT6/PDE6B/ATP8A2/GALNT7/PGAM2/PIGC/PIK3CG/PITX2/PKHD1/PKM/PLA2G2A/PLAGL1/SPA17/PLEC/PRKAG3/PML/RIPK4/TLR9/POMC/SSH1/RIN2/ZDHHC13/APBB1IP/FBLIM1/BNC2/PALMD/CYP2W1/LPCAT2/PPP1CB/HERC6/PPP1CC/PIWIL2/ELP3/ARHGEF10L/PRMT6/DNAJC17/GOLPH3L/PPP2R2B/FANCI/MOB1A/SLC29A3/SMPD3/CNOT11/CHRNA9/SYBU/PEX26/LIMS2/FRMD4A/VAC14/CARKD/PARVA/PRKAR1B/TTC17/IFT122/ERMARD/LMBRD1/CSGALNACT1/CISD1/PRKD1/BIN3/MAPK3/MAP2K2/PROC/MRAP/PRMT8/HTRA1/CDC42SE1/PSMB4/PAK6/ARNTL2/RGMA/PRDM11/TRPC7/PSMD7/ACTR3B/PTGFR/PLEKHG5/TENM2/ERMN/RDH14/METTL14/MARK4/CCAR2/PTPRE/PXN/ACTA2/RASGRF2/RFC2/TRIM27/RGS12/EXOC4/RPL8/RPL29/S100A4/S100A6/BGLAP/MRPS14/NPAS3/PARVG/NOD2/MAP1LC3B2/ARHGAP9/GZF1/DNAI2/SGK1/SYNDIG1L/MICAL1/CERK/VPS33A/BMP4/SLC4A1/SPATS2/ZG16/SLC8A1/BOK/BPI/SRP68/STAT2/STK3/STK10/BST2/VAMP2/TAF4B/TBP/TCEB2/ZEB1/ACTC1/TERF1/TGM2/TIMP3/TRAPPC10/TNFAIP3/TNFRSF1A/TRAF1/TRAF5/TRPC4/TRPC6/PHLDA2/CCR2/UCP1/UPP1/VARS/YWHAG/ZAP70/CA7/PTP4A1/MOGS/CXCR4/FZD5/RAB7A/ERI3/CARD14/BCL2L14/LST1/CERS4/NLRX1/CSPP1/ZC3H14/ERMP1/CALD1/ZNF606/ZC3H12A/RAB11FIP1/FAAP100/CPEB4/RNF39/C6orf25/COL18A1/ZNF436/CALR/COL21A1/UNC93B1/QTRT1/SLIRP/CAPS/CAST/CAPZB/SH3BGRL3/GSG1/CCDC3/DYNLRB2/SLC25A18/SPATA16/ANTXR1/SLA2/CMAHP/BFSP2/ATP13A4/YIPF4/ZNF397/NR0B2/CASQ1/PARD6G/PARD6B/TTBK1/TRIM63/LOXL3/GTPBP3/MGARP/RAE1/C1orf198/GAS7/SCIN/KMO/RUNX1/TP63/RUNX3/IRS2/ACTN1/CRADD/FADD/ALDH1A2/SYNJ2/SPHK1/CCNA1/ENDOU/SKAP2/STBD1/HSPB3/LIMD1/CH25H/ERI1/MAP7/PRC1/STARD13/ZFAND2A/MAP3K6/SYT7/RSAD2/SMDT1/AURKB/REEP6/TRIP10/ADIPOQ/ARHGAP29/RAB3D/PREPL/RAB36/MTL5/ARHGEF10/RAPGEF2/ULK2/USP6NL/CD79A/KIAA0513/DAZAP2/TELO2/RABGAP1L/LPGAT1	604
GO:0005615	extracellular space	102/942	1213/17046	1.15E-05	0.00088	0.00078	CDH13/SPON2/CHI3L1/CHI3L2/C1QTNF7/CLCA1/ZG16B/SEZ6/CNP/APOA1BP/COMP/ADM/PXDNL/CSTA/CTGF/DDB1/DMBT1/EGFR/A2M/LIPH/ENO2/F11/FGA/FGF10/VASH1/AKR1B1/FGF22/DKK3/BMP10/GSTP1/ANXA2/SERPIND1/NRG1/ANXA13/HSPA1L/RSPO2/IGF1/IGF2/IL1R1/IL1RN/IL6/IL15RA/IL16/INHBA/ITIH4/LCP1/LDLR/LGALS9/LOX/LTB/MFI2/MFNG/SCGB2A1/MOV10/IGFL4/NPPC/SPOCK3/UTP11L/ANGPT4/PDE4C/PCYOX1/PLA2G2A/PRKAG3/CYTL1/POMC/PON1/APOBR/PROC/MASP1/HTRA1/SLURP1/ACTA2/S100A4/BGLAP/SCT/CCL11/CCL17/TINAGL1/SFRP2/BMP4/SLC4A1/ZNF649/SLIT1/BPI/ACTC1/TIMP3/TNFRSF1A/TNXB/WNT10B/ZNF177/COL18A1/CALR/COLQ/IL1F10/LOXL3/PPFIBP2/SERPINA6/ACTN1/ENDOU/IL32/ADIPOQ/LY86	102
GO:0044421	extracellular region part	238/942	3516/17046	0.00023	0.01579	0.01397	ABI1/CCDC180/CDH13/SPON2/ZBTB18/TMED10/SLC27A2/LECT1/CHI3L1/ERLIN2/CHI3L2/KIF12/ACOT7/C1QTNF7/CLCA1/CLN5/ZG16B/SEZ6/CNP/APOA1BP/COL9A3/COL11A1/GALM/COMP/SCLT1/ADM/EGFLAM/CPD/CPM/PXDNL/PARP4/CSTA/CTGF/PPM1L/DDB1/COCH/DMBT1/ABAT/DSG3/ECE1/EEF2/EGFR/A2M/LIPH/ENO2/EPHB4/F11/FAH/FAT2/FCGR2A/FGA/FGF10/FHIT/VASH1/AKR1B1/NFASC/FLNB/FLOT2/NEDD4L/ARHGEF18/FUCA1/SAMM50/ACOT11/FBXO2/GATM/PABPC1/FGF22/SDCBP2/DKK3/VPS4A/BMP10/GNAS/GSTP1/PADI1/ANXA2/SERPIND1/NRG1/ANXA6/HLA-B/ANXA13/HLA-E/HSPA1L/HSP90AA1/HSP90AB1/ADAMTSL5/COL28A1/RSPO2/CD300E/IGF1/IGF2/CYR61/IL1R1/IL1RN/IL6/AQP2/IL15RA/IL16/AQP5/INHBA/INPP5A/ITGB2/ITGB7/ITIH3/ITIH4/IVL/JUP/CD82/ACAT1/KRT7/KRT15/LAMA3/STMN1/LCK/LCP1/MUC21/LDLR/ARHGDIA/LGALS9/RAB19/LOX/LTB/LTBP1/MFI2/MFNG/MGAT1/SCGB2A1/MOV10/IGFL4/NUDT1/NUBP1/NDUFB4/NEU1/ATP1A2/NOV/NPPC/NRAS/ATP5B/SPOCK3/UTP11L/ANGPT4/PDE4C/PCYOX1/C11orf73/GALNT7/PGAM2/PI3/PKHD1/PKM/PLA2G2A/PLEC/PRKAG3/CYTL1/POMC/PON1/ROBO4/MXRA8/PPP1CB/MOB1A/TRPV6/CISD1/APOBR/MAPK3/PROC/MASP1/HTRA1/PSMB4/RGMA/PSMD7/SLURP1/ACTR3B/ERMN/ACTA2/S100A4/S100A6/BGLAP/SCT/CCL11/CCL17/TINAGL1/SFRP2/BMP4/SLC4A1/ZNF649/ZG16/SLC9A3/SLC20A2/SLIT1/LYNX1/BPI/STK10/BST2/VAMP2/TCEB2/ACTC1/TGM2/TIMP3/TNFAIP3/TNFRSF1A/TNXB/WNT10B/YWHAG/ZNF177/PTP4A1/MOGS/CXCR4/RAB7A/REEP5/GDPD3/PSCA/COL18A1/CALR/COL21A1/CAPS/COLQ/CAPZB/HIST1H2BM/SH3BGRL3/HIST1H3A/DYNLRB2/ANTXR1/PARD6B/IL1F10/LOXL3/PPFIBP2/SCIN/KMO/RUNX1/SERPINA6/ACTN1/ENDOU/SYT7/ESAM/IL32/TRIP10/ADIPOQ/LY86/RAB3D/H2AFY	238
GO:0042588	zymogen granule	5/942	12/17046	0.00029	0.01812	0.01603	TMED10/DMBT1/ZG16/VAMP2/RAB3D	5

GO:0044459	plasma membrane part	168/942	2392/17046	0.00047	0.02649	0.02344	ABI1/TSPAN5/CDH13/FARP1/KCNMB2/TCIRG1/TRDN/PDPN/GJB6/NPFFR2/ADCY3/TMED10/ADAM29/CHRNA1/CHRNA2/CHRNA5/CLCA1/CCR1/TRPM6/MPP7/APCDD1/CYLD/ADRB3/DLG2/DRD4/DTNA/ECE1/EGFR/TMEM17/SLC10A4/EPHA1/EPHA3/EPHB4/SPATA13/FGA/RASA3/EPB41L3/FLOT2/MLC1/SYNE1/PSD3/GABBR1/TENM4/STEAP2/PLEK2/SLC17A5/GJA3/NPTN/GJB2/GNAS/GPR26/GPER1/FFAR2/FLVCR1/GRIK4/GPR132/GZMA/ANXA2/HAS1/KCNIP2/NRG1/HLA-B/HLA-DOA/HLA-DPA1/ANXA13/HLA-E/HLA-F/HPCA/HRH1/HSP90AA1/HSP90AB1/HTR3A/HTR5A/GPR142/IL1R1/IL6/AQP2/IL11RA/IL12RB2/AQP5/AQP9/ITGA7/ITGB2/ITGB7/JUP/CD82/KCNH2/KCNJ8/KCNJ9/KCNMB1/KDR/SLC6A17/LCK/LCP1/LDLR/LHCGR/MC2R/MFI2/ASGR1/MPZ/ATP1A2/NMBR/OPRL1/OR3A2/SLC22A18/P2RY6/PALM/PDE6B/PKHD1/SPA17/FXYD6/GPR84/SLCO1C1/TLR9/APBB1IP/TRPV6/CHRNA9/PRKD1/MAPK3/MAP2K2/TRPV5/TRPC7/PTGFR/TENM2/TRIM27/RGR/EXOC4/S100A6/NOD2/CXCR5/DNAI2/SLC4A1/SLC6A12/SLC8A1/SLC9A3/SLC20A2/BMPR1B/BPI/BST2/VAMP2/TGM2/TNFRSF1A/TRAF5/TRPC4/TRPC6/TRPM2/CCR2/TNFRSF4/ZAP70/CACNA1E/PTP4A1/CACNB2/CALR/ANTXR1/ATP13A4/CASQ1/SLC43A1/FADD/TNFRSF11A/STBD1/TSPAN18/CCRL2/MAP7/SLC16A3/CD8A/TRIP10/RAPGEF2/CD79A	168
GO:0044424	intracellular part	730/942	12427/17046	0.00052	0.02649	0.02344	AKT3/ABI1/CDH3/TANK/GNE/ZNF783/CDH13/SUGP2/MBNL2/FARP1/RCAN2/CDKN1C/SPEG/BCKDK/TCIRG1/MRVI1/TRDN/ABCA9/C1D/COG5/ZBTB18/PITRM1/TACC2/MTHFS/DMRT2/CELF1/CELF2/TBR1/SEPT9/GJB6/NPFFR2/ADCY3/PNRC1/TMED10/SLC27A2/RER1/HNRNPUL1/RPP14/HIBADH/CHGA/CHI3L1/ERLIN2/PSIP1/PKP3/EGLN2/PXMP4/ATXN2L/B4GALT7/KIF12/ACOT7/EXOC3/CIDEA/GBP4/ALPK2/RBP7/GALNT15/AP3S1/CLCA1/C10orf90/CLN5/MRPL52/FRMD6/CCR1/SPATA33/SLC38A10/SEZ6/KRT40/TNFAIP8L1/CNP/APOA1BP/NEU4/COL9A3/COL11A1/GALM/SCLT1/MAP3K8/ZFP42/ADM/UBLCP1/HUS1B/CPS1/NDUFAF6/PXDNL/CRABP1/ZNF358/MIB2/PARP4/MPP7/FAM101A/B3GLCT/CEP128/MGAT5B/CSTA/KLC3/ZNF738/CTGF/SMYD1/SGOL1/PPM1L/SH3D19/CYLD/MBOAT1/ADRB3/ESCO2/CYP11A1/ZNF782/FITM1/ADAL/ZNF709/ZNF781/CALML6/CITED4/DDB1/WBP2NL/DDOST/RNF168/ZNF366/BHLHA15/PPP1R18/NLRP6/DIO3/DMBT1/DNAH6/DNAH8/DNMT3A/ABAT/DPH1/DRD4/DSG3/DTNA/ECE1/AGXT/EEF2/EGFR/EGR3/PATL2/EIF4G1/A2M/ELK4/ANKRD23/EML1/UNC13D/DNAH12/SMIM14/ENO2/ADCK5/EPHA3/EPHB4/ESR1/ALAS1/FAH/FAT2/SPATA13/PRSS54/RNF182/PHACTR1/SP8/FGA/FGF10/FHIT/XRN2/RASA3/PPM1E/VASH1/BTBD3/TRAK1/MSRB2/ACIN1/FOXL1/FOXC2/FOXO1/EXPH5/AKR1B1/SPG20/EPB41L3/GGA3/FLNB/DIP2A/FLOT2/MLC1/TBC1D1/RHOBTB2/NUP210/SEL1L3/ATP11A/NEDD4L/SYNE1/LARP1/PPP1R13B/PUM2/ARHGEF18/RYBP/MORC3/MAPK8IP2/TSSK2/VGLL2/MTOR/FUCA1/WDR27/SLC37A4/GABBR1/RNF144B/ZNF549/CCDC110/ST6GALNAC3/NUTM1/GAK/SAMM50/DFNB31/ALS2CL/PNKD/SEC31B/TENM4/ACOT11/FAM169A/RAI14/RGS22/STEAP2/GAS2/FBXL21/FBXO2/LCE2B/SACS/GATM/GBGT1/GAPDHS/PLEK2/SLC17A5/RPS6KC1/PABPC1/AKAP8L/DNAJC2/FGF22/NPTN/GJB2/AMPD2/SDCBP2/PDE7B/GLS2/VPS4A/AMPD3/DHDH/BMP10/ZNF638/GNAS/ZNF311/CRACR2B/PIGW/IZUMO1/ZNF844/THEM5/GPER1/EOGT/GRB10/MRPS18B/FLVCR1/ZBTB44/DNAJC15/SCG3/GSTP1/GTF2B/BRF1/TMOD4/GUCY1A3/CCDC106/NME7/PADI1/GZMA/ANXA2/HAS1/SOX8/KCNIP2/NRG1/ANXA6/HK1/HLA-B/HLA-DOA/HLA-DPA1/ANXA13/HLA-E/HLA-F/HLX/HMGA1/NR4A1/ACACB/HPCA/APBA2/HOXB3/HOXC4/HOXC5/HOXC6/HOXD3/HRH1/HSD11B1/HSD17B2/ACADL/HSPA1L/HSP90AA1/HSP90AB1/HTR3A/HTR5A/DUPD1/TFAP2E/ID3/ZC3H12D/COL28A1/FMN1/BARHL2/NME9/IGF1/IGF2/GPR142/LCE1C/LCE1D/LCE2D/IL1R1/IL1RN/IL6/AQP2/IL15RA/PRSS41/IL16/FOXK2/AQP5/IRF1/AQP9/ISL1/ITGA7/ITIH4/IVL/JUP/USP50/HILS1/ATP9B/KCNH2/KCNJ8/KDR/ACAT1/KIF25/IPO5/KRT7/KRT15/INSC/TOMM20L/HES5/SLC6A17/RESP18/AFF3/STMN1/LCK/LCP1/MUC21/LDLR/ARHGDIA/LGALS9/LHCGR/LLGL1/LMNA/LMO2/RAB19/LOX/LPP/SMAD3/MC2R/MCC/ME1/ME2/MEF2D/MAP3K1/MEOX1/MEOX2/MFNG/MGAT1/MITF/LHX8/MOCS1/MOV10/MPZ/MT1A/NUDT1/MYH4/MYL2/NUBP1/NDUFB4/DRG1/NEDD9/NEU1/ATP1A2/NFATC3/NFYB/NHLH2/NMBR/NOV/NPPC/NRAS/NTF3/OAS2/WRAP73/OPRL1/OR2C1/SLC22A18/PAFAH2/ATP5B/DEF6/ANO7/PALM/ARHGEF3/PARK2/BOLA1/UTP11L/LEF1/DDX47/CHST15/PDE4C/PCYOX1/PDE7A/C11orf73/SIRT6/PDE6B/ATP8A2/GALNT7/PGAM2/PIGC/PIK3CG/PITX2/PKHD1/PKM/PLA2G2A/PLAGL1/SPA17/PLEC/PRKAG3/PML/RIPPLY3/RIPK4/TLR9/POMC/SSH1/PON1/RIN2/POU2AF1/ZDHHC13/APBB1IP/FBLIM1/BNC2/MED18/PALMD/CYP2W1/RPP25/LPCAT2/BANP/PPP1CB/HERC6/PPP1CC/PIWIL2/ELP3/ARHGEF10L/PRMT6/DNAJC17/GOLPH3L/ZNF532/PPP2R2B/FANCI/MOB1A/SLC29A3/MIS18BP1/WDR33/SMPD3/CNOT11/CHRNA9/SYBU/PEX26/LIMS2/FRMD4A/VAC14/CARKD/PARVA/PRKAR1B/TTC17/IFT122/ERMARD/LMBRD1/CSGALNACT1/CISD1/PRKD1/MYNN/BIN3/MAPK3/MAP2K2/PRKRIR/PROC/MRAP/PRMT8/HTRA1/CDC42SE1/PSMB4/PAK6/ARNTL2/RGMA/PRDM11/TRPC7/PSMD7/ACTR3B/PTGFR/PLEKHG5/TENM2/GATAD2B/ERMN/KLHL8/RDH14/METTL14/MARK4/CCAR2/PTPRE/PXN/CREBZF/FAM60A/ACTA2/RASGRF2/RFC2/TRIM27/RGS12/RIT2/EXOC4/RPA3/RPL8/RPL29/S100A4/S100A5/S100A6/BGLAP/ABHD4/MRPS14/NPAS3/C19orf33/PARVG/NOD2/MAP1LC3B2/ARHGAP9/TRA2B/GZF1/DNAI2/SGK1/SYNDIG1L/MICAL1/CERK/VPS33A/BMP4/SLC4A1/SPATS2/ZNF649/ZG16/SLC8A1/ZSCAN18/BOK/SOX9/BPI/SRP68/STAT2/STK3/STK10/SUPT6H/BST2/VAMP2/TAF4B/TBP/TCEA1/TCEB2/ZEB1/ACTC1/TEAD3/TERF1/TGM2/TCHH/TIMP3/TLE3/TRAPPC10/TNFAIP3/TNFRSF1A/TRAF1/TRAF5/TRPC4/TRPC6/PHLDA2/TWIST1/CCR2/UCP1/UPP1/VARS/YWHAG/ZAP70/ZNF7/CA7/ZNF124/ZNF177/PTP4A1/MOGS/PAX8/CXCR4/FZD5/RAB7A/ERI3/CCDC86/CARD14/ESRG/BCL2L14/LST1/CERS4/NLRX1/ZNF665/ZMYM1/CSPP1/ZC3H14/ERMP1/CALD1/FAM188A/ZNF606/ZC3H12A/RAB11FIP1/FAAP100/CPEB4/RNF39/C6orf25/COL18A1/ZNF436/CALR/COL21A1/UNC93B1/KRTAP2-1/QTRT1/SLIRP/CAPS/CAST/CAPZB/HIST1H2BM/SH3BGRL3/GSG1/SCRT1/HIST1H3A/CCDC3/DYNLRB2/SLC25A18/SPATA16/KRTAP17-	730
GO:0042589	zymogen granule membrane	4/942	8/17046	0.00054	0.02649	0.02344	TMED10/DMBT1/ZG16/VAMP2	4

GO:0005622	intracellular	746/942	12742/17046	0.00058	0.02651	0.02346	AKT3/ABI1/CDH3/TANK/GNE/ZNF783/CDH13/SUGP2/MBNL2/FARP1/RCAN2/CDKN1C/SPEG/BCKDK/TCIRG1/MRVI1/TRDN/ABCA9/C1D/COG5/ZBTB18/PITRM1/TACC2/MTHFS/DMRT2/CELF1/CELF2/TBR1/SEPT9/GJB6/NPFFR2/ADCY3/PNRC1/TMED10/SLC27A2/RER1/HNRNPUL1/RPP14/HIBADH/CHGA/CHI3L1/ERLIN2/PSIP1/PKP3/EGLN2/PXMP4/ATXN2L/B4GALT7/KIF12/ACOT7/EXOC3/CIDEA/GBP4/ALPK2/RBP7/GALNT15/AP3S1/CLCA1/C10orf90/CLN5/MRPL52/FRMD6/CCR1/SPATA33/SLC38A10/SEZ6/KRT40/TNFAIP8L1/MOB3A/CNP/APOA1BP/NEU4/COL9A3/COL11A1/GALM/SCLT1/MAP3K8/ZFP42/ADM/IL31RA/UBLCP1/HUS1B/CPS1/NDUFAF6/PXDNL/CRABP1/ZNF358/MIB2/PARP4/MPP7/FAM101A/B3GLCT/CEP128/MGAT5B/CSTA/KLC3/ZNF738/CTGF/SMYD1/SGOL1/PPM1L/SH3D19/CYLD/MBOAT1/ADRB3/ESCO2/CYP11A1/ZNF782/FITM1/ADAL/ZNF709/ZNF781/CALML6/CITED4/DDB1/WBP2NL/DDOST/RNF168/ZNF366/BHLHA15/PPP1R18/NLRP6/DIO3/DMBT1/DNAH6/DNAH8/DNMT3A/ABAT/DPH1/DRD4/DSG3/DTNA/ECE1/AGXT/EEF2/EGFR/EGR3/PATL2/EIF4G1/A2M/ELK4/ANKRD23/EML1/UNC13D/DNAH12/SMIM14/ENO2/ADCK5/EPHA3/EPHB4/ESR1/ALAS1/FAH/FAT2/SPATA13/PRSS54/RNF182/PHACTR1/SP8/FGA/FGF10/FHIT/XRN2/RASA3/PPM1E/VASH1/BTBD3/TRAK1/MSRB2/ACIN1/FOXL1/FOXC2/FOXO1/EXPH5/AKR1B1/SPG20/NFASC/EPB41L3/GGA3/FLNB/DIP2A/FLOT2/MLC1/TBC1D1/RHOBTB2/NUP210/SEL1L3/ATP11A/NEDD4L/SYNE1/LARP1/PPP1R13B/PUM2/ARHGEF18/RYBP/MORC3/MAPK8IP2/TSSK2/VGLL2/MTOR/FUCA1/WDR27/SLC37A4/GABBR1/RASGEF1C/RNF144B/ZNF549/CCDC110/ST6GALNAC3/NUTM1/GAK/SAMM50/DFNB31/ALS2CL/PNKD/SEC31B/TENM4/ACOT11/FAM169A/RAI14/RGS22/STEAP2/GAS2/FBXL21/FBXO2/LCE2B/SACS/GATM/GBGT1/GAPDHS/PLEK2/SLC17A5/RPS6KC1/PABPC1/AKAP8L/DNAJC2/FGF22/NPTN/GJB2/AMPD2/SDCBP2/PDE7B/GLS2/VPS4A/AMPD3/DHDH/BMP10/ZNF638/GNAS/ZNF311/CRACR2B/PIGW/IZUMO1/ZNF844/THEM5/GPER1/EOGT/TRIM42/GRB10/MRPS18B/FLVCR1/ZBTB44/DNAJC15/SCG3/GSTP1/GTF2B/BRF1/TMOD4/GUCY1A3/CCDC106/NME7/PADI1/GZMA/ANXA2/HAS1/SOX8/KCNIP2/NRG1/ANXA6/HK1/HLA-B/HLA-DOA/HLA-DPA1/ANXA13/HLA-E/HLA-F/HLX/HMGA1/NR4A1/ACACB/HPCA/APBA2/HOXB3/HOXC4/HOXC5/HOXC6/HOXD3/HRH1/HSD11B1/HSD17B2/ACADL/HSPA1L/HSP90AA1/HSP90AB1/HTR3A/HTR5A/DUPD1/TFAP2E/ID3/ZC3H12D/COL28A1/FMN1/BARHL2/NME9/IGF1/IGF2/GPR142/LCE1C/LCE1D/LCE2D/IL1R1/IL1RN/IL6/AQP2/IL15RA/PRSS41/IL16/FOXK2/AQP5/IRF1/AQP9/ISL1/ITGA7/ITIH4/IVL/JUP/USP50/HILS1/ATP9B/KCNH2/KCNJ8/KDR/ACAT1/KIF25/IPO5/KRT7/KRT15/INSC/TOMM20L/HES5/SLC6A17/RESP18/AFF3/STMN1/LCK/LCP1/MUC21/LDLR/ARHGDIA/LGALS9/LHCGR/LLGL1/LMNA/LMO2/RAB19/LOX/LPP/SMAD3/MC2R/MCC/ME1/ME2/MEF2D/MAP3K1/MEOX1/MEOX2/MFNG/MGAT1/MITF/LHX8/MOCS1/MOV10/MPZ/MT1A/NUDT1/MYH4/MYL2/NUBP1/NDUFB4/DRG1/NEDD9/NEU1/ATP1A2/NFATC3/NFYB/NHLH2/NMBR/NOV/NPPC/NRAS/NTF3/OAS2/WRAP73/OPRL1/OR2C1/SLC22A18/PAFAH2/ATP5B/DEF6/ANO7/PALM/ARHGEF3/PARK2/BOLA1/UTP11L/LEF1/DDX47/CHST15/PDE4C/PCYOX1/PDE7A/C11orf73/SIRT6/PDE6B/ATP8A2/GALNT7/PGAM2/PIGC/PIK3CG/PITX2/PKHD1/PKM/PLA2G2A/PLAGL1/SPA17/PLEC/PRKAG3/PML/RIPPLY3/RIPK4/TLR9/TREM1/POMC/SSH1/PON1/RIN2/MOV10L1/POU2AF1/ZDHHC13/APBB1IP/FBLIM1/BNC2/MED18/PALMD/CYP2W1/RPP25/LPCAT2/BANP/PPP1CB/HERC6/PPP1CC/PIWIL2/ELP3/ARHGEF10L/PRMT6/DNAJC17/GOLPH3L/ZNF532/PPP2R2B/FANCI/MOB1A/SLC29A3/MIS18BP1/WDR33/SMPD3/CNOT11/CHRNA9/SYBU/PEX26/LIMS2/FRMD4A/VAC14/CARKD/PARVA/PRKAR1B/TTC17/IFT122/ERMARD/MCTP2/LMBRD1/CSGALNACT1/PAG1/CISD1/PRKD1/WSB2/MYNN/BIN3/MAPK3/MAP2K2/PRKRIR/PROC/MRAP/PRMT8/HTRA1/SLAMF8/CDC42SE1/PSMB4/PAK6/ARNTL2/RGMA/PRDM11/TRPC7/PSMD7/ACTR3B/PTGFR/PLEKHG5/TENM2/GATAD2B/ERMN/KLHL8/RDH14/METTL14/MARK4/CCAR2/PTPRE/PXN/CREBZF/FAM60A/ACTA2/RASGRF2/RFC2/TRIM27/RGS12/RIT2/EXOC4/RPA3/RPL8/RPL29/S100A4/S100A5/S100A6/BGLAP/CCL11/ABHD4/MRPS14/NPAS3/C19orf33/PARVG/NOD2/MAP1LC3B2/ARHGAP9/TRA2B/GZF1/DNAI2/SGK1/SYNDIG1L/MICAL1/CERK/VPS33A/BMP4/SLC4A1/SPATS2/ZNF649/ZG16/SLC8A1/ZSCAN18/BOK/SOX9/BPI/SRP68/STAT2/STK3/STK10/SUPT6H/BST2/VAMP2/TAF4B/TBP/TCEA1/TCEB2/ZEB1/ACTC1/TEAD3/TERF1/TGM2/TCHH/TIMP3/TLE3/TRAPPC10/TNFAIP3/TNFRSF1A/TNXB/TRAF1/TRAF5/TRPC4/TRPC6/PHLDA2/TWIST1/CCR2/UCP1/UPP1/VARS/YWHAG/ZAP70/ZNF7/CA7/ZNF124/ZNF177/PTP4A1/MOGS/PAX8/CXCR4/FZD5/RAB7A/ERI3/CCDC86/CARD14/ESRG/BCL2L14/LST1/CERS4/NLRX1/ZNF665/ZMYM1/CSPP1/ZC3H14/ERMP1/CALD1/FAM188A/ZNF606/ZC3H12A/RAB11FIP1/FAAP100/CPEB4/RNF39/C6orf25/COL18A1/ZNF436/CALR/COL21A1/UNC93B1/KRTAP2-	746
GO:0009986	cell surface	58/942	680/17046	0.00066	0.02841	0.02514	CDH13/PDPN/HCST/RER1/CHRNA1/CCR1/CPM/ECE1/EGFR/FGA/FGF10/DIP2A/FGF22/NPTN/BMP10/ANXA2/HLA-B/HLA-DPA1/HLA-E/HLA-F/HSP90AB1/RSPO2/IL1R1/IL6/IL12RB2/ITGA7/ITGB2/ITGB7/KCNH2/KDR/CLEC17A/LDLR/MFI2/NEU1/ATP5B/PKHD1/SPA17/SSH1/MXRA8/CYP2W1/PPP1CC/DEFB134/NOD2/CXCR5/DNAI2/SLC9A3/BST2/TNFRSF1A/TRPC4/TNFRSF4/CXCR4/FZD5/CALR/ANTXR1/TNFRSF11A/CD8A/ADIPOQ/CD79A	58
GO:0005911	cell-cell junction	35/942	358/17046	0.00077	0.03108	0.0275	CDH3/GJB6/PKP3/PANX3/FRMD6/MPP7/DSG3/ANKRD23/FAT2/AKR1B1/NFASC/EPB41L3/FLOT2/GJA3/GJB2/JUP/LCK/ATP1A2/NOV/FRMD4A/PRKD1/MAP2K2/PLEKHG5/TENM2/CLDN25/SLC8A1/TRPC4/TRPC6/ZAP70/FZD5/PARD6G/PARD6B/ACTN1/ESAM/RAPGEF2	35
GO:0005769	early endosome	28/942	266/17046	0.00082	0.03117	0.02758	MIB2/ECE1/EGFR/EPHA3/TRAK1/MLC1/ATP11A/STEAP2/RPS6KC1/VPS4A/GPER1/ANXA2/HLA-B/HLA-E/HLA-F/KDR/LDLR/LLGL1/PML/VAC14/MAPK3/MAP2K2/TRIM27/VPS33A/PTP4A1/CXCR4/FZD5/RABGAP1L	28
GO:0005576	extracellular region	270/942	4149/17046	0.00099	0.03562	0.03152	ABI1/CCDC180/CDH13/SPON2/ZBTB18/TMED10/SLC27A2/LECT1/ESM1/CHGA/CHI3L1/ERLIN2/CHI3L2/PTH2/KIF12/ACOT7/C1QTNF7/CLCA1/CLN5/ZG16B/SEZ6/CNP/APOA1BP/COL9A3/COL11A1/GALM/COMP/SCLT1/ADM/EGFLAM/C7orf34/CPD/CPM/PXDNL/PARP4/CSTA/CTGF/PPM1L/DDB1/COCH/DMBT1/ABAT/DSG3/ECE1/EEF2/EFNA2/EGFR/A2M/LIPH/ENO2/EPHA3/EPHB4/F11/FAH/FAT2/PRSS54/FCGR2A/FGA/FGF10/FHIT/VASH1/AKR1B1/NFASC/FLNB/FLOT2/NEDD4L/ARHGEF18/FUCA1/GABBR1/SAMM50/ACOT11/FBXO2/GATM/ADGRF1/PABPC1/FGF22/CLUL1/SDCBP2/DKK3/VPS4A/BMP10/GNAS/SCG3/GSTP1/PADI1/GZMA/ANXA2/SERPIND1/NRG1/ANXA6/HLA-B/ANXA13/HLA-E/HSPA1L/HSP90AA1/HSP90AB1/ADAMTSL5/COL28A1/RSPO2/CD300E/IGF1/IGF2/CYR61/IL1R1/IL1RN/IL6/AQP2/IL15RA/IL16/AQP5/INHBA/INPP5A/ITGB2/ITGB7/ITIH3/ITIH4/IVL/JUP/CD82/KCP/KDR/ACAT1/KRT7/KRT15/RESP18/LAIR2/LAMA3/STMN1/LCK/LCP1/MUC21/LDLR/ARHGDIA/LGALS9/RAB19/LOX/LTB/LTBP1/MFI2/MFNG/MGAT1/SCGB2A1/ASGR1/MOV10/IGFL4/NUDT1/NUBP1/NDUFB4/NEU1/ATP1A2/NOV/NPPC/NRAS/NTF3/ATP5B/SPOCK3/BOLA1/UTP11L/ANGPT4/PDE4C/PCYOX1/C11orf73/GALNT7/PGAM2/PI3/PKHD1/PKM/PLA2G2A/PLEC/PRKAG3/PNLIP/TLR9/TREM1/CYTL1/POMC/PON1/ROBO4/MXRA8/PPP1CB/MOB1A/TRPV6/CISD1/APOBR/MAPK3/MAP2K2/PROC/MASP1/HTRA1/PSMB4/RGMA/PSMD7/SLURP1/ACTR3B/PTGFR/ERMN/ACTA2/DEFB134/S100A4/S100A6/BGLAP/SCT/CCL11/CCL17/PRSS22/TINAGL1/SFRP2/BMP4/SLC4A1/ZNF649/ZG16/SLC9A3/SLC20A2/SLIT1/LYNX1/BPI/STK10/BST2/VAMP2/TCEB2/ACTC1/TGM2/TIMP3/TNFAIP3/TNFRSF1A/TNXB/WNT10B/YWHAG/ZNF177/PTP4A1/MOGS/CXCR4/RAB7A/REEP5/GDPD3/EPHX3/PSCA/COL18A1/CALR/COL21A1/CAPS/COLQ/CAPZB/HIST1H2BM/SH3BGRL3/HIST1H3A/CCDC3/DYNLRB2/ANTXR1/PARD6B/IL1F10/SPINK7/RETNLB/LOXL3/PPFIBP2/SCIN/KMO/RUNX1/SERPINA6/ACTN1/ENDOU/SYT7/ESAM/IL32/CD8A/TRIP10/ADIPOQ/LY86/RAB3D/H2AFY/FGF19	270

**All DMC, Molecular Function**								
	Description	GeneRatio	BgRatio	pvalue	p.adjust	qvalue	geneID	Count
GO:0003674	molecular_function	893/893	15274/17046	1.62E-44	1.63E-41	1.44E-41	AKT3/ABI1/CDH3/TANK/CD300LD/GNE/ZNF783/CDH9/TSPAN5/CDH12/CDH13/SUGP2/MBNL2/FARP1/KLRG1/RCAN2/KCNMB2/CDKN1C/SPEG/BCKDK/TCIRG1/MRVI1/TRDN/ABCA9/SPON2/C1D/COG5/ZBTB18/PITRM1/TACC2/MTHFS/DMRT2/CELF1/CELF2/TBR1/RFPL2/SEPT9/HCST/NPFFR2/ADCY3/PNRC1/TMED10/SLC27A2/RER1/ESM1/ADAM29/HNRNPUL1/RPP14/HIBADH/CHI3L1/ERLIN2/PSIP1/CHI3L2/PKP3/EGLN2/TP53TG1/PXMP4/ATXN2L/B4GALT7/KIF12/ACOT7/PDAP1/EXOC3/CHRNA1/CHRNA2/ADPRHL1/CHRNA5/CARD16/GPRIN1/SORCS1/ZBED9/PWWP2A/CIDEA/GBP4/ALPK2/PANX3/RBP7/GALNT15/AP3S1/CLCA1/FAT3/CLN5/MRPL52/FRMD6/CCR1/SLC51B/C15orf27/ZG16B/SLC38A10/KRT40/TNFAIP8L1/MOB3A/CNP/AADACL3/LRRIQ3/APOA1BP/NEU4/COL9A3/COL11A1/GALM/COMP/SCLT1/MAP3K8/ZFP42/ADM/IL31RA/EGFLAM/UBLCP1/OR2A14/CPD/CPM/CPS1/NDUFAF6/PXDNL/CRABP1/ZNF358/TRPM6/CRYBB3/MIB2/PARP4/MPP7/LDLRAD3/FAM101A/B3GLCT/MGAT5B/APCDD1/CSTA/KLC3/ZNF738/CTGF/ABCC13/SMYD1/SGOL1/PPM1L/LRRC34/SH3D19/CYB561/CYLD/MBOAT1/ADRB3/ESCO2/CYP11A1/ZNF782/FITM1/ADAL/TRPV3/ZNF709/ZNF781/CALML6/CITED4/DDB1/WBP2NL/LONRF2/DDOST/RNF168/RBM46/ZNF366/BHLHA15/COCH/PPP1R18/NLRP6/DIO3/DLG2/DMBT1/DNAH6/DNAH8/DNMT3A/ABAT/DPH1/DRD4/DSG3/DTNA/ECE1/AGXT/EEF2/EFNA2/EGFR/EGR3/PATL2/EIF4G1/A2M/ELK4/ANKRD23/LIPH/EML1/UNC13D/ZBTB7C/DNAH12/SLC10A4/ENO2/ADCK5/EPHA1/EPHA3/EPHB4/ESR1/ALAS1/F11/FAH/FAT2/SPATA13/PRSS54/FCGR2A/RNF182/PHACTR1/SP8/FGA/FGF10/FHIT/XRN2/RASA3/RNF44/PPM1E/VASH1/TRAK1/MSRB2/ACIN1/LIMCH1/FOXL1/FOXC2/TBC1D9B/FOXO1/EXPH5/AKR1B1/SPG20/NFASC/EPB41L3/GGA3/FLNB/DIP2A/FLOT2/MLC1/TBC1D1/RHOBTB2/NUP210/ATP11A/KLHL18/NEDD4L/SYNE1/PSD3/LARP1/PPP1R13B/PUM2/ARHGEF18/RYBP/MORC3/MAPK8IP2/TSSK2/VGLL2/MTOR/FUCA1/WDR27/TTLL10/SLC37A4/GABBR1/RASGEF1C/RNF144B/ZNF549/CCDC110/ST6GALNAC3/NUTM1/DSCR9/GAK/SAMM50/DFNB31/ALS2CL/PNKD/TENM4/ACOT11/LTN1/RGS22/STEAP2/FBXL21/FBXO2/LCE2B/SACS/GATM/GBGT1/GAPDHS/SLC17A5/ADGRF1/RPS6KC1/PABPC1/AKAP8L/GJA3/DNAJC2/FGF22/NPTN/GJB2/AMPD2/SDCBP2/PDE7B/CYTH4/GLS2/VPS4A/AMPD3/GPR162/DHDH/BMP10/ZNF638/GNAS/C11orf31/ZNF311/CRACR2B/TMPRSS12/PIGW/IZUMO1/ZNF844/THEM5/GPR26/GPER1/EOGT/DOK7/DCBLD1/FFAR2/TRIM42/GRB10/MRPS18B/FLVCR1/GRIK4/ZBTB44/DNAJC15/SCG3/GSTP1/GTF2B/BRF1/TMOD4/GUCY1A3/CCDC106/NME7/GPR132/PADI1/GZMA/ANXA2/HAS1/SERPIND1/SOX8/KCNIP2/NRG1/ANXA6/HK1/HLA-B/HLA-DOA/HLA-DPA1/ANXA13/HLA-E/HLA-F/HLX/HMGA1/NR4A1/ACACB/HPCA/APBA2/HOXB3/HOXC4/HOXC5/HOXC6/HOXD3/AGFG2/HRH1/HSD11B1/HSD17B2/ACADL/HSPA1L/HSP90AA1/HSP90AB1/HTR3A/HTR5A/DUPD1/ADAMTSL5/ANKRD45/TFAP2E/ID3/ZC3H12D/COL28A1/RSPO2/FMN1/BARHL2/NME9/IGF1/IGF2/CYR61/GPR142/LCE1C/LCE1D/LCE2D/IL1R1/IL1RN/IL6/IL10RA/AQP2/IL11RA/IL12RB2/IL15RA/PRSS41/IL16/FOXK2/AQP5/INHBA/INPP5A/IRF1/AQP9/ISL1/ITGA7/ITGB2/ITGB7/ITIH3/ITIH4/IVL/JUP/CD82/USP50/HILS1/ATP9B/KCNH2/KCNJ8/KCNJ9/KCNMB1/KDR/ACAT1/KIF25/IPO5/KRT7/KRT15/C17orf82/CLEC17A/HES5/SLC6A17/CDHR4/RBM12B/AFF3/LAIR2/LAMA3/STMN1/OR2A5/LCK/LCP1/LDLR/ARHGDIA/LGALS9/LHCGR/LLGL1/C11orf87/LMNA/LMO2/RAB19/LOX/ZNF833P/LPP/LTB/LTBP1/SMAD3/MC2R/MCC/ME1/ME2/MEF2D/MAP3K1/MEOX1/MEOX2/MFI2/MFNG/MGAT1/SCGB2A1/MITF/LHX8/ASGR1/MOCS1/MOV10/MPZ/PLEKHG7/MT1A/NUDT1/MYH4/MYL2/NUBP1/NDUFB4/DRG1/NEDD9/NEU1/ATP1A2/NFATC3/NFYB/NHLH2/NMBR/NOV/NPPC/NRAS/NTF3/OAS2/RNF165/OPRL1/OR2C1/OR3A2/SLC22A18/P2RY6/PAFAH2/ATP5B/IL21R/DEF6/ANO7/PALM/ARHGEF3/PARK2/SPOCK3/BOLA1/UTP11L/LEF1/DDX47/PRR16/CHST15/ANGPT4/PDE4C/PCYOX1/PDE7A/C11orf73/SIRT6/PDE6B/ATP8A2/GALNT7/PGAM2/PI3/PIGC/PIK3CG/PITX2/PKHD1/PKM/PLA2G2A/PLAGL1/SPA17/LRP1B/PLEC/PRKAG3/PML/FXYD6/GPR84/IL20RB/SLCO1C1/PNLIP/RIPK4/TLR9/TREM1/CYTL1/POMC/SSH1/PON1/RIN2/MOV10L1/POU2AF1/ZDHHC13/APBB1IP/ROBO4/MXRA8/FBLIM1/BORCS6/BNC2/MED18/CYP2W1/RPP25/LPCAT2/TTC12/BANP/PPP1CB/FAM118A/HERC6/PPP1CC/PIWIL2/ELP3/ARHGEF10L/PRMT6/DNAJC17/GOLPH3L/ZNF532/PPP2R2B/FANCI/MOB1A/SLC47A1/SLC29A3/MIS18BP1/WDR33/TRPV6/SMPD3/SLC30A10/CNOT11/CHRNA9/THUMPD1/SYBU/PEX26/LIMS2/FRMD4A/VAC14/CARKD/PARVA/PRKAR1B/TTC17/IFT122/MCTP2/LMBRD1/CSGALNACT1/PAG1/CISD1/PRKD1/MYNN/BIN3/APOBR/MAPK3/MAP2K2/PCDHGC4/PCDHGB7/PCDHGB3/PCDHGA11/PRKRIR/PROC/MRAP/TRPV5/PRMT8/MASP1/HTRA1/SLAMF8/CDC42SE1/PSMB4/PAK6/ARNTL2/RGMA/PRDM11/TRPC7/LPAR5/PSMD7/SLURP1/ACTR3B/PTGFR/PLEKHG5/TENM2/GATAD2B/ERMN/RNF150/RDH14/METTL14/MARK4/CCAR2/PTPRE/PXN/CREBZF/AB	893

GO:0005488	binding	774/893	12915/17046	7.46E-17	3.75E-14	3.33E-14	AKT3/ABI1/CDH3/TANK/CD300LD/GNE/ZNF783/CDH9/TSPAN5/CDH12/CDH13/SUGP2/MBNL2/FARP1/KLRG1/RCAN2/CDKN1C/SPEG/BCKDK/TCIRG1/MRVI1/TRDN/ABCA9/SPON2/C1D/COG5/ZBTB18/PITRM1/TACC2/MTHFS/DMRT2/CELF1/CELF2/TBR1/RFPL2/SEPT9/HCST/NPFFR2/ADCY3/PNRC1/TMED10/SLC27A2/RER1/ESM1/ADAM29/HNRNPUL1/RPP14/HIBADH/CHI3L1/ERLIN2/PSIP1/CHI3L2/PKP3/EGLN2/PXMP4/ATXN2L/B4GALT7/KIF12/ACOT7/PDAP1/EXOC3/CHRNA1/CHRNA2/ADPRHL1/CHRNA5/GPRIN1/SORCS1/ZBED9/PWWP2A/CIDEA/GBP4/ALPK2/RBP7/GALNT15/AP3S1/CLCA1/FAT3/CLN5/FRMD6/CCR1/SLC51B/ZG16B/KRT40/TNFAIP8L1/MOB3A/CNP/LRRIQ3/APOA1BP/NEU4/COL11A1/GALM/COMP/SCLT1/MAP3K8/ZFP42/ADM/IL31RA/EGFLAM/UBLCP1/CPD/CPM/CPS1/PXDNL/CRABP1/ZNF358/TRPM6/CRYBB3/MIB2/PARP4/MPP7/LDLRAD3/FAM101A/MGAT5B/APCDD1/CSTA/KLC3/ZNF738/CTGF/ABCC13/SMYD1/SGOL1/PPM1L/SH3D19/CYB561/CYLD/ADRB3/ESCO2/CYP11A1/ZNF782/ADAL/ZNF709/ZNF781/CALML6/DDB1/WBP2NL/LONRF2/DDOST/RNF168/RBM46/ZNF366/BHLHA15/COCH/PPP1R18/NLRP6/DLG2/DMBT1/DNAH6/DNAH8/DNMT3A/ABAT/DPH1/DRD4/DSG3/DTNA/ECE1/AGXT/EEF2/EFNA2/EGFR/EGR3/PATL2/EIF4G1/A2M/ELK4/ANKRD23/LIPH/EML1/UNC13D/ZBTB7C/DNAH12/ENO2/ADCK5/EPHA1/EPHA3/EPHB4/ESR1/ALAS1/F11/FAH/FAT2/SPATA13/FCGR2A/RNF182/PHACTR1/SP8/FGA/FGF10/FHIT/XRN2/RASA3/RNF44/PPM1E/VASH1/TRAK1/MSRB2/ACIN1/LIMCH1/FOXL1/FOXC2/TBC1D9B/FOXO1/EXPH5/SPG20/NFASC/EPB41L3/GGA3/FLNB/DIP2A/FLOT2/MLC1/TBC1D1/RHOBTB2/NUP210/ATP11A/KLHL18/NEDD4L/SYNE1/LARP1/PPP1R13B/PUM2/RYBP/MORC3/MAPK8IP2/TSSK2/VGLL2/MTOR/WDR27/TTLL10/GABBR1/RNF144B/ZNF549/CCDC110/NUTM1/GAK/SAMM50/DFNB31/ALS2CL/PNKD/TENM4/ACOT11/LTN1/STEAP2/FBXO2/SACS/GBGT1/GAPDHS/RPS6KC1/PABPC1/AKAP8L/GJA3/DNAJC2/FGF22/NPTN/AMPD2/SDCBP2/PDE7B/CYTH4/GLS2/VPS4A/AMPD3/BMP10/ZNF638/GNAS/C11orf31/ZNF311/CRACR2B/IZUMO1/ZNF844/THEM5/GPER1/DOK7/DCBLD1/FFAR2/TRIM42/GRB10/FLVCR1/ZBTB44/DNAJC15/SCG3/GSTP1/GTF2B/BRF1/TMOD4/GUCY1A3/CCDC106/NME7/PADI1/GZMA/ANXA2/SERPIND1/SOX8/KCNIP2/NRG1/ANXA6/HK1/HLA-B/HLA-DOA/HLA-DPA1/ANXA13/HLA-E/HLA-F/HLX/HMGA1/NR4A1/ACACB/HPCA/APBA2/HOXB3/HOXC4/HOXC5/HOXC6/HOXD3/AGFG2/HRH1/ACADL/HSPA1L/HSP90AA1/HSP90AB1/HTR3A/ADAMTSL5/ANKRD45/TFAP2E/ID3/ZC3H12D/RSPO2/FMN1/BARHL2/IGF1/IGF2/CYR61/LCE2D/IL1R1/IL1RN/IL6/IL10RA/IL12RB2/IL15RA/IL16/FOXK2/AQP5/INHBA/INPP5A/IRF1/ISL1/ITGA7/ITGB2/ITGB7/ITIH4/IVL/JUP/CD82/HILS1/ATP9B/KCNH2/KCNJ8/KCNJ9/KDR/ACAT1/KIF25/IPO5/KRT7/KRT15/C17orf82/CLEC17A/HES5/CDHR4/RBM12B/AFF3/LAIR2/LAMA3/STMN1/LCK/LCP1/LDLR/ARHGDIA/LGALS9/LHCGR/LLGL1/C11orf87/LMNA/LMO2/RAB19/LOX/ZNF833P/LPP/LTB/LTBP1/SMAD3/MC2R/MCC/ME1/ME2/MEF2D/MAP3K1/MEOX1/MEOX2/MFI2/MFNG/MGAT1/SCGB2A1/MITF/LHX8/ASGR1/MOCS1/MOV10/MT1A/NUDT1/MYH4/MYL2/NUBP1/DRG1/NEDD9/ATP1A2/NFATC3/NFYB/NHLH2/NOV/NPPC/NRAS/NTF3/OAS2/RNF165/OPRL1/SLC22A18/P2RY6/PAFAH2/ATP5B/DEF6/PALM/ARHGEF3/PARK2/SPOCK3/BOLA1/UTP11L/LEF1/DDX47/PRR16/CHST15/ANGPT4/PDE4C/PDE7A/C11orf73/SIRT6/PDE6B/ATP8A2/GALNT7/PGAM2/PIK3CG/PITX2/PKHD1/PKM/PLA2G2A/PLAGL1/SPA17/LRP1B/PLEC/PRKAG3/PML/FXYD6/IL20RB/PNLIP/RIPK4/TLR9/TREM1/CYTL1/POMC/SSH1/PON1/MOV10L1/POU2AF1/ZDHHC13/APBB1IP/FBLIM1/BORCS6/BNC2/MED18/CYP2W1/RPP25/LPCAT2/TTC12/BANP/PPP1CB/FAM118A/PPP1CC/PIWIL2/ELP3/PRMT6/DNAJC17/GOLPH3L/ZNF532/PPP2R2B/FANCI/MOB1A/MIS18BP1/WDR33/TRPV6/SMPD3/CNOT11/CHRNA9/THUMPD1/SYBU/PEX26/LIMS2/FRMD4A/VAC14/CARKD/PARVA/PRKAR1B/TTC17/IFT122/MCTP2/LMBRD1/CSGALNACT1/PAG1/CISD1/PRKD1/MYNN/BIN3/MAPK3/MAP2K2/PCDHGC4/PCDHGB7/PCDHGB3/PCDHGA11/PRKRIR/PROC/MRAP/TRPV5/PRMT8/MASP1/HTRA1/PSMB4/PAK6/ARNTL2/RGMA/TRPC7/PSMD7/SLURP1/ACTR3B/TENM2/GATAD2B/ERMN/RNF150/METTL14/MARK4/CCAR2/PTPRE/PXN/CREBZF/FAM60A/ACTA2/RASGRF2/RFC2/TRIM27/RGR/RIT2/EXOC4/RPA3/RPL8/RPL29/S100A4/S100A5/S100A6/BGLAP/SCT/CCL11/CCL17/MRPS14/NPAS3/PARVG/NOD2/TINAGL1/SFRP2/CXCR5/ARHGAP9/TRA2B/GZF1/DNAI2/SGK1/MICAL1/CERK/PCDH20/VPS33A/BMP4/SLC4A1/SPATS2/ZNF649/ZG16/SLC6A12/SLC8A1/SLC9A3/BMPR1B/SLIT1/BRD9/ZSCAN18/BOK/SOX9/BPI/SRP68/STAT2/STK3/STK10/SUPT6H/BST2/VAMP2/TAF4B/TBP/TCEA1/TCEB2/ZEB1/ACTC1/TEAD3/TERF1/TGM2/TCHH/TIMP3/TLE3/TLR5/TRAPPC10/TNFAIP3/TNFRSF1A/TNXB/TRAF1/TRAF5/TRPC4/TRPC6/TWIST1/CCR2/VARS/ZNHIT2/WNT10B/YWHAG/ZAP70/ZNF7/CA7/ZNF124/ZNF177/CACNA1E/CACNB2/PAX8/CXCR4/FZD5/RAB7A/ERI3/REEP5/CCDC86/CARD14/GDPD3/BCL2L14/	774

GO:0005515	protein binding	594/893	9755/17046	3.42E-09	1.15E-06	1.02E-06	AKT3/ABI1/TANK/CD300LD/GNE/ZNF783/TSPAN5/CDH13/FARP1/KLRG1/CDKN1C/SPEG/BCKDK/TCIRG1/MRVI1/TRDN/SPON2/C1D/COG5/ZBTB18/TACC2/DMRT2/CELF1/SEPT9/HCST/NPFFR2/ADCY3/PNRC1/TMED10/SLC27A2/RER1/ESM1/HNRNPUL1/ERLIN2/PSIP1/PKP3/EGLN2/PXMP4/ATXN2L/B4GALT7/KIF12/ACOT7/EXOC3/CHRNA5/GPRIN1/SORCS1/PWWP2A/CIDEA/RBP7/AP3S1/CLN5/FRMD6/CCR1/SLC51B/KRT40/TNFAIP8L1/LRRIQ3/APOA1BP/NEU4/COL11A1/COMP/SCLT1/MAP3K8/ADM/IL31RA/UBLCP1/CPS1/CRABP1/TRPM6/CRYBB3/MIB2/PARP4/MPP7/FAM101A/MGAT5B/APCDD1/CSTA/KLC3/CTGF/SMYD1/SGOL1/SH3D19/CYB561/CYLD/ADRB3/DDB1/WBP2NL/DDOST/RNF168/ZNF366/BHLHA15/COCH/PPP1R18/DLG2/DMBT1/DNMT3A/ABAT/DPH1/DRD4/DTNA/ECE1/AGXT/EEF2/EFNA2/EGFR/PATL2/EIF4G1/A2M/ELK4/ANKRD23/EML1/UNC13D/ADCK5/EPHA1/EPHA3/EPHB4/ESR1/ALAS1/F11/FAH/SPATA13/FCGR2A/RNF182/PHACTR1/FGA/FGF10/FHIT/PPM1E/VASH1/TRAK1/MSRB2/ACIN1/LIMCH1/FOXC2/TBC1D9B/FOXO1/EXPH5/SPG20/NFASC/EPB41L3/GGA3/FLNB/DIP2A/FLOT2/MLC1/TBC1D1/NUP210/ATP11A/KLHL18/NEDD4L/SYNE1/LARP1/PPP1R13B/PUM2/RYBP/MAPK8IP2/TSSK2/VGLL2/MTOR/WDR27/TTLL10/GABBR1/RNF144B/CCDC110/NUTM1/GAK/SAMM50/DFNB31/ALS2CL/PNKD/TENM4/LTN1/FBXO2/SACS/GAPDHS/RPS6KC1/PABPC1/AKAP8L/GJA3/DNAJC2/FGF22/NPTN/AMPD2/SDCBP2/GLS2/VPS4A/BMP10/GNAS/CRACR2B/IZUMO1/ZNF844/THEM5/GPER1/DOK7/FFAR2/TRIM42/GRB10/FLVCR1/ZBTB44/DNAJC15/GSTP1/GTF2B/BRF1/TMOD4/GUCY1A3/CCDC106/NME7/GZMA/ANXA2/SOX8/KCNIP2/NRG1/ANXA6/HK1/HLA-B/HLA-DOA/HLA-E/HLA-F/HLX/HMGA1/NR4A1/ACACB/HPCA/APBA2/HOXC4/HOXD3/HSPA1L/HSP90AA1/HSP90AB1/ADAMTSL5/ANKRD45/TFAP2E/ID3/RSPO2/FMN1/BARHL2/IGF1/IGF2/CYR61/LCE2D/IL1R1/IL1RN/IL6/IL10RA/IL12RB2/IL15RA/IL16/FOXK2/AQP5/INHBA/INPP5A/IRF1/ISL1/ITGA7/ITGB2/ITGB7/ITIH4/IVL/JUP/CD82/HILS1/KCNH2/KCNJ8/KCNJ9/KDR/ACAT1/KIF25/IPO5/KRT7/KRT15/C17orf82/HES5/RBM12B/LAIR2/LAMA3/STMN1/LCK/LCP1/LDLR/ARHGDIA/LGALS9/LLGL1/C11orf87/LMNA/LMO2/LOX/LPP/LTB/LTBP1/SMAD3/MC2R/MCC/MEF2D/MAP3K1/MEOX1/MEOX2/MFI2/SCGB2A1/MITF/LHX8/ASGR1/MOV10/MT1A/NUDT1/MYH4/MYL2/NUBP1/DRG1/NEDD9/ATP1A2/NFATC3/NFYB/NHLH2/NOV/NPPC/NRAS/NTF3/OAS2/OPRL1/SLC22A18/P2RY6/ATP5B/DEF6/PALM/ARHGEF3/PARK2/BOLA1/UTP11L/LEF1/DDX47/PRR16/ANGPT4/PDE4C/C11orf73/SIRT6/PIK3CG/PITX2/PKHD1/PKM/SPA17/PLEC/PRKAG3/PML/FXYD6/IL20RB/RIPK4/TLR9/TREM1/CYTL1/POMC/SSH1/PON1/POU2AF1/APBB1IP/FBLIM1/BORCS6/MED18/RPP25/TTC12/BANP/PPP1CB/FAM118A/PPP1CC/ELP3/PRMT6/GOLPH3L/PPP2R2B/FANCI/MOB1A/MIS18BP1/TRPV6/CNOT11/SYBU/PEX26/LIMS2/FRMD4A/VAC14/CARKD/PARVA/PRKAR1B/TTC17/IFT122/LMBRD1/PAG1/PRKD1/BIN3/MAPK3/MAP2K2/PRKRIR/PROC/MRAP/TRPV5/PRMT8/MASP1/HTRA1/PSMB4/PAK6/ARNTL2/RGMA/TRPC7/PSMD7/SLURP1/ACTR3B/TENM2/GATAD2B/ERMN/MARK4/CCAR2/PTPRE/PXN/CREBZF/FAM60A/ACTA2/RASGRF2/RFC2/TRIM27/RGR/RIT2/EXOC4/RPA3/S100A4/S100A5/S100A6/SCT/CCL11/CCL17/NPAS3/PARVG/NOD2/TINAGL1/SFRP2/CXCR5/ARHGAP9/TRA2B/DNAI2/SGK1/MICAL1/CERK/VPS33A/BMP4/SLC4A1/ZNF649/ZG16/SLC6A12/SLC8A1/SLC9A3/BMPR1B/SLIT1/BRD9/BOK/SOX9/STAT2/STK3/STK10/SUPT6H/BST2/VAMP2/TAF4B/TBP/TCEA1/TCEB2/ZEB1/ACTC1/TEAD3/TERF1/TGM2/TCHH/TIMP3/TLE3/TLR5/TRAPPC10/TNFAIP3/TNFRSF1A/TNXB/TRAF1/TRAF5/TRPC4/TRPC6/TWIST1/CCR2/VARS/WNT10B/YWHAG/ZAP70/ZNF124/ZNF177/CACNB2/PAX8/CXCR4/FZD5/RAB7A/REEP5/CCDC86/CARD14/BCL2L14/NLRX1/IGFLR1/ZMYM1/CSPP1/ZC3H14/CALD1/FAM188A/ZC3H12A/RAB11FIP1/FAAP100/CPEB4/COL18A1/ZNF436/CALR/SLIRP/COLQ/CAST/CAPZB/HIST1H2BM/SH3BGRL3/GSG1/HIST1H3A/SLC25A18/ANTXR1/SLA2/BFSP2/YIPF4/ZNF397/NR0B2/HOPX/PARD6G/PARD6B/IL1F10/SPINK7/RETNLB/TRIM63/KDM2B/LOXL3/GTPBP3/MGARP/CBX2/RAE1/IFITM1/GAS7/SCIN/CDK10/RUNX1/TP63/RUNX3/IRS2/ACTN1/CRADD/FADD/TNFRSF11A/SYNJ2/SPHK1/BUD31/CCNA1/ENDOU/SKAP2/STBD1/LIMD1/THAP3/CCRL2/MAP7/PRC1/STARD13/PIAS2/SYT7/LDB2/SLC16A3/CBFA2T2/RSAD2/AURKB/DAPL1/RCSD1/IL32/CD8A/REEP6/TRIP10/ADIPOQ/ARHGAP29/RAB3D/H2AFY/ENTPD3/ARHGEF10/MICAL2/N4BP1/VGLL4/RAPGEF2/ULK2/USP6NL/CD79A/DAZAP2/TELO2/RABGAP1L/TMCC2/FGF19/NR1H4	594
GO:0043169	cation binding	264/893	3856/17046	4.55E-07	0.00011	0.0001	CDH3/TANK/GNE/CDH9/CDH12/CDH13/MBNL2/SPON2/ZBTB18/PITRM1/MTHFS/DMRT2/RFPL2/ADCY3/ADAM29/EGLN2/B4GALT7/CHRNA1/CHRNA2/ADPRHL1/CHRNA5/GALNT15/CLCA1/FAT3/MOB3A/APOA1BP/COL11A1/COMP/MAP3K8/ZFP42/CPD/CPM/CPS1/PXDNL/ZNF358/TRPM6/MIB2/MGAT5B/SMYD1/PPM1L/CYB561/CYLD/ESCO2/CYP11A1/ZNF782/ADAL/ZNF709/ZNF781/CALML6/LONRF2/RNF168/ZNF366/DNMT3A/ABAT/DRD4/DSG3/DTNA/ECE1/EGR3/EML1/ZBTB7C/ENO2/ESR1/FAH/FAT2/RNF182/SP8/XRN2/RASA3/RNF44/PPM1E/MSRB2/LIMCH1/TBC1D9B/ATP11A/RYBP/MORC3/TSSK2/RNF144B/ZNF549/PNKD/LTN1/STEAP2/GBGT1/AKAP8L/AMPD2/PDE7B/AMPD3/ZNF638/GNAS/ZNF311/CRACR2B/ZNF844/TRIM42/ZBTB44/GTF2B/BRF1/NME7/PADI1/ANXA2/KCNIP2/ANXA6/ANXA13/NR4A1/ACACB/HPCA/AGFG2/HRH1/HTR3A/ADAMTSL5/ZC3H12D/FOXK2/ISL1/ITGA7/ITGB2/ITGB7/ATP9B/ACAT1/CLEC17A/CDHR4/LCP1/LDLR/LMO2/LOX/ZNF833P/LPP/LTBP1/SMAD3/ME1/ME2/MAP3K1/MFI2/MFNG/MGAT1/LHX8/ASGR1/MOCS1/MT1A/NUDT1/MYL2/NUBP1/ATP1A2/OAS2/RNF165/PARK2/SPOCK3/PDE4C/PDE7A/SIRT6/PDE6B/ATP8A2/GALNT7/PKM/PLA2G2A/PLAGL1/LRP1B/PML/PNLIP/PON1/MOV10L1/ZDHHC13/FBLIM1/BNC2/CYP2W1/LPCAT2/PPP1CB/PPP1CC/ELP3/ZNF532/MOB1A/SMPD3/CHRNA9/LIMS2/MCTP2/CSGALNACT1/CISD1/PRKD1/MYNN/PCDHGC4/PCDHGB7/PCDHGB3/PCDHGA11/PRKRIR/PROC/MASP1/GATAD2B/RNF150/PXN/TRIM27/S100A4/S100A5/S100A6/BGLAP/GZF1/MICAL1/CERK/PCDH20/ZNF649/SLC8A1/BMPR1B/SLIT1/ZSCAN18/STK3/TCEA1/ZEB1/TGM2/TCHH/TIMP3/TNFAIP3/TRAF1/TRAF5/ZNHIT2/ZNF7/CA7/ZNF124/ZNF177/CACNA1E/ERI3/GDPD3/ZNF665/ZMYM1/ZC3H14/ERMP1/ZNF606/ZC3H12A/RNF39/COL18A1/ZNF436/CALR/QTRT1/CAPS/SCRT1/ANTXR1/ATP13A4/ZNF397/CASQ1/TRIM63/KDM2B/LOXL3/SCIN/RUNX1/TP63/ACTN1/TNFRSF11A/SPHK1/ENDOU/LIMD1/CH25H/THAP3/ERI1/PIAS2/ZFAND2A/MAP3K6/SYT7/CBFA2T2/RSAD2/AURKB/NEURL3/ARHGAP29/MTL5/MICAL2/RAPGEF2/ZBTB39/NR1H4	264

GO:0043167	ion binding	362/893	5637/17046	9.40E-07	0.00019	0.00017	AKT3/CDH3/TANK/GNE/CDH9/CDH12/CDH13/MBNL2/SPEG/BCKDK/ABCA9/SPON2/ZBTB18/PITRM1/MTHFS/DMRT2/RFPL2/SEPT9/ADCY3/SLC27A2/ADAM29/EGLN2/B4GALT7/KIF12/ACOT7/CHRNA1/CHRNA2/ADPRHL1/CHRNA5/GBP4/ALPK2/GALNT15/CLCA1/FAT3/MOB3A/APOA1BP/COL11A1/COMP/MAP3K8/ZFP42/CPD/CPM/CPS1/PXDNL/CRABP1/ZNF358/TRPM6/MIB2/MGAT5B/CTGF/ABCC13/SMYD1/PPM1L/CYB561/CYLD/ESCO2/CYP11A1/ZNF782/ADAL/ZNF709/ZNF781/CALML6/LONRF2/RNF168/ZNF366/NLRP6/DNAH6/DNAH8/DNMT3A/ABAT/DRD4/DSG3/DTNA/ECE1/AGXT/EEF2/EGFR/EGR3/LIPH/EML1/ZBTB7C/DNAH12/ENO2/EPHA1/EPHA3/EPHB4/ESR1/ALAS1/F11/FAH/FAT2/RNF182/SP8/FGF10/XRN2/RASA3/RNF44/PPM1E/MSRB2/LIMCH1/TBC1D9B/RHOBTB2/ATP11A/RYBP/MORC3/TSSK2/MTOR/TTLL10/RNF144B/ZNF549/GAK/PNKD/LTN1/STEAP2/GBGT1/RPS6KC1/AKAP8L/AMPD2/PDE7B/VPS4A/AMPD3/ZNF638/GNAS/ZNF311/CRACR2B/ZNF844/TRIM42/ZBTB44/GTF2B/BRF1/GUCY1A3/NME7/PADI1/ANXA2/SERPIND1/KCNIP2/ANXA6/HK1/ANXA13/NR4A1/ACACB/HPCA/AGFG2/HRH1/ACADL/HSPA1L/HSP90AA1/HSP90AB1/HTR3A/ADAMTSL5/ZC3H12D/RSPO2/CYR61/FOXK2/ISL1/ITGA7/ITGB2/ITGB7/ATP9B/KCNJ8/KDR/ACAT1/KIF25/CLEC17A/CDHR4/LCK/LCP1/LDLR/LMO2/RAB19/LOX/ZNF833P/LPP/LTBP1/SMAD3/ME1/ME2/MAP3K1/MFI2/MFNG/MGAT1/LHX8/ASGR1/MOCS1/MOV10/MT1A/NUDT1/MYH4/MYL2/NUBP1/DRG1/ATP1A2/NOV/NRAS/OAS2/RNF165/PAFAH2/ATP5B/PARK2/SPOCK3/DDX47/CHST15/PDE4C/PDE7A/SIRT6/PDE6B/ATP8A2/GALNT7/PIK3CG/PKM/PLA2G2A/PLAGL1/LRP1B/PRKAG3/PML/PNLIP/RIPK4/PON1/MOV10L1/ZDHHC13/FBLIM1/BNC2/CYP2W1/LPCAT2/PPP1CB/PPP1CC/ELP3/GOLPH3L/ZNF532/MOB1A/SMPD3/CHRNA9/LIMS2/CARKD/PRKAR1B/MCTP2/CSGALNACT1/CISD1/PRKD1/MYNN/MAPK3/MAP2K2/PCDHGC4/PCDHGB7/PCDHGB3/PCDHGA11/PRKRIR/PROC/MASP1/PAK6/TRPC7/ACTR3B/GATAD2B/RNF150/MARK4/PXN/ACTA2/RFC2/TRIM27/RIT2/RPL29/S100A4/S100A5/S100A6/BGLAP/NOD2/ARHGAP9/GZF1/SGK1/MICAL1/CERK/PCDH20/BMP4/ZNF649/SLC8A1/BMPR1B/SLIT1/ZSCAN18/STK3/STK10/VAMP2/TCEA1/ZEB1/ACTC1/TGM2/TCHH/TIMP3/TNFAIP3/TNXB/TRAF1/TRAF5/TRPC4/TRPC6/VARS/ZNHIT2/ZAP70/ZNF7/CA7/ZNF124/ZNF177/CACNA1E/RAB7A/ERI3/GDPD3/NLRX1/ZNF665/ZMYM1/ZC3H14/ERMP1/ZNF606/ZC3H12A/RNF39/C6orf25/COL18A1/ZNF436/CALR/QTRT1/CAPS/SCRT1/ANTXR1/ATP13A4/ZNF397/CASQ1/TTBK1/TRIM63/KDM2B/LOXL3/GTPBP3/SCIN/CDK10/KMO/RUNX1/TP63/RUNX3/ACTN1/TNFRSF11A/STK19/SPHK1/ENDOU/LIMD1/CH25H/THAP3/ERI1/PIAS2/ZFAND2A/MAP3K6/SYT7/CBFA2T2/RSAD2/AURKB/NEURL3/ADIPOQ/ARHGAP29/RAB3D/ENTPD3/RAB36/MTL5/MICAL2/RAPGEF2/ULK2/ZBTB39/NR1H4	362
GO:0046872	metal ion binding	257/893	3777/17046	1.19E-06	0.0002	0.00018	CDH3/TANK/GNE/CDH9/CDH12/CDH13/MBNL2/SPON2/ZBTB18/PITRM1/MTHFS/DMRT2/RFPL2/ADCY3/ADAM29/EGLN2/B4GALT7/ADPRHL1/GALNT15/CLCA1/FAT3/MOB3A/APOA1BP/COL11A1/COMP/MAP3K8/ZFP42/CPD/CPM/CPS1/PXDNL/ZNF358/TRPM6/MIB2/MGAT5B/SMYD1/PPM1L/CYB561/CYLD/ESCO2/CYP11A1/ZNF782/ADAL/ZNF709/ZNF781/CALML6/LONRF2/RNF168/ZNF366/DNMT3A/ABAT/DSG3/DTNA/ECE1/EGR3/EML1/ZBTB7C/ENO2/ESR1/FAH/FAT2/RNF182/SP8/XRN2/RASA3/RNF44/PPM1E/MSRB2/LIMCH1/TBC1D9B/ATP11A/RYBP/MORC3/TSSK2/RNF144B/ZNF549/PNKD/LTN1/STEAP2/GBGT1/AKAP8L/AMPD2/PDE7B/AMPD3/ZNF638/GNAS/ZNF311/CRACR2B/ZNF844/TRIM42/ZBTB44/GTF2B/BRF1/NME7/PADI1/ANXA2/KCNIP2/ANXA6/ANXA13/NR4A1/ACACB/HPCA/AGFG2/ADAMTSL5/ZC3H12D/FOXK2/ISL1/ITGA7/ITGB2/ITGB7/ATP9B/ACAT1/CLEC17A/CDHR4/LCP1/LDLR/LMO2/LOX/ZNF833P/LPP/LTBP1/SMAD3/ME1/ME2/MAP3K1/MFI2/MFNG/MGAT1/LHX8/ASGR1/MOCS1/MT1A/NUDT1/MYL2/NUBP1/ATP1A2/OAS2/RNF165/PARK2/SPOCK3/PDE4C/PDE7A/SIRT6/PDE6B/ATP8A2/GALNT7/PKM/PLA2G2A/PLAGL1/LRP1B/PML/PNLIP/PON1/MOV10L1/ZDHHC13/FBLIM1/BNC2/CYP2W1/LPCAT2/PPP1CB/PPP1CC/ELP3/ZNF532/MOB1A/SMPD3/LIMS2/MCTP2/CSGALNACT1/CISD1/PRKD1/MYNN/PCDHGC4/PCDHGB7/PCDHGB3/PCDHGA11/PRKRIR/PROC/MASP1/GATAD2B/RNF150/PXN/TRIM27/S100A4/S100A5/S100A6/BGLAP/GZF1/MICAL1/CERK/PCDH20/ZNF649/SLC8A1/BMPR1B/SLIT1/ZSCAN18/STK3/TCEA1/ZEB1/TGM2/TCHH/TIMP3/TNFAIP3/TRAF1/TRAF5/ZNHIT2/ZNF7/CA7/ZNF124/ZNF177/CACNA1E/ERI3/GDPD3/ZNF665/ZMYM1/ZC3H14/ERMP1/ZNF606/ZC3H12A/RNF39/COL18A1/ZNF436/CALR/QTRT1/CAPS/SCRT1/ANTXR1/ATP13A4/ZNF397/CASQ1/TRIM63/KDM2B/LOXL3/SCIN/RUNX1/TP63/ACTN1/TNFRSF11A/SPHK1/ENDOU/LIMD1/CH25H/THAP3/ERI1/PIAS2/ZFAND2A/MAP3K6/SYT7/CBFA2T2/RSAD2/AURKB/NEURL3/ARHGAP29/MTL5/MICAL2/RAPGEF2/ZBTB39/NR1H4	257
GO:0005102	receptor binding	100/893	1310/17046	7.80E-05	0.01121	0.00994	TRDN/C1D/TACC2/HCST/NPFFR2/SLC27A2/RER1/ESM1/ADM/CTGF/ADRB3/ZNF366/DLG2/AGXT/EFNA2/A2M/FGA/FGF10/TRAK1/FLOT2/FGF22/NPTN/BMP10/GNAS/IZUMO1/DOK7/GRB10/GTF2B/NRG1/HLA-B/HLA-E/HLA-F/HMGA1/HSP90AB1/RSPO2/IGF1/IGF2/CYR61/IL1R1/IL1RN/IL6/IL16/INHBA/ISL1/KCNJ8/KDR/LAMA3/LCK/LTB/SMAD3/NOV/NPPC/NTF3/ATP5B/PALM/PARK2/LEF1/ANGPT4/PIK3CG/TLR9/CYTL1/POMC/LMBRD1/MRAP/RGMA/SLURP1/TENM2/PXN/S100A4/SCT/CCL11/CCL17/SFRP2/BMP4/SLIT1/TLR5/TNXB/TRAF1/CCR2/WNT10B/YWHAG/ZAP70/CALR/SH3BGRL3/NR0B2/IL1F10/RETNLB/IRS2/ACTN1/FADD/ENDOU/CCRL2/MAP7/PIAS2/IL32/CD8A/ADIPOQ/RAPGEF2/FGF19/NR1H4	100
GO:0060089	molecular transducer activity	119/893	1631/17046	0.00011	0.01382	0.01225	KLRG1/NPFFR2/CHRNA1/CHRNA2/CHRNA5/SORCS1/CCR1/MAP3K8/IL31RA/OR2A14/MIB2/ADRB3/NLRP6/DMBT1/DRD4/EGFR/EPHA1/EPHA3/EPHB4/ESR1/GABBR1/ADGRF1/NPTN/GPR162/GNAS/GPR26/GPER1/FFAR2/GRIK4/GUCY1A3/GPR132/HLA-DOA/HLA-DPA1/NR4A1/HRH1/HTR3A/HTR5A/GPR142/IL1R1/IL10RA/IL11RA/IL12RB2/IL15RA/ITGB2/ITGB7/JUP/KCNH2/KDR/STMN1/OR2A5/LDLR/LGALS9/LHCGR/LTBP1/SMAD3/MC2R/MCC/MAP3K1/ASGR1/NMBR/OPRL1/OR2C1/OR3A2/P2RY6/IL21R/LEF1/PKHD1/LRP1B/GPR84/IL20RB/TLR9/TREM1/ZDHHC13/ROBO4/CHRNA9/VAC14/APOBR/MAPK3/SLAMF8/RGMA/LPAR5/PTGFR/PLEKHG5/PTPRE/TRIM27/RGR/RGS12/TINAGL1/CXCR5/SLC20A2/BMPR1B/STAT2/STK3/STK10/BST2/TNFRSF1A/TRAF5/CCR2/TNFRSF4/PAX8/CXCR4/FZD5/GPR157/ANTXR1/NR0B2/TRIM63/LOXL3/IFITM1/IRS2/TNFRSF11A/SPHK1/ENDOU/SLAMF9/CCRL2/MAP3K6/CD8A/RAPGEF2/CD79A/NR1H4	119
GO:0004896	cytokine receptor activity	13/893	80/17046	0.00025	0.02766	0.02452	CCR1/IL31RA/IL1R1/IL10RA/IL11RA/IL12RB2/IL15RA/IL21R/IL20RB/CXCR5/CCR2/CXCR4/CCRL2	13
GO:0008092	cytoskeletal protein binding	62/893	761/17046	0.00036	0.03482	0.03086	ABI1/FARP1/KIF12/MIB2/FAM101A/KLC3/PPP1R18/EGFR/ANKRD23/EML1/PHACTR1/MSRB2/LIMCH1/EPB41L3/FLNB/SYNE1/MAPK8IP2/BMP10/TMOD4/ANXA2/HPCA/FMN1/KIF25/STMN1/LCP1/MYH4/MYL2/PARK2/PLEC/SSH1/FBLIM1/SYBU/PARVA/BIN3/ACTR3B/ERMN/MARK4/PXN/S100A4/S100A6/PARVG/NOD2/MICAL1/SLC4A1/SLC8A1/VAMP2/ACTC1/TERF1/CXCR4/CALD1/CAPZB/ANTXR1/TRIM63/RAE1/GAS7/SCIN/ACTN1/PRC1/RCSD1/RAB3D/ARHGEF10/MICAL2	62
GO:0003779	actin binding	35/893	363/17046	0.00038	0.03482	0.03086	MIB2/PPP1R18/EGFR/PHACTR1/MSRB2/LIMCH1/EPB41L3/FLNB/SYNE1/TMOD4/HPCA/FMN1/LCP1/MYH4/MYL2/PARK2/PLEC/SSH1/PARVA/ACTR3B/ERMN/S100A4/PARVG/NOD2/MICAL1/SLC4A1/CXCR4/CALD1/CAPZB/ANTXR1/GAS7/SCIN/ACTN1/RCSD1/MICAL2	35

Hypomethylated DMC, Biological Process								
	Description	GeneRatio	BgRatio	pvalue	p.adjust	qvalue	geneID	Count
GO:0008150	biological_process	590/590	15230/17046	3.93E-30	2.05E-26	1.78E-26	AKT3/ABI1/TANK/CD300LD/GNE/ZNF783/CDH9/CDH12/SUGP2/FARP1/KLRG1/RCAN2/CDKN1C/SPEG/MRVI1/TRDN/SPON2/COG5/PITRM1/TACC2/MTHFS/PDPN/CELF1/GJB6/PNRC1/TMED10/LECT1/RER1/ESM1/ADAM29/HNRNPUL1/RPP14/CHGA/CHI3L1/ERLIN2/CHI3L2/PKP3/EGLN2/TP53TG1/ATXN2L/KIF12/ACOT7/PDAP1/EXOC3/CHRNA1/CHRNA2/CARD16/GPRIN1/SORCS1/C1QTNF7/GBP4/PANX3/RBP7/GALNT15/AP3S1/FAT3/CLN5/MRPL52/CCR1/SLC51B/SEZ6/TNFAIP8L1/CNP/AADACL3/NEU4/COL9A3/COMP/MAP3K8/ADM/IL31RA/EGFLAM/UBLCP1/HUS1B/OR2A14/CPM/CPS1/NDUFAF6/PXDNL/CRABP1/ZNF358/CRYBB3/MIB2/PARP4/MPP7/LDLRAD3/FAM101A/B3GLCT/MGAT5B/CSTA/KLC3/ZNF738/ABCC13/SMYD1/SGOL1/PPM1L/SH3D19/CYLD/MBOAT1/ESCO2/CYP11A1/ZNF782/DDB1/LONRF2/DDOST/ZNF366/BHLHA15/DIO3/DLG2/DMBT1/DNAH6/ABAT/DPH1/DTNA/AGXT/EEF2/EFNA2/EIF4G1/A2M/ELK4/ANKRD23/LIPH/SLC10A4/ENO2/ADCK5/EPHA1/ESR1/F11/FAH/FAT2/SPATA13/PRSS54/FCGR2A/PHACTR1/FGA/FGF10/FHIT/BTBD3/SBNO2/TRAK1/MSRB2/FOXL1/FOXC2/EXPH5/AKR1B1/NFASC/EPB41L3/GGA3/DIP2A/FLOT2/MLC1/TBC1D1/RHOBTB2/NUP210/NEDD4L/SYNE1/PSD3/PPP1R13B/PUM2/ARHGEF18/RYBP/MORC3/MAPK8IP2/TSSK2/VGLL2/SLC37A4/RASGEF1C/RNF144B/ZNF549/ST6GALNAC3/SAMM50/DFNB31/ALS2CL/PNKD/SEC31B/ACOT11/STEAP2/GAS2/FBXL21/LCE2B/SACS/GATM/GBGT1/PLEK2/SLC17A5/ADGRF1/RPS6KC1/PABPC1/AKAP8L/DNAJC2/FGF22/NPTN/CLUL1/PDE7B/CYTH4/AMPD3/DHDH/BMP10/ZNF638/ZNF311/TMPRSS12/ZNF844/THEM5/GPR26/EOGT/FFAR2/GRB10/MRPS18B/GRIK4/DNAJC15/SCG3/TMOD4/GUCY1A3/GPR132/PADI1/HAS1/SOX8/HK1/HLA-DOA/HLA-DPA1/ANXA13/NR4A1/ACACB/HOXC4/AGFG2/HRH1/HSD11B1/HSPA1L/HSP90AB1/HTR3A/DUPD1/ADAMTSL5/ANKRD45/TFAP2E/ID3/ZC3H12D/RSPO2/CD300E/NME9/IGF1/IGF2/GPR142/LCE1C/LCE1D/LCE2D/IL1R1/IL1RN/IL10RA/AQP2/IL11RA/IL12RB2/IL15RA/PRSS41/IL16/FOXK2/AQP5/INHBA/INPP5A/IRF1/AQP9/ITGA7/ITGB2/ITGB7/ITIH4/IVL/JUP/CD82/USP50/KCNH2/KCNJ8/KCNJ9/KCNMB1/KDR/KIF25/IPO5/AMIGO3/HES5/AFF3/LAMA3/STMN1/OR2A5/LCP1/MUC21/LDLR/ARHGDIA/LGALS9/LHCGR/LMNA/LMO2/RAB19/SRRD/LPP/MC2R/MCC/ME2/MFNG/MGAT1/SCGB2A1/MITF/LHX8/ASGR1/MOCS1/MOV10/MPZ/PLEKHG7/MYH4/MYL2/NUBP1/NDUFB4/DRG1/NEU1/ATP1A2/NFATC3/NHLH2/NPPC/NRAS/OAS2/OPRL1/OR2C1/OR3A2/SLC22A18/P2RY6/PAFAH2/ATP5B/IL21R/ANO7/PARK2/SPOCK3/UTP11L/PRR16/CHST15/PCYOX1/C11orf73/SIRT6/PDE6B/ATP8A2/PI3/PITX2/PKHD1/PKM/PLA2G2A/PLAGL1/LRP1B/PRKAG3/PML/RIPPLY3/FXYD6/GPR84/SLCO1C1/PNLIP/TLR9/TREM1/SSH1/RIN2/POU2AF1/APBB1IP/MXRA8/FBLIM1/MED18/PALMD/CYP2W1/RPP25/BANP/PPP1CB/HERC6/ELP3/DNAJC17/GOLPH3L/PPP2R2B/FANCI/MOB1A/SLC47A1/SLC29A3/MIS18BP1/TRPV6/SLC30A10/CHRNA9/PEX26/FRMD4A/CARKD/PARVA/PRKAR1B/TTC17/IFT122/ERMARD/MCTP2/LMBRD1/CSGALNACT1/PAG1/CISD1/WSB2/MYNN/APOBR/MAPK3/MAP2K2/PCDHGB3/PRKRIR/PROC/MRAP/TRPV5/PRMT8/HTRA1/SLAMF8/CDC42SE1/PAK6/ARNTL2/RGMA/PRDM11/PSMD7/SLURP1/PTGFR/PLEKHG5/TENM2/GATAD2B/ERMN/KLHL8/RDH14/MARK4/PTPRE/CREBZF/ABHD17C/FAM60A/ACTA2/RFC2/TRIM27/RGR/RGS12/RIT2/RPA3/BGLAP/SCT/CCL11/ABHD4/MRPS14/PRSS22/NPAS3/PARVG/NOD2/TINAGL1/STRA6/MAP1LC3B2/ARHGAP9/GZF1/DNAI2/PCDH20/TMEM237/VPS33A/BMP4/SLC4A1/ZNF649/ZG16/SLC6A12/SLC8A1/SLC9A3/SLC20A2/BMPR1B/SLIT1/BRD9/ZSCAN18/TMEM108/BOK/BPI/SRP68/STAT2/STK3/SUPT6H/BST2/TCEB2/ZEB1/TEAD3/TERF1/TGM2/TCHH/TNFAIP3/TNFRSF1A/TRAF5/TRPC6/TRPM2/CCR2/TNFRSF4/UCP1/WNT10B/ZAP70/ZNF7/CA7/CACNA1E/MOGS/RAB7A/ERI3/CCDC86/CARD14/BCL2L14/LST1/CERS4/ZNF665/CSPP1/ERMP1/CALD1/FAM188A/TMEM62/GPR157/ZC3H12A/FAAP100/CPEB4/C6orf25/ZNF436/EEPD1/CLPTM1L/CALR/COL21A1/QTRT1/SLIRP/CAST/CAPZB/DYNLRB2/SLC25A18/SPATA16/ANTXR1/MFSD7/CMAHP/BFSP2/ATP13A4/ZNF397/NR0B2/MON1A/CASQ1/HOPX/PARD6G/KRBA1/TTBK1/RETNLB/TRIM63/GTPBP3/RAE1/SLC43A1/IFITM1/SCIN/CDK10/KMO/RUNX1/TP63/RUNX3/SERPINA6/IRS2/ACTN1/CRADD/TNFRSF11A/ALDH1A2/STK19/SYNJ2/SPHK1/BUD31/CCNA1/ENDOU/SKAP2/STBD1/HSPB3/TSPAN18/CH25H/CCRL2/ERI1/PRC1/STARD13/PIAS2/MAP3K6/SYT7/ESAM/SLC16A3/CBFA2T2/RSAD2/AURKB/DAPL1/CD8A/CCDC102A/NEURL3/TRIP10/ADIPOQ/ENTPD3/PREPL/RAB36/MICAL2/N4BP1/VGLL4/NUP93/RAPGEF2/CD79A/ZBTB39/IQSEC1/LPGAT1/FGF19/NR1H4	590

GO:0009987	cellular process	542/590	13765/17046	1.64E-14	2.90E-11	2.51E-11	AKT3/ABI1/TANK/GNE/ZNF783/CDH9/CDH12/SUGP2/FARP1/KLRG1/RCAN2/CDKN1C/SPEG/MRVI1/TRDN/SPON2/TACC2/MTHFS/PDPN/CELF1/GJB6/PNRC1/TMED10/LECT1/RER1/ESM1/HNRNPUL1/RPP14/CHGA/CHI3L1/ERLIN2/PKP3/EGLN2/TP53TG1/ATXN2L/KIF12/ACOT7/PDAP1/EXOC3/CHRNA1/CHRNA2/CARD16/GPRIN1/SORCS1/C1QTNF7/PANX3/GALNT15/AP3S1/CLN5/MRPL52/CCR1/SLC51B/SEZ6/TNFAIP8L1/CNP/NEU4/COL9A3/COMP/MAP3K8/ADM/IL31RA/EGFLAM/UBLCP1/HUS1B/OR2A14/CPS1/NDUFAF6/PXDNL/CRABP1/ZNF358/MIB2/PARP4/MPP7/FAM101A/B3GLCT/MGAT5B/CSTA/KLC3/ZNF738/ABCC13/SMYD1/SGOL1/PPM1L/SH3D19/CYLD/MBOAT1/ESCO2/CYP11A1/ZNF782/DDB1/DDOST/ZNF366/BHLHA15/DIO3/DLG2/DMBT1/DNAH6/ABAT/DPH1/DTNA/AGXT/EEF2/EFNA2/EIF4G1/A2M/ELK4/SLC10A4/ENO2/ADCK5/EPHA1/ESR1/FAH/FAT2/SPATA13/FCGR2A/PHACTR1/FGA/FGF10/FHIT/BTBD3/SBNO2/TRAK1/MSRB2/FOXL1/FOXC2/EXPH5/AKR1B1/NFASC/EPB41L3/DIP2A/FLOT2/MLC1/TBC1D1/RHOBTB2/NUP210/NEDD4L/SYNE1/PSD3/PPP1R13B/PUM2/ARHGEF18/RYBP/MORC3/MAPK8IP2/TSSK2/VGLL2/SLC37A4/RASGEF1C/RNF144B/ZNF549/ST6GALNAC3/SAMM50/DFNB31/ALS2CL/PNKD/ACOT11/STEAP2/GAS2/FBXL21/LCE2B/SACS/GATM/GBGT1/PLEK2/SLC17A5/ADGRF1/RPS6KC1/PABPC1/DNAJC2/FGF22/NPTN/CLUL1/PDE7B/CYTH4/AMPD3/BMP10/ZNF638/ZNF311/ZNF844/THEM5/GPR26/EOGT/FFAR2/GRB10/MRPS18B/GRIK4/DNAJC15/SCG3/TMOD4/GUCY1A3/GPR132/PADI1/HAS1/SOX8/HK1/HLA-DOA/HLA-DPA1/ANXA13/NR4A1/ACACB/HOXC4/HRH1/HSPA1L/HSP90AB1/HTR3A/DUPD1/TFAP2E/ID3/ZC3H12D/RSPO2/NME9/IGF1/IGF2/GPR142/LCE1C/LCE1D/LCE2D/IL1R1/IL1RN/IL10RA/AQP2/IL11RA/IL12RB2/IL15RA/IL16/FOXK2/AQP5/INHBA/INPP5A/IRF1/AQP9/ITGA7/ITGB2/ITGB7/ITIH4/IVL/JUP/CD82/USP50/KCNH2/KCNJ8/KCNJ9/KCNMB1/KDR/KIF25/IPO5/AMIGO3/HES5/AFF3/LAMA3/STMN1/OR2A5/LCP1/MUC21/LDLR/ARHGDIA/LGALS9/LHCGR/LMNA/LMO2/RAB19/MC2R/MCC/ME2/MFNG/MGAT1/SCGB2A1/MITF/LHX8/ASGR1/MOCS1/MOV10/MPZ/PLEKHG7/MYH4/MYL2/NUBP1/NDUFB4/DRG1/NEU1/ATP1A2/NFATC3/NHLH2/NPPC/NRAS/OAS2/OPRL1/OR2C1/OR3A2/SLC22A18/P2RY6/PAFAH2/ATP5B/IL21R/ANO7/PARK2/SPOCK3/UTP11L/PRR16/CHST15/PCYOX1/C11orf73/SIRT6/PDE6B/ATP8A2/PI3/PITX2/PKHD1/PKM/PLA2G2A/PLAGL1/PRKAG3/PML/RIPPLY3/FXYD6/GPR84/SLCO1C1/PNLIP/TLR9/TREM1/SSH1/RIN2/POU2AF1/APBB1IP/MXRA8/FBLIM1/MED18/PALMD/CYP2W1/RPP25/BANP/PPP1CB/HERC6/ELP3/DNAJC17/GOLPH3L/PPP2R2B/FANCI/MOB1A/SLC47A1/SLC29A3/MIS18BP1/TRPV6/SLC30A10/CHRNA9/PEX26/FRMD4A/PARVA/PRKAR1B/TTC17/IFT122/MCTP2/LMBRD1/CSGALNACT1/PAG1/CISD1/WSB2/MYNN/APOBR/MAPK3/MAP2K2/PRKRIR/PROC/MRAP/TRPV5/PRMT8/HTRA1/SLAMF8/CDC42SE1/PAK6/ARNTL2/RGMA/PRDM11/PSMD7/SLURP1/PTGFR/PLEKHG5/TENM2/GATAD2B/ERMN/KLHL8/RDH14/MARK4/PTPRE/CREBZF/FAM60A/ACTA2/RFC2/TRIM27/RGR/RGS12/RIT2/RPA3/BGLAP/SCT/CCL11/MRPS14/NPAS3/PARVG/NOD2/STRA6/MAP1LC3B2/ARHGAP9/GZF1/DNAI2/TMEM237/VPS33A/BMP4/SLC4A1/ZNF649/SLC6A12/SLC8A1/SLC9A3/SLC20A2/BMPR1B/SLIT1/BRD9/ZSCAN18/BOK/BPI/SRP68/STAT2/STK3/SUPT6H/BST2/TCEB2/ZEB1/TEAD3/TERF1/TGM2/TCHH/TNFAIP3/TNFRSF1A/TRAF5/TRPC6/TRPM2/CCR2/TNFRSF4/UCP1/WNT10B/ZAP70/ZNF7/CA7/CACNA1E/MOGS/RAB7A/ERI3/CCDC86/CARD14/BCL2L14/LST1/CERS4/ZNF665/CSPP1/CALD1/FAM188A/GPR157/ZC3H12A/FAAP100/CPEB4/C6orf25/ZNF436/EEPD1/CLPTM1L/CALR/COL21A1/QTRT1/SLIRP/CAST/CAPZB/DYNLRB2/SLC25A18/SPATA16/ANTXR1/MFSD7/CMAHP/BFSP2/ATP13A4/ZNF397/NR0B2/MON1A/CASQ1/HOPX/PARD6G/TTBK1/TRIM63/GTPBP3/RAE1/SLC43A1/IFITM1/SCIN/CDK10/KMO/RUNX1/TP63/RUNX3/SERPINA6/IRS2/ACTN1/CRADD/TNFRSF11A/ALDH1A2/STK19/SYNJ2/SPHK1/BUD31/CCNA1/ENDOU/SKAP2/STBD1/TSPAN18/CH25H/CCRL2/ERI1/PRC1/STARD13/PIAS2/MAP3K6/SYT7/ESAM/SLC16A3/CBFA2T2/RSAD2/AURKB/DAPL1/CD8A/NEURL3/TRIP10/ADIPOQ/ENTPD3/RAB36/MICAL2/N4BP1/VGLL4/NUP93/RAPGEF2/CD79A/ZBTB39/IQSEC1/LPGAT1/FGF19/NR1H4	542
GO:0044763	single-organism cellular process	474/590	11314/17046	1.67E-14	2.90E-11	2.51E-11	AKT3/ABI1/TANK/GNE/CDH9/CDH12/FARP1/KLRG1/RCAN2/CDKN1C/SPEG/MRVI1/TRDN/SPON2/TACC2/MTHFS/PDPN/CELF1/GJB6/TMED10/LECT1/RER1/ESM1/CHGA/CHI3L1/ERLIN2/PKP3/EGLN2/KIF12/ACOT7/PDAP1/EXOC3/CHRNA1/CHRNA2/CARD16/GPRIN1/SORCS1/PANX3/GALNT15/AP3S1/CLN5/MRPL52/CCR1/SLC51B/SEZ6/TNFAIP8L1/CNP/NEU4/COL9A3/COMP/MAP3K8/ADM/IL31RA/EGFLAM/HUS1B/OR2A14/CPS1/NDUFAF6/PXDNL/CRABP1/MIB2/PARP4/MPP7/FAM101A/B3GLCT/MGAT5B/CSTA/KLC3/ABCC13/SMYD1/SGOL1/PPM1L/SH3D19/CYLD/MBOAT1/ESCO2/CYP11A1/DDB1/DDOST/ZNF366/BHLHA15/DLG2/DMBT1/DNAH6/ABAT/DPH1/DTNA/AGXT/EEF2/EFNA2/EIF4G1/A2M/ELK4/SLC10A4/ENO2/EPHA1/ESR1/FAH/FAT2/SPATA13/FCGR2A/PHACTR1/FGA/FGF10/FHIT/BTBD3/SBNO2/TRAK1/MSRB2/FOXL1/FOXC2/EXPH5/AKR1B1/NFASC/EPB41L3/DIP2A/FLOT2/RHOBTB2/NUP210/NEDD4L/SYNE1/PSD3/PPP1R13B/PUM2/ARHGEF18/RYBP/MORC3/MAPK8IP2/TSSK2/SLC37A4/RASGEF1C/RNF144B/ST6GALNAC3/SAMM50/DFNB31/ALS2CL/PNKD/ACOT11/STEAP2/GAS2/LCE2B/GATM/GBGT1/PLEK2/SLC17A5/ADGRF1/RPS6KC1/PABPC1/FGF22/NPTN/CLUL1/PDE7B/CYTH4/AMPD3/BMP10/THEM5/GPR26/EOGT/FFAR2/GRB10/MRPS18B/GRIK4/DNAJC15/SCG3/TMOD4/GUCY1A3/GPR132/PADI1/HAS1/SOX8/HK1/HLA-DOA/HLA-DPA1/ANXA13/NR4A1/ACACB/HRH1/HSPA1L/HSP90AB1/HTR3A/ID3/ZC3H12D/RSPO2/NME9/IGF1/IGF2/GPR142/LCE1C/LCE1D/LCE2D/IL1R1/IL1RN/IL10RA/AQP2/IL11RA/IL12RB2/IL15RA/IL16/FOXK2/AQP5/INHBA/INPP5A/IRF1/AQP9/ITGA7/ITGB2/ITGB7/IVL/JUP/CD82/USP50/KCNH2/KCNJ8/KCNJ9/KCNMB1/KDR/KIF25/AMIGO3/HES5/LAMA3/STMN1/OR2A5/LCP1/MUC21/LDLR/ARHGDIA/LGALS9/LHCGR/LMNA/RAB19/MC2R/MCC/ME2/MFNG/MGAT1/SCGB2A1/MITF/LHX8/ASGR1/MOV10/MPZ/PLEKHG7/MYH4/MYL2/NUBP1/NDUFB4/NEU1/ATP1A2/NFATC3/NHLH2/NPPC/NRAS/OAS2/OPRL1/OR2C1/OR3A2/SLC22A18/P2RY6/PAFAH2/ATP5B/IL21R/ANO7/PARK2/SPOCK3/UTP11L/PRR16/CHST15/PCYOX1/SIRT6/PDE6B/ATP8A2/PITX2/PKHD1/PKM/PLA2G2A/PLAGL1/PRKAG3/PML/FXYD6/GPR84/SLCO1C1/PNLIP/TLR9/TREM1/SSH1/RIN2/APBB1IP/MXRA8/FBLIM1/PALMD/CYP2W1/BANP/PPP1CB/HERC6/ELP3/GOLPH3L/PPP2R2B/FANCI/MOB1A/SLC47A1/SLC29A3/MIS18BP1/TRPV6/SLC30A10/CHRNA9/PEX26/FRMD4A/PARVA/PRKAR1B/TTC17/IFT122/MCTP2/LMBRD1/CSGALNACT1/PAG1/CISD1/WSB2/APOBR/MAPK3/MAP2K2/PRKRIR/PROC/MRAP/TRPV5/PRMT8/HTRA1/SLAMF8/CDC42SE1/PAK6/RGMA/PSMD7/SLURP1/PTGFR/PLEKHG5/TENM2/ERMN/RDH14/MARK4/PTPRE/FAM60A/ACTA2/RFC2/TRIM27/RGR/RGS12/RIT2/RPA3/BGLAP/SCT/CCL11/MRPS14/PARVG/NOD2/STRA6/ARHGAP9/DNAI2/TMEM237/VPS33A/BMP4/SLC4A1/SLC6A12/SLC8A1/SLC9A3/SLC20A2/BMPR1B/SLIT1/BOK/BPI/SRP68/STAT2/STK3/SUPT6H/BST2/ZEB1/TEAD3/TERF1/TGM2/TCHH/TNFAIP3/TNFRSF1A/TRAF5/TRPC6/TRPM2/CCR2/TNFRSF4/UCP1/WNT10B/ZAP70/CA7/CACNA1E/MOGS/RAB7A/CARD14/BCL2L14/LST1/CERS4/CSPP1/CALD1/FAM188A/GPR157/ZC3H12A/FAAP100/CPEB4/C6orf25/EEPD1/CLPTM1L/CALR/COL21A1/QTRT1/SLIRP/CAST/CAPZB/DYNLRB2/SLC25A18/SPATA16/ANTXR1/MFSD7/CMAHP/BFSP2/ATP13A4/NR0B2/MON1A/CASQ1/HOPX/PARD6G/TTBK1/TRIM63/RAE1/SLC43A1/IFITM1/SCIN/CDK10/KMO/RUNX1/TP63/RUNX3/IRS2/ACTN1/CRADD/TNFRSF11A/ALDH1A2/SYNJ2/SPHK1/CCNA1/SKAP2/STBD1/TSPAN18/CH25H/CCRL2/ERI1/PRC1/STARD13/PIAS2/MAP3K6/SYT7/ESAM/SLC16A3/CBFA2T2/RSAD2/AURKB/DAPL1/CD8A/TRIP10/ADIPOQ/ENTPD3/RAB36/MICAL2/NUP93/RAPGEF2/CD79A/IQSEC1/LPGAT1/FGF19/NR1H4	474

GO:0044699	single-organism process	503/590	12449/17046	3.90E-13	5.09E-10	4.42E-10	AKT3/ABI1/TANK/GNE/CDH9/CDH12/FARP1/KLRG1/RCAN2/CDKN1C/SPEG/MRVI1/TRDN/SPON2/COG5/TACC2/MTHFS/PDPN/CELF1/GJB6/TMED10/LECT1/RER1/ESM1/ADAM29/CHGA/CHI3L1/ERLIN2/PKP3/EGLN2/KIF12/ACOT7/PDAP1/EXOC3/CHRNA1/CHRNA2/CARD16/GPRIN1/SORCS1/PANX3/GALNT15/AP3S1/FAT3/CLN5/MRPL52/CCR1/SLC51B/SEZ6/TNFAIP8L1/CNP/NEU4/COL9A3/COMP/MAP3K8/ADM/IL31RA/EGFLAM/HUS1B/OR2A14/CPS1/NDUFAF6/PXDNL/CRABP1/ZNF358/CRYBB3/MIB2/PARP4/MPP7/FAM101A/B3GLCT/MGAT5B/CSTA/KLC3/ABCC13/SMYD1/SGOL1/PPM1L/SH3D19/CYLD/MBOAT1/ESCO2/CYP11A1/DDB1/DDOST/ZNF366/BHLHA15/DIO3/DLG2/DMBT1/DNAH6/ABAT/DPH1/DTNA/AGXT/EEF2/EFNA2/EIF4G1/A2M/ELK4/LIPH/SLC10A4/ENO2/EPHA1/ESR1/F11/FAH/FAT2/SPATA13/FCGR2A/PHACTR1/FGA/FGF10/FHIT/BTBD3/SBNO2/TRAK1/MSRB2/FOXL1/FOXC2/EXPH5/AKR1B1/NFASC/EPB41L3/DIP2A/FLOT2/MLC1/RHOBTB2/NUP210/NEDD4L/SYNE1/PSD3/PPP1R13B/PUM2/ARHGEF18/RYBP/MORC3/MAPK8IP2/TSSK2/VGLL2/SLC37A4/RASGEF1C/RNF144B/ST6GALNAC3/SAMM50/DFNB31/ALS2CL/PNKD/ACOT11/STEAP2/GAS2/LCE2B/GATM/GBGT1/PLEK2/SLC17A5/ADGRF1/RPS6KC1/PABPC1/FGF22/NPTN/CLUL1/PDE7B/CYTH4/AMPD3/DHDH/BMP10/THEM5/GPR26/EOGT/FFAR2/GRB10/MRPS18B/GRIK4/DNAJC15/SCG3/TMOD4/GUCY1A3/GPR132/PADI1/HAS1/SOX8/HK1/HLA-DOA/HLA-DPA1/ANXA13/NR4A1/ACACB/HOXC4/HRH1/HSD11B1/HSPA1L/HSP90AB1/HTR3A/TFAP2E/ID3/ZC3H12D/RSPO2/NME9/IGF1/IGF2/GPR142/LCE1C/LCE1D/LCE2D/IL1R1/IL1RN/IL10RA/AQP2/IL11RA/IL12RB2/IL15RA/IL16/FOXK2/AQP5/INHBA/INPP5A/IRF1/AQP9/ITGA7/ITGB2/ITGB7/IVL/JUP/CD82/USP50/KCNH2/KCNJ8/KCNJ9/KCNMB1/KDR/KIF25/IPO5/AMIGO3/HES5/AFF3/LAMA3/STMN1/OR2A5/LCP1/MUC21/LDLR/ARHGDIA/LGALS9/LHCGR/LMNA/LMO2/RAB19/MC2R/MCC/ME2/MFNG/MGAT1/SCGB2A1/MITF/LHX8/ASGR1/MOCS1/MOV10/MPZ/PLEKHG7/MYH4/MYL2/NUBP1/NDUFB4/DRG1/NEU1/ATP1A2/NFATC3/NHLH2/NPPC/NRAS/OAS2/OPRL1/OR2C1/OR3A2/SLC22A18/P2RY6/PAFAH2/ATP5B/IL21R/ANO7/PARK2/SPOCK3/UTP11L/PRR16/CHST15/PCYOX1/C11orf73/SIRT6/PDE6B/ATP8A2/PITX2/PKHD1/PKM/PLA2G2A/PLAGL1/PRKAG3/PML/RIPPLY3/FXYD6/GPR84/SLCO1C1/PNLIP/TLR9/TREM1/SSH1/RIN2/APBB1IP/MXRA8/FBLIM1/PALMD/CYP2W1/BANP/PPP1CB/HERC6/ELP3/GOLPH3L/PPP2R2B/FANCI/MOB1A/SLC47A1/SLC29A3/MIS18BP1/TRPV6/SLC30A10/CHRNA9/PEX26/FRMD4A/PARVA/PRKAR1B/TTC17/IFT122/ERMARD/MCTP2/LMBRD1/CSGALNACT1/PAG1/CISD1/WSB2/APOBR/MAPK3/MAP2K2/PRKRIR/PROC/MRAP/TRPV5/PRMT8/HTRA1/SLAMF8/CDC42SE1/PAK6/RGMA/PSMD7/SLURP1/PTGFR/PLEKHG5/TENM2/ERMN/RDH14/MARK4/PTPRE/FAM60A/ACTA2/RFC2/TRIM27/RGR/RGS12/RIT2/RPA3/BGLAP/SCT/CCL11/ABHD4/MRPS14/NPAS3/PARVG/NOD2/TINAGL1/STRA6/ARHGAP9/GZF1/DNAI2/TMEM237/VPS33A/BMP4/SLC4A1/SLC6A12/SLC8A1/SLC9A3/SLC20A2/BMPR1B/SLIT1/BOK/BPI/SRP68/STAT2/STK3/SUPT6H/BST2/ZEB1/TEAD3/TERF1/TGM2/TCHH/TNFAIP3/TNFRSF1A/TRAF5/TRPC6/TRPM2/CCR2/TNFRSF4/UCP1/WNT10B/ZAP70/ZNF7/CA7/CACNA1E/MOGS/RAB7A/CARD14/BCL2L14/LST1/CERS4/CSPP1/CALD1/FAM188A/GPR157/ZC3H12A/FAAP100/CPEB4/C6orf25/EEPD1/CLPTM1L/CALR/COL21A1/QTRT1/SLIRP/CAST/CAPZB/DYNLRB2/SLC25A18/SPATA16/ANTXR1/MFSD7/CMAHP/BFSP2/ATP13A4/NR0B2/MON1A/CASQ1/HOPX/PARD6G/TTBK1/RETNLB/TRIM63/RAE1/SLC43A1/IFITM1/SCIN/CDK10/KMO/RUNX1/TP63/RUNX3/SERPINA6/IRS2/ACTN1/CRADD/TNFRSF11A/ALDH1A2/SYNJ2/SPHK1/CCNA1/SKAP2/STBD1/TSPAN18/CH25H/CCRL2/ERI1/PRC1/STARD13/PIAS2/MAP3K6/SYT7/ESAM/SLC16A3/CBFA2T2/RSAD2/AURKB/DAPL1/CD8A/TRIP10/ADIPOQ/ENTPD3/RAB36/MICAL2/NUP93/RAPGEF2/CD79A/IQSEC1/LPGAT1/FGF19/NR1H4	503
GO:0065007	biological regulation	431/590	10343/17046	9.59E-11	1.00E-07	8.68E-08	AKT3/ABI1/TANK/ZNF783/FARP1/KLRG1/RCAN2/CDKN1C/SPEG/MRVI1/TRDN/SPON2/PITRM1/TACC2/PDPN/CELF1/GJB6/PNRC1/TMED10/LECT1/RER1/ESM1/HNRNPUL1/CHGA/CHI3L1/ERLIN2/EGLN2/ATXN2L/PDAP1/CHRNA1/CHRNA2/CARD16/SORCS1/AP3S1/CLN5/CCR1/SLC51B/SEZ6/TNFAIP8L1/CNP/COMP/MAP3K8/ADM/IL31RA/EGFLAM/HUS1B/OR2A14/CPS1/CRABP1/ZNF358/MIB2/MPP7/LDLRAD3/FAM101A/CSTA/ZNF738/SMYD1/SGOL1/PPM1L/SH3D19/CYLD/ESCO2/CYP11A1/ZNF782/DDB1/ZNF366/BHLHA15/DIO3/DLG2/DMBT1/ABAT/DTNA/AGXT/EEF2/EFNA2/EIF4G1/A2M/ELK4/EPHA1/ESR1/F11/SPATA13/FCGR2A/PHACTR1/FGA/FGF10/FHIT/SBNO2/TRAK1/MSRB2/FOXL1/FOXC2/EXPH5/AKR1B1/EPB41L3/GGA3/DIP2A/FLOT2/MLC1/TBC1D1/RHOBTB2/NUP210/NEDD4L/SYNE1/PSD3/PPP1R13B/PUM2/ARHGEF18/RYBP/MORC3/MAPK8IP2/TSSK2/VGLL2/SLC37A4/RASGEF1C/RNF144B/ZNF549/DFNB31/ALS2CL/PNKD/ACOT11/STEAP2/GAS2/FBXL21/SACS/PLEK2/ADGRF1/RPS6KC1/PABPC1/DNAJC2/FGF22/NPTN/PDE7B/CYTH4/AMPD3/BMP10/ZNF638/ZNF311/ZNF844/GPR26/FFAR2/GRB10/GRIK4/DNAJC15/SCG3/TMOD4/GUCY1A3/GPR132/HAS1/SOX8/HK1/HLA-DOA/HLA-DPA1/ANXA13/NR4A1/ACACB/HOXC4/AGFG2/HRH1/HSPA1L/HSP90AB1/HTR3A/TFAP2E/ID3/ZC3H12D/RSPO2/NME9/IGF1/IGF2/GPR142/IL1R1/IL1RN/IL10RA/AQP2/IL11RA/IL12RB2/IL15RA/IL16/FOXK2/AQP5/INHBA/IRF1/AQP9/ITGA7/ITGB2/ITGB7/ITIH4/JUP/CD82/USP50/KCNH2/KCNJ8/KCNJ9/KCNMB1/KDR/KIF25/IPO5/AMIGO3/HES5/AFF3/LAMA3/STMN1/OR2A5/LCP1/LDLR/ARHGDIA/LGALS9/LHCGR/LMNA/LMO2/RAB19/MC2R/MCC/ME2/MFNG/SCGB2A1/MITF/LHX8/MOV10/MPZ/PLEKHG7/MYL2/NUBP1/NEU1/ATP1A2/NFATC3/NHLH2/NPPC/NRAS/OAS2/OPRL1/OR2C1/OR3A2/P2RY6/PAFAH2/ATP5B/IL21R/ANO7/PARK2/SPOCK3/UTP11L/PRR16/C11orf73/SIRT6/PDE6B/ATP8A2/PI3/PITX2/PKHD1/PLA2G2A/PLAGL1/PRKAG3/PML/RIPPLY3/FXYD6/GPR84/PNLIP/TLR9/TREM1/SSH1/RIN2/POU2AF1/APBB1IP/FBLIM1/MED18/PALMD/BANP/PPP1CB/ELP3/DNAJC17/GOLPH3L/PPP2R2B/FANCI/MOB1A/TRPV6/SLC30A10/CHRNA9/PARVA/PRKAR1B/IFT122/MCTP2/LMBRD1/PAG1/CISD1/WSB2/MYNN/MAPK3/MAP2K2/PRKRIR/PROC/MRAP/PRMT8/HTRA1/SLAMF8/CDC42SE1/PAK6/ARNTL2/RGMA/PRDM11/PSMD7/SLURP1/PTGFR/PLEKHG5/TENM2/GATAD2B/ERMN/RDH14/MARK4/PTPRE/CREBZF/FAM60A/ACTA2/RFC2/TRIM27/RGR/RGS12/RIT2/RPA3/BGLAP/SCT/CCL11/ABHD4/NPAS3/NOD2/STRA6/ARHGAP9/GZF1/TMEM237/VPS33A/BMP4/SLC4A1/ZNF649/SLC6A12/SLC8A1/SLC9A3/BMPR1B/SLIT1/BRD9/ZSCAN18/BOK/BPI/STAT2/STK3/SUPT6H/BST2/TCEB2/ZEB1/TEAD3/TERF1/TGM2/TNFAIP3/TNFRSF1A/TRAF5/TRPC6/CCR2/TNFRSF4/UCP1/WNT10B/ZAP70/ZNF7/CA7/CACNA1E/RAB7A/CARD14/BCL2L14/LST1/ZNF665/CSPP1/GPR157/ZC3H12A/CPEB4/C6orf25/ZNF436/CALR/SLIRP/CAST/CAPZB/ANTXR1/CMAHP/ATP13A4/ZNF397/NR0B2/CASQ1/HOPX/TTBK1/TRIM63/RAE1/IFITM1/SCIN/CDK10/RUNX1/TP63/RUNX3/SERPINA6/IRS2/ACTN1/CRADD/TNFRSF11A/ALDH1A2/SPHK1/BUD31/CCNA1/SKAP2/TSPAN18/CCRL2/ERI1/PRC1/STARD13/PIAS2/MAP3K6/SYT7/ESAM/SLC16A3/CBFA2T2/RSAD2/AURKB/DAPL1/CD8A/TRIP10/ADIPOQ/RAB36/MICAL2/N4BP1/VGLL4/NUP93/RAPGEF2/CD79A/ZBTB39/IQSEC1/FGF19/NR1H4	431

GO:0050794	regulation of cellular process	394/590	9347/17046	1.23E-09	9.87E-07	8.56E-07	AKT3/ABI1/TANK/ZNF783/FARP1/KLRG1/RCAN2/CDKN1C/SPEG/MRVI1/TRDN/TACC2/PDPN/CELF1/GJB6/PNRC1/TMED10/LECT1/RER1/ESM1/HNRNPUL1/CHGA/CHI3L1/ERLIN2/EGLN2/ATXN2L/PDAP1/CHRNA1/CHRNA2/CARD16/SORCS1/AP3S1/CCR1/SLC51B/SEZ6/TNFAIP8L1/COMP/MAP3K8/ADM/IL31RA/EGFLAM/HUS1B/OR2A14/CRABP1/ZNF358/MIB2/MPP7/FAM101A/CSTA/ZNF738/SMYD1/SGOL1/PPM1L/SH3D19/CYLD/ESCO2/ZNF782/DDB1/ZNF366/BHLHA15/DLG2/DMBT1/ABAT/DTNA/AGXT/EEF2/EFNA2/EIF4G1/A2M/ELK4/EPHA1/ESR1/SPATA13/FCGR2A/FGA/FGF10/FHIT/SBNO2/TRAK1/MSRB2/FOXL1/FOXC2/EXPH5/AKR1B1/EPB41L3/DIP2A/FLOT2/MLC1/RHOBTB2/NUP210/NEDD4L/PSD3/PPP1R13B/PUM2/ARHGEF18/RYBP/MORC3/MAPK8IP2/TSSK2/VGLL2/RASGEF1C/RNF144B/ZNF549/ALS2CL/PNKD/ACOT11/GAS2/SACS/PLEK2/ADGRF1/RPS6KC1/PABPC1/DNAJC2/FGF22/NPTN/PDE7B/CYTH4/BMP10/ZNF638/ZNF311/ZNF844/GPR26/FFAR2/GRB10/GRIK4/DNAJC15/TMOD4/GUCY1A3/GPR132/HAS1/SOX8/HK1/HLA-DOA/HLA-DPA1/ANXA13/NR4A1/ACACB/HOXC4/HRH1/HSPA1L/HSP90AB1/HTR3A/TFAP2E/ID3/ZC3H12D/RSPO2/NME9/IGF1/IGF2/GPR142/IL1R1/IL1RN/IL10RA/IL11RA/IL12RB2/IL15RA/IL16/FOXK2/INHBA/IRF1/ITGA7/ITGB2/ITGB7/ITIH4/JUP/CD82/USP50/KCNH2/KCNJ8/KCNJ9/KDR/KIF25/IPO5/AMIGO3/HES5/AFF3/LAMA3/STMN1/OR2A5/LCP1/LDLR/ARHGDIA/LGALS9/LHCGR/LMNA/LMO2/RAB19/MC2R/MCC/ME2/MFNG/SCGB2A1/MITF/LHX8/MOV10/MPZ/PLEKHG7/MYL2/NUBP1/NEU1/ATP1A2/NFATC3/NHLH2/NPPC/NRAS/OAS2/OPRL1/OR2C1/OR3A2/P2RY6/PAFAH2/ATP5B/IL21R/PARK2/SPOCK3/UTP11L/PRR16/C11orf73/SIRT6/PDE6B/ATP8A2/PI3/PITX2/PKHD1/PLA2G2A/PLAGL1/PRKAG3/PML/RIPPLY3/FXYD6/GPR84/PNLIP/TLR9/TREM1/SSH1/RIN2/POU2AF1/APBB1IP/FBLIM1/MED18/PALMD/BANP/PPP1CB/ELP3/DNAJC17/GOLPH3L/PPP2R2B/FANCI/MOB1A/TRPV6/SLC30A10/CHRNA9/PARVA/PRKAR1B/IFT122/MCTP2/LMBRD1/PAG1/CISD1/WSB2/MYNN/MAPK3/MAP2K2/PRKRIR/PROC/MRAP/PRMT8/HTRA1/SLAMF8/CDC42SE1/PAK6/ARNTL2/RGMA/PRDM11/PSMD7/SLURP1/PTGFR/PLEKHG5/TENM2/GATAD2B/ERMN/MARK4/PTPRE/CREBZF/FAM60A/TRIM27/RGR/RGS12/RIT2/RPA3/BGLAP/SCT/CCL11/NPAS3/NOD2/STRA6/ARHGAP9/GZF1/TMEM237/BMP4/ZNF649/SLC8A1/BMPR1B/SLIT1/BRD9/ZSCAN18/BOK/BPI/STAT2/STK3/SUPT6H/BST2/TCEB2/ZEB1/TEAD3/TERF1/TGM2/TNFAIP3/TNFRSF1A/TRAF5/TRPC6/CCR2/TNFRSF4/UCP1/WNT10B/ZAP70/ZNF7/CA7/CACNA1E/RAB7A/CARD14/BCL2L14/LST1/ZNF665/CSPP1/GPR157/ZC3H12A/CPEB4/C6orf25/ZNF436/CALR/SLIRP/CAST/CAPZB/ANTXR1/CMAHP/ZNF397/NR0B2/CASQ1/HOPX/TTBK1/TRIM63/RAE1/IFITM1/SCIN/CDK10/RUNX1/TP63/RUNX3/SERPINA6/IRS2/ACTN1/CRADD/TNFRSF11A/ALDH1A2/SPHK1/BUD31/CCNA1/SKAP2/TSPAN18/CCRL2/PRC1/STARD13/PIAS2/MAP3K6/SYT7/CBFA2T2/RSAD2/AURKB/DAPL1/CD8A/TRIP10/ADIPOQ/RAB36/MICAL2/N4BP1/VGLL4/NUP93/RAPGEF2/CD79A/ZBTB39/IQSEC1/FGF19/NR1H4	394
GO:0050789	regulation of biological process	410/590	9837/17046	1.32E-09	9.87E-07	8.56E-07	AKT3/ABI1/TANK/ZNF783/FARP1/KLRG1/RCAN2/CDKN1C/SPEG/MRVI1/TRDN/SPON2/PITRM1/TACC2/PDPN/CELF1/GJB6/PNRC1/TMED10/LECT1/RER1/ESM1/HNRNPUL1/CHGA/CHI3L1/ERLIN2/EGLN2/ATXN2L/PDAP1/CHRNA1/CHRNA2/CARD16/SORCS1/AP3S1/CCR1/SLC51B/SEZ6/TNFAIP8L1/COMP/MAP3K8/ADM/IL31RA/EGFLAM/HUS1B/OR2A14/CPS1/CRABP1/ZNF358/MIB2/MPP7/LDLRAD3/FAM101A/CSTA/ZNF738/SMYD1/SGOL1/PPM1L/SH3D19/CYLD/ESCO2/ZNF782/DDB1/ZNF366/BHLHA15/DIO3/DLG2/DMBT1/ABAT/DTNA/AGXT/EEF2/EFNA2/EIF4G1/A2M/ELK4/EPHA1/ESR1/F11/SPATA13/FCGR2A/PHACTR1/FGA/FGF10/FHIT/SBNO2/TRAK1/MSRB2/FOXL1/FOXC2/EXPH5/AKR1B1/EPB41L3/GGA3/DIP2A/FLOT2/MLC1/TBC1D1/RHOBTB2/NUP210/NEDD4L/PSD3/PPP1R13B/PUM2/ARHGEF18/RYBP/MORC3/MAPK8IP2/TSSK2/VGLL2/SLC37A4/RASGEF1C/RNF144B/ZNF549/ALS2CL/PNKD/ACOT11/GAS2/FBXL21/SACS/PLEK2/ADGRF1/RPS6KC1/PABPC1/DNAJC2/FGF22/NPTN/PDE7B/CYTH4/BMP10/ZNF638/ZNF311/ZNF844/GPR26/FFAR2/GRB10/GRIK4/DNAJC15/TMOD4/GUCY1A3/GPR132/HAS1/SOX8/HK1/HLA-DOA/HLA-DPA1/ANXA13/NR4A1/ACACB/HOXC4/AGFG2/HRH1/HSPA1L/HSP90AB1/HTR3A/TFAP2E/ID3/ZC3H12D/RSPO2/NME9/IGF1/IGF2/GPR142/IL1R1/IL1RN/IL10RA/IL11RA/IL12RB2/IL15RA/IL16/FOXK2/INHBA/IRF1/ITGA7/ITGB2/ITGB7/ITIH4/JUP/CD82/USP50/KCNH2/KCNJ8/KCNJ9/KDR/KIF25/IPO5/AMIGO3/HES5/AFF3/LAMA3/STMN1/OR2A5/LCP1/LDLR/ARHGDIA/LGALS9/LHCGR/LMNA/LMO2/RAB19/MC2R/MCC/ME2/MFNG/SCGB2A1/MITF/LHX8/MOV10/MPZ/PLEKHG7/MYL2/NUBP1/NEU1/ATP1A2/NFATC3/NHLH2/NPPC/NRAS/OAS2/OPRL1/OR2C1/OR3A2/P2RY6/PAFAH2/ATP5B/IL21R/PARK2/SPOCK3/UTP11L/PRR16/C11orf73/SIRT6/PDE6B/ATP8A2/PI3/PITX2/PKHD1/PLA2G2A/PLAGL1/PRKAG3/PML/RIPPLY3/FXYD6/GPR84/PNLIP/TLR9/TREM1/SSH1/RIN2/POU2AF1/APBB1IP/FBLIM1/MED18/PALMD/BANP/PPP1CB/ELP3/DNAJC17/GOLPH3L/PPP2R2B/FANCI/MOB1A/TRPV6/SLC30A10/CHRNA9/PARVA/PRKAR1B/IFT122/MCTP2/LMBRD1/PAG1/CISD1/WSB2/MYNN/MAPK3/MAP2K2/PRKRIR/PROC/MRAP/PRMT8/HTRA1/SLAMF8/CDC42SE1/PAK6/ARNTL2/RGMA/PRDM11/PSMD7/SLURP1/PTGFR/PLEKHG5/TENM2/GATAD2B/ERMN/MARK4/PTPRE/CREBZF/FAM60A/ACTA2/TRIM27/RGR/RGS12/RIT2/RPA3/BGLAP/SCT/CCL11/NPAS3/NOD2/STRA6/ARHGAP9/GZF1/TMEM237/VPS33A/BMP4/ZNF649/SLC8A1/SLC9A3/BMPR1B/SLIT1/BRD9/ZSCAN18/BOK/BPI/STAT2/STK3/SUPT6H/BST2/TCEB2/ZEB1/TEAD3/TERF1/TGM2/TNFAIP3/TNFRSF1A/TRAF5/TRPC6/CCR2/TNFRSF4/UCP1/WNT10B/ZAP70/ZNF7/CA7/CACNA1E/RAB7A/CARD14/BCL2L14/LST1/ZNF665/CSPP1/GPR157/ZC3H12A/CPEB4/C6orf25/ZNF436/CALR/SLIRP/CAST/CAPZB/ANTXR1/CMAHP/ZNF397/NR0B2/CASQ1/HOPX/TTBK1/TRIM63/RAE1/IFITM1/SCIN/CDK10/RUNX1/TP63/RUNX3/SERPINA6/IRS2/ACTN1/CRADD/TNFRSF11A/ALDH1A2/SPHK1/BUD31/CCNA1/SKAP2/TSPAN18/CCRL2/ERI1/PRC1/STARD13/PIAS2/MAP3K6/SYT7/CBFA2T2/RSAD2/AURKB/DAPL1/CD8A/TRIP10/ADIPOQ/RAB36/MICAL2/N4BP1/VGLL4/NUP93/RAPGEF2/CD79A/ZBTB39/IQSEC1/FGF19/NR1H4	410

GO:0050896	response to stimulus	333/590	7634/17046	4.93E-09	3.21E-06	2.79E-06	AKT3/ABI1/TANK/FARP1/KLRG1/RCAN2/CDKN1C/MRVI1/SPON2/PDPN/GJB6/TMED10/LECT1/ESM1/HNRNPUL1/CHGA/CHI3L1/ERLIN2/EGLN2/TP53TG1/PDAP1/CHRNA1/CHRNA2/SORCS1/AP3S1/CCR1/SEZ6/TNFAIP8L1/CNP/COL9A3/MAP3K8/ADM/IL31RA/HUS1B/OR2A14/CPS1/PXDNL/CRABP1/MIB2/PARP4/MPP7/SGOL1/PPM1L/CYLD/ESCO2/CYP11A1/DDB1/DDOST/ZNF366/BHLHA15/DMBT1/ABAT/DTNA/AGXT/EFNA2/EIF4G1/A2M/ANKRD23/EPHA1/ESR1/F11/SPATA13/FCGR2A/FGA/FGF10/FHIT/SBNO2/MSRB2/FOXL1/FOXC2/AKR1B1/NFASC/MLC1/RHOBTB2/NUP210/NEDD4L/PSD3/PPP1R13B/PUM2/ARHGEF18/MAPK8IP2/TSSK2/SLC37A4/RASGEF1C/ALS2CL/ACOT11/STEAP2/FBXL21/GATM/PLEK2/ADGRF1/RPS6KC1/DNAJC2/FGF22/NPTN/PDE7B/CYTH4/BMP10/GPR26/FFAR2/GRB10/GRIK4/DNAJC15/SCG3/GUCY1A3/GPR132/HAS1/SOX8/HK1/HLA-DOA/HLA-DPA1/NR4A1/HRH1/HSPA1L/HSP90AB1/HTR3A/ID3/RSPO2/CD300E/IGF1/IGF2/GPR142/LCE1D/IL1R1/IL1RN/IL10RA/AQP2/IL11RA/IL12RB2/IL15RA/IL16/INHBA/IRF1/AQP9/ITGA7/ITGB2/ITGB7/ITIH4/IVL/JUP/CD82/KCNH2/KCNJ8/KCNMB1/KDR/IPO5/HES5/AFF3/STMN1/OR2A5/LCP1/LDLR/ARHGDIA/LGALS9/LHCGR/LMNA/LMO2/RAB19/MC2R/MCC/MFNG/SCGB2A1/MITF/ASGR1/MOV10/PLEKHG7/MYH4/NDUFB4/ATP1A2/NFATC3/NPPC/NRAS/OAS2/OPRL1/OR2C1/OR3A2/SLC22A18/P2RY6/IL21R/PARK2/SPOCK3/C11orf73/SIRT6/PDE6B/ATP8A2/PITX2/PKHD1/PKM/PLA2G2A/PRKAG3/PML/GPR84/PNLIP/TLR9/TREM1/SSH1/RIN2/POU2AF1/APBB1IP/CYP2W1/PPP1CB/FANCI/MOB1A/SLC47A1/SLC30A10/CHRNA9/PARVA/PRKAR1B/IFT122/MCTP2/LMBRD1/PAG1/WSB2/MAPK3/MAP2K2/PRKRIR/PROC/HTRA1/SLAMF8/CDC42SE1/PAK6/ARNTL2/RGMA/PSMD7/PTGFR/PLEKHG5/TENM2/PTPRE/CREBZF/ACTA2/RFC2/TRIM27/RGR/RGS12/RIT2/RPA3/BGLAP/SCT/CCL11/NPAS3/NOD2/TINAGL1/STRA6/ARHGAP9/TMEM237/VPS33A/BMP4/SLC8A1/SLC9A3/BMPR1B/SLIT1/BOK/BPI/SRP68/STAT2/STK3/SUPT6H/BST2/TCEB2/ZEB1/TEAD3/TERF1/TGM2/TNFAIP3/TNFRSF1A/TRAF5/TRPC6/TRPM2/CCR2/TNFRSF4/WNT10B/ZAP70/CA7/CACNA1E/RAB7A/CARD14/BCL2L14/LST1/GPR157/ZC3H12A/FAAP100/CPEB4/C6orf25/EEPD1/CALR/CAPZB/ANTXR1/CMAHP/BFSP2/NR0B2/CASQ1/HOPX/TRIM63/RAE1/IFITM1/CDK10/KMO/RUNX1/TP63/RUNX3/IRS2/ACTN1/CRADD/TNFRSF11A/ALDH1A2/SPHK1/ENDOU/SKAP2/STBD1/HSPB3/TSPAN18/CCRL2/PRC1/STARD13/PIAS2/MAP3K6/SYT7/ESAM/SLC16A3/RSAD2/AURKB/DAPL1/CD8A/TRIP10/ADIPOQ/RAB36/N4BP1/NUP93/RAPGEF2/CD79A/IQSEC1/FGF19/NR1H4	333
GO:0007154	cell communication	258/590	5832/17046	6.74E-07	0.00039	0.00034	AKT3/ABI1/TANK/FARP1/KLRG1/RCAN2/CDKN1C/MRVI1/TRDN/PDPN/GJB6/LECT1/ESM1/CHI3L1/ERLIN2/EGLN2/PDAP1/CHRNA1/CHRNA2/SORCS1/PANX3/AP3S1/CCR1/SEZ6/TNFAIP8L1/CNP/MAP3K8/ADM/IL31RA/OR2A14/CRABP1/MIB2/MPP7/SGOL1/PPM1L/CYLD/DDB1/ZNF366/BHLHA15/DLG2/DMBT1/ABAT/DTNA/AGXT/EFNA2/EIF4G1/A2M/EPHA1/ESR1/SPATA13/FCGR2A/FGA/FGF10/FHIT/FOXL1/FOXC2/AKR1B1/NFASC/RHOBTB2/NUP210/NEDD4L/PSD3/PPP1R13B/PUM2/ARHGEF18/MAPK8IP2/TSSK2/SLC37A4/RASGEF1C/ALS2CL/PNKD/ACOT11/PLEK2/ADGRF1/RPS6KC1/FGF22/NPTN/PDE7B/CYTH4/BMP10/GPR26/FFAR2/GRB10/GRIK4/DNAJC15/GUCY1A3/GPR132/SOX8/HK1/HLA-DOA/HLA-DPA1/NR4A1/HRH1/HSP90AB1/HTR3A/RSPO2/IGF1/IGF2/GPR142/IL1R1/IL1RN/IL10RA/AQP2/IL11RA/IL12RB2/IL15RA/INHBA/IRF1/ITGA7/ITGB2/ITGB7/JUP/CD82/KCNH2/KCNJ8/KCNJ9/KCNMB1/KDR/HES5/STMN1/OR2A5/LCP1/LDLR/ARHGDIA/LGALS9/LHCGR/LMNA/RAB19/MC2R/MCC/MFNG/SCGB2A1/MITF/ASGR1/MOV10/MPZ/PLEKHG7/ATP1A2/NFATC3/NPPC/NRAS/OAS2/OPRL1/OR2C1/OR3A2/P2RY6/IL21R/PARK2/SPOCK3/PDE6B/PITX2/PKHD1/PLA2G2A/PRKAG3/PML/GPR84/PNLIP/TLR9/TREM1/RIN2/APBB1IP/PPP1CB/MOB1A/CHRNA9/PRKAR1B/IFT122/MCTP2/LMBRD1/PAG1/WSB2/MAPK3/MAP2K2/PRKRIR/HTRA1/CDC42SE1/PAK6/RGMA/PSMD7/PTGFR/PLEKHG5/TENM2/PTPRE/RGR/RGS12/RIT2/BGLAP/SCT/CCL11/NOD2/STRA6/ARHGAP9/TMEM237/VPS33A/BMP4/SLC6A12/SLC8A1/BMPR1B/BOK/STAT2/STK3/BST2/ZEB1/TEAD3/TGM2/TNFAIP3/TNFRSF1A/TRAF5/CCR2/TNFRSF4/WNT10B/ZAP70/CA7/CACNA1E/RAB7A/CARD14/BCL2L14/GPR157/CPEB4/C6orf25/CALR/ANTXR1/CMAHP/NR0B2/CASQ1/TRIM63/RAE1/IFITM1/CDK10/RUNX1/TP63/RUNX3/IRS2/CRADD/TNFRSF11A/ALDH1A2/SPHK1/SKAP2/STBD1/TSPAN18/CCRL2/PRC1/STARD13/PIAS2/MAP3K6/SYT7/RSAD2/AURKB/DAPL1/CD8A/TRIP10/ADIPOQ/RAB36/NUP93/RAPGEF2/CD79A/IQSEC1/FGF19/NR1H4	258
GO:0010033	response to organic substance	137/590	2687/17046	9.71E-07	0.00049	0.00043	CDKN1C/SPON2/GJB6/TMED10/LECT1/CHI3L1/ERLIN2/CHRNA1/CHRNA2/AP3S1/CCR1/CNP/ADM/IL31RA/CPS1/CYP11A1/DDOST/ZNF366/BHLHA15/ABAT/AGXT/EIF4G1/ESR1/FGA/FGF10/SBNO2/FOXC2/AKR1B1/MLC1/NUP210/NEDD4L/ARHGEF18/STEAP2/GATM/FGF22/NPTN/BMP10/FFAR2/GRB10/GUCY1A3/HAS1/HLA-DPA1/NR4A1/HRH1/HSPA1L/HSP90AB1/HTR3A/IGF2/IL1R1/IL1RN/IL10RA/IL11RA/IL12RB2/IL15RA/INHBA/IRF1/AQP9/ITIH4/JUP/KCNJ8/KDR/IPO5/HES5/AFF3/ARHGDIA/LGALS9/LHCGR/LMNA/LMO2/MOV10/ATP1A2/NPPC/NRAS/OAS2/OPRL1/P2RY6/IL21R/PARK2/PITX2/PKM/PRKAG3/PML/TLR9/SSH1/PPP1CB/CHRNA9/PRKAR1B/LMBRD1/MAPK3/MAP2K2/HTRA1/RGMA/PSMD7/PTGFR/PLEKHG5/PTPRE/RIT2/BGLAP/CCL11/NOD2/BMP4/SLC8A1/SLC9A3/BMPR1B/STAT2/BST2/ZEB1/TNFAIP3/TNFRSF1A/CCR2/TNFRSF4/WNT10B/CACNA1E/RAB7A/CARD14/ZC3H12A/CPEB4/CALR/NR0B2/CASQ1/TRIM63/RAE1/IFITM1/RUNX1/RUNX3/IRS2/TNFRSF11A/ALDH1A2/SPHK1/HSPB3/CCRL2/RSAD2/ADIPOQ/NUP93/RAPGEF2/FGF19/NR1H4	137
GO:0007165	signal transduction	233/590	5179/17046	1.03E-06	0.00049	0.00043	AKT3/ABI1/TANK/FARP1/KLRG1/RCAN2/CDKN1C/MRVI1/PDPN/LECT1/ESM1/CHI3L1/ERLIN2/EGLN2/PDAP1/CHRNA1/CHRNA2/SORCS1/AP3S1/CCR1/SEZ6/TNFAIP8L1/MAP3K8/ADM/IL31RA/OR2A14/CRABP1/MIB2/MPP7/SGOL1/PPM1L/CYLD/DDB1/ZNF366/BHLHA15/DMBT1/DTNA/AGXT/EFNA2/EIF4G1/A2M/EPHA1/ESR1/SPATA13/FCGR2A/FGA/FGF10/FHIT/FOXL1/FOXC2/AKR1B1/RHOBTB2/NUP210/NEDD4L/PSD3/PPP1R13B/PUM2/ARHGEF18/MAPK8IP2/TSSK2/RASGEF1C/ALS2CL/ACOT11/PLEK2/ADGRF1/RPS6KC1/FGF22/NPTN/PDE7B/CYTH4/BMP10/GPR26/FFAR2/GRB10/GRIK4/GUCY1A3/GPR132/SOX8/HLA-DOA/HLA-DPA1/NR4A1/HRH1/HSP90AB1/HTR3A/RSPO2/IGF1/IGF2/GPR142/IL1R1/IL1RN/IL10RA/IL11RA/IL12RB2/IL15RA/INHBA/IRF1/ITGA7/ITGB2/ITGB7/JUP/CD82/KCNH2/KDR/HES5/STMN1/OR2A5/LCP1/LDLR/ARHGDIA/LGALS9/LHCGR/LMNA/RAB19/MC2R/MCC/MFNG/SCGB2A1/MITF/MOV10/PLEKHG7/ATP1A2/NFATC3/NPPC/NRAS/OAS2/OPRL1/OR2C1/OR3A2/P2RY6/IL21R/PARK2/SPOCK3/PDE6B/PITX2/PKHD1/PLA2G2A/PRKAG3/PML/GPR84/PNLIP/TLR9/TREM1/RIN2/APBB1IP/PPP1CB/MOB1A/CHRNA9/PRKAR1B/IFT122/MCTP2/LMBRD1/PAG1/WSB2/MAPK3/MAP2K2/PRKRIR/HTRA1/CDC42SE1/PAK6/RGMA/PSMD7/PTGFR/PLEKHG5/TENM2/PTPRE/RGR/RGS12/RIT2/CCL11/NOD2/STRA6/ARHGAP9/TMEM237/BMP4/SLC8A1/BMPR1B/BOK/STAT2/STK3/BST2/ZEB1/TEAD3/TGM2/TNFAIP3/TNFRSF1A/TRAF5/CCR2/TNFRSF4/WNT10B/ZAP70/RAB7A/CARD14/BCL2L14/GPR157/CPEB4/C6orf25/CALR/ANTXR1/CMAHP/NR0B2/CASQ1/TRIM63/RAE1/IFITM1/CDK10/RUNX1/TP63/RUNX3/IRS2/CRADD/TNFRSF11A/ALDH1A2/SPHK1/SKAP2/TSPAN18/CCRL2/PRC1/STARD13/PIAS2/MAP3K6/RSAD2/AURKB/DAPL1/CD8A/TRIP10/ADIPOQ/RAB36/NUP93/RAPGEF2/CD79A/IQSEC1/FGF19/NR1H4	233

GO:0044700	single organism signaling	249/590	5624/17046	1.22E-06	0.00052	0.00045	AKT3/ABI1/TANK/FARP1/KLRG1/RCAN2/CDKN1C/MRVI1/TRDN/PDPN/LECT1/ESM1/CHI3L1/ERLIN2/EGLN2/PDAP1/CHRNA1/CHRNA2/SORCS1/PANX3/AP3S1/CCR1/SEZ6/TNFAIP8L1/CNP/MAP3K8/ADM/IL31RA/OR2A14/CRABP1/MIB2/MPP7/SGOL1/PPM1L/CYLD/DDB1/ZNF366/BHLHA15/DLG2/DMBT1/ABAT/DTNA/AGXT/EFNA2/EIF4G1/A2M/EPHA1/ESR1/SPATA13/FCGR2A/FGA/FGF10/FHIT/FOXL1/FOXC2/AKR1B1/NFASC/RHOBTB2/NUP210/NEDD4L/PSD3/PPP1R13B/PUM2/ARHGEF18/MAPK8IP2/TSSK2/RASGEF1C/ALS2CL/PNKD/ACOT11/PLEK2/ADGRF1/RPS6KC1/FGF22/NPTN/PDE7B/CYTH4/BMP10/GPR26/FFAR2/GRB10/GRIK4/GUCY1A3/GPR132/SOX8/HLA-DOA/HLA-DPA1/NR4A1/HRH1/HSP90AB1/HTR3A/RSPO2/IGF1/IGF2/GPR142/IL1R1/IL1RN/IL10RA/IL11RA/IL12RB2/IL15RA/INHBA/IRF1/ITGA7/ITGB2/ITGB7/JUP/CD82/KCNH2/KCNJ8/KCNJ9/KCNMB1/KDR/HES5/STMN1/OR2A5/LCP1/LDLR/ARHGDIA/LGALS9/LHCGR/LMNA/RAB19/MC2R/MCC/MFNG/SCGB2A1/MITF/MOV10/MPZ/PLEKHG7/ATP1A2/NFATC3/NPPC/NRAS/OAS2/OPRL1/OR2C1/OR3A2/P2RY6/IL21R/PARK2/SPOCK3/PDE6B/PITX2/PKHD1/PLA2G2A/PRKAG3/PML/GPR84/PNLIP/TLR9/TREM1/RIN2/APBB1IP/PPP1CB/MOB1A/CHRNA9/PRKAR1B/IFT122/MCTP2/LMBRD1/PAG1/WSB2/MAPK3/MAP2K2/PRKRIR/HTRA1/CDC42SE1/PAK6/RGMA/PSMD7/PTGFR/PLEKHG5/TENM2/PTPRE/RGR/RGS12/RIT2/SCT/CCL11/NOD2/STRA6/ARHGAP9/TMEM237/BMP4/SLC6A12/SLC8A1/BMPR1B/BOK/STAT2/STK3/BST2/ZEB1/TEAD3/TGM2/TNFAIP3/TNFRSF1A/TRAF5/CCR2/TNFRSF4/WNT10B/ZAP70/CA7/CACNA1E/RAB7A/CARD14/BCL2L14/GPR157/CPEB4/C6orf25/CALR/ANTXR1/CMAHP/NR0B2/CASQ1/TRIM63/RAE1/IFITM1/CDK10/RUNX1/TP63/RUNX3/IRS2/CRADD/TNFRSF11A/ALDH1A2/SPHK1/SKAP2/TSPAN18/CCRL2/PRC1/STARD13/PIAS2/MAP3K6/SYT7/RSAD2/AURKB/DAPL1/CD8A/TRIP10/ADIPOQ/RAB36/NUP93/RAPGEF2/CD79A/IQSEC1/FGF19/NR1H4	249
GO:0023052	signaling	249/590	5629/17046	1.32E-06	0.00052	0.00045	AKT3/ABI1/TANK/FARP1/KLRG1/RCAN2/CDKN1C/MRVI1/TRDN/PDPN/LECT1/ESM1/CHI3L1/ERLIN2/EGLN2/PDAP1/CHRNA1/CHRNA2/SORCS1/PANX3/AP3S1/CCR1/SEZ6/TNFAIP8L1/CNP/MAP3K8/ADM/IL31RA/OR2A14/CRABP1/MIB2/MPP7/SGOL1/PPM1L/CYLD/DDB1/ZNF366/BHLHA15/DLG2/DMBT1/ABAT/DTNA/AGXT/EFNA2/EIF4G1/A2M/EPHA1/ESR1/SPATA13/FCGR2A/FGA/FGF10/FHIT/FOXL1/FOXC2/AKR1B1/NFASC/RHOBTB2/NUP210/NEDD4L/PSD3/PPP1R13B/PUM2/ARHGEF18/MAPK8IP2/TSSK2/RASGEF1C/ALS2CL/PNKD/ACOT11/PLEK2/ADGRF1/RPS6KC1/FGF22/NPTN/PDE7B/CYTH4/BMP10/GPR26/FFAR2/GRB10/GRIK4/GUCY1A3/GPR132/SOX8/HLA-DOA/HLA-DPA1/NR4A1/HRH1/HSP90AB1/HTR3A/RSPO2/IGF1/IGF2/GPR142/IL1R1/IL1RN/IL10RA/IL11RA/IL12RB2/IL15RA/INHBA/IRF1/ITGA7/ITGB2/ITGB7/JUP/CD82/KCNH2/KCNJ8/KCNJ9/KCNMB1/KDR/HES5/STMN1/OR2A5/LCP1/LDLR/ARHGDIA/LGALS9/LHCGR/LMNA/RAB19/MC2R/MCC/MFNG/SCGB2A1/MITF/MOV10/MPZ/PLEKHG7/ATP1A2/NFATC3/NPPC/NRAS/OAS2/OPRL1/OR2C1/OR3A2/P2RY6/IL21R/PARK2/SPOCK3/PDE6B/PITX2/PKHD1/PLA2G2A/PRKAG3/PML/GPR84/PNLIP/TLR9/TREM1/RIN2/APBB1IP/PPP1CB/MOB1A/CHRNA9/PRKAR1B/IFT122/MCTP2/LMBRD1/PAG1/WSB2/MAPK3/MAP2K2/PRKRIR/HTRA1/CDC42SE1/PAK6/RGMA/PSMD7/PTGFR/PLEKHG5/TENM2/PTPRE/RGR/RGS12/RIT2/SCT/CCL11/NOD2/STRA6/ARHGAP9/TMEM237/BMP4/SLC6A12/SLC8A1/BMPR1B/BOK/STAT2/STK3/BST2/ZEB1/TEAD3/TGM2/TNFAIP3/TNFRSF1A/TRAF5/CCR2/TNFRSF4/WNT10B/ZAP70/CA7/CACNA1E/RAB7A/CARD14/BCL2L14/GPR157/CPEB4/C6orf25/CALR/ANTXR1/CMAHP/NR0B2/CASQ1/TRIM63/RAE1/IFITM1/CDK10/RUNX1/TP63/RUNX3/IRS2/CRADD/TNFRSF11A/ALDH1A2/SPHK1/SKAP2/TSPAN18/CCRL2/PRC1/STARD13/PIAS2/MAP3K6/SYT7/RSAD2/AURKB/DAPL1/CD8A/TRIP10/ADIPOQ/RAB36/NUP93/RAPGEF2/CD79A/IQSEC1/FGF19/NR1H4	249
GO:0051716	cellular response to stimulus	277/590	6405/17046	1.40E-06	0.00052	0.00045	AKT3/ABI1/TANK/FARP1/KLRG1/RCAN2/CDKN1C/MRVI1/SPON2/PDPN/GJB6/LECT1/ESM1/CHGA/CHI3L1/ERLIN2/EGLN2/TP53TG1/PDAP1/CHRNA1/CHRNA2/SORCS1/AP3S1/CCR1/SEZ6/TNFAIP8L1/MAP3K8/ADM/IL31RA/HUS1B/OR2A14/CPS1/CRABP1/MIB2/PARP4/MPP7/SGOL1/PPM1L/CYLD/ESCO2/CYP11A1/DDB1/ZNF366/BHLHA15/DMBT1/DTNA/AGXT/EFNA2/EIF4G1/A2M/EPHA1/ESR1/SPATA13/FCGR2A/FGA/FGF10/FHIT/SBNO2/FOXL1/FOXC2/AKR1B1/MLC1/RHOBTB2/NUP210/NEDD4L/PSD3/PPP1R13B/PUM2/ARHGEF18/MAPK8IP2/TSSK2/SLC37A4/RASGEF1C/ALS2CL/ACOT11/PLEK2/ADGRF1/RPS6KC1/DNAJC2/FGF22/NPTN/PDE7B/CYTH4/BMP10/GPR26/FFAR2/GRB10/GRIK4/DNAJC15/GUCY1A3/GPR132/HAS1/SOX8/HK1/HLA-DOA/HLA-DPA1/NR4A1/HRH1/HSPA1L/HSP90AB1/HTR3A/RSPO2/IGF1/IGF2/GPR142/LCE1D/IL1R1/IL1RN/IL10RA/AQP2/IL11RA/IL12RB2/IL15RA/IL16/INHBA/IRF1/AQP9/ITGA7/ITGB2/ITGB7/JUP/CD82/KCNH2/KDR/IPO5/HES5/STMN1/OR2A5/LCP1/LDLR/ARHGDIA/LGALS9/LHCGR/LMNA/LMO2/RAB19/MC2R/MCC/MFNG/SCGB2A1/MITF/ASGR1/MOV10/PLEKHG7/ATP1A2/NFATC3/NPPC/NRAS/OAS2/OPRL1/OR2C1/OR3A2/P2RY6/IL21R/PARK2/SPOCK3/C11orf73/SIRT6/PDE6B/PITX2/PKHD1/PLA2G2A/PRKAG3/PML/GPR84/PNLIP/TLR9/TREM1/SSH1/RIN2/APBB1IP/CYP2W1/PPP1CB/FANCI/MOB1A/CHRNA9/PARVA/PRKAR1B/IFT122/MCTP2/LMBRD1/PAG1/WSB2/MAPK3/MAP2K2/PRKRIR/HTRA1/SLAMF8/CDC42SE1/PAK6/RGMA/PSMD7/PTGFR/PLEKHG5/TENM2/PTPRE/RFC2/RGR/RGS12/RIT2/RPA3/BGLAP/CCL11/NOD2/STRA6/ARHGAP9/TMEM237/VPS33A/BMP4/SLC8A1/BMPR1B/BOK/STAT2/STK3/BST2/TCEB2/ZEB1/TEAD3/TGM2/TNFAIP3/TNFRSF1A/TRAF5/TRPC6/CCR2/TNFRSF4/WNT10B/ZAP70/CA7/CACNA1E/RAB7A/CARD14/BCL2L14/GPR157/ZC3H12A/FAAP100/CPEB4/C6orf25/EEPD1/CALR/ANTXR1/CMAHP/NR0B2/CASQ1/TRIM63/RAE1/IFITM1/CDK10/RUNX1/TP63/RUNX3/IRS2/CRADD/TNFRSF11A/ALDH1A2/SPHK1/SKAP2/STBD1/TSPAN18/CCRL2/PRC1/STARD13/PIAS2/MAP3K6/RSAD2/AURKB/DAPL1/CD8A/TRIP10/ADIPOQ/RAB36/N4BP1/NUP93/RAPGEF2/CD79A/IQSEC1/FGF19/NR1H4	277
GO:0071310	cellular response to organic substance	110/590	2092/17046	3.83E-06	0.00133	0.00116	CDKN1C/SPON2/GJB6/LECT1/CHI3L1/AP3S1/CCR1/IL31RA/CPS1/CYP11A1/BHLHA15/EIF4G1/ESR1/FGA/FGF10/SBNO2/FOXC2/AKR1B1/MLC1/NUP210/NEDD4L/ARHGEF18/FGF22/NPTN/BMP10/FFAR2/GRB10/HAS1/HLA-DPA1/NR4A1/HRH1/HSP90AB1/HTR3A/IGF2/IL1R1/IL1RN/IL10RA/IL11RA/IL12RB2/IL15RA/INHBA/IRF1/AQP9/JUP/KDR/IPO5/HES5/ARHGDIA/LGALS9/LHCGR/LMNA/LMO2/MOV10/ATP1A2/NRAS/OAS2/P2RY6/IL21R/PARK2/PRKAG3/PML/TLR9/SSH1/PPP1CB/PRKAR1B/LMBRD1/MAPK3/MAP2K2/HTRA1/RGMA/PSMD7/PTGFR/PLEKHG5/PTPRE/RIT2/BGLAP/CCL11/NOD2/BMP4/SLC8A1/BMPR1B/STAT2/BST2/ZEB1/TNFAIP3/TNFRSF1A/CCR2/TNFRSF4/WNT10B/CACNA1E/CARD14/ZC3H12A/CPEB4/CALR/NR0B2/TRIM63/RAE1/IFITM1/RUNX1/IRS2/TNFRSF11A/ALDH1A2/SPHK1/CCRL2/RSAD2/ADIPOQ/NUP93/RAPGEF2/FGF19/NR1H4	110
GO:0007166	cell surface receptor signaling pathway	126/590	2511/17046	6.86E-06	0.00224	0.00194	ABI1/KLRG1/RCAN2/CDKN1C/LECT1/ESM1/AP3S1/CCR1/IL31RA/MIB2/PPM1L/CYLD/DDB1/AGXT/EFNA2/EIF4G1/EPHA1/FCGR2A/FGA/FGF10/FOXL1/FOXC2/NUP210/NEDD4L/ARHGEF18/MAPK8IP2/ADGRF1/FGF22/NPTN/BMP10/FFAR2/GRB10/GRIK4/HLA-DPA1/NR4A1/HSP90AB1/RSPO2/IGF1/IGF2/IL1R1/IL1RN/IL10RA/IL11RA/IL12RB2/IL15RA/INHBA/IRF1/ITGA7/ITGB2/ITGB7/JUP/CD82/KDR/HES5/ARHGDIA/LMNA/MCC/MFNG/MITF/MOV10/NFATC3/NPPC/NRAS/OAS2/P2RY6/IL21R/PARK2/PITX2/PRKAG3/PML/TLR9/PPP1CB/PRKAR1B/IFT122/LMBRD1/PAG1/MAPK3/MAP2K2/HTRA1/RGMA/PSMD7/PLEKHG5/PTPRE/RIT2/CCL11/NOD2/TMEM237/BMP4/BMPR1B/BOK/STAT2/STK3/BST2/ZEB1/TNFAIP3/TNFRSF1A/CCR2/TNFRSF4/WNT10B/ZAP70/RAB7A/CARD14/BCL2L14/GPR157/CPEB4/C6orf25/CMAHP/NR0B2/RAE1/IFITM1/TP63/RUNX3/IRS2/CRADD/TNFRSF11A/SPHK1/TSPAN18/CCRL2/RSAD2/CD8A/ADIPOQ/NUP93/RAPGEF2/CD79A/FGF19/NR1H4	126

GO:0009605	response to external stimulus	120/590	2366/17046	7.30E-06	0.00224	0.00194	SPON2/GJB6/HNRNPUL1/CHGA/CHI3L1/CCR1/CNP/COL9A3/ADM/CPS1/CYP11A1/BHLHA15/DMBT1/EFNA2/A2M/ANKRD23/EPHA1/F11/FGA/FGF10/SBNO2/NFASC/PUM2/SLC37A4/FBXL21/GATM/FGF22/FFAR2/DNAJC15/GUCY1A3/HK1/NR4A1/HRH1/HSP90AB1/IL1R1/IL1RN/IL10RA/AQP2/IL12RB2/IL16/IRF1/ITGB2/JUP/KCNJ8/KDR/STMN1/LDLR/LGALS9/ASGR1/ATP1A2/NRAS/OAS2/PARK2/SIRT6/PDE6B/ATP8A2/PITX2/PKM/PLA2G2A/PRKAG3/PML/PNLIP/TLR9/TREM1/PPP1CB/CHRNA9/PARVA/MAPK3/MAP2K2/PROC/HTRA1/SLAMF8/ARNTL2/RGMA/PSMD7/PTGFR/PLEKHG5/TENM2/CREBZF/ACTA2/RGR/BGLAP/CCL11/NPAS3/NOD2/STRA6/VPS33A/BMP4/SLC8A1/BMPR1B/SLIT1/BPI/STAT2/BST2/TNFAIP3/TNFRSF1A/TRPC6/CCR2/CA7/RAB7A/ZC3H12A/CPEB4/CALR/HOPX/IFITM1/RUNX3/IRS2/CRADD/TNFRSF11A/ALDH1A2/STBD1/CCRL2/RSAD2/DAPL1/CD8A/ADIPOQ/NUP93/RAPGEF2/FGF19/NR1H4	120
GO:0048869	cellular developmental process	172/590	3716/17046	1.25E-05	0.00362	0.00314	ABI1/FARP1/CDKN1C/SPEG/SPON2/TACC2/PDPN/CELF1/LECT1/GPRIN1/CLN5/CCR1/SEZ6/CNP/COL9A3/ADM/IL31RA/CPS1/FAM101A/CSTA/SMYD1/SH3D19/CYLD/ESCO2/CYP11A1/BHLHA15/DMBT1/EEF2/EFNA2/EIF4G1/A2M/ELK4/EPHA1/ESR1/FGA/FGF10/BTBD3/SBNO2/FOXL1/FOXC2/EXPH5/NFASC/EPB41L3/FLOT2/NEDD4L/SYNE1/PSD3/ARHGEF18/MAPK8IP2/TSSK2/DFNB31/GAS2/LCE2B/FGF22/NPTN/BMP10/FFAR2/TMOD4/SOX8/HLA-DOA/ANXA13/NR4A1/HSP90AB1/ID3/RSPO2/IGF1/IGF2/LCE1C/LCE1D/LCE2D/FOXK2/INHBA/IRF1/ITGA7/ITGB2/ITGB7/IVL/JUP/KDR/HES5/LAMA3/STMN1/ARHGDIA/LGALS9/LMNA/MITF/LHX8/MYL2/NEU1/NFATC3/NHLH2/NPPC/NRAS/ATP5B/PARK2/SIRT6/ATP8A2/PITX2/PKHD1/PLA2G2A/PLAGL1/PML/SSH1/MXRA8/FBLIM1/PALMD/HERC6/ELP3/PARVA/IFT122/MAPK3/MAP2K2/MRAP/HTRA1/CDC42SE1/RGMA/PSMD7/TENM2/ERMN/RDH14/ACTA2/BGLAP/CCL11/DNAI2/TMEM237/VPS33A/BMP4/SLC8A1/BMPR1B/SLIT1/BOK/STK3/SUPT6H/ZEB1/TEAD3/TCHH/TNFRSF1A/TRPC6/UCP1/WNT10B/ZAP70/LST1/ZC3H12A/C6orf25/CALR/SLIRP/CAST/CAPZB/SPATA16/ANTXR1/BFSP2/CASQ1/HOPX/TTBK1/IFITM1/SCIN/RUNX1/TP63/RUNX3/IRS2/ACTN1/TNFRSF11A/ALDH1A2/PIAS2/CBFA2T2/RSAD2/DAPL1/CD8A/ADIPOQ/RAPGEF2/CD79A/FGF19	172
GO:0048513	organ development	138/590	2848/17046	1.37E-05	0.00376	0.00326	ABI1/CDKN1C/SPEG/TACC2/PDPN/GJB6/TMED10/LECT1/CHI3L1/CHRNA1/CLN5/CCR1/SEZ6/CNP/COL9A3/COMP/ADM/IL31RA/CPS1/FAM101A/CSTA/SMYD1/CYLD/ESCO2/CYP11A1/EEF2/EFNA2/ESR1/FGA/FGF10/BTBD3/SBNO2/FOXL1/FOXC2/EXPH5/AKR1B1/VGLL2/DFNB31/LCE2B/GATM/BMP10/SOX8/HLA-DOA/ACACB/HOXC4/HSD11B1/HSP90AB1/ID3/RSPO2/IGF1/IGF2/LCE1C/LCE1D/LCE2D/AQP2/AQP5/INHBA/IRF1/ITGA7/IVL/JUP/KCNJ8/KDR/AMIGO3/HES5/STMN1/LGALS9/LHCGR/LMNA/LMO2/MC2R/MITF/LHX8/MYL2/NFATC3/NPPC/NRAS/C11orf73/SIRT6/PDE6B/ATP8A2/PITX2/PKHD1/PKM/PLAGL1/PML/RIPPLY3/HERC6/CHRNA9/PARVA/IFT122/CSGALNACT1/MAPK3/MAP2K2/HTRA1/ACTA2/BGLAP/SCT/CCL11/STRA6/GZF1/VPS33A/BMP4/SLC8A1/BMPR1B/SLIT1/BOK/STK3/ZEB1/TGM2/TCHH/TNFAIP3/WNT10B/ZAP70/C6orf25/CALR/CAST/BFSP2/NR0B2/CASQ1/HOPX/TTBK1/SCIN/RUNX1/TP63/RUNX3/IRS2/ACTN1/TNFRSF11A/ALDH1A2/SPHK1/RSAD2/CD8A/ADIPOQ/MICAL2/RAPGEF2/CD79A/FGF19	138
GO:0034113	heterotypic cell-cell adhesion	9/590	44/17046	1.61E-05	0.0042	0.00364	FGA/NFASC/IL1RN/ITGA7/ITGB2/ITGB7/JUP/PARVA/ADIPOQ	9
GO:0032502	developmental process	231/590	5327/17046	2.10E-05	0.00518	0.00449	ABI1/FARP1/CDKN1C/SPEG/SPON2/TACC2/PDPN/CELF1/GJB6/TMED10/LECT1/ESM1/CHI3L1/CHRNA1/GPRIN1/FAT3/CLN5/CCR1/SEZ6/CNP/COL9A3/COMP/ADM/IL31RA/CPM/CPS1/CRABP1/ZNF358/FAM101A/CSTA/SMYD1/SH3D19/CYLD/ESCO2/CYP11A1/BHLHA15/DIO3/DLG2/DMBT1/EEF2/EFNA2/EIF4G1/A2M/ELK4/EPHA1/ESR1/FGA/FGF10/BTBD3/SBNO2/FOXL1/FOXC2/EXPH5/AKR1B1/NFASC/EPB41L3/DIP2A/FLOT2/NEDD4L/SYNE1/PSD3/ARHGEF18/RYBP/MORC3/MAPK8IP2/TSSK2/VGLL2/DFNB31/GAS2/LCE2B/GATM/FGF22/NPTN/BMP10/FFAR2/TMOD4/SOX8/HLA-DOA/ANXA13/NR4A1/ACACB/HOXC4/HSD11B1/HSP90AB1/TFAP2E/ID3/RSPO2/IGF1/IGF2/LCE1C/LCE1D/LCE2D/IL1RN/AQP2/IL11RA/FOXK2/AQP5/INHBA/IRF1/ITGA7/ITGB2/ITGB7/IVL/JUP/KCNJ8/KDR/AMIGO3/HES5/AFF3/LAMA3/STMN1/ARHGDIA/LGALS9/LHCGR/LMNA/LMO2/MC2R/MFNG/MGAT1/MITF/LHX8/MYL2/DRG1/NEU1/NFATC3/NHLH2/NPPC/NRAS/ATP5B/PARK2/UTP11L/C11orf73/SIRT6/PDE6B/ATP8A2/PITX2/PKHD1/PKM/PLA2G2A/PLAGL1/PML/RIPPLY3/SSH1/MXRA8/FBLIM1/PALMD/BANP/HERC6/ELP3/CHRNA9/PARVA/IFT122/ERMARD/MCTP2/CSGALNACT1/MAPK3/MAP2K2/MRAP/HTRA1/CDC42SE1/RGMA/PSMD7/TENM2/ERMN/RDH14/MARK4/ACTA2/BGLAP/SCT/CCL11/NPAS3/STRA6/GZF1/DNAI2/TMEM237/VPS33A/BMP4/SLC8A1/BMPR1B/SLIT1/BOK/STK3/SUPT6H/BST2/ZEB1/TEAD3/TGM2/TCHH/TNFAIP3/TNFRSF1A/TRPC6/CCR2/UCP1/WNT10B/ZAP70/ZNF7/LST1/ZC3H12A/C6orf25/CALR/SLIRP/CAST/CAPZB/SPATA16/ANTXR1/BFSP2/NR0B2/CASQ1/HOPX/TTBK1/IFITM1/SCIN/RUNX1/TP63/RUNX3/IRS2/ACTN1/TNFRSF11A/ALDH1A2/SPHK1/PIAS2/CBFA2T2/RSAD2/AURKB/DAPL1/CD8A/ADIPOQ/MICAL2/RAPGEF2/CD79A/FGF19	231
GO:0044767	single-organism developmental process	228/590	5251/17046	2.28E-05	0.00518	0.00449	ABI1/FARP1/CDKN1C/SPEG/SPON2/TACC2/PDPN/CELF1/GJB6/TMED10/LECT1/ESM1/CHI3L1/CHRNA1/GPRIN1/FAT3/CLN5/CCR1/SEZ6/CNP/COL9A3/COMP/ADM/IL31RA/CPS1/CRABP1/ZNF358/FAM101A/CSTA/SMYD1/SH3D19/CYLD/ESCO2/CYP11A1/BHLHA15/DIO3/DLG2/DMBT1/EEF2/EFNA2/EIF4G1/A2M/ELK4/EPHA1/ESR1/FGA/FGF10/BTBD3/SBNO2/FOXL1/FOXC2/EXPH5/AKR1B1/NFASC/EPB41L3/DIP2A/FLOT2/NEDD4L/SYNE1/PSD3/ARHGEF18/RYBP/MORC3/MAPK8IP2/TSSK2/VGLL2/DFNB31/GAS2/LCE2B/GATM/FGF22/NPTN/BMP10/FFAR2/TMOD4/SOX8/HLA-DOA/ANXA13/NR4A1/ACACB/HOXC4/HSD11B1/HSP90AB1/ID3/RSPO2/IGF1/IGF2/LCE1C/LCE1D/LCE2D/IL1RN/AQP2/IL11RA/FOXK2/AQP5/INHBA/IRF1/ITGA7/ITGB2/ITGB7/IVL/JUP/KCNJ8/KDR/AMIGO3/HES5/AFF3/LAMA3/STMN1/ARHGDIA/LGALS9/LHCGR/LMNA/LMO2/MC2R/MFNG/MGAT1/MITF/LHX8/MYL2/DRG1/NEU1/NFATC3/NHLH2/NPPC/NRAS/ATP5B/PARK2/UTP11L/C11orf73/SIRT6/PDE6B/ATP8A2/PITX2/PKHD1/PKM/PLA2G2A/PLAGL1/PML/RIPPLY3/SSH1/MXRA8/FBLIM1/PALMD/BANP/HERC6/ELP3/CHRNA9/PARVA/IFT122/ERMARD/MCTP2/CSGALNACT1/MAPK3/MAP2K2/MRAP/HTRA1/CDC42SE1/RGMA/PSMD7/TENM2/ERMN/RDH14/MARK4/ACTA2/BGLAP/SCT/CCL11/STRA6/GZF1/DNAI2/TMEM237/VPS33A/BMP4/SLC8A1/BMPR1B/SLIT1/BOK/STK3/SUPT6H/BST2/ZEB1/TEAD3/TGM2/TCHH/TNFAIP3/TNFRSF1A/TRPC6/CCR2/UCP1/WNT10B/ZAP70/ZNF7/LST1/ZC3H12A/C6orf25/CALR/SLIRP/CAST/CAPZB/SPATA16/ANTXR1/BFSP2/NR0B2/CASQ1/HOPX/TTBK1/IFITM1/SCIN/RUNX1/TP63/RUNX3/IRS2/ACTN1/TNFRSF11A/ALDH1A2/SPHK1/PIAS2/CBFA2T2/RSAD2/AURKB/DAPL1/CD8A/ADIPOQ/MICAL2/RAPGEF2/CD79A/FGF19	228

GO:0070887	cellular response to chemical stimulus	124/590	2524/17046	2.28E-05	0.00518	0.00449	CDKN1C/SPON2/GJB6/LECT1/CHGA/CHI3L1/EGLN2/AP3S1/CCR1/IL31RA/CPS1/CYP11A1/BHLHA15/EIF4G1/ESR1/FGA/FGF10/SBNO2/FOXC2/AKR1B1/MLC1/NUP210/NEDD4L/ARHGEF18/FGF22/NPTN/BMP10/FFAR2/GRB10/HAS1/HLA-DPA1/NR4A1/HRH1/HSP90AB1/HTR3A/IGF2/LCE1D/IL1R1/IL1RN/IL10RA/AQP2/IL11RA/IL12RB2/IL15RA/IL16/INHBA/IRF1/AQP9/ITGB2/JUP/KCNH2/KDR/IPO5/HES5/ARHGDIA/LGALS9/LHCGR/LMNA/LMO2/MOV10/ATP1A2/NFATC3/NRAS/OAS2/P2RY6/IL21R/PARK2/PRKAG3/PML/TLR9/TREM1/SSH1/CYP2W1/PPP1CB/PARVA/PRKAR1B/LMBRD1/MAPK3/MAP2K2/HTRA1/SLAMF8/RGMA/PSMD7/PTGFR/PLEKHG5/PTPRE/RIT2/BGLAP/CCL11/NOD2/BMP4/SLC8A1/BMPR1B/STAT2/BST2/TCEB2/ZEB1/TNFAIP3/TNFRSF1A/TRPC6/CCR2/TNFRSF4/WNT10B/CACNA1E/CARD14/ZC3H12A/CPEB4/CALR/NR0B2/TRIM63/RAE1/IFITM1/RUNX1/IRS2/TNFRSF11A/ALDH1A2/SPHK1/CCRL2/RSAD2/ADIPOQ/NUP93/RAPGEF2/FGF19/NR1H4	124
GO:0009628	response to abiotic stimulus	63/590	1071/17046	2.48E-05	0.0053	0.0046	GJB6/CHI3L1/EGLN2/ADM/CYP11A1/DDB1/ABAT/ANKRD23/AKR1B1/MLC1/NUP210/ACOT11/FBXL21/DNAJC2/HRH1/HSPA1L/HSP90AB1/IGF1/IL1R1/AQP2/IRF1/AQP9/IVL/JUP/KCNJ8/LDLR/LMNA/ATP1A2/NFATC3/NPPC/C11orf73/PDE6B/ATP8A2/PKM/PML/PNLIP/PPP1CB/CHRNA9/MAPK3/RGR/RPA3/BGLAP/SCT/CCL11/STRA6/BMP4/SLC8A1/TCEB2/TNFRSF1A/TRPC6/CACNA1E/CPEB4/CASQ1/TRIM63/RAE1/KMO/TP63/CRADD/TNFRSF11A/AURKB/ADIPOQ/N4BP1/NUP93	63
GO:0048518	positive regulation of biological process	217/590	4960/17046	2.54E-05	0.0053	0.0046	ABI1/TANK/FARP1/CDKN1C/TRDN/SPON2/PITRM1/PDPN/CELF1/RER1/ESM1/CHGA/CHI3L1/ERLIN2/EGLN2/CCR1/SLC51B/SEZ6/MAP3K8/ADM/IL31RA/EGFLAM/CPS1/MIB2/MPP7/SMYD1/SH3D19/CYLD/DDB1/BHLHA15/DIO3/DMBT1/ABAT/EEF2/EIF4G1/A2M/EPHA1/ESR1/F11/SPATA13/FCGR2A/FGA/FGF10/SBNO2/FOXC2/EXPH5/AKR1B1/GGA3/FLOT2/MLC1/TBC1D1/NEDD4L/PSD3/PUM2/ARHGEF18/MAPK8IP2/VGLL2/RASGEF1C/RNF144B/ALS2CL/PABPC1/DNAJC2/FGF22/NPTN/CYTH4/BMP10/GPR26/FFAR2/GRB10/DNAJC15/GUCY1A3/SOX8/HK1/HLA-DPA1/ANXA13/NR4A1/ACACB/AGFG2/HRH1/HSPA1L/HSP90AB1/TFAP2E/ID3/RSPO2/IGF1/IGF2/IL1RN/IL12RB2/IL16/FOXK2/INHBA/IRF1/ITGB2/JUP/KCNH2/KDR/IPO5/AMIGO3/HES5/STMN1/LCP1/LDLR/ARHGDIA/LGALS9/LHCGR/LMNA/LMO2/MC2R/MFNG/MITF/PLEKHG7/NEU1/NFATC3/NHLH2/NPPC/NRAS/OPRL1/P2RY6/PARK2/UTP11L/PRR16/SIRT6/ATP8A2/PITX2/PKHD1/PLA2G2A/PLAGL1/PML/PNLIP/TLR9/RIN2/APBB1IP/BANP/ELP3/GOLPH3L/FANCI/PRKAR1B/PAG1/MAPK3/MAP2K2/MRAP/HTRA1/PAK6/ARNTL2/RGMA/PSMD7/PTGFR/PLEKHG5/TENM2/MARK4/ACTA2/TRIM27/RGS12/SCT/CCL11/NPAS3/NOD2/STRA6/ARHGAP9/BMP4/ZNF649/SLC8A1/BMPR1B/BOK/STK3/SUPT6H/BST2/TCEB2/ZEB1/TEAD3/TERF1/TGM2/TNFAIP3/TNFRSF1A/TRAF5/TRPC6/CCR2/TNFRSF4/WNT10B/ZAP70/CA7/RAB7A/CARD14/BCL2L14/CSPP1/ZC3H12A/CALR/NR0B2/HOPX/IFITM1/SCIN/CDK10/RUNX1/TP63/RUNX3/IRS2/CRADD/TNFRSF11A/ALDH1A2/SPHK1/SKAP2/PRC1/STARD13/PIAS2/MAP3K6/SYT7/CBFA2T2/RSAD2/AURKB/CD8A/ADIPOQ/MICAL2/RAPGEF2/CD79A/IQSEC1/FGF19/NR1H4	217
GO:0022610	biological adhesion	75/590	1348/17046	2.71E-05	0.00545	0.00473	GNE/CDH9/CDH12/SPON2/PDPN/PKP3/FAT3/CCR1/COMP/MAP3K8/EGFLAM/CSTA/CYLD/DDOST/EPHA1/FAT2/FGA/FOXC2/NFASC/FLOT2/NPTN/CYTH4/BMP10/HAS1/HLA-DOA/HLA-DPA1/HSP90AB1/ZC3H12D/IGF1/IGF2/IL1RN/IRF1/ITGA7/ITGB2/ITGB7/JUP/KDR/AMIGO3/HES5/LAMA3/LCP1/ARHGDIA/LGALS9/LPP/NFATC3/ATP5B/PKHD1/PML/APBB1IP/FBLIM1/PPP1CB/PARVA/PAG1/PCDHGB3/SLURP1/TENM2/BGLAP/CCL11/PARVG/NOD2/TINAGL1/PCDH20/BMP4/ZEB1/TGM2/CCR2/TNFRSF4/ZAP70/CALR/ANTXR1/ACTN1/ESAM/RSAD2/CD8A/ADIPOQ	75
GO:0065008	regulation of biological quality	151/590	3225/17046	2.97E-05	0.00575	0.00499	MRVI1/TRDN/PDPN/GJB6/CHGA/EGLN2/CHRNA1/CHRNA2/CLN5/CCR1/SLC51B/SEZ6/CNP/ADM/IL31RA/CPS1/CRABP1/CYP11A1/DDB1/BHLHA15/DIO3/ABAT/A2M/ESR1/F11/FGA/FGF10/FOXC2/AKR1B1/EPB41L3/FLOT2/NEDD4L/SYNE1/ARHGEF18/MORC3/MAPK8IP2/SLC37A4/DFNB31/STEAP2/GAS2/PABPC1/NPTN/AMPD3/GPR26/FFAR2/SCG3/TMOD4/GUCY1A3/SOX8/HK1/ACACB/HRH1/HSP90AB1/HTR3A/NME9/IGF1/IGF2/IL1R1/IL1RN/AQP2/AQP5/INHBA/IRF1/AQP9/ITGA7/ITGB2/JUP/KCNH2/KCNJ8/KCNMB1/KDR/AMIGO3/LDLR/LGALS9/MYL2/NUBP1/ATP1A2/NPPC/NRAS/OPRL1/ATP5B/ANO7/PARK2/PRR16/SIRT6/PDE6B/ATP8A2/PKHD1/PML/TLR9/TREM1/SSH1/APBB1IP/FBLIM1/PALMD/SLC30A10/CHRNA9/PARVA/PRKAR1B/MAPK3/PROC/SLAMF8/CDC42SE1/ERMN/RDH14/ACTA2/RFC2/TRIM27/RPA3/BGLAP/SCT/CCL11/ABHD4/NOD2/STRA6/BMP4/SLC4A1/SLC6A12/SLC8A1/SLC9A3/SLIT1/STK3/TERF1/TGM2/TNFAIP3/TRPC6/CCR2/WNT10B/CA7/CACNA1E/RAB7A/LST1/ZC3H12A/C6orf25/CALR/CAPZB/ATP13A4/NR0B2/CASQ1/TTBK1/SCIN/TP63/IRS2/ACTN1/TNFRSF11A/ALDH1A2/SYT7/ESAM/SLC16A3/ADIPOQ/RAPGEF2	151
GO:0061448	connective tissue development	21/590	224/17046	3.54E-05	0.0066	0.00573	LECT1/CHI3L1/COMP/FAM101A/FOXC2/BMP10/SOX8/HOXC4/RSPO2/IGF1/HES5/NPPC/CSGALNACT1/MAPK3/ACTA2/BMP4/BMPR1B/ZEB1/WNT10B/SCIN/RUNX3	21
GO:0009653	anatomical structure morphogenesis	125/590	2579/17046	3.93E-05	0.00707	0.00613	ABI1/FARP1/CDKN1C/SPON2/PDPN/GJB6/LECT1/ESM1/CHI3L1/CNP/COL9A3/COMP/ADM/CPM/ZNF358/FAM101A/SH3D19/CYLD/EFNA2/EPHA1/ESR1/FGA/FGF10/BTBD3/SBNO2/FOXL1/FOXC2/NFASC/EPB41L3/NEDD4L/ARHGEF18/MAPK8IP2/GAS2/FGF22/BMP10/TMOD4/SOX8/NR4A1/HOXC4/HSP90AB1/ID3/RSPO2/IGF1/IGF2/IL1RN/FOXK2/AQP5/INHBA/ITGA7/ITGB2/ITGB7/KDR/HES5/AFF3/LAMA3/STMN1/ARHGDIA/LHX8/MYL2/NFATC3/NPPC/NRAS/ATP5B/PARK2/SIRT6/ATP8A2/PITX2/PKHD1/PML/SSH1/FBLIM1/PALMD/CHRNA9/PARVA/IFT122/CSGALNACT1/MAPK3/MAP2K2/HTRA1/CDC42SE1/RGMA/PSMD7/TENM2/ERMN/ACTA2/BGLAP/CCL11/STRA6/GZF1/DNAI2/TMEM237/VPS33A/BMP4/SLC8A1/BMPR1B/SLIT1/STK3/ZEB1/TGM2/TNFAIP3/TRPC6/CCR2/WNT10B/LST1/ZC3H12A/C6orf25/CALR/SLIRP/CAST/CAPZB/ANTXR1/CASQ1/HOPX/TTBK1/RUNX1/TP63/RUNX3/IRS2/ACTN1/ALDH1A2/SPHK1/ADIPOQ/MICAL2/RAPGEF2/FGF19	125
GO:0007155	cell adhesion	74/590	1343/17046	4.25E-05	0.00728	0.00632	GNE/CDH9/CDH12/SPON2/PDPN/PKP3/FAT3/CCR1/COMP/MAP3K8/EGFLAM/CSTA/CYLD/DDOST/EPHA1/FAT2/FGA/FOXC2/NFASC/FLOT2/NPTN/CYTH4/BMP10/HAS1/HLA-DOA/HLA-DPA1/ZC3H12D/IGF1/IGF2/IL1RN/IRF1/ITGA7/ITGB2/ITGB7/JUP/KDR/AMIGO3/HES5/LAMA3/LCP1/ARHGDIA/LGALS9/LPP/NFATC3/ATP5B/PKHD1/PML/APBB1IP/FBLIM1/PPP1CB/PARVA/PAG1/PCDHGB3/SLURP1/TENM2/BGLAP/CCL11/PARVG/NOD2/TINAGL1/PCDH20/BMP4/ZEB1/TGM2/CCR2/TNFRSF4/ZAP70/CALR/ANTXR1/ACTN1/ESAM/RSAD2/CD8A/ADIPOQ	74

GO:0019932	second-messenger-mediated signaling	20/590	210/17046	4.33E-05	0.00728	0.00632	RCAN2/MRVI1/ADM/BHLHA15/GUCY1A3/HRH1/IGF1/KDR/LHCGR/ATP1A2/NFATC3/MCTP2/PTGFR/TENM2/SLC8A1/ZAP70/CASQ1/SPHK1/CD8A/RAPGEF2	20
GO:0044707	single-multicellular organism process	261/590	6214/17046	4.62E-05	0.00754	0.00654	ABI1/FARP1/RCAN2/CDKN1C/SPEG/MRVI1/TRDN/SPON2/TACC2/PDPN/CELF1/GJB6/TMED10/LECT1/ESM1/CHGA/CHI3L1/CHRNA1/CHRNA2/GPRIN1/FAT3/CLN5/CCR1/SEZ6/CNP/COL9A3/COMP/ADM/IL31RA/OR2A14/CPS1/CRABP1/ZNF358/CRYBB3/FAM101A/CSTA/SMYD1/CYLD/ESCO2/CYP11A1/DIO3/DLG2/DMBT1/ABAT/DTNA/EEF2/EFNA2/EIF4G1/A2M/EPHA1/ESR1/F11/FAT2/FGA/FGF10/BTBD3/SBNO2/FOXL1/FOXC2/EXPH5/AKR1B1/NFASC/EPB41L3/DIP2A/NEDD4L/PSD3/RYBP/MORC3/MAPK8IP2/TSSK2/VGLL2/DFNB31/PNKD/LCE2B/GATM/FGF22/NPTN/AMPD3/BMP10/FFAR2/SCG3/TMOD4/GUCY1A3/SOX8/HLA-DOA/HLA-DPA1/NR4A1/ACACB/HOXC4/HRH1/HSD11B1/HSP90AB1/HTR3A/ID3/RSPO2/IGF1/IGF2/LCE1C/LCE1D/LCE2D/IL1RN/AQP2/IL12RB2/AQP5/INHBA/IRF1/AQP9/ITGA7/ITGB2/IVL/JUP/KCNH2/KCNJ8/KCNMB1/KDR/AMIGO3/HES5/AFF3/LAMA3/STMN1/OR2A5/LDLR/ARHGDIA/LGALS9/LHCGR/LMNA/LMO2/MC2R/MCC/MFNG/MGAT1/MITF/LHX8/MYH4/MYL2/DRG1/NEU1/ATP1A2/NFATC3/NHLH2/NPPC/NRAS/OPRL1/OR2C1/OR3A2/SLC22A18/ATP5B/PARK2/UTP11L/C11orf73/SIRT6/PDE6B/ATP8A2/PITX2/PKHD1/PKM/PLA2G2A/PLAGL1/PML/RIPPLY3/PNLIP/TLR9/TREM1/SSH1/APBB1IP/MXRA8/BANP/PPP1CB/HERC6/ELP3/CHRNA9/PARVA/PRKAR1B/IFT122/ERMARD/MCTP2/CSGALNACT1/MAPK3/MAP2K2/PROC/HTRA1/RGMA/PSMD7/SLURP1/PLEKHG5/TENM2/ERMN/RDH14/MARK4/ACTA2/TRIM27/RGR/BGLAP/SCT/CCL11/NPAS3/NOD2/STRA6/GZF1/DNAI2/VPS33A/BMP4/SLC8A1/BMPR1B/SLIT1/BOK/BPI/STK3/SUPT6H/BST2/ZEB1/TEAD3/TGM2/TCHH/TNFAIP3/TNFRSF1A/TRPC6/CCR2/TNFRSF4/WNT10B/ZAP70/ZNF7/CACNA1E/RAB7A/LST1/CALD1/ZC3H12A/C6orf25/CALR/CAST/CAPZB/SPATA16/BFSP2/NR0B2/CASQ1/HOPX/TTBK1/TRIM63/IFITM1/SCIN/RUNX1/TP63/RUNX3/IRS2/ACTN1/TNFRSF11A/ALDH1A2/SPHK1/STBD1/PIAS2/ESAM/SLC16A3/CBFA2T2/RSAD2/AURKB/CD8A/ADIPOQ/MICAL2/RAPGEF2/CD79A/FGF19	261
GO:0048583	regulation of response to stimulus	152/590	3283/17046	4.99E-05	0.00789	0.00685	ABI1/TANK/FARP1/KLRG1/RCAN2/CDKN1C/SPON2/LECT1/ESM1/CHI3L1/CCR1/SEZ6/TNFAIP8L1/MAP3K8/ADM/IL31RA/MIB2/MPP7/CYLD/ZNF366/DMBT1/A2M/ESR1/F11/SPATA13/FCGR2A/FGA/FGF10/SBNO2/FOXL1/FOXC2/AKR1B1/MLC1/RHOBTB2/NUP210/NEDD4L/PSD3/PUM2/ARHGEF18/MAPK8IP2/SLC37A4/ALS2CL/DNAJC2/FGF22/NPTN/CYTH4/BMP10/FFAR2/GRB10/HK1/HLA-DPA1/NR4A1/HSPA1L/HSP90AB1/RSPO2/IGF1/IGF2/IL1R1/IL1RN/IL16/INHBA/IRF1/ITGB2/ITGB7/JUP/KDR/HES5/ARHGDIA/LGALS9/LHCGR/LMNA/MCC/MFNG/MOV10/PLEKHG7/NFATC3/NRAS/PARK2/C11orf73/SIRT6/PDE6B/PKHD1/PLA2G2A/PML/TLR9/TREM1/PPP1CB/IFT122/LMBRD1/PAG1/MAPK3/MAP2K2/PROC/HTRA1/PAK6/PSMD7/PLEKHG5/PTPRE/TRIM27/RGS12/RPA3/CCL11/NOD2/STRA6/ARHGAP9/TMEM237/BMP4/BMPR1B/STAT2/STK3/SUPT6H/BST2/ZEB1/TNFAIP3/TNFRSF1A/TRAF5/CCR2/WNT10B/ZAP70/CA7/CACNA1E/RAB7A/CARD14/BCL2L14/CALR/CMAHP/CASQ1/HOPX/TRIM63/RAE1/IFITM1/CDK10/RUNX1/TP63/RUNX3/IRS2/CRADD/TNFRSF11A/SPHK1/SKAP2/STARD13/PIAS2/MAP3K6/RSAD2/CD8A/TRIP10/ADIPOQ/NUP93/RAPGEF2/CD79A/IQSEC1/FGF19	152
GO:0008360	regulation of cell shape	15/590	132/17046	5.26E-05	0.00808	0.00701	PDPN/EPB41L3/ARHGEF18/GAS2/ITGA7/ITGB2/KDR/FBLIM1/PALMD/PARVA/CDC42SE1/ERMN/CCL11/LST1/TTBK1	15
GO:0018149	peptide cross-linking	9/590	51/17046	5.56E-05	0.00814	0.00706	EGFLAM/CSTA/LCE2B/LCE1C/LCE1D/LCE2D/IVL/SPOCK3/TGM2	9
GO:0032501	multicellular organismal process	268/590	6425/17046	5.62E-05	0.00814	0.00706	ABI1/FARP1/RCAN2/CDKN1C/SPEG/MRVI1/TRDN/SPON2/TACC2/PDPN/CELF1/GJB6/TMED10/LECT1/ESM1/ADAM29/CHGA/CHI3L1/CHRNA1/CHRNA2/GPRIN1/FAT3/CLN5/CCR1/SEZ6/CNP/COL9A3/COMP/ADM/IL31RA/OR2A14/CPS1/CRABP1/ZNF358/CRYBB3/FAM101A/CSTA/SMYD1/CYLD/ESCO2/CYP11A1/DIO3/DLG2/DMBT1/ABAT/DTNA/EEF2/EFNA2/EIF4G1/A2M/EPHA1/ESR1/F11/FAT2/FGA/FGF10/BTBD3/SBNO2/FOXL1/FOXC2/EXPH5/AKR1B1/NFASC/EPB41L3/DIP2A/NEDD4L/PSD3/RYBP/MORC3/MAPK8IP2/TSSK2/VGLL2/DFNB31/PNKD/LCE2B/GATM/FGF22/NPTN/AMPD3/BMP10/FFAR2/SCG3/TMOD4/GUCY1A3/HAS1/SOX8/HLA-DOA/HLA-DPA1/NR4A1/ACACB/HOXC4/HRH1/HSD11B1/HSP90AB1/HTR3A/ID3/RSPO2/IGF1/IGF2/LCE1C/LCE1D/LCE2D/IL1R1/IL1RN/AQP2/IL12RB2/AQP5/INHBA/IRF1/AQP9/ITGA7/ITGB2/IVL/JUP/KCNH2/KCNJ8/KCNMB1/KDR/AMIGO3/HES5/AFF3/LAMA3/STMN1/OR2A5/LDLR/ARHGDIA/LGALS9/LHCGR/LMNA/LMO2/MC2R/MCC/MFNG/MGAT1/MITF/LHX8/MYH4/MYL2/DRG1/NEU1/ATP1A2/NFATC3/NHLH2/NPPC/NRAS/OPRL1/OR2C1/OR3A2/SLC22A18/ATP5B/PARK2/UTP11L/C11orf73/SIRT6/PDE6B/ATP8A2/PITX2/PKHD1/PKM/PLA2G2A/PLAGL1/PML/RIPPLY3/PNLIP/TLR9/TREM1/SSH1/APBB1IP/MXRA8/BANP/PPP1CB/HERC6/ELP3/CHRNA9/PARVA/PRKAR1B/IFT122/ERMARD/MCTP2/CSGALNACT1/MAPK3/MAP2K2/PROC/HTRA1/RGMA/PSMD7/SLURP1/PTGFR/PLEKHG5/TENM2/ERMN/RDH14/MARK4/ACTA2/TRIM27/RGR/BGLAP/SCT/CCL11/NPAS3/NOD2/STRA6/GZF1/DNAI2/VPS33A/BMP4/SLC8A1/BMPR1B/SLIT1/BOK/BPI/STK3/SUPT6H/BST2/ZEB1/TEAD3/TGM2/TCHH/TNFAIP3/TNFRSF1A/TRPC6/CCR2/TNFRSF4/WNT10B/ZAP70/ZNF7/CACNA1E/RAB7A/LST1/CALD1/ZC3H12A/C6orf25/CALR/SLIRP/CAST/CAPZB/SPATA16/BFSP2/NR0B2/CASQ1/HOPX/TTBK1/TRIM63/IFITM1/SCIN/RUNX1/TP63/RUNX3/IRS2/ACTN1/TNFRSF11A/ALDH1A2/SPHK1/CCNA1/ENDOU/STBD1/PIAS2/ESAM/SLC16A3/CBFA2T2/RSAD2/AURKB/CD8A/ADIPOQ/MICAL2/RAPGEF2/CD79A/FGF19	268
GO:0006928	movement of cell or subcellular component	92/590	1789/17046	6.39E-05	0.00901	0.00782	ABI1/SPON2/PDPN/CHGA/KIF12/AP3S1/CCR1/COL9A3/KLC3/DNAH6/EFNA2/EPHA1/FAT2/SPATA13/PHACTR1/FGF10/FOXC2/NFASC/FGF22/BMP10/FFAR2/HAS1/SOX8/NR4A1/HRH1/HSP90AB1/IGF1/IL1RN/IL16/ITGA7/ITGB2/ITGB7/JUP/KDR/KIF25/LAMA3/STMN1/LCP1/ARHGDIA/LGALS9/LMNA/MCC/MYH4/MYL2/ATP1A2/NRAS/P2RY6/ATP5B/PITX2/PKHD1/PML/TREM1/ELP3/PARVA/IFT122/MAPK3/MAP2K2/PROC/PAK6/RGMA/PSMD7/PLEKHG5/TENM2/FAM60A/CCL11/NOD2/DNAI2/BMP4/SLC8A1/BMPR1B/SLIT1/BST2/TRPC6/CCR2/ZAP70/CACNA1E/CALD1/CALR/SLIRP/CAPZB/DYNLRB2/IFITM1/RUNX3/IRS2/ACTN1/TNFRSF11A/SPHK1/ESAM/SLC16A3/ADIPOQ/RAPGEF2/FGF19	92
GO:0042127	regulation of cell proliferation	75/590	1385/17046	6.62E-05	0.00909	0.00789	ABI1/CDKN1C/SPEG/GJB6/LECT1/ESM1/ADM/IL31RA/EPHA1/ESR1/FGF10/AKR1B1/MORC3/BMP10/SOX8/HLA-DPA1/NR4A1/TFAP2E/ZC3H12D/IGF1/IGF2/IL12RB2/INHBA/IRF1/JUP/KDR/HES5/LGALS9/MCC/MITF/NEU1/NPPC/NRAS/SIRT6/ATP8A2/PITX2/PKHD1/PLA2G2A/PML/RIPPLY3/IFT122/PRKRIR/HTRA1/SLURP1/PTGFR/RPA3/CCL11/NOD2/BMP4/BMPR1B/STK3/ZEB1/TNFAIP3/TRAF5/CCR2/TNFRSF4/WNT10B/ZAP70/LST1/CALR/IFITM1/SCIN/CDK10/RUNX1/TP63/RUNX3/IRS2/TNFRSF11A/ALDH1A2/SPHK1/SKAP2/PRC1/ADIPOQ/RAPGEF2/FGF19	75

GO:0009719	response to endogenous stimulus	83/590	1578/17046	7.17E-05	0.00959	0.00832	CDKN1C/TMED10/CHRNA1/CHRNA2/AP3S1/ADM/CPS1/CYP11A1/ZNF366/ABAT/AGXT/EIF4G1/ESR1/FGA/FGF10/FOXC2/AKR1B1/NEDD4L/ARHGEF18/STEAP2/GATM/FGF22/NPTN/BMP10/GRB10/HAS1/NR4A1/HRH1/HTR3A/IGF2/IL1R1/IL1RN/INHBA/AQP9/JUP/IPO5/HES5/LHCGR/LMO2/MOV10/ATP1A2/NRAS/OPRL1/P2RY6/PARK2/PITX2/PKM/PRKAG3/PML/TLR9/SSH1/PPP1CB/CHRNA9/PRKAR1B/LMBRD1/MAPK3/MAP2K2/HTRA1/RGMA/PSMD7/PTGFR/PTPRE/BGLAP/NOD2/BMP4/SLC8A1/SLC9A3/BMPR1B/ZEB1/TNFAIP3/WNT10B/CPEB4/CALR/NR0B2/TRIM63/RUNX1/RUNX3/IRS2/ALDH1A2/ADIPOQ/RAPGEF2/FGF19/NR1H4	83
GO:0035556	intracellular signal transduction	119/590	2478/17046	9.45E-05	0.01233	0.0107	ABI1/TANK/FARP1/RCAN2/MRVI1/CHI3L1/CCR1/SEZ6/TNFAIP8L1/MAP3K8/ADM/IL31RA/MIB2/SGOL1/PPM1L/CYLD/BHLHA15/A2M/ESR1/SPATA13/FGA/FGF10/FHIT/AKR1B1/RHOBTB2/PSD3/PPP1R13B/PUM2/ARHGEF18/MAPK8IP2/TSSK2/RASGEF1C/ALS2CL/ACOT11/PLEK2/FGF22/CYTH4/BMP10/GUCY1A3/NR4A1/HRH1/IGF1/IGF2/IL1RN/INHBA/KCNH2/KDR/HES5/STMN1/LCP1/ARHGDIA/LGALS9/LHCGR/RAB19/MOV10/PLEKHG7/ATP1A2/NFATC3/NRAS/PARK2/PKHD1/PLA2G2A/PRKAG3/PML/TLR9/TREM1/RIN2/PPP1CB/MOB1A/IFT122/MCTP2/LMBRD1/PAG1/WSB2/MAPK3/MAP2K2/PAK6/PSMD7/PTGFR/PLEKHG5/TENM2/RGS12/RIT2/CCL11/NOD2/ARHGAP9/BMP4/SLC8A1/BOK/STAT2/STK3/BST2/TEAD3/TNFAIP3/TNFRSF1A/TRAF5/CCR2/ZAP70/RAB7A/CARD14/CASQ1/IFITM1/CDK10/TP63/IRS2/CRADD/TNFRSF11A/SPHK1/PRC1/STARD13/MAP3K6/AURKB/CD8A/TRIP10/ADIPOQ/RAB36/RAPGEF2/IQSEC1/FGF19	119
GO:0051179	localization	221/590	5173/17046	0.0001	0.013	0.01128	ABI1/TRDN/SPON2/COG5/TACC2/PDPN/TMED10/RER1/CHGA/CHI3L1/PKP3/EXOC3/CHRNA1/CHRNA2/PANX3/RBP7/AP3S1/CCR1/SLC51B/CNP/ADM/CRABP1/PARP4/MPP7/LDLRAD3/KLC3/ABCC13/SH3D19/CYLD/ESCO2/DDOST/BHLHA15/DLG2/DMBT1/DNAH6/ABAT/AGXT/A2M/SLC10A4/EPHA1/FAT2/SPATA13/FCGR2A/PHACTR1/FGA/FGF10/TRAK1/FOXC2/EXPH5/NFASC/EPB41L3/GGA3/FLOT2/MLC1/TBC1D1/NUP210/NEDD4L/SYNE1/MORC3/MAPK8IP2/SLC37A4/SAMM50/SEC31B/STEAP2/SLC17A5/BMP10/GPR26/FFAR2/GRB10/GRIK4/DNAJC15/SCG3/HAS1/SOX8/HK1/ANXA13/NR4A1/ACACB/HRH1/HSPA1L/HSP90AB1/HTR3A/IGF1/IGF2/IL1RN/AQP2/IL16/AQP5/INHBA/AQP9/ITGA7/ITGB2/ITGB7/JUP/KCNH2/KCNJ8/KCNJ9/KCNMB1/KDR/IPO5/LAMA3/STMN1/LCP1/LDLR/LGALS9/LMNA/RAB19/MCC/ASGR1/NUBP1/ATP1A2/NFATC3/NRAS/OPRL1/SLC22A18/P2RY6/ATP5B/ANO7/PARK2/PCYOX1/C11orf73/SIRT6/ATP8A2/PITX2/PKHD1/LRP1B/PRKAG3/PML/FXYD6/SLCO1C1/PNLIP/TLR9/TREM1/RIN2/BANP/ELP3/DNAJC17/GOLPH3L/SLC47A1/SLC29A3/TRPV6/SLC30A10/CHRNA9/PEX26/PARVA/PRKAR1B/IFT122/LMBRD1/APOBR/MAPK3/MAP2K2/PROC/MRAP/TRPV5/CDC42SE1/PAK6/PLEKHG5/FAM60A/TRIM27/SCT/CCL11/ABHD4/NOD2/TINAGL1/STRA6/VPS33A/BMP4/SLC4A1/ZG16/SLC6A12/SLC8A1/SLC9A3/SLC20A2/SLIT1/SRP68/SUPT6H/BST2/TGM2/TNFAIP3/TNFRSF1A/TRPC6/TRPM2/CCR2/TNFRSF4/UCP1/ZAP70/CA7/CACNA1E/RAB7A/ZC3H12A/CALR/SLIRP/DYNLRB2/SLC25A18/MFSD7/ATP13A4/NR0B2/MON1A/CASQ1/RAE1/SLC43A1/IFITM1/SCIN/SERPINA6/IRS2/ACTN1/TNFRSF11A/SPHK1/ENDOU/SYT7/ESAM/SLC16A3/RSAD2/AURKB/TRIP10/ADIPOQ/RAB36/NUP93/RAPGEF2/FGF19/NR1H4	221
GO:0019722	calcium-mediated signaling	14/590	125/17046	0.00011	0.01323	0.01148	RCAN2/BHLHA15/IGF1/KDR/ATP1A2/NFATC3/MCTP2/PTGFR/TENM2/SLC8A1/ZAP70/CASQ1/SPHK1/CD8A	14
GO:0007275	multicellular organismal development	197/590	4527/17046	0.00011	0.01323	0.01148	ABI1/FARP1/CDKN1C/SPEG/SPON2/TACC2/PDPN/CELF1/GJB6/TMED10/LECT1/ESM1/CHI3L1/CHRNA1/GPRIN1/FAT3/CLN5/CCR1/SEZ6/CNP/COL9A3/COMP/ADM/IL31RA/CPS1/CRABP1/ZNF358/FAM101A/CSTA/SMYD1/CYLD/ESCO2/CYP11A1/DLG2/DMBT1/EEF2/EFNA2/EIF4G1/EPHA1/ESR1/FGA/FGF10/BTBD3/SBNO2/FOXL1/FOXC2/EXPH5/AKR1B1/NFASC/EPB41L3/DIP2A/NEDD4L/PSD3/RYBP/MORC3/MAPK8IP2/TSSK2/VGLL2/DFNB31/LCE2B/GATM/FGF22/NPTN/BMP10/SOX8/HLA-DOA/NR4A1/ACACB/HOXC4/HSD11B1/HSP90AB1/ID3/RSPO2/IGF1/IGF2/LCE1C/LCE1D/LCE2D/IL1RN/AQP2/AQP5/INHBA/IRF1/ITGA7/ITGB2/IVL/JUP/KCNJ8/KDR/AMIGO3/HES5/AFF3/LAMA3/STMN1/ARHGDIA/LGALS9/LHCGR/LMNA/LMO2/MC2R/MFNG/MGAT1/MITF/LHX8/MYL2/DRG1/NEU1/NFATC3/NHLH2/NPPC/NRAS/ATP5B/PARK2/UTP11L/C11orf73/SIRT6/PDE6B/ATP8A2/PITX2/PKHD1/PKM/PLAGL1/PML/RIPPLY3/SSH1/MXRA8/BANP/HERC6/ELP3/CHRNA9/PARVA/IFT122/ERMARD/MCTP2/CSGALNACT1/MAPK3/MAP2K2/HTRA1/RGMA/PSMD7/TENM2/MARK4/ACTA2/BGLAP/SCT/CCL11/STRA6/GZF1/DNAI2/VPS33A/BMP4/SLC8A1/BMPR1B/SLIT1/BOK/STK3/SUPT6H/BST2/ZEB1/TGM2/TCHH/TNFAIP3/TNFRSF1A/TRPC6/CCR2/WNT10B/ZAP70/ZNF7/LST1/ZC3H12A/C6orf25/CALR/CAST/SPATA16/BFSP2/NR0B2/CASQ1/HOPX/TTBK1/SCIN/RUNX1/TP63/RUNX3/IRS2/ACTN1/TNFRSF11A/ALDH1A2/SPHK1/CBFA2T2/RSAD2/AURKB/CD8A/ADIPOQ/MICAL2/RAPGEF2/CD79A/FGF19	197
GO:0030154	cell differentiation	157/590	3469/17046	0.00012	0.01383	0.012	ABI1/FARP1/CDKN1C/SPEG/SPON2/TACC2/CELF1/LECT1/GPRIN1/CLN5/CCR1/SEZ6/CNP/COL9A3/ADM/IL31RA/CPS1/FAM101A/CSTA/SMYD1/CYLD/ESCO2/CYP11A1/BHLHA15/DMBT1/EEF2/EFNA2/EIF4G1/A2M/ELK4/EPHA1/ESR1/FGA/FGF10/BTBD3/SBNO2/FOXL1/FOXC2/EXPH5/NFASC/EPB41L3/FLOT2/NEDD4L/SYNE1/PSD3/MAPK8IP2/TSSK2/DFNB31/LCE2B/FGF22/NPTN/BMP10/FFAR2/TMOD4/SOX8/HLA-DOA/ANXA13/NR4A1/HSP90AB1/ID3/RSPO2/IGF1/IGF2/LCE1C/LCE1D/LCE2D/FOXK2/INHBA/IRF1/ITGA7/ITGB2/ITGB7/IVL/JUP/KDR/HES5/LAMA3/STMN1/ARHGDIA/LGALS9/LMNA/MITF/LHX8/MYL2/NEU1/NFATC3/NHLH2/NPPC/NRAS/ATP5B/PARK2/SIRT6/ATP8A2/PITX2/PLA2G2A/PLAGL1/PML/SSH1/MXRA8/HERC6/ELP3/PARVA/MAPK3/MAP2K2/MRAP/HTRA1/RGMA/PSMD7/TENM2/RDH14/ACTA2/BGLAP/VPS33A/BMP4/SLC8A1/BMPR1B/SLIT1/BOK/STK3/SUPT6H/ZEB1/TEAD3/TCHH/TNFRSF1A/TRPC6/UCP1/WNT10B/ZAP70/LST1/ZC3H12A/C6orf25/CALR/SLIRP/CAST/SPATA16/ANTXR1/BFSP2/CASQ1/HOPX/IFITM1/SCIN/RUNX1/TP63/RUNX3/IRS2/ACTN1/TNFRSF11A/ALDH1A2/PIAS2/CBFA2T2/RSAD2/DAPL1/CD8A/ADIPOQ/RAPGEF2/CD79A/FGF19	157
GO:0002376	immune system process	115/590	2392/17046	0.00012	0.01414	0.01226	ABI1/TANK/CD300LD/KLRG1/CDKN1C/SPON2/CHGA/CCR1/MAP3K8/ADM/IL31RA/CYLD/ESCO2/DDOST/DMBT1/EEF2/EFNA2/A2M/FCGR2A/FGA/FGF10/SBNO2/FOXL1/FLOT2/PUM2/SLC37A4/FGF22/AMPD3/FFAR2/HLA-DOA/HLA-DPA1/NR4A1/HRH1/HSP90AB1/ZC3H12D/CD300E/IGF1/IGF2/IL1R1/IL1RN/IL16/INHBA/IRF1/AQP9/ITGB2/ITGB7/KCNJ8/KDR/HES5/LCP1/LGALS9/LMO2/MITF/MOV10/NFATC3/NRAS/OAS2/IL21R/PITX2/PML/TLR9/TREM1/POU2AF1/APBB1IP/HERC6/PRKAR1B/PAG1/MAPK3/MAP2K2/PROC/HTRA1/PSMD7/PTPRE/TRIM27/BGLAP/CCL11/NOD2/TINAGL1/VPS33A/BMP4/BPI/STAT2/STK3/SUPT6H/BST2/ZEB1/TNFAIP3/CCR2/TNFRSF4/WNT10B/ZAP70/CA7/RAB7A/LST1/ZC3H12A/C6orf25/CALR/IFITM1/SCIN/RUNX1/RUNX3/IRS2/ACTN1/TNFRSF11A/ENDOU/SKAP2/ESAM/SLC16A3/RSAD2/CD8A/ADIPOQ/NUP93/RAPGEF2/CD79A/FGF19	115
GO:0008283	cell proliferation	90/590	1775/17046	0.00013	0.01455	0.01262	ABI1/CDKN1C/SPEG/TACC2/PDPN/GJB6/LECT1/ESM1/PDAP1/ADM/IL31RA/DMBT1/DPH1/EPHA1/ESR1/FGF10/FOXC2/AKR1B1/MORC3/BMP10/SOX8/HLA-DPA1/NR4A1/TFAP2E/ZC3H12D/IGF1/IGF2/IL12RB2/IL15RA/INHBA/IRF1/ITGB2/JUP/KDR/HES5/LGALS9/MCC/MITF/NEU1/NPPC/NRAS/SIRT6/ATP8A2/PITX2/PKHD1/PLA2G2A/PML/RIPPLY3/IFT122/CSGALNACT1/MAP2K2/PRKRIR/HTRA1/SLURP1/PTGFR/TRIM27/RPA3/CCL11/NOD2/BMP4/BMPR1B/BOK/STK3/BST2/ZEB1/TNFAIP3/TRAF5/CCR2/TNFRSF4/WNT10B/ZAP70/LST1/CALR/RETNLB/IFITM1/SCIN/CDK10/RUNX1/TP63/RUNX3/IRS2/TNFRSF11A/ALDH1A2/SPHK1/SKAP2/PRC1/ADIPOQ/RAPGEF2/CD79A/FGF19	90

GO:0009888	tissue development	86/590	1684/17046	0.00015	0.01612	0.01399	ABI1/SPEG/LECT1/CHI3L1/CHRNA1/CCR1/COMP/ADM/CPS1/ZNF358/FAM101A/CSTA/SMYD1/CYP11A1/DMBT1/ESR1/FGF10/SBNO2/FOXL1/FOXC2/EXPH5/FLOT2/VGLL2/LCE2B/GATM/BMP10/SOX8/HOXC4/ID3/RSPO2/IGF1/LCE1C/LCE1D/LCE2D/INHBA/ITGA7/ITGB2/IVL/KDR/HES5/LAMA3/LMNA/MITF/MYL2/NFATC3/NPPC/NRAS/SIRT6/PDE6B/ATP8A2/PITX2/PKM/PLAGL1/PML/IFT122/CSGALNACT1/MAPK3/MAP2K2/ACTA2/BGLAP/CCL11/STRA6/GZF1/BMP4/SLC8A1/BMPR1B/STK3/ZEB1/TGM2/TCHH/TNFRSF1A/WNT10B/CALR/BFSP2/CASQ1/HOPX/SCIN/RUNX1/TP63/RUNX3/ALDH1A2/CBFA2T2/ADIPOQ/MICAL2/RAPGEF2/FGF19	86
GO:0044723	single-organism carbohydrate metabolic process	42/590	669/17046	0.00015	0.0162	0.01406	GNE/GALNT15/CLN5/SLC51B/NEU4/CPS1/PARP4/B3GLCT/MGAT5B/DDOST/ENO2/TRAK1/AKR1B1/SLC37A4/ST6GALNAC3/GBGT1/SLC17A5/DHDH/EOGT/GRB10/HAS1/HK1/HRH1/IGF1/IGF2/MUC21/LHCGR/MGAT1/NEU1/OAS2/PARK2/CHST15/SIRT6/PKM/PRKAG3/PPP1CB/CSGALNACT1/MOGS/CALR/IRS2/STBD1/ADIPOQ	42
GO:0048519	negative regulation of biological process	182/590	4153/17046	0.00016	0.01691	0.01467	ABI1/FARP1/CDKN1C/SPEG/MRVI1/TRDN/CELF1/GJB6/LECT1/CHGA/ERLIN2/CARD16/CCR1/SEZ6/TNFAIP8L1/COMP/ADM/IL31RA/HUS1B/FAM101A/CSTA/SMYD1/CYLD/DDB1/ZNF366/BHLHA15/DLG2/A2M/ELK4/EPHA1/ESR1/F11/PHACTR1/FGA/FGF10/FHIT/SBNO2/FOXC2/DIP2A/FLOT2/NEDD4L/PPP1R13B/PUM2/RYBP/MORC3/GAS2/SACS/PABPC1/DNAJC2/BMP10/GRB10/DNAJC15/TMOD4/GUCY1A3/GPR132/HAS1/SOX8/HLA-DOA/ANXA13/NR4A1/ACACB/HSP90AB1/ID3/ZC3H12D/RSPO2/IGF1/IL1RN/INHBA/IRF1/ITIH4/KCNH2/KDR/KIF25/IPO5/HES5/STMN1/ARHGDIA/LGALS9/LMNA/MCC/MITF/MOV10/MPZ/MYL2/NUBP1/ATP1A2/NPPC/NRAS/OPRL1/PAFAH2/ATP5B/PARK2/SPOCK3/SIRT6/ATP8A2/PI3/PITX2/PKHD1/PLA2G2A/PLAGL1/PRKAG3/PML/RIPPLY3/TLR9/BANP/PPP1CB/DNAJC17/FANCI/PRKAR1B/IFT122/LMBRD1/PAG1/MAP2K2/PRKRIR/PROC/HTRA1/CDC42SE1/PSMD7/SLURP1/PTGFR/TENM2/GATAD2B/PTPRE/CREBZF/FAM60A/TRIM27/RGS12/SCT/NOD2/GZF1/BMP4/ZNF649/SLC8A1/BMPR1B/SLIT1/BPI/STK3/SUPT6H/BST2/ZEB1/TERF1/TNFAIP3/TNFRSF1A/CCR2/TNFRSF4/WNT10B/RAB7A/CARD14/LST1/ZC3H12A/CPEB4/CALR/SLIRP/CAST/CAPZB/NR0B2/CASQ1/HOPX/IFITM1/SCIN/CDK10/RUNX1/TP63/RUNX3/SERPINA6/IRS2/ACTN1/CRADD/ALDH1A2/SPHK1/SKAP2/ERI1/PIAS2/CBFA2T2/RSAD2/AURKB/DAPL1/ADIPOQ/N4BP1/RAPGEF2/FGF19/NR1H4	182
GO:0051216	cartilage development	17/590	179/17046	0.00016	0.01691	0.01467	LECT1/CHI3L1/COMP/FAM101A/BMP10/HOXC4/RSPO2/HES5/NPPC/CSGALNACT1/MAPK3/BMP4/BMPR1B/ZEB1/WNT10B/SCIN/RUNX3	17
GO:0008285	negative regulation of cell proliferation	39/590	608/17046	0.00017	0.01691	0.01467	ABI1/CDKN1C/SPEG/GJB6/LECT1/ADM/FGF10/MORC3/ZC3H12D/IGF1/INHBA/IRF1/LGALS9/MCC/NPPC/SIRT6/ATP8A2/PLA2G2A/PML/RIPPLY3/IFT122/PRKRIR/SLURP1/BMP4/BMPR1B/STK3/ZEB1/TNFAIP3/WNT10B/LST1/IFITM1/SCIN/CDK10/RUNX1/RUNX3/ALDH1A2/SKAP2/ADIPOQ/RAPGEF2	39
GO:0048856	anatomical structure development	204/590	4751/17046	0.00017	0.01719	0.01491	ABI1/FARP1/CDKN1C/SPEG/SPON2/TACC2/PDPN/CELF1/GJB6/TMED10/LECT1/ESM1/CHI3L1/CHRNA1/GPRIN1/CLN5/CCR1/SEZ6/CNP/COL9A3/COMP/ADM/IL31RA/CPM/CPS1/ZNF358/FAM101A/CSTA/SMYD1/SH3D19/CYLD/ESCO2/CYP11A1/BHLHA15/DLG2/DMBT1/EEF2/EFNA2/EIF4G1/EPHA1/ESR1/FGA/FGF10/BTBD3/SBNO2/FOXL1/FOXC2/EXPH5/AKR1B1/NFASC/EPB41L3/FLOT2/NEDD4L/SYNE1/PSD3/ARHGEF18/MAPK8IP2/TSSK2/VGLL2/DFNB31/GAS2/LCE2B/GATM/FGF22/NPTN/BMP10/TMOD4/SOX8/HLA-DOA/NR4A1/ACACB/HOXC4/HSD11B1/HSP90AB1/TFAP2E/ID3/RSPO2/IGF1/IGF2/LCE1C/LCE1D/LCE2D/IL1RN/AQP2/IL11RA/FOXK2/AQP5/INHBA/IRF1/ITGA7/ITGB2/ITGB7/IVL/JUP/KCNJ8/KDR/AMIGO3/HES5/AFF3/LAMA3/STMN1/ARHGDIA/LGALS9/LHCGR/LMNA/LMO2/MC2R/MGAT1/MITF/LHX8/MYL2/NEU1/NFATC3/NHLH2/NPPC/NRAS/ATP5B/PARK2/UTP11L/C11orf73/SIRT6/PDE6B/ATP8A2/PITX2/PKHD1/PKM/PLAGL1/PML/RIPPLY3/SSH1/MXRA8/FBLIM1/PALMD/HERC6/ELP3/CHRNA9/PARVA/IFT122/CSGALNACT1/MAPK3/MAP2K2/HTRA1/CDC42SE1/RGMA/PSMD7/TENM2/ERMN/MARK4/ACTA2/BGLAP/SCT/CCL11/STRA6/GZF1/DNAI2/TMEM237/VPS33A/BMP4/SLC8A1/BMPR1B/SLIT1/BOK/STK3/SUPT6H/ZEB1/TEAD3/TGM2/TCHH/TNFAIP3/TNFRSF1A/TRPC6/CCR2/WNT10B/ZAP70/LST1/ZC3H12A/C6orf25/CALR/SLIRP/CAST/CAPZB/ANTXR1/BFSP2/NR0B2/CASQ1/HOPX/TTBK1/SCIN/RUNX1/TP63/RUNX3/IRS2/ACTN1/TNFRSF11A/ALDH1A2/SPHK1/CBFA2T2/RSAD2/CD8A/ADIPOQ/MICAL2/RAPGEF2/CD79A/FGF19	204
GO:0042692	muscle cell differentiation	26/590	346/17046	0.00019	0.01849	0.01604	SPEG/ADM/SMYD1/BHLHA15/FGF10/FLOT2/SYNE1/BMP10/TMOD4/SOX8/ID3/IGF1/IGF2/LMNA/MYL2/NFATC3/PITX2/BMP4/SLC8A1/SUPT6H/ZEB1/WNT10B/CALR/CAST/CASQ1/HOPX	26
GO:0002682	regulation of immune system process	73/590	1391/17046	0.00022	0.02049	0.01778	ABI1/TANK/KLRG1/SPON2/CCR1/MAP3K8/IL31RA/CYLD/DMBT1/A2M/FCGR2A/FGF10/FLOT2/PUM2/SLC37A4/FGF22/FFAR2/HLA-DOA/HLA-DPA1/NR4A1/HSP90AB1/ZC3H12D/IGF1/IGF2/INHBA/IRF1/ITGB2/ITGB7/KDR/HES5/LGALS9/MITF/MOV10/NFATC3/NRAS/PML/TLR9/TREM1/PAG1/MAPK3/MAP2K2/HTRA1/PSMD7/PTPRE/TRIM27/BGLAP/NOD2/BMP4/BPI/STAT2/SUPT6H/BST2/ZEB1/TNFAIP3/CCR2/TNFRSF4/ZAP70/CA7/LST1/ZC3H12A/CALR/IFITM1/SCIN/RUNX1/IRS2/SKAP2/RSAD2/CD8A/ADIPOQ/NUP93/RAPGEF2/CD79A/FGF19	73
GO:0019725	cellular homeostasis	44/590	725/17046	0.00022	0.02049	0.01778	TRDN/GJB6/EGLN2/CHRNA1/CLN5/CCR1/ADM/ESR1/NEDD4L/NPTN/HK1/NME9/IL1R1/AQP2/AQP5/AQP9/NUBP1/ATP1A2/OPRL1/ATP5B/PARK2/PDE6B/PKHD1/PML/SLC30A10/CHRNA9/SLAMF8/RFC2/RPA3/CCL11/BMP4/SLC4A1/SLC8A1/TERF1/TGM2/TRPC6/CCR2/CA7/CACNA1E/RAB7A/CALR/ATP13A4/CASQ1/IRS2	44
GO:0048878	chemical homeostasis	54/590	949/17046	0.00022	0.02049	0.01778	TRDN/GJB6/CLN5/CCR1/ADM/CPS1/DDB1/BHLHA15/ESR1/NEDD4L/SLC37A4/STEAP2/NPTN/FFAR2/HK1/IGF1/IL1R1/AQP2/AQP5/AQP9/KCNH2/KDR/LDLR/NUBP1/ATP1A2/OPRL1/ATP5B/PARK2/SIRT6/PDE6B/PKHD1/PML/SLC30A10/CHRNA9/PRKAR1B/SLAMF8/CCL11/ABHD4/BMP4/SLC4A1/SLC8A1/SLC9A3/TGM2/TRPC6/CCR2/CA7/CACNA1E/RAB7A/CALR/ATP13A4/CASQ1/TP63/IRS2/ADIPOQ	54
GO:0098602	single organism cell adhesion	43/590	704/17046	0.00023	0.02049	0.01778	CDH9/PDPN/PKP3/MAP3K8/CSTA/CYLD/DDOST/EPHA1/FGA/NFASC/FLOT2/HLA-DOA/HLA-DPA1/ZC3H12D/IGF1/IGF2/IL1RN/IRF1/ITGA7/ITGB2/ITGB7/JUP/LCP1/LGALS9/NFATC3/PKHD1/APBB1IP/FBLIM1/PARVA/PAG1/TENM2/NOD2/BMP4/ZEB1/CCR2/TNFRSF4/ZAP70/CALR/ANTXR1/ESAM/RSAD2/CD8A/ADIPOQ	43
GO:0019221	cytokine-mediated signaling pathway	34/590	512/17046	0.00023	0.02049	0.01778	CCR1/IL31RA/EIF4G1/NUP210/HLA-DPA1/HSP90AB1/IL1R1/IL1RN/IL10RA/IL11RA/IL12RB2/IL15RA/IRF1/OAS2/IL21R/PARK2/PML/MAPK3/PSMD7/CCL11/STAT2/BST2/TNFRSF1A/CCR2/TNFRSF4/CARD14/RAE1/IFITM1/TNFRSF11A/SPHK1/CCRL2/RSAD2/ADIPOQ/NUP93	34

GO:0034097	response to cytokine	44/590	732/17046	0.00027	0.02402	0.02084	CHI3L1/CCR1/IL31RA/CYP11A1/DDOST/EIF4G1/FGA/SBNO2/NUP210/HLA-DPA1/HSP90AB1/IL1R1/IL1RN/IL10RA/IL11RA/IL12RB2/IL15RA/IRF1/ITIH4/AFF3/LGALS9/OAS2/IL21R/PARK2/PML/MAPK3/PSMD7/CCL11/STAT2/BST2/TNFRSF1A/CCR2/TNFRSF4/CARD14/TRIM63/RAE1/IFITM1/TNFRSF11A/ALDH1A2/SPHK1/CCRL2/RSAD2/ADIPOQ/NUP93	44
GO:0032720	negative regulation of tumor necrosis factor production	7/590	38/17046	0.00028	0.02476	0.02148	LGALS9/TRIM27/NOD2/BPI/TNFAIP3/ZC3H12A/ADIPOQ	7
GO:0045321	leukocyte activation	40/590	647/17046	0.00029	0.02482	0.02153	CHGA/MAP3K8/IL31RA/CYLD/DDOST/FGF10/SBNO2/FLOT2/HLA-DOA/HLA-DPA1/ZC3H12D/IGF1/IGF2/INHBA/IRF1/ITGB2/LCP1/LGALS9/NFATC3/IL21R/APBB1IP/PAG1/PTPRE/NOD2/BMP4/BPI/SUPT6H/BST2/ZEB1/TNFAIP3/CCR2/TNFRSF4/ZAP70/LST1/ZC3H12A/IRS2/SKAP2/RSAD2/CD8A/CD79A	40
GO:0043207	response to external biotic stimulus	47/590	802/17046	0.0003	0.02482	0.02153	SPON2/GJB6/HNRNPUL1/CHGA/CNP/ADM/CPS1/CYP11A1/DMBT1/FGA/FGF10/SBNO2/PUM2/SLC37A4/GUCY1A3/IL1RN/IL10RA/IL12RB2/IRF1/KCNJ8/STMN1/LGALS9/OAS2/PLA2G2A/PML/TLR9/TREM1/MAPK3/HTRA1/SLAMF8/PTGFR/CREBZF/ACTA2/CCL11/NOD2/BPI/STAT2/BST2/TNFAIP3/TNFRSF1A/CA7/ZC3H12A/IFITM1/TNFRSF11A/RSAD2/CD8A/NUP93	47
GO:0051707	response to other organism	47/590	802/17046	0.0003	0.02482	0.02153	SPON2/GJB6/HNRNPUL1/CHGA/CNP/ADM/CPS1/CYP11A1/DMBT1/FGA/FGF10/SBNO2/PUM2/SLC37A4/GUCY1A3/IL1RN/IL10RA/IL12RB2/IRF1/KCNJ8/STMN1/LGALS9/OAS2/PLA2G2A/PML/TLR9/TREM1/MAPK3/HTRA1/SLAMF8/PTGFR/CREBZF/ACTA2/CCL11/NOD2/BPI/STAT2/BST2/TNFAIP3/TNFRSF1A/CA7/ZC3H12A/IFITM1/TNFRSF11A/RSAD2/CD8A/NUP93	47
GO:0035295	tube development	37/590	585/17046	0.00032	0.02592	0.02248	PDPN/CHI3L1/ADM/CPS1/ZNF358/ESR1/FGF10/FOXL1/FOXC2/AKR1B1/SOX8/HSD11B1/RSPO2/IGF1/AQP2/KDR/HES5/NFATC3/C11orf73/PITX2/PML/IFT122/MAPK3/MAP2K2/SCT/CCL11/STRA6/GZF1/BMP4/SLC8A1/STK3/ZEB1/TGM2/HOPX/TP63/ALDH1A2/MICAL2	37
GO:0071407	cellular response to organic cyclic compound	26/590	359/17046	0.00033	0.0268	0.02325	CPS1/CYP11A1/ESR1/FGA/MLC1/NR4A1/HRH1/HSP90AB1/IL1RN/INHBA/AQP9/JUP/ATP1A2/P2RY6/PARK2/SSH1/BGLAP/BMP4/SLC8A1/WNT10B/NR0B2/TRIM63/RAE1/ADIPOQ/RAPGEF2/NR1H4	26
GO:1903556	negative regulation of tumor necrosis factor superfamily cytokine production	7/590	40/17046	0.0004	0.03098	0.02687	LGALS9/TRIM27/NOD2/BPI/TNFAIP3/ZC3H12A/ADIPOQ	7
GO:0016337	single organismal cell-cell adhesion	40/590	657/17046	0.0004	0.03098	0.02687	CDH9/PDPN/PKP3/MAP3K8/CSTA/CYLD/DDOST/FGA/NFASC/FLOT2/HLA-DOA/HLA-DPA1/ZC3H12D/IGF1/IGF2/IL1RN/IRF1/ITGA7/ITGB2/ITGB7/JUP/LCP1/LGALS9/NFATC3/PKHD1/APBB1IP/FBLIM1/PARVA/PAG1/TENM2/NOD2/BMP4/ZEB1/CCR2/TNFRSF4/ZAP70/ESAM/RSAD2/CD8A/ADIPOQ	40
GO:1901701	cellular response to oxygen-containing compound	54/590	975/17046	0.00043	0.03269	0.02836	SPON2/GJB6/AP3S1/CPS1/CYP11A1/EIF4G1/ESR1/FGF10/SBNO2/FOXC2/AKR1B1/MLC1/FGF22/FFAR2/GRB10/NR4A1/IGF2/IL1R1/IL1RN/AQP2/INHBA/AQP9/JUP/IPO5/NRAS/P2RY6/PARK2/PRKAG3/TLR9/SSH1/PRKAR1B/LMBRD1/MAPK3/MAP2K2/PSMD7/PTGFR/PTPRE/BGLAP/NOD2/SLC8A1/ZEB1/TNFAIP3/TRPC6/WNT10B/CACNA1E/ZC3H12A/CPEB4/TRIM63/IRS2/ALDH1A2/ADIPOQ/RAPGEF2/FGF19/NR1H4	54
GO:0042221	response to chemical	169/590	3882/17046	0.00043	0.03269	0.02836	CDKN1C/SPON2/GJB6/TMED10/LECT1/CHGA/CHI3L1/ERLIN2/EGLN2/CHRNA1/CHRNA2/AP3S1/CCR1/CNP/COL9A3/ADM/IL31RA/OR2A14/CPS1/PARP4/CYP11A1/DDOST/ZNF366/BHLHA15/ABAT/AGXT/EFNA2/EIF4G1/EPHA1/ESR1/FGA/FGF10/SBNO2/FOXC2/AKR1B1/NFASC/MLC1/NUP210/NEDD4L/ARHGEF18/STEAP2/GATM/FGF22/NPTN/BMP10/FFAR2/GRB10/GUCY1A3/HAS1/HLA-DPA1/NR4A1/HRH1/HSPA1L/HSP90AB1/HTR3A/IGF2/LCE1D/IL1R1/IL1RN/IL10RA/AQP2/IL11RA/IL12RB2/IL15RA/IL16/INHBA/IRF1/AQP9/ITGB2/ITIH4/JUP/KCNH2/KCNJ8/KCNMB1/KDR/IPO5/HES5/AFF3/OR2A5/ARHGDIA/LGALS9/LHCGR/LMNA/LMO2/MOV10/ATP1A2/NFATC3/NPPC/NRAS/OAS2/OPRL1/OR2C1/OR3A2/SLC22A18/P2RY6/IL21R/PARK2/PITX2/PKM/PRKAG3/PML/TLR9/TREM1/SSH1/CYP2W1/PPP1CB/SLC47A1/SLC30A10/CHRNA9/PARVA/PRKAR1B/LMBRD1/MAPK3/MAP2K2/HTRA1/SLAMF8/RGMA/PSMD7/PTGFR/PLEKHG5/TENM2/PTPRE/RIT2/BGLAP/CCL11/NOD2/BMP4/SLC8A1/SLC9A3/BMPR1B/SLIT1/SRP68/STAT2/BST2/TCEB2/ZEB1/TERF1/TNFAIP3/TNFRSF1A/TRPC6/TRPM2/CCR2/TNFRSF4/WNT10B/CACNA1E/RAB7A/CARD14/ZC3H12A/CPEB4/CALR/NR0B2/CASQ1/TRIM63/RAE1/IFITM1/RUNX1/RUNX3/IRS2/TNFRSF11A/ALDH1A2/SPHK1/HSPB3/CCRL2/RSAD2/ADIPOQ/NUP93/RAPGEF2/FGF19/NR1H4	169
GO:0098609	cell-cell adhesion	47/590	816/17046	0.00044	0.03275	0.02841	CDH9/CDH12/PDPN/PKP3/FAT3/MAP3K8/CSTA/CYLD/DDOST/FAT2/FGA/NFASC/FLOT2/NPTN/HLA-DOA/HLA-DPA1/ZC3H12D/IGF1/IGF2/IL1RN/IRF1/ITGA7/ITGB2/ITGB7/JUP/AMIGO3/LCP1/LGALS9/NFATC3/PKHD1/APBB1IP/FBLIM1/PARVA/PAG1/PCDHGB3/TENM2/NOD2/PCDH20/BMP4/ZEB1/CCR2/TNFRSF4/ZAP70/ESAM/RSAD2/CD8A/ADIPOQ	47

GO:0012501	programmed cell death	85/590	1718/17046	0.00045	0.03278	0.02844	GJB6/CHI3L1/EGLN2/CARD16/COMP/MAP3K8/ADM/IL31RA/CYLD/DDB1/ESR1/FGA/FGF10/FHIT/FOXC2/EPB41L3/DIP2A/PPP1R13B/ARHGEF18/RYBP/RNF144B/GAS2/BMP10/SOX8/HSP90AB1/ID3/IGF1/IL1RN/INHBA/IRF1/ITGB2/KDR/ARHGDIA/LGALS9/LMNA/MPZ/PAFAH2/PARK2/UTP11L/PKHD1/PKM/PLAGL1/PML/PPP2R2B/MAPK3/PROC/PAK6/PSMD7/PTGFR/PLEKHG5/MARK4/SCT/NOD2/BMP4/BMPR1B/BOK/STK3/TERF1/TGM2/TNFAIP3/TNFRSF1A/TRAF5/TNFRSF4/WNT10B/CARD14/BCL2L14/FAM188A/ZC3H12A/CPEB4/CLPTM1L/CALR/CAST/SCIN/TP63/RUNX3/IRS2/ACTN1/CRADD/ALDH1A2/SPHK1/MAP3K6/AURKB/DAPL1/ADIPOQ/RAPGEF2	85
GO:0008643	carbohydrate transport	16/590	178/17046	0.00047	0.03419	0.02966	NUP210/SLC37A4/GRB10/HK1/IGF1/AQP2/AQP5/AQP9/SIRT6/PRKAG3/LMBRD1/RAE1/IRS2/ADIPOQ/NUP93/FGF19	16
GO:0071345	cellular response to cytokine stimulus	39/590	642/17046	0.00049	0.03474	0.03014	CHI3L1/CCR1/IL31RA/CYP11A1/EIF4G1/FGA/SBNO2/NUP210/HLA-DPA1/HSP90AB1/IL1R1/IL1RN/IL10RA/IL11RA/IL12RB2/IL15RA/IRF1/LGALS9/OAS2/IL21R/PARK2/PML/MAPK3/PSMD7/CCL11/STAT2/BST2/TNFRSF1A/CCR2/TNFRSF4/CARD14/RAE1/IFITM1/TNFRSF11A/SPHK1/CCRL2/RSAD2/ADIPOQ/NUP93	39
GO:0042476	odontogenesis	12/590	112/17046	0.0005	0.03474	0.03014	ADM/FGF10/ID3/RSPO2/AQP5/INHBA/LHX8/PITX2/HTRA1/BGLAP/BMP4/TP63	12
GO:0046660	female sex differentiation	12/590	112/17046	0.0005	0.03474	0.03014	COL9A3/ESR1/FGF10/INHBA/KDR/LHCGR/LHX8/PITX2/STRA6/BMP4/BMPR1B/TP63	12
GO:0042592	homeostatic process	73/590	1434/17046	0.00053	0.03633	0.03152	TRDN/GJB6/EGLN2/CHRNA1/CLN5/CCR1/ADM/IL31RA/CPS1/DDB1/BHLHA15/ABAT/ESR1/AKR1B1/NEDD4L/SLC37A4/DFNB31/STEAP2/NPTN/AMPD3/FFAR2/HK1/NME9/IGF1/IL1R1/IL1RN/AQP2/AQP5/INHBA/AQP9/KCNH2/KDR/LDLR/LGALS9/NUBP1/ATP1A2/OPRL1/ATP5B/PARK2/SIRT6/PDE6B/PKHD1/PML/TLR9/SLC30A10/CHRNA9/PRKAR1B/SLAMF8/RFC2/RPA3/BGLAP/CCL11/ABHD4/NOD2/BMP4/SLC4A1/SLC8A1/SLC9A3/TERF1/TGM2/TNFAIP3/TRPC6/CCR2/CA7/CACNA1E/RAB7A/CALR/ATP13A4/CASQ1/TP63/IRS2/TNFRSF11A/ADIPOQ	73
GO:1901698	response to nitrogen compound	53/590	961/17046	0.00054	0.03646	0.03163	TMED10/CHRNA1/CHRNA2/AP3S1/ADM/CPS1/CYP11A1/ABAT/AGXT/EIF4G1/FGA/FGF10/FOXC2/AKR1B1/GATM/FGF22/GRB10/NR4A1/HRH1/HTR3A/IGF2/IL1R1/IL1RN/AQP9/JUP/KCNJ8/IPO5/ATP1A2/NRAS/PARK2/PKM/PRKAG3/TLR9/SSH1/CHRNA9/PRKAR1B/LMBRD1/MAPK3/MAP2K2/PSMD7/PTPRE/NOD2/BMP4/SLC8A1/ZEB1/TNFAIP3/WNT10B/CPEB4/IRS2/ADIPOQ/RAPGEF2/FGF19/NR1H4	53
GO:0050793	regulation of developmental process	94/590	1953/17046	0.00055	0.03646	0.03163	PDPN/CELF1/LECT1/CHI3L1/CCR1/SEZ6/ADM/FAM101A/SMYD1/SH3D19/CYLD/BHLHA15/DIO3/DMBT1/EIF4G1/EPHA1/ESR1/FGA/FGF10/FOXC2/EPB41L3/FLOT2/NEDD4L/ARHGEF18/GAS2/NPTN/BMP10/SOX8/HLA-DOA/ACACB/ID3/RSPO2/IGF1/IL1RN/INHBA/IRF1/ITGA7/ITGB2/JUP/KDR/AMIGO3/HES5/LAMA3/ARHGDIA/LGALS9/LMNA/MITF/NEU1/NPPC/NRAS/PARK2/ATP8A2/PLA2G2A/PML/SSH1/FBLIM1/PALMD/PARVA/IFT122/MAP2K2/CDC42SE1/ERMN/BGLAP/CCL11/VPS33A/BMP4/SLC8A1/BMPR1B/SLIT1/STK3/SUPT6H/ZEB1/TEAD3/TNFAIP3/TNFRSF1A/CCR2/WNT10B/ZAP70/LST1/ZC3H12A/CALR/CAPZB/HOPX/TTBK1/IFITM1/SCIN/RUNX1/TP63/RUNX3/SPHK1/PIAS2/CBFA2T2/ADIPOQ/RAPGEF2	94
GO:0010243	response to organonitrogen compound	50/590	893/17046	0.00055	0.03646	0.03163	TMED10/CHRNA1/CHRNA2/AP3S1/ADM/CPS1/CYP11A1/ABAT/AGXT/EIF4G1/FGA/FGF10/FOXC2/AKR1B1/GATM/FGF22/GRB10/NR4A1/HRH1/HTR3A/IGF2/IL1RN/AQP9/JUP/IPO5/ATP1A2/NRAS/PARK2/PKM/PRKAG3/TLR9/SSH1/CHRNA9/PRKAR1B/LMBRD1/MAPK3/MAP2K2/PSMD7/PTPRE/NOD2/SLC8A1/ZEB1/TNFAIP3/WNT10B/CPEB4/IRS2/ADIPOQ/RAPGEF2/FGF19/NR1H4	50
GO:0060439	trachea morphogenesis	4/590	12/17046	0.00056	0.0367	0.03184	RSPO2/MAPK3/MAP2K2/BMP4	4
GO:1901700	response to oxygen-containing compound	74/590	1462/17046	0.00057	0.0367	0.03184	SPON2/GJB6/AP3S1/CNP/ADM/CPS1/CYP11A1/ABAT/AGXT/EIF4G1/ESR1/FGA/FGF10/SBNO2/FOXC2/AKR1B1/MLC1/GATM/FGF22/FFAR2/GRB10/NR4A1/HTR3A/IGF2/IL1R1/IL1RN/IL10RA/AQP2/IL12RB2/INHBA/AQP9/JUP/KCNJ8/IPO5/LGALS9/ATP1A2/NPPC/NRAS/OPRL1/P2RY6/PARK2/PKM/PRKAG3/TLR9/SSH1/PRKAR1B/LMBRD1/MAPK3/MAP2K2/PSMD7/PTGFR/PTPRE/BGLAP/NOD2/BMP4/SLC8A1/ZEB1/TNFAIP3/TRPC6/TRPM2/WNT10B/CACNA1E/ZC3H12A/CPEB4/CALR/NR0B2/TRIM63/IRS2/TNFRSF11A/ALDH1A2/ADIPOQ/RAPGEF2/FGF19/NR1H4	74
GO:0009100	glycoprotein metabolic process	28/590	413/17046	0.00058	0.03674	0.03187	GNE/LECT1/GALNT15/SLC51B/NEU4/EGFLAM/PARP4/B3GLCT/MGAT5B/DDOST/TRAK1/FOXL1/ST6GALNAC3/GBGT1/SLC17A5/EOGT/IGF1/MUC21/MGAT1/NEU1/OAS2/SPOCK3/CHST15/SIRT6/CSGALNACT1/BMPR1B/MOGS/CALR	28
GO:0048522	positive regulation of cellular process	183/590	4283/17046	0.00059	0.03674	0.03187	ABI1/CDKN1C/TRDN/PDPN/RER1/ESM1/CHI3L1/ERLIN2/CCR1/SLC51B/SEZ6/MAP3K8/ADM/IL31RA/EGFLAM/MIB2/MPP7/SMYD1/SH3D19/CYLD/DDB1/BHLHA15/DMBT1/ABAT/EEF2/EIF4G1/EPHA1/ESR1/FGA/FGF10/SBNO2/FOXC2/EXPH5/AKR1B1/FLOT2/NEDD4L/PUM2/ARHGEF18/MAPK8IP2/VGLL2/RNF144B/PABPC1/DNAJC2/FGF22/NPTN/BMP10/GPR26/FFAR2/GRB10/GUCY1A3/SOX8/HK1/HLA-DPA1/ANXA13/NR4A1/ACACB/HRH1/HSPA1L/HSP90AB1/TFAP2E/ID3/RSPO2/IGF1/IGF2/IL1RN/IL12RB2/FOXK2/INHBA/IRF1/JUP/KCNH2/KDR/IPO5/AMIGO3/HES5/STMN1/LCP1/LDLR/ARHGDIA/LGALS9/LHCGR/LMNA/LMO2/MC2R/MFNG/MITF/NEU1/NFATC3/NHLH2/NPPC/NRAS/OPRL1/P2RY6/PARK2/UTP11L/PRR16/SIRT6/ATP8A2/PITX2/PKHD1/PLA2G2A/PLAGL1/PML/TLR9/APBB1IP/BANP/ELP3/GOLPH3L/FANCI/PRKAR1B/PAG1/MAPK3/MAP2K2/MRAP/HTRA1/PAK6/ARNTL2/RGMA/PSMD7/PTGFR/PLEKHG5/TENM2/MARK4/TRIM27/SCT/CCL11/NPAS3/NOD2/BMP4/ZNF649/SLC8A1/BMPR1B/BOK/STK3/SUPT6H/BST2/TCEB2/ZEB1/TEAD3/TERF1/TGM2/TNFAIP3/TNFRSF1A/TRAF5/TRPC6/CCR2/TNFRSF4/WNT10B/ZAP70/CA7/RAB7A/CARD14/BCL2L14/CSPP1/ZC3H12A/CALR/NR0B2/HOPX/IFITM1/SCIN/CDK10/RUNX1/TP63/RUNX3/IRS2/CRADD/TNFRSF11A/ALDH1A2/SPHK1/SKAP2/PRC1/PIAS2/MAP3K6/SYT7/CBFA2T2/RSAD2/AURKB/CD8A/ADIPOQ/MICAL2/RAPGEF2/FGF19/NR1H4	183

GO:0048731	system development	173/590	4014/17046	0.00059	0.03674	0.03187	ABI1/FARP1/CDKN1C/SPEG/SPON2/TACC2/PDPN/GJB6/TMED10/LECT1/ESM1/CHI3L1/CHRNA1/GPRIN1/CLN5/CCR1/SEZ6/CNP/COL9A3/COMP/ADM/IL31RA/CPS1/ZNF358/FAM101A/CSTA/SMYD1/CYLD/ESCO2/CYP11A1/DLG2/EEF2/EFNA2/EIF4G1/EPHA1/ESR1/FGA/FGF10/BTBD3/SBNO2/FOXL1/FOXC2/EXPH5/AKR1B1/NFASC/EPB41L3/NEDD4L/PSD3/MAPK8IP2/VGLL2/DFNB31/LCE2B/GATM/FGF22/NPTN/BMP10/SOX8/HLA-DOA/NR4A1/ACACB/HOXC4/HSD11B1/HSP90AB1/ID3/RSPO2/IGF1/IGF2/LCE1C/LCE1D/LCE2D/AQP2/AQP5/INHBA/IRF1/ITGA7/IVL/JUP/KCNJ8/KDR/AMIGO3/HES5/STMN1/ARHGDIA/LGALS9/LHCGR/LMNA/LMO2/MC2R/MITF/LHX8/MYL2/NEU1/NFATC3/NHLH2/NPPC/NRAS/ATP5B/PARK2/UTP11L/C11orf73/SIRT6/PDE6B/ATP8A2/PITX2/PKHD1/PKM/PLAGL1/PML/RIPPLY3/SSH1/MXRA8/HERC6/ELP3/CHRNA9/PARVA/IFT122/CSGALNACT1/MAPK3/MAP2K2/HTRA1/RGMA/PSMD7/TENM2/MARK4/ACTA2/BGLAP/SCT/CCL11/STRA6/GZF1/VPS33A/BMP4/SLC8A1/BMPR1B/SLIT1/BOK/STK3/SUPT6H/ZEB1/TGM2/TCHH/TNFAIP3/TRPC6/CCR2/WNT10B/ZAP70/LST1/ZC3H12A/C6orf25/CALR/CAST/BFSP2/NR0B2/CASQ1/HOPX/TTBK1/SCIN/RUNX1/TP63/RUNX3/IRS2/ACTN1/TNFRSF11A/ALDH1A2/SPHK1/CBFA2T2/RSAD2/CD8A/ADIPOQ/MICAL2/RAPGEF2/CD79A/FGF19	173
GO:0009966	regulation of signal transduction	110/590	2366/17046	0.00063	0.03856	0.03345	FARP1/RCAN2/CDKN1C/LECT1/ESM1/CHI3L1/CCR1/SEZ6/TNFAIP8L1/MAP3K8/ADM/IL31RA/MIB2/MPP7/CYLD/ZNF366/A2M/ESR1/SPATA13/FGA/FGF10/FOXL1/AKR1B1/RHOBTB2/NEDD4L/PSD3/PUM2/ARHGEF18/MAPK8IP2/ALS2CL/FGF22/NPTN/CYTH4/BMP10/GRB10/HSP90AB1/RSPO2/IGF1/IGF2/IL1RN/INHBA/IRF1/JUP/KDR/HES5/ARHGDIA/LGALS9/LHCGR/LMNA/MCC/MFNG/PLEKHG7/NRAS/PARK2/PDE6B/PKHD1/PLA2G2A/PML/TLR9/PPP1CB/IFT122/LMBRD1/PAG1/MAPK3/MAP2K2/HTRA1/PAK6/PSMD7/PLEKHG5/PTPRE/RGS12/CCL11/NOD2/ARHGAP9/TMEM237/BMP4/BMPR1B/STAT2/STK3/BST2/ZEB1/TNFAIP3/TNFRSF1A/TRAF5/WNT10B/ZAP70/RAB7A/CARD14/BCL2L14/CALR/CMAHP/CDK10/RUNX1/TP63/RUNX3/IRS2/CRADD/TNFRSF11A/SPHK1/SKAP2/STARD13/PIAS2/MAP3K6/RSAD2/CD8A/TRIP10/ADIPOQ/RAPGEF2/IQSEC1/FGF19	110
GO:0055082	cellular chemical homeostasis	37/590	607/17046	0.00064	0.03865	0.03353	TRDN/GJB6/CLN5/CCR1/ADM/ESR1/NEDD4L/NPTN/HK1/IL1R1/AQP2/AQP5/AQP9/NUBP1/ATP1A2/OPRL1/ATP5B/PDE6B/PKHD1/PML/SLC30A10/CHRNA9/SLAMF8/CCL11/BMP4/SLC4A1/SLC8A1/TGM2/TRPC6/CCR2/CA7/CACNA1E/RAB7A/CALR/ATP13A4/CASQ1/IRS2	37
GO:1900034	regulation of cellular response to heat	9/590	70/17046	0.00066	0.03984	0.03456	NUP210/DNAJC2/HSPA1L/HSP90AB1/C11orf73/MAPK3/RPA3/RAE1/NUP93	9
GO:0010646	regulation of cell communication	124/590	2731/17046	0.00068	0.04006	0.03475	FARP1/RCAN2/CDKN1C/TRDN/LECT1/ESM1/CHI3L1/CCR1/SEZ6/TNFAIP8L1/MAP3K8/ADM/IL31RA/MIB2/MPP7/CYLD/ZNF366/ABAT/A2M/ESR1/SPATA13/FGA/FGF10/FOXL1/AKR1B1/RHOBTB2/NEDD4L/PSD3/PUM2/ARHGEF18/MAPK8IP2/ALS2CL/PNKD/FGF22/NPTN/CYTH4/BMP10/FFAR2/GRB10/HK1/HRH1/HSP90AB1/RSPO2/IGF1/IGF2/IL1RN/INHBA/IRF1/JUP/KDR/HES5/ARHGDIA/LGALS9/LHCGR/LMNA/MCC/MFNG/PLEKHG7/ATP1A2/NRAS/PARK2/PDE6B/PKHD1/PLA2G2A/PML/TLR9/PPP1CB/PRKAR1B/IFT122/LMBRD1/PAG1/MAPK3/MAP2K2/HTRA1/PAK6/PSMD7/PLEKHG5/PTPRE/RGS12/SCT/CCL11/NOD2/ARHGAP9/TMEM237/BMP4/SLC8A1/BMPR1B/STAT2/STK3/BST2/ZEB1/TNFAIP3/TNFRSF1A/TRAF5/WNT10B/ZAP70/CA7/CACNA1E/RAB7A/CARD14/BCL2L14/CALR/CMAHP/NR0B2/CDK10/RUNX1/TP63/RUNX3/IRS2/CRADD/TNFRSF11A/SPHK1/SKAP2/STARD13/PIAS2/MAP3K6/SYT7/RSAD2/CD8A/TRIP10/ADIPOQ/RAPGEF2/IQSEC1/FGF19	124
GO:0001763	morphogenesis of a branching structure	17/590	203/17046	0.00072	0.04146	0.03597	ADM/ESR1/FGF10/FOXC2/SOX8/RSPO2/IGF1/KDR/NFATC3/PITX2/PML/ERMN/CCL11/GZF1/BMP4/TGM2/TP63	17
GO:0005975	carbohydrate metabolic process	47/590	835/17046	0.00072	0.04146	0.03597	GNE/CHI3L1/CHI3L2/GALNT15/CLN5/SLC51B/NEU4/CPS1/PARP4/B3GLCT/MGAT5B/DDOST/ENO2/TRAK1/AKR1B1/NUP210/SLC37A4/ST6GALNAC3/GBGT1/SLC17A5/DHDH/EOGT/GRB10/HAS1/HK1/HRH1/IGF1/IGF2/MUC21/LHCGR/MGAT1/NEU1/OAS2/PARK2/CHST15/SIRT6/PKM/PRKAG3/PPP1CB/CSGALNACT1/MOGS/CALR/RAE1/IRS2/STBD1/ADIPOQ/NUP93	47
GO:0023051	regulation of signaling	122/590	2685/17046	0.00073	0.04146	0.03597	FARP1/RCAN2/CDKN1C/TRDN/LECT1/ESM1/CHI3L1/CCR1/SEZ6/TNFAIP8L1/MAP3K8/ADM/IL31RA/MIB2/MPP7/CYLD/ZNF366/ABAT/A2M/ESR1/SPATA13/FGA/FGF10/FOXL1/AKR1B1/RHOBTB2/NEDD4L/PSD3/PUM2/ARHGEF18/MAPK8IP2/ALS2CL/PNKD/FGF22/NPTN/CYTH4/BMP10/FFAR2/GRB10/HRH1/HSP90AB1/RSPO2/IGF1/IGF2/IL1RN/INHBA/IRF1/JUP/KDR/HES5/ARHGDIA/LGALS9/LHCGR/LMNA/MCC/MFNG/PLEKHG7/ATP1A2/NRAS/PARK2/PDE6B/PKHD1/PLA2G2A/PML/TLR9/PPP1CB/PRKAR1B/IFT122/LMBRD1/PAG1/MAPK3/MAP2K2/HTRA1/PAK6/PSMD7/PLEKHG5/PTPRE/RGS12/SCT/CCL11/NOD2/ARHGAP9/TMEM237/BMP4/BMPR1B/STAT2/STK3/BST2/ZEB1/TNFAIP3/TNFRSF1A/TRAF5/WNT10B/ZAP70/CA7/CACNA1E/RAB7A/CARD14/BCL2L14/CALR/CMAHP/NR0B2/CDK10/RUNX1/TP63/RUNX3/IRS2/CRADD/TNFRSF11A/SPHK1/SKAP2/STARD13/PIAS2/MAP3K6/SYT7/RSAD2/CD8A/TRIP10/ADIPOQ/RAPGEF2/IQSEC1/FGF19	122
GO:0002694	regulation of leukocyte activation	27/590	399/17046	0.00074	0.04146	0.03597	MAP3K8/IL31RA/CYLD/FGF10/FLOT2/HLA-DOA/HLA-DPA1/ZC3H12D/IGF1/IGF2/INHBA/IRF1/LGALS9/PAG1/PTPRE/NOD2/BMP4/BPI/SUPT6H/ZEB1/TNFAIP3/CCR2/TNFRSF4/ZAP70/LST1/ZC3H12A/IRS2	27
GO:0014070	response to organic cyclic compound	44/590	768/17046	0.00074	0.04146	0.03597	CHRNA1/CHRNA2/ADM/CPS1/CYP11A1/ZNF366/ABAT/AGXT/ESR1/FGA/FGF10/MLC1/GUCY1A3/NR4A1/HRH1/HSP90AB1/HTR3A/IL1RN/INHBA/AQP9/JUP/KCNJ8/ATP1A2/OPRL1/P2RY6/PARK2/SSH1/CHRNA9/MAPK3/PTGFR/BGLAP/NOD2/BMP4/SLC8A1/SLC9A3/WNT10B/CALR/NR0B2/TRIM63/RAE1/ALDH1A2/ADIPOQ/RAPGEF2/NR1H4	44
GO:0009607	response to biotic stimulus	47/590	837/17046	0.00076	0.04146	0.03597	SPON2/GJB6/HNRNPUL1/CHGA/CNP/ADM/CPS1/CYP11A1/DMBT1/FGA/FGF10/SBNO2/PUM2/SLC37A4/GUCY1A3/IL1RN/IL10RA/IL12RB2/IRF1/KCNJ8/STMN1/LGALS9/OAS2/PLA2G2A/PML/TLR9/TREM1/MAPK3/HTRA1/SLAMF8/PTGFR/CREBZF/ACTA2/CCL11/NOD2/BPI/STAT2/BST2/TNFAIP3/TNFRSF1A/CA7/ZC3H12A/IFITM1/TNFRSF11A/RSAD2/CD8A/NUP93	47
GO:0043031	negative regulation of macrophage activation	3/590	6/17046	0.00076	0.04146	0.03597	IL31RA/BPI/ZC3H12A	3

GO:0002695	negative regulation of leukocyte activation	13/590	134/17046	0.00077	0.04146	0.03597	IL31RA/CYLD/ZC3H12D/INHBA/IRF1/LGALS9/PAG1/BMP4/BPI/TNFAIP3/CCR2/LST1/ZC3H12A	13
GO:0046649	lymphocyte activation	34/590	548/17046	0.00077	0.04146	0.03597	MAP3K8/CYLD/DDOST/FGF10/FLOT2/HLA-DOA/HLA-DPA1/ZC3H12D/IGF1/IGF2/INHBA/IRF1/ITGB2/LCP1/LGALS9/NFATC3/IL21R/APBB1IP/PAG1/NOD2/BMP4/SUPT6H/BST2/ZEB1/TNFAIP3/CCR2/TNFRSF4/ZAP70/LST1/IRS2/SKAP2/RSAD2/CD8A/CD79A	34
GO:0006915	apoptotic process	83/590	1700/17046	0.00078	0.04175	0.03622	GJB6/CHI3L1/EGLN2/CARD16/COMP/MAP3K8/ADM/IL31RA/CYLD/DDB1/ESR1/FGA/FGF10/FHIT/FOXC2/EPB41L3/DIP2A/PPP1R13B/ARHGEF18/RYBP/RNF144B/GAS2/BMP10/SOX8/HSP90AB1/ID3/IGF1/IL1RN/INHBA/IRF1/ITGB2/KDR/ARHGDIA/LGALS9/LMNA/MPZ/PAFAH2/PARK2/UTP11L/PKHD1/PLAGL1/PML/PPP2R2B/MAPK3/PROC/PAK6/PSMD7/PTGFR/PLEKHG5/SCT/NOD2/BMP4/BMPR1B/BOK/STK3/TERF1/TGM2/TNFAIP3/TNFRSF1A/TRAF5/TNFRSF4/WNT10B/CARD14/BCL2L14/FAM188A/ZC3H12A/CPEB4/CLPTM1L/CALR/CAST/SCIN/TP63/RUNX3/IRS2/ACTN1/CRADD/ALDH1A2/SPHK1/MAP3K6/AURKB/DAPL1/ADIPOQ/RAPGEF2	83
GO:0048286	lung alveolus development	7/590	45/17046	0.00083	0.04378	0.03798	PDPN/FGF10/IGF1/KDR/STRA6/BMP4/HOPX	7
GO:0043067	regulation of programmed cell death	67/590	1314/17046	0.00087	0.04516	0.03918	EGLN2/CARD16/COMP/MAP3K8/ADM/IL31RA/CYLD/DDB1/ESR1/FGA/FGF10/FOXC2/DIP2A/ARHGEF18/BMP10/SOX8/HSP90AB1/ID3/IGF1/IL1RN/INHBA/KDR/ARHGDIA/LGALS9/LMNA/MITF/MPZ/PAFAH2/PARK2/UTP11L/PKHD1/PML/PROC/PAK6/PSMD7/PTGFR/PLEKHG5/MARK4/SCT/NOD2/BMP4/BMPR1B/BOK/STK3/TERF1/TGM2/TNFAIP3/TRAF5/TNFRSF4/WNT10B/CARD14/BCL2L14/CPEB4/CALR/CAST/SCIN/TP63/RUNX3/IRS2/ACTN1/CRADD/ALDH1A2/SPHK1/MAP3K6/AURKB/ADIPOQ/RAPGEF2	67
GO:1901137	carbohydrate derivative biosynthetic process	42/590	729/17046	0.00087	0.04516	0.03918	GNE/ACOT7/GALNT15/SLC51B/NEU4/ADM/PARP4/B3GLCT/MGAT5B/DDOST/TRAK1/FOXL1/ST6GALNAC3/GBGT1/SLC17A5/AMPD3/EOGT/GUCY1A3/HAS1/ACACB/NME9/IGF1/MUC21/LHCGR/MC2R/MGAT1/NEU1/NPPC/OAS2/OPRL1/ATP5B/CHST15/SIRT6/PKM/CSGALNACT1/MRAP/SCT/BMPR1B/CCR2/MOGS/CALR/QTRT1	42
GO:0061138	morphogenesis of a branching epithelium	16/590	189/17046	0.00091	0.04523	0.03924	ADM/ESR1/FGF10/FOXC2/SOX8/RSPO2/IGF1/KDR/NFATC3/PITX2/PML/CCL11/GZF1/BMP4/TGM2/TP63	16
GO:0071417	cellular response to organonitrogen compound	37/590	619/17046	0.00091	0.04523	0.03924	AP3S1/CPS1/CYP11A1/EIF4G1/FGF10/FOXC2/AKR1B1/FGF22/GRB10/NR4A1/HRH1/IGF2/IL1RN/AQP9/JUP/IPO5/NRAS/PARK2/PRKAG3/TLR9/SSH1/PRKAR1B/LMBRD1/MAPK3/MAP2K2/PSMD7/PTPRE/NOD2/SLC8A1/ZEB1/WNT10B/CPEB4/IRS2/ADIPOQ/RAPGEF2/FGF19/NR1H4	37
GO:0001775	cell activation	48/590	868/17046	0.00093	0.04523	0.03924	CHGA/MAP3K8/IL31RA/CYLD/DDOST/A2M/FGA/FGF10/SBNO2/FLOT2/SCG3/HLA-DOA/HLA-DPA1/ZC3H12D/IGF1/IGF2/INHBA/IRF1/ITGB2/LCP1/LGALS9/NFATC3/IL21R/APBB1IP/PAG1/MAPK3/SLURP1/PTPRE/NOD2/BMP4/BPI/SUPT6H/BST2/ZEB1/TNFAIP3/TRPC6/CCR2/TNFRSF4/ZAP70/LST1/ZC3H12A/CAST/IRS2/ACTN1/SKAP2/RSAD2/CD8A/CD79A	48
GO:0061061	muscle structure development	34/590	554/17046	0.00093	0.04523	0.03924	SPEG/CHRNA1/ADM/SMYD1/BHLHA15/FGF10/FOXC2/FLOT2/SYNE1/VGLL2/BMP10/TMOD4/SOX8/ID3/IGF1/IGF2/ITGA7/LMNA/MYL2/NFATC3/NRAS/SIRT6/PITX2/PLAGL1/STRA6/BMP4/SLC8A1/SUPT6H/ZEB1/WNT10B/CALR/CAST/CASQ1/HOPX	34
GO:0009101	glycoprotein biosynthetic process	25/590	364/17046	0.00093	0.04523	0.03924	GNE/GALNT15/SLC51B/NEU4/PARP4/B3GLCT/MGAT5B/DDOST/TRAK1/FOXL1/ST6GALNAC3/GBGT1/SLC17A5/EOGT/IGF1/MUC21/MGAT1/NEU1/OAS2/CHST15/SIRT6/CSGALNACT1/BMPR1B/MOGS/CALR	25
GO:0070374	positive regulation of ERK1 and ERK2 cascade	14/590	154/17046	0.00094	0.04523	0.03924	CHI3L1/CCR1/FGA/FGF10/KDR/LGALS9/PLA2G2A/MAPK3/CCL11/NOD2/BMP4/TNFRSF11A/RAPGEF2/FGF19	14
GO:0030036	actin cytoskeleton organization	32/590	511/17046	0.00094	0.04523	0.03924	ABI1/FAM101A/EPHA1/PHACTR1/FGF10/MSRB2/EPB41L3/ARHGEF18/PLEK2/BMP10/TMOD4/LCP1/MYL2/PARK2/SSH1/PARVA/TTC17/PAK6/ERMN/TRIM27/CCL11/PARVG/BST2/CALR/CAPZB/ANTXR1/CASQ1/SCIN/ACTN1/TRIP10/MICAL2/IQSEC1	32
GO:0044264	cellular polysaccharide metabolic process	10/590	89/17046	0.001	0.04737	0.04109	CPS1/GRB10/HAS1/IGF1/IGF2/PRKAG3/PPP1CB/CSGALNACT1/IRS2/STBD1	10
GO:0034637	cellular carbohydrate biosynthetic process	9/590	74/17046	0.001	0.04737	0.04109	AKR1B1/GRB10/HAS1/IGF1/IGF2/PRKAG3/PPP1CB/CSGALNACT1/IRS2	9

GO:0070371	ERK1 and ERK2 cascade	18/590	228/17046	0.00101	0.04737	0.04109	CHI3L1/CCR1/FGA/FGF10/IGF1/KDR/LGALS9/PKHD1/PLA2G2A/MAPK3/MAP2K2/CCL11/NOD2/BMP4/TNFRSF11A/ADIPOQ/RAPGEF2/FGF19	18
GO:0008219	cell death	87/590	1816/17046	0.00104	0.04796	0.04161	GJB6/CHI3L1/EGLN2/CARD16/COMP/MAP3K8/ADM/IL31RA/PARP4/CYLD/DDB1/ESR1/FGA/FGF10/FHIT/FOXC2/EPB41L3/DIP2A/PPP1R13B/ARHGEF18/RYBP/RNF144B/GAS2/CLUL1/BMP10/SOX8/HSP90AB1/ID3/IGF1/IL1RN/INHBA/IRF1/ITGB2/KDR/ARHGDIA/LGALS9/LMNA/MPZ/PAFAH2/PARK2/UTP11L/PKHD1/PKM/PLAGL1/PML/PPP2R2B/MAPK3/PROC/PAK6/PSMD7/PTGFR/PLEKHG5/MARK4/SCT/NOD2/BMP4/BMPR1B/BOK/STK3/TERF1/TGM2/TNFAIP3/TNFRSF1A/TRAF5/TNFRSF4/WNT10B/CARD14/BCL2L14/FAM188A/ZC3H12A/CPEB4/CLPTM1L/CALR/CAST/SCIN/TP63/RUNX3/IRS2/ACTN1/CRADD/ALDH1A2/SPHK1/MAP3K6/AURKB/DAPL1/ADIPOQ/RAPGEF2	87
GO:0016265	death	87/590	1816/17046	0.00104	0.04796	0.04161	GJB6/CHI3L1/EGLN2/CARD16/COMP/MAP3K8/ADM/IL31RA/PARP4/CYLD/DDB1/ESR1/FGA/FGF10/FHIT/FOXC2/EPB41L3/DIP2A/PPP1R13B/ARHGEF18/RYBP/RNF144B/GAS2/CLUL1/BMP10/SOX8/HSP90AB1/ID3/IGF1/IL1RN/INHBA/IRF1/ITGB2/KDR/ARHGDIA/LGALS9/LMNA/MPZ/PAFAH2/PARK2/UTP11L/PKHD1/PKM/PLAGL1/PML/PPP2R2B/MAPK3/PROC/PAK6/PSMD7/PTGFR/PLEKHG5/MARK4/SCT/NOD2/BMP4/BMPR1B/BOK/STK3/TERF1/TGM2/TNFAIP3/TNFRSF1A/TRAF5/TNFRSF4/WNT10B/CARD14/BCL2L14/FAM188A/ZC3H12A/CPEB4/CLPTM1L/CALR/CAST/SCIN/TP63/RUNX3/IRS2/ACTN1/CRADD/ALDH1A2/SPHK1/MAP3K6/AURKB/DAPL1/ADIPOQ/RAPGEF2	87
GO:0044281	small molecule metabolic process	109/590	2375/17046	0.00106	0.04833	0.04192	GNE/MTHFS/PDPN/ERLIN2/EGLN2/ACOT7/CNP/NEU4/ADM/EGFLAM/CPS1/NDUFAF6/CRABP1/B3GLCT/PPM1L/MBOAT1/CYP11A1/DIO3/DLG2/ABAT/AGXT/ENO2/FAH/FHIT/AKR1B1/NUP210/SLC37A4/PNKD/ACOT11/GATM/PDE7B/AMPD3/DHDH/THEM5/GRB10/DNAJC15/GUCY1A3/PADI1/HAS1/HK1/ACACB/HRH1/HSD11B1/NME9/IGF1/IGF2/IL1RN/INPP5A/LDLR/LHCGR/MC2R/ME2/MGAT1/MOCS1/MYH4/NUBP1/NDUFB4/NEU1/ATP1A2/NPPC/OAS2/OPRL1/ATP5B/PARK2/SPOCK3/CHST15/PCYOX1/SIRT6/PDE6B/PKM/PLA2G2A/PRKAG3/SLCO1C1/PNLIP/CYP2W1/PPP1CB/PRKAR1B/LMBRD1/CSGALNACT1/APOBR/MAPK3/MRAP/PSMD7/RDH14/SCT/STRA6/SLC4A1/TNFRSF1A/CCR2/UCP1/CA7/CACNA1E/CERS4/QTRT1/NR0B2/RAE1/KMO/IRS2/ALDH1A2/SYNJ2/SPHK1/CH25H/SLC16A3/ADIPOQ/ENTPD3/NUP93/LPGAT1/FGF19/NR1H4	109
GO:0071320	cellular response to cAMP	7/590	47/17046	0.00108	0.04921	0.04269	CPS1/CYP11A1/AQP9/SLC8A1/WNT10B/ADIPOQ/RAPGEF2	7
GO:0001503	ossification	24/590	348/17046	0.0011	0.04965	0.04307	CCR1/FAM101A/SBNO2/FOXC2/SOX8/ID3/RSPO2/IGF1/IGF2/NPPC/ATP5B/CSGALNACT1/RDH14/BGLAP/BMP4/SLC8A1/BMPR1B/WNT10B/IFITM1/TP63/RUNX3/TNFRSF11A/PIAS2/RSAD2	24
**Hypomethylated DMC, Cellular Component**								
	Description	GeneRatio	BgRatio	pvalue	p.adjust	qvalue	geneID	Count

GO:0005575	cellular_component	617/617	16277/17046	2.49E-13	1.47E-10	1.37E-10	AKT3/ABI1/TANK/SMIM6/CD300LD/GNE/ZNF783/CDH9/CDH12/SUGP2/FARP1/KLRG1/RCAN2/CDKN1C/SPEG/MRVI1/TRDN/SPON2/COG5/PITRM1/TACC2/MTHFS/PDPN/CELF1/GJB6/PNRC1/TMED10/LECT1/RER1/ESM1/ADAM29/HNRNPUL1/RPP14/CHGA/CHI3L1/ERLIN2/CHI3L2/PKP3/EGLN2/ATXN2L/KIF12/ACOT7/EXOC3/CHRNA1/CHRNA2/GPRIN1/SORCS1/C1QTNF7/GBP4/PANX3/RBP7/GALNT15/AP3S1/C10orf90/FAT3/CLN5/MRPL52/CCR1/SLC51B/SPATA33/SEZ6/TNFAIP8L1/MOB3A/CNP/NEU4/COL9A3/COMP/MAP3K8/ADM/IL31RA/EGFLAM/UBLCP1/HUS1B/C7orf34/OR2A14/CPM/CPS1/NDUFAF6/PXDNL/CRABP1/ZNF358/MIB2/PARP4/MPP7/LDLRAD3/FAM101A/B3GLCT/CEP128/MGAT5B/CSTA/KLC3/ZNF738/ABCC13/XKR3/SMYD1/SGOL1/PPM1L/SH3D19/CYLD/MBOAT1/ANKRD46/ESCO2/CYP11A1/ZNF782/CALML6/DDB1/DDOST/ZNF366/BHLHA15/PPP1R18/DIO3/DLG2/DMBT1/DNAH6/ABAT/DPH1/DTNA/AGXT/EEF2/EFNA2/EIF4G1/A2M/ELK4/ANKRD23/LIPH/SLC10A4/SMIM14/ENO2/ADCK5/EPHA1/ESR1/F11/FAH/FAT2/SPATA13/PRSS54/FCGR2A/PHACTR1/FGA/FGF10/FHIT/BTBD3/SBNO2/TRAK1/MSRB2/FOXL1/FOXC2/EXPH5/AKR1B1/NFASC/EPB41L3/GGA3/DIP2A/FLOT2/MLC1/TBC1D1/RHOBTB2/NUP210/NEDD4L/SYNE1/PSD3/PPP1R13B/PUM2/ARHGEF18/RYBP/MORC3/MAPK8IP2/TSSK2/VGLL2/SLC37A4/RASGEF1C/RNF144B/ZNF549/CCDC110/ST6GALNAC3/TMEM151A/SAMM50/DFNB31/ALS2CL/PNKD/SEC31B/ACOT11/RAI14/STEAP2/GAS2/FBXL21/LCE2B/SACS/GATM/GBGT1/PLEK2/SLC17A5/ADGRF1/RPS6KC1/PABPC1/AKAP8L/DNAJC2/FGF22/NPTN/CLUL1/PDE7B/CYTH4/AMPD3/DHDH/BMP10/ZNF638/ZNF311/TMPRSS12/ZNF844/THEM5/GPR26/EOGT/FFAR2/TRIM42/GRB10/MRPS18B/GRIK4/DNAJC15/SCG3/TMOD4/GUCY1A3/GPR132/PADI1/HAS1/SOX8/HK1/HLA-DOA/HLA-DPA1/ANXA13/NR4A1/ACACB/HOXC4/AGFG2/HRH1/HSD11B1/HSPA1L/HSP90AB1/HTR3A/DUPD1/ADAMTSL5/ANKRD45/TFAP2E/ID3/ZC3H12D/RSPO2/CD300E/NME9/IGF1/IGF2/GPR142/LCE1C/LCE1D/LCE2D/IL1R1/IL1RN/IL10RA/AQP2/IL11RA/IL12RB2/IL15RA/PRSS41/IL16/FOXK2/AQP5/INHBA/INPP5A/IRF1/AQP9/ITGA7/ITGB2/ITGB7/ITIH4/IVL/JUP/CD82/USP50/KCNH2/KCNJ8/KCNJ9/KCNMB1/KDR/KIF25/IPO5/AMIGO3/CLEC17A/HES5/AFF3/LAIR2/LAMA3/STMN1/OR2A5/LCP1/MUC21/LDLR/ARHGDIA/LGALS9/LHCGR/C11orf87/LMNA/LMO2/RAB19/LPP/MC2R/MCC/ME2/MFNG/MGAT1/SCGB2A1/MITF/LHX8/ASGR1/MOCS1/MOV10/MPZ/IGFL4/MYH4/MYL2/NUBP1/NDUFB4/DRG1/NEU1/ATP1A2/NFATC3/NHLH2/NPPC/NRAS/OAS2/OPRL1/OR2C1/OR3A2/SLC22A18/P2RY6/PAFAH2/ATP5B/IL21R/ANO7/PARK2/SPOCK3/BOLA1/UTP11L/CHST15/PCYOX1/C11orf73/SIRT6/PDE6B/HIGD1B/ATP8A2/PI3/PITX2/PKHD1/PKM/PLA2G2A/PLAGL1/LRP1B/PRKAG3/PML/RIPPLY3/FXYD6/GPR84/SLCO1C1/PNLIP/TLR9/TREM1/SSH1/RIN2/POU2AF1/APBB1IP/MXRA8/FBLIM1/MED18/PALMD/CYP2W1/RPP25/BANP/PPP1CB/HERC6/ELP3/DNAJC17/GOLPH3L/PPP2R2B/FANCI/MOB1A/SLC47A1/SLC29A3/MIS18BP1/TRPV6/SLC30A10/CHRNA9/PEX26/FRMD4A/CARKD/PARVA/PRKAR1B/TTC17/IFT122/CFAP44/ERMARD/MCTP2/LMBRD1/CSGALNACT1/PAG1/CISD1/WSB2/MYNN/APOBR/MAPK3/MAP2K2/PCDHGB3/PRKRIR/PROC/MRAP/TRPV5/PRMT8/HTRA1/SLAMF8/CDC42SE1/PAK6/ARNTL2/RGMA/CEACAM19/PRDM11/PSMD7/SLURP1/PTGFR/PLEKHG5/TENM2/GATAD2B/ERMN/RNF150/KLHL8/RDH14/MARK4/PTPRE/CREBZF/FAM60A/ACTA2/RFC2/TRIM27/RGR/RGS12/RIT2/RPA3/BGLAP/SCT/CCL11/ABHD4/MRPS14/PRSS22/NPAS3/PARVG/NOD2/TINAGL1/STRA6/MAP1LC3B2/ARHGAP9/GZF1/DNAI2/CLDN25/PCDH20/TMEM237/VPS33A/BMP4/SLC4A1/SPATS2/ZNF649/ZG16/SLC6A12/SLC8A1/SLC9A3/SLC20A2/BMPR1B/SLIT1/BRD9/ZSCAN18/TMEM108/LYNX1/BOK/BPI/SRP68/STAT2/STK3/SUPT6H/BST2/TCEB2/ZEB1/TEAD3/TERF1/TGM2/TCHH/TNFAIP3/TNFRSF1A/TRAF5/TRPC6/TRPM2/CCR2/TNFRSF4/UCP1/WNT10B/ZAP70/ZNF7/CA7/CACNA1E/MOGS/RAB7A/ERI3/CCDC86/CARD14/ESRG/BCL2L14/LST1/CERS4/IGFLR1/ZNF665/CSPP1/ERMP1/CALD1/PSCA/FAM188A/TMEM62/GPR157/ZC3H12A/FAAP100/CPEB4/C6orf25/ZNF436/CLPTM1L/CALR/COL21A1/KRTAP2-1/QTRT1/SLIRP/CAST/CAPZB/CCDC3/DYNLRB2/SLC25A18/SPATA16/KRTAP17-1/ANTXR1/MFSD7/CMAHP/BFSP2/ATP13A4/YIPF4/ZNF397/NR0B2/MON1A/CASQ1/HOPX/PARD6G/FAXC/KRBA1/TTBK1/RETNLB/TRIM63/GTPBP3/RAE1/C1orf198/PPFIBP2/SLC43A1/IFITM1/ITPRIP/SCIN/KMO/RUNX1/TP63/RUNX3/SERPINA6/IRS2/ACTN1/CRADD/TNFRSF11A/ALDH1A2/STK19/SYNJ2/SPHK1/BUD31/CCNA1/ENDOU/SKAP2/STBD1/HSPB3/SLAMF9/TSPAN18/CH25H/CCRL2/ERI1/PRC1/STARD13/PIAS2/MAP3K6/SYT7/ESAM/SLC16A3/CBFA2T2/RSAD2/AURKB/CD8A/CCDC102A/TRIP10/ADIPOQ/ENTPD3/PREPL/RAB36/MICAL2/N4BP1/VGLL4/NUP93/RAPGEF2/CD79A/KIAA0513/DAZAP2/ZBTB39/IQSEC1/LPGAT1/FGF19/NR1H4	617
GO:0071944	cell periphery	234/617	4563/17046	4.69E-10	1.38E-07	1.29E-07	AKT3/ABI1/CD300LD/CDH9/CDH12/FARP1/KLRG1/TRDN/PDPN/GJB6/TMED10/ADAM29/ERLIN2/PKP3/EXOC3/CHRNA1/CHRNA2/GPRIN1/PANX3/FAT3/CCR1/SLC51B/SEZ6/CNP/IL31RA/OR2A14/CPM/MPP7/LDLRAD3/CSTA/XKR3/SH3D19/CYLD/DIO3/DLG2/DTNA/EEF2/EFNA2/LIPH/SLC10A4/ENO2/EPHA1/ESR1/F11/FAT2/SPATA13/FCGR2A/FGA/FGF10/NFASC/EPB41L3/FLOT2/MLC1/RHOBTB2/NEDD4L/SYNE1/PSD3/PPP1R13B/RAI14/STEAP2/LCE2B/PLEK2/SLC17A5/ADGRF1/NPTN/CYTH4/GPR26/FFAR2/GRB10/GRIK4/GUCY1A3/GPR132/HAS1/HLA-DOA/HLA-DPA1/ANXA13/HRH1/HSP90AB1/HTR3A/CD300E/IGF1/IGF2/GPR142/LCE1C/LCE1D/LCE2D/IL1R1/IL1RN/IL10RA/AQP2/IL11RA/IL12RB2/PRSS41/IL16/AQP5/INPP5A/AQP9/ITGA7/ITGB2/ITGB7/ITIH4/IVL/JUP/CD82/KCNH2/KCNJ8/KCNJ9/KCNMB1/KDR/OR2A5/LCP1/MUC21/LDLR/LHCGR/RAB19/LPP/MC2R/MCC/ASGR1/MPZ/NUBP1/NEU1/ATP1A2/NRAS/OPRL1/OR2C1/OR3A2/SLC22A18/P2RY6/ATP5B/ANO7/PCYOX1/PDE6B/ATP8A2/PKHD1/PKM/FXYD6/GPR84/SLCO1C1/TLR9/TREM1/SSH1/APBB1IP/FBLIM1/CYP2W1/FANCI/SLC47A1/TRPV6/SLC30A10/CHRNA9/PARVA/PRKAR1B/TTC17/LMBRD1/PAG1/APOBR/MAPK3/MAP2K2/PCDHGB3/MRAP/TRPV5/PRMT8/CDC42SE1/RGMA/PTGFR/PLEKHG5/TENM2/ERMN/PTPRE/TRIM27/RGR/RGS12/RIT2/PARVG/NOD2/STRA6/DNAI2/CLDN25/PCDH20/SLC4A1/SLC6A12/SLC8A1/SLC9A3/SLC20A2/BMPR1B/LYNX1/BPI/STAT2/BST2/TGM2/TNFRSF1A/TRAF5/TRPC6/TRPM2/CCR2/TNFRSF4/ZAP70/CACNA1E/CARD14/IGFLR1/CALD1/PSCA/GPR157/ZC3H12A/C6orf25/CALR/ANTXR1/BFSP2/ATP13A4/YIPF4/CASQ1/PARD6G/SLC43A1/IFITM1/ITPRIP/SCIN/IRS2/ACTN1/TNFRSF11A/SPHK1/ENDOU/SKAP2/STBD1/TSPAN18/CCRL2/PRC1/SYT7/ESAM/SLC16A3/CD8A/TRIP10/ENTPD3/RAPGEF2/CD79A	234

GO:0005886	plasma membrane	229/617	4464/17046	8.17E-10	1.60E-07	1.50E-07	AKT3/ABI1/CD300LD/CDH9/CDH12/FARP1/KLRG1/TRDN/PDPN/GJB6/TMED10/ADAM29/ERLIN2/PKP3/CHRNA1/CHRNA2/GPRIN1/PANX3/FAT3/CCR1/SLC51B/SEZ6/CNP/IL31RA/OR2A14/CPM/MPP7/LDLRAD3/CSTA/XKR3/SH3D19/CYLD/DIO3/DLG2/DTNA/EEF2/EFNA2/LIPH/SLC10A4/ENO2/EPHA1/ESR1/F11/FAT2/SPATA13/FCGR2A/FGA/FGF10/NFASC/EPB41L3/FLOT2/MLC1/RHOBTB2/NEDD4L/SYNE1/PSD3/PPP1R13B/STEAP2/LCE2B/PLEK2/SLC17A5/ADGRF1/NPTN/CYTH4/GPR26/FFAR2/GRB10/GRIK4/GUCY1A3/GPR132/HAS1/HLA-DOA/HLA-DPA1/ANXA13/HRH1/HSP90AB1/HTR3A/CD300E/IGF1/IGF2/GPR142/LCE1C/LCE1D/LCE2D/IL1R1/IL1RN/IL10RA/AQP2/IL11RA/IL12RB2/PRSS41/IL16/AQP5/INPP5A/AQP9/ITGA7/ITGB2/ITGB7/ITIH4/IVL/JUP/CD82/KCNH2/KCNJ8/KCNJ9/KCNMB1/KDR/OR2A5/LCP1/MUC21/LDLR/LHCGR/RAB19/LPP/MC2R/MCC/ASGR1/MPZ/NUBP1/NEU1/ATP1A2/NRAS/OPRL1/OR2C1/OR3A2/SLC22A18/P2RY6/ATP5B/ANO7/PCYOX1/PDE6B/ATP8A2/PKHD1/PKM/FXYD6/GPR84/SLCO1C1/TLR9/TREM1/SSH1/APBB1IP/CYP2W1/FANCI/SLC47A1/TRPV6/SLC30A10/CHRNA9/PARVA/PRKAR1B/TTC17/LMBRD1/PAG1/APOBR/MAPK3/MAP2K2/PCDHGB3/MRAP/TRPV5/PRMT8/CDC42SE1/RGMA/PTGFR/PLEKHG5/TENM2/PTPRE/TRIM27/RGR/RGS12/RIT2/PARVG/NOD2/STRA6/DNAI2/CLDN25/PCDH20/SLC4A1/SLC6A12/SLC8A1/SLC9A3/SLC20A2/BMPR1B/LYNX1/BPI/STAT2/BST2/TGM2/TNFRSF1A/TRAF5/TRPC6/TRPM2/CCR2/TNFRSF4/ZAP70/CACNA1E/CARD14/IGFLR1/CALD1/PSCA/GPR157/ZC3H12A/C6orf25/CALR/ANTXR1/BFSP2/ATP13A4/YIPF4/CASQ1/PARD6G/SLC43A1/IFITM1/ITPRIP/IRS2/ACTN1/TNFRSF11A/SPHK1/ENDOU/SKAP2/STBD1/TSPAN18/CCRL2/PRC1/SYT7/ESAM/SLC16A3/CD8A/TRIP10/ENTPD3/RAPGEF2/CD79A	229
GO:0005623	cell	570/617	14587/17046	9.05E-08	1.33E-05	1.25E-05	AKT3/ABI1/TANK/CD300LD/GNE/ZNF783/CDH9/CDH12/SUGP2/FARP1/KLRG1/RCAN2/CDKN1C/SPEG/MRVI1/TRDN/COG5/PITRM1/TACC2/MTHFS/PDPN/CELF1/GJB6/PNRC1/TMED10/LECT1/RER1/ADAM29/HNRNPUL1/RPP14/CHGA/CHI3L1/ERLIN2/PKP3/EGLN2/ATXN2L/KIF12/ACOT7/EXOC3/CHRNA1/CHRNA2/GPRIN1/GBP4/PANX3/RBP7/GALNT15/AP3S1/C10orf90/FAT3/CLN5/MRPL52/CCR1/SLC51B/SPATA33/SEZ6/TNFAIP8L1/MOB3A/CNP/NEU4/COL9A3/MAP3K8/ADM/IL31RA/UBLCP1/HUS1B/OR2A14/CPM/CPS1/NDUFAF6/PXDNL/CRABP1/ZNF358/MIB2/PARP4/MPP7/LDLRAD3/FAM101A/B3GLCT/CEP128/MGAT5B/CSTA/KLC3/ZNF738/XKR3/SMYD1/SGOL1/PPM1L/SH3D19/CYLD/MBOAT1/ESCO2/CYP11A1/ZNF782/CALML6/DDB1/DDOST/ZNF366/BHLHA15/PPP1R18/DIO3/DLG2/DMBT1/DNAH6/ABAT/DPH1/DTNA/AGXT/EEF2/EFNA2/EIF4G1/A2M/ELK4/ANKRD23/LIPH/SLC10A4/SMIM14/ENO2/ADCK5/EPHA1/ESR1/F11/FAH/FAT2/SPATA13/PRSS54/FCGR2A/PHACTR1/FGA/FGF10/FHIT/BTBD3/TRAK1/MSRB2/FOXL1/FOXC2/EXPH5/AKR1B1/NFASC/EPB41L3/GGA3/DIP2A/FLOT2/MLC1/TBC1D1/RHOBTB2/NUP210/NEDD4L/SYNE1/PSD3/PPP1R13B/PUM2/ARHGEF18/RYBP/MORC3/MAPK8IP2/TSSK2/VGLL2/SLC37A4/RASGEF1C/RNF144B/ZNF549/CCDC110/ST6GALNAC3/SAMM50/DFNB31/ALS2CL/PNKD/SEC31B/ACOT11/RAI14/STEAP2/GAS2/FBXL21/LCE2B/SACS/GATM/GBGT1/PLEK2/SLC17A5/ADGRF1/RPS6KC1/PABPC1/AKAP8L/DNAJC2/FGF22/NPTN/PDE7B/CYTH4/AMPD3/DHDH/BMP10/ZNF638/ZNF311/ZNF844/THEM5/GPR26/EOGT/FFAR2/TRIM42/GRB10/MRPS18B/GRIK4/DNAJC15/SCG3/TMOD4/GUCY1A3/GPR132/PADI1/HAS1/SOX8/HK1/HLA-DOA/HLA-DPA1/ANXA13/NR4A1/ACACB/HOXC4/HRH1/HSD11B1/HSPA1L/HSP90AB1/HTR3A/DUPD1/TFAP2E/ID3/ZC3H12D/RSPO2/CD300E/NME9/IGF1/IGF2/GPR142/LCE1C/LCE1D/LCE2D/IL1R1/IL1RN/IL10RA/AQP2/IL11RA/IL12RB2/IL15RA/PRSS41/IL16/FOXK2/AQP5/INHBA/INPP5A/IRF1/AQP9/ITGA7/ITGB2/ITGB7/ITIH4/IVL/JUP/CD82/USP50/KCNH2/KCNJ8/KCNJ9/KCNMB1/KDR/KIF25/IPO5/CLEC17A/HES5/AFF3/STMN1/OR2A5/LCP1/MUC21/LDLR/ARHGDIA/LGALS9/LHCGR/LMNA/LMO2/RAB19/LPP/MC2R/MCC/ME2/MFNG/MGAT1/MITF/LHX8/ASGR1/MOCS1/MOV10/MPZ/MYH4/MYL2/NUBP1/NDUFB4/DRG1/NEU1/ATP1A2/NFATC3/NHLH2/NPPC/NRAS/OAS2/OPRL1/OR2C1/OR3A2/SLC22A18/P2RY6/PAFAH2/ATP5B/ANO7/PARK2/BOLA1/UTP11L/CHST15/PCYOX1/C11orf73/SIRT6/PDE6B/ATP8A2/PITX2/PKHD1/PKM/PLA2G2A/PLAGL1/PRKAG3/PML/RIPPLY3/FXYD6/GPR84/SLCO1C1/TLR9/TREM1/SSH1/RIN2/POU2AF1/APBB1IP/MXRA8/FBLIM1/MED18/PALMD/CYP2W1/RPP25/BANP/PPP1CB/HERC6/ELP3/DNAJC17/GOLPH3L/PPP2R2B/FANCI/MOB1A/SLC47A1/SLC29A3/MIS18BP1/TRPV6/SLC30A10/CHRNA9/PEX26/FRMD4A/CARKD/PARVA/PRKAR1B/TTC17/IFT122/CFAP44/ERMARD/MCTP2/LMBRD1/CSGALNACT1/PAG1/CISD1/WSB2/MYNN/APOBR/MAPK3/MAP2K2/PCDHGB3/PRKRIR/PROC/MRAP/TRPV5/PRMT8/HTRA1/SLAMF8/CDC42SE1/PAK6/ARNTL2/RGMA/PRDM11/PSMD7/PTGFR/PLEKHG5/TENM2/GATAD2B/ERMN/KLHL8/RDH14/MARK4/PTPRE/CREBZF/FAM60A/ACTA2/RFC2/TRIM27/RGR/RGS12/RIT2/RPA3/BGLAP/CCL11/ABHD4/MRPS14/NPAS3/PARVG/NOD2/STRA6/MAP1LC3B2/ARHGAP9/GZF1/DNAI2/CLDN25/PCDH20/TMEM237/VPS33A/BMP4/SLC4A1/SPATS2/ZNF649/ZG16/SLC6A12/SLC8A1/SLC9A3/SLC20A2/BMPR1B/SLIT1/ZSCAN18/LYNX1/BOK/BPI/SRP68/STAT2/STK3/SUPT6H/BST2/TCEB2/ZEB1/TEAD3/TERF1/TGM2/TCHH/TNFAIP3/TNFRSF1A/TRAF5/TRPC6/TRPM2/CCR2/TNFRSF4/UCP1/ZAP70/ZNF7/CA7/CACNA1E/MOGS/RAB7A/ERI3/CCDC86/CARD14/ESRG/BCL2L14/LST1/CERS4/IGFLR1/ZNF665/CSPP1/ERMP1/CALD1/PSCA/FAM188A/GPR157/ZC3H12A/FAAP100/CPEB4/C6orf25/ZNF436/CALR/COL21A1/KRTAP2-1/QTRT1/SLIRP/CAST/CAPZB/CCDC3/DYNLRB2/SLC25A18/SPATA16/KRTAP17-1/ANTXR1/CMAHP/BFSP2/ATP13A4/YIPF4/ZNF397/NR0B2/MON1A/CASQ1/HOPX/PARD6G/TTBK1/TRIM63/GTPBP3/RAE1/C1orf198/PPFIBP2/SLC43A1/IFITM1/ITPRIP/SCIN/KMO/RUNX1/TP63/RUNX3/IRS2/ACTN1/CRADD/TNFRSF11A/ALDH1A2/STK19/SYNJ2/SPHK1/BUD31/CCNA1/ENDOU/SKAP2/STBD1/HSPB3/TSPAN18/CH25H/CCRL2/ERI1/PRC1/STARD13/PIAS2/MAP3K6/SYT7/ESAM/SLC16A3/CBFA2T2/RSAD2/AURKB/CD8A/CCDC102A/TRIP10/ADIPOQ/ENTPD3/PREPL/RAB36/MICAL2/N4BP1/VGLL4/NUP93/RAPGEF2/CD79A/KIAA0513/DAZAP2/ZBTB39/IQSEC1/LPGAT1/FGF19/NR1H4	570

GO:0044464	cell part	567/617	14556/17046	4.17E-07	4.91E-05	4.60E-05	AKT3/ABI1/TANK/CD300LD/GNE/ZNF783/CDH9/CDH12/SUGP2/FARP1/KLRG1/RCAN2/CDKN1C/SPEG/MRVI1/TRDN/COG5/PITRM1/TACC2/MTHFS/PDPN/CELF1/GJB6/PNRC1/TMED10/LECT1/RER1/ADAM29/HNRNPUL1/RPP14/CHGA/CHI3L1/ERLIN2/PKP3/EGLN2/ATXN2L/KIF12/ACOT7/EXOC3/CHRNA1/CHRNA2/GPRIN1/GBP4/PANX3/RBP7/GALNT15/AP3S1/C10orf90/FAT3/CLN5/MRPL52/CCR1/SLC51B/SPATA33/SEZ6/TNFAIP8L1/MOB3A/CNP/NEU4/COL9A3/MAP3K8/ADM/IL31RA/UBLCP1/HUS1B/OR2A14/CPM/CPS1/NDUFAF6/PXDNL/CRABP1/ZNF358/MIB2/PARP4/MPP7/LDLRAD3/FAM101A/B3GLCT/CEP128/MGAT5B/CSTA/KLC3/ZNF738/XKR3/SMYD1/SGOL1/PPM1L/SH3D19/CYLD/MBOAT1/ESCO2/CYP11A1/ZNF782/CALML6/DDB1/DDOST/ZNF366/BHLHA15/PPP1R18/DIO3/DLG2/DMBT1/DNAH6/ABAT/DPH1/DTNA/AGXT/EEF2/EFNA2/EIF4G1/A2M/ELK4/ANKRD23/LIPH/SLC10A4/SMIM14/ENO2/ADCK5/EPHA1/ESR1/F11/FAH/FAT2/SPATA13/PRSS54/FCGR2A/PHACTR1/FGA/FGF10/FHIT/BTBD3/TRAK1/MSRB2/FOXL1/FOXC2/EXPH5/AKR1B1/NFASC/EPB41L3/GGA3/DIP2A/FLOT2/MLC1/TBC1D1/RHOBTB2/NUP210/NEDD4L/SYNE1/PSD3/PPP1R13B/PUM2/ARHGEF18/RYBP/MORC3/MAPK8IP2/TSSK2/VGLL2/SLC37A4/RASGEF1C/RNF144B/ZNF549/CCDC110/ST6GALNAC3/SAMM50/DFNB31/ALS2CL/PNKD/SEC31B/ACOT11/RAI14/STEAP2/GAS2/FBXL21/LCE2B/SACS/GATM/GBGT1/PLEK2/SLC17A5/ADGRF1/RPS6KC1/PABPC1/AKAP8L/DNAJC2/FGF22/NPTN/PDE7B/CYTH4/AMPD3/DHDH/BMP10/ZNF638/ZNF311/ZNF844/THEM5/GPR26/EOGT/FFAR2/TRIM42/GRB10/MRPS18B/GRIK4/DNAJC15/SCG3/TMOD4/GUCY1A3/GPR132/PADI1/HAS1/SOX8/HK1/HLA-DOA/HLA-DPA1/ANXA13/NR4A1/ACACB/HOXC4/HRH1/HSD11B1/HSPA1L/HSP90AB1/HTR3A/DUPD1/TFAP2E/ID3/ZC3H12D/RSPO2/CD300E/NME9/IGF1/IGF2/GPR142/LCE1C/LCE1D/LCE2D/IL1R1/IL1RN/IL10RA/AQP2/IL11RA/IL12RB2/IL15RA/PRSS41/IL16/FOXK2/AQP5/INPP5A/IRF1/AQP9/ITGA7/ITGB2/ITGB7/ITIH4/IVL/JUP/CD82/USP50/KCNH2/KCNJ8/KCNJ9/KCNMB1/KDR/KIF25/IPO5/CLEC17A/HES5/AFF3/STMN1/OR2A5/LCP1/MUC21/LDLR/ARHGDIA/LGALS9/LHCGR/LMNA/LMO2/RAB19/LPP/MC2R/MCC/ME2/MFNG/MGAT1/MITF/LHX8/ASGR1/MOCS1/MOV10/MPZ/MYH4/MYL2/NUBP1/NDUFB4/DRG1/NEU1/ATP1A2/NFATC3/NHLH2/NPPC/NRAS/OAS2/OPRL1/OR2C1/OR3A2/SLC22A18/P2RY6/PAFAH2/ATP5B/ANO7/PARK2/BOLA1/UTP11L/CHST15/PCYOX1/C11orf73/SIRT6/PDE6B/ATP8A2/PITX2/PKHD1/PKM/PLA2G2A/PLAGL1/PRKAG3/PML/RIPPLY3/FXYD6/GPR84/SLCO1C1/TLR9/TREM1/SSH1/RIN2/POU2AF1/APBB1IP/MXRA8/FBLIM1/MED18/PALMD/CYP2W1/RPP25/BANP/PPP1CB/HERC6/ELP3/DNAJC17/GOLPH3L/PPP2R2B/FANCI/MOB1A/SLC47A1/SLC29A3/MIS18BP1/TRPV6/SLC30A10/CHRNA9/PEX26/FRMD4A/CARKD/PARVA/PRKAR1B/TTC17/IFT122/CFAP44/ERMARD/MCTP2/LMBRD1/CSGALNACT1/PAG1/CISD1/WSB2/MYNN/APOBR/MAPK3/MAP2K2/PCDHGB3/PRKRIR/PROC/MRAP/TRPV5/PRMT8/HTRA1/SLAMF8/CDC42SE1/PAK6/ARNTL2/RGMA/PRDM11/PSMD7/PTGFR/PLEKHG5/TENM2/GATAD2B/ERMN/KLHL8/RDH14/MARK4/PTPRE/CREBZF/FAM60A/ACTA2/RFC2/TRIM27/RGR/RGS12/RIT2/RPA3/BGLAP/CCL11/ABHD4/MRPS14/NPAS3/PARVG/NOD2/STRA6/MAP1LC3B2/ARHGAP9/GZF1/DNAI2/CLDN25/PCDH20/TMEM237/VPS33A/BMP4/SLC4A1/SPATS2/ZNF649/ZG16/SLC6A12/SLC8A1/SLC9A3/SLC20A2/BMPR1B/ZSCAN18/LYNX1/BOK/BPI/SRP68/STAT2/STK3/SUPT6H/BST2/TCEB2/ZEB1/TEAD3/TERF1/TGM2/TCHH/TNFAIP3/TNFRSF1A/TRAF5/TRPC6/TRPM2/CCR2/TNFRSF4/UCP1/ZAP70/ZNF7/CA7/CACNA1E/MOGS/RAB7A/ERI3/CDC86/CARD14/ESRG/BCL2L14/LST1/CERS4/IGFLR1/ZNF665/CSPP1/ERMP1/CALD1/PSCA/FAM188A/GPR157/ZC3H12A/FAAP100/CPEB4/C6orf25/ZNF436/CALR/COL21A1/KRTAP2-1/QTRT1/SLIRP/CAST/CAPZB/CCDC3/DYNLRB2/SLC25A18/SPATA16/KRTAP17-1/ANTXR1/CMAHP/BFSP2/ATP13A4/YIPF4/ZNF397/NR0B2/CASQ1/HOPX/PARD6G/TTBK1/TRIM63/GTPBP3/RAE1/C1orf198/PPFIBP2/SLC43A1/IFITM1/ITPRIP/SCIN/KMO/RUNX1/TP63/RUNX3/IRS2/ACTN1/CRADD/TNFRSF11A/ALDH1A2/STK19/SYNJ2/SPHK1/BUD31/CCNA1/ENDOU/SKAP2/STBD1/HSPB3/TSPAN18/CH25H/CCRL2/ERI1/PRC1/STARD13/PIAS2/MAP3K6/SYT7/ESAM/SLC16A3/CBFA2T2/RSAD2/AURKB/CD8A/CCDC102A/TRIP10/ADIPOQ/ENTPD3/PREPL/RAB36/MICAL2/N4BP1/VGLL4/NUP93/RAPGEF2/CD79A/KIAA0513/DAZAP2/ZBTB39/IQSEC1/LPGAT1/FGF19/NR1H4	567
GO:0016020	membrane	351/617	8177/17046	3.80E-06	0.00037	0.00035	AKT3/ABI1/SMIM6/CD300LD/CDH9/CDH12/FARP1/KLRG1/MRVI1/TRDN/COG5/PDPN/CELF1/GJB6/TMED10/LECT1/RER1/ADAM29/CHGA/ERLIN2/PKP3/ATXN2L/EXOC3/CHRNA1/CHRNA2/GPRIN1/SORCS1/PANX3/GALNT15/AP3S1/FAT3/CLN5/MRPL52/CCR1/SLC51B/SEZ6/CNP/NEU4/IL31RA/OR2A14/CPM/CPS1/NDUFAF6/PARP4/MPP7/LDLRAD3/B3GLCT/MGAT5B/CSTA/ABCC13/XKR3/PPM1L/SH3D19/CYLD/MBOAT1/ANKRD46/CYP11A1/DDOST/DIO3/DLG2/DMBT1/DTNA/EEF2/EFNA2/EIF4G1/LIPH/SLC10A4/SMIM14/ENO2/ADCK5/EPHA1/ESR1/F11/FAT2/SPATA13/FCGR2A/FGA/FGF10/NFASC/EPB41L3/GGA3/FLOT2/MLC1/RHOBTB2/NUP210/NEDD4L/SYNE1/PSD3/PPP1R13B/PUM2/SLC37A4/RNF144B/ST6GALNAC3/TMEM151A/SAMM50/PNKD/SEC31B/STEAP2/GAS2/LCE2B/GATM/GBGT1/PLEK2/SLC17A5/ADGRF1/RPS6KC1/PABPC1/DNAJC2/NPTN/CYTH4/TMPRSS12/GPR26/FFAR2/GRB10/MRPS18B/GRIK4/DNAJC15/SCG3/GUCY1A3/GPR132/HAS1/HK1/HLA-DOA/HLA-DPA1/ANXA13/NR4A1/ACACB/AGFG2/HRH1/HSD11B1/HSP90AB1/HTR3A/CD300E/IGF1/IGF2/GPR142/LCE1C/LCE1D/LCE2D/IL1R1/IL1RN/IL10RA/AQP2/IL11RA/IL12RB2/IL15RA/PRSS41/IL16/AQP5/INPP5A/AQP9/ITGA7/ITGB2/ITGB7/ITIH4/IVL/JUP/CD82/KCNH2/KCNJ8/KCNJ9/KCNMB1/KDR/IPO5/AMIGO3/CLEC17A/STMN1/OR2A5/LCP1/MUC21/LDLR/LHCGR/C11orf87/LMNA/RAB19/LPP/MC2R/MCC/MFNG/MGAT1/ASGR1/MPZ/NUBP1/NDUFB4/DRG1/NEU1/ATP1A2/NRAS/OAS2/OPRL1/OR2C1/OR3A2/SLC22A18/P2RY6/ATP5B/IL21R/ANO7/CHST15/PCYOX1/PDE6B/HIGD1B/ATP8A2/PKHD1/PKM/PLA2G2A/LRP1B/PML/FXYD6/GPR84/SLCO1C1/TLR9/TREM1/SSH1/APBB1IP/MXRA8/PALMD/CYP2W1/GOLPH3L/PPP2R2B/FANCI/SLC47A1/SLC29A3/TRPV6/SLC30A10/CHRNA9/PEX26/PARVA/PRKAR1B/TTC17/IFT122/ERMARD/MCTP2/LMBRD1/CSGALNACT1/PAG1/CISD1/APOBR/MAPK3/MAP2K2/PCDHGB3/MRAP/TRPV5/PRMT8/SLAMF8/CDC42SE1/RGMA/CEACAM19/PSMD7/PTGFR/PLEKHG5/TENM2/RNF150/RDH14/PTPRE/TRIM27/RGR/RGS12/RIT2/MRPS14/PARVG/NOD2/STRA6/MAP1LC3B2/DNAI2/CLDN25/PCDH20/TMEM237/VPS33A/SLC4A1/ZG16/SLC6A12/SLC8A1/SLC9A3/SLC20A2/BMPR1B/TMEM108/LYNX1/BOK/BPI/STAT2/BST2/TGM2/TNFRSF1A/TRAF5/TRPC6/TRPM2/CCR2/TNFRSF4/UCP1/ZAP70/CACNA1E/MOGS/RAB7A/CARD14/BCL2L14/LST1/CERS4/IGFLR1/ERMP1/CALD1/PSCA/FAM188A/TMEM62/GPR157/ZC3H12A/C6orf25/CLPTM1L/CALR/CAST/SLC25A18/ANTXR1/MFSD7/CMAHP/BFSP2/ATP13A4/YIPF4/CASQ1/PARD6G/FAXC/RAE1/SLC43A1/IFITM1/ITPRIP/KMO/IRS2/ACTN1/TNFRSF11A/SYNJ2/SPHK1/ENDOU/SKAP2/STBD1/SLAMF9/TSPAN18/CH25H/CCRL2/PRC1/STARD13/SYT7/ESAM/SLC16A3/RSAD2/CD8A/TRIP10/ENTPD3/RAB36/NUP93/RAPGEF2/CD79A/IQSEC1/LPGAT1	351

GO:0005737	cytoplasm	396/617	9735/17046	0.00016	0.01333	0.01249	AKT3/ABI1/TANK/GNE/FARP1/RCAN2/CDKN1C/MRVI1/TRDN/COG5/PITRM1/TACC2/MTHFS/CELF1/GJB6/TMED10/RER1/CHGA/CHI3L1/ERLIN2/EGLN2/ATXN2L/KIF12/ACOT7/EXOC3/GBP4/RBP7/GALNT15/AP3S1/C10orf90/CLN5/MRPL52/CCR1/SPATA33/SEZ6/TNFAIP8L1/CNP/NEU4/COL9A3/MAP3K8/ADM/CPS1/NDUFAF6/PXDNL/CRABP1/MIB2/PARP4/MPP7/FAM101A/B3GLCT/CEP128/MGAT5B/CSTA/KLC3/SMYD1/SGOL1/PPM1L/SH3D19/CYLD/MBOAT1/ESCO2/CYP11A1/CALML6/DDB1/DDOST/PPP1R18/DIO3/DMBT1/DNAH6/ABAT/DPH1/DTNA/AGXT/EEF2/EIF4G1/A2M/ELK4/ANKRD23/SMIM14/ENO2/ADCK5/ESR1/FAH/SPATA13/PRSS54/PHACTR1/FGA/FHIT/BTBD3/TRAK1/MSRB2/EXPH5/AKR1B1/EPB41L3/GGA3/FLOT2/MLC1/TBC1D1/RHOBTB2/NUP210/NEDD4L/SYNE1/PPP1R13B/PUM2/ARHGEF18/RYBP/MAPK8IP2/TSSK2/VGLL2/SLC37A4/RNF144B/CCDC110/ST6GALNAC3/SAMM50/DFNB31/ALS2CL/PNKD/SEC31B/ACOT11/RAI14/STEAP2/GAS2/FBXL21/LCE2B/SACS/GATM/GBGT1/PLEK2/SLC17A5/RPS6KC1/PABPC1/AKAP8L/DNAJC2/FGF22/NPTN/PDE7B/AMPD3/DHDH/BMP10/ZNF638/THEM5/EOGT/GRB10/MRPS18B/DNAJC15/SCG3/TMOD4/GUCY1A3/PADI1/HAS1/SOX8/HK1/HLA-DOA/HLA-DPA1/ANXA13/NR4A1/ACACB/HRH1/HSD11B1/HSPA1L/HSP90AB1/HTR3A/DUPD1/ID3/ZC3H12D/NME9/IGF1/IGF2/GPR142/LCE1C/LCE1D/LCE2D/IL1RN/AQP2/IL15RA/IL16/AQP5/IRF1/ITGA7/ITIH4/IVL/JUP/KCNH2/KCNJ8/KDR/KIF25/IPO5/AFF3/STMN1/LCP1/MUC21/LDLR/ARHGDIA/LGALS9/LHCGR/LMNA/RAB19/LPP/MC2R/MCC/ME2/MFNG/MGAT1/MITF/MOCS1/MOV10/MPZ/MYH4/MYL2/NUBP1/NDUFB4/DRG1/NEU1/ATP1A2/NFATC3/NPPC/NRAS/OAS2/OPRL1/OR2C1/SLC22A18/PAFAH2/ATP5B/ANO7/PARK2/BOLA1/UTP11L/CHST15/PCYOX1/C11orf73/SIRT6/PDE6B/ATP8A2/PITX2/PKHD1/PKM/PLA2G2A/PLAGL1/PRKAG3/PML/TLR9/SSH1/RIN2/APBB1IP/FBLIM1/PALMD/CYP2W1/PPP1CB/HERC6/ELP3/DNAJC17/GOLPH3L/PPP2R2B/FANCI/MOB1A/SLC29A3/CHRNA9/PEX26/FRMD4A/CARKD/PARVA/PRKAR1B/TTC17/IFT122/ERMARD/LMBRD1/CSGALNACT1/CISD1/MAPK3/MAP2K2/PROC/MRAP/PRMT8/HTRA1/CDC42SE1/PAK6/ARNTL2/RGMA/PRDM11/PSMD7/PTGFR/PLEKHG5/TENM2/ERMN/RDH14/MARK4/PTPRE/ACTA2/RFC2/TRIM27/RGS12/BGLAP/MRPS14/NPAS3/PARVG/NOD2/MAP1LC3B2/ARHGAP9/GZF1/DNAI2/VPS33A/BMP4/SLC4A1/SPATS2/ZG16/SLC8A1/BOK/BPI/SRP68/STAT2/STK3/BST2/TCEB2/ZEB1/TERF1/TGM2/TNFAIP3/TNFRSF1A/TRAF5/TRPC6/CCR2/UCP1/ZAP70/CA7/MOGS/RAB7A/ERI3/CARD14/BCL2L14/LST1/CERS4/CSPP1/ERMP1/CALD1/ZC3H12A/FAAP100/CPEB4/C6orf25/ZNF436/CALR/COL21A1/QTRT1/SLIRP/CAST/CAPZB/CCDC3/DYNLRB2/SLC25A18/SPATA16/ANTXR1/CMAHP/BFSP2/ATP13A4/YIPF4/ZNF397/NR0B2/CASQ1/PARD6G/TTBK1/TRIM63/GTPBP3/RAE1/C1orf198/SCIN/KMO/RUNX1/TP63/RUNX3/IRS2/ACTN1/CRADD/ALDH1A2/SYNJ2/SPHK1/CCNA1/ENDOU/SKAP2/STBD1/HSPB3/CH25H/ERI1/PRC1/STARD13/MAP3K6/SYT7/RSAD2/AURKB/TRIP10/ADIPOQ/PREPL/RAB36/RAPGEF2/CD79A/KIAA0513/DAZAP2/LPGAT1	396
GO:0005615	extracellular space	67/617	1213/17046	0.00034	0.02516	0.02356	SPON2/CHI3L1/CHI3L2/C1QTNF7/SEZ6/CNP/COMP/ADM/PXDNL/CSTA/DDB1/DMBT1/A2M/LIPH/ENO2/F11/FGA/FGF10/AKR1B1/FGF22/BMP10/ANXA13/HSPA1L/RSPO2/IGF1/IGF2/IL1R1/IL1RN/IL15RA/IL16/INHBA/ITIH4/LCP1/LDLR/LGALS9/MFNG/SCGB2A1/MOV10/IGFL4/NPPC/SPOCK3/UTP11L/PCYOX1/PLA2G2A/PRKAG3/APOBR/PROC/HTRA1/SLURP1/ACTA2/BGLAP/SCT/CCL11/TINAGL1/BMP4/SLC4A1/ZNF649/SLIT1/BPI/TNFRSF1A/WNT10B/CALR/PPFIBP2/SERPINA6/ACTN1/ENDOU/ADIPOQ	67
GO:0044459	plasma membrane part	115/617	2392/17046	0.00073	0.04762	0.0446	ABI1/FARP1/TRDN/PDPN/GJB6/TMED10/ADAM29/CHRNA1/CHRNA2/CCR1/MPP7/CYLD/DLG2/DTNA/SLC10A4/EPHA1/SPATA13/FGA/EPB41L3/FLOT2/MLC1/SYNE1/PSD3/STEAP2/PLEK2/SLC17A5/NPTN/GPR26/FFAR2/GRIK4/GPR132/HAS1/HLA-DOA/HLA-DPA1/ANXA13/HRH1/HSP90AB1/HTR3A/GPR142/IL1R1/AQP2/IL11RA/IL12RB2/AQP5/AQP9/ITGA7/ITGB2/ITGB7/JUP/CD82/KCNH2/KCNJ8/KCNJ9/KCNMB1/KDR/LCP1/LDLR/LHCGR/MC2R/ASGR1/MPZ/ATP1A2/OPRL1/OR3A2/SLC22A18/P2RY6/PDE6B/PKHD1/FXYD6/GPR84/SLCO1C1/TLR9/APBB1IP/TRPV6/CHRNA9/MAPK3/MAP2K2/TRPV5/PTGFR/TENM2/TRIM27/RGR/NOD2/DNAI2/SLC4A1/SLC6A12/SLC8A1/SLC9A3/SLC20A2/BMPR1B/BPI/BST2/TGM2/TNFRSF1A/TRAF5/TRPC6/TRPM2/CCR2/TNFRSF4/ZAP70/CACNA1E/CALR/ANTXR1/ATP13A4/CASQ1/SLC43A1/TNFRSF11A/STBD1/TSPAN18/CCRL2/SLC16A3/CD8A/TRIP10/RAPGEF2/CD79A	115
**Hypomethylated DMC, Molecular Function**								
	Description	GeneRatio	BgRatio	pvalue	p.adjust	qvalue	geneID	Count

GO:0003674	molecular_function	581/581	15274/17046	6.22E-29	5.40E-26	5.05E-26	AKT3/ABI1/TANK/CD300LD/GNE/ZNF783/CDH9/CDH12/SUGP2/FARP1/KLRG1/RCAN2/CDKN1C/SPEG/MRVI1/TRDN/SPON2/COG5/PITRM1/TACC2/MTHFS/CELF1/RFPL2/PNRC1/TMED10/RER1/ESM1/ADAM29/HNRNPUL1/RPP14/CHI3L1/ERLIN2/CHI3L2/PKP3/EGLN2/TP53TG1/ATXN2L/KIF12/ACOT7/PDAP1/EXOC3/CHRNA1/CHRNA2/CARD16/GPRIN1/SORCS1/GBP4/PANX3/RBP7/GALNT15/AP3S1/FAT3/CLN5/MRPL52/CCR1/SLC51B/TNFAIP8L1/MOB3A/CNP/AADACL3/LRRIQ3/NEU4/COL9A3/COMP/MAP3K8/ADM/IL31RA/EGFLAM/UBLCP1/OR2A14/CPM/CPS1/NDUFAF6/PXDNL/CRABP1/ZNF358/CRYBB3/MIB2/PARP4/MPP7/LDLRAD3/FAM101A/B3GLCT/MGAT5B/CSTA/KLC3/ZNF738/ABCC13/SMYD1/SGOL1/PPM1L/SH3D19/CYLD/MBOAT1/ESCO2/CYP11A1/ZNF782/CALML6/DDB1/LONRF2/DDOST/ZNF366/BHLHA15/PPP1R18/DIO3/DLG2/DMBT1/DNAH6/ABAT/DPH1/DTNA/AGXT/EEF2/EFNA2/EIF4G1/A2M/ELK4/ANKRD23/LIPH/SLC10A4/ENO2/ADCK5/EPHA1/ESR1/F11/FAH/FAT2/SPATA13/PRSS54/FCGR2A/PHACTR1/FGA/FGF10/FHIT/TRAK1/MSRB2/FOXL1/FOXC2/EXPH5/AKR1B1/NFASC/EPB41L3/GGA3/DIP2A/FLOT2/MLC1/TBC1D1/RHOBTB2/NUP210/NEDD4L/SYNE1/PSD3/PPP1R13B/PUM2/ARHGEF18/RYBP/MORC3/MAPK8IP2/TSSK2/VGLL2/TTLL10/SLC37A4/RASGEF1C/RNF144B/ZNF549/CCDC110/ST6GALNAC3/SAMM50/DFNB31/ALS2CL/PNKD/ACOT11/STEAP2/FBXL21/LCE2B/SACS/GATM/GBGT1/SLC17A5/ADGRF1/RPS6KC1/PABPC1/AKAP8L/DNAJC2/FGF22/NPTN/PDE7B/CYTH4/AMPD3/DHDH/BMP10/ZNF638/C11orf31/ZNF311/TMPRSS12/ZNF844/THEM5/GPR26/EOGT/FFAR2/TRIM42/GRB10/MRPS18B/GRIK4/DNAJC15/SCG3/TMOD4/GUCY1A3/GPR132/PADI1/HAS1/SOX8/HK1/HLA-DOA/HLA-DPA1/ANXA13/NR4A1/ACACB/HOXC4/AGFG2/HRH1/HSD11B1/HSPA1L/HSP90AB1/HTR3A/DUPD1/ADAMTSL5/ANKRD45/TFAP2E/ID3/ZC3H12D/RSPO2/NME9/IGF1/IGF2/GPR142/LCE1C/LCE1D/LCE2D/IL1R1/IL1RN/IL10RA/AQP2/IL11RA/IL12RB2/IL15RA/PRSS41/IL16/FOXK2/AQP5/INHBA/INPP5A/IRF1/AQP9/ITGA7/ITGB2/ITGB7/ITIH4/IVL/JUP/CD82/USP50/KCNH2/KCNJ8/KCNJ9/KCNMB1/KDR/KIF25/IPO5/C17orf82/CLEC17A/HES5/RBM12B/AFF3/LAIR2/LAMA3/STMN1/OR2A5/LCP1/LDLR/ARHGDIA/LGALS9/LHCGR/C11orf87/LMNA/LMO2/RAB19/LPP/MC2R/MCC/ME2/MFNG/MGAT1/SCGB2A1/MITF/LHX8/ASGR1/MOCS1/MOV10/MPZ/PLEKHG7/MYH4/MYL2/NUBP1/NDUFB4/DRG1/NEU1/ATP1A2/NFATC3/NHLH2/NPPC/NRAS/OAS2/RNF165/OPRL1/OR2C1/OR3A2/SLC22A18/P2RY6/PAFAH2/ATP5B/IL21R/ANO7/PARK2/SPOCK3/BOLA1/UTP11L/PRR16/CHST15/PCYOX1/C11orf73/SIRT6/PDE6B/ATP8A2/PI3/PITX2/PKHD1/PKM/PLA2G2A/PLAGL1/LRP1B/PRKAG3/PML/FXYD6/GPR84/SLCO1C1/PNLIP/TLR9/TREM1/SSH1/RIN2/POU2AF1/APBB1IP/MXRA8/FBLIM1/MED18/CYP2W1/RPP25/TTC12/BANP/PPP1CB/HERC6/ELP3/DNAJC17/GOLPH3L/PPP2R2B/FANCI/MOB1A/SLC47A1/SLC29A3/MIS18BP1/TRPV6/SLC30A10/CHRNA9/THUMPD1/PEX26/FRMD4A/CARKD/PARVA/PRKAR1B/TTC17/IFT122/MCTP2/LMBRD1/CSGALNACT1/PAG1/CISD1/MYNN/APOBR/MAPK3/MAP2K2/PCDHGB3/PRKRIR/PROC/MRAP/TRPV5/PRMT8/HTRA1/SLAMF8/CDC42SE1/PAK6/ARNTL2/RGMA/PRDM11/PSMD7/SLURP1/PTGFR/PLEKHG5/TENM2/GATAD2B/ERMN/RNF150/RDH14/MARK4/PTPRE/CREBZF/ABHD17C/FAM60A/ACTA2/RFC2/TRIM27/RGR/RGS12/RIT2/RPA3/BGLAP/SCT/CCL11/ABHD4/MRPS14/PRSS22/NPAS3/PARVG/NOD2/TINAGL1/ARHGAP9/GZF1/DNAI2/CLDN25/PCDH20/VPS33A/BMP4/SLC4A1/SPATS2/ZNF649/ZG16/SLC6A12/SLC8A1/SLC9A3/SLC20A2/BMPR1B/SLIT1/BRD9/ZSCAN18/TMEM108/BOK/BPI/SRP68/STAT2/STK3/SUPT6H/BST2/TCEB2/ZEB1/TEAD3/TERF1/TGM2/TCHH/TNFAIP3/TNFRSF1A/TRAF5/TRPC6/TRPM2/CCR2/TNFRSF4/UCP1/WNT10B/ZAP70/ZNF7/CA7/CACNA1E/MOGS/RAB7A/ERI3/CCDC86/CARD14/BCL2L14/CERS4/IGFLR1/ZNF665/CSPP1/ERMP1/CALD1/FAM188A/TMEM62/GPR157/ZC3H12A/FAAP100/CPEB4/C6orf25/ZNF436/EEPD1/CALR/QTRT1/SLIRP/CAST/CAPZB/DYNLRB2/SLC25A18/ANTXR1/BFSP2/ATP13A4/YIPF4/ZNF397/NR0B2/CASQ1/HOPX/PARD6G/KRBA1/TTBK1/RETNLB/TRIM63/GTPBP3/RAE1/PPFIBP2/SLC43A1/IFITM1/SCIN/CDK10/KMO/RUNX1/TP63/RUNX3/SERPINA6/IRS2/ACTN1/CRADD/TNFRSF11A/ALDH1A2/STK19/SYNJ2/SPHK1/BUD31/CCNA1/ENDOU/SKAP2/STBD1/SLAMF9/CH25H/CCRL2/ERI1/PRC1/STARD13/PIAS2/MAP3K6/SYT7/SLC16A3/CBFA2T2/RSAD2/AURKB/DAPL1/CD8A/CCDC102A/NEURL3/TRIP10/ADIPOQ/ENTPD3/PREPL/RAB36/MICAL2/N4BP1/VGLL4/NUP93/RAPGEF2/CD79A/DAZAP2/ZBTB39/IQSEC1/LPGAT1/FGF19/NR1H4	581
GO:0005488	binding	489/581	12915/17046	3.40E-07	0.00015	0.00014	AKT3/ABI1/TANK/CD300LD/GNE/ZNF783/CDH9/CDH12/SUGP2/FARP1/KLRG1/RCAN2/CDKN1C/SPEG/MRVI1/TRDN/SPON2/COG5/PITRM1/TACC2/MTHFS/CELF1/RFPL2/PNRC1/TMED10/RER1/ESM1/ADAM29/HNRNPUL1/RPP14/CHI3L1/ERLIN2/CHI3L2/PKP3/EGLN2/ATXN2L/KIF12/ACOT7/PDAP1/EXOC3/CHRNA1/CHRNA2/GPRIN1/SORCS1/GBP4/RBP7/GALNT15/AP3S1/FAT3/CLN5/CCR1/SLC51B/TNFAIP8L1/MOB3A/CNP/LRRIQ3/NEU4/COMP/MAP3K8/ADM/IL31RA/EGFLAM/UBLCP1/CPM/CPS1/PXDNL/CRABP1/ZNF358/CRYBB3/MIB2/PARP4/MPP7/LDLRAD3/FAM101A/MGAT5B/CSTA/KLC3/ZNF738/ABCC13/SMYD1/SGOL1/PPM1L/SH3D19/CYLD/ESCO2/CYP11A1/ZNF782/CALML6/DDB1/LONRF2/DDOST/ZNF366/BHLHA15/PPP1R18/DLG2/DMBT1/DNAH6/ABAT/DPH1/DTNA/AGXT/EEF2/EFNA2/EIF4G1/A2M/ELK4/ANKRD23/LIPH/ENO2/ADCK5/EPHA1/ESR1/F11/FAH/FAT2/SPATA13/FCGR2A/PHACTR1/FGA/FGF10/FHIT/TRAK1/MSRB2/FOXL1/FOXC2/EXPH5/NFASC/EPB41L3/GGA3/DIP2A/FLOT2/MLC1/TBC1D1/RHOBTB2/NUP210/NEDD4L/SYNE1/PPP1R13B/PUM2/RYBP/MORC3/MAPK8IP2/TSSK2/VGLL2/TTLL10/RNF144B/ZNF549/CCDC110/SAMM50/DFNB31/ALS2CL/PNKD/ACOT11/STEAP2/SACS/GBGT1/RPS6KC1/PABPC1/AKAP8L/DNAJC2/FGF22/NPTN/PDE7B/CYTH4/AMPD3/BMP10/ZNF638/C11orf31/ZNF311/ZNF844/THEM5/FFAR2/TRIM42/GRB10/DNAJC15/SCG3/TMOD4/GUCY1A3/PADI1/SOX8/HK1/HLA-DOA/HLA-DPA1/ANXA13/NR4A1/ACACB/HOXC4/AGFG2/HRH1/HSPA1L/HSP90AB1/HTR3A/ADAMTSL5/ANKRD45/TFAP2E/ID3/ZC3H12D/RSPO2/IGF1/IGF2/LCE2D/IL1R1/IL1RN/IL10RA/IL12RB2/IL15RA/IL16/FOXK2/AQP5/INHBA/INPP5A/IRF1/ITGA7/ITGB2/ITGB7/ITIH4/IVL/JUP/CD82/KCNH2/KCNJ8/KCNJ9/KDR/KIF25/IPO5/C17orf82/CLEC17A/HES5/RBM12B/AFF3/LAIR2/LAMA3/STMN1/LCP1/LDLR/ARHGDIA/LGALS9/LHCGR/C11orf87/LMNA/LMO2/RAB19/LPP/MC2R/MCC/ME2/MFNG/MGAT1/SCGB2A1/MITF/LHX8/ASGR1/MOCS1/MOV10/MYH4/MYL2/NUBP1/DRG1/ATP1A2/NFATC3/NHLH2/NPPC/NRAS/OAS2/RNF165/OPRL1/SLC22A18/P2RY6/PAFAH2/ATP5B/PARK2/SPOCK3/BOLA1/UTP11L/PRR16/CHST15/C11orf73/SIRT6/PDE6B/ATP8A2/PITX2/PKHD1/PKM/PLA2G2A/PLAGL1/LRP1B/PRKAG3/PML/FXYD6/PNLIP/TLR9/TREM1/SSH1/POU2AF1/APBB1IP/FBLIM1/MED18/CYP2W1/RPP25/TTC12/BANP/PPP1CB/ELP3/DNAJC17/GOLPH3L/PPP2R2B/FANCI/MOB1A/MIS18BP1/TRPV6/CHRNA9/THUMPD1/PEX26/FRMD4A/CARKD/PARVA/PRKAR1B/TTC17/IFT122/MCTP2/LMBRD1/CSGALNACT1/PAG1/CISD1/MYNN/MAPK3/MAP2K2/PCDHGB3/PRKRIR/PROC/MRAP/TRPV5/PRMT8/HTRA1/PAK6/ARNTL2/RGMA/PSMD7/SLURP1/TENM2/GATAD2B/ERMN/RNF150/MARK4/PTPRE/CREBZF/FAM60A/ACTA2/RFC2/TRIM27/RGR/RIT2/RPA3/BGLAP/SCT/CCL11/MRPS14/NPAS3/PARVG/NOD2/TINAGL1/ARHGAP9/GZF1/DNAI2/PCDH20/VPS33A/BMP4/SLC4A1/SPATS2/ZNF649/ZG16/SLC6A12/SLC8A1/SLC9A3/BMPR1B/SLIT1/BRD9/ZSCAN18/BOK/BPI/SRP68/STAT2/STK3/SUPT6H/BST2/TCEB2/ZEB1/TEAD3/TERF1/TGM2/TCHH/TNFAIP3/TNFRSF1A/TRAF5/TRPC6/CCR2/WNT10B/ZAP70/ZNF7/CA7/CACNA1E/RAB7A/ERI3/CCDC86/CARD14/BCL2L14/CERS4/IGFLR1/ZNF665/CSPP1/ERMP1/CALD1/FAM188A/ZC3H12A/FAAP100/CPEB4/C6orf25/ZNF436/EEPD1/CALR/QTRT1/SLIRP/CAST/CAPZB/SLC25A18/ANTXR1/BFSP2/ATP13A4/YIPF4/ZNF397/NR0B2/CASQ1/HOPX/PARD6G/TTBK1/RETNLB/TRIM63/GTPBP3/RAE1/IFITM1/SCIN/CDK10/KMO/RUNX1/TP63/RUNX3/SERPINA6/IRS2/ACTN1/CRADD/TNFRSF11A/ALDH1A2/STK19/SYNJ2/SPHK1/BUD31/CCNA1/ENDOU/SKAP2/STBD1/CH25H/CCRL2/ERI1/PRC1/STARD13/PIAS2/MAP3K6/SYT7/SLC16A3/CBFA2T2/RSAD2/AURKB/DAPL1/CD8A/NEURL3/TRIP10/ADIPOQ/ENTPD3/RAB36/MICAL2/N4BP1/VGLL4/RAPGEF2/CD79A/DAZAP2/ZBTB39/IQSEC1/FGF19/NR1H4	489

GO:0060089	molecular transducer activity	90/581	1631/17046	2.80E-06	0.00081	0.00076	KLRG1/CHRNA1/CHRNA2/SORCS1/CCR1/MAP3K8/IL31RA/OR2A14/MIB2/DMBT1/EPHA1/ESR1/ADGRF1/NPTN/GPR26/FFAR2/GRIK4/GUCY1A3/GPR132/HLA-DOA/HLA-DPA1/NR4A1/HRH1/HTR3A/GPR142/IL1R1/IL10RA/IL11RA/IL12RB2/IL15RA/ITGB2/ITGB7/JUP/KCNH2/KDR/STMN1/OR2A5/LDLR/LGALS9/LHCGR/MC2R/MCC/ASGR1/OPRL1/OR2C1/OR3A2/P2RY6/IL21R/PKHD1/LRP1B/GPR84/TLR9/TREM1/CHRNA9/APOBR/MAPK3/SLAMF8/RGMA/PTGFR/PLEKHG5/PTPRE/TRIM27/RGR/RGS12/TINAGL1/SLC20A2/BMPR1B/STAT2/STK3/BST2/TNFRSF1A/TRAF5/CCR2/TNFRSF4/GPR157/ANTXR1/NR0B2/TRIM63/IFITM1/IRS2/TNFRSF11A/SPHK1/ENDOU/SLAMF9/CCRL2/MAP3K6/CD8A/RAPGEF2/CD79A/NR1H4	90
GO:0004872	receptor activity	73/581	1364/17046	7.40E-05	0.01606	0.01502	KLRG1/CHRNA1/CHRNA2/SORCS1/CCR1/IL31RA/OR2A14/DMBT1/EPHA1/ESR1/ADGRF1/NPTN/GPR26/FFAR2/GRIK4/GUCY1A3/GPR132/HLA-DOA/HLA-DPA1/NR4A1/HRH1/HTR3A/GPR142/IL1R1/IL10RA/IL11RA/IL12RB2/IL15RA/ITGB2/ITGB7/KCNH2/KDR/OR2A5/LDLR/LHCGR/MC2R/MCC/ASGR1/OPRL1/OR2C1/OR3A2/P2RY6/IL21R/PKHD1/LRP1B/GPR84/TLR9/TREM1/CHRNA9/APOBR/SLAMF8/RGMA/PTGFR/PTPRE/TRIM27/RGR/TINAGL1/SLC20A2/BMPR1B/TNFRSF1A/CCR2/TNFRSF4/GPR157/ANTXR1/NR0B2/TNFRSF11A/SPHK1/ENDOU/SLAMF9/CCRL2/CD8A/CD79A/NR1H4	73
GO:0004871	signal transducer activity	75/581	1444/17046	0.00015	0.02624	0.02453	CHRNA1/CHRNA2/SORCS1/CCR1/MAP3K8/IL31RA/OR2A14/MIB2/DMBT1/EPHA1/ESR1/ADGRF1/NPTN/GPR26/FFAR2/GRIK4/GPR132/HLA-DOA/HLA-DPA1/NR4A1/HRH1/HTR3A/GPR142/IL1R1/IL10RA/IL11RA/IL12RB2/IL15RA/ITGB2/JUP/KCNH2/KDR/STMN1/OR2A5/LGALS9/LHCGR/MC2R/OPRL1/OR2C1/OR3A2/P2RY6/IL21R/GPR84/TLR9/CHRNA9/MAPK3/RGMA/PTGFR/PLEKHG5/PTPRE/TRIM27/RGR/RGS12/BMPR1B/STAT2/STK3/BST2/TNFRSF1A/TRAF5/CCR2/TNFRSF4/GPR157/ANTXR1/NR0B2/TRIM63/IFITM1/IRS2/TNFRSF11A/SPHK1/CCRL2/MAP3K6/CD8A/RAPGEF2/CD79A/NR1H4	75
GO:0004896	cytokine receptor activity	10/581	80/17046	0.00038	0.04963	0.04641	CCR1/IL31RA/IL1R1/IL10RA/IL11RA/IL12RB2/IL15RA/IL21R/CCR2/CCRL2	10
GO:0005515	protein binding	372/581	9755/17046	0.0004	0.04963	0.04641	AKT3/ABI1/TANK/CD300LD/GNE/ZNF783/FARP1/KLRG1/CDKN1C/SPEG/MRVI1/TRDN/SPON2/COG5/TACC2/CELF1/PNRC1/TMED10/RER1/ESM1/HNRNPUL1/ERLIN2/PKP3/EGLN2/ATXN2L/KIF12/ACOT7/EXOC3/GPRIN1/SORCS1/RBP7/AP3S1/CLN5/CCR1/SLC51B/TNFAIP8L1/LRRIQ3/NEU4/COMP/MAP3K8/ADM/IL31RA/UBLCP1/CPS1/CRABP1/CRYBB3/MIB2/PARP4/MPP7/FAM101A/MGAT5B/CSTA/KLC3/SMYD1/SGOL1/SH3D19/CYLD/DDB1/DDOST/ZNF366/BHLHA15/PPP1R18/DLG2/DMBT1/ABAT/DPH1/DTNA/AGXT/EEF2/EFNA2/EIF4G1/A2M/ELK4/ANKRD23/ADCK5/EPHA1/ESR1/F11/FAH/SPATA13/FCGR2A/PHACTR1/FGA/FGF10/FHIT/TRAK1/MSRB2/FOXC2/EXPH5/NFASC/EPB41L3/GGA3/DIP2A/FLOT2/MLC1/TBC1D1/NUP210/NEDD4L/SYNE1/PPP1R13B/PUM2/RYBP/MAPK8IP2/TSSK2/VGLL2/TTLL10/RNF144B/CCDC110/SAMM50/DFNB31/ALS2CL/PNKD/SACS/RPS6KC1/PABPC1/AKAP8L/DNAJC2/FGF22/NPTN/BMP10/ZNF844/THEM5/FFAR2/TRIM42/GRB10/DNAJC15/TMOD4/GUCY1A3/SOX8/HK1/HLA-DOA/NR4A1/ACACB/HOXC4/HSPA1L/HSP90AB1/ADAMTSL5/ANKRD45/TFAP2E/ID3/RSPO2/IGF1/IGF2/LCE2D/IL1R1/IL1RN/IL10RA/IL12RB2/IL15RA/IL16/FOXK2/AQP5/INHBA/INPP5A/IRF1/ITGA7/ITGB2/ITGB7/ITIH4/IVL/JUP/CD82/KCNH2/KCNJ8/KCNJ9/KDR/KIF25/IPO5/C17orf82/HES5/RBM12B/LAIR2/LAMA3/STMN1/LCP1/LDLR/ARHGDIA/LGALS9/C11orf87/LMNA/LMO2/LPP/MC2R/MCC/SCGB2A1/MITF/LHX8/ASGR1/MOV10/MYH4/MYL2/NUBP1/DRG1/ATP1A2/NFATC3/NHLH2/NPPC/NRAS/OAS2/OPRL1/SLC22A18/P2RY6/ATP5B/PARK2/BOLA1/UTP11L/PRR16/C11orf73/SIRT6/PITX2/PKHD1/PKM/PRKAG3/PML/FXYD6/TLR9/TREM1/SSH1/POU2AF1/APBB1IP/FBLIM1/MED18/RPP25/TTC12/BANP/PPP1CB/ELP3/GOLPH3L/PPP2R2B/FANCI/MOB1A/MIS18BP1/TRPV6/PEX26/FRMD4A/CARKD/PARVA/PRKAR1B/TTC17/IFT122/LMBRD1/PAG1/MAPK3/MAP2K2/PRKRIR/PROC/MRAP/TRPV5/PRMT8/HTRA1/PAK6/ARNTL2/RGMA/PSMD7/SLURP1/TENM2/GATAD2B/ERMN/MARK4/PTPRE/CREBZF/FAM60A/ACTA2/RFC2/TRIM27/RGR/RIT2/RPA3/SCT/CCL11/NPAS3/PARVG/NOD2/TINAGL1/ARHGAP9/DNAI2/VPS33A/BMP4/SLC4A1/ZNF649/ZG16/SLC6A12/SLC8A1/SLC9A3/BMPR1B/SLIT1/BRD9/BOK/STAT2/STK3/SUPT6H/BST2/TCEB2/ZEB1/TEAD3/TERF1/TGM2/TCHH/TNFAIP3/TNFRSF1A/TRAF5/TRPC6/CCR2/WNT10B/ZAP70/RAB7A/CCDC86/CARD14/BCL2L14/IGFLR1/CSPP1/CALD1/FAM188A/ZC3H12A/FAAP100/CPEB4/ZNF436/CALR/SLIRP/CAST/CAPZB/SLC25A18/ANTXR1/BFSP2/YIPF4/ZNF397/NR0B2/HOPX/PARD6G/RETNLB/TRIM63/GTPBP3/RAE1/IFITM1/SCIN/CDK10/RUNX1/TP63/RUNX3/IRS2/ACTN1/CRADD/TNFRSF11A/SYNJ2/SPHK1/BUD31/CCNA1/ENDOU/SKAP2/STBD1/CCRL2/PRC1/STARD13/PIAS2/SYT7/SLC16A3/CBFA2T2/RSAD2/AURKB/DAPL1/CD8A/TRIP10/ADIPOQ/ENTPD3/MICAL2/N4BP1/VGLL4/RAPGEF2/CD79A/DAZAP2/FGF19/NR1H4	372
**Hypermethylated DMC, Biological Process**								
	Description	GeneRatio	BgRatio	pvalue	p.adjust	qvalue	geneID	Count

GO:0008150	biological_process	311/311	15230/17046	4.33E-16	1.86E-12	1.48E-12	CDH3/TSPAN5/CDH13/MBNL2/KCNMB2/BCKDK/TCIRG1/ABCA9/C1D/ZBTB18/DMRT2/CELF2/TBR1/SEPT9/HCST/NPFFR2/ADCY3/SLC27A2/HIBADH/PSIP1/PXMP4/B4GALT7/PTH2/ADPRHL1/CHRNA5/ZBED9/CIDEA/ALPK2/CLCA1/ANKRD9/FRMD6/C15orf27/ZG16B/SLC38A10/APOA1BP/COL11A1/GALM/SCLT1/ZFP42/CPD/TRPM6/APCDD1/CTGF/LRRC34/CYB561/ADRB3/FITM1/ADAL/TRPV3/ZNF709/ZNF781/CITED4/WBP2NL/RNF168/COCH/NLRP6/DNAH8/DNMT3A/DRD4/DSG3/ECE1/EGFR/EGR3/PATL2/TMEM17/EML1/UNC13D/DNAH12/EPHA3/EPHB4/ALAS1/RNF182/SP8/XRN2/RASA3/PPM1E/VASH1/ACIN1/LIMCH1/TBC1D9B/FOXO1/SPG20/FLNB/ATP11A/LARP1/MTOR/FUCA1/GABBR1/DSCR9/GAK/TENM4/LTN1/RGS22/FBXO2/GAPDHS/GJA3/GJB2/AMPD2/SDCBP2/DKK3/GLS2/VPS4A/GPR162/GNAS/CRACR2B/PIGW/IZUMO1/GPER1/DOK7/FLVCR1/ZBTB44/GSTP1/GTF2B/BRF1/NME7/GZMA/ANXA2/SERPIND1/KCNIP2/NRG1/ANXA6/HLA-B/HLA-E/HLA-F/HLX/HMGA1/HPCA/APBA2/HOXB3/HOXC5/HOXC6/HOXD3/HSD17B2/ACADL/HSP90AA1/HTR5A/COL28A1/FMN1/BARHL2/CYR61/IL6/ISL1/ITIH3/HILS1/ATP9B/ACAT1/KRT7/KRT15/INSC/TOMM20L/SLC6A17/RESP18/CDHR4/LCK/LLGL1/LOX/LTB/LTBP1/SMAD3/ME1/MEF2D/MAP3K1/MEOX1/MEOX2/MFI2/MT1A/NUDT1/NEDD9/NFYB/NMBR/NOV/NTF3/PALM/ARHGEF3/LEF1/DDX47/CEND1/ANGPT4/PDE4C/PDE7A/GALNT7/PGAM2/PIGC/PIK3CG/SPA17/PLEC/IL20RB/RIPK4/CYTL1/POMC/PON1/MOV10L1/ZDHHC13/ROBO4/BNC2/LPCAT2/PPP1CC/PIWIL2/ARHGEF10L/PRMT6/ZNF532/WDR33/SMPD3/CNOT11/SYBU/LIMS2/VAC14/PRKD1/BIN3/PCDHGC4/PCDHGB7/PCDHGA11/MASP1/PSMB4/TRPC7/LPAR5/ACTR3B/METTL14/CCAR2/PTPRCAP/PXN/RASGRF2/EXOC4/RPL8/DEFB134/RPL29/S100A4/S100A6/CCL17/C19orf33/SFRP2/CXCR5/TRA2B/SGK1/SYNDIG1L/MICAL1/CERK/SOX9/STK10/VAMP2/TAF4B/TBP/TCEA1/ACTC1/TIMP3/TLE3/TLR5/TRAPPC10/TNXB/TRAF1/TRPC4/PHLDA2/TWIST1/UPP1/VARS/YWHAG/ZNF124/ZNF177/PTP4A1/CACNB2/PAX8/CXCR4/FZD5/REEP5/PPDPF/GDPD3/TMEM204/NLRX1/EPHX3/ZC3H14/ZNF606/RAB11FIP1/RNF39/COL18A1/UNC93B1/CAPS/COLQ/HIST1H2BM/SH3BGRL3/SCRT1/HIST1H3A/SLA2/PARD6B/IL1F10/SPINK7/KDM2B/LOXL3/MGARP/CBX2/GAS7/FADD/LIMD1/MAP7/ZFAND2A/LDB2/SMDT1/RCSD1/IL32/REEP6/SDR42E1/ARHGAP29/LY86/RAB3D/H2AFY/SMAD5-AS1/MTL5/ARHGEF10/ULK2/USP6NL/TELO2/RABGAP1L	311
GO:0007275	multicellular organismal development	132/311	4527/17046	7.10E-10	1.52E-06	1.21E-06	CDH3/CDH13/ZBTB18/DMRT2/TBR1/SLC38A10/COL11A1/SCLT1/ZFP42/APCDD1/CTGF/RNF168/DNMT3A/DRD4/ECE1/EGFR/EGR3/EML1/EPHA3/EPHB4/SP8/RASA3/VASH1/ACIN1/FOXO1/SPG20/FLNB/MTOR/TENM4/GJB2/SDCBP2/DKK3/VPS4A/GNAS/GPER1/FLVCR1/GSTP1/NME7/ANXA2/KCNIP2/NRG1/HLA-B/HLX/HPCA/APBA2/HOXB3/HOXC5/HOXC6/HOXD3/HSD17B2/HSP90AA1/HTR5A/FMN1/BARHL2/CYR61/IL6/ISL1/HILS1/ACAT1/INSC/SLC6A17/RESP18/LCK/LLGL1/LOX/LTB/SMAD3/MEF2D/MAP3K1/MEOX1/MEOX2/NOV/NTF3/PALM/LEF1/CEND1/ANGPT4/PIK3CG/CYTL1/MOV10L1/ROBO4/BNC2/PPP1CC/PIWIL2/SMPD3/LIMS2/PRKD1/BIN3/PSMB4/TRPC7/PXN/RASGRF2/RPL29/S100A4/S100A6/CCL17/SFRP2/CXCR5/TRA2B/SGK1/SOX9/TCEA1/ACTC1/TIMP3/TLE3/TLR5/TRPC4/PHLDA2/TWIST1/YWHAG/PTP4A1/CACNB2/PAX8/CXCR4/FZD5/PPDPF/TMEM204/COL18A1/COLQ/SCRT1/PARD6B/KDM2B/LOXL3/CBX2/GAS7/FADD/LIMD1/LDB2/H2AFY/MTL5/ARHGEF10/ULK2	132
GO:0048856	anatomical structure development	136/311	4751/17046	1.14E-09	1.63E-06	1.30E-06	CDH3/CDH13/ZBTB18/DMRT2/TBR1/FRMD6/SLC38A10/COL11A1/SCLT1/ZFP42/APCDD1/CTGF/RNF168/COCH/DNMT3A/DRD4/ECE1/EGFR/EGR3/TMEM17/EML1/UNC13D/EPHA3/EPHB4/SP8/RASA3/VASH1/ACIN1/FOXO1/SPG20/FLNB/MTOR/TENM4/GAPDHS/GJB2/SDCBP2/DKK3/GNAS/GPER1/FLVCR1/GSTP1/NME7/ANXA2/KCNIP2/NRG1/HLA-B/HLX/HPCA/APBA2/HOXB3/HOXC5/HOXC6/HOXD3/HSD17B2/HSP90AA1/HTR5A/FMN1/BARHL2/CYR61/IL6/ISL1/HILS1/ACAT1/KRT15/INSC/SLC6A17/RESP18/LCK/LLGL1/LOX/LTB/SMAD3/MEF2D/MAP3K1/MEOX1/MEOX2/MFI2/NOV/NTF3/PALM/LEF1/CEND1/ANGPT4/PIK3CG/RIPK4/CYTL1/MOV10L1/ROBO4/BNC2/PPP1CC/PIWIL2/SMPD3/LIMS2/PRKD1/BIN3/PSMB4/TRPC7/PXN/RASGRF2/S100A4/S100A6/CCL17/SFRP2/CXCR5/TRA2B/SGK1/SOX9/TAF4B/TCEA1/ACTC1/TIMP3/TLE3/TLR5/TRPC4/PHLDA2/TWIST1/YWHAG/CACNB2/PAX8/CXCR4/FZD5/PPDPF/TMEM204/COL18A1/COLQ/SCRT1/PARD6B/KDM2B/LOXL3/GAS7/FADD/LIMD1/LDB2/H2AFY/ARHGEF10/ULK2	136
GO:0048731	system development	119/311	4014/17046	3.49E-09	3.74E-06	2.98E-06	CDH3/CDH13/ZBTB18/DMRT2/TBR1/SLC38A10/COL11A1/SCLT1/ZFP42/APCDD1/CTGF/RNF168/DNMT3A/DRD4/ECE1/EGFR/EGR3/EML1/EPHA3/EPHB4/RASA3/VASH1/ACIN1/FOXO1/SPG20/FLNB/MTOR/TENM4/GJB2/SDCBP2/DKK3/GNAS/GPER1/FLVCR1/GSTP1/NME7/ANXA2/KCNIP2/NRG1/HLA-B/HLX/HPCA/APBA2/HOXB3/HOXC5/HOXC6/HOXD3/HSD17B2/HSP90AA1/HTR5A/FMN1/BARHL2/CYR61/IL6/ISL1/ACAT1/INSC/SLC6A17/LCK/LLGL1/LOX/LTB/SMAD3/MEF2D/MAP3K1/MEOX1/MEOX2/NOV/NTF3/PALM/LEF1/CEND1/ANGPT4/PIK3CG/CYTL1/ROBO4/BNC2/PPP1CC/SMPD3/LIMS2/PRKD1/BIN3/PSMB4/TRPC7/RASGRF2/S100A4/S100A6/SFRP2/CXCR5/TRA2B/SGK1/SOX9/TCEA1/ACTC1/TIMP3/TLE3/TLR5/TRPC4/PHLDA2/TWIST1/YWHAG/CACNB2/PAX8/CXCR4/FZD5/PPDPF/TMEM204/COL18A1/COLQ/SCRT1/PARD6B/KDM2B/LOXL3/GAS7/FADD/LDB2/H2AFY/ARHGEF10/ULK2	119
GO:0044767	single-organism developmental process	144/311	5251/17046	5.31E-09	4.55E-06	3.63E-06	CDH3/CDH13/ZBTB18/DMRT2/TBR1/FRMD6/SLC38A10/COL11A1/SCLT1/ZFP42/APCDD1/CTGF/ADRB3/RNF168/COCH/DNMT3A/DRD4/ECE1/EGFR/EGR3/TMEM17/EML1/UNC13D/EPHA3/EPHB4/SP8/RASA3/VASH1/ACIN1/FOXO1/SPG20/FLNB/MTOR/TENM4/GAPDHS/GJB2/SDCBP2/DKK3/VPS4A/GNAS/GPER1/FLVCR1/GSTP1/NME7/ANXA2/KCNIP2/NRG1/HLA-B/HLX/HMGA1/HPCA/APBA2/HOXB3/HOXC5/HOXC6/HOXD3/HSD17B2/HSP90AA1/HTR5A/FMN1/BARHL2/CYR61/IL6/ISL1/HILS1/ACAT1/INSC/SLC6A17/RESP18/LCK/LLGL1/LOX/LTB/SMAD3/MEF2D/MAP3K1/MEOX1/MEOX2/MFI2/NOV/NTF3/PALM/LEF1/CEND1/ANGPT4/PIK3CG/RIPK4/CYTL1/MOV10L1/ROBO4/BNC2/PPP1CC/PIWIL2/PRMT6/SMPD3/LIMS2/PRKD1/BIN3/PSMB4/TRPC7/METTL14/PXN/RASGRF2/RPL29/S100A4/S100A6/CCL17/SFRP2/CXCR5/TRA2B/SGK1/SOX9/TAF4B/TCEA1/ACTC1/TIMP3/TLE3/TLR5/TRPC4/PHLDA2/TWIST1/YWHAG/PTP4A1/CACNB2/PAX8/CXCR4/FZD5/PPDPF/TMEM204/COL18A1/COLQ/SCRT1/PARD6B/KDM2B/LOXL3/CBX2/GAS7/FADD/LIMD1/LDB2/H2AFY/MTL5/ARHGEF10/ULK2	144
GO:0032502	developmental process	145/311	5327/17046	7.59E-09	5.42E-06	4.33E-06	CDH3/CDH13/ZBTB18/DMRT2/TBR1/FRMD6/SLC38A10/COL11A1/SCLT1/ZFP42/APCDD1/CTGF/ADRB3/RNF168/COCH/DNMT3A/DRD4/ECE1/EGFR/EGR3/TMEM17/EML1/UNC13D/EPHA3/EPHB4/SP8/RASA3/VASH1/ACIN1/FOXO1/SPG20/FLNB/MTOR/TENM4/GAPDHS/GJB2/SDCBP2/DKK3/VPS4A/GNAS/GPER1/FLVCR1/GSTP1/NME7/ANXA2/KCNIP2/NRG1/HLA-B/HLX/HMGA1/HPCA/APBA2/HOXB3/HOXC5/HOXC6/HOXD3/HSD17B2/HSP90AA1/HTR5A/FMN1/BARHL2/CYR61/IL6/ISL1/HILS1/ACAT1/KRT15/INSC/SLC6A17/RESP18/LCK/LLGL1/LOX/LTB/SMAD3/MEF2D/MAP3K1/MEOX1/MEOX2/MFI2/NOV/NTF3/PALM/LEF1/CEND1/ANGPT4/PIK3CG/RIPK4/CYTL1/MOV10L1/ROBO4/BNC2/PPP1CC/PIWIL2/PRMT6/SMPD3/LIMS2/PRKD1/BIN3/PSMB4/TRPC7/METTL14/PXN/RASGRF2/RPL29/S100A4/S100A6/CCL17/SFRP2/CXCR5/TRA2B/SGK1/SOX9/TAF4B/TCEA1/ACTC1/TIMP3/TLE3/TLR5/TRPC4/PHLDA2/TWIST1/YWHAG/PTP4A1/CACNB2/PAX8/CXCR4/FZD5/PPDPF/TMEM204/COL18A1/COLQ/SCRT1/PARD6B/KDM2B/LOXL3/CBX2/GAS7/FADD/LIMD1/LDB2/H2AFY/MTL5/ARHGEF10/ULK2	145

GO:0065007	biological regulation	235/311	10343/17046	1.60E-08	9.80E-06	7.81E-06	CDH3/TSPAN5/CDH13/MBNL2/KCNMB2/TCIRG1/C1D/ZBTB18/DMRT2/CELF2/TBR1/HCST/NPFFR2/ADCY3/PSIP1/B4GALT7/PTH2/CHRNA5/ZBED9/CIDEA/ANKRD9/FRMD6/C15orf27/ZG16B/ZFP42/APCDD1/CTGF/ADRB3/FITM1/TRPV3/ZNF709/ZNF781/CITED4/RNF168/COCH/NLRP6/DNMT3A/DRD4/ECE1/EGFR/EGR3/PATL2/TMEM17/UNC13D/EPHA3/EPHB4/SP8/XRN2/RASA3/PPM1E/VASH1/ACIN1/TBC1D9B/FOXO1/SPG20/FLNB/ATP11A/LARP1/MTOR/GABBR1/TENM4/RGS22/FBXO2/GAPDHS/AMPD2/SDCBP2/DKK3/GLS2/VPS4A/GPR162/GNAS/CRACR2B/GPER1/DOK7/FLVCR1/ZBTB44/GSTP1/GTF2B/BRF1/NME7/GZMA/ANXA2/SERPIND1/KCNIP2/NRG1/ANXA6/HLA-B/HLA-E/HLA-F/HLX/HMGA1/HPCA/APBA2/HOXB3/HOXC5/HOXC6/HOXD3/ACADL/HSP90AA1/HTR5A/COL28A1/FMN1/BARHL2/CYR61/IL6/ISL1/ITIH3/HILS1/ATP9B/LCK/LLGL1/LTB/LTBP1/SMAD3/ME1/MEF2D/MAP3K1/MEOX1/MEOX2/MFI2/MT1A/NEDD9/NFYB/NMBR/NOV/NTF3/PALM/ARHGEF3/LEF1/DDX47/CEND1/ANGPT4/PDE4C/PDE7A/PGAM2/PIK3CG/IL20RB/RIPK4/CYTL1/POMC/PON1/ZDHHC13/ROBO4/BNC2/PPP1CC/PIWIL2/ARHGEF10L/PRMT6/ZNF532/SMPD3/CNOT11/SYBU/LIMS2/VAC14/PRKD1/BIN3/MASP1/PSMB4/TRPC7/LPAR5/ACTR3B/METTL14/CCAR2/PXN/RASGRF2/S100A4/S100A6/CCL17/SFRP2/CXCR5/TRA2B/SGK1/MICAL1/SOX9/STK10/VAMP2/TAF4B/TBP/TCEA1/ACTC1/TIMP3/TLE3/TLR5/TNXB/TRAF1/TRPC4/PHLDA2/TWIST1/VARS/YWHAG/ZNF124/ZNF177/PTP4A1/CACNB2/PAX8/CXCR4/FZD5/TMEM204/NLRX1/ZC3H14/ZNF606/RAB11FIP1/COL18A1/UNC93B1/CAPS/COLQ/SH3BGRL3/SCRT1/HIST1H3A/SLA2/PARD6B/IL1F10/SPINK7/KDM2B/LOXL3/MGARP/CBX2/GAS7/FADD/LIMD1/ZFAND2A/LDB2/SMDT1/REEP6/ARHGAP29/LY86/RAB3D/H2AFY/SMAD5-AS1/MTL5/ARHGEF10/ULK2/USP6NL/TELO2/RABGAP1L	235
GO:0032501	multicellular organismal process	165/311	6425/17046	2.02E-08	1.08E-05	8.61E-06	CDH3/CDH13/KCNMB2/ZBTB18/DMRT2/CELF2/TBR1/ADCY3/CHRNA5/CIDEA/ZG16B/SLC38A10/COL11A1/SCLT1/ZFP42/APCDD1/CTGF/ADRB3/TRPV3/RNF168/COCH/NLRP6/DNMT3A/DRD4/ECE1/EGFR/EGR3/EML1/EPHA3/EPHB4/SP8/XRN2/RASA3/VASH1/ACIN1/FOXO1/SPG20/FLNB/MTOR/TENM4/GAPDHS/GJA3/GJB2/AMPD2/SDCBP2/DKK3/VPS4A/GNAS/IZUMO1/GPER1/FLVCR1/GSTP1/NME7/ANXA2/SERPIND1/KCNIP2/NRG1/ANXA6/HLA-B/HLA-E/HLX/HPCA/APBA2/HOXB3/HOXC5/HOXC6/HOXD3/HSD17B2/ACADL/HSP90AA1/HTR5A/FMN1/BARHL2/CYR61/IL6/ISL1/HILS1/ACAT1/INSC/SLC6A17/RESP18/LCK/LLGL1/LOX/LTB/SMAD3/MEF2D/MAP3K1/MEOX1/MEOX2/NOV/NTF3/PALM/LEF1/CEND1/ANGPT4/PGAM2/PIK3CG/SPA17/IL20RB/CYTL1/POMC/MOV10L1/ROBO4/BNC2/PPP1CC/PIWIL2/WDR33/SMPD3/LIMS2/PRKD1/BIN3/PSMB4/TRPC7/METTL14/PXN/RASGRF2/RPL29/S100A4/S100A6/CCL17/SFRP2/CXCR5/TRA2B/SGK1/SOX9/TAF4B/TBP/TCEA1/ACTC1/TIMP3/TLE3/TLR5/TNXB/TRPC4/PHLDA2/TWIST1/YWHAG/PTP4A1/CACNB2/PAX8/CXCR4/FZD5/PPDPF/TMEM204/NLRX1/RAB11FIP1/COL18A1/COLQ/SH3BGRL3/SCRT1/HIST1H3A/PARD6B/KDM2B/LOXL3/CBX2/GAS7/FADD/LIMD1/LDB2/RCSD1/H2AFY/MTL5/ARHGEF10/ULK2	165
GO:0050789	regulation of biological process	226/311	9837/17046	2.42E-08	1.15E-05	9.18E-06	CDH3/TSPAN5/CDH13/MBNL2/KCNMB2/TCIRG1/C1D/ZBTB18/DMRT2/CELF2/TBR1/HCST/NPFFR2/ADCY3/PSIP1/B4GALT7/PTH2/CHRNA5/ZBED9/CIDEA/ANKRD9/FRMD6/ZFP42/APCDD1/CTGF/ADRB3/FITM1/TRPV3/ZNF709/ZNF781/CITED4/RNF168/COCH/NLRP6/DNMT3A/DRD4/ECE1/EGFR/EGR3/PATL2/TMEM17/UNC13D/EPHA3/EPHB4/SP8/XRN2/RASA3/PPM1E/VASH1/ACIN1/TBC1D9B/FOXO1/SPG20/FLNB/LARP1/MTOR/GABBR1/TENM4/RGS22/FBXO2/GAPDHS/SDCBP2/DKK3/GLS2/VPS4A/GPR162/GNAS/CRACR2B/GPER1/DOK7/FLVCR1/ZBTB44/GSTP1/GTF2B/BRF1/NME7/GZMA/ANXA2/SERPIND1/KCNIP2/NRG1/ANXA6/HLA-B/HLA-E/HLA-F/HLX/HMGA1/HPCA/APBA2/HOXB3/HOXC5/HOXC6/HOXD3/ACADL/HSP90AA1/HTR5A/COL28A1/FMN1/BARHL2/CYR61/IL6/ISL1/ITIH3/HILS1/LCK/LLGL1/LTB/LTBP1/SMAD3/ME1/MEF2D/MAP3K1/MEOX1/MEOX2/MFI2/MT1A/NEDD9/NFYB/NMBR/NOV/NTF3/PALM/ARHGEF3/LEF1/DDX47/CEND1/ANGPT4/PDE4C/PDE7A/PGAM2/PIK3CG/IL20RB/RIPK4/CYTL1/POMC/PON1/ZDHHC13/ROBO4/BNC2/PPP1CC/PIWIL2/ARHGEF10L/PRMT6/ZNF532/SMPD3/CNOT11/SYBU/LIMS2/VAC14/PRKD1/BIN3/MASP1/PSMB4/LPAR5/ACTR3B/METTL14/CCAR2/PXN/RASGRF2/S100A4/S100A6/CCL17/SFRP2/CXCR5/TRA2B/SGK1/MICAL1/SOX9/STK10/VAMP2/TAF4B/TBP/TCEA1/ACTC1/TIMP3/TLE3/TLR5/TNXB/TRAF1/PHLDA2/TWIST1/VARS/YWHAG/ZNF124/ZNF177/PTP4A1/CACNB2/PAX8/CXCR4/FZD5/TMEM204/NLRX1/ZC3H14/ZNF606/RAB11FIP1/COL18A1/UNC93B1/CAPS/COLQ/SH3BGRL3/SCRT1/HIST1H3A/SLA2/PARD6B/IL1F10/SPINK7/KDM2B/LOXL3/MGARP/CBX2/GAS7/FADD/LIMD1/ZFAND2A/LDB2/REEP6/ARHGAP29/LY86/RAB3D/H2AFY/SMAD5-AS1/ARHGEF10/ULK2/USP6NL/TELO2/RABGAP1L	226
GO:0044699	single-organism process	267/311	12449/17046	3.37E-08	1.44E-05	1.15E-05	CDH3/TSPAN5/CDH13/KCNMB2/BCKDK/TCIRG1/ABCA9/C1D/ZBTB18/DMRT2/CELF2/TBR1/SEPT9/HCST/NPFFR2/ADCY3/SLC27A2/HIBADH/B4GALT7/PTH2/CHRNA5/CIDEA/CLCA1/ANKRD9/FRMD6/C15orf27/ZG16B/SLC38A10/APOA1BP/COL11A1/GALM/SCLT1/ZFP42/TRPM6/APCDD1/CTGF/CYB561/ADRB3/FITM1/ADAL/TRPV3/WBP2NL/RNF168/COCH/NLRP6/DNAH8/DNMT3A/DRD4/DSG3/ECE1/EGFR/EGR3/TMEM17/EML1/UNC13D/DNAH12/EPHA3/EPHB4/ALAS1/SP8/XRN2/RASA3/PPM1E/VASH1/ACIN1/LIMCH1/FOXO1/SPG20/FLNB/ATP11A/LARP1/MTOR/FUCA1/GABBR1/GAK/TENM4/RGS22/FBXO2/GAPDHS/GJA3/GJB2/AMPD2/SDCBP2/DKK3/GLS2/VPS4A/GPR162/GNAS/CRACR2B/PIGW/IZUMO1/GPER1/FLVCR1/GSTP1/NME7/GZMA/ANXA2/SERPIND1/KCNIP2/NRG1/ANXA6/HLA-B/HLA-E/HLA-F/HLX/HMGA1/HPCA/APBA2/HOXB3/HOXC5/HOXC6/HOXD3/HSD17B2/ACADL/HSP90AA1/HTR5A/COL28A1/FMN1/BARHL2/CYR61/IL6/ISL1/HILS1/ATP9B/ACAT1/KRT15/INSC/TOMM20L/SLC6A17/RESP18/LCK/LLGL1/LOX/LTB/LTBP1/SMAD3/ME1/MEF2D/MAP3K1/MEOX1/MEOX2/MFI2/NUDT1/NEDD9/NFYB/NMBR/NOV/NTF3/PALM/ARHGEF3/LEF1/DDX47/CEND1/ANGPT4/PDE4C/PDE7A/GALNT7/PGAM2/PIGC/PIK3CG/SPA17/PLEC/IL20RB/RIPK4/CYTL1/POMC/PON1/MOV10L1/ZDHHC13/ROBO4/BNC2/LPCAT2/PPP1CC/PIWIL2/ARHGEF10L/PRMT6/WDR33/SMPD3/CNOT11/SYBU/LIMS2/VAC14/PRKD1/BIN3/MASP1/PSMB4/TRPC7/LPAR5/ACTR3B/METTL14/CCAR2/PXN/RASGRF2/EXOC4/RPL8/RPL29/S100A4/S100A6/CCL17/SFRP2/CXCR5/TRA2B/SGK1/MICAL1/CERK/SOX9/STK10/VAMP2/TAF4B/TBP/TCEA1/ACTC1/TIMP3/TLE3/TLR5/TRAPPC10/TNXB/TRAF1/TRPC4/PHLDA2/TWIST1/UPP1/VARS/YWHAG/PTP4A1/CACNB2/PAX8/CXCR4/FZD5/PPDPF/GDPD3/TMEM204/NLRX1/EPHX3/RAB11FIP1/COL18A1/UNC93B1/CAPS/COLQ/SH3BGRL3/SCRT1/HIST1H3A/SLA2/PARD6B/IL1F10/KDM2B/LOXL3/MGARP/CBX2/GAS7/FADD/LIMD1/MAP7/LDB2/SMDT1/RCSD1/SDR42E1/ARHGAP29/LY86/RAB3D/H2AFY/SMAD5-AS1/MTL5/ARHGEF10/ULK2/USP6NL/TELO2	267

GO:0009987	cellular process	285/311	13765/17046	6.00E-08	2.34E-05	1.86E-05	CDH3/TSPAN5/CDH13/MBNL2/KCNMB2/BCKDK/TCIRG1/ABCA9/C1D/ZBTB18/DMRT2/CELF2/TBR1/SEPT9/HCST/NPFFR2/ADCY3/SLC27A2/HIBADH/PSIP1/B4GALT7/PTH2/ADPRHL1/CHRNA5/ZBED9/CIDEA/ALPK2/CLCA1/ANKRD9/FRMD6/C15orf27/SLC38A10/APOA1BP/COL11A1/SCLT1/ZFP42/TRPM6/APCDD1/CTGF/CYB561/ADRB3/FITM1/ADAL/TRPV3/ZNF709/ZNF781/CITED4/WBP2NL/RNF168/COCH/NLRP6/DNAH8/DNMT3A/DRD4/DSG3/ECE1/EGFR/EGR3/PATL2/TMEM17/EML1/UNC13D/DNAH12/EPHA3/EPHB4/ALAS1/RNF182/SP8/XRN2/RASA3/PPM1E/VASH1/ACIN1/LIMCH1/FOXO1/SPG20/FLNB/ATP11A/LARP1/MTOR/GABBR1/GAK/TENM4/LTN1/RGS22/FBXO2/GAPDHS/GJA3/GJB2/AMPD2/SDCBP2/DKK3/GLS2/VPS4A/GPR162/GNAS/PIGW/IZUMO1/GPER1/DOK7/FLVCR1/ZBTB44/GSTP1/GTF2B/BRF1/NME7/GZMA/ANXA2/SERPIND1/KCNIP2/NRG1/ANXA6/HLA-B/HLA-E/HLA-F/HLX/HMGA1/HPCA/APBA2/HOXB3/HOXC5/HOXC6/HOXD3/ACADL/HSP90AA1/HTR5A/COL28A1/FMN1/BARHL2/CYR61/IL6/ISL1/ITIH3/HILS1/ATP9B/ACAT1/KRT7/KRT15/INSC/SLC6A17/LCK/LLGL1/LOX/LTB/LTBP1/SMAD3/ME1/MEF2D/MAP3K1/MEOX1/MEOX2/MFI2/MT1A/NUDT1/NEDD9/NFYB/NMBR/NOV/NTF3/PALM/ARHGEF3/LEF1/DDX47/CEND1/ANGPT4/PDE4C/PDE7A/GALNT7/PGAM2/PIGC/PIK3CG/SPA17/PLEC/IL20RB/RIPK4/CYTL1/POMC/PON1/MOV10L1/ZDHHC13/ROBO4/BNC2/LPCAT2/PPP1CC/PIWIL2/ARHGEF10L/PRMT6/ZNF532/WDR33/SMPD3/CNOT11/SYBU/LIMS2/VAC14/PRKD1/BIN3/MASP1/PSMB4/TRPC7/LPAR5/ACTR3B/METTL14/CCAR2/PXN/RASGRF2/EXOC4/RPL8/RPL29/S100A4/S100A6/CCL17/SFRP2/CXCR5/TRA2B/SGK1/MICAL1/CERK/SOX9/STK10/VAMP2/TAF4B/TBP/TCEA1/ACTC1/TIMP3/TLE3/TLR5/TRAPPC10/TNXB/TRAF1/TRPC4/PHLDA2/TWIST1/UPP1/VARS/YWHAG/ZNF124/ZNF177/PTP4A1/CACNB2/PAX8/CXCR4/FZD5/PPDPF/TMEM204/NLRX1/EPHX3/ZC3H14/ZNF606/RAB11FIP1/COL18A1/UNC93B1/CAPS/COLQ/HIST1H2BM/SH3BGRL3/SCRT1/HIST1H3A/SLA2/PARD6B/IL1F10/SPINK7/KDM2B/LOXL3/MGARP/CBX2/GAS7/FADD/LIMD1/MAP7/ZFAND2A/LDB2/SMDT1/RCSD1/REEP6/ARHGAP29/LY86/RAB3D/H2AFY/SMAD5-AS1/MTL5/ARHGEF10/ULK2/USP6NL/TELO2	285
GO:0050794	regulation of cellular process	216/311	9347/17046	7.37E-08	2.63E-05	2.10E-05	CDH3/TSPAN5/CDH13/MBNL2/TCIRG1/C1D/ZBTB18/DMRT2/TBR1/HCST/NPFFR2/ADCY3/PSIP1/B4GALT7/PTH2/CHRNA5/ZBED9/CIDEA/ANKRD9/FRMD6/ZFP42/APCDD1/CTGF/ADRB3/TRPV3/ZNF709/ZNF781/CITED4/RNF168/COCH/NLRP6/DNMT3A/DRD4/ECE1/EGFR/EGR3/PATL2/TMEM17/UNC13D/EPHA3/EPHB4/SP8/XRN2/RASA3/PPM1E/VASH1/ACIN1/FOXO1/SPG20/FLNB/LARP1/MTOR/GABBR1/TENM4/RGS22/FBXO2/GAPDHS/SDCBP2/DKK3/GLS2/VPS4A/GPR162/GNAS/GPER1/DOK7/ZBTB44/GSTP1/GTF2B/BRF1/NME7/GZMA/ANXA2/SERPIND1/KCNIP2/NRG1/ANXA6/HLA-B/HLA-E/HLA-F/HLX/HMGA1/HPCA/HOXB3/HOXC5/HOXC6/HOXD3/ACADL/HSP90AA1/HTR5A/COL28A1/FMN1/BARHL2/CYR61/IL6/ISL1/ITIH3/HILS1/LCK/LLGL1/LTB/LTBP1/SMAD3/ME1/MEF2D/MAP3K1/MEOX1/MEOX2/MFI2/NEDD9/NFYB/NMBR/NOV/NTF3/PALM/ARHGEF3/LEF1/DDX47/CEND1/ANGPT4/PDE4C/PDE7A/PGAM2/PIK3CG/IL20RB/RIPK4/CYTL1/POMC/ZDHHC13/ROBO4/BNC2/PPP1CC/PIWIL2/ARHGEF10L/PRMT6/ZNF532/SMPD3/CNOT11/SYBU/LIMS2/VAC14/PRKD1/BIN3/MASP1/PSMB4/LPAR5/ACTR3B/METTL14/CCAR2/PXN/RASGRF2/S100A4/S100A6/CCL17/SFRP2/CXCR5/TRA2B/SGK1/MICAL1/SOX9/STK10/VAMP2/TAF4B/TBP/TCEA1/ACTC1/TIMP3/TLE3/TLR5/TNXB/TRAF1/PHLDA2/TWIST1/VARS/YWHAG/ZNF124/ZNF177/PTP4A1/CACNB2/PAX8/CXCR4/FZD5/TMEM204/NLRX1/ZC3H14/ZNF606/RAB11FIP1/COL18A1/UNC93B1/CAPS/COLQ/SH3BGRL3/SCRT1/HIST1H3A/SLA2/PARD6B/IL1F10/SPINK7/KDM2B/LOXL3/MGARP/CBX2/GAS7/FADD/LIMD1/ZFAND2A/LDB2/REEP6/ARHGAP29/LY86/RAB3D/H2AFY/SMAD5-AS1/ARHGEF10/ULK2/USP6NL/TELO2	216
GO:0044707	single-multicellular organism process	158/311	6214/17046	1.30E-07	4.29E-05	3.42E-05	CDH3/CDH13/KCNMB2/ZBTB18/DMRT2/CELF2/TBR1/ADCY3/CHRNA5/CIDEA/ZG16B/SLC38A10/COL11A1/SCLT1/ZFP42/APCDD1/CTGF/ADRB3/TRPV3/RNF168/COCH/NLRP6/DNMT3A/DRD4/ECE1/EGFR/EGR3/EML1/EPHA3/EPHB4/SP8/RASA3/VASH1/ACIN1/FOXO1/SPG20/FLNB/MTOR/TENM4/GJA3/GJB2/AMPD2/SDCBP2/DKK3/VPS4A/GNAS/GPER1/FLVCR1/GSTP1/NME7/ANXA2/SERPIND1/KCNIP2/NRG1/ANXA6/HLA-B/HLA-E/HLX/HPCA/APBA2/HOXB3/HOXC5/HOXC6/HOXD3/HSD17B2/ACADL/HSP90AA1/HTR5A/FMN1/BARHL2/CYR61/IL6/ISL1/HILS1/ACAT1/INSC/SLC6A17/RESP18/LCK/LLGL1/LOX/LTB/SMAD3/MEF2D/MAP3K1/MEOX1/MEOX2/NOV/NTF3/PALM/LEF1/CEND1/ANGPT4/PGAM2/PIK3CG/IL20RB/CYTL1/POMC/MOV10L1/ROBO4/BNC2/PPP1CC/PIWIL2/SMPD3/LIMS2/PRKD1/BIN3/PSMB4/TRPC7/METTL14/PXN/RASGRF2/RPL29/S100A4/S100A6/CCL17/SFRP2/CXCR5/TRA2B/SGK1/SOX9/TCEA1/ACTC1/TIMP3/TLE3/TLR5/TNXB/TRPC4/PHLDA2/TWIST1/YWHAG/PTP4A1/CACNB2/PAX8/CXCR4/FZD5/PPDPF/TMEM204/NLRX1/RAB11FIP1/COL18A1/COLQ/SH3BGRL3/SCRT1/HIST1H3A/PARD6B/KDM2B/LOXL3/CBX2/GAS7/FADD/LIMD1/LDB2/RCSD1/H2AFY/MTL5/ARHGEF10/ULK2	158
GO:0048468	cell development	67/311	1998/17046	4.85E-07	0.00015	0.00012	ZBTB18/TBR1/FRMD6/COL11A1/SCLT1/ZFP42/EGFR/UNC13D/EPHA3/EPHB4/RASA3/SPG20/FLNB/MTOR/TENM4/GAPDHS/GPER1/FLVCR1/ANXA2/KCNIP2/NRG1/HOXB3/HOXD3/HSP90AA1/FMN1/BARHL2/CYR61/IL6/ISL1/HILS1/LLGL1/SMAD3/MAP3K1/MFI2/NTF3/PALM/LEF1/MOV10L1/PIWIL2/PRKD1/BIN3/PSMB4/TRPC7/PXN/RASGRF2/S100A4/S100A6/SFRP2/SGK1/SOX9/TAF4B/ACTC1/TRPC4/TWIST1/YWHAG/CACNB2/PAX8/CXCR4/FZD5/COL18A1/SCRT1/PARD6B/LOXL3/GAS7/LIMD1/ARHGEF10/ULK2	67
GO:0009653	anatomical structure morphogenesis	80/311	2579/17046	6.92E-07	0.0002	0.00016	CDH13/DMRT2/TBR1/FRMD6/COL11A1/SCLT1/CTGF/COCH/ECE1/EGFR/EGR3/TMEM17/UNC13D/EPHA3/EPHB4/SP8/RASA3/VASH1/FOXO1/SPG20/FLNB/TENM4/DKK3/GNAS/FLVCR1/ANXA2/NRG1/HLX/HOXB3/HOXD3/HSP90AA1/FMN1/BARHL2/CYR61/IL6/ISL1/LLGL1/SMAD3/MEF2D/MAP3K1/MEOX1/MEOX2/MFI2/NOV/NTF3/PALM/LEF1/CEND1/ANGPT4/PIK3CG/RIPK4/ROBO4/BNC2/LIMS2/PRKD1/BIN3/PSMB4/TRPC7/PXN/RASGRF2/S100A4/S100A6/SFRP2/SGK1/SOX9/ACTC1/TLE3/TRPC4/PHLDA2/TWIST1/CACNB2/PAX8/FZD5/COL18A1/PARD6B/KDM2B/LOXL3/GAS7/LIMD1/ULK2	80
GO:0048518	positive regulation of biological process	130/311	4960/17046	9.63E-07	0.00026	0.00021	CDH3/TSPAN5/CDH13/TCIRG1/DMRT2/TBR1/HCST/ADCY3/PSIP1/CIDEA/CTGF/ADRB3/FITM1/TRPV3/CITED4/RNF168/COCH/NLRP6/DNMT3A/DRD4/ECE1/EGFR/EGR3/UNC13D/EPHA3/RASA3/PPM1E/ACIN1/TBC1D9B/FOXO1/LARP1/MTOR/TENM4/RGS22/GAPDHS/GLS2/VPS4A/GNAS/GPER1/DOK7/GSTP1/BRF1/GZMA/ANXA2/NRG1/HLA-B/HLA-E/HLX/HMGA1/HPCA/HOXD3/HSP90AA1/FMN1/BARHL2/CYR61/IL6/ISL1/LCK/LLGL1/LTB/SMAD3/MEF2D/MAP3K1/MEOX1/MEOX2/MFI2/NFYB/NOV/NTF3/PALM/ARHGEF3/LEF1/ANGPT4/PIK3CG/IL20RB/RIPK4/CYTL1/POMC/PON1/ZDHHC13/PIWIL2/ARHGEF10L/SMPD3/LIMS2/PRKD1/MASP1/PSMB4/ACTR3B/CCAR2/PXN/RASGRF2/S100A4/S100A6/CCL17/SFRP2/CXCR5/TRA2B/SGK1/SOX9/STK10/VAMP2/TBP/TCEA1/ACTC1/TLR5/TRAF1/TWIST1/YWHAG/PTP4A1/CACNB2/PAX8/CXCR4/FZD5/NLRX1/COL18A1/UNC93B1/SH3BGRL3/SLA2/MGARP/FADD/LIMD1/ZFAND2A/LDB2/ARHGAP29/LY86/RAB3D/H2AFY/ARHGEF10/USP6NL/RABGAP1L	130

GO:0019222	regulation of metabolic process	152/311	6084/17046	1.03E-06	0.00026	0.00021	CDH3/CDH13/MBNL2/C1D/ZBTB18/DMRT2/TBR1/NPFFR2/ADCY3/PSIP1/ZBED9/CIDEA/ZFP42/CTGF/ADRB3/ZNF709/ZNF781/CITED4/RNF168/NLRP6/DNMT3A/DRD4/ECE1/EGFR/EGR3/PATL2/EPHA3/SP8/XRN2/RASA3/PPM1E/ACIN1/TBC1D9B/FOXO1/SPG20/LARP1/MTOR/GABBR1/RGS22/FBXO2/GAPDHS/DKK3/GNAS/GPER1/DOK7/ZBTB44/GSTP1/GTF2B/BRF1/GZMA/ANXA2/SERPIND1/NRG1/HLX/HMGA1/HPCA/APBA2/HOXB3/HOXC5/HOXC6/HOXD3/ACADL/HSP90AA1/COL28A1/BARHL2/CYR61/IL6/ISL1/ITIH3/HILS1/LCK/LLGL1/LTB/SMAD3/ME1/MEF2D/MAP3K1/MEOX1/MEOX2/MFI2/NFYB/NOV/NTF3/PALM/ARHGEF3/LEF1/ANGPT4/PGAM2/PIK3CG/RIPK4/CYTL1/POMC/BNC2/PPP1CC/PIWIL2/ARHGEF10L/PRMT6/ZNF532/CNOT11/VAC14/PRKD1/MASP1/PSMB4/METTL14/CCAR2/PXN/RASGRF2/CCL17/SFRP2/TRA2B/SGK1/SOX9/STK10/TAF4B/TBP/TCEA1/ACTC1/TIMP3/TLE3/TLR5/TNXB/TRAF1/PHLDA2/TWIST1/VARS/YWHAG/ZNF124/ZNF177/PAX8/CXCR4/FZD5/ZC3H14/ZNF606/SH3BGRL3/SCRT1/HIST1H3A/SLA2/SPINK7/KDM2B/LOXL3/CBX2/GAS7/FADD/LIMD1/ZFAND2A/LDB2/ARHGAP29/H2AFY/ARHGEF10/ULK2/USP6NL/RABGAP1L	152
GO:0031323	regulation of cellular metabolic process	137/311	5352/17046	1.54E-06	0.00037	0.00029	CDH3/CDH13/MBNL2/C1D/ZBTB18/DMRT2/TBR1/NPFFR2/ADCY3/PSIP1/ZBED9/CIDEA/ZFP42/CTGF/ADRB3/ZNF709/ZNF781/CITED4/RNF168/NLRP6/DNMT3A/DRD4/ECE1/EGFR/EGR3/PATL2/SP8/XRN2/RASA3/PPM1E/ACIN1/FOXO1/SPG20/LARP1/MTOR/GABBR1/FBXO2/GAPDHS/DKK3/GNAS/GPER1/DOK7/ZBTB44/GSTP1/GTF2B/BRF1/GZMA/ANXA2/SERPIND1/NRG1/HLX/HMGA1/HPCA/HOXB3/HOXC5/HOXC6/HOXD3/ACADL/HSP90AA1/COL28A1/BARHL2/CYR61/IL6/ISL1/ITIH3/HILS1/LCK/LTB/SMAD3/ME1/MEF2D/MAP3K1/MEOX1/MEOX2/MFI2/NFYB/NTF3/PALM/LEF1/ANGPT4/PGAM2/PIK3CG/RIPK4/CYTL1/POMC/BNC2/PIWIL2/PRMT6/ZNF532/CNOT11/VAC14/PRKD1/MASP1/PSMB4/METTL14/CCAR2/PXN/RASGRF2/CCL17/SFRP2/TRA2B/SGK1/SOX9/STK10/TAF4B/TBP/TCEA1/TIMP3/TLE3/TLR5/TNXB/TRAF1/PHLDA2/TWIST1/VARS/YWHAG/ZNF124/ZNF177/PAX8/CXCR4/FZD5/ZC3H14/ZNF606/SCRT1/HIST1H3A/SLA2/SPINK7/KDM2B/LOXL3/CBX2/GAS7/FADD/LIMD1/ZFAND2A/LDB2/H2AFY/ULK2	137
GO:0006928	movement of cell or subcellular component	60/311	1789/17046	2.23E-06	0.0005	0.0004	CDH13/TBR1/ADCY3/FRMD6/APCDD1/CTGF/DNAH8/EGFR/EGR3/DNAH12/EPHA3/EPHB4/RASA3/VASH1/GAPDHS/GPER1/NME7/NRG1/HSP90AA1/BARHL2/CYR61/IL6/ISL1/LCK/SMAD3/MAP3K1/NOV/NTF3/PALM/LEF1/CEND1/ANGPT4/PIK3CG/SPA17/ROBO4/PRKD1/BIN3/PSMB4/TRPC7/RASGRF2/CCL17/SFRP2/CXCR5/SGK1/SOX9/STK10/ACTC1/TRPC4/PHLDA2/TWIST1/PTP4A1/CACNB2/CXCR4/COL18A1/SH3BGRL3/SCRT1/PARD6B/MGARP/FADD/LIMD1	60
GO:0030154	cell differentiation	97/311	3469/17046	3.55E-06	0.00076	0.00061	CDH3/ZBTB18/TBR1/FRMD6/COL11A1/SCLT1/ZFP42/APCDD1/CTGF/ADRB3/DNMT3A/EGFR/EGR3/EML1/UNC13D/EPHA3/EPHB4/RASA3/ACIN1/FOXO1/SPG20/FLNB/MTOR/TENM4/GAPDHS/GNAS/GPER1/FLVCR1/ANXA2/KCNIP2/NRG1/HLA-B/HLX/HOXB3/HOXD3/HSP90AA1/FMN1/BARHL2/CYR61/IL6/ISL1/HILS1/INSC/LCK/LLGL1/SMAD3/MEF2D/MAP3K1/MEOX1/MFI2/NOV/NTF3/PALM/LEF1/CEND1/CYTL1/MOV10L1/ROBO4/PPP1CC/PIWIL2/SMPD3/PRKD1/BIN3/PSMB4/TRPC7/PXN/RASGRF2/S100A4/S100A6/CCL17/SFRP2/SGK1/SOX9/TAF4B/TCEA1/ACTC1/TRPC4/TWIST1/YWHAG/CACNB2/PAX8/CXCR4/FZD5/PPDPF/TMEM204/COL18A1/SCRT1/PARD6B/LOXL3/CBX2/GAS7/FADD/LIMD1/H2AFY/MTL5/ARHGEF10/ULK2	97
GO:0048870	cell motility	44/311	1187/17046	5.41E-06	0.00105	0.00084	CDH13/ADCY3/APCDD1/CTGF/DNAH8/EGFR/EGR3/EPHA3/EPHB4/VASH1/GAPDHS/GPER1/NRG1/BARHL2/CYR61/IL6/ISL1/LCK/SMAD3/MAP3K1/NOV/NTF3/LEF1/CEND1/ANGPT4/PIK3CG/ROBO4/PRKD1/BIN3/CCL17/SFRP2/SGK1/SOX9/STK10/PHLDA2/TWIST1/PTP4A1/CXCR4/COL18A1/SH3BGRL3/SCRT1/PARD6B/FADD/LIMD1	44
GO:0051674	localization of cell	44/311	1187/17046	5.41E-06	0.00105	0.00084	CDH13/ADCY3/APCDD1/CTGF/DNAH8/EGFR/EGR3/EPHA3/EPHB4/VASH1/GAPDHS/GPER1/NRG1/BARHL2/CYR61/IL6/ISL1/LCK/SMAD3/MAP3K1/NOV/NTF3/LEF1/CEND1/ANGPT4/PIK3CG/ROBO4/PRKD1/BIN3/CCL17/SFRP2/SGK1/SOX9/STK10/PHLDA2/TWIST1/PTP4A1/CXCR4/COL18A1/SH3BGRL3/SCRT1/PARD6B/FADD/LIMD1	44
GO:0080090	regulation of primary metabolic process	133/311	5271/17046	5.85E-06	0.00109	0.00087	CDH13/MBNL2/C1D/ZBTB18/DMRT2/TBR1/NPFFR2/ADCY3/PSIP1/ZBED9/CIDEA/ZFP42/CTGF/ADRB3/ZNF709/ZNF781/CITED4/RNF168/NLRP6/DNMT3A/DRD4/ECE1/EGFR/EGR3/PATL2/SP8/XRN2/RASA3/PPM1E/ACIN1/FOXO1/SPG20/LARP1/MTOR/GABBR1/FBXO2/GAPDHS/DKK3/GNAS/GPER1/DOK7/ZBTB44/GSTP1/GTF2B/BRF1/GZMA/ANXA2/SERPIND1/NRG1/HLX/HMGA1/HPCA/HOXB3/HOXC5/HOXC6/HOXD3/ACADL/COL28A1/BARHL2/CYR61/IL6/ISL1/ITIH3/HILS1/LCK/LTB/SMAD3/ME1/MEF2D/MAP3K1/MEOX1/MEOX2/MFI2/NFYB/NTF3/PALM/LEF1/ANGPT4/PGAM2/PIK3CG/RIPK4/CYTL1/POMC/BNC2/PIWIL2/PRMT6/ZNF532/CNOT11/VAC14/PRKD1/MASP1/PSMB4/METTL14/CCAR2/PXN/RASGRF2/CCL17/SFRP2/TRA2B/SGK1/SOX9/STK10/TAF4B/TBP/TCEA1/TIMP3/TLE3/TNXB/TRAF1/PHLDA2/TWIST1/VARS/YWHAG/ZNF124/ZNF177/PAX8/CXCR4/FZD5/ZC3H14/ZNF606/SCRT1/HIST1H3A/SLA2/SPINK7/KDM2B/LOXL3/CBX2/GAS7/FADD/LIMD1/ZFAND2A/LDB2/H2AFY	133
GO:0060255	regulation of macromolecule metabolic process	132/311	5249/17046	7.92E-06	0.0014	0.00112	CDH3/CDH13/MBNL2/C1D/ZBTB18/DMRT2/TBR1/NPFFR2/ADCY3/PSIP1/ZBED9/CIDEA/ZFP42/CTGF/ADRB3/ZNF709/ZNF781/CITED4/RNF168/NLRP6/DNMT3A/DRD4/ECE1/EGFR/EGR3/PATL2/SP8/XRN2/RASA3/PPM1E/ACIN1/FOXO1/SPG20/LARP1/MTOR/FBXO2/GAPDHS/DKK3/GNAS/GPER1/DOK7/ZBTB44/GSTP1/GTF2B/BRF1/GZMA/ANXA2/SERPIND1/NRG1/HLX/HMGA1/APBA2/HOXB3/HOXC5/HOXC6/HOXD3/COL28A1/BARHL2/CYR61/IL6/ISL1/ITIH3/HILS1/LCK/LTB/SMAD3/MEF2D/MAP3K1/MEOX1/MEOX2/MFI2/NFYB/NOV/NTF3/LEF1/ANGPT4/PGAM2/PIK3CG/RIPK4/CYTL1/POMC/BNC2/PPP1CC/PIWIL2/PRMT6/ZNF532/CNOT11/PRKD1/MASP1/PSMB4/METTL14/CCAR2/PXN/RASGRF2/CCL17/SFRP2/TRA2B/SGK1/SOX9/STK10/TAF4B/TBP/TCEA1/ACTC1/TIMP3/TLE3/TNXB/TRAF1/PHLDA2/TWIST1/VARS/YWHAG/ZNF124/ZNF177/PAX8/CXCR4/FZD5/ZC3H14/ZNF606/SCRT1/HIST1H3A/SLA2/SPINK7/KDM2B/LOXL3/CBX2/GAS7/FADD/LIMD1/ZFAND2A/LDB2/H2AFY	132
GO:0051239	regulation of multicellular organismal process	70/311	2298/17046	8.18E-06	0.0014	0.00112	CDH3/KCNMB2/DMRT2/CELF2/TBR1/CIDEA/CTGF/ADRB3/TRPV3/NLRP6/DRD4/ECE1/EGR3/EPHA3/VASH1/ACIN1/FOXO1/SPG20/MTOR/TENM4/GNAS/GPER1/FLVCR1/GSTP1/ANXA2/KCNIP2/NRG1/ANXA6/HLA-B/HLA-E/HLX/HOXB3/HOXD3/BARHL2/CYR61/IL6/ISL1/LCK/LTB/SMAD3/NOV/NTF3/PALM/LEF1/CEND1/ANGPT4/PIK3CG/IL20RB/POMC/LIMS2/PRKD1/SFRP2/SGK1/SOX9/TLR5/PHLDA2/TWIST1/YWHAG/PAX8/CXCR4/FZD5/NLRX1/RAB11FIP1/COLQ/SH3BGRL3/SCRT1/FADD/LIMD1/H2AFY/ULK2	70
GO:0016477	cell migration	41/311	1095/17046	9.25E-06	0.00152	0.00122	CDH13/APCDD1/CTGF/EGFR/EGR3/EPHA3/EPHB4/VASH1/GPER1/NRG1/BARHL2/CYR61/IL6/ISL1/LCK/SMAD3/MAP3K1/NOV/NTF3/LEF1/CEND1/ANGPT4/PIK3CG/ROBO4/PRKD1/BIN3/CCL17/SFRP2/SGK1/SOX9/STK10/PHLDA2/TWIST1/PTP4A1/CXCR4/COL18A1/SH3BGRL3/SCRT1/PARD6B/FADD/LIMD1	41

GO:0044763	single-organism cellular process	241/311	11314/17046	1.03E-05	0.00163	0.0013	CDH3/TSPAN5/CDH13/KCNMB2/BCKDK/TCIRG1/ABCA9/C1D/ZBTB18/TBR1/SEPT9/HCST/NPFFR2/ADCY3/SLC27A2/HIBADH/B4GALT7/PTH2/CHRNA5/CIDEA/CLCA1/ANKRD9/FRMD6/C15orf27/SLC38A10/APOA1BP/COL11A1/SCLT1/ZFP42/TRPM6/APCDD1/CTGF/CYB561/ADRB3/FITM1/ADAL/TRPV3/WBP2NL/RNF168/COCH/NLRP6/DNAH8/DNMT3A/DRD4/DSG3/EGFR/EGR3/TMEM17/EML1/UNC13D/DNAH12/EPHA3/EPHB4/ALAS1/XRN2/RASA3/PPM1E/VASH1/ACIN1/LIMCH1/FOXO1/SPG20/FLNB/ATP11A/LARP1/MTOR/GABBR1/GAK/TENM4/RGS22/FBXO2/GAPDHS/GJA3/GJB2/AMPD2/SDCBP2/DKK3/GLS2/VPS4A/GPR162/GNAS/PIGW/IZUMO1/GPER1/FLVCR1/GSTP1/NME7/GZMA/ANXA2/KCNIP2/NRG1/ANXA6/HLA-B/HLA-E/HLA-F/HLX/HMGA1/HPCA/APBA2/HOXB3/HOXD3/ACADL/HSP90AA1/HTR5A/COL28A1/FMN1/BARHL2/CYR61/IL6/ISL1/HILS1/ATP9B/ACAT1/KRT15/INSC/SLC6A17/LCK/LLGL1/LOX/LTB/LTBP1/SMAD3/ME1/MEF2D/MAP3K1/MEOX1/MEOX2/MFI2/NUDT1/NEDD9/NMBR/NOV/NTF3/PALM/ARHGEF3/LEF1/DDX47/CEND1/ANGPT4/PDE4C/PDE7A/GALNT7/PGAM2/PIGC/PIK3CG/SPA17/PLEC/IL20RB/CYTL1/POMC/PON1/MOV10L1/ZDHHC13/ROBO4/LPCAT2/PPP1CC/PIWIL2/ARHGEF10L/PRMT6/WDR33/SMPD3/CNOT11/SYBU/LIMS2/VAC14/PRKD1/BIN3/PSMB4/TRPC7/LPAR5/ACTR3B/CCAR2/PXN/RASGRF2/EXOC4/RPL8/RPL29/S100A4/S100A6/CCL17/SFRP2/CXCR5/SGK1/MICAL1/CERK/SOX9/STK10/VAMP2/TAF4B/TBP/TCEA1/ACTC1/TLE3/TLR5/TRAPPC10/TNXB/TRAF1/TRPC4/PHLDA2/TWIST1/UPP1/VARS/YWHAG/PTP4A1/CACNB2/PAX8/CXCR4/FZD5/PPDPF/TMEM204/NLRX1/EPHX3/RAB11FIP1/COL18A1/UNC93B1/CAPS/COLQ/SH3BGRL3/SCRT1/HIST1H3A/SLA2/PARD6B/IL1F10/KDM2B/LOXL3/MGARP/CBX2/GAS7/FADD/LIMD1/MAP7/SMDT1/ARHGAP29/LY86/RAB3D/H2AFY/SMAD5-AS1/MTL5/ARHGEF10/ULK2/TELO2	241
GO:0001525	angiogenesis	21/311	398/17046	1.43E-05	0.00218	0.00174	CDH13/CTGF/EGR3/EPHB4/VASH1/ANXA2/HOXB3/CYR61/IL6/ISL1/MEOX2/NOV/LEF1/ANGPT4/PIK3CG/ROBO4/PRKD1/SFRP2/TWIST1/FZD5/COL18A1	21
GO:0072358	cardiovascular system development	34/311	861/17046	1.96E-05	0.0028	0.00223	CDH13/COL11A1/CTGF/ECE1/EGR3/EPHB4/VASH1/FOXO1/MTOR/TENM4/FLVCR1/ANXA2/NRG1/HOXB3/CYR61/IL6/ISL1/LOX/SMAD3/MEF2D/MEOX2/NOV/LEF1/ANGPT4/PIK3CG/ROBO4/PRKD1/SFRP2/SOX9/ACTC1/TWIST1/FZD5/TMEM204/COL18A1	34
GO:0072359	circulatory system development	34/311	861/17046	1.96E-05	0.0028	0.00223	CDH13/COL11A1/CTGF/ECE1/EGR3/EPHB4/VASH1/FOXO1/MTOR/TENM4/FLVCR1/ANXA2/NRG1/HOXB3/CYR61/IL6/ISL1/LOX/SMAD3/MEF2D/MEOX2/NOV/LEF1/ANGPT4/PIK3CG/ROBO4/PRKD1/SFRP2/SOX9/ACTC1/TWIST1/FZD5/TMEM204/COL18A1	34
GO:0040011	locomotion	54/311	1666/17046	2.09E-05	0.00289	0.00231	CDH13/TBR1/ADCY3/APCDD1/CTGF/DNAH8/EGFR/EGR3/EPHA3/EPHB4/RASA3/VASH1/GAPDHS/GPER1/SERPIND1/NRG1/HSP90AA1/BARHL2/CYR61/IL6/ISL1/LCK/SMAD3/MAP3K1/NOV/NTF3/LEF1/CEND1/ANGPT4/PIK3CG/ROBO4/PRKD1/BIN3/PSMB4/TRPC7/RASGRF2/CCL17/SFRP2/CXCR5/SGK1/SOX9/STK10/TRPC4/PHLDA2/TWIST1/PTP4A1/CACNB2/CXCR4/COL18A1/SH3BGRL3/SCRT1/PARD6B/FADD/LIMD1	54
GO:0048869	cellular developmental process	99/311	3716/17046	2.25E-05	0.00301	0.0024	CDH3/ZBTB18/TBR1/FRMD6/COL11A1/SCLT1/ZFP42/APCDD1/CTGF/ADRB3/COCH/DNMT3A/EGFR/EGR3/TMEM17/EML1/UNC13D/EPHA3/EPHB4/RASA3/ACIN1/FOXO1/SPG20/FLNB/MTOR/TENM4/GAPDHS/GNAS/GPER1/FLVCR1/ANXA2/KCNIP2/NRG1/HLA-B/HLX/HOXB3/HOXD3/HSP90AA1/FMN1/BARHL2/CYR61/IL6/ISL1/HILS1/INSC/LCK/LLGL1/SMAD3/MEF2D/MAP3K1/MEOX1/MFI2/NOV/NTF3/PALM/LEF1/CEND1/CYTL1/MOV10L1/ROBO4/PPP1CC/PIWIL2/SMPD3/PRKD1/BIN3/PSMB4/TRPC7/PXN/RASGRF2/S100A4/S100A6/CCL17/SFRP2/SGK1/SOX9/TAF4B/TCEA1/ACTC1/TRPC4/TWIST1/YWHAG/CACNB2/PAX8/CXCR4/FZD5/PPDPF/TMEM204/COL18A1/SCRT1/PARD6B/LOXL3/CBX2/GAS7/FADD/LIMD1/H2AFY/MTL5/ARHGEF10/ULK2	99
GO:0072132	mesenchyme morphogenesis	6/311	33/17046	2.57E-05	0.00333	0.00266	ISL1/SMAD3/LEF1/SOX9/ACTC1/TWIST1	6
GO:0031348	negative regulation of defense response	11/311	130/17046	2.64E-05	0.00333	0.00266	NLRP6/GPER1/GSTP1/HLA-B/HLA-E/ISL1/SMAD3/NOV/IL20RB/PSMB4/NLRX1	11
GO:0009888	tissue development	54/311	1684/17046	2.82E-05	0.00345	0.00275	CDH3/ZBTB18/DMRT2/FRMD6/COL11A1/APCDD1/CTGF/DRD4/EGFR/EPHA3/SPG20/FLNB/TENM4/GJB2/GNAS/NRG1/HLX/HPCA/HOXB3/HOXD3/FMN1/BARHL2/CYR61/IL6/ISL1/ACAT1/KRT15/INSC/SMAD3/MEF2D/MAP3K1/MEOX1/MEOX2/NOV/NTF3/LEF1/RIPK4/CYTL1/BNC2/BIN3/PXN/S100A4/SFRP2/SOX9/ACTC1/TIMP3/TWIST1/PAX8/FZD5/COL18A1/KDM2B/LOXL3/LDB2/H2AFY	54
GO:0051171	regulation of nitrogen compound metabolic process	102/311	3889/17046	3.08E-05	0.00347	0.00277	CDH13/MBNL2/C1D/ZBTB18/DMRT2/TBR1/NPFFR2/PSIP1/ZBED9/CIDEA/ZFP42/ADRB3/ZNF709/ZNF781/CITED4/RNF168/DNMT3A/DRD4/EGFR/EGR3/PATL2/SP8/XRN2/ACIN1/FOXO1/SPG20/LARP1/MTOR/GABBR1/GAPDHS/DKK3/GNAS/GPER1/ZBTB44/GTF2B/BRF1/GZMA/NRG1/HLX/HMGA1/HPCA/HOXB3/HOXC5/HOXC6/HOXD3/HSP90AA1/BARHL2/CYR61/IL6/ISL1/HILS1/SMAD3/ME1/MEF2D/MEOX1/MEOX2/NFYB/NTF3/PALM/LEF1/PGAM2/RIPK4/CYTL1/POMC/BNC2/PIWIL2/PRMT6/ZNF532/CNOT11/PRKD1/PSMB4/METTL14/CCAR2/SFRP2/TRA2B/SGK1/SOX9/TAF4B/TBP/TCEA1/TLE3/TLR5/TRAF1/TWIST1/VARS/ZNF124/ZNF177/PAX8/FZD5/ZC3H14/ZNF606/SCRT1/HIST1H3A/SLA2/KDM2B/LOXL3/CBX2/GAS7/FADD/LIMD1/LDB2/H2AFY	102
GO:0040012	regulation of locomotion	28/311	659/17046	3.09E-05	0.00347	0.00277	CDH13/TBR1/EGFR/VASH1/GPER1/CYR61/IL6/SMAD3/MAP3K1/NOV/NTF3/LEF1/ANGPT4/ROBO4/PRKD1/SFRP2/SGK1/SOX9/STK10/PHLDA2/TWIST1/PTP4A1/CXCR4/COL18A1/SH3BGRL3/SCRT1/PARD6B/FADD	28
GO:0048513	organ development	80/311	2848/17046	3.14E-05	0.00347	0.00277	CDH3/ZBTB18/TBR1/SLC38A10/COL11A1/ZFP42/APCDD1/CTGF/DRD4/ECE1/EGFR/EGR3/EML1/EPHA3/EPHB4/ACIN1/FOXO1/SPG20/FLNB/MTOR/TENM4/GJB2/DKK3/GNAS/FLVCR1/NME7/ANXA2/NRG1/HLA-B/HLX/HPCA/HOXB3/HOXD3/HSD17B2/HTR5A/FMN1/BARHL2/CYR61/IL6/ISL1/ACAT1/INSC/SLC6A17/LCK/LOX/LTB/SMAD3/MEF2D/MAP3K1/MEOX1/MEOX2/NOV/NTF3/LEF1/CEND1/CYTL1/BNC2/SMPD3/LIMS2/BIN3/S100A4/SFRP2/CXCR5/TRA2B/SOX9/TCEA1/ACTC1/TLE3/TLR5/PHLDA2/TWIST1/PAX8/FZD5/PPDPF/COL18A1/KDM2B/LOXL3/FADD/LDB2/H2AFY	80

GO:0000904	cell morphogenesis involved in differentiation	36/311	958/17046	3.16E-05	0.00347	0.00277	TBR1/FRMD6/EGFR/UNC13D/EPHA3/EPHB4/RASA3/SPG20/FLNB/NRG1/HSP90AA1/FMN1/BARHL2/ISL1/LLGL1/SMAD3/MAP3K1/MFI2/NTF3/LEF1/PSMB4/TRPC7/PXN/RASGRF2/S100A4/S100A6/SFRP2/SOX9/TRPC4/TWIST1/CACNB2/PAX8/COL18A1/PARD6B/LOXL3/ULK2	36
GO:0007399	nervous system development	65/311	2174/17046	3.39E-05	0.00359	0.00286	ZBTB18/TBR1/SCLT1/APCDD1/DNMT3A/EGFR/EGR3/EML1/EPHA3/EPHB4/RASA3/SPG20/MTOR/TENM4/SDCBP2/GPER1/GSTP1/NME7/KCNIP2/NRG1/HLX/HPCA/APBA2/HOXB3/HOXD3/HSP90AA1/HTR5A/FMN1/BARHL2/IL6/ISL1/ACAT1/INSC/SLC6A17/LLGL1/MEF2D/NTF3/PALM/LEF1/CEND1/PPP1CC/PRKD1/PSMB4/TRPC7/RASGRF2/S100A6/SFRP2/TRA2B/SGK1/SOX9/TIMP3/TRPC4/TWIST1/YWHAG/CACNB2/PAX8/CXCR4/FZD5/COLQ/SCRT1/PARD6B/KDM2B/GAS7/ARHGEF10/ULK2	65
GO:0001944	vasculature development	25/311	557/17046	3.43E-05	0.00359	0.00286	CDH13/CTGF/EGR3/EPHB4/VASH1/FOXO1/FLVCR1/ANXA2/HOXB3/CYR61/IL6/ISL1/LOX/MEOX2/NOV/LEF1/ANGPT4/PIK3CG/ROBO4/PRKD1/SFRP2/TWIST1/FZD5/TMEM204/COL18A1	25
GO:0003002	regionalization	18/311	332/17046	4.13E-05	0.00411	0.00327	DMRT2/TBR1/SP8/NME7/HOXB3/HOXC5/HOXC6/HOXD3/ISL1/SMAD3/MEOX1/MEOX2/LEF1/SFRP2/TRA2B/PAX8/FZD5/KDM2B	18
GO:2000145	regulation of cell motility	26/311	599/17046	4.19E-05	0.00411	0.00327	CDH13/EGFR/VASH1/GPER1/CYR61/IL6/SMAD3/MAP3K1/NOV/NTF3/LEF1/ANGPT4/ROBO4/PRKD1/SFRP2/SGK1/SOX9/STK10/PHLDA2/TWIST1/PTP4A1/COL18A1/SH3BGRL3/SCRT1/PARD6B/FADD	26
GO:0050728	negative regulation of inflammatory response	9/311	91/17046	4.21E-05	0.00411	0.00327	NLRP6/GPER1/GSTP1/ISL1/SMAD3/NOV/IL20RB/PSMB4/NLRX1	9
GO:0030334	regulation of cell migration	25/311	568/17046	4.72E-05	0.00449	0.00358	CDH13/EGFR/VASH1/GPER1/CYR61/IL6/SMAD3/MAP3K1/NOV/NTF3/LEF1/ANGPT4/ROBO4/PRKD1/SFRP2/SGK1/SOX9/STK10/PHLDA2/PTP4A1/COL18A1/SH3BGRL3/SCRT1/PARD6B/FADD	25
GO:0001568	blood vessel development	24/311	536/17046	5.12E-05	0.00477	0.00381	CDH13/CTGF/EGR3/EPHB4/VASH1/FOXO1/FLVCR1/ANXA2/HOXB3/CYR61/IL6/ISL1/LOX/MEOX2/NOV/LEF1/ANGPT4/PIK3CG/ROBO4/PRKD1/SFRP2/TWIST1/FZD5/COL18A1	24
GO:0048522	positive regulation of cellular process	109/311	4283/17046	5.33E-05	0.00486	0.00388	CDH3/TSPAN5/CDH13/TCIRG1/DMRT2/TBR1/HCST/ADCY3/PSIP1/CTGF/ADRB3/TRPV3/CITED4/RNF168/DNMT3A/DRD4/ECE1/EGFR/EGR3/UNC13D/EPHA3/RASA3/PPM1E/ACIN1/FOXO1/LARP1/MTOR/TENM4/GAPDHS/GLS2/VPS4A/GNAS/GPER1/DOK7/GSTP1/BRF1/GZMA/ANXA2/NRG1/HLA-E/HLX/HMGA1/HPCA/HOXD3/HSP90AA1/FMN1/BARHL2/CYR61/IL6/ISL1/LCK/LTB/SMAD3/MEF2D/MAP3K1/MEOX1/MEOX2/MFI2/NFYB/NOV/NTF3/PALM/ARHGEF3/LEF1/ANGPT4/PIK3CG/RIPK4/CYTL1/POMC/ZDHHC13/PIWIL2/SMPD3/LIMS2/PRKD1/PSMB4/ACTR3B/CCAR2/PXN/RASGRF2/S100A4/S100A6/CCL17/SFRP2/CXCR5/TRA2B/SOX9/STK10/VAMP2/TBP/TCEA1/TLR5/TRAF1/TWIST1/YWHAG/PTP4A1/PAX8/CXCR4/FZD5/COL18A1/SLA2/MGARP/FADD/LIMD1/ZFAND2A/LDB2/LY86/RAB3D/H2AFY/ARHGEF10	109
GO:0032989	cellular component morphogenesis	47/311	1432/17046	5.76E-05	0.00514	0.0041	TBR1/FRMD6/SCLT1/COCH/EGFR/TMEM17/UNC13D/EPHA3/EPHB4/RASA3/SPG20/FLNB/TENM4/NRG1/HSP90AA1/FMN1/BARHL2/IL6/ISL1/LLGL1/SMAD3/MAP3K1/MFI2/NTF3/PALM/LEF1/BIN3/PSMB4/TRPC7/PXN/RASGRF2/S100A4/S100A6/SFRP2/SGK1/SOX9/ACTC1/TRPC4/TWIST1/CACNB2/PAX8/COL18A1/PARD6B/LOXL3/GAS7/LIMD1/ULK2	47
GO:0000902	cell morphogenesis	45/311	1352/17046	6.08E-05	0.00532	0.00424	TBR1/FRMD6/SCLT1/COCH/EGFR/TMEM17/UNC13D/EPHA3/EPHB4/RASA3/SPG20/FLNB/NRG1/HSP90AA1/FMN1/BARHL2/IL6/ISL1/LLGL1/SMAD3/MAP3K1/MFI2/NTF3/PALM/LEF1/BIN3/PSMB4/TRPC7/PXN/RASGRF2/S100A4/S100A6/SFRP2/SGK1/SOX9/TRPC4/TWIST1/CACNB2/PAX8/COL18A1/PARD6B/LOXL3/GAS7/LIMD1/ULK2	45
GO:0045761	regulation of adenylate cyclase activity	8/311	75/17046	6.55E-05	0.00556	0.00443	NPFFR2/ADRB3/DRD4/GABBR1/GNAS/GPER1/HPCA/PALM	8
GO:0061448	connective tissue development	14/311	224/17046	6.62E-05	0.00556	0.00443	COL11A1/CTGF/SPG20/GNAS/HOXB3/HOXD3/CYR61/ACAT1/SMAD3/MEF2D/NOV/CYTL1/SFRP2/SOX9	14
GO:0010468	regulation of gene expression	99/311	3819/17046	6.97E-05	0.00575	0.00458	CDH3/CDH13/MBNL2/C1D/ZBTB18/DMRT2/TBR1/PSIP1/ZBED9/CIDEA/ZFP42/CTGF/ZNF709/ZNF781/CITED4/RNF168/DNMT3A/EGFR/EGR3/PATL2/SP8/XRN2/ACIN1/FOXO1/SPG20/LARP1/MTOR/DKK3/GNAS/GPER1/ZBTB44/GTF2B/BRF1/NRG1/HLX/HMGA1/APBA2/HOXB3/HOXC5/HOXC6/HOXD3/BARHL2/CYR61/IL6/ISL1/HILS1/LCK/SMAD3/MEF2D/MEOX1/MEOX2/MFI2/NFYB/NOV/NTF3/LEF1/RIPK4/CYTL1/POMC/BNC2/PPP1CC/PIWIL2/PRMT6/ZNF532/CNOT11/PRKD1/MASP1/METTL14/CCAR2/SFRP2/TRA2B/SGK1/SOX9/TAF4B/TBP/TCEA1/ACTC1/TLE3/TRAF1/PHLDA2/TWIST1/VARS/ZNF124/ZNF177/PAX8/FZD5/ZC3H14/ZNF606/SCRT1/HIST1H3A/SLA2/KDM2B/LOXL3/CBX2/GAS7/FADD/LIMD1/LDB2/H2AFY	99
GO:0051179	localization	126/311	5173/17046	7.73E-05	0.00625	0.00498	CDH3/TSPAN5/CDH13/KCNMB2/TCIRG1/ABCA9/TBR1/ADCY3/SLC27A2/CHRNA5/CIDEA/CLCA1/FRMD6/C15orf27/SLC38A10/TRPM6/APCDD1/CTGF/CYB561/FITM1/TRPV3/NLRP6/DNAH8/DRD4/EGFR/EGR3/UNC13D/EPHA3/EPHB4/RASA3/VASH1/FLNB/ATP11A/MTOR/GAPDHS/GJA3/GJB2/SDCBP2/GLS2/VPS4A/GNAS/CRACR2B/PIGW/GPER1/FLVCR1/GSTP1/NME7/ANXA2/KCNIP2/NRG1/ANXA6/HLA-E/HPCA/APBA2/HSP90AA1/BARHL2/CYR61/IL6/ISL1/ATP9B/INSC/TOMM20L/SLC6A17/LCK/LLGL1/LTBP1/SMAD3/MAP3K1/MFI2/NOV/NTF3/PALM/LEF1/CEND1/ANGPT4/PDE4C/PIK3CG/POMC/PON1/ZDHHC13/ROBO4/SMPD3/SYBU/PRKD1/BIN3/MASP1/TRPC7/RASGRF2/EXOC4/RPL8/RPL29/S100A6/CCL17/SFRP2/SGK1/SOX9/STK10/VAMP2/TRAPPC10/TRPC4/PHLDA2/TWIST1/YWHAG/PTP4A1/CACNB2/PAX8/CXCR4/FZD5/RAB11FIP1/COL18A1/UNC93B1/COLQ/SH3BGRL3/SCRT1/PARD6B/LOXL3/MGARP/FADD/LIMD1/MAP7/SMDT1/REEP6/RAB3D/H2AFY/USP6NL/RABGAP1L	126

GO:0032879	regulation of localization	63/311	2151/17046	8.49E-05	0.00661	0.00527	CDH3/CDH13/TBR1/CIDEA/FITM1/TRPV3/NLRP6/DRD4/EGFR/UNC13D/VASH1/MTOR/GLS2/VPS4A/GNAS/CRACR2B/GPER1/ANXA2/KCNIP2/NRG1/HLA-E/HPCA/CYR61/IL6/ISL1/LCK/LLGL1/SMAD3/MAP3K1/NOV/NTF3/LEF1/ANGPT4/PDE4C/PIK3CG/POMC/PON1/ROBO4/SMPD3/SYBU/PRKD1/RASGRF2/SFRP2/SGK1/SOX9/STK10/VAMP2/PHLDA2/TWIST1/YWHAG/PTP4A1/CACNB2/PAX8/FZD5/RAB11FIP1/COL18A1/SH3BGRL3/SCRT1/PARD6B/FADD/REEP6/RAB3D/RABGAP1L	63
GO:0050790	regulation of catalytic activity	63/311	2151/17046	8.49E-05	0.00661	0.00527	CDH3/NPFFR2/ADCY3/CTGF/ADRB3/DRD4/EGFR/EPHA3/RASA3/PPM1E/TBC1D9B/MTOR/GABBR1/RGS22/GNAS/GPER1/DOK7/GSTP1/GZMA/ANXA2/SERPIND1/NRG1/HPCA/HSP90AA1/COL28A1/CYR61/IL6/ITIH3/LCK/LLGL1/SMAD3/MAP3K1/NTF3/PALM/ARHGEF3/LEF1/ANGPT4/PIK3CG/ARHGEF10L/VAC14/PRKD1/PSMB4/CCAR2/PXN/RASGRF2/CCL17/SFRP2/SGK1/STK10/TCEA1/TIMP3/TNXB/YWHAG/CXCR4/FZD5/SH3BGRL3/SPINK7/FADD/ARHGAP29/H2AFY/ARHGEF10/USP6NL/RABGAP1L	63
GO:0043009	chordate embryonic development	25/311	593/17046	9.38E-05	0.00718	0.00573	ZBTB18/DMRT2/COL11A1/ZFP42/ECE1/EGFR/GNAS/FLVCR1/APBA2/HOXB3/HOXC5/HOXC6/HOXD3/HSD17B2/CYR61/ISL1/RESP18/SMAD3/MEOX1/MEOX2/LEF1/SFRP2/TWIST1/FZD5/KDM2B	25
GO:0046058	cAMP metabolic process	11/311	150/17046	9.81E-05	0.00738	0.00588	NPFFR2/ADCY3/ADRB3/DRD4/GABBR1/GNAS/GPER1/HPCA/PALM/PDE4C/PDE7A	11
GO:0019219	regulation of nucleobase-containing compound metabolic process	94/311	3617/17046	0.00011	0.00771	0.00615	CDH13/MBNL2/C1D/ZBTB18/DMRT2/TBR1/NPFFR2/PSIP1/ZBED9/CIDEA/ZFP42/ADRB3/ZNF709/ZNF781/CITED4/RNF168/DNMT3A/DRD4/EGFR/EGR3/SP8/XRN2/ACIN1/FOXO1/SPG20/MTOR/GABBR1/GAPDHS/DKK3/GNAS/GPER1/ZBTB44/GTF2B/BRF1/GZMA/NRG1/HLX/HMGA1/HPCA/HOXB3/HOXC5/HOXC6/HOXD3/BARHL2/CYR61/IL6/ISL1/HILS1/SMAD3/ME1/MEF2D/MEOX1/MEOX2/NFYB/NTF3/PALM/LEF1/PGAM2/RIPK4/CYTL1/POMC/BNC2/PRMT6/ZNF532/CNOT11/PRKD1/CCAR2/SFRP2/TRA2B/SGK1/SOX9/TAF4B/TBP/TCEA1/TLE3/TRAF1/TWIST1/ZNF124/ZNF177/PAX8/FZD5/ZC3H14/ZNF606/SCRT1/HIST1H3A/SLA2/KDM2B/LOXL3/CBX2/GAS7/FADD/LIMD1/LDB2/H2AFY	94
GO:0009792	embryo development ending in birth or egg hatching	25/311	598/17046	0.00011	0.00771	0.00615	ZBTB18/DMRT2/COL11A1/ZFP42/ECE1/EGFR/GNAS/FLVCR1/APBA2/HOXB3/HOXC5/HOXC6/HOXD3/HSD17B2/CYR61/ISL1/RESP18/SMAD3/MEOX1/MEOX2/LEF1/SFRP2/TWIST1/FZD5/KDM2B	25
GO:0060485	mesenchyme development	13/311	206/17046	0.00011	0.00771	0.00615	EPHA3/NRG1/ISL1/SMAD3/MEOX1/LEF1/BNC2/S100A4/SFRP2/SOX9/ACTC1/TWIST1/LOXL3	13
GO:0051270	regulation of cellular component movement	27/311	672/17046	0.00011	0.00771	0.00615	CDH13/TBR1/EGFR/VASH1/GPER1/CYR61/IL6/SMAD3/MAP3K1/NOV/NTF3/LEF1/ANGPT4/ROBO4/PRKD1/SFRP2/SGK1/SOX9/STK10/PHLDA2/TWIST1/PTP4A1/COL18A1/SH3BGRL3/SCRT1/PARD6B/FADD	27
GO:0051216	cartilage development	12/311	179/17046	0.00011	0.00781	0.00623	COL11A1/CTGF/GNAS/HOXB3/HOXD3/CYR61/SMAD3/MEF2D/NOV/CYTL1/SFRP2/SOX9	12
GO:0065009	regulation of molecular function	72/311	2588/17046	0.00012	0.00825	0.00658	CDH3/NPFFR2/ADCY3/CTGF/ADRB3/DRD4/EGFR/EPHA3/RASA3/PPM1E/TBC1D9B/MTOR/GABBR1/RGS22/GNAS/GPER1/DOK7/GSTP1/GZMA/ANXA2/SERPIND1/KCNIP2/NRG1/HPCA/HSP90AA1/COL28A1/CYR61/IL6/ISL1/ITIH3/LCK/LLGL1/SMAD3/MAP3K1/NTF3/PALM/ARHGEF3/LEF1/ANGPT4/PIK3CG/RIPK4/CYTL1/PON1/ARHGEF10L/VAC14/PRKD1/PSMB4/CCAR2/PXN/RASGRF2/CCL17/SFRP2/SGK1/STK10/VAMP2/TCEA1/TIMP3/TNXB/TRAF1/TWIST1/YWHAG/CACNB2/CXCR4/FZD5/SH3BGRL3/SPINK7/FADD/ARHGAP29/H2AFY/ARHGEF10/USP6NL/RABGAP1L	72
GO:0048706	embryonic skeletal system development	10/311	129/17046	0.00013	0.00858	0.00684	DMRT2/COL11A1/GNAS/FLVCR1/HOXB3/HOXC5/HOXC6/HOXD3/SMAD3/TWIST1	10
GO:0009605	response to external stimulus	67/311	2366/17046	0.00013	0.00876	0.00698	CDH13/TBR1/COL11A1/ADRB3/COCH/NLRP6/DNMT3A/EGFR/EGR3/UNC13D/EPHA3/EPHB4/RASA3/ACIN1/FOXO1/LARP1/MTOR/GPER1/GSTP1/ANXA2/SERPIND1/NRG1/HLA-B/HLA-E/HMGA1/HSP90AA1/CYR61/IL6/ISL1/ACAT1/KRT15/LCK/SMAD3/MAP3K1/NUDT1/NOV/NTF3/LEF1/PIK3CG/IL20RB/POMC/PON1/PPP1CC/PRKD1/MASP1/PSMB4/TRPC7/RASGRF2/DEFB134/CCL17/SFRP2/CXCR5/SOX9/TIMP3/TLR5/TRPC4/UPP1/CACNB2/CXCR4/FZD5/NLRX1/UNC93B1/CAPS/HIST1H3A/FADD/LY86/ULK2	67
GO:0009889	regulation of biosynthetic process	98/311	3834/17046	0.00013	0.00876	0.00699	CDH3/CDH13/C1D/ZBTB18/DMRT2/TBR1/NPFFR2/PSIP1/ZBED9/CIDEA/ZFP42/CTGF/ADRB3/ZNF709/ZNF781/CITED4/RNF168/DNMT3A/DRD4/EGFR/EGR3/PATL2/SP8/XRN2/FOXO1/SPG20/LARP1/MTOR/GABBR1/DKK3/GNAS/GPER1/ZBTB44/GSTP1/GTF2B/BRF1/NRG1/HLX/HMGA1/HPCA/HOXB3/HOXC5/HOXC6/HOXD3/ACADL/HSP90AA1/BARHL2/CYR61/IL6/ISL1/HILS1/LTB/SMAD3/MEF2D/MEOX1/MEOX2/NFYB/NTF3/PALM/LEF1/RIPK4/CYTL1/POMC/BNC2/PIWIL2/PRMT6/ZNF532/CNOT11/PRKD1/METTL14/CCAR2/SFRP2/SGK1/SOX9/TAF4B/TBP/TCEA1/TLE3/TLR5/TRAF1/TWIST1/VARS/ZNF124/ZNF177/PAX8/FZD5/ZNF606/SCRT1/HIST1H3A/SLA2/KDM2B/LOXL3/CBX2/GAS7/FADD/LIMD1/LDB2/H2AFY	98
GO:1901362	organic cyclic compound biosynthetic process	102/311	4032/17046	0.00014	0.00881	0.00702	CDH3/CDH13/TCIRG1/C1D/ZBTB18/DMRT2/TBR1/NPFFR2/ADCY3/SLC27A2/PSIP1/ZBED9/CIDEA/ZFP42/CTGF/ADRB3/ADAL/ZNF709/ZNF781/CITED4/RNF168/DNMT3A/DRD4/EGFR/EGR3/ALAS1/SP8/XRN2/FOXO1/MTOR/GABBR1/AMPD2/DKK3/GLS2/GNAS/GPER1/ZBTB44/GTF2B/BRF1/NME7/NRG1/HLX/HMGA1/HPCA/HOXB3/HOXC5/HOXC6/HOXD3/HSD17B2/BARHL2/CYR61/IL6/ISL1/HILS1/SMAD3/ME1/MEF2D/MEOX1/MEOX2/NFYB/NTF3/PALM/LEF1/RIPK4/CYTL1/POMC/BNC2/PRMT6/ZNF532/CNOT11/PRKD1/CCAR2/RPL8/RPL29/SFRP2/SGK1/SOX9/TAF4B/TBP/TCEA1/TLE3/TRAF1/TWIST1/UPP1/YWHAG/ZNF124/ZNF177/PAX8/FZD5/ZNF606/SCRT1/HIST1H3A/SLA2/KDM2B/LOXL3/CBX2/GAS7/FADD/LIMD1/LDB2/SDR42E1/H2AFY	102

GO:0065008	regulation of biological quality	85/311	3225/17046	0.00016	0.01034	0.00825	CDH3/KCNMB2/TCIRG1/ZBTB18/ADCY3/CHRNA5/CIDEA/C15orf27/ZG16B/CTGF/ADRB3/FITM1/COCH/NLRP6/DRD4/ECE1/ACIN1/FOXO1/SPG20/FLNB/ATP11A/MTOR/AMPD2/DKK3/GLS2/GNAS/GPER1/FLVCR1/GSTP1/BRF1/ANXA2/SERPIND1/KCNIP2/ANXA6/ACADL/FMN1/BARHL2/IL6/ISL1/ATP9B/LCK/LTBP1/SMAD3/MAP3K1/MFI2/NOV/PALM/LEF1/ANGPT4/PDE4C/PIK3CG/IL20RB/CYTL1/POMC/SMPD3/SYBU/PRKD1/TRPC7/ACTR3B/METTL14/CCAR2/PXN/RASGRF2/SGK1/SOX9/VAMP2/TCEA1/TRPC4/VARS/YWHAG/PAX8/CXCR4/ZC3H14/RAB11FIP1/COLQ/SH3BGRL3/HIST1H3A/GAS7/FADD/LIMD1/LDB2/SMDT1/RAB3D/MTL5/ULK2	85
GO:0048705	skeletal system morphogenesis	13/311	215/17046	0.00017	0.01037	0.00827	COL11A1/CTGF/GNAS/FLVCR1/HOXB3/HOXD3/FMN1/SMAD3/MEF2D/BNC2/SFRP2/SOX9/TWIST1	13
GO:0048514	blood vessel morphogenesis	21/311	475/17046	0.00018	0.01091	0.0087	CDH13/CTGF/EGR3/EPHB4/VASH1/ANXA2/HOXB3/CYR61/IL6/ISL1/MEOX2/NOV/LEF1/ANGPT4/PIK3CG/ROBO4/PRKD1/SFRP2/TWIST1/FZD5/COL18A1	21
GO:0006796	phosphate-containing compound metabolic process	76/311	2808/17046	0.00018	0.01091	0.0087	BCKDK/TCIRG1/HCST/NPFFR2/ADCY3/ALPK2/APOA1BP/TRPM6/CTGF/ADRB3/FITM1/ADAL/NLRP6/DRD4/EGFR/EPHA3/EPHB4/RASA3/PPM1E/FOXO1/MTOR/GABBR1/GAK/GAPDHS/AMPD2/GNAS/PIGW/GPER1/DOK7/GSTP1/NME7/ANXA2/NRG1/HPCA/CYR61/IL6/ISL1/LCK/SMAD3/ME1/MAP3K1/NUDT1/NTF3/PALM/ANGPT4/PDE4C/PDE7A/PGAM2/PIGC/PIK3CG/RIPK4/PON1/LPCAT2/PPP1CC/SMPD3/VAC14/PRKD1/PSMB4/PXN/RASGRF2/CCL17/SFRP2/SGK1/CERK/SOX9/STK10/TNXB/TWIST1/UPP1/YWHAG/PTP4A1/CXCR4/FZD5/LIMD1/H2AFY/ULK2	76
GO:0019438	aromatic compound biosynthetic process	99/311	3915/17046	0.00019	0.01107	0.00882	CDH3/CDH13/TCIRG1/C1D/ZBTB18/DMRT2/TBR1/NPFFR2/ADCY3/PSIP1/ZBED9/CIDEA/ZFP42/CTGF/ADRB3/ADAL/ZNF709/ZNF781/CITED4/RNF168/DNMT3A/DRD4/EGFR/EGR3/ALAS1/SP8/XRN2/FOXO1/MTOR/GABBR1/AMPD2/DKK3/GLS2/GNAS/GPER1/ZBTB44/GTF2B/BRF1/NME7/NRG1/HLX/HMGA1/HPCA/HOXB3/HOXC5/HOXC6/HOXD3/BARHL2/CYR61/IL6/ISL1/HILS1/SMAD3/ME1/MEF2D/MEOX1/MEOX2/NFYB/NTF3/PALM/LEF1/RIPK4/CYTL1/POMC/BNC2/PRMT6/ZNF532/CNOT11/PRKD1/CCAR2/RPL8/RPL29/SFRP2/SGK1/SOX9/TAF4B/TBP/TCEA1/TLE3/TRAF1/TWIST1/UPP1/YWHAG/ZNF124/ZNF177/PAX8/FZD5/ZNF606/SCRT1/HIST1H3A/SLA2/KDM2B/LOXL3/CBX2/GAS7/FADD/LIMD1/LDB2/H2AFY	99
GO:0001501	skeletal system development	21/311	478/17046	0.0002	0.01148	0.00915	DMRT2/SLC38A10/COL11A1/CTGF/GNAS/FLVCR1/ANXA2/HOXB3/HOXC5/HOXC6/HOXD3/FMN1/CYR61/SMAD3/MEF2D/NOV/CYTL1/BNC2/SFRP2/SOX9/TWIST1	21
GO:0048646	anatomical structure formation involved in morphogenesis	37/311	1090/17046	0.0002	0.01148	0.00915	CDH13/DMRT2/TBR1/COL11A1/SCLT1/CTGF/EGR3/TMEM17/UNC13D/EPHB4/VASH1/TENM4/GNAS/ANXA2/HOXB3/FMN1/CYR61/IL6/ISL1/SMAD3/MEOX1/MEOX2/NOV/LEF1/CEND1/ANGPT4/PIK3CG/ROBO4/PRKD1/SFRP2/SOX9/ACTC1/TWIST1/PAX8/FZD5/COL18A1/KDM2B	37
GO:0009187	cyclic nucleotide metabolic process	12/311	191/17046	0.00021	0.01188	0.00947	NPFFR2/ADCY3/ADRB3/DRD4/GABBR1/AMPD2/GNAS/GPER1/HPCA/PALM/PDE4C/PDE7A	12
GO:0007155	cell adhesion	43/311	1343/17046	0.00021	0.01199	0.00956	CDH3/CDH13/CTGF/DSG3/EGFR/EGR3/UNC13D/EPHA3/EPHB4/MTOR/GNAS/IZUMO1/NRG1/HLA-E/HLX/HOXD3/COL28A1/FMN1/CYR61/IL6/CDHR4/LCK/SMAD3/MFI2/NEDD9/NOV/LEF1/PIK3CG/IL20RB/LIMS2/PCDHGC4/PCDHGB7/PCDHGA11/PXN/SFRP2/SOX9/STK10/TNXB/FZD5/COL18A1/SLA2/FADD/IL32	43
GO:0031279	regulation of cyclase activity	8/311	89/17046	0.00022	0.0121	0.00965	NPFFR2/ADRB3/DRD4/GABBR1/GNAS/GPER1/HPCA/PALM	8
GO:0023051	regulation of signaling	73/311	2685/17046	0.00022	0.0121	0.00965	CDH3/TSPAN5/CDH13/HCST/NPFFR2/CIDEA/APCDD1/CTGF/ADRB3/NLRP6/DRD4/ECE1/EGFR/RASA3/FOXO1/SPG20/MTOR/RGS22/DKK3/GNAS/GPER1/GSTP1/ANXA2/NRG1/CYR61/IL6/ISL1/LCK/LLGL1/LTBP1/SMAD3/MAP3K1/NOV/NTF3/PALM/ARHGEF3/LEF1/PDE4C/PIK3CG/POMC/ZDHHC13/PPP1CC/ARHGEF10L/SYBU/LIMS2/PRKD1/PSMB4/CCAR2/PXN/RASGRF2/S100A4/CCL17/SFRP2/SOX9/VAMP2/TLR5/TNXB/TRAF1/TWIST1/YWHAG/PAX8/CXCR4/FZD5/TMEM204/NLRX1/RAB11FIP1/SLA2/FADD/LIMD1/ARHGAP29/LY86/ARHGEF10/TELO2	73
GO:0022610	biological adhesion	43/311	1348/17046	0.00023	0.01246	0.00993	CDH3/CDH13/CTGF/DSG3/EGFR/EGR3/UNC13D/EPHA3/EPHB4/MTOR/GNAS/IZUMO1/NRG1/HLA-E/HLX/HOXD3/COL28A1/FMN1/CYR61/IL6/CDHR4/LCK/SMAD3/MFI2/NEDD9/NOV/LEF1/PIK3CG/IL20RB/LIMS2/PCDHGC4/PCDHGB7/PCDHGA11/PXN/SFRP2/SOX9/STK10/TNXB/FZD5/COL18A1/SLA2/FADD/IL32	43
GO:0009966	regulation of signal transduction	66/311	2366/17046	0.00023	0.01246	0.00993	CDH3/TSPAN5/CDH13/HCST/NPFFR2/CIDEA/APCDD1/CTGF/ADRB3/NLRP6/DRD4/EGFR/RASA3/FOXO1/SPG20/MTOR/RGS22/DKK3/GNAS/GPER1/GSTP1/ANXA2/NRG1/CYR61/IL6/ISL1/LCK/LLGL1/LTBP1/SMAD3/MAP3K1/NOV/NTF3/PALM/ARHGEF3/LEF1/PIK3CG/ZDHHC13/PPP1CC/ARHGEF10L/LIMS2/PRKD1/PSMB4/CCAR2/PXN/RASGRF2/S100A4/CCL17/SFRP2/SOX9/TLR5/TNXB/TRAF1/TWIST1/YWHAG/CXCR4/FZD5/TMEM204/NLRX1/SLA2/FADD/LIMD1/ARHGAP29/LY86/ARHGEF10/TELO2	66
GO:0031326	regulation of cellular biosynthetic process	96/311	3792/17046	0.00024	0.01246	0.00993	CDH3/CDH13/C1D/ZBTB18/DMRT2/TBR1/NPFFR2/PSIP1/ZBED9/CIDEA/ZFP42/ADRB3/ZNF709/ZNF781/CITED4/RNF168/DNMT3A/DRD4/EGFR/EGR3/PATL2/SP8/XRN2/FOXO1/SPG20/LARP1/MTOR/GABBR1/DKK3/GNAS/GPER1/ZBTB44/GTF2B/BRF1/NRG1/HLX/HMGA1/HPCA/HOXB3/HOXC5/HOXC6/HOXD3/ACADL/HSP90AA1/BARHL2/CYR61/IL6/ISL1/HILS1/LTB/SMAD3/MEF2D/MEOX1/MEOX2/NFYB/NTF3/PALM/LEF1/RIPK4/CYTL1/POMC/BNC2/PIWIL2/PRMT6/ZNF532/CNOT11/PRKD1/METTL14/CCAR2/SFRP2/SGK1/SOX9/TAF4B/TBP/TCEA1/TLE3/TLR5/TRAF1/TWIST1/VARS/ZNF124/ZNF177/PAX8/FZD5/ZNF606/SCRT1/HIST1H3A/SLA2/KDM2B/LOXL3/CBX2/GAS7/FADD/LIMD1/LDB2/H2AFY	96
GO:1902531	regulation of intracellular signal transduction	46/311	1479/17046	0.00024	0.01267	0.0101	CDH13/HCST/NPFFR2/CTGF/ADRB3/NLRP6/DRD4/EGFR/RASA3/FOXO1/MTOR/GNAS/GPER1/GSTP1/NRG1/CYR61/IL6/ISL1/LCK/MAP3K1/NOV/NTF3/ARHGEF3/PIK3CG/ZDHHC13/ARHGEF10L/PRKD1/PSMB4/CCAR2/PXN/RASGRF2/S100A4/CCL17/SFRP2/SOX9/TNXB/TWIST1/CXCR4/FZD5/NLRX1/SLA2/FADD/LIMD1/ARHGAP29/ARHGEF10/TELO2	46

GO:0034654	nucleobase-containing compound biosynthetic process	97/311	3846/17046	0.00025	0.01276	0.01018	CDH13/TCIRG1/C1D/ZBTB18/DMRT2/TBR1/NPFFR2/ADCY3/PSIP1/ZBED9/CIDEA/ZFP42/CTGF/ADRB3/ADAL/ZNF709/ZNF781/CITED4/RNF168/DNMT3A/DRD4/EGFR/EGR3/SP8/XRN2/FOXO1/MTOR/GABBR1/AMPD2/DKK3/GLS2/GNAS/GPER1/ZBTB44/GTF2B/BRF1/NME7/NRG1/HLX/HMGA1/HPCA/HOXB3/HOXC5/HOXC6/HOXD3/BARHL2/CYR61/IL6/ISL1/HILS1/SMAD3/ME1/MEF2D/MEOX1/MEOX2/NFYB/NTF3/PALM/LEF1/RIPK4/CYTL1/POMC/BNC2/PRMT6/ZNF532/CNOT11/PRKD1/CCAR2/RPL8/RPL29/SFRP2/SGK1/SOX9/TAF4B/TBP/TCEA1/TLE3/TRAF1/TWIST1/UPP1/YWHAG/ZNF124/ZNF177/PAX8/FZD5/ZNF606/SCRT1/HIST1H3A/SLA2/KDM2B/LOXL3/CBX2/GAS7/FADD/LIMD1/LDB2/H2AFY	97
GO:0051339	regulation of lyase activity	8/311	91/17046	0.00026	0.01299	0.01036	NPFFR2/ADRB3/DRD4/GABBR1/GNAS/GPER1/HPCA/PALM	8
GO:0006915	apoptotic process	51/311	1700/17046	0.00026	0.01299	0.01036	C1D/CIDEA/ANKRD9/CTGF/DNMT3A/DSG3/EGFR/EGR3/ACIN1/FOXO1/GLS2/GPER1/GSTP1/GZMA/NRG1/ANXA6/CYR61/IL6/ISL1/LCK/SMAD3/MEF2D/MAP3K1/NTF3/ARHGEF3/LEF1/DDX47/ANGPT4/PIK3CG/PLEC/LIMS2/PRKD1/PSMB4/CCAR2/RASGRF2/SFRP2/SGK1/SOX9/STK10/ACTC1/TRAF1/PHLDA2/TWIST1/YWHAG/PAX8/CXCR4/FZD5/COL18A1/KDM2B/FADD/LY86	51
GO:0006816	calcium ion transport	17/311	352/17046	0.00027	0.01331	0.01062	CLCA1/C15orf27/TRPM6/CTGF/TRPV3/DRD4/RASA3/CRACR2B/GPER1/ANXA6/LCK/PIK3CG/PRKD1/TRPC7/TRPC4/CACNB2/SMDT1	17
GO:0030878	thyroid gland development	4/311	18/17046	0.00027	0.01331	0.01062	HOXB3/HOXD3/SMAD3/PAX8	4
GO:0048519	negative regulation of biological process	103/311	4153/17046	0.00027	0.01331	0.01062	CDH3/CDH13/C1D/ZBTB18/B4GALT7/CIDEA/ANKRD9/APCDD1/CTGF/ADRB3/TRPV3/RNF168/NLRP6/DNMT3A/DRD4/EGFR/EGR3/PATL2/RASA3/PPM1E/VASH1/ACIN1/FOXO1/SPG20/MTOR/GABBR1/RGS22/FBXO2/DKK3/VPS4A/GNAS/GPER1/GSTP1/GZMA/ANXA2/SERPIND1/NRG1/HLA-B/HLA-E/HLX/HMGA1/HPCA/HOXB3/HOXC6/ACADL/COL28A1/CYR61/IL6/ISL1/ITIH3/HILS1/LCK/LTBP1/SMAD3/MFI2/MT1A/NOV/NTF3/PALM/LEF1/CEND1/ANGPT4/PDE4C/PIK3CG/IL20RB/POMC/PPP1CC/PIWIL2/PRMT6/CNOT11/LIMS2/PRKD1/MASP1/PSMB4/METTL14/CCAR2/CCL17/SFRP2/SOX9/TBP/ACTC1/TIMP3/TWIST1/YWHAG/ZNF177/PAX8/FZD5/NLRX1/ZC3H14/RAB11FIP1/COL18A1/SCRT1/HIST1H3A/SLA2/SPINK7/KDM2B/LOXL3/CBX2/GAS7/FADD/LIMD1/H2AFY/ULK2	103
GO:0009952	anterior/posterior pattern specification	12/311	197/17046	0.00028	0.01331	0.01062	DMRT2/HOXB3/HOXC5/HOXC6/HOXD3/SMAD3/MEOX1/MEOX2/LEF1/SFRP2/FZD5/KDM2B	12
GO:0018130	heterocycle biosynthetic process	98/311	3911/17046	0.00029	0.01372	0.01094	CDH13/TCIRG1/C1D/ZBTB18/DMRT2/TBR1/NPFFR2/ADCY3/PSIP1/ZBED9/CIDEA/ZFP42/CTGF/ADRB3/ADAL/ZNF709/ZNF781/CITED4/RNF168/DNMT3A/DRD4/EGFR/EGR3/ALAS1/SP8/XRN2/FOXO1/MTOR/GABBR1/AMPD2/DKK3/GLS2/GNAS/GPER1/ZBTB44/GTF2B/BRF1/NME7/NRG1/HLX/HMGA1/HPCA/HOXB3/HOXC5/HOXC6/HOXD3/BARHL2/CYR61/IL6/ISL1/HILS1/SMAD3/ME1/MEF2D/MEOX1/MEOX2/NFYB/NTF3/PALM/LEF1/RIPK4/CYTL1/POMC/BNC2/PRMT6/ZNF532/CNOT11/PRKD1/CCAR2/RPL8/RPL29/SFRP2/SGK1/SOX9/TAF4B/TBP/TCEA1/TLE3/TRAF1/TWIST1/UPP1/YWHAG/ZNF124/ZNF177/PAX8/FZD5/ZNF606/SCRT1/HIST1H3A/SLA2/KDM2B/LOXL3/CBX2/GAS7/FADD/LIMD1/LDB2/H2AFY	98
GO:0006793	phosphorus metabolic process	76/311	2851/17046	0.00029	0.01373	0.01095	BCKDK/TCIRG1/HCST/NPFFR2/ADCY3/ALPK2/APOA1BP/TRPM6/CTGF/ADRB3/FITM1/ADAL/NLRP6/DRD4/EGFR/EPHA3/EPHB4/RASA3/PPM1E/FOXO1/MTOR/GABBR1/GAK/GAPDHS/AMPD2/GNAS/PIGW/GPER1/DOK7/GSTP1/NME7/ANXA2/NRG1/HPCA/CYR61/IL6/ISL1/LCK/SMAD3/ME1/MAP3K1/NUDT1/NTF3/PALM/ANGPT4/PDE4C/PDE7A/PGAM2/PIGC/PIK3CG/RIPK4/PON1/LPCAT2/PPP1CC/SMPD3/VAC14/PRKD1/PSMB4/PXN/RASGRF2/CCL17/SFRP2/SGK1/CERK/SOX9/STK10/TNXB/TWIST1/UPP1/YWHAG/PTP4A1/CXCR4/FZD5/LIMD1/H2AFY/ULK2	76
GO:0007267	cell-cell signaling	38/311	1154/17046	0.0003	0.01373	0.01095	KCNMB2/NPFFR2/ADCY3/CHRNA5/CTGF/ADRB3/DRD4/EGR3/GABBR1/GJA3/GJB2/GLS2/GNAS/GPER1/KCNIP2/APBA2/CYR61/IL6/ISL1/LTB/NOV/NTF3/PDE4C/POMC/SMPD3/SYBU/RASGRF2/CCL17/SFRP2/SOX9/VAMP2/YWHAG/CACNB2/PAX8/FZD5/RAB11FIP1/COLQ/RAB3D	38
GO:0002062	chondrocyte differentiation	8/311	93/17046	0.0003	0.01373	0.01095	COL11A1/CTGF/SMAD3/MEF2D/NOV/CYTL1/SFRP2/SOX9	8
GO:0014031	mesenchymal cell development	10/311	144/17046	0.00031	0.01435	0.01145	EPHA3/NRG1/ISL1/SMAD3/LEF1/S100A4/SFRP2/SOX9/TWIST1/LOXL3	10
GO:0012501	programmed cell death	51/311	1718/17046	0.00033	0.01493	0.01191	C1D/CIDEA/ANKRD9/CTGF/DNMT3A/DSG3/EGFR/EGR3/ACIN1/FOXO1/GLS2/GPER1/GSTP1/GZMA/NRG1/ANXA6/CYR61/IL6/ISL1/LCK/SMAD3/MEF2D/MAP3K1/NTF3/ARHGEF3/LEF1/DDX47/ANGPT4/PIK3CG/PLEC/LIMS2/PRKD1/PSMB4/CCAR2/RASGRF2/SFRP2/SGK1/SOX9/STK10/ACTC1/TRAF1/PHLDA2/TWIST1/YWHAG/PAX8/CXCR4/FZD5/COL18A1/KDM2B/FADD/LY86	51
GO:0009790	embryo development	33/311	958/17046	0.00034	0.01535	0.01224	ZBTB18/DMRT2/COL11A1/ZFP42/DNMT3A/ECE1/EGFR/SP8/TENM4/GNAS/FLVCR1/NRG1/HLX/APBA2/HOXB3/HOXC5/HOXC6/HOXD3/HSD17B2/CYR61/ISL1/RESP18/SMAD3/MEOX1/MEOX2/LEF1/SFRP2/SOX9/PHLDA2/TWIST1/PAX8/FZD5/KDM2B	33
GO:0016049	cell growth	19/311	429/17046	0.00036	0.01574	0.01255	CDH13/CTGF/XRN2/SPG20/MTOR/NRG1/BARHL2/CYR61/IL6/SMAD3/NOV/LEF1/PRMT6/BIN3/CCAR2/SFRP2/SGK1/SOX9/ULK2	19
GO:0010646	regulation of cell communication	73/311	2731/17046	0.00037	0.0158	0.0126	CDH3/TSPAN5/CDH13/HCST/NPFFR2/CIDEA/APCDD1/CTGF/ADRB3/NLRP6/DRD4/EGFR/RASA3/FOXO1/SPG20/LARP1/MTOR/RGS22/DKK3/GNAS/GPER1/GSTP1/ANXA2/NRG1/CYR61/IL6/ISL1/LCK/LLGL1/LTBP1/SMAD3/MAP3K1/NOV/NTF3/PALM/ARHGEF3/LEF1/PDE4C/PIK3CG/POMC/ZDHHC13/PPP1CC/ARHGEF10L/SYBU/LIMS2/PRKD1/PSMB4/CCAR2/PXN/RASGRF2/S100A4/CCL17/SFRP2/SOX9/VAMP2/TLR5/TNXB/TRAF1/TWIST1/YWHAG/PAX8/CXCR4/FZD5/TMEM204/NLRX1/RAB11FIP1/SLA2/FADD/LIMD1/ARHGAP29/LY86/ARHGEF10/TELO2	73

GO:1903035	negative regulation of response to wounding	10/311	147/17046	0.00037	0.0158	0.0126	NLRP6/GPER1/GSTP1/ANXA2/ISL1/SMAD3/NOV/IL20RB/PSMB4/NLRX1	10
GO:0071840	cellular component organization or biogenesis	133/311	5713/17046	0.00038	0.0158	0.0126	CDH3/TSPAN5/CDH13/TCIRG1/C1D/TBR1/SEPT9/ADCY3/PSIP1/B4GALT7/CIDEA/FRMD6/APOA1BP/COL11A1/SCLT1/ZFP42/CTGF/FITM1/WBP2NL/RNF168/COCH/DNMT3A/DSG3/EGFR/PATL2/TMEM17/EML1/UNC13D/EPHA3/EPHB4/ALAS1/RASA3/PPM1E/ACIN1/LIMCH1/SPG20/FLNB/ATP11A/MTOR/TENM4/GJB2/GLS2/VPS4A/PIGW/IZUMO1/GPER1/NME7/ANXA2/KCNIP2/NRG1/ANXA6/HMGA1/HPCA/ACADL/HSP90AA1/COL28A1/FMN1/BARHL2/CYR61/IL6/ISL1/HILS1/ATP9B/ACAT1/KRT15/INSC/LLGL1/LOX/LTBP1/SMAD3/ME1/MAP3K1/MFI2/NEDD9/NOV/NTF3/PALM/LEF1/DDX47/ANGPT4/PLEC/LPCAT2/PIWIL2/PRMT6/LIMS2/PRKD1/BIN3/PSMB4/TRPC7/ACTR3B/CCAR2/PXN/RASGRF2/EXOC4/RPL8/RPL29/S100A4/S100A6/SFRP2/SGK1/MICAL1/SOX9/VAMP2/ACTC1/TNXB/TRAF1/TRPC4/TWIST1/YWHAG/CACNB2/PAX8/CXCR4/FZD5/COL18A1/CAPS/COLQ/HIST1H2BM/SH3BGRL3/HIST1H3A/PARD6B/KDM2B/LOXL3/MGARP/CBX2/GAS7/FADD/LIMD1/MAP7/LDB2/H2AFY/ARHGEF10/ULK2/USP6NL	133
GO:0008219	cell death	53/311	1816/17046	0.00038	0.0158	0.0126	C1D/CIDEA/ANKRD9/CTGF/DNMT3A/DSG3/EGFR/EGR3/ACIN1/FOXO1/GLS2/GPER1/GSTP1/GZMA/NRG1/ANXA6/CYR61/IL6/ISL1/LCK/SMAD3/MEF2D/MAP3K1/MEOX2/NOV/NTF3/ARHGEF3/LEF1/DDX47/ANGPT4/PIK3CG/PLEC/LIMS2/PRKD1/PSMB4/CCAR2/RASGRF2/SFRP2/SGK1/SOX9/STK10/ACTC1/TRAF1/PHLDA2/TWIST1/YWHAG/PAX8/CXCR4/FZD5/COL18A1/KDM2B/FADD/LY86	53
GO:0016265	death	53/311	1816/17046	0.00038	0.0158	0.0126	C1D/CIDEA/ANKRD9/CTGF/DNMT3A/DSG3/EGFR/EGR3/ACIN1/FOXO1/GLS2/GPER1/GSTP1/GZMA/NRG1/ANXA6/CYR61/IL6/ISL1/LCK/SMAD3/MEF2D/MAP3K1/MEOX2/NOV/NTF3/ARHGEF3/LEF1/DDX47/ANGPT4/PIK3CG/PLEC/LIMS2/PRKD1/PSMB4/CCAR2/RASGRF2/SFRP2/SGK1/SOX9/STK10/ACTC1/TRAF1/PHLDA2/TWIST1/YWHAG/PAX8/CXCR4/FZD5/COL18A1/KDM2B/FADD/LY86	53
GO:0048864	stem cell development	10/311	148/17046	0.00039	0.01631	0.013	EPHA3/NRG1/ISL1/SMAD3/LEF1/S100A4/SFRP2/SOX9/TWIST1/LOXL3	10
GO:0050896	response to stimulus	169/311	7634/17046	0.0004	0.01654	0.01319	CDH3/TSPAN5/CDH13/KCNMB2/TCIRG1/TBR1/HCST/NPFFR2/ADCY3/PSIP1/PTH2/CHRNA5/CIDEA/COL11A1/TRPM6/APCDD1/CTGF/ADRB3/TRPV3/CITED4/RNF168/COCH/NLRP6/DNMT3A/DRD4/EGFR/EGR3/TMEM17/UNC13D/EPHA3/EPHB4/RASA3/PPM1E/VASH1/ACIN1/FOXO1/SPG20/FLNB/LARP1/MTOR/GABBR1/TENM4/RGS22/FBXO2/GJA3/GJB2/SDCBP2/DKK3/GPR162/GNAS/GPER1/GSTP1/NME7/GZMA/ANXA2/SERPIND1/KCNIP2/NRG1/ANXA6/HLA-B/HLA-E/HLA-F/HLX/HMGA1/HPCA/HOXD3/HSD17B2/HSP90AA1/HTR5A/CYR61/IL6/ISL1/ACAT1/KRT15/LCK/LLGL1/LOX/LTB/LTBP1/SMAD3/ME1/MAP3K1/MT1A/NUDT1/NEDD9/NMBR/NOV/NTF3/PALM/ARHGEF3/LEF1/DDX47/ANGPT4/PDE4C/PDE7A/PGAM2/PIK3CG/IL20RB/CYTL1/POMC/PON1/ZDHHC13/PPP1CC/ARHGEF10L/PRMT6/WDR33/SYBU/LIMS2/VAC14/PRKD1/BIN3/MASP1/PSMB4/TRPC7/LPAR5/CCAR2/PTPRCAP/PXN/RASGRF2/DEFB134/S100A4/S100A6/CCL17/SFRP2/CXCR5/SGK1/SYNDIG1L/MICAL1/SOX9/STK10/VAMP2/TCEA1/ACTC1/TIMP3/TLE3/TLR5/TNXB/TRAF1/TRPC4/TWIST1/UPP1/YWHAG/CACNB2/PAX8/CXCR4/FZD5/TMEM204/NLRX1/COL18A1/UNC93B1/CAPS/HIST1H3A/SLA2/IL1F10/MGARP/FADD/LIMD1/MAP7/ZFAND2A/RCSD1/IL32/ARHGAP29/LY86/RAB3D/SMAD5-AS1/MTL5/ARHGEF10/ULK2/TELO2	169
GO:0070838	divalent metal ion transport	18/311	399/17046	0.00041	0.01655	0.0132	CLCA1/C15orf27/TRPM6/CTGF/TRPV3/DRD4/RASA3/CRACR2B/GPER1/ANXA6/LCK/PIK3CG/ZDHHC13/PRKD1/TRPC7/TRPC4/CACNB2/SMDT1	18
GO:0040007	growth	31/311	887/17046	0.00041	0.01655	0.0132	CDH13/CTGF/ADRB3/XRN2/SPG20/MTOR/TENM4/GNAS/FLVCR1/NRG1/HLX/APBA2/FMN1/BARHL2/CYR61/IL6/SMAD3/MT1A/NEDD9/NOV/LEF1/BNC2/PRMT6/BIN3/CCAR2/SFRP2/SGK1/SOX9/TIMP3/COLQ/ULK2	31
GO:0006171	cAMP biosynthetic process	9/311	123/17046	0.00043	0.01706	0.0136	NPFFR2/ADCY3/ADRB3/DRD4/GABBR1/GNAS/GPER1/HPCA/PALM	9
GO:0072511	divalent inorganic cation transport	18/311	402/17046	0.00045	0.01769	0.01411	CLCA1/C15orf27/TRPM6/CTGF/TRPV3/DRD4/RASA3/CRACR2B/GPER1/ANXA6/LCK/PIK3CG/ZDHHC13/PRKD1/TRPC7/TRPC4/CACNB2/SMDT1	18
GO:0002480	antigen processing and presentation of exogenous peptide antigen via MHC class I, TAP-independent	3/311	9/17046	0.00047	0.01814	0.01447	HLA-B/HLA-E/HLA-F	3
GO:0002862	negative regulation of inflammatory response to antigenic stimulus	3/311	9/17046	0.00047	0.01814	0.01447	NLRP6/IL20RB/PSMB4	3

GO:0019220	regulation of phosphate metabolic process	47/311	1568/17046	0.00047	0.01823	0.01454	NPFFR2/ADCY3/CTGF/ADRB3/NLRP6/DRD4/EGFR/RASA3/PPM1E/FOXO1/MTOR/GABBR1/GAPDHS/GNAS/GPER1/DOK7/GSTP1/ANXA2/NRG1/HPCA/CYR61/IL6/ISL1/LCK/SMAD3/ME1/MAP3K1/NTF3/PALM/ANGPT4/PGAM2/PIK3CG/VAC14/PRKD1/PSMB4/PXN/RASGRF2/CCL17/SFRP2/SOX9/STK10/TNXB/TWIST1/YWHAG/CXCR4/FZD5/H2AFY	47
GO:0052652	cyclic purine nucleotide metabolic process	10/311	152/17046	0.00048	0.01838	0.01466	NPFFR2/ADCY3/ADRB3/DRD4/GABBR1/AMPD2/GNAS/GPER1/HPCA/PALM	10
GO:0001837	epithelial to mesenchymal transition	8/311	100/17046	0.00049	0.01838	0.01466	EPHA3/SMAD3/LEF1/S100A4/SFRP2/SOX9/TWIST1/LOXL3	8
GO:0048583	regulation of response to stimulus	84/311	3283/17046	0.00049	0.01838	0.01466	CDH3/TSPAN5/CDH13/TBR1/HCST/NPFFR2/CIDEA/APCDD1/CTGF/ADRB3/RNF168/COCH/NLRP6/DRD4/EGFR/UNC13D/RASA3/VASH1/ACIN1/FOXO1/SPG20/LARP1/MTOR/RGS22/DKK3/GNAS/GPER1/GSTP1/ANXA2/NRG1/HLA-B/HLA-E/HLA-F/HLX/HMGA1/HSP90AA1/CYR61/IL6/ISL1/LCK/LLGL1/LTBP1/SMAD3/MAP3K1/NOV/NTF3/PALM/ARHGEF3/LEF1/PIK3CG/IL20RB/ZDHHC13/PPP1CC/ARHGEF10L/SYBU/LIMS2/PRKD1/MASP1/PSMB4/CCAR2/PXN/RASGRF2/S100A4/CCL17/SFRP2/SOX9/TLR5/TNXB/TRAF1/TWIST1/YWHAG/CXCR4/FZD5/TMEM204/NLRX1/UNC93B1/HIST1H3A/SLA2/FADD/LIMD1/ARHGAP29/LY86/ARHGEF10/TELO2	84
GO:0019932	second-messenger-mediated signaling	12/311	210/17046	0.00049	0.01838	0.01466	CDH13/ADCY3/DRD4/EGFR/GNAS/NRG1/HPCA/HTR5A/PDE7A/SOX9/CXCR4/SLA2	12
GO:0016043	cellular component organization	130/311	5594/17046	0.00051	0.01865	0.01487	CDH3/TSPAN5/CDH13/TCIRG1/TBR1/SEPT9/ADCY3/PSIP1/B4GALT7/CIDEA/FRMD6/APOA1BP/COL11A1/SCLT1/ZFP42/CTGF/FITM1/WBP2NL/RNF168/COCH/DNMT3A/DSG3/EGFR/PATL2/TMEM17/EML1/UNC13D/EPHA3/EPHB4/ALAS1/RASA3/PPM1E/ACIN1/LIMCH1/SPG20/FLNB/ATP11A/MTOR/TENM4/GJB2/GLS2/VPS4A/PIGW/IZUMO1/GPER1/NME7/ANXA2/KCNIP2/NRG1/ANXA6/HMGA1/HPCA/ACADL/HSP90AA1/COL28A1/FMN1/BARHL2/CYR61/IL6/ISL1/HILS1/ATP9B/ACAT1/KRT15/INSC/LLGL1/LOX/LTBP1/SMAD3/ME1/MAP3K1/MFI2/NEDD9/NOV/NTF3/PALM/LEF1/ANGPT4/PLEC/LPCAT2/PIWIL2/PRMT6/LIMS2/PRKD1/BIN3/PSMB4/TRPC7/ACTR3B/CCAR2/PXN/RASGRF2/EXOC4/RPL8/RPL29/S100A4/S100A6/SFRP2/SGK1/MICAL1/SOX9/VAMP2/ACTC1/TNXB/TRAF1/TRPC4/TWIST1/YWHAG/CACNB2/PAX8/CXCR4/FZD5/COL18A1/CAPS/COLQ/HIST1H2BM/SH3BGRL3/HIST1H3A/PARD6B/KDM2B/LOXL3/MGARP/CBX2/GAS7/FADD/LIMD1/MAP7/H2AFY/ARHGEF10/ULK2/USP6NL	130
GO:0009190	cyclic nucleotide biosynthetic process	10/311	153/17046	0.00051	0.01865	0.01487	NPFFR2/ADCY3/ADRB3/DRD4/GABBR1/AMPD2/GNAS/GPER1/HPCA/PALM	10
GO:0006468	protein phosphorylation	49/311	1662/17046	0.00051	0.01865	0.01487	BCKDK/HCST/NPFFR2/ADCY3/ALPK2/TRPM6/CTGF/ADRB3/NLRP6/DRD4/EGFR/EPHA3/EPHB4/RASA3/PPM1E/FOXO1/MTOR/GAK/GPER1/DOK7/GSTP1/ANXA2/NRG1/CYR61/IL6/ISL1/LCK/SMAD3/MAP3K1/NTF3/ANGPT4/PIK3CG/RIPK4/PRKD1/PSMB4/PXN/RASGRF2/CCL17/SFRP2/SGK1/SOX9/STK10/TNXB/TWIST1/YWHAG/CXCR4/FZD5/H2AFY/ULK2	49
GO:0051174	regulation of phosphorus metabolic process	47/311	1579/17046	0.00055	0.01981	0.0158	NPFFR2/ADCY3/CTGF/ADRB3/NLRP6/DRD4/EGFR/RASA3/PPM1E/FOXO1/MTOR/GABBR1/GAPDHS/GNAS/GPER1/DOK7/GSTP1/ANXA2/NRG1/HPCA/CYR61/IL6/ISL1/LCK/SMAD3/ME1/MAP3K1/NTF3/PALM/ANGPT4/PGAM2/PIK3CG/VAC14/PRKD1/PSMB4/PXN/RASGRF2/CCL17/SFRP2/SOX9/STK10/TNXB/TWIST1/YWHAG/CXCR4/FZD5/H2AFY	47
GO:0044093	positive regulation of molecular function	49/311	1672/17046	0.00059	0.02091	0.01668	CDH3/ADCY3/CTGF/ADRB3/DRD4/EGFR/RASA3/TBC1D9B/RGS22/GNAS/GPER1/DOK7/ANXA2/NRG1/HPCA/CYR61/IL6/ISL1/LCK/LLGL1/SMAD3/MAP3K1/NTF3/ARHGEF3/ANGPT4/PIK3CG/RIPK4/CYTL1/PON1/ARHGEF10L/PRKD1/PSMB4/PXN/RASGRF2/CCL17/SFRP2/SGK1/STK10/TCEA1/TRAF1/TWIST1/CXCR4/FZD5/SH3BGRL3/FADD/ARHGAP29/ARHGEF10/USP6NL/RABGAP1L	49
GO:0048762	mesenchymal cell differentiation	10/311	156/17046	0.00059	0.02091	0.01668	EPHA3/NRG1/ISL1/SMAD3/LEF1/S100A4/SFRP2/SOX9/TWIST1/LOXL3	10
GO:0009628	response to abiotic stimulus	35/311	1071/17046	0.0006	0.02091	0.01668	NPFFR2/PSIP1/COL11A1/CTGF/ADRB3/TRPV3/RNF168/DNMT3A/DRD4/EGFR/FOXO1/MTOR/GJA3/HPCA/HSP90AA1/IL6/LCK/SMAD3/MAP3K1/PALM/ANGPT4/PPP1CC/CCAR2/SFRP2/SOX9/TIMP3/TLR5/TWIST1/CXCR4/COL18A1/MGARP/FADD/LIMD1/MAP7/RCSD1	35
GO:0022008	neurogenesis	46/311	1548/17046	0.00066	0.02289	0.01825	ZBTB18/TBR1/SCLT1/APCDD1/DNMT3A/EGFR/EML1/EPHA3/EPHB4/RASA3/SPG20/TENM4/GPER1/KCNIP2/NRG1/HOXB3/HOXD3/HSP90AA1/FMN1/BARHL2/IL6/ISL1/LLGL1/NTF3/PALM/LEF1/CEND1/PPP1CC/PRKD1/PSMB4/TRPC7/RASGRF2/S100A6/SGK1/SOX9/TRPC4/TWIST1/YWHAG/CACNB2/CXCR4/FZD5/SCRT1/PARD6B/GAS7/ARHGEF10/ULK2	46
GO:0045667	regulation of osteoblast differentiation	8/311	105/17046	0.00068	0.02304	0.01838	GNAS/CYR61/IL6/SMAD3/PRKD1/SFRP2/TWIST1/LIMD1	8

GO:0030199	collagen fibril organization	5/311	39/17046	0.00068	0.02304	0.01838	COL11A1/ANXA2/LOX/SFRP2/TNXB	5
GO:0051496	positive regulation of stress fiber assembly	5/311	39/17046	0.00068	0.02304	0.01838	CTGF/PPM1E/MTOR/SMAD3/ARHGEF10	5
GO:0050793	regulation of developmental process	55/311	1953/17046	0.00068	0.02304	0.01838	CDH3/DMRT2/TBR1/CTGF/ADRB3/COCH/EGR3/UNC13D/EPHA3/VASH1/ACIN1/FOXO1/SPG20/MTOR/TENM4/GNAS/GPER1/FLVCR1/NRG1/HLA-B/HLX/HMGA1/HOXB3/HOXD3/BARHL2/CYR61/IL6/ISL1/LCK/SMAD3/MFI2/NOV/NTF3/PALM/LEF1/CEND1/ANGPT4/LIMS2/PRKD1/PXN/CCL17/SFRP2/SOX9/PHLDA2/TWIST1/YWHAG/PAX8/CXCR4/COLQ/SCRT1/GAS7/FADD/LIMD1/H2AFY/ULK2	55
GO:0010638	positive regulation of organelle organization	20/311	489/17046	0.00069	0.02304	0.01838	CTGF/PPM1E/MTOR/GLS2/VPS4A/GPER1/ANXA2/NRG1/FMN1/ISL1/SMAD3/MAP3K1/NTF3/PIWIL2/PRKD1/ACTR3B/YWHAG/FZD5/MGARP/ARHGEF10	20
GO:0035270	endocrine system development	9/311	132/17046	0.00071	0.02357	0.0188	FOXO1/DKK3/HOXB3/HOXD3/IL6/ISL1/SMAD3/SOX9/PAX8	9
GO:0051336	regulation of hydrolase activity	37/311	1166/17046	0.00071	0.02357	0.0188	ADCY3/CTGF/EGFR/EPHA3/RASA3/TBC1D9B/MTOR/RGS22/GNAS/GPER1/GZMA/SERPIND1/HPCA/COL28A1/CYR61/IL6/ITIH3/LCK/LLGL1/SMAD3/NTF3/ARHGEF3/LEF1/ARHGEF10L/PRKD1/RASGRF2/CCL17/SFRP2/TCEA1/TIMP3/SH3BGRL3/SPINK7/FADD/ARHGAP29/ARHGEF10/USP6NL/RABGAP1L	37
GO:0044271	cellular nitrogen compound biosynthetic process	105/311	4363/17046	0.00073	0.02391	0.01907	CDH13/TCIRG1/C1D/ZBTB18/DMRT2/TBR1/NPFFR2/ADCY3/PSIP1/ZBED9/CIDEA/ZFP42/CTGF/ADRB3/ADAL/ZNF709/ZNF781/CITED4/RNF168/DNMT3A/DRD4/EGFR/EGR3/PATL2/ALAS1/SP8/XRN2/FOXO1/LARP1/MTOR/GABBR1/AMPD2/DKK3/GLS2/GNAS/GPER1/ZBTB44/GTF2B/BRF1/NME7/NRG1/HLX/HMGA1/HPCA/HOXB3/HOXC5/HOXC6/HOXD3/HSP90AA1/BARHL2/CYR61/IL6/ISL1/HILS1/SMAD3/ME1/MEF2D/MEOX1/MEOX2/NFYB/NTF3/PALM/LEF1/RIPK4/CYTL1/POMC/BNC2/PIWIL2/PRMT6/ZNF532/CNOT11/PRKD1/METTL14/CCAR2/RPL8/RPL29/SFRP2/SGK1/SOX9/TAF4B/TBP/TCEA1/TLE3/TLR5/TRAF1/TWIST1/UPP1/VARS/YWHAG/ZNF124/ZNF177/PAX8/FZD5/ZNF606/SCRT1/HIST1H3A/SLA2/KDM2B/LOXL3/CBX2/GAS7/FADD/LIMD1/LDB2/H2AFY	105
GO:0009260	ribonucleotide biosynthetic process	13/311	251/17046	0.00074	0.02391	0.01907	TCIRG1/NPFFR2/ADCY3/ADRB3/DRD4/GABBR1/AMPD2/GNAS/GPER1/NME7/HPCA/PALM/UPP1	13
GO:0048699	generation of neurons	44/311	1471/17046	0.00076	0.0244	0.01946	ZBTB18/TBR1/SCLT1/DNMT3A/EGFR/EML1/EPHA3/EPHB4/RASA3/SPG20/TENM4/GPER1/KCNIP2/NRG1/HOXB3/HOXD3/HSP90AA1/FMN1/BARHL2/IL6/ISL1/LLGL1/NTF3/PALM/LEF1/CEND1/PPP1CC/PRKD1/PSMB4/TRPC7/RASGRF2/S100A6/SGK1/SOX9/TRPC4/TWIST1/YWHAG/CACNB2/CXCR4/FZD5/SCRT1/PARD6B/GAS7/ULK2	44
GO:0042325	regulation of phosphorylation	41/311	1341/17046	0.00076	0.0244	0.01946	NPFFR2/ADCY3/CTGF/ADRB3/NLRP6/DRD4/EGFR/RASA3/PPM1E/FOXO1/MTOR/GAPDHS/GPER1/DOK7/GSTP1/ANXA2/NRG1/CYR61/IL6/ISL1/LCK/SMAD3/MAP3K1/NTF3/ANGPT4/PIK3CG/VAC14/PRKD1/PSMB4/PXN/RASGRF2/CCL17/SFRP2/SOX9/STK10/TNXB/TWIST1/YWHAG/CXCR4/FZD5/H2AFY	41
GO:0016310	phosphorylation	55/311	1964/17046	0.00078	0.02474	0.01973	BCKDK/HCST/NPFFR2/ADCY3/ALPK2/TRPM6/CTGF/ADRB3/NLRP6/DRD4/EGFR/EPHA3/EPHB4/RASA3/PPM1E/FOXO1/MTOR/GAK/GAPDHS/GPER1/DOK7/GSTP1/NME7/ANXA2/NRG1/CYR61/IL6/ISL1/LCK/SMAD3/MAP3K1/NTF3/ANGPT4/PGAM2/PIK3CG/RIPK4/VAC14/PRKD1/PSMB4/PXN/RASGRF2/CCL17/SFRP2/SGK1/CERK/SOX9/STK10/TNXB/TWIST1/YWHAG/CXCR4/FZD5/LIMD1/H2AFY/ULK2	55
GO:0002460	adaptive immune response based on somatic recombination of immune receptors built from immunoglobulin superfamily domains	11/311	191/17046	0.00079	0.02474	0.01973	RNF168/UNC13D/HLA-B/HLA-E/HLX/IL6/LEF1/IL20RB/FZD5/SLA2/FADD	11
GO:0046390	ribose phosphate biosynthetic process	13/311	253/17046	0.00079	0.02474	0.01973	TCIRG1/NPFFR2/ADCY3/ADRB3/DRD4/GABBR1/AMPD2/GNAS/GPER1/NME7/HPCA/PALM/UPP1	13

GO:0001932	regulation of protein phosphorylation	39/311	1258/17046	0.0008	0.02474	0.01973	NPFFR2/ADCY3/CTGF/ADRB3/NLRP6/DRD4/EGFR/RASA3/PPM1E/FOXO1/MTOR/GPER1/DOK7/GSTP1/ANXA2/NRG1/CYR61/IL6/ISL1/LCK/SMAD3/MAP3K1/NTF3/ANGPT4/PIK3CG/PRKD1/PSMB4/PXN/RASGRF2/CCL17/SFRP2/SOX9/STK10/TNXB/TWIST1/YWHAG/CXCR4/FZD5/H2AFY	39
GO:0007389	pattern specification process	18/311	423/17046	0.00081	0.02486	0.01982	DMRT2/TBR1/SP8/NME7/HOXB3/HOXC5/HOXC6/HOXD3/ISL1/SMAD3/MEOX1/MEOX2/LEF1/SFRP2/TRA2B/PAX8/FZD5/KDM2B	18
GO:0009887	organ morphogenesis	30/311	884/17046	0.00082	0.02501	0.01994	TBR1/COL11A1/CTGF/EGFR/EPHB4/GNAS/FLVCR1/NRG1/HLX/HOXB3/HOXD3/FMN1/CYR61/IL6/ISL1/SMAD3/MEF2D/LEF1/BNC2/LIMS2/SFRP2/SOX9/ACTC1/TLE3/PHLDA2/TWIST1/PAX8/FZD5/COL18A1/KDM2B	30
GO:0085029	extracellular matrix assembly	4/311	24/17046	0.00087	0.02631	0.02098	LOX/SMAD3/SOX9/TNXB	4
GO:0010941	regulation of cell death	42/311	1395/17046	0.00089	0.02668	0.02128	CIDEA/ANKRD9/CTGF/DNMT3A/EGFR/EGR3/ACIN1/FOXO1/GLS2/GPER1/GSTP1/GZMA/NRG1/CYR61/IL6/ISL1/LCK/SMAD3/MAP3K1/NOV/NTF3/ARHGEF3/LEF1/ANGPT4/PIK3CG/LIMS2/PRKD1/PSMB4/CCAR2/RASGRF2/SFRP2/SGK1/SOX9/STK10/ACTC1/TRAF1/TWIST1/YWHAG/PAX8/COL18A1/KDM2B/FADD	42
GO:0035413	positive regulation of catenin import into nucleus	3/311	11/17046	0.00089	0.02668	0.02128	EGFR/SMAD3/SFRP2	3
GO:0045595	regulation of cell differentiation	41/311	1356/17046	0.00095	0.02819	0.02248	TBR1/CTGF/EGR3/UNC13D/EPHA3/ACIN1/FOXO1/SPG20/MTOR/TENM4/GNAS/GPER1/NRG1/HLA-B/HLX/HOXB3/HOXD3/BARHL2/CYR61/IL6/ISL1/LCK/SMAD3/MFI2/NOV/NTF3/PALM/LEF1/PRKD1/CCL17/SFRP2/SOX9/TWIST1/YWHAG/PAX8/CXCR4/SCRT1/FADD/LIMD1/H2AFY/ULK2	41
GO:0044700	single organism signaling	129/311	5624/17046	0.00096	0.02844	0.02268	CDH3/TSPAN5/CDH13/KCNMB2/TCIRG1/HCST/NPFFR2/ADCY3/PTH2/CHRNA5/CIDEA/APCDD1/CTGF/ADRB3/NLRP6/DRD4/ECE1/EGFR/EGR3/TMEM17/EPHA3/EPHB4/RASA3/FOXO1/SPG20/FLNB/LARP1/MTOR/GABBR1/TENM4/RGS22/GJA3/GJB2/SDCBP2/DKK3/GLS2/GPR162/GNAS/GPER1/GSTP1/NME7/ANXA2/KCNIP2/NRG1/ANXA6/HLA-B/HLA-E/HLA-F/HPCA/APBA2/HOXD3/HSP90AA1/HTR5A/CYR61/IL6/ISL1/LCK/LLGL1/LTB/LTBP1/SMAD3/MAP3K1/NEDD9/NMBR/NOV/NTF3/PALM/ARHGEF3/LEF1/DDX47/ANGPT4/PDE4C/PDE7A/PGAM2/PIK3CG/IL20RB/CYTL1/POMC/ZDHHC13/PPP1CC/ARHGEF10L/SMPD3/SYBU/LIMS2/VAC14/PRKD1/PSMB4/LPAR5/CCAR2/PXN/RASGRF2/S100A4/S100A6/CCL17/SFRP2/CXCR5/SGK1/MICAL1/SOX9/STK10/VAMP2/TLE3/TLR5/TNXB/TRAF1/TWIST1/YWHAG/CACNB2/PAX8/CXCR4/FZD5/TMEM204/NLRX1/RAB11FIP1/UNC93B1/CAPS/COLQ/HIST1H3A/SLA2/IL1F10/FADD/LIMD1/ARHGAP29/LY86/RAB3D/SMAD5-AS1/ARHGEF10/ULK2/TELO2	129
GO:0050777	negative regulation of immune response	8/311	111/17046	0.00097	0.02857	0.02278	NLRP6/HLA-B/HLA-E/HLX/IL20RB/MASP1/PSMB4/NLRX1	8
GO:0072522	purine-containing compound biosynthetic process	13/311	259/17046	0.00098	0.02857	0.02278	TCIRG1/NPFFR2/ADCY3/ADRB3/ADAL/DRD4/GABBR1/AMPD2/GNAS/GPER1/NME7/HPCA/PALM	13
GO:0001756	somitogenesis	6/311	63/17046	0.001	0.02857	0.02278	DMRT2/SMAD3/MEOX1/MEOX2/LEF1/SFRP2	6
GO:0048584	positive regulation of response to stimulus	52/311	1848/17046	0.001	0.02857	0.02278	CDH3/TSPAN5/CDH13/HCST/CTGF/ADRB3/RNF168/COCH/NLRP6/DRD4/EGFR/RASA3/LARP1/MTOR/GNAS/GPER1/NRG1/HLA-B/HLA-E/HLX/HMGA1/HSP90AA1/CYR61/IL6/ISL1/LCK/SMAD3/MAP3K1/NOV/NTF3/PIK3CG/ZDHHC13/LIMS2/PRKD1/MASP1/PSMB4/CCAR2/PXN/RASGRF2/S100A4/CCL17/SFRP2/SOX9/TLR5/YWHAG/CXCR4/FZD5/NLRX1/UNC93B1/SLA2/FADD/LY86	52
GO:0023052	signaling	129/311	5629/17046	0.001	0.02857	0.02278	CDH3/TSPAN5/CDH13/KCNMB2/TCIRG1/HCST/NPFFR2/ADCY3/PTH2/CHRNA5/CIDEA/APCDD1/CTGF/ADRB3/NLRP6/DRD4/ECE1/EGFR/EGR3/TMEM17/EPHA3/EPHB4/RASA3/FOXO1/SPG20/FLNB/LARP1/MTOR/GABBR1/TENM4/RGS22/GJA3/GJB2/SDCBP2/DKK3/GLS2/GPR162/GNAS/GPER1/GSTP1/NME7/ANXA2/KCNIP2/NRG1/ANXA6/HLA-B/HLA-E/HLA-F/HPCA/APBA2/HOXD3/HSP90AA1/HTR5A/CYR61/IL6/ISL1/LCK/LLGL1/LTB/LTBP1/SMAD3/MAP3K1/NEDD9/NMBR/NOV/NTF3/PALM/ARHGEF3/LEF1/DDX47/ANGPT4/PDE4C/PDE7A/PGAM2/PIK3CG/IL20RB/CYTL1/POMC/ZDHHC13/PPP1CC/ARHGEF10L/SMPD3/SYBU/LIMS2/VAC14/PRKD1/PSMB4/LPAR5/CCAR2/PXN/RASGRF2/S100A4/S100A6/CCL17/SFRP2/CXCR5/SGK1/MICAL1/SOX9/STK10/VAMP2/TLE3/TLR5/TNXB/TRAF1/TWIST1/YWHAG/CACNB2/PAX8/CXCR4/FZD5/TMEM204/NLRX1/RAB11FIP1/UNC93B1/CAPS/COLQ/HIST1H3A/SLA2/IL1F10/FADD/LIMD1/ARHGAP29/LY86/RAB3D/SMAD5-AS1/ARHGEF10/ULK2/TELO2	129
GO:0007015	actin filament organization	14/311	293/17046	0.00102	0.02892	0.02306	CTGF/PPM1E/MTOR/FMN1/SMAD3/MAP3K1/NEDD9/BIN3/ACTR3B/MICAL1/ACTC1/SH3BGRL3/GAS7/ARHGEF10	14
GO:0098609	cell-cell adhesion	28/311	816/17046	0.00103	0.02892	0.02306	CDH3/CDH13/DSG3/EGFR/EGR3/MTOR/GNAS/HLA-E/HLX/CYR61/IL6/CDHR4/LCK/SMAD3/NOV/LEF1/PIK3CG/IL20RB/LIMS2/PCDHGC4/PCDHGB7/PCDHGA11/SOX9/STK10/TNXB/FZD5/SLA2/FADD	28

GO:0010033	response to organic substance	70/311	2687/17046	0.00103	0.02892	0.02306	CDH13/TCIRG1/NPFFR2/ADCY3/CHRNA5/CIDEA/CTGF/CITED4/DNMT3A/DRD4/EGFR/EGR3/EPHA3/RASA3/FOXO1/SPG20/FLNB/MTOR/FBXO2/GJB2/GNAS/GPER1/GSTP1/NRG1/HLA-B/HLA-E/HLA-F/HPCA/HSD17B2/HSP90AA1/HTR5A/CYR61/IL6/ISL1/ACAT1/LCK/LOX/LTB/LTBP1/SMAD3/ME1/MAP3K1/ARHGEF3/LEF1/PIK3CG/IL20RB/PON1/PPP1CC/SYBU/PRKD1/PSMB4/PXN/RASGRF2/CCL17/SFRP2/CXCR5/SOX9/VAMP2/ACTC1/TIMP3/TLR5/TWIST1/PAX8/CXCR4/FZD5/TMEM204/IL1F10/MGARP/FADD/LY86	70
GO:0030817	regulation of cAMP biosynthetic process	8/311	113/17046	0.00109	0.03021	0.02409	NPFFR2/ADRB3/DRD4/GABBR1/GNAS/GPER1/HPCA/PALM	8
GO:0048736	appendage development	10/311	169/17046	0.0011	0.03021	0.02409	ECE1/SP8/GNAS/FLVCR1/FMN1/MEOX2/LEF1/SFRP2/SOX9/TWIST1	10
GO:0060173	limb development	10/311	169/17046	0.0011	0.03021	0.02409	ECE1/SP8/GNAS/FLVCR1/FMN1/MEOX2/LEF1/SFRP2/SOX9/TWIST1	10
GO:0051345	positive regulation of hydrolase activity	27/311	781/17046	0.00114	0.03103	0.02475	ADCY3/CTGF/EGFR/RASA3/TBC1D9B/RGS22/GNAS/GPER1/HPCA/CYR61/LCK/LLGL1/SMAD3/NTF3/ARHGEF3/ARHGEF10L/PRKD1/RASGRF2/CCL17/SFRP2/TCEA1/SH3BGRL3/FADD/ARHGAP29/ARHGEF10/USP6NL/RABGAP1L	27
GO:0009893	positive regulation of metabolic process	82/311	3277/17046	0.00117	0.03131	0.02496	CDH3/CDH13/DMRT2/TBR1/ADCY3/PSIP1/CTGF/ADRB3/CITED4/RNF168/DRD4/ECE1/EGFR/RASA3/TBC1D9B/FOXO1/LARP1/MTOR/RGS22/GAPDHS/GNAS/GPER1/DOK7/GSTP1/BRF1/ANXA2/NRG1/HMGA1/HPCA/HOXD3/HSP90AA1/BARHL2/CYR61/IL6/ISL1/LCK/LLGL1/LTB/SMAD3/MEF2D/MAP3K1/MEOX1/MEOX2/MFI2/NFYB/NTF3/ARHGEF3/LEF1/ANGPT4/PIK3CG/RIPK4/CYTL1/POMC/PIWIL2/ARHGEF10L/PRKD1/PSMB4/PXN/RASGRF2/CCL17/SFRP2/TRA2B/SOX9/STK10/TBP/TCEA1/ACTC1/TLR5/TRAF1/TWIST1/PAX8/CXCR4/FZD5/SH3BGRL3/FADD/ZFAND2A/LDB2/ARHGAP29/H2AFY/ARHGEF10/USP6NL/RABGAP1L	82
GO:0043534	blood vessel endothelial cell migration	6/311	65/17046	0.00118	0.03131	0.02496	EGR3/EPHB4/VASH1/ANGPT4/PRKD1/SH3BGRL3	6
GO:0030036	actin cytoskeleton organization	20/311	511/17046	0.00118	0.03131	0.02496	CTGF/EPHA3/PPM1E/LIMCH1/FLNB/MTOR/FMN1/LLGL1/SMAD3/MAP3K1/NEDD9/NTF3/BIN3/ACTR3B/MICAL1/ACTC1/TNXB/SH3BGRL3/GAS7/ARHGEF10	20
GO:0007166	cell surface receptor signaling pathway	66/311	2511/17046	0.00118	0.03131	0.02496	CDH3/TSPAN5/CDH13/TCIRG1/ADCY3/CIDEA/APCDD1/CTGF/EGFR/TMEM17/EPHA3/EPHB4/RASA3/FOXO1/SPG20/FLNB/MTOR/DKK3/GPER1/GSTP1/NME7/NRG1/HLA-B/HLA-E/HLA-F/HOXD3/HSP90AA1/CYR61/IL6/ISL1/LCK/LLGL1/LTB/LTBP1/SMAD3/MAP3K1/NEDD9/NOV/NTF3/ARHGEF3/LEF1/DDX47/ANGPT4/PGAM2/IL20RB/PPP1CC/LIMS2/PRKD1/PSMB4/CCAR2/PXN/RASGRF2/CCL17/SFRP2/CXCR5/SOX9/TLE3/TRAF1/CXCR4/FZD5/TMEM204/SLA2/IL1F10/FADD/LIMD1/LY86	66
GO:0060561	apoptotic process involved in morphogenesis	4/311	26/17046	0.00118	0.03131	0.02496	CYR61/LEF1/PAX8/FZD5	4
GO:0098602	single organism cell adhesion	25/311	704/17046	0.00119	0.03141	0.02505	CDH3/EGFR/EGR3/UNC13D/MTOR/GNAS/HLA-E/HLX/CYR61/IL6/LCK/SMAD3/MFI2/NOV/LEF1/PIK3CG/IL20RB/LIMS2/PXN/SOX9/STK10/TNXB/FZD5/SLA2/FADD	25
GO:0050678	regulation of epithelial cell proliferation	13/311	265/17046	0.00121	0.03162	0.02521	CDH3/CDH13/EGFR/EGR3/VASH1/MTOR/IL6/SMAD3/LIMS2/PRKD1/SFRP2/SOX9/TWIST1	13
GO:2000026	regulation of multicellular organismal development	43/311	1465/17046	0.00127	0.03297	0.02629	CDH3/DMRT2/TBR1/CTGF/EGR3/EPHA3/VASH1/ACIN1/SPG20/MTOR/TENM4/GNAS/GPER1/NRG1/HLA-B/HLX/HOXB3/HOXD3/BARHL2/CYR61/IL6/ISL1/LCK/SMAD3/NTF3/PALM/LEF1/CEND1/ANGPT4/LIMS2/PRKD1/SFRP2/SOX9/PHLDA2/TWIST1/YWHAG/PAX8/CXCR4/COLQ/SCRT1/FADD/H2AFY/ULK2	43
GO:0009967	positive regulation of signal transduction	40/311	1334/17046	0.00128	0.03297	0.02629	CDH3/TSPAN5/CDH13/HCST/CTGF/ADRB3/DRD4/EGFR/RASA3/MTOR/GNAS/GPER1/NRG1/CYR61/IL6/ISL1/LCK/SMAD3/MAP3K1/NOV/NTF3/PIK3CG/ZDHHC13/LIMS2/PRKD1/PSMB4/CCAR2/PXN/RASGRF2/S100A4/CCL17/SFRP2/SOX9/TLR5/YWHAG/CXCR4/FZD5/SLA2/FADD/LY86	40

GO:0006140	regulation of nucleotide metabolic process	11/311	203/17046	0.00129	0.03297	0.02629	NPFFR2/ADRB3/DRD4/GABBR1/GAPDHS/GNAS/GPER1/HPCA/ME1/PALM/PGAM2	11
GO:0051252	regulation of RNA metabolic process	83/311	3337/17046	0.00129	0.03297	0.02629	CDH13/MBNL2/C1D/ZBTB18/DMRT2/TBR1/PSIP1/ZBED9/CIDEA/ZFP42/ZNF709/ZNF781/CITED4/RNF168/DNMT3A/EGFR/EGR3/SP8/XRN2/ACIN1/FOXO1/SPG20/MTOR/DKK3/GPER1/ZBTB44/GTF2B/BRF1/NRG1/HLX/HMGA1/HOXB3/HOXC5/HOXC6/HOXD3/BARHL2/CYR61/IL6/ISL1/HILS1/SMAD3/MEF2D/MEOX1/MEOX2/NFYB/NTF3/LEF1/RIPK4/CYTL1/POMC/BNC2/PRMT6/ZNF532/CNOT11/PRKD1/CCAR2/SFRP2/TRA2B/SGK1/SOX9/TAF4B/TBP/TCEA1/TLE3/TRAF1/TWIST1/ZNF124/ZNF177/PAX8/FZD5/ZC3H14/ZNF606/SCRT1/HIST1H3A/SLA2/KDM2B/LOXL3/CBX2/GAS7/FADD/LIMD1/LDB2/H2AFY	83
GO:0048523	negative regulation of cellular process	93/311	3831/17046	0.00131	0.03297	0.02629	CDH13/C1D/ZBTB18/B4GALT7/CIDEA/ANKRD9/APCDD1/CTGF/RNF168/NLRP6/DNMT3A/DRD4/EGFR/EGR3/PATL2/RASA3/PPM1E/VASH1/ACIN1/FOXO1/SPG20/MTOR/GABBR1/RGS22/FBXO2/DKK3/VPS4A/GPER1/GSTP1/GZMA/ANXA2/SERPIND1/NRG1/HLX/HMGA1/HPCA/HOXB3/HOXC6/ACADL/COL28A1/CYR61/IL6/ISL1/ITIH3/HILS1/LCK/LTBP1/SMAD3/MFI2/NOV/NTF3/PALM/LEF1/CEND1/ANGPT4/PDE4C/PIK3CG/IL20RB/PPP1CC/PRMT6/LIMS2/PRKD1/MASP1/PSMB4/METTL14/CCAR2/CCL17/SFRP2/SOX9/TBP/ACTC1/TIMP3/TWIST1/YWHAG/ZNF177/PAX8/FZD5/NLRX1/ZC3H14/RAB11FIP1/COL18A1/SCRT1/HIST1H3A/SLA2/SPINK7/KDM2B/LOXL3/CBX2/GAS7/FADD/LIMD1/H2AFY/ULK2	93
GO:0002709	regulation of T cell mediated immunity	5/311	45/17046	0.00132	0.03297	0.02629	HLA-B/HLA-E/IL20RB/FZD5/FADD	5
GO:0032233	positive regulation of actin filament bundle assembly	5/311	45/17046	0.00132	0.03297	0.02629	CTGF/PPM1E/MTOR/SMAD3/ARHGEF10	5
GO:0061061	muscle structure development	21/311	554/17046	0.00133	0.03311	0.02641	ZBTB18/COL11A1/EGR3/FLNB/MTOR/NRG1/HLX/IL6/ISL1/SMAD3/MEF2D/MEOX2/NOV/NTF3/LEF1/BIN3/CCL17/SOX9/ACTC1/TWIST1/TMEM204	21
GO:0032680	regulation of tumor necrosis factor production	7/311	91/17046	0.00138	0.03388	0.02702	CIDEA/GSTP1/HLA-E/ISL1/POMC/TWIST1/FADD	7
GO:0035282	segmentation	7/311	91/17046	0.00138	0.03388	0.02702	DMRT2/SMAD3/MEOX1/MEOX2/LEF1/SFRP2/FZD5	7
GO:0051046	regulation of secretion	23/311	633/17046	0.00139	0.03392	0.02705	CIDEA/NLRP6/DRD4/UNC13D/VPS4A/GNAS/GPER1/NRG1/HLA-E/IL6/ISL1/LLGL1/NOV/PDE4C/POMC/SMPD3/SYBU/SGK1/VAMP2/TWIST1/PAX8/RAB11FIP1/RAB3D	23
GO:0007517	muscle organ development	15/311	337/17046	0.00139	0.03392	0.02705	ZBTB18/COL11A1/EGR3/FLNB/NRG1/HLX/IL6/ISL1/SMAD3/MEF2D/MEOX2/LEF1/BIN3/ACTC1/TWIST1	15
GO:0007154	cell communication	132/311	5832/17046	0.00142	0.03427	0.02733	CDH3/TSPAN5/CDH13/KCNMB2/TCIRG1/HCST/NPFFR2/ADCY3/PTH2/CHRNA5/CIDEA/APCDD1/CTGF/ADRB3/NLRP6/DRD4/EGFR/EGR3/TMEM17/EPHA3/EPHB4/RASA3/ACIN1/FOXO1/SPG20/FLNB/LARP1/MTOR/GABBR1/TENM4/RGS22/GJA3/GJB2/SDCBP2/DKK3/GLS2/GPR162/GNAS/GPER1/GSTP1/NME7/ANXA2/KCNIP2/NRG1/ANXA6/HLA-B/HLA-E/HLA-F/HPCA/APBA2/HOXD3/HSP90AA1/HTR5A/CYR61/IL6/ISL1/KRT15/LCK/LLGL1/LTB/LTBP1/SMAD3/MAP3K1/NUDT1/NEDD9/NMBR/NOV/NTF3/PALM/ARHGEF3/LEF1/DDX47/ANGPT4/PDE4C/PDE7A/PGAM2/PIK3CG/IL20RB/CYTL1/POMC/ZDHHC13/PPP1CC/ARHGEF10L/SMPD3/SYBU/LIMS2/VAC14/PRKD1/PSMB4/LPAR5/CCAR2/PXN/RASGRF2/S100A4/S100A6/CCL17/SFRP2/CXCR5/SGK1/MICAL1/SOX9/STK10/VAMP2/TLE3/TLR5/TNXB/TRAF1/TWIST1/UPP1/YWHAG/CACNB2/PAX8/CXCR4/FZD5/TMEM204/NLRX1/RAB11FIP1/UNC93B1/CAPS/COLQ/HIST1H3A/SLA2/IL1F10/FADD/LIMD1/ARHGAP29/LY86/RAB3D/SMAD5-AS1/ARHGEF10/ULK2/TELO2	132
GO:0060537	muscle tissue development	15/311	338/17046	0.00143	0.03453	0.02754	ZBTB18/COL11A1/FLNB/TENM4/NRG1/HLX/ISL1/SMAD3/MEF2D/MEOX2/LEF1/BIN3/SOX9/ACTC1/TWIST1	15
GO:0010628	positive regulation of gene expression	46/311	1608/17046	0.00144	0.03459	0.02759	CDH3/CDH13/DMRT2/TBR1/PSIP1/CTGF/CITED4/EGFR/FOXO1/LARP1/MTOR/GPER1/BRF1/HMGA1/HOXD3/BARHL2/CYR61/IL6/ISL1/LCK/SMAD3/MEF2D/MEOX1/MEOX2/MFI2/NFYB/NTF3/LEF1/RIPK4/CYTL1/POMC/PIWIL2/PRKD1/SFRP2/TRA2B/SOX9/TBP/TCEA1/ACTC1/TRAF1/TWIST1/PAX8/FZD5/FADD/LDB2/H2AFY	46
GO:0060548	negative regulation of cell death	28/311	835/17046	0.00145	0.03461	0.0276	CIDEA/ANKRD9/CTGF/EGFR/EGR3/FOXO1/GSTP1/NRG1/CYR61/IL6/ISL1/SMAD3/NOV/NTF3/LEF1/ANGPT4/PIK3CG/LIMS2/PRKD1/PSMB4/CCAR2/SFRP2/SOX9/ACTC1/TWIST1/PAX8/KDM2B/FADD	28
GO:0048863	stem cell differentiation	12/311	238/17046	0.00147	0.03473	0.02769	EPHA3/NRG1/ISL1/SMAD3/MEOX1/LEF1/S100A4/SFRP2/SOX9/TWIST1/PAX8/LOXL3	12
GO:0006935	chemotaxis	27/311	796/17046	0.0015	0.03506	0.02796	CDH13/TBR1/EGFR/EGR3/EPHA3/EPHB4/RASA3/SERPIND1/NRG1/HSP90AA1/CYR61/IL6/ISL1/SMAD3/NOV/NTF3/LEF1/PIK3CG/PRKD1/PSMB4/TRPC7/RASGRF2/CCL17/CXCR5/TRPC4/CACNB2/CXCR4	27
GO:0042330	taxis	27/311	796/17046	0.0015	0.03506	0.02796	CDH13/TBR1/EGFR/EGR3/EPHA3/EPHB4/RASA3/SERPIND1/NRG1/HSP90AA1/CYR61/IL6/ISL1/SMAD3/NOV/NTF3/LEF1/PIK3CG/PRKD1/PSMB4/TRPC7/RASGRF2/CCL17/CXCR5/TRPC4/CACNB2/CXCR4	27

GO:0030029	actin filament-based process	21/311	561/17046	0.00155	0.03588	0.02862	FRMD6/CTGF/EPHA3/PPM1E/LIMCH1/FLNB/MTOR/FMN1/LLGL1/SMAD3/MAP3K1/NEDD9/NTF3/BIN3/ACTR3B/MICAL1/ACTC1/TNXB/SH3BGRL3/GAS7/ARHGEF10	21
GO:0032640	tumor necrosis factor production	7/311	93/17046	0.00156	0.03588	0.02862	CIDEA/GSTP1/HLA-E/ISL1/POMC/TWIST1/FADD	7
GO:2000147	positive regulation of cell motility	15/311	341/17046	0.00156	0.03588	0.02862	CDH13/EGFR/GPER1/CYR61/IL6/SMAD3/NTF3/LEF1/ANGPT4/PRKD1/SOX9/TWIST1/PTP4A1/COL18A1/FADD	15
GO:0042981	regulation of apoptotic process	39/311	1305/17046	0.00157	0.03588	0.02862	CIDEA/ANKRD9/CTGF/EGFR/EGR3/ACIN1/FOXO1/GLS2/GPER1/GSTP1/GZMA/NRG1/CYR61/IL6/ISL1/LCK/SMAD3/MAP3K1/NTF3/ARHGEF3/LEF1/ANGPT4/PIK3CG/LIMS2/PSMB4/CCAR2/RASGRF2/SFRP2/SGK1/SOX9/STK10/ACTC1/TRAF1/TWIST1/YWHAG/PAX8/COL18A1/KDM2B/FADD	39
GO:0009152	purine ribonucleotide biosynthetic process	12/311	240/17046	0.00157	0.03588	0.02862	TCIRG1/NPFFR2/ADCY3/ADRB3/DRD4/GABBR1/AMPD2/GNAS/GPER1/NME7/HPCA/PALM	12
GO:0035107	appendage morphogenesis	9/311	148/17046	0.0016	0.03603	0.02874	ECE1/SP8/GNAS/FLVCR1/FMN1/LEF1/SFRP2/SOX9/TWIST1	9
GO:0035108	limb morphogenesis	9/311	148/17046	0.0016	0.03603	0.02874	ECE1/SP8/GNAS/FLVCR1/FMN1/LEF1/SFRP2/SOX9/TWIST1	9
GO:0045785	positive regulation of cell adhesion	15/311	343/17046	0.00166	0.03707	0.02956	CDH13/EGR3/UNC13D/MTOR/NRG1/HLA-E/HLX/FMN1/CYR61/IL6/LCK/SMAD3/LEF1/SFRP2/FADD	15
GO:1903555	regulation of tumor necrosis factor superfamily cytokine production	7/311	94/17046	0.00166	0.03707	0.02956	CIDEA/GSTP1/HLA-E/ISL1/POMC/TWIST1/FADD	7
GO:0031325	positive regulation of cellular metabolic process	69/311	2688/17046	0.00167	0.03714	0.02962	CDH3/CDH13/DMRT2/TBR1/ADCY3/PSIP1/CTGF/ADRB3/CITED4/RNF168/DRD4/ECE1/EGFR/RASA3/FOXO1/LARP1/MTOR/GAPDHS/GNAS/GPER1/DOK7/GSTP1/BRF1/ANXA2/NRG1/HMGA1/HPCA/HSP90AA1/BARHL2/CYR61/IL6/ISL1/LCK/LTB/SMAD3/MEF2D/MAP3K1/MEOX1/MEOX2/MFI2/NFYB/NTF3/LEF1/ANGPT4/PIK3CG/RIPK4/CYTL1/POMC/PIWIL2/PRKD1/PSMB4/PXN/RASGRF2/CCL17/SFRP2/TRA2B/SOX9/STK10/TBP/TCEA1/TLR5/TRAF1/TWIST1/PAX8/CXCR4/FZD5/FADD/ZFAND2A/LDB2	69
GO:0006955	immune response	43/311	1487/17046	0.0017	0.03752	0.02992	HCST/ADCY3/RNF168/COCH/NLRP6/EGFR/UNC13D/RASA3/FOXO1/MTOR/GPER1/GZMA/NRG1/HLA-B/HLA-E/HLA-F/HLX/HSP90AA1/IL6/LCK/LTB/SMAD3/MAP3K1/LEF1/PIK3CG/IL20RB/PRKD1/MASP1/PSMB4/RASGRF2/DEFB134/CCL17/CXCR5/VAMP2/TLR5/FZD5/NLRX1/UNC93B1/SLA2/IL1F10/FADD/IL32/LY86	43
GO:0050679	positive regulation of epithelial cell proliferation	9/311	150/17046	0.00175	0.03852	0.03072	CDH3/CDH13/EGFR/EGR3/MTOR/IL6/PRKD1/SOX9/TWIST1	9
GO:0043067	regulation of programmed cell death	39/311	1314/17046	0.00177	0.03879	0.03093	CIDEA/ANKRD9/CTGF/EGFR/EGR3/ACIN1/FOXO1/GLS2/GPER1/GSTP1/GZMA/NRG1/CYR61/IL6/ISL1/LCK/SMAD3/MAP3K1/NTF3/ARHGEF3/LEF1/ANGPT4/PIK3CG/LIMS2/PSMB4/CCAR2/RASGRF2/SFRP2/SGK1/SOX9/STK10/ACTC1/TRAF1/TWIST1/YWHAG/PAX8/COL18A1/KDM2B/FADD	39
GO:1902107	positive regulation of leukocyte differentiation	8/311	122/17046	0.00179	0.03879	0.03093	EGR3/ACIN1/GNAS/HLX/IL6/LCK/LEF1/FADD	8

GO:0002711	positive regulation of T cell mediated immunity	4/311	29/17046	0.0018	0.03879	0.03093	HLA-B/HLA-E/FZD5/FADD	4
GO:0031280	negative regulation of cyclase activity	4/311	29/17046	0.0018	0.03879	0.03093	DRD4/GABBR1/HPCA/PALM	4
GO:0071822	protein complex subunit organization	46/311	1629/17046	0.00187	0.03948	0.03149	TCIRG1/SEPT9/APOA1BP/COL11A1/CTGF/EML1/PPM1E/MTOR/VPS4A/ANXA2/NRG1/ANXA6/HMGA1/ACADL/HSP90AA1/FMN1/HILS1/ACAT1/LLGL1/LOX/SMAD3/ME1/MAP3K1/NEDD9/ANGPT4/BIN3/ACTR3B/PXN/RPL8/RPL29/SFRP2/MICAL1/SOX9/VAMP2/ACTC1/TNXB/TRAF1/TWIST1/HIST1H2BM/SH3BGRL3/HIST1H3A/PARD6B/GAS7/FADD/H2AFY/ARHGEF10	46
GO:0045597	positive regulation of cell differentiation	26/311	768/17046	0.00187	0.03948	0.03149	CTGF/EGR3/UNC13D/EPHA3/ACIN1/MTOR/TENM4/GNAS/GPER1/NRG1/HLX/HOXD3/CYR61/IL6/LCK/SMAD3/PALM/LEF1/PRKD1/SFRP2/SOX9/TWIST1/PAX8/CXCR4/FADD/H2AFY	26
GO:0048704	embryonic skeletal system morphogenesis	7/311	96/17046	0.00188	0.03948	0.03149	COL11A1/GNAS/FLVCR1/HOXB3/HOXD3/SMAD3/TWIST1	7
GO:0021781	glial cell fate commitment	3/311	14/17046	0.00189	0.03948	0.03149	NRG1/NTF3/SOX9	3
GO:0048871	multicellular organismal homeostasis	14/311	313/17046	0.0019	0.03948	0.03149	CDH3/ADCY3/CIDEA/ZG16B/CTGF/ADRB3/FOXO1/AMPD2/GNAS/ACADL/IL20RB/CYTL1/SOX9/LDB2	14
GO:0030001	metal ion transport	26/311	769/17046	0.00191	0.03948	0.03149	KCNMB2/TCIRG1/CLCA1/C15orf27/SLC38A10/TRPM6/CTGF/TRPV3/DRD4/RASA3/CRACR2B/GPER1/KCNIP2/ANXA6/LCK/MFI2/PIK3CG/ZDHHC13/PRKD1/TRPC7/SGK1/VAMP2/TRAPPC10/TRPC4/CACNB2/SMDT1	26
GO:0022604	regulation of cell morphogenesis	18/311	457/17046	0.00191	0.03948	0.03149	TBR1/COCH/UNC13D/EPHA3/SPG20/BARHL2/IL6/SMAD3/MFI2/PALM/LEF1/PXN/SFRP2/TWIST1/PAX8/GAS7/LIMD1/ULK2	18
GO:0070588	calcium ion transmembrane transport	10/311	182/17046	0.00191	0.03948	0.03149	C15orf27/TRPM6/TRPV3/DRD4/RASA3/PIK3CG/TRPC7/TRPC4/CACNB2/SMDT1	10
GO:1901564	organonitrogen compound metabolic process	54/311	1996/17046	0.00192	0.03948	0.03149	BCKDK/TCIRG1/NPFFR2/ADCY3/HIBADH/B4GALT7/APOA1BP/ADRB3/ADAL/DNMT3A/DRD4/ECE1/PATL2/ALAS1/LARP1/MTOR/FUCA1/GABBR1/GAPDHS/AMPD2/GLS2/GNAS/GPER1/GSTP1/NME7/HPCA/ACADL/BARHL2/CYR61/IL6/ITIH3/ACAT1/ME1/NUDT1/PALM/PDE4C/PDE7A/PGAM2/CYTL1/POMC/PON1/LPCAT2/PIWIL2/SMPD3/CNOT11/PRKD1/PSMB4/METTL14/RPL8/RPL29/CERK/UPP1/VARS/PAX8	54
GO:0014032	neural crest cell development	5/311	49/17046	0.00193	0.03948	0.03149	NRG1/ISL1/LEF1/SOX9/TWIST1	5
GO:0006164	purine nucleotide biosynthetic process	12/311	246/17046	0.00194	0.03948	0.03149	TCIRG1/NPFFR2/ADCY3/ADRB3/DRD4/GABBR1/AMPD2/GNAS/GPER1/NME7/HPCA/PALM	12
GO:0051272	positive regulation of cellular component movement	15/311	349/17046	0.00196	0.03948	0.03149	CDH13/EGFR/GPER1/CYR61/IL6/SMAD3/NTF3/LEF1/ANGPT4/PRKD1/SOX9/TWIST1/PTP4A1/COL18A1/FADD	15
GO:0010562	positive regulation of phosphorus metabolic process	33/311	1062/17046	0.00196	0.03948	0.03149	ADCY3/CTGF/ADRB3/DRD4/EGFR/RASA3/MTOR/GAPDHS/GNAS/GPER1/DOK7/ANXA2/NRG1/HPCA/CYR61/IL6/ISL1/LCK/SMAD3/MAP3K1/NTF3/ANGPT4/PIK3CG/PRKD1/PSMB4/PXN/RASGRF2/CCL17/SFRP2/SOX9/STK10/CXCR4/FZD5	33

GO:0045937	positive regulation of phosphate metabolic process	33/311	1062/17046	0.00196	0.03948	0.03149	ADCY3/CTGF/ADRB3/DRD4/EGFR/RASA3/MTOR/GAPDHS/GNAS/GPER1/DOK7/ANXA2/NRG1/HPCA/CYR61/IL6/ISL1/LCK/SMAD3/MAP3K1/NTF3/ANGPT4/PIK3CG/PRKD1/PSMB4/PXN/RASGRF2/CCL17/SFRP2/SOX9/STK10/CXCR4/FZD5	33
GO:0009165	nucleotide biosynthetic process	14/311	315/17046	0.00202	0.04043	0.03224	TCIRG1/NPFFR2/ADCY3/ADRB3/DRD4/GABBR1/AMPD2/GNAS/GPER1/NME7/HPCA/ME1/PALM/UPP1	14
GO:0044085	cellular component biogenesis	61/311	2329/17046	0.00205	0.04087	0.03259	CDH3/CDH13/TCIRG1/C1D/SEPT9/PSIP1/APOA1BP/SCLT1/CTGF/WBP2NL/PATL2/TMEM17/EPHA3/PPM1E/MTOR/TENM4/GJB2/VPS4A/ANXA2/NRG1/ANXA6/HMGA1/ACADL/HSP90AA1/FMN1/CYR61/HILS1/ACAT1/LLGL1/LOX/SMAD3/ME1/MAP3K1/NEDD9/PALM/DDX47/ANGPT4/PLEC/LIMS2/BIN3/ACTR3B/PXN/SOX9/VAMP2/ACTC1/TNXB/TRAF1/TWIST1/FZD5/COLQ/HIST1H2BM/HIST1H3A/PARD6B/GAS7/FADD/LIMD1/LDB2/H2AFY/ARHGEF10/ULK2/USP6NL	61
GO:0071706	tumor necrosis factor superfamily cytokine production	7/311	98/17046	0.00211	0.04188	0.0334	CIDEA/GSTP1/HLA-E/ISL1/POMC/TWIST1/FADD	7
GO:0034329	cell junction assembly	11/311	217/17046	0.00219	0.04315	0.03441	CDH3/CDH13/EPHA3/GJB2/FMN1/SMAD3/PLEC/LIMS2/PXN/FZD5/PARD6B	11
GO:0010467	gene expression	111/311	4805/17046	0.0022	0.04315	0.03441	CDH3/TSPAN5/CDH13/MBNL2/C1D/ZBTB18/DMRT2/CELF2/TBR1/PSIP1/ZBED9/CIDEA/ZFP42/CTGF/ZNF709/ZNF781/CITED4/RNF168/DNMT3A/ECE1/EGFR/EGR3/PATL2/SP8/XRN2/ACIN1/FOXO1/LARP1/MTOR/DKK3/GLS2/GNAS/GPER1/ZBTB44/GTF2B/BRF1/NRG1/HLX/HMGA1/APBA2/HOXB3/HOXC5/HOXC6/HOXD3/FMN1/BARHL2/CYR61/IL6/ISL1/HILS1/LCK/LTB/SMAD3/MEF2D/MEOX1/MEOX2/MFI2/NFYB/NOV/NTF3/LEF1/DDX47/RIPK4/CYTL1/POMC/MOV10L1/BNC2/PPP1CC/PIWIL2/PRMT6/ZNF532/WDR33/CNOT11/PRKD1/MASP1/PSMB4/METTL14/CCAR2/RPL8/RPL29/SFRP2/TRA2B/SGK1/SOX9/TAF4B/TBP/TCEA1/ACTC1/TLE3/TRAF1/PHLDA2/TWIST1/VARS/YWHAG/ZNF124/ZNF177/PAX8/FZD5/ZC3H14/ZNF606/SCRT1/HIST1H3A/SLA2/KDM2B/LOXL3/CBX2/GAS7/FADD/LIMD1/LDB2/H2AFY	111
GO:0009725	response to hormone	31/311	985/17046	0.00221	0.04315	0.03441	TCIRG1/NPFFR2/ADCY3/CTGF/CITED4/DNMT3A/DRD4/EGFR/EGR3/RASA3/FOXO1/MTOR/GJB2/GNAS/GPER1/NRG1/HTR5A/IL6/ISL1/ACAT1/LCK/LOX/ME1/LEF1/PSMB4/PXN/RASGRF2/VAMP2/TIMP3/PAX8/MGARP	31
GO:0001101	response to acid chemical	13/311	284/17046	0.00224	0.04315	0.03441	CTGF/DNMT3A/EGFR/EPHA3/MTOR/GNAS/HPCA/HSD17B2/IL6/PON1/SOX9/TIMP3/COL18A1	13
GO:1901293	nucleoside phosphate biosynthetic process	14/311	319/17046	0.00227	0.04315	0.03441	TCIRG1/NPFFR2/ADCY3/ADRB3/DRD4/GABBR1/AMPD2/GNAS/GPER1/NME7/HPCA/ME1/PALM/UPP1	14
GO:0050727	regulation of inflammatory response	12/311	251/17046	0.00229	0.04315	0.03441	NLRP6/GPER1/GSTP1/IL6/ISL1/SMAD3/NOV/PIK3CG/IL20RB/MASP1/PSMB4/NLRX1	12
GO:0022607	cellular component assembly	57/311	2152/17046	0.00229	0.04315	0.03441	CDH3/CDH13/TCIRG1/SEPT9/PSIP1/APOA1BP/SCLT1/CTGF/WBP2NL/PATL2/TMEM17/EPHA3/PPM1E/MTOR/TENM4/GJB2/VPS4A/ANXA2/NRG1/ANXA6/HMGA1/ACADL/HSP90AA1/FMN1/HILS1/ACAT1/LLGL1/LOX/SMAD3/ME1/MAP3K1/NEDD9/PALM/ANGPT4/PLEC/LIMS2/BIN3/ACTR3B/PXN/SOX9/VAMP2/ACTC1/TNXB/TRAF1/TWIST1/FZD5/COLQ/HIST1H2BM/HIST1H3A/PARD6B/GAS7/FADD/LIMD1/H2AFY/ARHGEF10/ULK2/USP6NL	57
GO:0030814	regulation of cAMP metabolic process	8/311	127/17046	0.0023	0.04315	0.03441	NPFFR2/ADRB3/DRD4/GABBR1/GNAS/GPER1/HPCA/PALM	8
GO:0060284	regulation of cell development	25/311	739/17046	0.00231	0.04315	0.03441	TBR1/UNC13D/EPHA3/SPG20/TENM4/GPER1/HOXB3/HOXD3/BARHL2/IL6/ISL1/SMAD3/MFI2/NTF3/PALM/LEF1/PRKD1/SFRP2/SOX9/TWIST1/YWHAG/PAX8/CXCR4/SCRT1/ULK2	25
GO:0003382	epithelial cell morphogenesis	5/311	51/17046	0.00231	0.04315	0.03441	FRMD6/FLNB/MAP3K1/PAX8/COL18A1	5
GO:0051350	negative regulation of lyase activity	4/311	31/17046	0.00232	0.04315	0.03441	DRD4/GABBR1/HPCA/PALM	4
GO:1902742	apoptotic process involved in development	4/311	31/17046	0.00232	0.04315	0.03441	CYR61/LEF1/PAX8/FZD5	4

GO:0023056	positive regulation of signaling	42/311	1467/17046	0.00232	0.04315	0.03441	CDH3/TSPAN5/CDH13/HCST/CTGF/ADRB3/DRD4/ECE1/EGFR/RASA3/MTOR/GNAS/GPER1/NRG1/CYR61/IL6/ISL1/LCK/SMAD3/MAP3K1/NOV/NTF3/PIK3CG/ZDHHC13/LIMS2/PRKD1/PSMB4/CCAR2/PXN/RASGRF2/S100A4/CCL17/SFRP2/SOX9/VAMP2/TLR5/YWHAG/CXCR4/FZD5/SLA2/FADD/LY86	42
GO:0042327	positive regulation of phosphorylation	30/311	946/17046	0.00232	0.04315	0.03441	ADCY3/CTGF/ADRB3/DRD4/EGFR/RASA3/MTOR/GAPDHS/GPER1/DOK7/ANXA2/NRG1/CYR61/IL6/ISL1/LCK/MAP3K1/NTF3/ANGPT4/PIK3CG/PRKD1/PSMB4/PXN/RASGRF2/CCL17/SFRP2/SOX9/STK10/CXCR4/FZD5	30
GO:1903055	positive regulation of extracellular matrix organization	3/311	15/17046	0.00233	0.04315	0.03441	SMAD3/MFI2/SOX9	3
GO:0031324	negative regulation of cellular metabolic process	57/311	2154/17046	0.00234	0.04318	0.03443	C1D/ZBTB18/RNF168/NLRP6/DNMT3A/DRD4/PATL2/PPM1E/ACIN1/FOXO1/MTOR/GABBR1/DKK3/GPER1/GSTP1/GZMA/ANXA2/SERPIND1/NRG1/HMGA1/HPCA/HOXB3/HOXC6/ACADL/COL28A1/IL6/ISL1/ITIH3/HILS1/SMAD3/NTF3/PALM/LEF1/PIK3CG/PRMT6/MASP1/PSMB4/METTL14/CCAR2/SFRP2/SOX9/TBP/TIMP3/TWIST1/YWHAG/ZNF177/ZC3H14/SCRT1/HIST1H3A/SLA2/SPINK7/KDM2B/LOXL3/CBX2/FADD/LIMD1/H2AFY	57
GO:0051960	regulation of nervous system development	23/311	660/17046	0.00237	0.04348	0.03468	TBR1/EPHA3/SPG20/MTOR/TENM4/GPER1/NRG1/HOXB3/HOXD3/BARHL2/IL6/ISL1/NTF3/PALM/CEND1/PRKD1/SFRP2/SOX9/YWHAG/CXCR4/COLQ/SCRT1/ULK2	23
GO:0006355	regulation of transcription, DNA-templated	79/311	3210/17046	0.00237	0.04348	0.03468	CDH13/C1D/ZBTB18/DMRT2/TBR1/PSIP1/ZBED9/CIDEA/ZFP42/ZNF709/ZNF781/CITED4/RNF168/DNMT3A/EGFR/EGR3/SP8/XRN2/FOXO1/SPG20/MTOR/DKK3/GPER1/ZBTB44/GTF2B/BRF1/NRG1/HLX/HMGA1/HOXB3/HOXC5/HOXC6/HOXD3/BARHL2/CYR61/IL6/ISL1/HILS1/SMAD3/MEF2D/MEOX1/MEOX2/NFYB/NTF3/LEF1/RIPK4/CYTL1/POMC/BNC2/PRMT6/ZNF532/CNOT11/PRKD1/CCAR2/SFRP2/SGK1/SOX9/TAF4B/TBP/TCEA1/TLE3/TRAF1/TWIST1/ZNF124/ZNF177/PAX8/FZD5/ZNF606/SCRT1/HIST1H3A/SLA2/KDM2B/LOXL3/CBX2/GAS7/FADD/LIMD1/LDB2/H2AFY	79
GO:0040017	positive regulation of locomotion	15/311	357/17046	0.00243	0.0444	0.03541	CDH13/EGFR/GPER1/CYR61/IL6/SMAD3/NTF3/LEF1/ANGPT4/PRKD1/SOX9/TWIST1/PTP4A1/COL18A1/FADD	15
GO:0051241	negative regulation of multicellular organismal process	29/311	908/17046	0.00247	0.04475	0.03568	CDH3/CIDEA/ADRB3/TRPV3/VASH1/SPG20/GNAS/GSTP1/ANXA2/HLX/IL6/ISL1/SMAD3/NOV/LEF1/CEND1/ANGPT4/PIK3CG/IL20RB/POMC/LIMS2/SFRP2/SOX9/TWIST1/PAX8/NLRX1/RAB11FIP1/LIMD1/ULK2	29
GO:0051128	regulation of cellular component organization	52/311	1928/17046	0.0025	0.04475	0.03568	CDH13/TBR1/CIDEA/CTGF/COCH/PATL2/UNC13D/EPHA3/PPM1E/SPG20/MTOR/GLS2/VPS4A/GPER1/ANXA2/NRG1/HMGA1/HPCA/FMN1/BARHL2/CYR61/IL6/ISL1/SMAD3/MAP3K1/MFI2/NOV/NTF3/PALM/LEF1/PIWIL2/PRKD1/BIN3/ACTR3B/CCAR2/PXN/SFRP2/SGK1/SOX9/TWIST1/YWHAG/PAX8/FZD5/COLQ/SH3BGRL3/MGARP/GAS7/LIMD1/H2AFY/ARHGEF10/ULK2/USP6NL	52
GO:0042303	molting cycle	7/311	101/17046	0.00251	0.04475	0.03568	CDH3/APCDD1/TRPV3/EGFR/GNAS/SOX9/LDB2	7
GO:0042633	hair cycle	7/311	101/17046	0.00251	0.04475	0.03568	CDH3/APCDD1/TRPV3/EGFR/GNAS/SOX9/LDB2	7
GO:0043200	response to amino acid	7/311	101/17046	0.00251	0.04475	0.03568	CTGF/DNMT3A/EGFR/MTOR/HPCA/IL6/TIMP3	7
GO:0060828	regulation of canonical Wnt signaling pathway	11/311	221/17046	0.00252	0.0449	0.0358	FOXO1/DKK3/ISL1/SMAD3/LEF1/PSMB4/CCAR2/SFRP2/SOX9/FZD5/LIMD1	11
GO:0050673	epithelial cell proliferation	14/311	323/17046	0.00254	0.04496	0.03585	CDH3/CDH13/EGFR/EGR3/VASH1/MTOR/IL6/SMAD3/NOV/LIMS2/PRKD1/SFRP2/SOX9/TWIST1	14
GO:0060429	epithelium development	32/311	1037/17046	0.00255	0.04505	0.03592	CDH3/DMRT2/FRMD6/APCDD1/CTGF/EGFR/FLNB/GNAS/NRG1/FMN1/CYR61/IL6/ACAT1/KRT15/INSC/SMAD3/MAP3K1/MEOX1/MEOX2/NTF3/LEF1/RIPK4/PXN/SFRP2/SOX9/TWIST1/PAX8/FZD5/COL18A1/KDM2B/LDB2/H2AFY	32
GO:0014706	striated muscle tissue development	14/311	324/17046	0.00261	0.04585	0.03656	ZBTB18/COL11A1/FLNB/TENM4/NRG1/HLX/ISL1/SMAD3/MEF2D/MEOX2/LEF1/BIN3/ACTC1/TWIST1	14

GO:0048015	phosphatidylinositol-mediated signaling	10/311	190/17046	0.00262	0.04585	0.03656	HCST/EGFR/FOXO1/MTOR/GPER1/NRG1/LCK/PIK3CG/SOX9/TWIST1	10
GO:0001934	positive regulation of protein phosphorylation	29/311	913/17046	0.00268	0.04663	0.03718	ADCY3/CTGF/ADRB3/DRD4/EGFR/RASA3/MTOR/GPER1/DOK7/ANXA2/NRG1/CYR61/IL6/ISL1/LCK/MAP3K1/NTF3/ANGPT4/PIK3CG/PRKD1/PSMB4/PXN/RASGRF2/CCL17/SFRP2/SOX9/STK10/CXCR4/FZD5	29
GO:0010556	regulation of macromolecule biosynthetic process	87/311	3621/17046	0.00272	0.04727	0.03769	CDH13/C1D/ZBTB18/DMRT2/TBR1/PSIP1/ZBED9/CIDEA/ZFP42/CTGF/ZNF709/ZNF781/CITED4/RNF168/DNMT3A/EGFR/EGR3/PATL2/SP8/XRN2/FOXO1/SPG20/LARP1/MTOR/DKK3/GPER1/ZBTB44/GSTP1/GTF2B/BRF1/NRG1/HLX/HMGA1/HOXB3/HOXC5/HOXC6/HOXD3/BARHL2/CYR61/IL6/ISL1/HILS1/LTB/SMAD3/MEF2D/MEOX1/MEOX2/NFYB/NTF3/LEF1/RIPK4/CYTL1/POMC/BNC2/PIWIL2/PRMT6/ZNF532/CNOT11/PRKD1/METTL14/CCAR2/SFRP2/SGK1/SOX9/TAF4B/TBP/TCEA1/TLE3/TRAF1/TWIST1/VARS/ZNF124/ZNF177/PAX8/FZD5/ZNF606/SCRT1/HIST1H3A/SLA2/KDM2B/LOXL3/CBX2/GAS7/FADD/LIMD1/LDB2/H2AFY	87
GO:1903506	regulation of nucleic acid-templated transcription	79/311	3227/17046	0.00275	0.04749	0.03787	CDH13/C1D/ZBTB18/DMRT2/TBR1/PSIP1/ZBED9/CIDEA/ZFP42/ZNF709/ZNF781/CITED4/RNF168/DNMT3A/EGFR/EGR3/SP8/XRN2/FOXO1/SPG20/MTOR/DKK3/GPER1/ZBTB44/GTF2B/BRF1/NRG1/HLX/HMGA1/HOXB3/HOXC5/HOXC6/HOXD3/BARHL2/CYR61/IL6/ISL1/HILS1/SMAD3/MEF2D/MEOX1/MEOX2/NFYB/NTF3/LEF1/RIPK4/CYTL1/POMC/BNC2/PRMT6/ZNF532/CNOT11/PRKD1/CCAR2/SFRP2/SGK1/SOX9/TAF4B/TBP/TCEA1/TLE3/TRAF1/TWIST1/ZNF124/ZNF177/PAX8/FZD5/ZNF606/SCRT1/HIST1H3A/SLA2/KDM2B/LOXL3/CBX2/GAS7/FADD/LIMD1/LDB2/H2AFY	79
GO:0001916	positive regulation of T cell mediated cytotoxicity	3/311	16/17046	0.00282	0.04861	0.03876	HLA-B/HLA-E/FADD	3
GO:0051093	negative regulation of developmental process	24/311	710/17046	0.00284	0.04861	0.03876	CDH3/ADRB3/VASH1/FOXO1/SPG20/GNAS/GPER1/HLX/IL6/ISL1/SMAD3/MFI2/NOV/LEF1/CEND1/ANGPT4/LIMS2/CCL17/SFRP2/SOX9/TWIST1/PAX8/LIMD1/ULK2	24
GO:0010647	positive regulation of cell communication	42/311	1485/17046	0.0029	0.04948	0.03946	CDH3/TSPAN5/CDH13/HCST/CTGF/ADRB3/DRD4/EGFR/RASA3/LARP1/MTOR/GNAS/GPER1/NRG1/CYR61/IL6/ISL1/LCK/SMAD3/MAP3K1/NOV/NTF3/PIK3CG/ZDHHC13/LIMS2/PRKD1/PSMB4/CCAR2/PXN/RASGRF2/S100A4/CCL17/SFRP2/SOX9/VAMP2/TLR5/YWHAG/CXCR4/FZD5/SLA2/FADD/LY86	42
GO:0009892	negative regulation of metabolic process	62/311	2412/17046	0.00292	0.04965	0.03959	C1D/ZBTB18/CIDEA/CTGF/RNF168/NLRP6/DNMT3A/DRD4/EGFR/PATL2/PPM1E/ACIN1/FOXO1/MTOR/GABBR1/DKK3/GPER1/GSTP1/GZMA/ANXA2/SERPIND1/NRG1/HMGA1/HPCA/HOXB3/HOXC6/ACADL/COL28A1/IL6/ISL1/ITIH3/HILS1/SMAD3/NTF3/PALM/LEF1/PIK3CG/PIWIL2/PRMT6/CNOT11/MASP1/PSMB4/METTL14/CCAR2/SFRP2/SOX9/TBP/TIMP3/TWIST1/YWHAG/ZNF177/ZC3H14/SCRT1/HIST1H3A/SLA2/SPINK7/KDM2B/LOXL3/CBX2/FADD/LIMD1/H2AFY	62
GO:0048017	inositol lipid-mediated signaling	10/311	193/17046	0.00293	0.04971	0.03964	HCST/EGFR/FOXO1/MTOR/GPER1/NRG1/LCK/PIK3CG/SOX9/TWIST1	10
GO:0006139	nucleobase-containing compound metabolic process	116/311	5102/17046	0.00295	0.04974	0.03966	CDH13/MBNL2/TCIRG1/C1D/ZBTB18/DMRT2/CELF2/TBR1/NPFFR2/ADCY3/PSIP1/ZBED9/CIDEA/APOA1BP/ZFP42/CTGF/ADRB3/ADAL/ZNF709/ZNF781/CITED4/RNF168/DNMT3A/DRD4/EGFR/EGR3/PATL2/SP8/XRN2/ACIN1/FOXO1/MTOR/GABBR1/GAPDHS/AMPD2/DKK3/GLS2/GNAS/GPER1/ZBTB44/GTF2B/BRF1/NME7/GZMA/NRG1/HLX/HMGA1/HPCA/HOXB3/HOXC5/HOXC6/HOXD3/BARHL2/CYR61/IL6/ISL1/HILS1/SMAD3/ME1/MEF2D/MEOX1/MEOX2/NUDT1/NFYB/NTF3/PALM/LEF1/DDX47/PDE4C/PDE7A/PGAM2/RIPK4/CYTL1/POMC/MOV10L1/BNC2/PIWIL2/PRMT6/ZNF532/WDR33/CNOT11/PRKD1/METTL14/CCAR2/RPL8/RPL29/SFRP2/TRA2B/SGK1/SOX9/TAF4B/TBP/TCEA1/TLE3/TRAF1/TWIST1/UPP1/VARS/YWHAG/ZNF124/ZNF177/PAX8/FZD5/ZC3H14/ZNF606/SCRT1/HIST1H3A/SLA2/KDM2B/LOXL3/CBX2/GAS7/FADD/LIMD1/LDB2/H2AFY	116
**Hypermethylated DMC, Cellular Component**								
	Description	GeneRatio	BgRatio	pvalue	p.adjust	qvalue	geneID	Count

GO:0005575	cellular_component	325/325	16277/17046	2.63E-07	0.00013	0.00011	CDH3/CCDC180/TSPAN5/CDH13/MBNL2/KCNMB2/BCKDK/TCIRG1/ABCA9/C1D/ZBTB18/DMRT2/CELF2/TBR1/SEPT9/HCST/NPFFR2/ADCY3/SLC27A2/HIBADH/PSIP1/PXMP4/B4GALT7/PTH2/CHRNA5/CIDEA/ALPK2/CLCA1/FRMD6/C15orf27/ZG16B/SLC38A10/KRT40/APOA1BP/COL11A1/LYPD6B/GALM/SCLT1/ZFP42/CPD/TRPM6/LYSMD4/APCDD1/CTGF/LRRC34/CYB561/ADRB3/FITM1/ADAL/TRPV3/ZNF709/ZNF781/CITED4/WBP2NL/RNF168/COCH/NLRP6/DNAH8/DNMT3A/DRD4/DSG3/ECE1/EGFR/EGR3/PATL2/TMEM17/EML1/UNC13D/DNAH12/EPHA3/EPHB4/ALAS1/RNF182/SP8/XRN2/RASA3/PPM1E/VASH1/ACIN1/TBC1D9B/FOXO1/SPG20/FLNB/SEL1L3/ATP11A/LARP1/MTOR/FUCA1/WDR27/GABBR1/NUTM1/DSCR9/GAK/TENM4/FAM169A/RGS22/FBXO2/GAPDHS/GJA3/GJB2/AMPD2/SDCBP2/DKK3/GLS2/VPS4A/GPR162/GNAS/CRACR2B/PIGW/IZUMO1/GPER1/DOK7/DCBLD1/FLVCR1/ZBTB44/GSTP1/GTF2B/BRF1/CCDC106/NME7/GZMA/ANXA2/SERPIND1/KCNIP2/NRG1/ANXA6/HLA-B/HLA-E/HLA-F/HLX/HMGA1/HPCA/APBA2/HOXB3/HOXC5/HOXC6/HOXD3/HSD17B2/ACADL/HSP90AA1/HTR5A/COL28A1/MS4A10/FMN1/BARHL2/CYR61/IL6/ISL1/ITIH3/HILS1/ATP9B/KCP/ACAT1/KRT7/KRT15/INSC/TOMM20L/SLC6A17/RESP18/CDHR4/LCK/LLGL1/LOX/LTB/LTBP1/SMAD3/ME1/MEF2D/MAP3K1/MEOX1/MEOX2/MFI2/MT1A/NUDT1/NEDD9/NFYB/NMBR/NOV/NTF3/WRAP73/DEF6/PALM/ARHGEF3/LEF1/DDX47/CEND1/ANGPT4/PDE4C/PDE7A/GALNT7/PGAM2/PIGC/PIK3CG/SPA17/PLEC/IL20RB/RIPK4/CYTL1/POMC/PON1/MOV10L1/ZDHHC13/ROBO4/BNC2/LPCAT2/FAM118A/PPP1CC/PIWIL2/ARHGEF10L/PRMT6/ZNF532/WDR33/SMPD3/CNOT11/SYBU/LIMS2/VAC14/PRKD1/BIN3/PCDHGC4/PCDHGB7/PCDHGA11/MASP1/PSMB4/TRPC7/LPAR5/ACTR3B/METTL14/CCAR2/PTPRCAP/PXN/RASGRF2/EXOC4/RPL8/DEFB134/CTXN3/RPL29/S100A4/S100A5/S100A6/CCL17/C19orf33/SFRP2/CXCR5/TRA2B/SGK1/SYNDIG1L/MICAL1/CERK/SOX9/STK10/VAMP2/TAF4B/TBP/TCEA1/ACTC1/TIMP3/TLE3/TLR5/TRAPPC10/TNXB/TRAF1/TRPC4/PHLDA2/TWIST1/UPP1/VARS/YWHAG/ZNF124/ZNF177/PTP4A1/CACNB2/PAX8/CXCR4/FZD5/REEP5/GDPD3/TMEM204/NLRX1/ZMYM1/EPHX3/ZC3H14/ZNF606/RAB11FIP1/RNF39/COL18A1/UNC93B1/CAPS/COLQ/HIST1H2BM/SH3BGRL3/GSG1/SCRT1/HIST1H3A/SLA2/PARD6B/IL1F10/SPINK7/KDM2B/LOXL3/MGARP/CBX2/GAS7/FADD/LIMD1/MAP7/ZFAND2A/LDB2/SMDT1/RCSD1/IL32/REEP6/SDR42E1/TBC1D31/ARHGAP29/LY86/RAB3D/H2AFY/MTL5/ARHGEF10/ULK2/USP6NL/TELO2/RABGAP1L/TMCC2	325
GO:0005769	early endosome	16/325	266/17046	5.44E-05	0.0133	0.01154	ECE1/EGFR/EPHA3/ATP11A/VPS4A/GPER1/ANXA2/HLA-B/HLA-E/HLA-F/LLGL1/VAC14/PTP4A1/CXCR4/FZD5/RABGAP1L	16
GO:0031931	TORC1 complex	3/325	6/17046	0.00013	0.02145	0.01861	LARP1/MTOR/TELO2	3
GO:0030054	cell junction	37/325	1073/17046	0.00035	0.04268	0.03702	CDH3/CDH13/CHRNA5/FRMD6/DSG3/EGFR/FLNB/GABBR1/GAK/GJA3/GJB2/GPER1/DOK7/ANXA6/HMGA1/HOXC5/FMN1/SLC6A17/LCK/NEDD9/NOV/PLEC/PPP1CC/LIMS2/PRKD1/PXN/RPL8/VAMP2/ACTC1/TRPC4/YWHAG/CXCR4/FZD5/TMEM204/COLQ/PARD6B/LIMD1	37
**Hypermethylated DMC, Molecular Function**								
	Description	GeneRatio	BgRatio	pvalue	p.adjust	qvalue	geneID	Count
GO:0003674	molecular_function	312/312	15274/17046	9.59E-16	6.31E-13	5.71E-13	CDH3/TSPAN5/CDH13/MBNL2/KCNMB2/BCKDK/TCIRG1/ABCA9/C1D/ZBTB18/DMRT2/CELF2/TBR1/SEPT9/HCST/NPFFR2/ADCY3/SLC27A2/HIBADH/PSIP1/PXMP4/B4GALT7/ADPRHL1/CHRNA5/ZBED9/PWWP2A/CIDEA/ALPK2/CLCA1/FRMD6/C15orf27/ZG16B/SLC38A10/KRT40/APOA1BP/COL11A1/GALM/SCLT1/ZFP42/CPD/TRPM6/APCDD1/CTGF/LRRC34/CYB561/ADRB3/FITM1/ADAL/TRPV3/ZNF709/ZNF781/CITED4/WBP2NL/RNF168/RBM46/COCH/NLRP6/DNAH8/DNMT3A/DRD4/DSG3/ECE1/EGFR/EGR3/PATL2/EML1/UNC13D/ZBTB7C/DNAH12/EPHA3/EPHB4/ALAS1/RNF182/SP8/XRN2/RASA3/RNF44/PPM1E/VASH1/ACIN1/LIMCH1/TBC1D9B/FOXO1/SPG20/FLNB/ATP11A/KLHL18/LARP1/MTOR/FUCA1/WDR27/GABBR1/NUTM1/DSCR9/GAK/TENM4/LTN1/RGS22/FBXO2/GAPDHS/GJA3/GJB2/AMPD2/SDCBP2/GLS2/VPS4A/GPR162/GNAS/CRACR2B/PIGW/IZUMO1/GPER1/DOK7/DCBLD1/FLVCR1/ZBTB44/GSTP1/GTF2B/BRF1/CCDC106/NME7/GZMA/ANXA2/SERPIND1/KCNIP2/NRG1/ANXA6/HLA-B/HLA-E/HLA-F/HLX/HMGA1/HPCA/APBA2/HOXB3/HOXC5/HOXC6/HOXD3/HSD17B2/ACADL/HSP90AA1/HTR5A/COL28A1/FMN1/BARHL2/CYR61/IL6/ISL1/ITIH3/HILS1/ATP9B/ACAT1/KRT7/KRT15/SLC6A17/CDHR4/LCK/LLGL1/LOX/ZNF833P/LTB/LTBP1/SMAD3/ME1/MEF2D/MAP3K1/MEOX1/MEOX2/MFI2/MT1A/NUDT1/NEDD9/NFYB/NMBR/NOV/NTF3/DEF6/PALM/ARHGEF3/LEF1/DDX47/ANGPT4/PDE4C/PDE7A/GALNT7/PGAM2/PIGC/PIK3CG/SPA17/PLEC/IL20RB/RIPK4/CYTL1/POMC/PON1/MOV10L1/ZDHHC13/ROBO4/BORCS6/BNC2/LPCAT2/FAM118A/PPP1CC/PIWIL2/ARHGEF10L/PRMT6/ZNF532/WDR33/SMPD3/CNOT11/SYBU/LIMS2/VAC14/PRKD1/BIN3/PCDHGC4/PCDHGB7/PCDHGA11/MASP1/PSMB4/TRPC7/LPAR5/ACTR3B/METTL14/CCAR2/PXN/RASGRF2/EXOC4/RPL8/RPL29/S100A4/S100A5/S100A6/CCL17/SFRP2/CXCR5/TRA2B/SGK1/MICAL1/CERK/SOX9/STK10/VAMP2/TAF4B/TBP/TCEA1/ACTC1/TIMP3/TLE3/TLR5/TRAPPC10/TNXB/TRAF1/TRPC4/TWIST1/UPP1/VARS/ZNHIT2/YWHAG/ZNF124/ZNF177/PTP4A1/CACNB2/PAX8/CXCR4/FZD5/REEP5/GDPD3/NLRX1/ZMYM1/EPHX3/ZC3H14/ZNF606/RAB11FIP1/RNF39/COL18A1/CAPS/COLQ/HIST1H2BM/SH3BGRL3/GSG1/SCRT1/HIST1H3A/SLA2/PARD6B/IL1F10/SPINK7/KDM2B/LOXL3/MGARP/CBX2/GAS7/FADD/LIMD1/THAP3/MAP7/ZFAND2A/LDB2/RCSD1/IL32/REEP6/SDR42E1/ARHGAP29/RAB3D/H2AFY/MTL5/ARHGEF10/ULK2/USP6NL/TELO2/RABGAP1L/TMCC2	312

GO:0005488	binding	285/312	12915/17046	6.51E-13	2.14E-10	1.94E-10	CDH3/TSPAN5/CDH13/MBNL2/BCKDK/TCIRG1/ABCA9/C1D/ZBTB18/DMRT2/CELF2/TBR1/SEPT9/HCST/NPFFR2/ADCY3/SLC27A2/HIBADH/PSIP1/PXMP4/B4GALT7/ADPRHL1/CHRNA5/ZBED9/PWWP2A/CIDEA/ALPK2/CLCA1/FRMD6/ZG16B/KRT40/APOA1BP/COL11A1/GALM/SCLT1/ZFP42/CPD/TRPM6/APCDD1/CTGF/CYB561/ADRB3/ADAL/ZNF709/ZNF781/WBP2NL/RNF168/RBM46/COCH/NLRP6/DNAH8/DNMT3A/DRD4/DSG3/ECE1/EGFR/EGR3/PATL2/EML1/UNC13D/ZBTB7C/DNAH12/EPHA3/EPHB4/ALAS1/RNF182/SP8/XRN2/RASA3/RNF44/PPM1E/VASH1/ACIN1/LIMCH1/TBC1D9B/FOXO1/SPG20/FLNB/ATP11A/KLHL18/LARP1/MTOR/WDR27/GABBR1/NUTM1/GAK/TENM4/LTN1/FBXO2/GAPDHS/GJA3/AMPD2/SDCBP2/GLS2/VPS4A/GNAS/CRACR2B/IZUMO1/GPER1/DOK7/DCBLD1/FLVCR1/ZBTB44/GSTP1/GTF2B/BRF1/CCDC106/NME7/GZMA/ANXA2/SERPIND1/KCNIP2/NRG1/ANXA6/HLA-B/HLA-E/HLA-F/HLX/HMGA1/HPCA/APBA2/HOXB3/HOXC5/HOXC6/HOXD3/ACADL/HSP90AA1/FMN1/BARHL2/CYR61/IL6/ISL1/HILS1/ATP9B/ACAT1/KRT7/KRT15/CDHR4/LCK/LLGL1/LOX/ZNF833P/LTB/LTBP1/SMAD3/ME1/MEF2D/MAP3K1/MEOX1/MEOX2/MFI2/MT1A/NUDT1/NEDD9/NFYB/NOV/NTF3/DEF6/PALM/ARHGEF3/LEF1/DDX47/ANGPT4/PDE4C/PDE7A/GALNT7/PGAM2/PIK3CG/SPA17/PLEC/IL20RB/RIPK4/CYTL1/POMC/PON1/MOV10L1/ZDHHC13/BORCS6/BNC2/LPCAT2/FAM118A/PPP1CC/PIWIL2/PRMT6/ZNF532/WDR33/SMPD3/CNOT11/SYBU/LIMS2/VAC14/PRKD1/BIN3/PCDHGC4/PCDHGB7/PCDHGA11/MASP1/PSMB4/TRPC7/ACTR3B/METTL14/CCAR2/PXN/RASGRF2/EXOC4/RPL8/RPL29/S100A4/S100A5/S100A6/CCL17/SFRP2/CXCR5/TRA2B/SGK1/MICAL1/CERK/SOX9/STK10/VAMP2/TAF4B/TBP/TCEA1/ACTC1/TIMP3/TLE3/TLR5/TRAPPC10/TNXB/TRAF1/TRPC4/TWIST1/VARS/ZNHIT2/YWHAG/ZNF124/ZNF177/CACNB2/PAX8/CXCR4/FZD5/REEP5/GDPD3/NLRX1/ZMYM1/ZC3H14/ZNF606/RAB11FIP1/RNF39/COL18A1/CAPS/COLQ/HIST1H2BM/SH3BGRL3/GSG1/SCRT1/HIST1H3A/SLA2/PARD6B/IL1F10/SPINK7/KDM2B/LOXL3/MGARP/CBX2/GAS7/FADD/LIMD1/THAP3/MAP7/ZFAND2A/LDB2/RCSD1/IL32/REEP6/ARHGAP29/RAB3D/H2AFY/MTL5/ARHGEF10/ULK2/USP6NL/TELO2/RABGAP1L/TMCC2	285
GO:0005515	protein binding	222/312	9755/17046	2.10E-07	4.60E-05	4.16E-05	TSPAN5/CDH13/BCKDK/TCIRG1/C1D/ZBTB18/DMRT2/SEPT9/HCST/NPFFR2/ADCY3/SLC27A2/PSIP1/PXMP4/B4GALT7/CHRNA5/PWWP2A/CIDEA/FRMD6/KRT40/APOA1BP/COL11A1/SCLT1/TRPM6/APCDD1/CTGF/CYB561/ADRB3/WBP2NL/RNF168/COCH/DNMT3A/DRD4/ECE1/EGFR/PATL2/EML1/UNC13D/EPHA3/EPHB4/ALAS1/RNF182/PPM1E/VASH1/ACIN1/LIMCH1/TBC1D9B/FOXO1/SPG20/FLNB/ATP11A/KLHL18/LARP1/MTOR/WDR27/GABBR1/NUTM1/GAK/TENM4/LTN1/FBXO2/GAPDHS/GJA3/AMPD2/SDCBP2/GLS2/VPS4A/GNAS/CRACR2B/IZUMO1/GPER1/DOK7/FLVCR1/ZBTB44/GSTP1/GTF2B/BRF1/CCDC106/NME7/GZMA/ANXA2/KCNIP2/NRG1/ANXA6/HLA-B/HLA-E/HLA-F/HLX/HMGA1/HPCA/APBA2/HOXD3/HSP90AA1/FMN1/BARHL2/CYR61/IL6/ISL1/HILS1/ACAT1/KRT7/KRT15/LCK/LLGL1/LOX/LTB/LTBP1/SMAD3/MEF2D/MAP3K1/MEOX1/MEOX2/MFI2/MT1A/NUDT1/NEDD9/NFYB/NOV/NTF3/DEF6/PALM/ARHGEF3/LEF1/DDX47/ANGPT4/PDE4C/PIK3CG/SPA17/PLEC/IL20RB/RIPK4/CYTL1/POMC/PON1/BORCS6/FAM118A/PPP1CC/PRMT6/CNOT11/SYBU/LIMS2/VAC14/PRKD1/BIN3/MASP1/PSMB4/TRPC7/ACTR3B/CCAR2/PXN/RASGRF2/EXOC4/S100A4/S100A5/S100A6/CCL17/SFRP2/CXCR5/TRA2B/SGK1/MICAL1/CERK/SOX9/STK10/VAMP2/TAF4B/TBP/TCEA1/ACTC1/TIMP3/TLE3/TLR5/TRAPPC10/TNXB/TRAF1/TRPC4/TWIST1/VARS/YWHAG/ZNF124/ZNF177/CACNB2/PAX8/CXCR4/FZD5/REEP5/NLRX1/ZMYM1/ZC3H14/RAB11FIP1/COL18A1/COLQ/HIST1H2BM/SH3BGRL3/GSG1/HIST1H3A/SLA2/PARD6B/IL1F10/SPINK7/KDM2B/LOXL3/MGARP/CBX2/GAS7/FADD/LIMD1/THAP3/MAP7/LDB2/RCSD1/IL32/REEP6/ARHGAP29/RAB3D/H2AFY/ARHGEF10/ULK2/USP6NL/TELO2/RABGAP1L/TMCC2	222
GO:0043167	ion binding	143/312	5637/17046	1.54E-06	0.00025	0.00023	CDH3/CDH13/MBNL2/BCKDK/ABCA9/ZBTB18/DMRT2/SEPT9/ADCY3/SLC27A2/B4GALT7/ADPRHL1/CHRNA5/ALPK2/CLCA1/APOA1BP/COL11A1/ZFP42/CPD/TRPM6/CTGF/CYB561/ADAL/ZNF709/ZNF781/RNF168/NLRP6/DNAH8/DNMT3A/DRD4/DSG3/ECE1/EGFR/EGR3/EML1/ZBTB7C/DNAH12/EPHA3/EPHB4/ALAS1/RNF182/SP8/XRN2/RASA3/RNF44/PPM1E/LIMCH1/TBC1D9B/ATP11A/MTOR/GAK/LTN1/AMPD2/VPS4A/GNAS/CRACR2B/ZBTB44/GTF2B/BRF1/NME7/ANXA2/SERPIND1/KCNIP2/ANXA6/HPCA/ACADL/HSP90AA1/CYR61/ISL1/ATP9B/ACAT1/CDHR4/LCK/LOX/ZNF833P/LTBP1/SMAD3/ME1/MAP3K1/MFI2/MT1A/NUDT1/NOV/DDX47/PDE4C/PDE7A/GALNT7/PIK3CG/RIPK4/PON1/MOV10L1/ZDHHC13/BNC2/LPCAT2/PPP1CC/ZNF532/SMPD3/LIMS2/PRKD1/PCDHGC4/PCDHGB7/PCDHGA11/MASP1/TRPC7/ACTR3B/PXN/RPL29/S100A4/S100A5/S100A6/SGK1/MICAL1/CERK/STK10/VAMP2/TCEA1/ACTC1/TIMP3/TNXB/TRAF1/TRPC4/VARS/ZNHIT2/ZNF124/ZNF177/GDPD3/NLRX1/ZMYM1/ZC3H14/ZNF606/RNF39/COL18A1/CAPS/SCRT1/KDM2B/LOXL3/LIMD1/THAP3/ZFAND2A/ARHGAP29/RAB3D/MTL5/ULK2	143
GO:0043169	cation binding	105/312	3856/17046	4.59E-06	0.0006	0.00055	CDH3/CDH13/MBNL2/ZBTB18/DMRT2/ADCY3/B4GALT7/ADPRHL1/CHRNA5/CLCA1/APOA1BP/COL11A1/ZFP42/CPD/TRPM6/CYB561/ADAL/ZNF709/ZNF781/RNF168/DNMT3A/DRD4/DSG3/ECE1/EGR3/EML1/ZBTB7C/RNF182/SP8/XRN2/RASA3/RNF44/PPM1E/LIMCH1/TBC1D9B/ATP11A/LTN1/AMPD2/GNAS/CRACR2B/ZBTB44/GTF2B/BRF1/NME7/ANXA2/KCNIP2/ANXA6/HPCA/ISL1/ATP9B/ACAT1/CDHR4/LOX/ZNF833P/LTBP1/SMAD3/ME1/MAP3K1/MFI2/MT1A/NUDT1/PDE4C/PDE7A/GALNT7/PON1/MOV10L1/ZDHHC13/BNC2/LPCAT2/PPP1CC/ZNF532/SMPD3/LIMS2/PRKD1/PCDHGC4/PCDHGB7/PCDHGA11/MASP1/PXN/S100A4/S100A5/S100A6/MICAL1/CERK/TCEA1/TIMP3/TRAF1/ZNHIT2/ZNF124/ZNF177/GDPD3/ZMYM1/ZC3H14/ZNF606/RNF39/COL18A1/CAPS/SCRT1/KDM2B/LOXL3/LIMD1/THAP3/ZFAND2A/ARHGAP29/MTL5	105
GO:0046872	metal ion binding	103/312	3777/17046	5.61E-06	0.00061	0.00056	CDH3/CDH13/MBNL2/ZBTB18/DMRT2/ADCY3/B4GALT7/ADPRHL1/CLCA1/APOA1BP/COL11A1/ZFP42/CPD/TRPM6/CYB561/ADAL/ZNF709/ZNF781/RNF168/DNMT3A/DSG3/ECE1/EGR3/EML1/ZBTB7C/RNF182/SP8/XRN2/RASA3/RNF44/PPM1E/LIMCH1/TBC1D9B/ATP11A/LTN1/AMPD2/GNAS/CRACR2B/ZBTB44/GTF2B/BRF1/NME7/ANXA2/KCNIP2/ANXA6/HPCA/ISL1/ATP9B/ACAT1/CDHR4/LOX/ZNF833P/LTBP1/SMAD3/ME1/MAP3K1/MFI2/MT1A/NUDT1/PDE4C/PDE7A/GALNT7/PON1/MOV10L1/ZDHHC13/BNC2/LPCAT2/PPP1CC/ZNF532/SMPD3/LIMS2/PRKD1/PCDHGC4/PCDHGB7/PCDHGA11/MASP1/PXN/S100A4/S100A5/S100A6/MICAL1/CERK/TCEA1/TIMP3/TRAF1/ZNHIT2/ZNF124/ZNF177/GDPD3/ZMYM1/ZC3H14/ZNF606/RNF39/COL18A1/CAPS/SCRT1/KDM2B/LOXL3/LIMD1/THAP3/ZFAND2A/ARHGAP29/MTL5	103
GO:0005102	receptor binding	42/312	1310/17046	0.00026	0.02482	0.02248	C1D/HCST/NPFFR2/SLC27A2/CTGF/ADRB3/GNAS/IZUMO1/DOK7/GTF2B/NRG1/HLA-B/HLA-E/HLA-F/HMGA1/CYR61/IL6/ISL1/LCK/LTB/SMAD3/NOV/NTF3/PALM/LEF1/ANGPT4/PIK3CG/CYTL1/POMC/PXN/S100A4/CCL17/SFRP2/TLR5/TNXB/TRAF1/YWHAG/SH3BGRL3/IL1F10/FADD/MAP7/IL32	42
